# Origin and Consequences of Chromosomal Inversions in the *virilis* Group of *Drosophila*

**DOI:** 10.1093/gbe/evy239

**Published:** 2018-10-30

**Authors:** Micael Reis, Cristina P Vieira, Rodrigo Lata, Nico Posnien, Jorge Vieira

**Affiliations:** 1 Instituto de Investigação e Inovação em Saúde, Universidade do Porto, Portugal; 2 Instituto de Biologia Molecular e Celular (IBMC), Universidade do Porto, Portugal; 3 Johann-Friedrich-Blumenbach-Institut für Zoologie und Anthropologie, Abteilung für Entwicklungsbiologie, GZMB Ernst-Caspari-Haus, Universität Göttingen, Germany

**Keywords:** chromosomal inversions, ectopic recombination, *Drosophila americana*, *Drosophila novamexicana*

## Abstract

In *Drosophila*, large variations in rearrangement rate have been reported among different lineages and among Muller’s elements. Nevertheless, the mechanisms that are involved in the generation of inversions, their increase in frequency, as well as their impact on the genome are not completely understood. This is in part due to the lack of comparative studies on species distantly related to *Drosophila melanogaster.* Therefore, we sequenced and assembled the genomes of two species of the *virilis* phylad (*Drosophila novamexicana* [15010-1031.00] and *Drosophila americana* [SF12]), which are diverging from *D. melanogaster* for more than 40 Myr. Based on these data, we identified the precise location of six novel inversion breakpoints. A molecular characterization provided clear evidence that DAIBAM (a miniature inverted–repeat transposable element) was involved in the generation of eight out of the nine inversions identified. In contrast to what has been previously reported for *D. melanogaster* and close relatives, ectopic recombination is thus the prevalent mechanism of generating inversions in species of the *virilis* phylad. Using pool-sequencing data for three populations of *D. americana*, we also show that common polymorphic inversions create a high degree of genetic differentiation between populations for chromosomes *X*, *4*, and *5* over large physical distances. We did not find statistically significant differences in expression levels between *D. americana* (SF12) and *D. novamexicana* (15010-1031.00) strains for the three genes surveyed (*CG9588*, *Fig 4*, and *fab1*) flanking three inversion breakpoints.

## Introduction

Chromosomal inversions are common in many groups of animals, although the production of nonfunctional gametes during meiosis in individuals heterozygous for such rearrangements may lead to reduction in fertility (Powell 1997). In *Drosophila*, inversions are widespread because recombination is generally suppressed in males, and in females aberrant recombinant products preferentially contribute to the polar body nurse cells. Therefore, the expected reduction in fertility is not so obvious in the *Drosophila* genus (Powell 1997), making this group of species ideal to study the mechanisms that are involved in the generation of inversions, their increase in frequency, as well as their impact on the genome.

Chromosomal inversions can be generated by chromosomal breakage and erroneous repair of the free ends by nonhomologous end-joining (Sonoda et al. 2006), and this is the prevalent mechanism observed in *Drosophilamelanogaster* and its close relatives (Ranz et al. 2007). However, chromosomal inversions can also be generated by ectopic recombination (or nonallelic homologous recombination) between multiple copies of DNA sequences present in the same chromosome in opposite directions. These DNA sequences can be transposable elements (TEs) (Kupiec and Petes 1988; Lim and Simmons 1994; Delprat et al. 2009; Rius et al. 2013), segmental duplications, or short repeat sequences (Richards et al. 2005; Caceres et al. 2007; Coulibaly et al. 2007). In *Drosophila*, TEs were implicated in the generation of chromosomal inversions in *Drosophilabuzzatii* (Caceres et al. 1999; Casals et al. 2003; Delprat et al. 2009), *Drosophilaamericana* (Evans et al. 2007; Fonseca et al. 2012), *Drosophilavirilis* (Fonseca et al. 2012), as well as in *Drosophilamojavensis* and *Drosophilauniseta* (Rius et al. 2013).

Large variations in chromosomal inversions rate have been reported among different lineages of *Drosophila* (Powell 1997; Bartolome and Charlesworth 2006; Papaceit et al. 2006; Gonzalez et al. 2007; Ranz et al. 2007; Bhutkar et al. 2008) and between Muller’s elements (Powell 1997; Vieira et al. 1997; Papaceit et al. 2006). Differences in mutation rate and fitness effects of chromosomal inversions have mostly been proposed as explanations for the observed discrepancies. Chromosomal inversions may increase in frequency in populations due to direct mutational effects associated with their breakpoints (the “position effect” hypothesis) (Sperlich and Pfreim 1986). According to Guillen and Ruiz (2012), this is a likely effect given the high gene density and compact structure of *Drosophila* genomes. The analysis of the genes around inversion breakpoints may, thus, reveal the targets of selection (Guillen and Ruiz 2012). Nevertheless, chromosomal inversions reduce recombination to some extent around inversion breakpoints (Navarro et al. 1997; Evans et al. 2007) which may also help keeping alleles together at loci with epistatic effects on fitness (the “coadaptation” hypothesis) (Dobzhansky 1970). Moreover, inversions may capture locally adapted sets of genes protecting them from recombination with immigrant chromosomes (Kirkpatrick and Barton 2006; Kirkpatrick 2010), which may facilitate population differentiation and ultimately lead to speciation (Noor et al. 2001; Feder et al. 2011; McGaugh and Noor 2012).

**Fig. 1.**
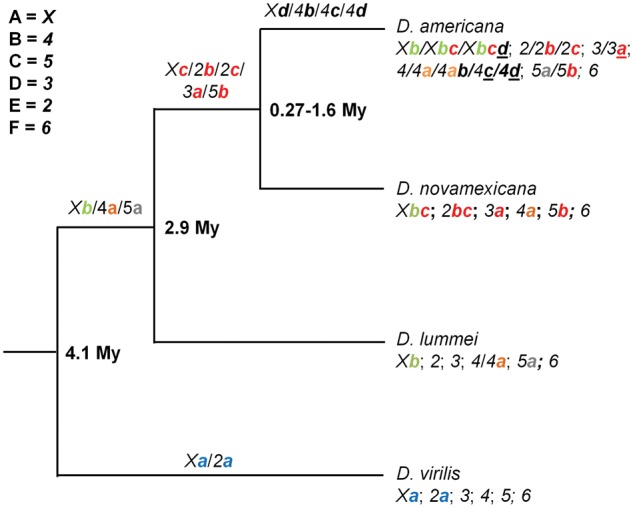
—Schematic representation of the inversions present in species of the *virilis* phylad. Inversions which occurred in the *D. virilis* lineage are shown in blue (*Xa* [Fonseca et al. 2012] and *2a* [Throckmorton 1982]). Inversion *Xb* (green) occurred before the split between *D. lummei* and the *americana* complex (*D. novamexicana* and *D. americana*), and it is fixed in the *americana* complex (Hsu 1952). Inversion *4a* (orange) occurred before the split between *D. lummei* and the *americana* complex, it is polymorphic in *D. americana*, and it is fixed in *D. novamexicana* (Hsu 1952). Inversion *5a* (gray) occurred before the split between *D. lummei* and the *americana* complex, it is polymorphic in *D. americana*, but it is absent in *D. novamexicana* (Hsu 1952). Inversions showed in red occurred after the split between *D. lummei* and the *americana* complex, and they are polymorphic in *D. americana* and fixed in *D. novamexicana* (Hsu 1952). All the other inversions (black) occurred in the *D. americana* lineage, and the underlined inversions have frequencies below 5% (Hsu 1952). Inversion *4d* was reported by Throckmorton (1982) but was not identified by Hsu (1952). The age estimates are based on Spicer and Bell (2002), Caletka and McAllister (2004), Morales-Hojas et al. (2006), Vieira et al. (2006), and Morales-Hojas et al. (2011). The Muller element equivalents for the *virilis* phylad chromosomes are shown on the upper left.

Our current understanding of the molecular basis of the generation and evolution of chromosomal inversions is still limited, mostly due to the lack of extensive comparative studies involving distantly related species. Species of the *virilis* group are diverging from *D. melanogaster* for at least 40 Myr (Morales-Hojas and Vieira 2012), and thus are suitable to test the generality of the observations made for *D. melanogaster* and closely related species. This group is divided into two large phylads, namely the *montana* and the *virilis* phylads, and consists of 13 recognized species (Morales-Hojas et al. 2011). Species of the *virilis* phylad (*D. virilis*, *Drosophilalummei*, *Drosophilanovamexicana*, and *D. americana*) have been diverging for <4.1 Myr but show contrasting patterns regarding inversions. There are 14 chromosomal inversions in species of the *virilis* phylad located on Muller’s elements A (*Xa*, *Xb*, *Xc*, and *Xd*), B (*4a*, *4 b*, *4c*, and *4d*), C (*5a* and *5b*), D (*3a*), and E (*2a*, *2 b*, and *2c*) (Hsu 1952; Throckmorton 1982) (fig. 1). Inversions *Xa*, *Xb*, and *2a* are fixed between *D. americana*/*D. novamexicana* and *D. virilis* (Throckmorton 1982). Inversions *Xc*, *2 b*, *2c*, *3a*, *4a*, and *5 b* that are apparently fixed between *D. virilis* and *D. novamexicana* are polymorphic in *D. americana.* Inversions *Xc*, *2 b, 4a*, and *4 b* are present at different frequencies in *D. americana* northwestern, central, and southern populations and all have estimated frequencies higher than 5% in this species as a whole or among populations. Inversions *Xd*, *2c*, *3a*, and *4c* show estimated frequencies lower than 5% and are present in northwestern *D. americana* populations only (Hsu 1952). Throckmorton (1982) reported the presence of an additional polymorphic inversion (*4d*) in this species that is probably rare because it was not identified by Hsu (1952). Inversion *Xc* overlaps the distal part of *Xb* inversion, and the small inversion *Xd* is included both in *Xb* and *Xc*. Inversion *4 b* is a small inverted segment completely included in the *4a* inversion, and it is only found in *4a* inverted chromosomes. The other inversions are nonoverlapping when they co-occur (Hsu 1952). Based on a large number of strains, no polymorphic inversions have been described for *D. virilis* (Hsu 1952). No polymorphic inversions have been reported for *D. novamexicana* as well, and a single polymorphic inversion has been reported for *D. lummei*, although in both cases very few strains have been analyzed (Hsu 1952; Throckmorton 1982). Inversions *Xb*, *4a*, and *5a* are present in *D. lummei* (Throckmorton 1982) and thus must be older than the *D. lummei*/*D. americana* lineage split (2.9 Myr) (Morales-Hojas et al. 2011). All inversions mentioned here are younger than the common ancestor of *virilis* and the other species that lived 4.1 Ma (Morales-Hojas et al. 2011). The *Xc* inversion is in between 0.27 and 1.6 Myr old (Spicer and Bell 2002; Caletka and McAllister 2004; Vieira et al. 2006; Morales-Hojas et al. 2008, 2011).

The *DAIBAM* miniature inverted–repeat transposable element (MITE) has been shown to be involved in the origin of *D. americana* polymorphic inversions *4a* (Evans et al. 2007) and *5a* (Fonseca et al. 2012), as well as the *Xa* inversion that is fixed between *D. virilis* and *D. americana* (Fonseca et al. 2012). Therefore, *DAIBAM* is responsible for at least 20% of the chromosomal inversions that are observed within and between species of the *virilis* phylad (Fonseca et al. 2012). These observations suggest that ectopic recombination between TEs may be the prevalent mechanism underlying inversion generation in this group of species. This mechanism is very different from that reported in *D. melanogaster* and close relatives and might be the cause of the observed discrepancies regarding chromosomal inversion rates in the *virilis* phylad. Nevertheless, only few chromosomal inversions in this phylad have been characterized in detail. The characterization of the remaining inversions will contribute to a more comprehensive understanding of the origin, rise to high frequency, and consequences of inversions at the genomic scale. Therefore, we sequenced the genome of a *D. americana* strain from the south of the distribution (SF12), as well as the genome of one *D. novamexicana* strain (15010-1031.00). In addition, we obtained pool-sequencing (pool-seq) data for northwestern, central, and southern populations of *D. americana* to characterize the impact of chromosomal rearrangements on differentiation patterns at the genome level.

## Materials and Methods

### Genome Sequencing, Assembly, and Inversion Breakpoint Identification

Genomic DNA was isolated from one set of ten females for both *D. americana* (strain: SF12, established with a single female collected in the summer of 2010 in Saint Francisville, LA) and *D. novamexicana* (strain: 15010-1031.00, obtained from the Tucson stock center in 1995 and kept in the lab since then, originally collected in Colorado) using a phenol–chloroform extraction protocol. DNA integrity was checked by gel electrophoresis. Library preparation and sequencing was performed at the Transcriptome Analysis Laboratory of the University of Göttingen. Briefly, 1 µg of genomic DNA was quantified using Quanti Flour (Promega) and diluted in 100 µl of TE Buffer. DNA was sonicated to 350 bp using the Nano NGS-Bioruptor (Diagenode). Library preparation was performed using 100 ng of sonicated DNA using the TruSeq DNA Nano Library Preparation Kit from Illumina (Cat. No. FC-121-4001). Libraries’ sizes were evaluated using the Bioanalyzer 2100 (Agilent), and the quantities were estimated using the Quanti Flour (Promega). Libraries were diluted to 8 pM and sequenced with the MiSeq System (MS-102-2003) using the 500 cycle kit and v3 Reagents (2 × 250 bp).

We started the analyses with two FASTQ files for each species, totaling 13,652,502 and 15,705,603 paired-end reads for *D. americana* (SF12) and *D. novamexicana* (15010-1031.00), respectively (raw reads available in ENA ERR2610691 and ERR2610692). The read quality was evaluated using FastQC v0.11.1 and appropriate read trimming and masking was achieved (http://www.bioinformatics.babraham.ac.uk/projects/fastqc/) using FASTQ-tools v0.8 (Blankenberg et al. 2010), as implemented in a locally installed Galaxy platform (using default settings) (Giardine et al. 2005). As for both *D. americana* (SF12) and *D. novamexicana* (15010-1031.00) a large fraction of the pair-ended reads overlap, we used Flash v1.2.11 (Magoc and Salzberg 2011) to obtain consensus sequences (16,702,093 and 19,372,252 consensus sequences for *D. americana* [SF12] and *D. novamexicana* [15010-1031.00], respectively).

To obtain the best assembly possible for the *D. novamexicana* 15010-1031.00 and *D. americana* SF12 genomes, three de novo assemblers were used, and different K-mers tested: Velvet v1.1 (K-mers used: 19, 21, 23, 25, 27, 29, 31, 109, 119, 129, 139) (Zerbino and Birney 2008), SOAPdenovo v2.04 (K-mers used: 31, 63, 95, 127) (Luo et al. 2012), and ABySS v1.5.2, (K-mers used: 32, 64, 96, 128, 160) (Simpson et al. 2009). Of the 20 test assemblies obtained for each of the genomes, the one using ABySS and a K-mer of 160 gave the best results for the N20, N50, N80, and maximum contig size statistics (data not shown). SSPACE v3.0 (default settings) (Boetzer et al. 2011) was used to grow the ABySS with a K-mer of 160 generated scaffolds even further. Only scaffolds larger than 500 bp were saved. Genome assemblies are available at ENA ERZ655455 and ERZ655456.

The quality of the obtained draft genomes compares well with the previously published *D. americana* (H5 and W11) draft genomes (supplementary table 1, Supplementary Material online) that were estimated to cover about 80% of the *D. americana* euchromatic genome (Fonseca et al. 2013). Further support for the comparable quality of these draft genomes comes from the annotation based on the *D. virilis* 20,302 coding sequences (CDS) that are encoded by 13,374 genes (http://flybase.org FB2017_05). Using these CDS as reference, and the Splign–Compart pipeline as implemented in BDBM (http://sing.ei.uvigo.es/BDBM/index.html), we were able to annotate 14,487 complete CDS in *D. novamexicana* (15010-1031.00) as well as 13,329, 13,878, and 12,007 complete CDS in the *D. americana* (SF12, H5, and W11) genomes, respectively (supplementary table 2, Supplementary Material online). The completeness of the *D. novamexicana* (15010-1031.00) and *D. americana* (SF12) genomes assembled here is thus similar to that of the *D. americana* (H5 and W11) draft genomes. Under the assumption that all CDS could be annotated this way, this analysis suggests that the genomes considered here cover between 59% and 71% of the genome. We also assessed assembly completeness for *D. novamexicana* (15010-1031.00) and *D. americana* (SF12) with BUSCO v3.0.2 (Simão et al. 2015; Waterhouse et al. 2017) employing NCBI-BLAST v2.6.0 (Camacho et al. 2009), HMMER v3.1b2 (Eddy 2011), and AUGUSTUS v3.2.2 (Keller et al. 2011). Default settings and the Diptera OrthoDB v9 database (Zdobnov et al. 2017) were used. A total of 2,799 BUSCO groups were searched; 97.0% and 92.3% of the BUSCOs were complete and only 0.3% and 0.4% were duplicated in *D. novamexicana* (15010-1031.00) and *D. americana* (SF12) assemblies, respectively. The percentage of fragmented BUSCOs was 2.0% and 6.1%, while only 1.0% and 1.6% of the BUSCOs were missing in *D. novamexicana* (15010-1031.00) and *D. americana* (SF12) assemblies, respectively.

Inversion breakpoints were identified using an alignment approach. The expected karyotypes of *D. novamexicana* (15010-1031.00) and *D. americana* (SF12) are *Xbc*; *2bc*; *3a*; *4a*; *5b*; *6* and *Xb*; *2*; *3*; *4*; *5a*; *6*, respectively. As the expected karyotype of *D. virilis* is *Xa*; *2a*; *3*; *4*; *5*; 6, we used the assembly of *D. novamexicana* (15010-1031.00) to locate inversions *Xc*, *2 b*, *2c*, *3a*, *4a*, and *5 b*, the assembly of *D. americana* (SF12) to locate inversion *5a*, and both assemblies to locate inversions *Xa*, *Xb*, and *2a*. The scaffolds obtained for *D. novamexicana* (15010-1031.00) and *D. americana* (SF12) were ordered using the program Mauve Contig Mover implemented in Mauve v2.4.0 (Rissman et al. 2009) with the *D. virilis* sequence of each chromosome (Clark et al. 2007) as references. These chromosomes were obtained by using the *D. virilis* anchored scaffolds (Schaeffer et al. 2008). Moreover, we relocated the region corresponding to the beginning of scaffold 12875 from position 1 to 1.7 Mb on the *5th* chromosome based on information provided by Schaeffer et al. (2008). Then, the alignment was manually inspected and the contigs which aligned in two different regions of the same chromosome were identified. The presence of a breakpoint was further confirmed by performing local BlastN (Camacho et al. 2009) or BlastN of these contigs against the *D. virilis* genome (http://flybase.org/FB2017_05) and the sequences annotated (supplementary files 1–10, Supplementary Material online).

### Pool-seq and Population Differentiation Analyses

To estimate differentiation levels between different populations of *D. americana*, we selected a total of 70 strains from different geographical origins in the United States. We used 25 isofemale lines from the northwest, 23 from the center, and 22 from the south of the distribution. The northwestern lines were established with flies collected at the end of July 2008 in Freemont, Nebraska, while the lines from the center were established with flies collected in the summer of 2004 in Howell Island and Lake Wappapelo, MO. The southern strains were established with flies collected in the late spring of 2005 in Corney Bayou and Cat Island, LA, as well as in the summer of 2010 at Pearl River, MS by Bryant McAllister (Iowa University, Iowa City, IA) who kindly sent us the strains. In addition to these lines, we also used strains established with flies collected in the summer of 2010 in Saint Francisville, LA. We used two females of each strain to prepare three independent pools (North, Center, and South) containing flies from northwestern, central, and southern populations, respectively. The genomic DNA was isolated using a phenol–chloroform extraction protocol. DNA integrity was checked by gel electrophoresis. Library preparation and sequencing was performed at the Beijing Genomics Institute. Illumina paired-end libraries with an average fragment size of 300 bp were generated following the instructions of the Illumina Paired-End Sample Preparation Guide (Illumina, San Diego, CA) and sequenced (2 × 101 bp). We started the analysis with 12 FASTQ files (four for each population) totaling 49,903,200, 58,574,986, and 45,780,837 paired-end reads for the samples from the north, center, and south, respectively. The raw reads are available at ENA ERR2610693, ERR2610694, and ERR2610695. Quality checks and appropriate read trimming were performed as described above.

In order to obtain a reference genome to map the reads, we reordered all *D. americana* (SF12) contigs using Mauve Contig Mover implemented in Mauve v2.4.0 (Rissman et al. 2009) and each of the estimated ancestral chromosomes with *D. virilis* sequences. The estimated ancestral chromosomes were obtained by using the *D. virilis* anchored scaffolds (Schaeffer et al. 2008), as described above, and by reordering the segments corresponding to inversions *Xa* and *2a* which occurred in the lineage leading to *D. virilis* (see Results; supplementary figs. 1 and 4, Supplementary Material online). After reordering, we identified the contigs aligning with each chromosome and removed the others. Then, these contigs were concatenated using Unipro UGENE (Okonechnikov et al. 2012) to get the estimated *D. americana* (SF12) chromosomes. We introduced 100 Ns between putative adjacent contigs, to preserve the original information obtained from the assembly.

Reads obtained for each of the three populations were mapped against the reference genome using Bowtie2 v2.2.5 (Langmead and Salzberg 2012) using default settings. The overall alignment rates for northwestern, central and southern populations were 80%, 81% and 79%, respectively. We have further mapped the reads using BWA v0.7.12 (Li and Durbin 2009) with default settings, because it has been reported that the intersection of more than one mapping algorithm efficiently reduces the amount of false positives (Kofler et al. 2016). Ambiguously mapped reads (quality below 20) and putative optical duplicates were removed with SAMtools v1.3.1 (Li et al. 2009). Index files were created for the reference genome as well as for the bam files using PICARD tools v2.1.1 (http://broadinstitute.github.io/picard/), and variants were called for each of the six chromosomes and populations using GATK HaplotypeCaller v3.4.46 (Van der Auwera et al. 2013) with default settings. The SNP data were extracted from the raw *.vcf files and we applied hard filtering criteria according to GATK developer’s guidelines (Van der Auwera et al. 2013). All monomorphic and triallelic SNPs were removed, thus only biallelic SNPs were further analyzed. Depth of coverage analysis was conducted using GATK DepthOfCoverage v3.4.46 (Van der Auwera et al. 2013) for each population. The distributions of coverage were close to normal, and the average values for north, center and south were 47X, 56X and 41X, respectively. For all populations, more than 94% of the sites show coverage values above 15×. To avoid individual resampling, for each population, we used a coverage interval which included 68.2% of the total amount of sites around the mean ([33–65×], [40–75×] and [31–55×], for northwestern, central and southern populations, respectively). All variants showing depth of coverage equal to one among the three populations were discarded. Information about chromosomal location, quality scores, as well as the frequencies of the reference and alternative variants for each population and chromosome was retained. The data obtained with the two alignment algorithms (Bowtie2 and BWA) were intersected, and only those SNPs identified using both tools were used for further analyses. The frequencies obtained for each polymorphic site were used to estimate total and within-population heterozygosity values as well as *F*_ST_ using classical formulas as implemented in Popoolation2 (Kofler et al. 2011) and described by Hartl and Clark (1997). These values were further averaged in 100-kb nonoverlapping sliding windows to estimate average population differentiation values along chromosomal arms for all pairwise comparisons. The values within the inverted segments were reordered accordingly to get the differentiation levels in inverted chromosomes.

### Gene Expression

To test whether the presence of inversions might affect the expression levels of at least some genes flanking the breakpoints, we performed qPCR for the orthologs of *D. melanogaster PI31*, *Fig4*, *CG9588*, and *fab1* in both *D. americana* (SF12) and *D. novamexicana* (15010-1031.00) strains. These genes flank inversions *5 b* (*PI31*), *4a* (*Fig4*), and *2c* (*CG9588*), while gene *fab1* is located <4 kb from inversion *5a* (see Results for further details about the rationale behind gene selection).

We used 1-day-old female flies, because there is no easy way to distinguish males from females during earlier life stages of these species and we wanted to avoid confounding effects caused by a mixture of males and females. The flies were reared under 12L:12D cycles at 25 °C (D. americana [SF12] and D. novamexicana [15010-1031.00]) to avoid putative effects of temperature and/or photoperiod in gene expression. Three independent sets of three female flies for each of the six strains were snap-frozen and stored at −80 °C for further mRNA extraction. Total RNA was isolated from whole bodies using TRIzol Reagent (Invitrogen, Spain) according to the manufacturer’s instructions and treated with Turbo DNA-free kit (Life technologies, Carlsbad, CA). The purity and concentration of each extracted sample were measured with NanoDrop ND-1000 spectrophotometer (NanoDrop, Thermo Scientific, Portugal), and RNA integrity was checked using Experion platform (Bio-Rad, Portugal; all the samples had RNA Quality Indicator (RQI) values above 8.5). cDNA was synthesized by reverse transcription of 1.0 μg of RNA of each sample with SuperScript III First-Strand Synthesis SuperMix for qRT-PCR (Invitrogen, Spain) using random primers. Reactions where template was not added and reactions with RNA that was not reverse transcribed were performed to confirm the absence of genomic DNA contamination. Specific primers were designed for *PI31* (5′-AACTCCGACTTCTTTTACG-3′ and 5′-ATTTGTAGTGCTTGGTCAG-3′), *Fig4* (5´CACAGCGACCTCCACATC-3′ and 5′-AGGGTGGGAGTCAAGTCA-3′), *CG9588* (5′-GCTCCATCAACGAAAATAAC-3′ and 5′-GGGACAAGTATCAAATCC-3′), and *Fab1* (5′-GAGCAAAGGCAACAATCGT-3′ and 5′-ACTGTGGAGGCGAGCATC-3′) orthologs based on the assembly of the *D. novamexicana* (15010-1031.00) and *D. americana* (SF12, H5, and W11) genomes (see Results). *RpL32* was used as the reference gene (5′-ACAACAGAGTGCGTCGTC-3′ and 5′-ATCTCCTTGCGTTTCTTC-3′) (Reis et al. 2015). Primers’ efficiencies were measured using serial dilutions of cDNA, and they were all in the 90–100% range. All qPCR reactions were carried out in 10 µl final volume solution containing 5 µl of iTaq SYBR green supermix (Bio-Rad), 3.75 µl of pure water, 0.125 µl of forward and reverse primers (10 µM), and 1 µl of cDNA. The reactions were performed on a Bio-Rad iCycler with the following program: 3 min at 95 °C; 40 cycles of 30 s at 94 °C, 30 s at 56 °C and 30 s at 72 °C followed by a standard melt curve. The threshold cycle (CT) values of technical replicates did not differ by more than 0.5 cycles in any case. Differences in expression levels were calculated using the 2^−^^ΔCT^ method (Livak and Schmittgen 2001; Schmittgen and Livak 2008).

## Results

### A Clear DAIBAM Signature Is Found at Proximal and Distal Breakpoints of Nearly All Characterized Inversions

We used an alignment approach to identify inversion breakpoints. The contigs obtained for *D. novamexicana* (15010-1031.00) and *D. americana* (SF12) genomes were reordered based on the *D. virilis* sequence for each chromosome (Clark et al. 2007) as references. Then, the contigs aligning in two different regions of the same chromosome were identified and the presence of a breakpoint was further confirmed by BlastN against the *D. virilis* genome (http://flybase.org/FB2017_05).

**Fig. 2.**
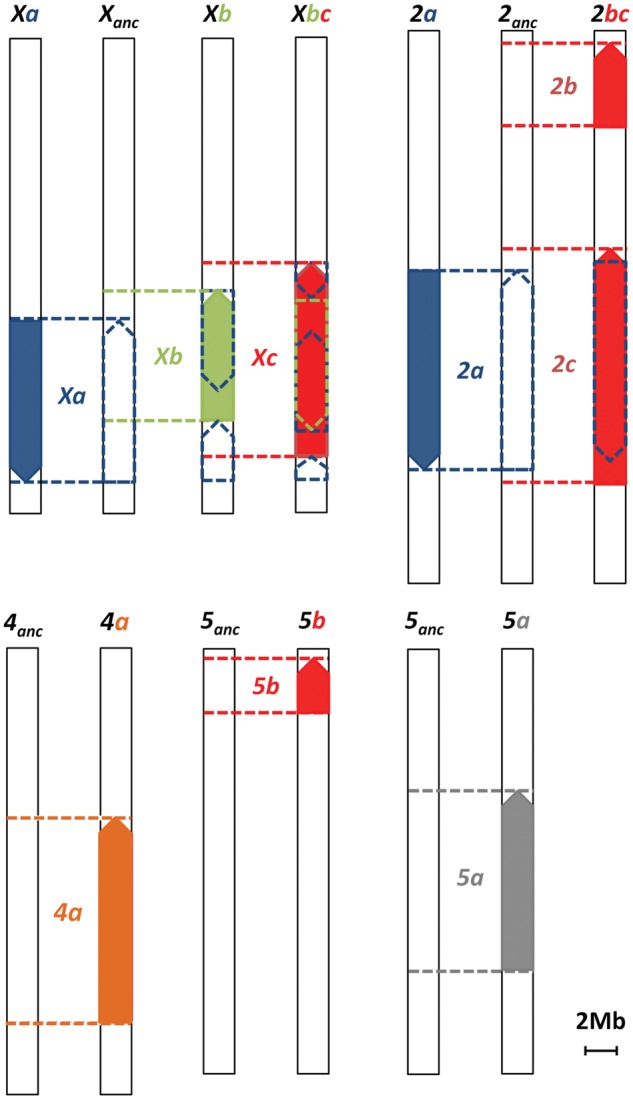
—Schematic representation of the location of the inversions present in species of the *virilis* phylad with molecularly characterized breakpoints. The location of the inversions was identified using an alignment approach. The contigs obtained for *D. americana* (SF12) and *D. novamexicana* (15010-1031.00) were reordered based on the *D. virilis* anchored scaffolds. The alignments were manually inspected, and the contigs which aligned in two different regions of the same chromosome were identified. The presence of a breakpoint was further confirmed by performing BlastN of these contigs against the *D. virilis* genome. The chromosomes are oriented from telomere (top) to centromere (bottom) based on the *D. virilis* anchored scaffolds information. The dashed arrow blocks depict the way chromosomal regions from *D. virilis* have to be rearranged to get derived inverted states either in *D. novamexicana* or in *D. americana*, and the dashed horizontal lines depict the location of chromosomal breakpoints. The order present in *D. virilis* is the ancestral one, except for the derived inversions *Xa* and *2a*. Inversions *2b* and *2c* are depicted in the same chromosome, because they co-occur in *D. novamexicana*. The colors used to depict the inversions are the same as in figure 1. anc, ancestral.

The precise chromosomal location of the newly identified breakpoints of the *Xb*, *Xc*, *2a*, *2 b*, *2c* and *5 b* inversions, as well as the previously identified breakpoints (Evans et al. 2007; Fonseca et al. 2012) (fig. 2 and supplementary figs. 1–8, Supplementary Material online) agree well with those estimated by Hsu (1952). The genes flanking the breakpoints of inversion *Xa* and the proximal breakpoint of inversion *2a* are the same in *D. novamexicana* (15010-1031.00) and *D. americana* (SF12), as well as in the genomes of more divergent *Drosophila* species, such as *D. mojavensis* and *D. grimshawi* (http://flybase.org). However, they are different in *D. virilis* indicating that inversions *Xa* and *2a* occurred in this lineage (fig. 2 and supplementary figs. 1 and 4, Supplementary Material online).

**Fig. 3.**
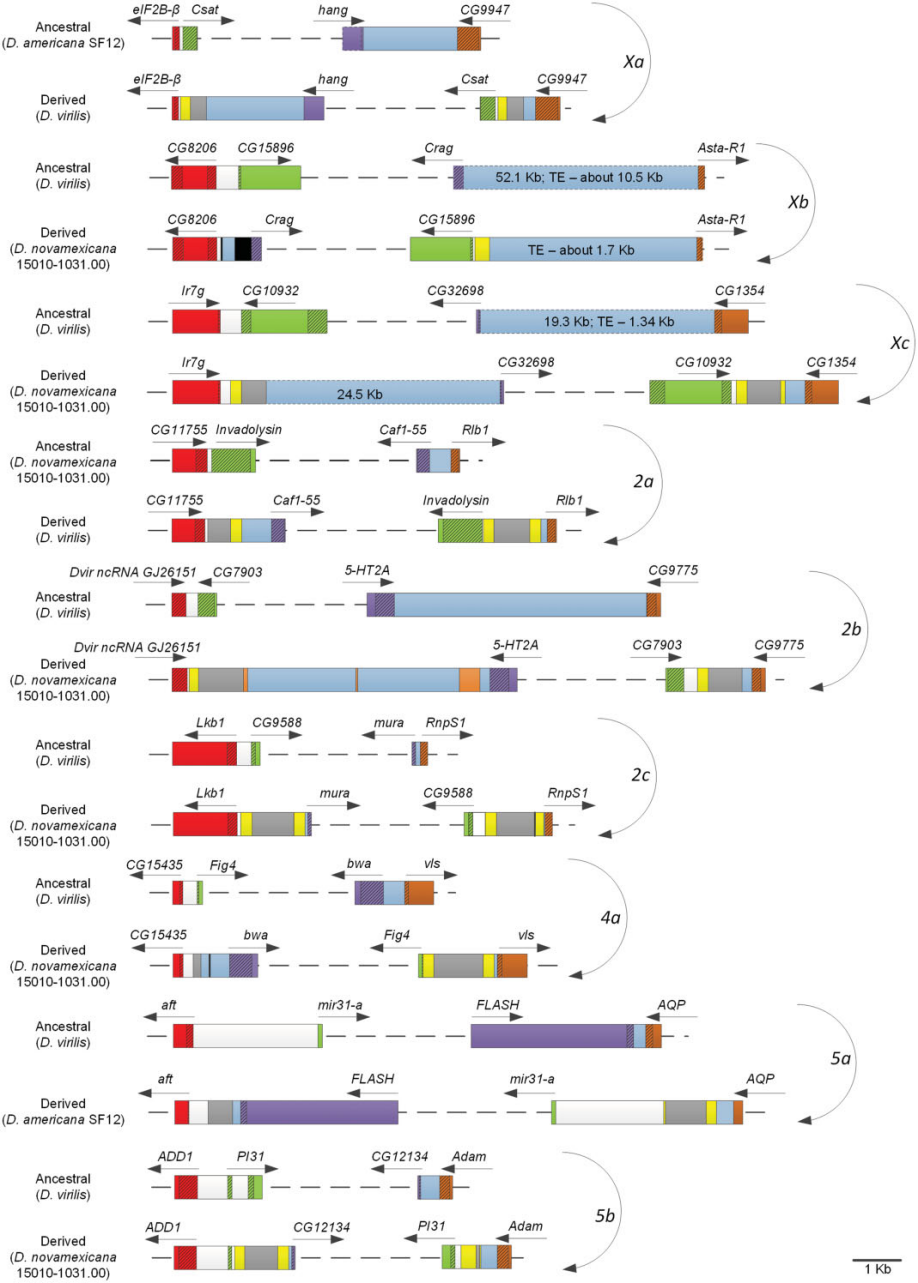
—Schematic representation of chromosomal inversion breakpoints. When broken lines are used, the representation is not to scale. Four different colors (red, light green, purple, and brown) are used to represent the four genes flanking the breakpoints of each inversion. Dashed boxes represent noncoding DNA. Intergenic regions are colored white and light blue. DAIBAM MITE elements are colored yellow (inverted terminal sequences) and gray. Black—unknown origin; orange—repetitive DNA, possibly an uncharacterized TE. Gene names are those of the *D. melanogaster* orthologous genes. See supplementary files 1–10, Supplementary Material online, for details on Contig locations and sequences used to get these diagrams.

At the molecular level we found a clear signature of the *DAIBAM* MITE element at all breakpoints, except for the two *Xb* breakpoints (fig. 3, supplementary file 3, Supplementary Material online) supporting the involvement of this element in the generation of at least eight out of nine characterized inversions in the *virilis* phylad. It should be noted that we found a sequence compatible with the terminal inverted repeats (TIR) of *DAIBAM* at one of the *Xb* inversion breakpoints but in *D. novamexicana* only. Nevertheless, Dias et al. (2014) found a nonautonomous foldback transposon, named *Tetris* that shares the final portions of its TIRs with *DAIBAM* and thus we cannot confidently say that this is a clear signature of a *DAIBAM* element, or that the inversion originated through ectopic recombination between two TE copies.

### Drosophila americana Polymorphic Inversions Create High Differentiation Levels between Populations over Large Physical Distances

To address the impact of polymorphic inversions on variability patterns between populations, we performed whole-genome sequencing of pools of individuals from the northwestern, center, and south of *D. americana* distribution in the United States. The *D. americana* (SF12) genome was used as reference after reordering the contigs based on the *D. virilis* genome (http://flybase.org FB2017_05) with the regions corresponding to the *Xa* and *2a* inversions reverted to the ancestral state (supplementary figs. 1 and 4, Supplementary Material online).

**Fig. 4.**
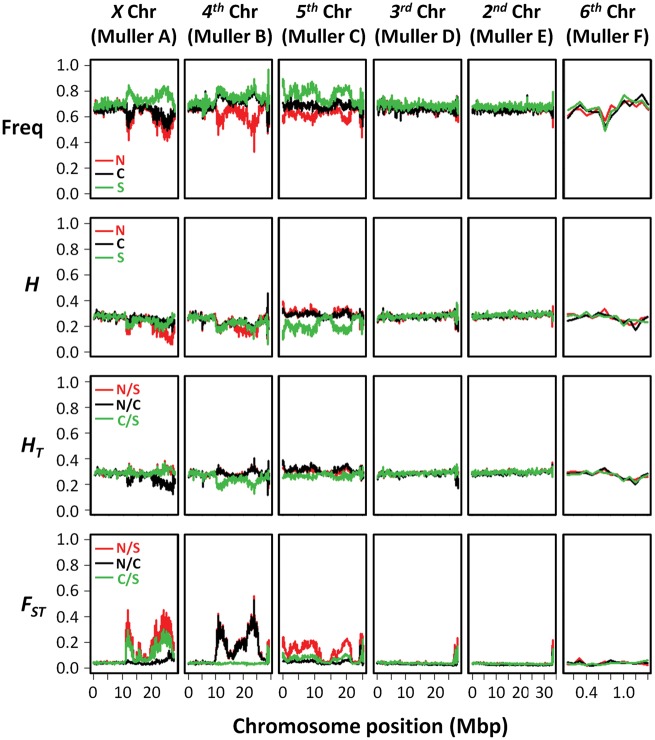
—High differentiation levels are observed only in regions of chromosomes carrying inversions. Red line—Northwest versus South; black line—Northwest versus Center; green line—Center versus South. The order shown is that of inverted chromosomes from Telomere (left) to Centromere (right). Freq-frequency of reference variant; H-within population heterozygosity; HT-total heterozygosity; FST- fixation index.

We only observed highly differentiated regions on the *X*, *4th*, and *5th* chromosomes (Muller’s elements A, B, and C, respectively) which are those carrying inversions (fig. 4). These differentiation levels were accompanied by an increase of the frequency of the reference allele in the southern population and a decrease in the northwestern one (fig. 4). Moreover, chromosome diversity, estimated by heterozygosity levels, was reduced in these regions and the effect was more pronounced in populations showing the inversions (fig. 4). The second, third, and sixth chromosomes (Muller’s elements E, D, and F, respectively) showed similar average levels of differentiation along the entire chromosomes irrespective of the pair of populations being compared (fig. 4). These differentiation levels were caused by similar frequencies and chromosomal diversity levels along the entire chromosomes among populations what should reflect the average levels observed in *D. americana* populations under free historical recombination.

**Fig. 5.**
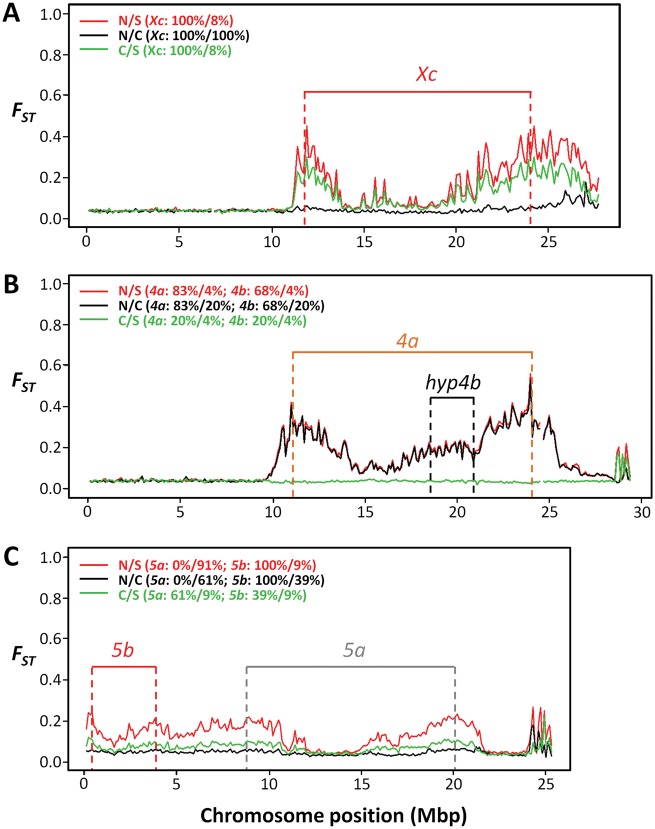
—The differentiation levels drop with increased distance to the breakpoints. Differentiation levels were measured by *F*_ST_ for every pairwise comparison between northwestern, central, and southern populations of *D. americana* for the *X* (*A*), *4th* (*B*), and *5th* (*C*) chromosomes (red—Northwest v. South; black—Northwest v. Center, and green—Center v. South). The position of the inversions present in these chromosomes was estimated based on *D. americana* (SF12) reference genome after performing BlastN of the genes flanking the breakpoints. The hypothetical position of the *4b* inversion (hyp4b), as well as the historical inversions frequencies were estimated based on data provided by Hsu (1952).

The *D. americana X* chromosome (Muller’s element A) shows two polymorphic inversions (*Xc* and *Xd*), but inversion *Xd* is limited to northwestern populations and is present at a very low frequency (6%). Inversion *Xc* is fixed in northwestern populations and is almost absent in southern populations (7.5% frequency). Its frequency shows a north–south cline that has been extensively documented (Vieira et al. 2001, 2006; McAllister 2002; Reis et al. 2008). The observed patterns of differentiation between populations suggest that the presence of inversion *Xc* strongly suppresses recombination in 64% of the chromosome. The differentiation levels dropped with increased distance to the breakpoints, and this effect was more pronounced for the distal breakpoint (fig. 5*A*). The differentiation levels also dropped toward the centromere and the most telomeric one-third of the chromosome likely undergoes free recombination, because it presented the same differentiation levels as the chromosomes not carrying inversions (fig. 4).

The *4th* chromosome (Muller’s element B) shows four polymorphic inversions (*4a*, *4 b*, *4c*, and *4d*). Inversion *4c* is present in northwestern populations only, and at a low frequency (6.1%), while *4d* must be extremely rare because it was not observed by Hsu (1952) based on a large sample size. The estimated frequencies in northwestern, central, and southern populations are 83.3%, 19.6%, and 3.8% for inversion *4a* and 68.2%, 19.6%, and 3.8% for inversion *4 b*, respectively. Inversion *4 b* mostly co-occurs with inversion *4a* (Hsu 1952). The population differentiation analysis showed that the telomeric one-third of the chromosome was the only portion that freely recombines in heterokaryotypes (figs. 4 and 5*B*). We also observed a reduction of the differentiation levels with increased distance to the breakpoints, and the effect was more pronounced in the distal breakpoint (fig. 5*B*). These levels were caused by an increase in the frequency of the reference variant in the southern population and a reduction in the northwestern one, as it was observed for the *Xc* inversion (fig. 4). The broader pattern of high differentiation might be caused by the co-occurrence of the *4 b* inversion (fig. 5*B*), which apparently creates a region of reduced heterozygosity within the *4a* inverted segment (fig. 4).

The *D. americana5th* chromosome (Muller’s element C) shows two polymorphic inversions (*5a* and *5b*). Inversion *5 b* is fixed in northwestern populations, while inversion *5a* is almost fixed in the southern populations (91% frequency) (Hsu 1952). Moreover, in the center of the distribution, inversions *5a* and *5 b* have an estimated frequency of 61% and 39%, respectively. Therefore, the frequency difference of inversion *5a* and *5 b* was higher between northwestern and southern populations than between the central and southern populations, which were reflected in the differentiation patterns observed (fig. 5*C*). Together, inversions *5a* and *5 b* likely suppress recombination in at least 80% of the chromosome, but the differentiation levels were remarkably lower than those observed for the *X* and *4th* chromosomes. Moreover, the differentiation levels were lower in the middle of the *5a* inversion (fig. 5*C*). The diversity levels observed for the *5th* chromosome in the southern population were lower than those observed for northwestern and central populations (fig. 4), and the reduction of the frequency of the reference allele in the northwestern population did not lead to a reduction in the diversity levels (fig. 4). This result coupled with the lower overall reduction in differentiation levels suggest that the *5 b* inversion is not totally fixed in the northwestern population.

### The Effect of Inversions on Genes Flanking the Breakpoints

The detailed analysis of the distance from DAIBAM insertions to transcription start sites (TSS), and depending on the transcription orientation of the gene being analyzed, to the start or stop codons, can give insight into whether the expression of genes flanking the inversion breakpoints could be affected by the presence of a given inversion.

**Fig. 6.**
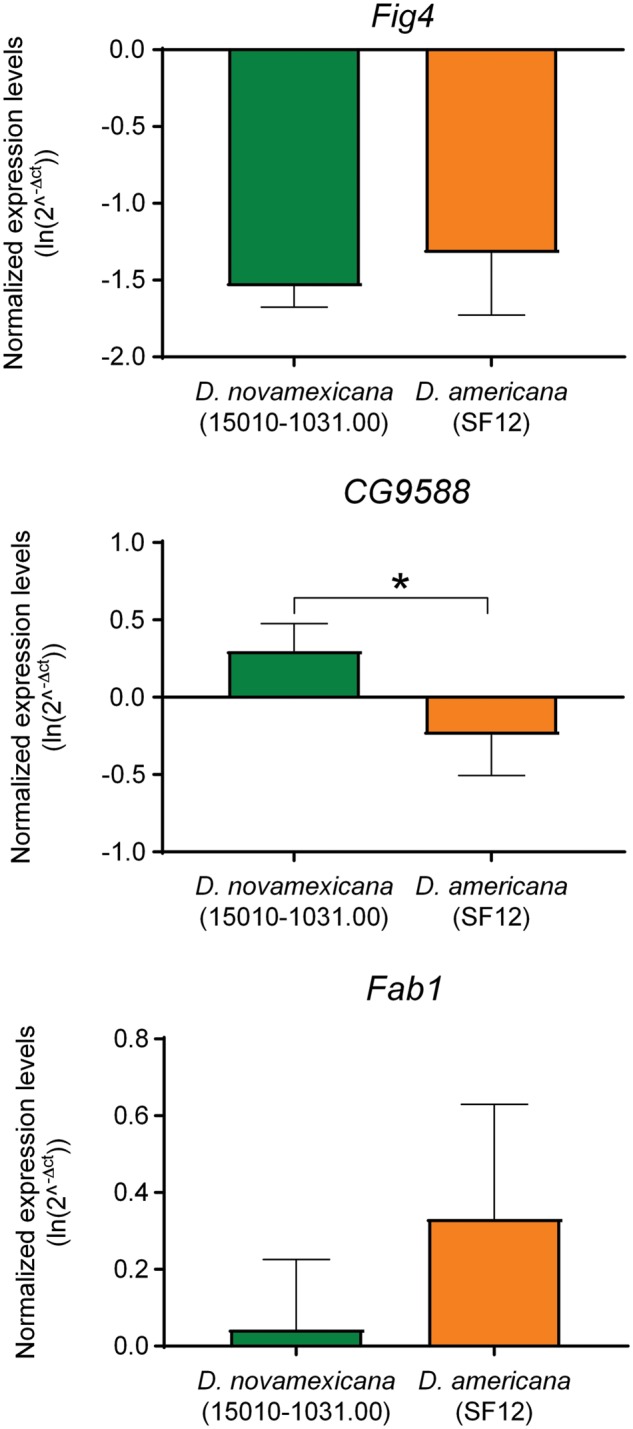
—Expression levels of three genes (*Fig4*, *CG9588*, and *Fab1*) flanking inversion breakpoints. Normalized expression levels were obtained for *D. novamexicana* 15010-1031.00 and *D. americana* SF12. *P < 0.05.

We found many cases where the DAIBAM insertion is located <100 bp away from the TSS meaning that the insertion could disrupt the enhancers and other regulatory elements of genes such as *elF2B-B*, *Csat*, *CG8206*, *CG15896*, *Asta-R1*, *CG11755*, *Invadolysin*, *ncRNA GJ26151*, *Lkb1*, *mura*, *RnpS1*, *Fig4*, *vls*, and *CG12134* (table 1). The TSS of one out of the four putative transcripts of *PI31* is disrupted by the proximal breakpoint of inversion *5 b*, and in the case of *Fig4* the DAIBAM insertion located at the proximal breakpoint of inversion *4a* disrupts the TSS and is located 20 bp from the start codon (table 1). Therefore, these two genes represent good candidates to test the “position effect” hypothesis (Sperlich and Pfreim 1986). According to the interactome data available in FlyBase (http://flybase.org), CG9588 interacts with PI31 (*5b*) and its gene locus flanks the proximal breakpoint of inversion *2c* (table 1). Moreover, there is also evidence for an interaction between Fig4 (*4a*) and fab1 (http://flybase.org) of which the gene locus is located <4 kb away from the proximal breakpoint of inversion *5a*. Thus, we also included *CG9588* and *fab1* to evaluate their expression levels between *D. americana* (SF12) and *D. novamexicana* (15010-1031.00). We were not able to evaluate the expression of *PI31*, because the expression levels were too low to be quantified in a proper manner. Moreover, we could not find statistically significant differences in expression levels between *D. americana* (SF12) and *D. novamexicana* (15010-1031.00) strains for the three genes surveyed (*CG9588*, *Fig4*, and *fab1*) (fig. 6). Although the result for *CG9588* is significant on its own at the 0.05 level, it is not listwise (after Bonferroni correction).

**Table 1:** Distance between DAIBAM Elements and Selected Gene Features

Species/Strain	Inv	BP	GP	Gene	TSS	ATG	TES	STOP
*Drosophila virilis*	*Xa*	d	d	*eIF2B-β/GJ18516* [**−**]	41	142		
		d	p	*hang/GJ18515* [**−**]	≈2000	≈2000		
		p	d	*Csat* /*GJ19143* [**−**]	51	379		
		p	p	*CG9947/GJ19142* [−]			256	749
*Drosophila americana* (H5, W11, SF12) *Drosophila novamexicana* (15010-1031.00)	*Xb*	d	d	*CG8206/GJ14731* [−]	62	108		
		d	p	*Crag/GJ18832* [+]	>44000	>44000		
		p	d	*CG15896*/*GJ14858* [−]	82	266		
		p	p	*Asta-R1*/*GJ19325* [+]	28	>47000		
*Drosophila novamexicana* (15010-1031.00) *Drosophila americana* (H5, W11)	*Xc*	d	d	*Ir7g/GJ17050* [+]			233	273
		d	p	*CG32698/GJ18715* [+]	≈24500	≈24500		
		p	d	*CG10932/GJ16378* [+]			118	305
		p	p	*CG1354/GJ18714* [−]			437	586
*Drosophila virilis*	*2a*	d	d	*CG11755/GJ23198* [+]			62	270
		d	p	*Caf1-55/GJ23199* [+]	635	910		
		p	d	*Invadolysin/GJ10317* [−]	21	849		
		p	p	*Rlb1/GJ10856* [+]	124	291		
*Drosophila novamexicana* (15010-1031.00)	*2b*	d	d	*Dvir ncRNA GJ26151* [+]			50	
		d	p	*5-HT2A/GJ22656* [−]			263	663
		p	d	*CG7903/GJ14327* [+]			283	634
		p	p	*CG9775/GJ24596* [−]			222	411
*Drosophila novamexicana* (15010-1031.00)	*2c*	d	d	*Lkb1/GJ24114* [−]	67	268		
		d	p	*mura/GJ10273* [+]	74	4631		
		p	d	*CG9588/GJ23109* [−]	258	343		
		p	p	*RnpS1/GJ10904* [+]	29	185		
*Drosophila novamexicana* (15010-1031.00)	*4a*	d	d	*CG15435/GJ17390* [−]	226	295		
		d	p	*bwa*/*GJ18341* [+]	>600	>1076		
		p	d	*FIG4/GJ23322* [−]	−19^a^	20		
		p	p	*vls/GJ17892* [+]	60	159		
*Drosophila americana* (H5, W11, SF12)	*5a*	d	d	*aft/GJ22245* [−]	293	432		
		d	p	*FLASH/GJ20866* [−]			185	312
		p	d	*mir-31a* [−]	2305			
		p	p	*AQP/GJ21471* [−]			118	290
*Drosophila novamexicana* (15010-1031.00)	*5b*	d	d	*ADD1/GJ17920* [−]	965	1202		
		d	p	*CG12134/GJ21267* [+]	76	122		
		p	d	*PI31/GJ18358* [−]	−140^a^	246		
		p	p	*Adam/GJ21266* [−]			350	594

Note.—Inv, inversion; BP, inversion breakpoint; GP, gene position; TSS, transcription start site; ATG, start codon; TES, transcription end site; STOP, stop codon d, distal; p, proximal. [+], plus strand; [−], minus strand. Both the name of the orthologous gene in *D. melanogaster* and *D. virilis* assembly are given.

a The negative distance indicates that DAIBAM is inserted inside the predicted transcript.

## Discussion

The *D. americana* (SF12) and *D. novamexicana* (15010-1031.00) draft genomes generated in this study allowed the molecular characterization of the breakpoints of six new inversions (*Xb*, *Xc*, *2a*, *2 b*, *2c*, and *5b*) as well as those of inversions *4a* (Evans et al. 2007), *Xa* and *5a* (Fonseca et al. 2012) already characterized. Therefore, only those of inversions *Xd*, *3a*, *4 b*, *4c*, and *4d* remain to be characterized in the *virilis* phylad. Inversions *Xd*, *4c*, and *4d* are not present in either *D. americana* (SF12, from the south of the distribution) or in *D. novamexicana* (15010-1031.00 strain), and inversion *4 b* is extremely rare in southern populations and it is absent in *D. novamexicana* (Hsu 1952). It is thus not surprising that inversion *4 b* is not present in *D. americana* (SF12) genome. Nevertheless, inversion *3a* is present in all *D. novamexicana* strains examined (Hsu 1952), but we failed to characterize its breakpoints most likely because there was no *D. novamexicana* (15010-1030.00) contig spanning the breakpoints. A similar result was observed for inversion *4a* (supplementary file 8, Supplementary Material online).

The MITE element DAIBAM is clearly present in eight breakpoints of the nine characterized inversions, suggesting that this element was fundamental in the generation of most inversions observed in the *virilis* phylad. These results resemble those of Delprat et al. (2009) that showed unequivocally that ectopic recombination between *Galileo* TEs generated three polymorphic *D. buzzatii* inversions. *DAIBAM* can only be found in species of the *virilis* group (*D. virilis*, *D. novamexicana*, and *D. americana*), and thus could be a relatively recent invader (Fonseca et al. 2012). *Penelope* elements have repeatedly invaded and reinvaded species of the *D. virilis* group (Morales-Hojas et al. 2006), and *Polyphemus* may have repeatedly invaded *D. virilis* as well (Blumenstiel 2014). Moreover, *Penelope* is able to promote the comobilization of other unrelated TEs (Vieira et al. 1998), and thus it is conceivable that the repeated invasion of the *virilis* group by *Penelope* led to an increase of TE activity and, as a consequence, of chromosomal inversions. Dias et al. (2014) found a nonautonomous foldback transposon, named *Tetris*, present in *D. virilis* and *D. americana*, that shares the final portions of its TIRs with *DAIBAM*. It is thus likely, that both elements are mobilized by the same unknown autonomous TE. *Tetris* may have contributed to the shaping of the genomes of *D. virilis* and *D. americana*, by providing internal tandem repeats that served as building blocks for the amplification of satellite DNA arrays.

The results presented here show that ectopic recombination between TEs (Kupiec and Petes 1988; Lim and Simmons 1994; Delprat et al. 2009) is the prevalent mechanism involved in the generation of inversions in the *virilis* phylad. This is in contrast with what happens in *D. melanogaster* and its close relatives where nonhomologous end-joining repair (Sonoda et al. 2006) is the prevalent mechanism (Ranz et al. 2007).

Our pool-seq data showed that inverted chromosomes show higher differentiation levels along the inverted segment when compared with regions not physically affected by the inversions. This is the likely reason why when analyzing genes located around inversion breakpoints, sometimes individuals cluster by the presence of the inversion rather than by known species relationships (Morales-Hojas et al. 2011; Fuller et al. 2018). One possible explanation for the observed differentiation patterns is the eventual disruption of genome regions that are more prone to recombination by crossing-over (“sensitive sites”; Corbett-Detig 2016) by inversion breakpoints. It has been suggested that inversions containing breakpoints affecting these regions in *D. melanogaster* have a high chance of reaching high frequencies and eventually go to fixation (Corbett-Detig 2016, and references therein). We showed here that the differentiation levels were remarkably higher around the breakpoints and dropped with increased distance to the breakpoints. This effect was most pronounced for inversions *Xc* and *4a*. It has been proposed that the regions within large inversions can pair in heterokaryotypes, and even if the frequency of crossing-over events is reduced, there is still gene conversion (reviewed in Andolfatto et al. 2001). Thus, the differentiation patterns within the inverted segments between *D. americana* populations may be explained by gene conversion over the last million years. Our observations in *D. americana* regarding the impact of inversions on the recombination landscape are thus compatible with the hypothesis that inversions locally capture adapted sets of genes, protecting them from recombination with immigrant chromosomes (Kirkpatrick and Barton 2006; Kirkpatrick 2010). It should be noted, however, that the differentiation levels within inverted segments are still higher than those expected under free historical recombination and we observed intermittent peaks of high differentiation (fig. 5). This pattern of highly differentiated regions in the presence of gene conversion was also observed by Fuller et al. (2017), and it is suggestive of adaptive selection.

Guillen and Ruiz (2012) have argued that given the high gene density and compact structure of *Drosophila* genomes, it is likely that genes around inversion breakpoints could be the targets of selection responsible for the increase in frequency and maintenance of inversions in natural populations. Nevertheless, differences in genome-wide expression levels evaluated by RNA-seq between different chromosomal arrangements in *D. pseudoobscura* identified only one differentially expressed gene associated with an inversion breakpoint (Fuller et al. 2016). However, there are cases of gene disruption in *Drosophila*, mice, and plants (see references in Fuller et al. [2016]). Additionally, Puig et al. (2004) observed dramatic changes in gene expression in a gene located close to an inversion breakpoint in *D. buzzatii*. They have found that a *Galileo* insertion 12 bp away from the stop codon leads to a reduction in gene expression. Here, we show that the presence of inversions does not significantly affect expression of the breakpoints flanking genes *Fig4*, *fab1*, and *CG9588*. However, we evaluated expression differences in 1-day-old adult females. It is conceivable that inversions may influence modular enhancers and, thus may only affect gene expression in a specific tissue or at a specific time point. Therefore, we cannot rule out the possibility of positional effects of inversions on gene expression (“position effect” hypothesis [Sperlich and Pfreim 1986]). As such, we cannot also rule out that the interactions between genes flanking different inversions (*Fig4* [*4a*] and *fab1* [*5a*] [Botelho et al. 2008], as well as *PI31* [*5b*] and *CG9588* [*2c*]); see the interactome data available in FlyBase (http://flybase.org) are maintaining these inversions at different frequencies in natural populations by keeping together alleles at loci with epistatic effects on fitness (“coadaptation” hypothesis [Dobzhansky 1970]).

Further analysis of genes flanking the inversion breakpoints can give insight into the nature of the selective forces that maintain the north–south inversion clines in *D. americana*. In a genome-wide survey, 580 out of 11,664 genes have been identified as being involved in heat nociception (or heat perception) (Neely et al. 2011). Of the 36 genes that surround the breakpoints shown here (table 1), 23 genes have been included in this genome-wide survey, and five of them are heat nociception candidates, including gene *CG9588.* This observation could indicate selection for high temperature avoidance associated with the inversions. This is in agreement with the observation made by Sillero et al. (2014) that avoidance plays an important role in thermoregulation in *D. americana*. Moreover, it has been shown that *D. virilis*, *D. lummei*, *D. novamexicana*, and southern populations of *D. americana* have different basal and inducible heat tolerances (Garbuz et al. 2003) at least partially due to variation in *Hsp70* gene family copy number (Garbuz et al. 2002). Temperature is also the environmental factor that contributes most to explain the ecological niche distribution of *D. americana* (Sillero et al. 2014). It is, thus, conceivable that at least a fraction of the chromosomal inversions could be associated with gene variants that promote different susceptibilities to temperature.

In *D. americana*, there is also a large amount of variation regarding traits such as diapause incidence (Reis et al. 2015), cold resistance (Reis et al. 2011), abdominal size (Reis et al. 2011), developmental time (Fonseca et al. 2013), body color (Wittkopp et al. 2011), and lifespan (Fonseca et al. 2013). Therefore, the very different frequency of common *D. americana* polymorphic inversions on chromosomes *X*, *4*, and *5* and associated differentiation over large physical distances also mean that very likely the molecular basis of many north–south phenotypic differences is located on these chromosomes.

In conclusion, species of the *virilis* phylad represent an excellent model to contrast the findings obtained for *D. melanogaster* and close relatives regarding the origin, rise to high frequency, and consequences of inversions. The information provided in this work will be extremely useful for many studies addressing the role of chromosomal inversions as a source of genomic variability explaining phenotypic differences and local adaptation.

## Supplementary Material

Supplementary Data

## References

[evy239-B1] Andolfatto P, Depaulis F, Navarro A. 2001 Inversion polymorphisms and nucleotide variability in *Drosophila*. Genet Res. 77(1):1–8. 11279826 10.1017/s0016672301004955

[evy239-B2] Bartolome C, Charlesworth B. 2006 Rates and patterns of chromosomal evolution in *Drosophila pseudoobscura* and *D. miranda*. Genetics 173(2):779–791. 16547107 10.1534/genetics.105.054585 PMC1526542

[evy239-B3] Bhutkar A, et al 2008 Chromosomal rearrangement inferred from comparisons of 12 *Drosophila* genomes. Genetics 179(3):1657–1680. 18622036 10.1534/genetics.107.086108 PMC2475759

[evy239-B4] Blankenberg D, et al 2010 Manipulation of FASTQ data with Galaxy. Bioinformatics 26(14):1783–1785. 20562416 10.1093/bioinformatics/btq281 PMC2894519

[evy239-B5] Blumenstiel JP. 2014 Whole genome sequencing in *Drosophila virilis* identifies *Polyphemus*, a recently activated Tc1-like transposon with a possible role in hybrid dysgenesis. Mob DNA. 5(1):6. 24555450 10.1186/1759-8753-5-6 PMC3941972

[evy239-B6] Boetzer M, Henkel CV, Jansen HJ, Butler D, Pirovano W. 2011 Scaffolding pre-assembled contigs using SSPACE. Bioinformatics 27(4):578–579. 21149342 10.1093/bioinformatics/btq683

[evy239-B7] Botelho RJ, Efe JA, Teis D, Emr SD. 2008 Assembly of a Fab1 phosphoinositide kinase signaling complex requires the Fig4 phosphoinositide phosphatase. Mol Biol Cell. 19(10):4273–4286. 18653468 10.1091/mbc.E08-04-0405 PMC2555960

[evy239-B8] Caceres M, Ranz JM, Barbadilla A, Long M, Ruiz A. 1999 Generation of a widespread *Drosophila* inversion by a transposable element. Science 285(5426):415–418. 10411506 10.1126/science.285.5426.415

[evy239-B9] Caceres M, Sullivan RT, Thomas JW. 2007 A recurrent inversion on the eutherian *X* chromosome. Proc Natl Acad Sci U S A. 104(47):18571–18576. 18003915 10.1073/pnas.0706604104 PMC2141818

[evy239-B10] Caletka BC, McAllister BF. 2004 A genealogical view of chromosomal evolution and species delimitation in the *Drosophila virilis* species subgroup. Mol Phylogenet Evol. 33(3):664–670. 15522794 10.1016/j.ympev.2004.08.007

[evy239-B11] Camacho C, et al 2009 BLAST plus: architecture and applications. BMC Bioinformatics 10:421. 20003500 10.1186/1471-2105-10-421 PMC2803857

[evy239-B12] Casals F, Caceres M, Ruiz A. 2003 The foldback-like transposon *Galileo* is involved in the generation of two different natural chromosomal inversions of *Drosophila buzzatii*. Mol Biol Evol. 20(5):674–685. 12679549 10.1093/molbev/msg070

[evy239-B13] Clark AG, et al 2007 Evolution of genes and genomes on the *Drosophila* phylogeny. Nature 450(7167):203–218. 17994087 10.1038/nature06341

[evy239-B14] Corbett-Detig RB. 2016 Selection on inversion breakpoints favors proximity to pairing sensitive sites in *Drosophila melanogaster*. Genetics 204(1):259–265. 27343234 10.1534/genetics.116.190389 PMC5012391

[evy239-B15] Coulibaly MB, et al 2007 Segmental duplication implicated in the genesis of inversion *2Rj* of *Anopheles gambiae*. PLoS One 2(9):e849. 17786220 10.1371/journal.pone.0000849 PMC1952172

[evy239-B16] Delprat A, Negre B, Puig M, Ruiz A. 2009 The transposon *Galileo* generates natural chromosomal inversions in *Drosophila* by ectopic recombination. PLoS One 4(11):e7883. 19936241 10.1371/journal.pone.0007883 PMC2775673

[evy239-B17] Dias GB, Svartman M, Delprat A, Ruiz A, Kuhn GCS. 2014 Tetris is a foldback transposon that provided the building blocks for an emerging satellite DNA of *Drosophila virilis*. Genome Biol Evol. 6(6):1302–1313. 24858539 10.1093/gbe/evu108 PMC4079207

[evy239-B18] Dobzhansky T. 1970 Genetics of the evolutionary process. New York: Columbia University Press.

[evy239-B19] Eddy SR. 2011 Accelerated profile HMM searches. PLoS Comput Biol. 7(10):e1002195. 22039361 10.1371/journal.pcbi.1002195 PMC3197634

[evy239-B20] Evans AL, Mena PA, McAllister BF. 2007 Positive selection near an inversion breakpoint on the Neo-*X* chromosome of *Drosophila americana*. Genetics 177(3):1303–1319. 17660565 10.1534/genetics.107.073932 PMC2147947

[evy239-B21] Feder JL, Gejji R, Powell TH, Nosil P. 2011 Adaptive chromosomal divergence driven by mixed geographic mode of evolution. Evolution 65(8):2157–2170. 21790566 10.1111/j.1558-5646.2011.01321.x

[evy239-B22] Fonseca NA, et al 2013 *Drosophila americana* as a model species for comparative studies on the molecular basis of phenotypic variation. Genome Biol Evol. 5(4):661–679. 23493635 10.1093/gbe/evt037 PMC3641629

[evy239-B23] Fonseca NA, Vieira CP, Schlötterer C, Vieira J. 2012 The DAIBAM MITE element is involved in the origin of one fixed and two polymorphic *Drosophila virilis* phylad inversions. Fly 6(2):71–74. 22561870 10.4161/fly.19423

[evy239-B24] Fuller ZL, Haynes GD, Richards S, Schaeffer SW. 2016 Genomics of natural populations: how differentially expressed genes shape the evolution of chromosomal inversions in *Drosophila pseudoobscura*. Genetics 204(1):287–301. 27401754 10.1534/genetics.116.191429 PMC5012393

[evy239-B25] Fuller ZL, Haynes GD, Richards S, Schaeffer SW. 2017 Genomics of natural populations: evolutionary forces that establish and maintain gene arrangements in *Drosophila pseudoobscura*. Mol Ecol. 26(23):6539–6562. 29055159 10.1111/mec.14381

[evy239-B26] Fuller ZL, Leonard CJ, Young RE, Schaeffer SW, Phadnis N. 2018 Ancestral polymorphisms explain the role of chromosomal inversions in speciation. PLoS Genet. 14(7):e1007526. 30059505 10.1371/journal.pgen.1007526 PMC6085072

[evy239-B27] Garbuz D, Evgenev MB, Feder ME, Zatsepina OG. 2003 Evolution of thermotolerance and the heat-shock response: evidence from inter/intraspecific comparison and interspecific hybridization in the *virilis* species group of *Drosophila*. I. Thermal phenotype. J Exp Biol. 206(Pt 14):2399–2408. 12796457 10.1242/jeb.00429

[evy239-B28] Garbuz DG, Molodtsov VB, Velikodvorskaia VV, Evgen'ev MB, Zatsepina OG. 2002 Evolution of the response to heat shock in genus *Drosophila*. Russ J Genet+ 38(8):925–936. 12244694

[evy239-B29] Giardine B, et al 2005 Galaxy: a platform for interactive large-scale genome analysis. Genome Res. 15(10):1451–1455. 16169926 10.1101/gr.4086505 PMC1240089

[evy239-B30] Gonzalez J, Casals F, Ruiz A. 2007 Testing chromosomal phylogenies and inversion breakpoint reuse in *Drosophila*. Genetics 175(1):167–177. 17028333 10.1534/genetics.106.062612 PMC1775012

[evy239-B31] Guillen Y, Ruiz A. 2012 Gene alterations at *Drosophila* inversion breakpoints provide prima facie evidence for natural selection as an explanation for rapid chromosomal evolution. BMC Genomics 13:53. 22296923 10.1186/1471-2164-13-53 PMC3355041

[evy239-B32] Hartl DL, Clark AG. 1997 Principles of population genetics. Sunderland (MA): Sinauer Associates.

[evy239-B33] Hsu TC. 1952 Chromosomal variation and evolution in the virilis group of Drosophila. Vol. 5204 Austin (TX): University of Texas Publications p. 35–72.

[evy239-B34] Keller O, Kollmar M, Stanke M, Waack S. 2011 A novel hybrid gene prediction method employing protein multiple sequence alignments. Bioinformatics 27(6):757–763. 21216780 10.1093/bioinformatics/btr010

[evy239-B35] Kirkpatrick M. 2010 How and why chromosome inversions evolve. PLoS Biol. 8(9):e1000501. 20927412 10.1371/journal.pbio.1000501 PMC2946949

[evy239-B36] Kirkpatrick M, Barton N. 2006 Chromosome inversions, local adaptation and speciation. Genetics 173(1):419–434. 16204214 10.1534/genetics.105.047985 PMC1461441

[evy239-B37] Kofler R, Langmüller AM, Nouhaud P, Otte KA, Schlötterer C. 2016 Suitability of different mapping algorithms for genome-wide polymorphism scans with Pool-seq data. G3 6:3507–3515. 27613752 10.1534/g3.116.034488 PMC5100849

[evy239-B38] Kofler R, Pandey RV, Schlotterer C. 2011 PoPoolation2: identifying differentiation between populations using sequencing of pooled DNA samples (Pool-Seq). Bioinformatics 27(24):3435–3436. 22025480 10.1093/bioinformatics/btr589 PMC3232374

[evy239-B39] Kupiec M, Petes TD. 1988 Allelic and ectopic recombination between Ty elements in yeast. Genetics 119(3):549–559. 2841187 10.1093/genetics/119.3.549 PMC1203441

[evy239-B40] Langmead B, Salzberg SL. 2012 Fast gapped-read alignment with Bowtie 2. Nat Methods. 9(4):357–359. 22388286 10.1038/nmeth.1923 PMC3322381

[evy239-B41] Li H, Durbin R. 2009 Fast and accurate short read alignment with Burrows–Wheeler transform. Bioinformatics 25(14):1754–1760. 19451168 10.1093/bioinformatics/btp324 PMC2705234

[evy239-B42] Li H, et al 2009 The Sequence Alignment/Map format and SAMtools. Bioinformatics 25(16):2078–2079. 19505943 10.1093/bioinformatics/btp352 PMC2723002

[evy239-B43] Lim JK, Simmons MJ. 1994 Gross chromosome rearrangements mediated by transposable elements in *Drosophila melanogaster*. BioEssays 16(4):269–275. 8031304 10.1002/bies.950160410

[evy239-B44] Livak KJ, Schmittgen TD. 2001 Analysis of relative gene expression data using real-time quantitative PCR and the 2^−ΔΔCT^ method. Methods 25(4):402–408. 11846609 10.1006/meth.2001.1262

[evy239-B45] Luo RB, et al 2012 SOAPdenovo2: an empirically improved memory-efficient short-read de novo assembler. Gigascience 1(1):18. 23587118 10.1186/2047-217X-1-18 PMC3626529

[evy239-B46] Magoc T, Salzberg SL. 2011 FLASH: fast length adjustment of short reads to improve genome assemblies. Bioinformatics 27(21):2957–2963. 21903629 10.1093/bioinformatics/btr507 PMC3198573

[evy239-B47] McAllister BF. 2002 Chromosomal and allelic variation in *Drosophila americana*: selective maintenance of a chromosomal cline. Genome 45(1):13–21. 11908655 10.1139/g01-112

[evy239-B48] McGaugh SE, Noor MA. 2012 Genomic impacts of chromosomal inversions in parapatric *Drosophila* species. Philos Trans R Soc Lond B Biol Sci. 367(1587):422–429. 22201171 10.1098/rstb.2011.0250 PMC3233717

[evy239-B49] Morales-Hojas R, Reis M, Vieira CP, Vieira J. 2011 Resolving the phylogenetic relationships and evolutionary history of the *Drosophila virilis* group using multilocus data. Mol Phylogenet Evol. 60(2):249–258. 21571080 10.1016/j.ympev.2011.04.022

[evy239-B50] Morales-Hojas R, Vieira CP, Vieira J. 2006 The evolutionary history of the transposable element *Penelope* in the *Drosophila virilis* group of species. J Mol Evol. 63(2):262–273. 16830099 10.1007/s00239-005-0213-1

[evy239-B51] Morales-Hojas R, Vieira CP, Vieira J. 2008 Inferring the evolutionary history of *Drosophila americana* and *Drosophila novamexicana* using a multilocus approach and the influence of chromosomal rearrangements in single gene analyses. Mol Ecol. 17(12):2910–2926. 18482259 10.1111/j.1365-294X.2008.03796.x

[evy239-B52] Morales-Hojas R, Vieira J. 2012 Phylogenetic patterns of geographical and ecological diversification in the subgenus *Drosophila*. PLoS One 7(11):e49552. 23152919 10.1371/journal.pone.0049552 PMC3495880

[evy239-B53] Navarro A, Betran E, Barbadilla A, Ruiz A. 1997 Recombination and gene flux caused by gene conversion and crossing over in inversion heterokaryotypes. Genetics 146(2):695–709. 9178017 10.1093/genetics/146.2.695 PMC1208008

[evy239-B54] Neely GG, et al 2011 TrpA1 regulates thermal nociception in *Drosophila*. PLoS One 6(8):e24343. 21909389 10.1371/journal.pone.0024343 PMC3164203

[evy239-B55] Noor MAF, Grams KL, Bertucci LA, Reiland J. 2001 Chromosomal inversions and the reproductive isolation of species. Proc Natl Acad Sci U S A. 98(21):12084–12088. 11593019 10.1073/pnas.221274498 PMC59771

[evy239-B56] Okonechnikov K, Golosova O, Fursov M, Team U. 2012 Unipro UGENE: a unified bioinformatics toolkit. Bioinformatics 28(8):1166–1167. 22368248 10.1093/bioinformatics/bts091

[evy239-B57] Papaceit M, Aguade M, Segarra C. 2006 Chromosomal evolution of elements B and C in the *Sophophora* subgenus of *Drosophila*: evolutionary rate and polymorphism. Evolution 60(4):768–781. 16739458

[evy239-B58] Powell JR. 1997 Progress and prospects in evolutionary biology: the Drosophila model. New York: Oxford University Press.

[evy239-B59] Puig M, Caceres M, Ruiz A. 2004 Silencing of a gene adjacent to the breakpoint of a widespread *Drosophila* inversion by a transposon-induced antisense RNA. Proc Natl Acad Sci U S A. 101(24):9013–9018. 15184654 10.1073/pnas.0403090101 PMC428464

[evy239-B60] Ranz JM, et al 2007 Principles of genome evolution in the *Drosophila melanogaster* species group. PLoS Biol. 5(6):e152. 17550304 10.1371/journal.pbio.0050152 PMC1885836

[evy239-B61] Reis M, et al 2011 A comparative study of the short term cold resistance response in distantly related *Drosophila* species: the role of *regucalcin* and *frost*. PLoS One 6(10):e25520. 21991316 10.1371/journal.pone.0025520 PMC3184994

[evy239-B62] Reis M, Valer FB, Vieira CP, Vieira J. 2015 *Drosophila americana* diapausing females show features typical of young flies. PLoS One 10(9):e0138758. 26398836 10.1371/journal.pone.0138758 PMC4580583

[evy239-B63] Reis M, Vieira CP, Morales-Hojas R, Vieira J. 2008 An old *bilbo*-like non-LTR retroelement insertion provides insight into the relationship of species of the *virilis* group. Gene 425(1–2):48–55. 18775768 10.1016/j.gene.2008.08.010

[evy239-B64] RichardsS, et al 2005 Comparative genome sequencing of *Drosophila pseudoobscura*: chromosomal, gene, and cis-element evolution. Genome Res. 15(1):1–18.1563208510.1101/gr.3059305PMC540289

[evy239-B65] Rissman AI, et al 2009 Reordering contigs of draft genomes using the Mauve aligner. Bioinformatics 25(16):2071–2073. 19515959 10.1093/bioinformatics/btp356 PMC2723005

[evy239-B66] Rius N, Delprat A, Ruiz A. 2013 A divergent P element and its associated MITE, BuT5, generate chromosomal inversions and are widespread within the *Drosophila repleta* species group. Genome Biol Evol. 5(6):1127–1141. 23682154 10.1093/gbe/evt076 PMC3698922

[evy239-B67] Schaeffer SW, et al 2008 Polytene chromosomal maps of 11 *Drosophila* species: the order of genomic scaffolds inferred from genetic and physical maps. Genetics 179(3):1601–1655. 18622037 10.1534/genetics.107.086074 PMC2475758

[evy239-B68] Schmittgen TD, Livak KJ. 2008 Analyzing real-time PCR data by the comparative C(T) method. Nat Protoc. 3(6):1101–1108. 18546601 10.1038/nprot.2008.73

[evy239-B69] Sillero N, Reis M, Vieira CP, Vieira J, Morales-Hojas R. 2014 Niche evolution and thermal adaptation in the temperate species *Drosophila americana*. J Evol Biol. 27(8):1549–1561. 24835376 10.1111/jeb.12400

[evy239-B70] Simão FA, Waterhouse RM, Ioannidis P, Kriventseva EV, Zdobnov EM. 2015 BUSCO: assessing genome assembly and annotation completeness with single-copy orthologs. Bioinformatics 31(19):3210–3212. 26059717 10.1093/bioinformatics/btv351

[evy239-B71] Simpson JT, et al 2009 ABySS: a parallel assembler for short read sequence data. Genome Res. 19(6):1117–1123. 19251739 10.1101/gr.089532.108 PMC2694472

[evy239-B72] Sonoda E, Hochegger H, Saberi A, Taniguchi Y, Takeda S. 2006 Differential usage of non-homologous end-joining and homologous recombination in double strand break repair. DNA Repair 5(9–10):1021–1029. 16807135 10.1016/j.dnarep.2006.05.022

[evy239-B73] Sperlich D, Pfreim P. 1986. Chromosomal polymorphism in natural and experimental populations. In: Ashburner M, Carson HL, Thompson JN Jr, editors. The Genetics and Biology of Drosophila. 3e. Academic Press, London. p. 257–309.

[evy239-B74] Spicer GS, Bell CD. 2002 Molecular phylogeny of the *Drosophila virilis* species group (Diptera: Drosophilidae) inferred from mitochondrial *12S* and *16S* ribosomal RNA genes. Ann Entomol Soc Am. 95:156–161.

[evy239-B75] Throckmorton LH. 1982 The *virilis* species group In: Ashburner M, Novistky E, editors. The genetics and biology of Drosophila. London: Academic p. 227–297.

[evy239-B76] Van der Auwera GA, et al 2013 From FastQ data to high‐confidence variant calls: the genome analysis toolkit best practices pipeline. Curr Protoc Bioinformatics. 43(1110):11.10.1–33. 25431634 10.1002/0471250953.bi1110s43 PMC4243306

[evy239-B77] Vieira CP, Almeida A, Dias JD, Vieira J. 2006 On the location of the gene(s) harbouring the advantageous variant that maintains the *X/4* fusion of *Drosophila americana*. Genet Res. 87(3):163. 16817999 10.1017/S0016672306008147

[evy239-B78] Vieira J, McAllister BF, Charlesworth B. 2001 Evidence for selection at the *fused1* locus of *Drosophila americana*. Genetics 158(1):279–290. 11333236 10.1093/genetics/158.1.279 PMC1461643

[evy239-B79] Vieira J, Vieira CP, Hartl DL, Lozovskaya ER. 1997 Discordant rates of chromosome evolution in the *Drosophila virilis* species group. Genetics 147(1):223–230. 9286682 10.1093/genetics/147.1.223 PMC1208106

[evy239-B80] Vieira J, Vieira CP, Hartl DL, Lozovskaya ER. 1998 Factors contributing to the hybrid dysgenesis syndrome in *Drosophila virilis*. Genet Res. 71(2):109–117. 9717433 10.1017/s001667239800322x

[evy239-B81] Waterhouse RM, et al 2017 BUSCO applications from quality assessments to gene prediction and phylogenomics. Mol Biol Evol.35(3):543–548. 10.1093/molbev/msx319 PMC5850278 29220515

[evy239-B82] Wittkopp PJ, et al 2011 Local adaptation for body color in *Drosophila americana*. Heredity 106(4):592–602. 20606690 10.1038/hdy.2010.90 PMC3183901

[evy239-B83] Zdobnov EM, et al 2017 OrthoDB v9.1: cataloging evolutionary and functional annotations for animal, fungal, plant, archaeal, bacterial and viral orthologs. Nucleic Acids Res. 45(D1):D744–D749. 27899580 10.1093/nar/gkw1119 PMC5210582

[evy239-B84] Zerbino DR, Birney E. 2008 Velvet: algorithms for de novo short read assembly using de Bruijn graphs. Genome Res. 18(5):821–829. 18349386 10.1101/gr.074492.107 PMC2336801

