# Supplementary material for: Origin and Consequences of Chromosomal Inversions in the virilis Group of Drosophila
Source: Genome Biol Evol. 2018 Oct 30;10(12):3152–66. doi: 10.1093/gbe/evy239 (PMC6278893; doi:10.1093/gbe/evy239)
Supplement: Supplementary Data [file evy239_supp.zip › File S1.pdf]

The purpose of this document is to guide the readers through the sequences available on Supplementary files 2-10.

1. Each file contains information about one inversion (Supplementary files 2-10, *Xa*; *Xb*; *Xc*; *2a*; *2b*; *2c*; *4a*; *5a*; and *5b*, respectively).

2. Each file contains sequences of the genes and intergenic regions of *D. virilis*, *D. novamexicana* (15010-1031-00) and *D. americana* (SF12).

3. The first sequences shown in each file are those referring to the ancestral state (*Xa* and *2a* - *D. novamexicana* (15010-1031-00) and *D. americana* (SF12); *Xb* - *D. virilis*; *Xc*, *2b*, *2c*, *4a*, and *5b* - *D. virilis* and *D. americana* (SF12); and *5a* - *D. virilis* and *D. novamexicana*).

4. The derived sequences are shown immediately after the ancestral ones for the proximal and distal breakpoints of each inversion (*Xa* and *2a* - *D. virilis*; *Xb* - *D. americana* (SF12) and *D. novamexicana* (15010-1031-00); *Xc*, *2b*, *2c*, *4a*, and *5b* - *D. novamexicana* (15010-1031-00); and *5a* - *D. americana* (SF12)).

5. Sequence headers provide information about the Contig/Scaffold number and coordinates, as well as the name and orientation of the genes in *D. virilis* (*GJ**) and in *D. melanogaster*. Additional notes were included about Contig breaks in *D. americana* (SF12) and *D. novamexicana* (15010-1031-00).

6. The color code used is the same as in Figure 3.
