# Supplementary material for: Origin and Consequences of Chromosomal Inversions in the virilis Group of Drosophila
Source: Genome Biol Evol. 2018 Oct 30;10(12):3152–66. doi: 10.1093/gbe/evy239 (PMC6278893; doi:10.1093/gbe/evy239)
Supplement: Supplementary Data [file evy239_supp.zip › File S10.pdf]

Ancestral state:

D. virilis

Distal region

>Dvir_scaffold_12823: 583,369.. 585,256 (*GJ17920*[-](*ADD1*) – *GJ18358*[+](*PI31*))

CCGTAAAATCAAAATCAAAGGCTTTGAGTGAAGGCGATGGCGTCGCTGGCGTTGTGACCGTCAG
CACAGCCTCGCCGCTGGGCGTGCTGCTCAT~~TTTGCCGGCTACGATAGTCAAGACTATCCACACG~~
~~TGGATTTGCAATCGCAATCAAGAAATATCTTCTTTCTTCACTCTCTTACGAACAAATATATATA~~
~~TATATATATGTATATATATATATACCTACACACACACACACGGACACACACTAGACTACTGCAA~~
~~AATGCTGGCTAACCAGCAGCGGCAGCAACAACGCGTCGCGGTATTTTTTGCAATAATAAATAAC~~
~~ACTAATACCAATGCATGCCCTACATATAT~~AAAATGGCGGCAATGCCGTAAAATGATCCCTGGCC
GTTTTGCAGTTTAATTCAAATGCTCTATTTTGATATTTTATATTAGCTCCATTTGCTTGGTCAA
CAGTATAAAAATGATTTTTCACGCGACTGCCGTTGTACTATCTCGTTTCCAATATGGCAGCCAC
TTCGTGTGAGCCGAGTGCACGAGCGAAACACGAAGTATCACAACAAGTTTTACCTAAAGTCTGG
GCCGATAATGGTAAAATATGTTATACATACACATTTCATGGATGGATATAGTTAAATAGTTGTA
CAAACTCGATATTATAATATACTTTATACCAACTTCAACAGCATAAAGTAATCTTAACTACCGA
TCGCATATAATACTCTTTCTGATTGATCATCATCAACATACGCGTAATTTGAATTTAAATGGGT
TTAATATCATAAATTATATTAATATTATAAATAATTTGCAAAGATTATAAAAAAGTTGATTAAA
ATGCGCAACTATACGCTTGGCAACTCTATCACCAACGTGTCGTCTTCTTCGCACTCATGCAACA
TTCCGTAAGGCACGAGCATAACGGAAAGGTAAATTATCAAAAATAAAGATGACTATATTTTATA
TTTATTATGATGTAATAATGTAAAAAAAGCTACAAAAGCATATTCAGTTGAATATATTTTTAAA
ATTTGAATTGAACAATCATCTAATATAATATATATGTTTTACAGCACTGGTGCACAAATGCAAC
ACTGAGTTTCAAATTGCCGGCACTAGCTTATCGTTTGCAGGCAGTAGCTAGCAGTCAAG~~CGTAC~~
~~TTAGAATTGTATACTTCAGAAAAATTGTGGAGAAGACCTACTCGGAGTTACACAAAAATATCAA~~
~~AACACTAAATGTGTTTTAATTAT~~GTAAGTGGCATACAAACATAGTGATGTTTACCAACGTCGGT
TTACAAATGTGTGACTTATGTTTGATTTCAATTGAACTGCGTTTTCAAAAAATTGCACTGAAAG
CGTTCTTAAGTCGCGTCCTTGCAGAATTTTGAAAAAAGCGAGGCCACTTAAAATAAAAGTATAC
ATACCTAGTATTTCTTATTATGTTATGTTATGTTATGTTATTAATATATAACTATGCGAGAATT
TTAGGCATGAGATGTGTTAAAACGCAGATGTGGATGCCTCTACTTAAAACGCATTTGTGGCTAT
CGATTATTTGTGTACGATAATTGATAGAGCTAAGGTTCGATTTCGTGCTGTC~~ATTGTTTTGGTT~~
~~TATTCTTCCGATTTTAACAAAAGTTTTAGGTTTCGTGTCCCTGTGAAATATGTTTCAATTTGAT~~
~~TTGTAATCTGTCTTTAAACATTATCTACAGTCATTAACAAA~~ATGGAGCCAACAACCTCCAGATC
TGCCGTCGAGTCGCTAAACTCCGACTTCTTTTACGGCTGGGATCTATTATACAAAACTGTAGAT
GGCATTATTGATAAGAAGGCAGATGTTCTCATGGTGCTTGCCCATTTTTTGCTGACCAAGCACT
ACAAATTCCGCTGCGTTGGTATTGGAGATGAC

Proximal region

>Dvir_scaffold_12875: 1,330,029.. 1,329,221 (*GJ21267*[-](*CG12134*) – *GJ21266*[-](*Adam*))

CGGCAATATAATCCACTATGCTGTCGGTCAT~~GTTTACGTTTGATATCAAGAAGAAGAAGAGATA~~
~~CACGTGCCTGACCAGA~~GTTACAGCTCAGTGAACAGAGTTGCATTTTAATGGTTTTTAAAAGAAC
ATTCACTGCCATATGGCATGAGTTTATGACGTCACAAGCTGAAAATTATCGACTGCAGTGTTCA
CTCGATTAATTTCGTTTCGAACGAGCCGATAACGATTATTTTAAAACAGTTTAAGCTTGAAAAC
CAAAAAAAAAAATTGAAGAATCCCAATTTGAGCTTGTTTTACAATTTAATATCGCTTTTTAATA
TGATACAAAAGTGAATTTACAAGAAGCCATTCATGTACCTAATTTATTGTATTTTTTTAAATCT
GAAAAATGTTCGTTGAGGTTTCTTGATTGCTTGCTTGACTTGACTTTAGTTAATCTATTAAATA
TATATGTATATATATATATATATATATTATTTTTACATGCATTTATGTCGGTATGT~~ATAATGGT~~
~~GTGTGTCTGTCCTGTCATACAAATATATATATATGCTTACACTTAAATGTACATATATGTATAT~~
~~TAATATGGCGCCTTCGAACAGCATTCTCAAAATCAACAAAAAAGGATTTAAATTCCAGCGCAAT~~
~~TTTCTTTAACAAGAAGAACTCTCTTCGACATCAACAAGCAACTATATATATATATATATGTGTA~~

~~TTCAATAATTAGTTATATGCGTTTATCTATTGATCCTATTAT~~TCACATGAAGTCATCGTAATCA
TCGGTAAAATCATTTCCATACTTTTGATAGTCATCAATATC

Distal region

***D. americana* SF12**

>SF12_Contig389: …12,187**..10236… (*GJ17920*[-](*ADD1*)-**
***GJ18358*[+](*PI31*))**

CCGTAAAATCAAAATCAAAGGCTTTGAGTGAAGGCGATGGCGTCGCTGGCGTTGCGACCGTCAG
CACAGCCTCGGCGCTGGGCGTGCTGCTCAT~~TTTGCCCGGCTACGATAGTCAAGACTATCCACACG~~
~~TGGATTTGCAATCGCAATCAAGAAATATCTTCTTTCTTCACTCTCTTACACACACACATGCACA~~
~~TGCACACACACACACACACACGCACGCACACTATCAGACTACCACACTAGACTACTGCAAAATG~~
~~CTGGCTAACCAGCAGCGGCAGCAACAACCGCTCGCCGTATTTTTTGCAATAATAAATAACACTA~~
~~ATACCAACGCATGCCCTATATATAT~~AAAATGGCGGCAACGCCGTAAATGATCTCTGCGCCGTTT
TGCAGTTTAATTCAAATGCTCGTTTTTGATATTTAATATTAGCTCCATTTGCTTGGTCGACAAT
ATAAAAATGATTTTTCACGCGACTGCCGTTGTACTATCTCGTTTCCAATATGGCAGCCACTTCG
TGTGAGCCGAGTGCACGAGCGAAACACGAAGTATACACAACAAGTTTTACCTAAAGTCTGGGCC
GATAATGTAAAAATATGTTATACATACACATTTCATGGATGGATATAGTTAAATAGTCGTACAA
AATCGATACTATAATATATATCCTTTATACTAACTTTAACTTAGAATAGCATAAAGTAACAATC
TTAACTACCGATCGCATATAATACTCTTTTTCTGATTGATCATCATCAACATAAGCGTAATTTG
AATTTAATGCTTTTATTCGTGGTTTTAACATCATAAATTATATTAATATTATATTTAATTTACA
GAGATTCTAAAAAAGTTGATTAAAATGCGCAACTATACGCTTGGCAACTCTATCAGCAACGTGT
CGTCCTCTTCGCACTCATGCAACATTCCGTAAGGCACGAACATAACGGAAAGATAAATTATCAA
AAATAAAGATGACTATATTTTTTATTTATTATGATGTCATTATGTAGAAAACCAGCTACAAACA
CATATTCATTTGAATATATTTTTAAAATTTGAATAAAACAATCATCTACTAACATGCAAGTTTT
ACAGCACTGGTGCACAAATGCAACACTGAGTTTCAAATTGCCGGCACTAGTCTAGTTTTTTGTT
TGCAGGCAGTAGCTAGCAGTCGAG~~CGTAATACTGTATATTATATTACTAGAAAATTGTGGATAA~~
~~GACCTAATTGGATTACACAAAAAGATCAAAACACTAAATGTGTTTAAATTAT~~GTAAGTGGCATA
AAAACATAGAGATATTCGGCAACGTCGGTTTACAAATTTGTGACATATTTCATTTCAATTGAAT
TGCGTTCTCAAAAAAATGTATTTCCACTGAATGCGTTCTAAAGTCGCGTTCTTCAGAGAATTTT
AAATAGCGACGCCACATTAAAAATTTAAATACACCTAGTGTTTCTTATTGTTATTAATATATTC
TTTTTTATTACGATTAACTTCGATACGAAGAAAAAACTTTATGTGAGAATTTTAAGCAGGAGAT
GTATTCAAAAGCAGATGTCTCCGATGTCTGGATGTCTCTACTTAAAACGCATTTGTGGCTATCG
ATTATTTCTGTACGAAAATTGATAGAGCTTAGAGCTTCGATTTCGTGCTGTC~~ATTGTTTTGCTT~~
~~TATTCTTACGATTTTAACAAAAGTTTTAGGTTTCGTGTCCCTGTGAAATATGTTTCAATTTGAT~~
~~TTGTAATCTGTCTTTAAACATTATCTACAGTCATTAACAAA~~ATGGAGCCAACAACCTCCAGATC
TGCCGTCGAGTCGCTAAACTCCGACTTCTTTTACGGCTGGGATCTATTATACAAAACTGTAGAT
GGCATTATTGATAAGAAGGCAGATGTTCTGATGGTGCTTGCTCATTTTTTGCTGACCAAGCACT
ACAAATTCCGCTGCGTTGGTATTGGAGATGAC

Proximal region

>SF12_Contig1668:… 4,462.. 5,254… (***GJ21267*[-](*CG12134*) –**
***GJ21266*[-](*Adam*))**

CGGCAATATAATCCATTATGCTGTCGGTCAT~~GTTTACGTTTGATATCAAGAAGAAGAGATGCAC~~
~~GTGCCTAGCCCCGA~~GTTATAGCTCAGTGAACAGAGTTGCATTTTAATGGTTTTCAAAAGAACATT
CACAGCCATATAACACGAGTTTATGACGTCACAAGCTGAATATTATCGACTGCAGCATTCATTC
GATCAATTTCGCTTTTGACGAGCCGATAACGATTATTTAAAAACAGTTTAAGCTTAAAAAGCAA
AAAAGAAAAAACTGAAAAATCCCAACTTGAGCTTGTTTTACAATTTAATATCGCTTTATAATAT
TATACAAAAGTGAATTTACAAGAAGCCATTCATGTACCTAATTTATTGTGTTTTGTTTTAATCT
GAAAAATGTTCGTTGAGGTTTCTTGATTGCTTGCTTGACTTGACTTTAGTTATTCTATTAAATA
TATATGTATATATATATAATTTTTACATGCATTTATGTCTGTATGT~~ATAATGGTGTGTGTCTGT~~
~~GCTGTCATACAAATATATATATATGCTTACACTTAAATGTACATATATGTATATTAATATGGCG~~

~~CCTTCGAACAGCATTCTCAAAATCAACAAAAAAGGATTTAAATTCCAGCGCAATTTTCTTTAAC~~
~~AAGAAGAACTCCCTTCGACATCAACAAGCAACTATATATATATATATATTCAATAATTACTTAT~~
~~ATGCGTTTATCTATTGATCCTATTAT~~TCACATGAAGTCATCGTAATCATCGGTAAAATCATTTC
CATACTTTTGATAGTCATCAATATC

2c inversion

D. novamexicana 15010-1031.00

Distal breakpoint

>Nova00_Contig1098: …17,583..14,947… (*GJ17920*[-]*ADD1* – *GJ18358*[+](*PI31*)5’UTR - *GJ21267*[+](*CG12134*)

CCGTAAAATCAAAATCAAAGGCTTTGAGTGAAGGCGATGGCGTCGCAGGCGTTGCGACCGTCAG
CACAGCCTCGGCGCTGGGCGTGCTGCTCAT~~TTTGCCGGCTACGATAGTCAAGACTATCCACACG~~
~~TGTATTTGCAATCGCAATCAAGAAATATCTTCTTTCTTCACTCTCTTACACACACACATACACA~~
~~CACACACACACACACACTATCAGACTACCACACTAGACTACTGCAAAATGCTGGCTAACCAGCA~~
~~GCGGCAGCAACAACGCGTCGCCGTATTTTTTGCAATAATAAATAACACTAATACCAACGCATGC~~
~~CCTACATATAT~~AAAATGGCGGCAACGCCGTTAATGATCTCTGCGCAGTTTTGCAGTTTAATTCA
AATGCTCGATTTTGATATTTAATATTAGCTCCATTTGCTTGGTCGACAAAATAAAAATGATTTT
TCACGCGACTGCCGTTGTACTATCTCGTTTCCAATATGGCAGCCACTTCGTGTGAGCCGAGTGC
ACGAGCAAAACACGAAGTATACACAACAAGTTTTACCTAAAGTCTGGGCCGATAATGTAAAAAT
ATGTTATACATACACATTTCATGGATGGATATAGTTAAATGGTCGTACAAAATCGATACTATAA
TATATATATATTTTATACAAACTTTAATTTAGGATAGCATAAAGTAACAATCTTAACTACCGAT
CGCATATAATACTCTTTTTCTGATTGATCATCATCAACATAAGCGTAATTTGAATTTAATGGTT
TTATTCATGGTTTTAACATCATAAATTATATTAATATTATATATAATTTACAAAGATCTAAAAA
AGTTGATTAAAATGCGCAACTATACGCTTGGCAACTCTATCAGCAACGTGTCGTCTTCTTCGCA
CTCATGCAACATTCCGTAAGGCACGAGCATAACGGAAAGGTAAATTATCAAAAATAAAGATGAC
TATATTTTATATTTATTATGATGTAATAATGTAGAAAATCAGCTACAAACACATACATATTCAT
TTGAATATATTTTTAAAATTTGAATAAAACAATCATCTACCAACATGCACGTTTTACAGCACTG
GTGCACAAATGCAACACTGAGTTTCAAATTGCCGGCACTAGTCTAGTTTATTGTTTGCAGGCAG
TAGC~~TATTTCTAGAAAATTGTGGATAAGACCTAATTGGATTTACACGAAAAGATCAAAACGCTA~~
~~AATGTGTTTTAATTAT~~GTAAGTGGCATAAAAACATTGAGATATTCAGCAACGTCGGTTTATAAA
TTTGTGACTTATATTTCGGCGGTGACTTAGCGTCGAATTTTGGTACTTGTTATCGGAAAAGTAT
CGGTCACTTTCAAATGCATTTATTTATGTTTTTAAGCATGAAACCAAAAAAAATAAATGCAGAA
TTGCAAAAAATTAAATTATTTACTATCTACATTCATTTATTATTGAACTTTAATTTATAATATG
GGTAAACAAAGGGGTTGAAAAGTTAGGTTTTCACCATTCATTAAATATCTTAATTTAGGGATAA
TTTAGGGAAGCTTTACGCATTAATAAGCAAAATGATTGATAATTCGCGATCTATATGTTAAACT
ACCGTGCATTTGGGGCCATTATCGGAGAAGTAGAATAATAAAGGTTGAAATATTGCCACCTTTT
CGTTATTATCCGTAAATAAATACTTAAATGCATAGTTGTCGTCTTTTTCAACAGTCTTTTTTAC
CAAAGATAATGTTTTATTGTAAAATGACAATTATTTCATATATTACTGCATTTATTTTTCATAT
ACAAGTATTTTGAATTAATTTGTTCCCGCTCAATACTGTATAGCATTGTTCAGCATTGATTCCG
AGCAGTGGTTCAGTTCCCGTTGGAGCTAGTAGCTCGATAGGCCTTTTGAATGAACTACATGAGG
TGTTTACATTTTGTTATTTTTCTAAAAAACTGGCTGATCGATTTGTTTAGAATTAATGCGTAAA
GCTTCCTTAAACATGTATCTTCAAGTTGGTATACCGTGCATTGGGGCCGTTATCCCTGGGAAAA
GATATTAGGGTTCAAGTGCAAGGGTGATTTTTTACGGTCATTTTGTATGGGGCTAGCAACGAGG
TTGCTGCACTGTAGTACAAATATCTTAAAAACGTGCTGATCGATTTTTATAGTTTTTTCAAGAT
TAGTTAGGTGAAACTATAGACTTCAATCGTGTGTATGCGGTTTTCGGGGCCATTATCCCTAAAT
TAAGATATTTAAAGAATGGTGAAAACCTAACTTTTCAACCCCTTTGTTTACCCATATTATAAAT
TAAAATTCGAAAATAAATGAATGTAGATAGTAAATAATTTAATTTTTTGCAATTCTGCATTTAT
TTTTTTTGGTTTCGTGCCTAAAAACATAAATAAATGCATTTGAAAGTGACCGATACTTTTCCGA
TAACAAGTACCAAAATTCGACGCTAAGTCACCGCCGCTAATTTGACGCACATCATAAACTCGTG
TTATATGGCTGTGAATGTTCTTTTGAAAACCATTAAAATGCAACTCTGTTCACTGAGCTGTAAC

~~TCTGGCCAGGCACGTGCATCTCTTCTTCTTGATATCAAACGTAAAC~~ATGACCGACAGCATAATG
GATTATATTGCCG

Note: the DAIBAM was inserted in the 5’UTR of PI31

Proximal breakpoint

>Nova00_Contig427: …46,401.. 44,886… (*GJ18358*[-](*PI31*) – *GJ21266*[-](*Adam*)

GTCATCTCCAATACCAACGCAGCGGAATTTGTAGTGCTTGGTCAGCAAAAAATGAGCAAGCACC
ATCAGAACATCTGCCTTCTTATCAATAATGCCATCTACAGTTTTGTATAATAGATCCCAGCCGT
AAAAGAAGTCGGAGTTTAGCGACTCGACGGCAGATCTGGAGGTTGTTGGCTCCAT~~TTTGTTAAT~~
~~GACTGTAGATAATGTTTAAAGACAGATTACAAATCAAATTGAAACATATTTCACAGGACACGA~~
~~AACCTAAAACTTTTGTTAAAATCGTAACAATAAACCAAAACAAT~~GACAGCACGAAATCGAAGCT
CTAAGCTCTATCAATTTTCGTACACAAATAATCGATATCCACAAATGCGTTTTAAGTAGAGGCA
TCCACATCTGCATTTTAATACATCTCATGCATTAAATTCTCAATGTGCGTCAAATTAGTGGCGG
TGACTTAGCGTCGAATTTTGTTACTTGTTATCGGAAAAGTATCGATCACTTTCAAATGCATTTA
TACGACGTGCGTCAAATTAGCGGCGGTGACTTAGCGTCGAATTTTGGTACTTGTTATCGGAAAA
CTATCGGTCACTTTCAAATGCATTTATTTATGTTTTTAAGCACGAAACCAAAAAATTTAAATGC
AGAATTGCAAAAAATTAAATTATTTACTATCTGCATTCATTTATTTTTGAATTTTAATTTATAA
TATGGGTAAACAAAGGGGTTGAAAAGTTAGGTTTTCACCATTCTTTAAATATCTTAATTTAGGG
ATAATGGCCCCGAAAACCGCATATACACGATTGAAGTCTATAGTTTCACCGCTATAGTCTATAG
TTTCAACGCCGCTAGTTTGACGCACATGAGTTTATGACGTCACAAGCTGAATATTATCGACTGC
AGCGTTCATTCGATCAATTTCGCTTTTGACGAGTCGATAACGATTATTTAAAAACAGTTTAAGC
TTGAAAAGCAAAAAAAAAAGCTAAAAAATCCCAACTTGAGCTTGTTTTACAATTTAATATCGCT
TTATAATATTATACAAAAGTGAATTTACAAGAAGCCATTCATGTACCTAATTTATTGTGTTTTT
TTTTAATCTGAAAAATGTTCGTTGAGGTTTCTTGATTGCTTGCTTGACTTGACTTTAGTTGTTC
TATTAAATATATATGTATATATATTTATAATTTTTACATGCATTTATGTCTGTATGT~~ATAATGG~~
~~TGTGTGTCTGTGCTATCATACAAATATATATATATGCTTACACTTAAATGTACATATATGTATA~~
~~TTAATATGGCGCCTTTGAACAGCATTCTCAAAATCAACAAAAAAGCATTTAAATTCCAGCGCAA~~
~~TTTTCTTTAACAAGAAGAACTCCCTTCGACATCAACAAGCAACTTTATATATATATATATATAT~~
~~ATATTCAATAATTAGTTATATGCGTTTATCTATTGATCCTATTAT~~TCACATGAAGTCATCGTAA
TCATCGGTAAAATCATTTCCATACTTTTGATAGTCATCAATATC
