# Supplementary material for: Origin and Consequences of Chromosomal Inversions in the virilis Group of Drosophila
Source: Genome Biol Evol. 2018 Oct 30;10(12):3152–66. doi: 10.1093/gbe/evy239 (PMC6278893; doi:10.1093/gbe/evy239)
Supplement: Supplementary Data [file evy239_supp.zip › File S2.pdf]

Ancestral state:

D. novamexicana 15010-1031.00

Distal region

>Nova00_Contig329:…247..1
(*GJ18516*[-](*eIF2B-β*) - *GJ19143*[+](*Csat*))

CCCATTTTGACGGCGTGTATCAGTTGTGTTACCTCGTTTAACGGCTGTTCACGCAT~~TTTGACAA~~
~~TGTATTTTTTTTTTTGTTACAATGCAATAAGTTAGTCGGTGTTAATAACAATTAGCACAGGCAA~~
~~CGTTGGCCCAACAAAAACAAGTTGCAATCG~~ATGCCAGCAGCTGCAAAACTACTCGTACTCGATG
CCCATTGTCGTTGCAATTGTTATCGATATCGGAGGCATTC~~CATTGGGCAATAGCC~~

Note: 15 nt of the 5'UTR of Csat are in this contig; the remaining sequence is located in Contig 3993 without overlap with Contig 329

Proximal region

>Nova00_Contig2549:…1,810..5,021…
(*GJ18515*[+](*hang*) - *GJ19142*[-](*CG9947*))

GAAGACGACGACGACGACGATGATGATGACGACGAAGATGATGATGACGACGACGATGATGGCG
ATGTGGACGATGACGATGACGAAGATATTGTGGAACAGCAACTGCTGCAACAACAACAACAACA
ACAGTGTCTCAATAGCAACAACAAGAAGAAGAACAACAACAATAATAACAACAACAACAACAAC
AGCGTAGTTGCAAATGACGATGACGATGATCTCATTGAGGAGGTAATCGAGGATGATGGCATTG
TCGAGGAGCTAGAAGATGATGAGGACGATGACGAGGACGACGACGAGGCCGAGGCCGATGGCGT
TGATGATGATGACGACGATGGTAACACAACGGATGAGCATCAGACGCTTCCTTCCAATGCCACC
AAACAGCAGCCGAGGCGCCTCAACTTTGACTACGCGCGCACAGACAGCTCCAAGCTGAACGGCA
ATAGCAATGGCAAACAGACGCCGCAGCATCAGCAAAAGTCAAAGGGCCAGCAGGCGGTGTTGGT
CGTGCACAGCTCAGAGGATGAGGATGAGGCTGATGTCGATGGGAACGGTGATGAGGATGTGGAT
GAGGAGGAGGAGGAGGAGGATGAGGGCGTGGGTGAAGCAATGACTATTGATGATATTATTGAAG
AAGATGATGGGGAGGATGATGACGATGTGGTTGGTGTCGATGGTGGTGTTGTTGAGGATGATGA
TGATGATGTTGACGAAGATATGGACGAGGATGACGTTGACGACGACGACGACGACGTCGACGAC
GACGACGGCGAAGATGATGATGAGGGTGGTGCTGGTGCTGGTCGACGCACTGGCGCCGCCAGTG
GCAGTGGCAGTGGTAGCGGCAGCGGCAGTCGCATCGGCGCGTCTGATGGCGTGGCTGCTGATGC
CGATGGTGGTACATCATCATCTGAGAGCGAGTCAACAACAACAACAACATCGCATTCAATTGGT
GAGCGACGTAAAAAAACAGTAGTTAAATCAGCGGCACATGGCGCTGCCGCTGATCAGTCTAATT
CCAGCTATACGTGTGATCTATGTCAACTTTGTTTCGATTCTCAGGAGTTACTGCAGTCACATAT
TAAAAGCCATTTTCTGAATGGGCCGTCGGCGGGCAGCAGCAGCGCCAGCGGCAGCAGTAGCAAC
AGAAGCGCCAGCCTCGGCGGCGGCGGCGGCAGCGGCAGCGGCAGCAGAAGCAACAATAATAATA
ATAACATCGACAGCAGCAGCAGCGCCAGCGGCAGCGGTAGCAGCAGCAATAACAAAACTAAAAC
CAAAAAGTCCGGCTTGGATGCAATCAAAAGTGGCAGTGCTGCAACAACGTCGACAGCAGCAACA
ACAACAACAACAGCAGCAAAAGTTGCAGCCGAAGCAGCTGCAACAACAGCAGCAGCGGACGCTG
CAACCAGCTTGAACTGAGCTTGAAAAAAATGTACAAAAGCAAAAACAAAACCACACCGCACACA
TCCGCAACGCATTCCATTGCCAAACACAACATACACACACTCGCGCACATCCACACATCCATAC
ATCCTCACACTCGCACACACACACTTGCTCACGCTCACGCATATGTATAAAAAATAGAAAACTG
GGTGTGTATGTGTGCAATACCATTTTTTTTTTCTTGCTGTGCTAATTGCAGTGTTGGTAAACTT
CTACCGCATATTAACTGCAAAATACAGATACACACACACACACACACACACACAAGCAGACATA
TTAAGCTTACATATTTGATATAACATGAATACATATATCCGTACGTACATACATATATATATAT
AAATATATATAAAATACTTTTTTTTTTACATGTTAAATTAGTTGGTATCGCAGACAACAGACAG
GCGCGGGAGTGTCTCCAGTTTTGAGTTTTAGTTAGTTAAGCGATATATATATATAAATATAAAT
ATATATATATACAATAAATACAAATACAATGAATACAAATATTTAAGAGGTTAATGAAAAATTA
GAATTGTCAAGCAAAAAATTACGACGATGACGACGCACCAAAGCAAACACAAACTGTAGAAGGC
AAAAAAACAAACAAAAAAACAAAATAGGCAAACAAAAGCTAAAATTCGTTTAGTTGTTAACGT

ACTTTAATTTTATTCCATTTTATTTTTATTGCTTTTTTTTTTGCTGTTTTTAGCTTCTGGTTTA
AAAAACCACTTTTCAGTTTTATATTTTCTCCTTTTTTTTTTTTTTTTTGTTTATTTTTTGATAA
GTGTATATATTTGATAACATTTTAGTGCGTTGCTCTAATTTTGAAATTATCTCAAGGCAATTCC
AAGTTTACAAATTAATTTAACGTCTGTTGGGACAGTAAATCGTTAACTCTTTCGACTCGCATAG
TTTAAGTTACAAAACATTTATTAATTTCACACACACACACACACACACACACATAGAGAGAGGA
AAACCCTCACCTACGAGCACCAGCATACCATGCCCACCAGAGACGCATTGGGAATATTATAATT
TCTTAATATTTATTTAAAATGTAAATTCAATATAATTTTGTTTAATTTGTTTCAACAAAAAACC
AGTTCAGAAGGAAATGAATCATTAATTTTGCAGGCTTACAAATCAAGAAAAAGAACAACAGCAC
AGATCGTTCGGATATCCATCCAAGTCTGATAAAATTTTGATATAAAAAGTTAACTTTTGCCATA
AACGCAGCATTAGCACTTTGCCCCATTTAAACACTTAGTATGTAATGTGTATATTAATTATTTA
TATTTAATGATGCATTTCACAAATAATTCGTTTCATGCTTCATTATTCAGATAAATCATATTCG
TATGTATGTGTATACACATTCGTAGATAAACAAAACACAAAAAAAAAGAAAGAAAAAAAAATAGT
GCATACTGCATATGTTAATTACGCTTACGAATTAATTAATTATTGCGATTGCAAACACTTACCT
TTTACTTGTAAAATTATTTTTAAGGTTTATGTATGTAATTGGAAGCTAGTTTAAAACCAGATAC
AAATAAAAAGAATCGTGAATGAAACAAATGCATCTGCATTTGCCGAATTGAATACCTTGAACAT
TTTGAATTTCTGTTACAACATTGAAAAGAAAAAACAAATTGCTGCGCGCCAAGTTCCGATAACA
GCTTTGGCAGCGACTGCTATCCAAAAATATATCGATATCGTTCTAGCTAACACGAGAACACGCT
AGTCGCAAAGCTAACTGTAATGTAAAGTATTTGCTGGGCTTTTGGAGTCGTGCTTACCGTGTTG
GTGTGTTGGTAAGGTTAAAGCACGGTCCATTAACATCATTAGCCAACACTGCTGCAAGCATTTA
CTATGTGCTTTTCCCATGCTCGACTTGTGCAACAGACAACTCGATGGAAAGAATAAAAAGAATT
TCTTTGTTTGAATCAACTTGTATTGCACAAT~~TTTAATAAAATATCAATGCATATTTATAAATAT~~
~~TTCTATAGCCGTCTGCTTTAAACGGCTGCCATACAAAACATATGTGTTAATGCCAAATTGCGCCA~~
~~CAGGCACTCATAGTCTAAAGTTTAATTGTTTAAATATTTTCTATGCATTTATGTGCGGAAACTT~~
~~TTCTAATTTATCTGGATGAGGCAATAGTATTATTATAGAAGTCGCGTATGTTATCAGATGCATT~~
~~ACAAGACGAATCATTAACAAACATACATATAATTAAAAGTGCAAAAACAAAAAAAAAAAACATT~~
~~ACAAAATGTTAACAAGAAGCACAGCCAACGATGCCCGTACAGTGTCATAAATACTAATAGAAACT~~
~~AATTGTGTGTATTCTGTTACAAAGATATACATAGATAATTATGCTTAAACAAGATATTATAGCT~~
~~AAATAATGGTGCATGGCCGAGAGAAGCTTTTGCCAAAATTACTCTCAAATCGAATCCATTGCTT~~
CTAGGAGTACTGTGTATGTGGATTAACGTTTATCATCTCCATGTTGCTG

D. americana SF12

Distal region

>SF12_Contig5507:…4703..4138…
(*GJ18516*[-](*eIF2B-β*) - *GJ19143*[+](*Csat*))

CCCATTTTGACGGCGTGTATCAGTTGTGTTACCTCGTTTAACGGCTGTTCACGCAT~~TTTGACAA~~
~~TGTATTTTTCCTTGTAACAATGCAATTAGTCGGTGTTAATAACAAATAGCACAGCACAACGTTG~~
~~GCCCAACAAAAACAAGCTGCAATCG~~ATGCCAGCAGCTGTAAAACTACTCGTACTCGATGCCCAC
TGTCGTTGCAATTGTTATCGATATCGGAGGCATTC~~CATTGGGCAATAGCCATACAAAAAAAAAA~~
~~AAAAAGCAAAAGCAAAAACAACAATGCACGATAAAACTGGACGCAATATGAGCTGAAAGCTTGT~~
~~TTACGTGTCCGTGCCTTCTTCATAGCCGAAAAATCGCCGAAGAAGCAACGCGCACGGCAGGCGG~~
~~GCAGAGCGTACTGTCTAAGCAATTGTTTATCACCGAAACAGGTGTTTCCCTTTATTATTATTTT~~
~~CTCTTTTTTTTTGTTTTTGTTTTCGTTTTTGTTTGGCTTAAACACTATTTTGCCTTAAAGTCAC~~
~~GCTTTGCCCGGTTCTGATACCAAAACAGACGCCAAGCGCAAA~~ATGAATATACACG

Proximal region

>SF12_Contig2063:… ..12,541…
(*hang*/*GJ18515*[+] - *CG9947*/*GJ19142*[-])

GACGACGACGACGACGACGATGATGATGACGACGAAGATGATGATGACGACGACGATGAGGGCG
ATGTGGACGATGACGATGACGAAGATATTGTGGAACAGCAACTGCTGCAACAACAACAACAACA
ACAACAACAGTGTCTCAATAGCAACAACAAGAAGAAGAACAACAACAATAATAATAACAACAAC

AACAACAGTGTAGTTGCAAATGACGATGACGATGATCTCATTGAGGAGGTAATCGAGGATGATG
GCATTGTCGAGGAGCTAGAAGATGATGAGGACGATGACGAGGACGACGACGAGGCCGAGGCCGA
TGGCGTTGATGATGATGACGACGATGGTAACACAACGGATGAGCATCAGACGCTTCCTTCCAAT
GCCACCAAACAGCAGCCGAGGCGCCTCAACTTTGACTACGCGCGCACAGACAGCTCCAAGCTGA
ACGGCAATAGCAATGGCAAACAGACGCCGCAGCATCAGCAAAAGTCAAAGGGCCAGCAGGCGGT
GTTGGTCGTGCACAGCTCAGAGGATGAGGATGAGGCTGATGTCGATGGGAACGGTGATGAGGAT
GTGGATGAGGAGGAGGAGGAGGAGGATGAGGGCGTGGGTGAAGCAATGACTATTGATGATATTA
TTGAAGAAGATGATGGGGAGGATGATGACGATGTGGTTGGTGTCGATGGTGGTGTTGTTGAGGA
TGATGATGATGATGTTGACGAAGATATGGACGAGGATGACGTTGACGACGACGACGACGACGTC
GACGACGACGACGGCGAAGATGATGATGAGGGTGGTGCTGGTGCTGGTCGTCGCACTGGCGCCG
CCAGTGGCAGTGGCAGTGGTAGCGGCAGCGGCAGCGGCAGTCGCATCGGCGCGTCTGATGGCGT
GGCTGCTGATGCCGATGGTGGTACATCATCATCTGAGAGCGAGTCAACAACAACAACAACATCG
CATTCAATTGGTGAGCGACGTAAAAAAACAGTAGTTAAATCAGCGGCACATGGCGCTGCCGCTG
ATCAGTCTAATTCCAGCTATACGTGTGATCTATGTCAACTTTGTTTCGATTCTCAGGAGTTACT
GCAGTCACATATTAAAAGCCATTTTCTGAATGGGCCGTCGGCGGGCAGCAGCAGCGCCAGCGGC
AGCAGTAGCAACAGAAGCGCCAGCCTCGGCGGCGGCGGCAGCGGCAGCGGCAGCAGAAGCAACA
ATAATAATAATAACATCGACAGCAGCAGCAGCGCCAGCGGCAGCGGTAGCAGCAGCAATAACAA
AACTAAAACCAAAAAGTCCGGCTTGGATGCAATCAAAAGTGGCAGTGCTGCAACAACGTCGACA
GCAACAACAACAACAACAGCAACAACAACAGCAGCTAAAGTTGCAGCCGAAGCAGCTGCAACAA
CAGCAGCAGCGGACGCTGCAACCAGCTTGAACTGAGCTTGAAAAAAATGTACAAAAGCAAAAAC
AAAACCACACCGCACACATCCGCAACGCATTCCATTGCCAAACACAACATACACACACTCGCGC
ACATCCACACATCCATACATCCTCACACTCGCACACACACACTTGCTCACGCTCACGCATATGT
ATAAAAAATAGAGAACTGGGTGTGTATGTGTGTGTGTGTGCAATACAATTTTTTTTTCTTGCTG
TGCTAATTGCAGTGTTGGTAAACTTCTACCGCATATTGACTGCAAAATACAGATACACACACAC
ACACACACACAAGCAGACATATTAAGCTTACACATTTGATATAACATGAATACATATATCCGTA
CGTACATACATATATATATATAAATATATATAAAATACTTTTTTTTTTTACATGTTAAATTAGT
TGGTATCGCAGACAACAGACAGGCGCGGGAGTGTCTCCAGTTTTGAGTTTTAGTTAGTTAAGCG
ATATATATATATAAATATAAATATATATATATACAATAAATACAAATACAATGAATACAAATAT
TTAAGAGGTTAATGAAAAATTAGAATTGTCAAGCAAAAAATTACGACGATGACGACGCACCAAA
GCAAACACAAACTGTAGAAGGCAAAAAACAAAAAAAAACAAACAAAAAAAAAACAAAATAGGCA
AACAAAAGCTAAAATTCGTTTTAGTTGTAAACGTACTTTAATTTTATTCCATTTTATTTGTATT
GCTTTTTTTTTTTGCTGTTTTTAGCTTCTGGTTTAAAAAACCACTCTTCAGTTTTATATTTTCT
CCCTTTTTTTTTTTTTGTTTATTTTTTGATAAGTGTATATATTTGATAACATTTTAGTGCGTTG
CTCTAATTTTGAAATTATCTCAAGGCAATTCCAAGTTTACAAATTAATTAAACGTCTGTTGGGA
CAGTAAATCGTTAACTCTTTCGACTCGCATAGTTTAAGTTACAAAACATTTATTAATTTCACAC
ACACACACACACAGAGGAAAACCTTCTCAGCATACCATGCCCACCAGAGACGCATTGGGAATAT
TATAATTTTTTAATATTTATTTAAAATGTAAATTCAATATAATTTTGTTTAATTTGTTTCAACA
AAAAACCAGTTCAGTAGGAAATGAATCATTAATTTTGCAGGCTTACAAATCAAGAAAAAAGAAC
AACAGCACAGATCGTTCGGATATCCATCCAAGTCTGATAAAATTTTGATATAAAAAGTTAACTT
TTGCCATAAACGCAGCATTAGCACTTTGCCCCATTTAAACACTTAGTATGTAATGTGTATATTA
ATTATTTATATTTAATGATGCATTTCACAAATAATTCGTTTCATGCTTCATTATTCAGATAAAT
CATATTCGTATGTATGTGTATACACATTCGTAGATAAACAAAACACAAAAAAAAAAGAAGAAAGA
AAAAAAATAGTGCATACTGCATATGTTAATTACGCTTACGAATTAATTAATTATTGCGATTGCA
AACACTTACCTTTTACTTGTAAAATTATTTTTAAGGTTTATGTATGTAATTGGAAGCTAGTTTA
AAACCAGATACAAATAAAAAGAATCGTGAATGAAACAAATGCATCTGCATTTGCCGAATTGAAT
ACCTTGAACATTTTGAATTTCTGTTACAACATTGAAAAAAAAAACAAATTGCTGCGCGCCAAGT
TCCGATAACAGCTTTGGCAGCGACTGCTATCCAAAAATATATCGATATCGTTCTAGCTAACACG
AGAACACGCTAGTCGCAAAGCTAACTGTAATGTAAAGTATTTGCTGGGCTTTTGGAGTCGTGCT
TACCGTGTTGGTGTGTTGGTAAGGTTAAAGCACGGTCCATTAACATCATTAGCCAACACTGCTG
CAAGCATTTACTATGTGCTTTTCCCATGCTCGACTTGTGCAACAGACAACTCGATGGAAAGAAT
AAAAAGAATTTCTTTGTTTGAATCAACTTGTATTGCACAAT~~TTTAATAAAATATCAATGCATAT~~
~~TTATAAATATTTCTATAGCGTCTGCTTTAAACGGCTGCCATACAAAACATATGTGTTAATGCCA~~
~~AATTGCGGCACAGGCACTCATAGTCTAAAGTTTAATTGTTTAAATATTTTCTATGCATTTATGT~~
~~GGGCAAACTTTTCTAATTTATCGGATGAGGCAATAGTAGTATTATAGAAATCGCCGTATGTTAT~~
~~CAGATGCATTACAAGACGAATCATTAACAAACATATACATATAATTAAAAGTGCAAAAACAAAA~~
~~AAAAAAAACATTACAAAATGGTAACAAGAAGCACAGCCAACGATGCCGTACAGTGTCATAAATA~~

~~CTAATAGAAACTAATTGTGTGTATTCTGTTACAAAGATATACATAGATAATTATGCTTAAACAA~~
~~GATATTATAGCTAAATAATGGTGCATGGCCGAGAGAAGCTTTTGCCAAAATTACTCTCAAATCG~~
~~AATCCATTGCTT~~CTAGGAGTACTGTGTATGTGGATTAACGTTTATCATCTCCATGTTGCTG

_Xa_ inversion:

D. virilis

Distal breakpoint:

>Dvir_scaffold_12472: 175,683..178,915 (*GJ18516*[-](*eIF2B-β*) - *GJ18515*[-](*hang*))

CCCATTTTGACGGCGTGTATCAGTTGTGTAACCTCGTTTAAGGGCTGTTCACGCAT~~TTTGACAA~~
~~TGTCTTTTTCTTTATAACAACAGCAATAAGTCGGTCGGTCTTAATAACAAATAGCACAGCCAAC~~
~~GTTGGCCCAACAAAAACAAGTTGCAATCG~~ATGCCAGCAGCTGTAAAACTACTCGTACTATGTGC
GTCAAGTTAGCGTCGAATTTTGGTACTTGTTATCGGAAAAGTATCGGTCACTTTCAAATGCATT
TATTTATGCTTTTTAGCACGAAACCAAATAAATTTAAATGCGAAATTGCAAAAAATTTAATTAT
TTACTATCTATATTCATTTATTTTTGAATTTTAATTTATAATATGGGTAAACAAAGGGGTTGAA
AAGTTAGGTTTTCACCATTCTTTAAATATCTTAGTTTAGGGATAATGGCCCCAAAAACCGCGTA
TACACGATTGAAGTCTATAGTTTCACCTAACCAATCTTGAAAAAATTATAAAAATCGATCAGCA
CGTTTTTAAGATATTTGTACTACAGTGCAGCAACCTCGTTGCTAGCCCCATACAAAATGACCGT
AAAAAATCACCTTTGCACCTTTGCAGGGATAATGGCCCTCAATGCACTGTATACCAACTTGAAG
ATACATGTTTAATGAAGCTTTACGCATTAATTCTAAACAAATCGATCAGCTGGTTTTTTAGAAA
AATAACAACATGTAAACACACCTCATGTTGTTCTTTCCCTAGGCCTATCGTTTGTTTGGCCTAT
CGCCATTGTTTGGTTAGTGGTGTTAGCTAGAACGCTATCGATATATTTATGGATAGAAATCGCT
GCCAAAGCTGTTATCGGAATTTGCCGCGCAATAATTAGTTTTTTGTTTTTTTTTTTTTTTCAA
CGTTGAAACAGAAATTCAAAATGTTCAAGGTATTCAATTCGGCAAATGCAGATGCATTTGTTTC
ATTCACGATTCTTTTTATTTGTATCTGGTTTTAAACTAGCTTCCAATTACATACATAAACCTTA
CAAATAATTTTACAAGTAAAAGGTAAGTGTTTGCAATCGCAATAATTAATTAATTCGTAAGCGT
AATTAACATATGCAGTATACACTATTTTTTTTCCTTCTTTTTTTTTCTTTGTGTTTTATTTATCT
ACGAATGTGTATACACATACATACGAATATGATTTACTTGATTAATGAAGCATGAAACGAATTA
TTTGTGAAATGCATCATTAAATATAAATAATTAATATACACAGTACATACTAAATGTTTAAATG
GGGCAAAGTGCTAATGCTGCGTCGTGTATGGCAAAAGTTAACTTTCTATTTCAAATTTTGTCAG
ACTTGAATGGATATCCGACCGATCTGTGCTGAATGAATTATTCATTTTCTGCTGAACTGGTTTT
TGTTTTTGTTGAAACAAATTAAACAAAATTATATTGAAAATACATTTTAAATAAATATTAAAAA
ATTATAATATTCCCAATGCGTCTCTGGTGGGCATGGTATGGGTGCTCGTAGGCGAGGGCTTTCG
TGTGTGTGCGTGTGCGTGTGTGTGTGAAATTAAATATGCTTTCATTTGTGTAGGCCTATGTTTT
GTAACTTAAACTATGCGAGTCGAAAGAGTTAACGATTTGCTGTCCGAACGGACTTTAAATTAAT
TTGTAAACTTGGAATTGCCTTGAGATAATTTCAAAATTAGAGCAACGCACTAAAATATTAAAAA
AATATATACACTTATCATAAAAAAAAAAAGAGAGAAAAAAATAAAACTGCAGGGTGGTTTTTTAAA
CCAGAAACTAAAATCAGCACAAAAAAAAAAAAGCAATACAAATAAAATATAATAAAATTAAAGTA
CGTTAACAACTAAAACGTAATTTAGCTTTTGTTTAACTATTTTTTGTTTTTTAGTTTTTGCGTT
TTTTTGCCTTCTACAGTTTGTGGTTTGCTTTGGTGCGTCGTCATCGTCGTAATTTTTCGCTTGA
CAATTCTAATTTTTCATTAACCTCTTAAATATTTGTATTCATTGTATATACGTATGTACGTATA
TATATATATATTAATATATATATATATATATATATATATCGCTTAATTAACTAAAACTCAAAACTG
GAGACACTTCCGCGCCTGTCTGTTGTCTGTGATACCAACTAATTTAACATGTAAAAAAAAAATT
ATATTATATATATTTATATATATATATATGTATGTATGTACGGATATATGTATTCATGTTATAT
CAAATATGTAAGCTTAATATGTCTGCTTGTGTGTGTTTGTGTGTGTGCATCTGTATTTTGCAGT
CAATATGCGGTAGAAGTTTACCAACACTGCAATTAGCACAGCAAGAAAAAAAAATTGTATTGCAC
ACACACACACACATACACACCCAGTTCTCTCTATTTTTTTTTATACATATGCGTGAGCGTGTGCA
AGTGTGTGTGTGTGTGCGAGTGCGCCAATGTGAGGATGTGAGGATGTGAGTGTGTGTGTGTGTA
TTTGGCAATGGAATGCGTTGCGGATGTGTGCGGTGTGGTTTTGTTTTTGCTTTTGCTTTTGTAC
ATTTTTTTTCAAGCTCAGTTCAAGCTGGTTGCAGCGTCCGCTGCTGCTGTTGTTGCAGCTGCTT
CAGCTGCAACTTTTGCTGCTGTTGTTGTTGTTGTTGTTGTTATTGTTGCTGTCGACGTTGTTGC

AGCACTGCCACTTTTGATTGCATCCAAGCCGGACTTTTTGGTTTTGGTTTTGTTGTTACCGCTG
CCGCTGCCGCTGGCGCTGGCGCTGCTGCTGTTGCTGCTGTCGATGTTATTATTATTGTTGCTTC
TGCTGCCGCTGCCTGTGCCGCCGCCGAGGCTGGCGCTTCTGTTGCTACTGCCGCCGCTGGCGCT
GCTGCTGCCCGCCGACGGCCCATTCAGAAAATGGCTTTTAATATGTGACTGCAGTAACTCCTGA
GAATCGAAACAAAGTTGACATAGATCACACGTATAGCTGGAATTAGACTGATCAGCGGCAGCGC
CATGTGCCGCTGATTTAACTACTGTTTTTTTACGCCGCTCACCAATTGAATGCGATGTTGTTGT
TGTTGTTGACTCGCTCTCAGATGATGATGTACCACCATCGGCATCAGCAGCCACGCCATCAGAC
GCGCCGATGCGACTGCCGCTGCCGCTACCACTGCCATTGCCAGTGGCGGCGCCAGTGCGACGAC
TAGCACCAGCACCACCATTATCATAATTTTCGCCGTCGTCGTCGTCGACGTCATCATCGTCGTC
GTCGTCGTCTTC

Proximal breakpoint

>Dvir scaffold_12970: 2,159,573.. 2,157,822 (*GJ19143*[-](*Csat*) - *GJ19142*[-](*CG9947*))

~~CGTGTATATTCATTTTGCGCTTGGCGTCTGTTTTGGTATCAGAACCGGCAAAGCGTGACTTTAA~~
~~GGCAAAATAGTGTTTAAGCCAAACAAAAACGAAAACAAAAACAAAAAAAAACGGAAAGAGAGAA~~
~~AATAATAATAAAGGGAAACACCTGTTTCGGTGATAAACAATTGCTTAGACAGTACGCTCTGCCC~~
~~GCCTGCCCGTGCGCCGTTGCTTCTTCGGCCGATTTTTCGGCTATGAAGAAGGCACGGACACGTAAAC~~
~~AAGCTTTCAGCTCATATTGCGTCCAGTTTTATCGTGCATTGTTGTTTTTGCTTTTGCTTTTTTT~~
~~TTAAATGGCTATTGCCCAATG~~GAATGTCTCCGATATCGATAACGATTGCAACGACAATGGGCAT
CGAGTACGAATGTGCGTCAAATTAGCGGCGGTGAGTTAGCGTCGAATTTTGTTACTTGGTATCG
GAAAAGTATCGGTCACTTTCAAATGCATTTATTTATGTTTTTAAGCACGAAATGCAGAATTGCA
AAAAATTTAATTATTTACTATCTACATTCATTTATTCTTGAATTTTAATTTACAATATGGGTAA
ACAAGGGGTTTTTTTTTGCATAACAGGGGGTATAATAAGAAAATTAGAATAATGCGAATTTCTC
AAAAACTGGCTGGACGATTTTCTTGATGTAAAAAGCAAAATGGTTGATAATTCGCGATCGGAGA
AATAGAATAATAAAGGTTGAAATATTGCCACCTTTTCGTTATTATACGTGTATAAATACTTAAA
TGCATAGTTGTCGTCTTTTGCAGCAGTCTATTTTACCAAAGATAATGTTTTATTGTAATATGAC
ATATATTATTGTAATATATGAAATAATTTCATATATTACTGCATGTCTTTTCCATATACAAGTA
TTTTGAATTAATTTGTTCCCGCTCAATACTGTATCACATTGTTCAACACTGATTCCGACATCAC
ATGCTAGTCGCAAAAGCTAACTGTAATCACATATGTTGTTTGGTAAGTGCTGTAATGTAAAGTA
TTCGCTAGACGTTTGAGACCGCGCTTACCGTGTTGGTAAGGTTCAAGCACGGTCCATTAACATC
ATTAGCCAACACTGCTGCAAGCATTAACTGTTTGCTTTTCCCATGCCCGACTTGTGCAACAGAC
AGCTCGATTGAAAGAATAAAATCAATTTCTTTGTTTGGATCAACATGTATTGCACAAA~~TTTAAT~~
~~AAAATATCAATGCATATTTATAAATATTTCTATAGCGTCTGCTTTAAACGGCTGCCATACAAAA~~
~~CATATGTGTTAATGCCAAATTGCGGCACAGGCACTCATAGTCTAAAGTTTAATTGTTTAAATAT~~
~~TTTCTATGCATTTATGTGGGAAACTTTTCTAATTTATCAGGATGAGGCAATAGTAGTACTATA~~
~~GTAGTCGCGTATGTGATCAGATGCATTACAAGACGAATCATTAACAAACATTACATACATATAA~~
~~TTAAAAGTGCAAAAACAAAAAACAAAAAAAAAAAACATTACAAAATGGTAACAAAAGGCACAGC~~
~~CAACGATGCCGTACAGTGTCATAAATACTAATAGAAACTAATTGTGTGTATTCTGTTACAAGGA~~
~~TATACATAGACAATTATGCTTAAACAAGATATTATAGCTAAATAATGGTGCATGGCCGAGAGAA~~
~~GCTTTTGCCAAAATTACTCCCAAATCGAATCAATTGCTT~~CTAGGAGTACTGCGTATGTGGATTA
ACGTTTATCATCTCCATGTTGCTG
