# Supplementary material for: Origin and Consequences of Chromosomal Inversions in the virilis Group of Drosophila
Source: Genome Biol Evol. 2018 Oct 30;10(12):3152–66. doi: 10.1093/gbe/evy239 (PMC6278893; doi:10.1093/gbe/evy239)
Supplement: Supplementary Data [file evy239_supp.zip › File S3.pdf]

## Ancestral state:

### *D. virilis*

### Distal region

>Dvir_scaffold_12932: 1,328,257.. 1,331,034 (*GJ14731*[-](*CG8206*) - *GJ14858*[+](*CG15896*))

~~TTCGATTTGAGTTACTCATAGCTGGTTTGACCGGCCATAACAAATTATATTTAGCCGTCGGCTC~~
~~TCATATATGTACATAACAAAACAAAAAACAAAAGAAGGTAAAATTGTTGTTTAGGTAGGTTGTT~~
~~ACTCTCTCTCTCTCTCGCTCGCTCTTTTCTGTCACCCTTTCGTTCGTTCCGAGTGTTGACAC~~
~~GACGACACTGCATCGCTGTCCTGGCTGGAACTGGAA~~CTAGATTAGTTCACCGTGCTCGTTCACT
TTGATGATGCGCACTCGCTCCTGTCGCTTCGATAGCACCTCATCGATATTCATGCTGCTGCCAT
CACTATTGGCGAACAGGCCCAGATTGTCCTTGATCAGATGATCGACGGCACAGCCATCGCACTG
AAGTATGACCACACCGCTGTAGTAGGCCTCCTCGCTGATGGTCTTCGTGTTGCGCGTATTGCAC
AGCTTGCACAGGTAGACGATCTCCATGCGGCGCTGCATGCGACGGAACCGTTTCAGGGTTGTCG
GCGACAATGAACCCTTTGGCTTAGCCCCAGCGGCATTGCCGGTTATCTCAAACGGCTTGGCCGT
AAAACGGCAGGCGTCGAGTATGGAGCGCGGTATCGAGAGCGCCGTTGAGCGTTGGGCATCCTTC
AGCGATTTGTCCGCATCGGCATCGGACAACGGCACTGGCGCTGGCTGTTGTGTGGCATTGAGGA
TCGCGTTCTGTCCATTCTGGCGTAGATAGTTGAGGGTACGCGATGTAAAAATATTGCGCAATGC
ATTCAT~~TTTACATCGGCATATTAACGATTATAATGCATACGGATGTCGATATGGAGTCGGCGG~~
~~GGTTTATTCTACACCAAAATATTGCCGAACTGCACCTGTGAATTATCTGTTTGTTACTAAACTG~~
~~TTTTTAGCATAAACAAACGGCGCAATTTTCTCCAGCGAGAGCGACCACGAAGGCAACACTATC~~A
AAAGCCGACTTGCGTATCAAGTGATGGCACTATCGATAGTATCGATAATTAAGTATCGCATACA
GTGGTAAAGATTGTGCAAATGTCTGCAATTGCATTTGGGTGGGCAGCCAACAAATGCGCATTCC
AGAATAAATGGGGTTGTAAAATACAATGGAATACATCAGTCTAGACGAAGCACTCTTTGCTCGG
TTTAGTTAAGACATTAAGTTAACTCTTAGGTTAATTCTTACACTTGTTAGATTATCAATATGAT
TTTCTTAAAAACGCCTACGGGTGATAAATGGATTGGAAAGTGTCAAAGCCAGCGAATACCAGCT
TTTCCTAAGAAAGGCTTCAATATTTTCAAACTGTACGCCTATCGAGTGGGTAAACACAAATAAC
GGCCATTCGATTTTGCATTGAGTGTTGACAACGATAACAAAGTGAGGTAGCATATGTGCCTTGC
TACTCGATAGTTTGTGAAATGTGCTTATCGATAGTCTTTCGA~~TTGCCGAACCGGCAACAAGTGT~~
~~AACTGACAATAAACAGAATA~~ATGTACAATTTACGTGTACTGCGTCTACTGCGCCACAGTTTGAT
CACAAAGAGCACTGCTGGGACAGCAATGACACAGTCACCGCCATACCGTCTGCTGGCCAGTCAA
CACAAGCGAAGGCCCCAACTGGGTGCAGTGCCTTCGGATCAGCTGGAACAGCTGAAGGCTGACT
ATTTTGAACGGAGCAGCGAGCTGAGCAACGACGAATGGGATCGGGTGCGTAGCACACTGACCGA
CAGCTATAAATACATCAATGACAGCAACGTGGACGCCGTCGTACTGGGCATGTGCAGCAGTGCA
GAGAAGCTGCCGTTGGCCAAAAGCTATCTGAGCTATCTGCAGGCGCAGGGCATTAAGCCCAATG
CAGCGACATTGGGTCGTATGCTGCGCGTTTATAACGCCGCCTACCACACACGCACGCTCAGCGA
CGAGGAGCAAGCGGAAATTGTACACATCTGTGACACGCTGCAGGGCGCCCACGAGATTTTGGAT
GCCAGCAGCTGTGAGCATTTGATACACGGCCTGGTAGCAACCGAGCAGCACTGGCAGCGCGCTG
TGCCACTGCTGGAGATGATGAAGGTGACCAGTGCACCCAGTGTAACAGCCTACAGTACGCTGGC
AGCCAAGGCATTTGCCGCGGAACAGCCAGAGCTGGCCTGGCGGCTGCTCGAGGAGATGCTGCAG
GCGCGCAAGCTGCCCAAGTGTGAGGTGTATCTGGCGCACTTGGCGCAAAGTGCACAGCAGGTGC
AGACCCTAGGTGCACAGCTGGAGCGACTGCTGTGCTTTCTGGAACGTCACGACATTGTCATCAG
CGATTTGGTCGCGCAGCAGTTGTTGGCGCTGGCGCAGCGGCTGCCGCAGCAATTGCAGGCGACG
ACCACGCGACTGGATCGCATGGGCAAATGTGGCGCCTGCCAGCAGCACTTGCAGCATGTGGCCA
TCAGCGATGCACAGTTCGCCGAACTGCGCGAATCCTTTCTGGCCAAGGTGCTCATACGCAACGA
CGTGTTCCAAAAGTCTACGCCGCAGGAGGTGGCACGCTTCAAGCAGTATGTGGAGCAAACTGCG
CCCTACGATTGCGTCATCGATGGCCTCAATGTGGCCTACTCCACGGGCAACAAGAAGCCGCCCC
AGCAGCTGGCCAAACTGCTGGCCACCGTGGTGCGTTACTTCAAGGAGCGACGCAAGCGCGTGCT
TGTCCTGGGTCGCCAGCACATGCGCAACTGGTCCAGGCCGGCCATGCAGTATATACAAAACAAT
GCCAGTGTTTTCCTCACCAACAATCT

**Proximal region**

>Dvir_scaffold_12970: 6,665,976.. 6,718,455 (*GJ18832*[-](*Crag*) – *GJ19325*[+](*Asta-R1*))

~~ATATCGATATGTAGGCGGCGACATTCGCCCACTTGCACTTTGTCTTGACTTTGTGCCCTTAACT~~
~~ATCGCGCGTCTCTTGACCAATTTGTTACGTCGCAGCCGTTTAATGCATGCGAAATCGTAATCAA~~
~~TTGAAAGCTAGAGTTGTTGATATATCGCACATCGTTTACATGCAATTTCGATATTACAGTATAT~~
~~GTATCGATTATCAATATTAATGT~~TATTTTTTAATAGTGGGACTCCTGAAACTAATTTTGATTTG
ATTTAGATTTCGGTGATCAGCTGTTCACAGCTGCTCCATATCAAGTGTGTACTGTGCCCAGAAA
AAGTAACTAAATTTGAGTCAAGCTAAGCGATTTAACGAAGTCAGTTTCAGAATTCATATCATTG
TGCTTTATTGACGGTTCTTCATAATTATTTGCTTTAGTAAGCCCAGCTAGAGAGCATCCAAATG
ATTCGGAGGTGGATGGATGATTATTCCACTTAATTCTTTCATTACGTATTTTGGTAAATTGCAA
CAATTAAAAGTTGCTCATATCCCGAATGCCGCGTGCCGTGCTGCAACAACGAATTGCCGATAGT
TTTGCACTATCGCCATCGCCTCGAGAAGAGCGAACATGGGCTGCTACTTATCGATGACGATTCA
TTGTAACTATCGAATTTGTTATTTACCGCGAACAGTTTTGTATTCAAAACTATAAGAATAATAT
ATTGTATTGCATGTTAACACAAAACAAAGATCGGCCGATCGGAAGGTGAGAGATAAAGGTTTCC
CACCCGCGTTTTTCATGATTTTTACCATTAAGCATGCACTTCCATCATATTAAGTAACTTCAGT
AAAATCAGTTTTAGTTTCCTACCACTTACCATTTGATCATCAATTAGTCAGCTAGACAAACAGT
TTTATATATTGAGGATTATATAAATATTTTTGAACCATTTATATCCATGTATGCTATGTACATG
TTTATTAATAATTTATAAACATTTTTATCATTTTGAAAAAAGAAATTATTTATAATATTTTGTA
CTGTATCTAATAATATCATTTATATACAAAACAAGGGCATAGATGCACACGCTTGCAATTGCAT
TATTTATACAGTTTTCAAATATATAACGGCCTGCGTTTAGTGGAAAATTCGATTAATTTGAATA
AATCGTCATCGATAGTTAGCTCATCTCGAAGAGTTGACGATAATTGAAAACTATGAGTTATCGG
TGCGGCAAGACATCGGCAATCGTGCTGCCAATGTGTTATTGGGGTCTACTAGTTGCGTATACTT
AATAAACTACAACATAAAATCAAGGTCCATCAACGCAAACGGAATCCTGGTGGATAAGACACCA
TCAACTCATGTGGACGTTATTTAGCTGGGGTAAATAAAGCTAAGATTAACCTCAACTGCTTAAT
TCTTTTGAAATATGCATCGCTTTGCGGCTGGATACGAAATCCCGTTAAAAGCTTAGAAAAATAG
ACACCAACCCGACTCATTTGGACTTTCTACTTTATCTAGGATAAAATCAACACAAATAATAACA
AATGTACAATTACCGTTATACCGTTGGGCGGCACAACTATTGATATACAAGTTATCGTAATCGG
TTGAGCGCCAGCAATTCCGCATTTTAAATTCGCTAGCATCTTTTTCTATTCTATAACACTTAAC
CCTTTTCTACTGCTACTACTACTACTATTTGATTACAGTATTAAGAGACAAATCGACTTCTTTT
AAACGCGACCCAATTAGTGCCTTGCAATTGTATTCACAGTTCTTTGCTCTGTGACCAAGCCAAG
CGGGGCGTAACCGGCCCCAACAATGGCGACGCATGCATTAGACCTAATTATGCTTTTTATGGAT
TTAAGCCAAGCTGCTGCTGTCTATTATTCCCAATTTACAATTGGCCCAGTTGGTGGCGGCACTT
GTCCATTGAGCCAAGCCGCTCGGTTTCATTCATAAAGGCAGTTGGCTCGAGTTTTAACTCAAGA
TGAGCGCATTGTGGACTGCGACTCGCGACTCAAATTGTGCGCAGCCAGTGGCGCCTTAAAGGGG
AGTGTTGCCTGTTGCCTGCTGCTTGCCCCCCCTGCGACAAGGACTCTGCTGCACAGTTTGACTC
GGGTTTGTTTACAAATATTCCATCGTGCTTGCCGCTCGTACCGTAGTCCTTTGAGATTTGTATG
CGCGCCTGTTGCGTGCGCCCGTTGGACATTCTCCTAATGGATTCTGCTGCGGCCTCGATTGCTC
TGGTTGCCCCGATAGCCTGGCTGCTATCCGTTTCTGTTTGTGATAATCTAATTATGCGGTCTTC
CCGAGCTGGTTATCGGCTGTGCCATTGCCATTTTATTTGCTTCTAATCAACTGGACAGCCCGTT
GTCCTTCGTCCGTCCTCCGGCTGGCCATTACAAAATAATAATATAACAAAGATTTATTTACAAA
TGCCATAATAAAAATCGCAGCCAAACAATTAACTAAGCAATTTAGGGCAACAAATCAGATTAAT
TAAGAACAATAGCCAACATTCAAATGCCAAATGGCATTGGACGTTTGGTATTTGGGACACTTGA
CAAAAATGGTCTTTGAAATTGTATTTCTCATGTCTCATATACGAAATTTCTGTCGACTCAGGAA
TACTCTGTAATCAGTCTGCTTTCAAAAATTATATACAATATTGGGGACGGGAACGGAACTATTG
CTAGATTTTTCAATGTTTCTAATACTTCTTCGTTTAAATGTTTATAAAATTTGTCATATAAATG
CGTTGAGAAGGAAAAAATAATAGCCCCTTTTTTTTATTAATGATTTTTAGTTCATTTACTTAAG
CCACAGATTGAGCAAGCTTCTAAAAATATATATAGGGTGCATGTTTGAATATGAGCTCGTTTAA
GGAATTGGAATTATACAACTATAAAACTTACCAAAAGACACTATATAATCTATATTAGAGTCTC
TTACTAAAGGACGTTGTGTTAACAAAATAAATAACTATAAGATCATTTTGTTTATATATTAGGC
TATAACATTTAATCTTTAATTGTTAAGTGGAAAAAGTGCTAAAATAGATGAAAGTGAAAAAAGA
AACACTTATGCGATATATTCTTTGCAAAAGGGTGAAAGCAACTTGTTACAGGTTTTTCATAATA
CATACATTATTGTATTTCAGAGAACAATTTTCAATGCATTTTTTTTTCTTTCGCATAATGCTTA
ATCTTAATTCATTTGGTTTGCTTTTCTTGTTCCATGTCTCAGGCGACAATGTCACTCAGGATAT

TTGAGTGGGCGGCCGCAGCGAAAGCGCTCAGAGTAAAATGGAAAAATAACTCATACGCCGTGTC
GGCCGTCGTTGGATGCGTGTTCGGGTTTCAGTTCGTCGATTCTTTGACTCTGACGATACTGCTA
CGGGGCGCTAGGATTGTGATTTGAATTGTAGAAAGCGCGCAGTTCGCATGCGAATGTCCTGACC
GACGGTTGTGTGGCCGGCCCAGCATCCGAGCAACCGCATAGTTTAAAAAAAATCTTCGAAAATT
GTTTTTGAAAATGTAAATTGGCAGAGTAGCTACGAGTACTGAAAATAATAATATATAATATTCC
TAAATACATACTTGTATCTGTGAAAGTTTTTCTTAATTTGTTTTTTTTTTTGGTGCTGTAAAAA
ACCATCAACAAAAAAACGGCGACAAAATCCAAAGTGAAGAAATTACAAGAAATTGCAATAAAGGT
TATTAAATTTTATAAAAGAGTCTTGAGCGTGGCAAATTTCTTGCGTCTAGTAGCTGTCACATCA
GCTGCTGATTTTCGGCTCCGCGTATATATTCAAATCTAAGCAGGTGAGTGCACCGCATAATTAC
CGGAAATTTAATTATGAGGTGAGGCGTCGACTTTGTAAACGGTAATTGCATTACCTAGCCAACC
GCGCTCCCGCTTCGCAGCCCGAAAATAAGCAAGTAATAAAAAATGTTGCAGTCAGAAAACTCCC
TTACTTCCAAACACAGGGTCTGGCTTAAAATAAACACCTCAATGCCCCATAAGAAAGTTTTTTT
TTCGAGTTTGAGAAGTGTCTATGCACAAAAATAATATTAACTTGTTTTTACTGTGAATGAATAC
CTTTCGAGTTCTCAAATTACATTCATACGTCGTTAGTTCGATTCAAATTAAATTTTGCGGTTAC
CCCAATCAAAGGCGATTTGTAAGCAACTCAAAAGGTTGAGGAATGTTTCATTTCTTTTTTTTTT
TTTTGTAAACCTTATTCAATTAGGTGCCCACCAATTTGTACCGCTTACATTTTACTTAGGGGCA
AGGGAGCTGCCGGACCTCTTTCAGGCCGAAGAGCTTCCCGTCGTGGGTGTAGACAATATCAAAG
ACAGGAGCCGAGCTATTTAAATTCGCAGACGGAATTAAGCTCATTAGCATATTGTCGCCGGTCG
GTCATCAAGTTCACGGGCAGATGTTACAATTTCCCATTTTCCAGTTGCCAAGTTTTCCACTTGA
TTGATGAGTCCAGTTACAACCCAAAACTGCCACCTCATACTCGGCGCCACGTCTACAAATTTAC
CTGCTCCCGCTGCAGAGCTAGAGAAATGATACGCATTAAACAAAAAGATGAGTTTGCCGTTTGT
CCAGCGACAATCAACATCCGCTCAAATCGCATGTACTATATTTCCTTTGTTCTTTTTACTTCGG
GTTTCCGAGCATTTTAAAACAAAACATTTGTGACTAAACACAAATTCGTATTGTATTTTTTTTA
AGTTGCCTTCGAAAACCAGGCTACAATACGTTTTCGAACCGAACTAGTTCGGAACGGAAATTCC
AATTTATTTTTACCTGCGTTCCGTACCTTAAACCAAGTCAGTTGCCCCAAAAACTGCTGGAACG
CATCATAGCTCTTTTTGTGAGACGGACTTAGCGCAAAAGCATAACAAATTTGGGCCCATAATAC
TATTCAACTATATTTTGTAGATGTCACACTTGGGTAGAGATAAAATTTAGCCAGCACTTCAACA
TGAAATAAAATTAAATAAAAAGTTTTATTGTCTGATTCTAAGCTAAGGCTTAAGTCAGAACCAA
GGTAATGAACCAAAGCTTAAGGCTGGGAGTTGGTTCGCACCATAAACCGAACCCGTTTTTTTTT
TTTTTTGGGCTGCAGACCTGTTAAATAATTGGAAACTGAATCAAACTGCTTGCTCCAACTAACT
GAGTGACAAATTAATCAAGAGTTGGTAACTACTATTTAGTTATTGCAATATGCTACAAATGTAT
GATGCGGTTTTCGTGATGAATATAAAAAAAGTCTACGATTTGCTGATAACTTACGGTCCACTTT
AACGTCTCGCCAAAAACCTATTTTGTTTTCAACTATTTCTTTAAATTGTTGAGCATATGTATTT
CAATCTTACGACTGATATCGCATCTAGTTCAAAAGTCACCGATTTTTGCCAGCAATTTCAGCCA
ATCGAACCACTGTGCATAGCTGGTCAGTTTCTTTGTGGCACACTCTTTACCTCATGTGGTGTTA
AGAAGGATTTTTGATATGGTCCAACCGCAAGTGCGGTAAATGATGGAAAAAGGTTCCAGCATCG
GGCTTGAACTCATTTTCATTGGGGATCAAAGTAGTAAATTAAAGTTTTTCCTCGGGCGGAAAAT
TGATTGCAATGAGATGGAAACTGGCAAATACTCGCTAGCTTAATAAACTTTGTATTATTATAAT
TTATGATTTTACTGCTTAAAATGATGAAGTAATTTTCGTGAAATGCTTGAAAATCAACCTGAGT
TCGGCTTGATAATACAGGCAAGCTTGCGAATTTCAAAACTAGACCACCAAAGTAGTTATCCAAG
GTTCCGTTTCAAAGCAAGCTAAGGAAATTTCAGAGAATATAAACTCAAGTTAAACTGATTCTGG
GAAAAAACTTTAAGCATTCTCCCTTTCTTATTCGCTTAACATACAACAAAGTAAAATATTTCAG
TAGTTATGATAAAATGTGAGTAAGCGACTTGTTTCTACACATTTTGGAATTGCAATCTTTGAAA
CTAGGGTGAACCAACCTTGAATATTTGTTGAGTAAATTTCTCAATTTCGCCTTTGACACGCGAT
TTTGTTTCATATAATGAAGAAAAGCTGAGAAGCATGCGACCTGCATTTAATTTCAATTCCTACT
TAATTTGCATTTAATGTGGGGACTTTTCGTTGAAATTTAATTGGCCTAATTAGGTGCGACATGG
CCGTAAATGTAACGTGAACATTCACGCATTAAGTGATTAAATGAAGACTGTGACCCATGGAGCG
AAGCAACTGAAACGAACTGAAGTGAAGTGAAACAAATGCGAGATATGAGAATTGCGCATTGAAT
TAATGAGGCAAATGTCAAAATTGAATGGGTTAAAAAATGTCTTACAAGCGCGACAACAAAGAGA
AAGTTGCTGCAAGGGAGCCGGGGGTGTCCTGACGAAAGATGCTCGCACTGGTTAAATGATTGTG
CTAGCCGCAGTCTTTAAAGTTCGTCCGAATGCTATGAAAGAATGAAATTTTTATAAACTTTTCC
ATAATGTTTTAGCAAGAATTTAACAACGCTTTAGGCAAGGCAAAGCCAGCATACTCCCATCATA
AAAAAAAAAAAAAAACAAAAACACAACCAACCTTCTACACTAAGGCAAACAGACCAAGATAAGG
CATTAAAAGCGAACAGCATAGGCTAGTACGACCTGCTAATGCCCTATAGCTTGGGATGCCGAAA
TATCACTTATGGCTATGGGCGTGACTCAAGTGTCATGCCGTTTCATAGCTAATACACCTGACTT
TGGCAGCCATATAGGGTATGCGTATGAAAAAGCATCGCAAAGCGTTGACTTCTCTTGAGTCTTG

CATCACTTTGGAAATAGGAACGGAACCGTAAAAATGCCATAACGCACAGTCAAAGCCAGCTGGC
GCATGGGCACAGGTGGGCGTGGCACGAGGTTGACCGAAAAAAAAACACACACACACACACTCGG
CTCATCTGGTGGCTGCCGAGCAAGCCACAACCCGTTCCAACCTATTGTCTATCTCTTCCGCCAC
GGCCTTCCTGTAAGCCCAGGCAGTCACAATGTTTTGCTTATTGTCCTGCTGCTAGTTGGCATGG
CAAGTGGTGTCCTCCCAGCAATTGCAGTTAGATAAGCGTAGCCATGCCCTCCACCCTACCCCAC
CCCCCTAGTCGACACCCTGCGAACCCTTTGTGGGATTTTTGCTCTCAGCTGGGCTAAGCAGCTC
AGCCAAGAAGATGCTGCATACATTGTGGCGCCAGTTAAAACAAATTTATATCTACGATATGCAT
CTCAAATGTACGAGCACAAAGAATTCCTGCGATTGCACTGAAACTTTGCACTGACATGTGGCTT
GGTCAAGAATCTGCAGCATGGCAATTTTAGGAGGAGAAAAAAGGTATCTCAATTTTGGTGCATA
ATAAGCTACATGTTTTATGTCCTCGAAATAGTTCAGACTTCGTGCAAAAGTTTTCTTTTGCCTT
CACTTTGAGCTGCGATGGTAAAAATTGTACGAAAATTTAAAACTTATATATGTACAGGTTTTTT
TTAAGGTCAACGAGTTGGTAAGCATTTTAGTCAATAATCAGTTTTTTAATTATATTTATTGACC
AGCTTGCAATAAATTTTAGTTGACTAAAGACTATAAAGAGAATGCTTAATGTGATATCAAATAA
AATATAGTTGAATTCGAATTTTCTTTAAATACCTAAATATCATCAATTGTCTAAAATTTGATCA
CGCAAGATATCTTACGTAACATATAATGAGAAGCATAACGAGAAACTAAACAAAATTAGTCAAT
TTCTATAGACACAGATGAAAATTTTTAAAGTCACCTAATGTCGCGTAACGTGTTACTATTTGCT
TACAAATATCTTGAAGCCAAAGCTAAAAACAACACAAGACATACGATTACAGCAGAAAATACTC
AGTTATATTATCGAAACCCAGCTAAAGTGTTTTCTACTTCTATGAGTAAAACAAAAATGTTTTT
TTTTTTTGCTGGCAAACAGAATCCAAACTTCAACAGAAAAAAAATATAATTTAAGAGTATTTAC
GGGCGTTCGATCCCTAAACTAGCGACTAGGTCATGTCAATGAAAAAAAGCTATAAAACTATATT
GATTTGTTGATTTGTTTCTATATAGTAGAAATAACAATATGTTCATAAACATAATATTTATTAT
ATTAATAAATTAAAATTTTAAATTTCAAGCATAAGTGTTTTGTTTCTTTTTTTTAACGTCAACA
AAGTTAAGCAAAAAATAAGTGTTTAACACAGTTGGGTTAAAACTGACTGATGTCCTATCCAGCA
ATACATCGATCTATAATGCTCACAAGAGATGCCATAAAAAATGAAGTTTTTTGTTTCTTATACA
AATTGTTCTTTGCTGACTGCTCACTGCTAACCGACTTCCGAATACTCTGCTTACAATGTAATAG
GAGCTCAAGACAGCTCAAATGGAGTCTGTTTAGTTGTGACCATAGAAACAGTTAAGGCGTGGCA
AATAATATTATAAATACTATTATATTTATCATTATTATTTATTAATTAAACGTTTGAGATATAC
TTATCTACAATTAATCTTTAACAAGCACTGGTCACAATAGCTTTCGTCTATATAAATTTCAAAT
ATTTAGCTGCATTGTTCAAAACAAATATGCTTATTTCTCTTTGTAACCACAGTATAAATATATG
TATAAAATGAAATGAAAAAAAAACACAAGGTAAATACGTTTAAAATTTGTATGCACCCTCGTAG
CAACACACACCCACACACGCACACACACTCCTAGTCAAAGTTTATGCATATCGTGAAAAATTTA
TAAAATTTTGCCAGCGGGCGGTTTCCAGGCCTGCTGTGACTCCACTGTATGTGTGTGTGTGTGT
GTGTGTGTTTAAATATAATTGATTGACATTATTTAAGCGTAAAGACAAAAAATTTGCTAGTAAA
TAAATGATATGTAAATTGTTTTTGCACACACAGCGTGCCTGGCATGTGTTCCCAAAAACAATAA
CTCCAAATTGTTTTTGCCTGGACGTGGGCTCAGTTTCGATATTTGATATACCGAATTGTGACTT
TAATTGAAATTCGCAGCGGCCTTTAACAAATATATCATTCAAATGCATTTATGAAATATTCGCC
AAATATACATTTTATACAAATTTATATTTCAGGAGCCGGCTCAAATAAATTCTAGCTGCATGCA
TATTACCTTAAAATGAAAGTATTTTATTTTTTTGATGACTTTAAATAAAGCCAGAACTGCTCAA
ATAGTCGAACCAAATAGTTTTGAGCTATATAAGAGAGATTCTCGCACCCGCGAAATTGTTGTGA
GTCGGCCGCTGGGACTGACATACGTTTATGTTGATGTTCATTTCAATTGTTCAAGTGCTTATTG
CTGTTGTTATGGTTGTTTTTTTTTTTATGAGTAAATTAAATGTGAATTGCCCTTTGCCAGCCTA
CAGGATGCCTCGGCTCTTGCTTGCCTCACAATTAGTTAATTGACTTAAAATTGTTCATTTCGTT
GTTGTTGCCGTTGGTGTTGCTGCTGCTTGTCGAGATGAAAGTAAAGTTCCCTTCCTCAGAAGCA
ACACGCACAAACAGATAAACATGCCAAGAGACATCTCGAGAGAGAGAGAGTGAGGGAGAGAATA
GTGAGAGAGACAGACACAGAGAGTGTATGAGAAACAAATGTGGTGCAAAGTCACTTTGAAGTTC
CCCGCACCCGCTCCAACCGCAAGACGTTCTGAGGTATGGCGCAGGGGCAGTGGGTGACGAGTTG
GGTACTGAGGGCACGTCGGGGTTTGCCTGCCTGTGGCAATTGCTGCTGCATGCTAATCGAGTCA
AAATGTGCCCCAGGAGCTGCTTCCGTCTACATAACAAGATGTGCGGTTGTTCTTTTTGTCGTGG
CTTTTGTGAATAACTTACGAACAAATTAAACAGTTTTTGGTTTTAATCTCACACACATACACAT
ACGAACACGCACTGAAATTGCCTGTGTTAATTATTTGCTGTGCTGTAAAAAATGGCTCTGAACT
AGTTAATTGAGGCGCAAATGTAAGAAAATTTTGCTGCTGCTCCGAGGCTAACTCACTTTGGCCC
TTGGCCATTTGTAAAATGTCAAAAATGAAAACTGTTTGTGGCTAAACAAGCACACACATACCAG
AACACCCATTCACACGCCCACACACAACCACACACACACACACACACGAGGAAAGACAGCGCTA
AGCAGAGTTATGAGCAAAATTGTATTGCCTGCGACTCGGAGGACAGTCAAAAGTCCGCTCAGGC
ATGTTCCAAATTTGTCTTGGCTATGAGGGCGACACAAAGCACATTGTTGGCCGTAAAGGGGGCA
AGTGGTAGCAAGGAGCGGGGGGCTGGGTGTGTGGCTGCAAATAACAAGGGAGGTCGGCCATAGC

TTGTTGCTTTCGACTGGGGCCCATCAAGGCCAAAGCGCTTTGTCCACGAAAATAGTTTGCGCTG
CATTTTGGCCTCACGAAATTGCGCACATTCATTTCCCTTCCAGGAACAGGGAGACAGTCAAATG
AACAGCGCTTCTCCAGCATAGCCCCTGCTTCGTAAGACCCACTGGGCCTGGACTTAGGAGAATA
GGTTAATTGCGAATTTATCAGCTGTAGGGAATTTTTTTTGGCCTGGTTTTAAGTCCACAATATA
TCTTCATAGCCAGCCACTTATTCTTTCCAAAATTAAACGGCGCTTGCATGTCTTTTCACATGTA
TATATATATATATATATATATATATATATATATATATATATACATATATTTACTCTCGAAGGCT
TGGACAGTAAATTGTAAATTTGGGACAAGCAGTATTACTTTCACTTTTACAACTGGTCGGGAGA
ATATTAAATTATGCAGTTGTTCTACCCCTTTCTAAATTTTAAAGTGAGTTTTACGTCAATCGAT
TAAAAGGGGAAATTGACAATGAACCGTTTCCGCCCGTATTATGATAAAGAATATTGCAAAATGA
GTTGAAAACCGCTCGGGCGATAATTAAAACTTCGTCCATGTAAGCTTTGTCCCATGTAACCTTT
GTCCTTAGTTTTTCACCCAAGGATGGTGTCCTTTCCGAAAAGTACAAAGAATGATGTTTCGAAT
TTAAAAAAAAATCTCTGAAAATGAGCTCAAATCGTATCGAATAAAATTGAATGAGCTCCCACAA
TTGAGTGAACAAGGTAAACAATTACTGAAAAAATGAACTGGATCGTAGGATACATACAAAGGTT
TCGACTATATAAAATAAAATATATGTAAAAAAAAGAAAAAGCAGTAGTAATTGAATGCAACTAG
ATCTTCTTTATCTTAATTTTTATAATTTATTGATGCCAATTGAATTATTAAATGAACTAGTTCA
GTGAGCTCTCTATACCAACATATATCAAAGATATTGAGAAACGTTGGTGCGTTTTCTATTAAAC
TGCAGCACTTTTCCTTTGATTTCTTAGGATCTGGATCATACCAGCGATTGTGAATCAGTTGAGT
TTGCAAAAAACTAGTTCAGCTAGTATTCTTCAGATACTTAATTAGTGATGGACGTTTCAATCTT
TGTCTTCTGTCTTTCAACTCTCGTTTAGCCTTAAAAAAAAAACAGGTTCGCAAACTAGTTTAAA
ACTTAGATCAAGTTGTGCTTGGCATGGTCCCAGCCCGTATAATTCATTCTCCCAGCAGTCGAGC
AACAATTGTCATAGATTCGCCATGATCAACTCATCACGTCTCACACGATTAAGCCTAATTCAAG
CTTAATATCAATGTGCGACTCGCTTGCTTATTAAAATTTATGATTTCCTTCTCTGCGCCCGTGT
GTGGGTGCCTTTGTTTGGGCTTGGGGCGGAGCGTTGTATGATGTGTTGGAAAAGTTTTACAACT
TTTACCAAATTGTAAAGTACGCCCCTCAAGTACTAAAACTTTTACGCCTCCAAGAAGTATGCAA
TGTGCATGTTACAGTCGTCAATTGTACTCATGATCTGCATGTGTGCGTGTGCGTGTGCGTGTGC
GTTTGCGTGTGTCTGTGTGTGCTTCGAGTCAAATGTCACAAAGGAGCAAGCTGCTCAAGCAACG
GGAGCAGCATAAAAAATTCAACAAAAGAAATGTGAAAATATGTCAGCGGGTTAAGCAGCAGCTG
AAAAACAACGCGCTGGCGCATGCCGTTTGCCCATGGAGCATGGGGCGTGGTCGTGGCATTAAAT
TATGAACTCTACAGTGCATGTGCTATGTAGCAGACGAGGGAGTCCAAGGGCAAGGCAGGGCGTT
CAGAGTTTAGGGGCGTGGACAGACACACAGCACACATACTGGCAGTGCGACAGGCAACGAAACG
TGAAAGTCACAAACTGCCAAATCTCTTCTACAATCGCAACAATTTCGCCAACTATTCGCCAACT
ATGTAACTCAGCTTAAACTCAACAAAAGCTGGGTAAATAAATTTCGCGATTCAATGTTGTCGTT
TGTGTTCTTAAAGCAAACTTTAGTTTTTGGACTATTTTGGAAACACTTTTGCGTGTTGAAGCGC
TGTTGCATGTTCATGTTAAGTGTAGAACATAACTTGTCCTTAAATGCTGAGTAGTTCCATACAC
CCACACATACATACATACACCCACACATACATACACGCATACACGGTTACCCTGAGGGAACAGC
CAAAGGCTAATTTGATTTTATTAATGAAATCATTAGTAGAAATGACAAAACGTAGCCCCGAAGG
TAAGGTTGAAGTCAGGACACGGCTGCAAGCCCGGTCAGCAGTCGACCAGACGAAATGAATTTAA
TGTAGCATGTGAGTGGTCGTAACAGCTATTCCCTTTTTATACCCTGTACTGTACTGGGGTATCA
TACAATTTTTGATATGGAAACATAACATTATTTTAAACTGGATTAATAACTGCTGTGATTTAAT
AATATAAAAATAATATATTAAAATACTATAAATTCAACTTCTGTAAAAATGTAATAATAAGTTA
GATAGTTAGTGATTTTTTTGATATTCGGCTATTCGAAATATATATTGATTCTTATTATTATAAT
GGTTATTATTAATAAATGTAAAGGATATCAAGCTTACATTTCAAACATTAAACTGTCTACTGAA
GGCTTGTGGTAAGACAAATACCACATTTAAACAATAGCCCGCATCCACGTTGTTCATTTGACGA
AAATTCTGGGTCACTTAAACAATTCATTTACCGTGGAATGCATTGTCAATGTGACTAAATGCTT
GTATAAACGTCAATCCTCTAATACAAAGCATCCCGGATTAATTGCCATTAAATTTCATTGATAT
TTATCGAAAAAGGGAAGTAATTCCGGGAAGAGCACCATTGTCTGGCCTATTCTTCGTTCTATCT
ATGCAGAGGGTATTTTAGTTTTGTGACAAAAAGTGTTATGCATTGAGCAAAAAATATTCTAATT
AAAGTATATATATATATATATATTTTATTGATCAGTCTATAAACTGGTTGACAAACTTCAAACTTT
TTTTCCTCAAGGAACATTTATTATGTATGCACAATTGAGTTGAATCGGAATATTAAATATCTGT
ACATCATATGACAACCAAAAGTTATATCGGTCGAGAGTTGAGTTATTCATTTACAAATCATATT
TGCTTTTCAAGATACCGAAAAAGTCATGCTTCGGATATATTTCTGCCAGGGTATATCAACTTCG
AAGCTCCGAAGAGAACTGTACTTCCATCTTTTTTTTTTTTGTTTGTGGCCATTGTCATTGCTTT
GTCCTGTAGCAGGAGTCCTGGTGCGACTCTTTGTCATGTCAGTGGCTCATTTCATGCGTACCCT
TCGTGGGCAAACTCCCACCCACCACCCACCACCCACCAGCCACCAGCCACGGAGCGGGGTATGC
CGCTGAAAGCTGAATGGTAAATACGTAGTGCGATATAACGGTAACGGATGTGGTCATTCACACA
AATTAAATATTTGCTTTTCGCTATTGTAAGCGGAACGTCATGGTCCGATGTGGGAAATTTTATG

CATTTCCCAACGCCATGTTAAATTAATTGAGGCAAATTTTCATGGTAGACCAGAAAACATGAGC
TTCTGGCACTCGGGTAAAACAGCTGTAAATACAAAATACGAAATACCAATTTACGCATTTCATT
TGCGCGCTTTGTTCATTTTTTGGATCGTCACAAGGTTTCGGCGTTCAACAAAGTTGAACAACAG
AAGCTTTAAAACTGGCAACTGTTGAACACTTTAATGGCACTTTAATAGTAGTTGAGTCTTTTAA
ACTGATTGCAATGCTGAAGAACAGTAATCTTGTGCTTGAATTAACTCGGTCTAAATTCCTTTAC
AACTTTTTTGATTGCTCTATGGCATTAAAAGTACTTTGAGCTACGGGATAAGTGCTATAACAAA
CTCGTTTAGTACAGTCCAACTCTTTCCACAAAAACATACTTTAAAAGCAATGCAAAAGTAAATT
GGAAATAATCAACTATACTACTAAATATAGTTAATAGAATCTGATTCAATTATATAAAAAATGA
ACAAGCAAATTTGATTTATAATCAATTTAATTGTTCTACTAAAACTTGTACTTAGTTGGTTTCA
CGTACCGAACGCTTCGGTATTACTTGCTTGTTAAAATTGATTGAATTGAGCCGAGCAACGACGG
GTTCTTCACTGAAATAAAAGAGAGTCCTTGGAACTGTTCAAAAATATTACCATAGTATCATAAC
ATCGTGTATCTGAACCTTTCTTCTTCTCTTCACATATTTGTATATATATTTATTGAGATTCTTT
TTTACACTTTCCACGTTTGTTACGCTTTCACAGATGATTTTTAACAAGAACCAATGTTTGACTT
GCAATAGTTCTCTCTTCGTGCATCAGCAAGGCTTTTGTTCCTTCGATTTAACTTTGGCTTAAGC
CAAGTTCCTTTGAATAGCATGCCTAACAATAAGCCACTTGCATTCGCCTGGATCTATTCAAATG
GATTTCTTAGAAGTGCCGCCAGACTAGAGACTATTTCCTTTTTCGCATTATTATTTGCATCTTG
GTATGTTGGCCATTGTCAGATGGACAGCCTTAATAATCATCTCACACACAGCAACAATCAAAGT
TCCGCCCATTGAGATGCTAATGTGGCCGTGTGCGTGCCTCTAGGCCCAACACTTGCCGTTATGT
CTACCCTACACCTACTTACATATGCATATACATATTATGGCTACTCAGCTTGCCAATTTGTATT
TCCTTGTAGAGGGTGTTATAATGTCACAAAGTGTGTAACGCACAGAAGAAGAGGTATCCGACCC
CATACACTATATGTACTATATACATATTCTTGATAATGTAGTCGTATCCGTTTGTTTGCTTCTA
TGTTGGCTTGGTTTTTTATAGATCCGTCTTTCTGTTACAGGATGTGTCGGGTCGGAGCACGATG
CCATTTAGGTGTCATATAAATGATTGCCCATGAAAGAAGTTGTTCTTCGACAACCTTTAGTTTA
TTTAAGATATCTTACCCAGAATTGGAATTCACGTGCTTCATTGTAATCTTATCAGAATCGATTA
TATCTTTGGGCAGATTCGGTTGGTTCTGATTCTGGTTTTAAGGCTCAGCAAATTCGAGCTCTAC
TTTTAATTAAACATATTCGCCAGGGCGTATCCAAGCGGATAAGATTGCTCCATTTGAAATTTTC
CATTATTTATTTTATTTCAATCAATTTTATGAAGCTTGAGTAGCGTGGTTAAAAAGTTACAAAG
TGTTTGCTGAAGATACTTATAGACGCAAGCACATGAATTTTCCATACTTGAGGTAACCGAAAAG
AGAATCTAGAAAGTACAGTTTTTCATAGGAGGATAATATTTCATTCAATGTTTCTTAGCCAGTT
CCGTTTTACTCTCTTCACGGATATACAATGCATAATGATATGTAAAATTACCTAAAGTCTGCTC
AGCATTTTTAAATAATTTACCTACAATTGTTTTCCTTTTGTTAGTGTAGGACATTTTACAGTCA
GGCATTTCTTAAAGTCTTAGTTATTGTTTAAGATATTTCACGAAAAATGGATTATAGAGCATGT
TTGTGTAAGTGGGTTTGCCAGTGACACTGGCTCCTGGCTCAGACTGTGACTTAGCACAACGGTA
GCATGTGTACACACACACATATATACACACAGATATACACACACACATAGACACACGCACACAC
CATAGGTAAGCAAGCATATCTATGTGGACGTGCCCCATTGAAGCGCACCTAAGCACAACAAACA
AATCAAATGTGGCAAGGATGCGTTGCGTATACGCACCGAGGCGCTAAACGTGCTGGCTAAATGT
TTGAGTTGGCTAGATAAAGACTAGAACGTACCGGCAGCATCAGCGGCAACGGTAGCGATAAACG
GATCGTCCTCTTATCGGGTCTGCATTGCGCCCAGACACGCCCCGTTTGGTGTACGAGTGCCTTC
GACACGGGCACATCGCTTTCGCTAAGACAATAACCGATGCTAATGTATTATACGTGTCAATTTT
GAATCCCGTTTGACTGATATGTGTGCTCACATCACTGGGCCACACTGCCTGGCTGCGCGTGTGC
CTGTGTGGGCGTCATAATACATTGCCAGCTTGTGCCACGCCCAGAAGCCCACTGTTATGCCCAC
AGAGTCTGCCTCTCTAGTCTAGGCAGTGGGTAGTCGTGGTCTCTTGATAGAAATGTCGCTGTCA
ATCTGTGACAAATAACTTTATCATACAATGCAACTGATAAGCCAACAAGTTGTGCGGCCAGCCG
AGAGCGGCGGTAGGGGAAGCGTAGGTGCAAGGGGAAGGCTGTCGATTGATAACCGATAACACAG
CATTGTGGAAAAAGCTATCGATCATAAAAGGCTAGGCAGCAGGAATAAATGTTAAATAGATTTT
TGCGCAAAAGTTATTTACGAAAATTGTGAAGGCAAAGGCAATCTGATTAAACCTTAAGCCGCTT
AAATCTGTGCTTAATTTAAATTTTCTATTTAACTTTCGATAAATATTAGCCTATGGCTTTTTCA
TTATATCTGTAAGGTAATAAGCATACGATCTTTTTGTATTCTACATACCGACCATGTATTTCTC
ACATTTTAGACACCTTTTTCCGCTCTCCGCAATATTAAAAAGCAACTTATTTTATCCCGCCAAA
CGCTGAATATTTTCCTACATGTGTTTATACAACGAGTGCATTCAAGAATGGAGTTCCTTTAGCG
TGGACCTGTAGAAGCACACAGGTTCGAAGGCATTGCAGGGCAGCTCGAAGTAGTGCATAGGGTA
TTTGCTGGTCGAGCACTCTCCACAATGATATCTAATATGTTTTGAATTCAATTGCTATTGTTCG
AACGCATGTACTATTACCTAAATCACAGATCAAATAGTTTGGTGGAGCAGTACTGCGGTATTTG
GTTGAGCTGTCGAGCGGTTGGCTACGCTACGGCCTCAAATTCTGACTGAGAGCTGTGGTTTTTC
TATTCAATGCGAACAGAACACAGATCTGTGGCTACGGCCTCAATGCGAACTGGCATATTTCTCC
TAGATTTAATGGTAGAACAATCGTCGGCACAGCAGACTGTGGGCCAGAAATATCGATTTTTGGC

CTCTCTTCAACCAAATCAAGCAGCTAAAATATGCTTAAAATTGCATTTGCGTGAGCTCTACTTT
TTCAATAGAAGATCCCAATACAAGTTAAAATATGTAGTGATTGTTTCATATTCTAAGCTTGAAA
AGAAGATAAGTTTAAGCACTAATTTAAAAATACAAAAAAAAGTCAAGAAATTATTCAGTTATTA
GCATAAACATTTACTAATTTTTAGTGCACAGCATGTGAAGATTTTTTAGTCTTAGACTTTTGCC
GTCTTTTTTGCAGCGTATCTTGTGGTCGAGCACTTTTGACTCTAGCCCTCTGGTTTATTTGCTT
CGTGAGCATGTGTGCGAGTTTTGATGTTTTATGCATCAAATGAGTTAGCGTCGAGCCTAAATAA
GAGCTAAACACTCGTGCAAGTACACACACACCCGCCCACAGATACATTTGCAGATACAGTTGGC
GGCAGCTACGTGAGCGGGCAACATTTATGGGCCTTCAAAGGCAAATATATGCACGTATATATGT
GTATGTGCATGCTGGCTTTTACTGTGCGTGTGTTTTTTTTAAGTAAGACATTTGCGTAAACTGT
GAACATTTAAATTGAATCAAGTTCTGGCCATGCCGAAAGGCTTCCGGACTAACACTGAGAGTCG
GATGCGTTGGTAATGAAACCCTTGTGGATTGTCAGCGTTTGGCCAACTTAAGTGACATTCTTTT
CTTCTCTTTTATTTTTCTGCACAACTTTTATGATGTTGGGGCAAAGTCTTGCGACAATAATTTA
ACTTGTCTGCTTTTTTATGCGACTTTCATATGCTGCTTGCCAACTGGGATAAAACGCAAAAAGT
TTAAACAGTTTACAGTTTACAGCTGCCCTCAAGCGTCGCAGTCGCCGTCGCAGTCGCTGTCGCA
GTTGCTGTCGTCGTCGTCGTCGCCGTTGCCGTCGCCATCGATACGCTTTCAGCGCCACGTTTCC
ATTCACAAATTCGAGCTTATGGGGCTTGGGAGGGGTTGAAAGCCACAAGGCGACAGTTGGGTTA
CAAAACGTTCTGGAACTGGTTCTGTTTTCCTGTGCTACAAGCGAAAGTTTAACGCTTGGCAGCG
CAGCACATCAAGCATACGCACTGTTGCATGCCCCAAGAACTGTCTGATGGCTGTCGACTGTTGC
TGCGGCTGCTGCTGGTGCGGCTACGGGTGTTATTGCGGGAAGTACTGTGACTGGGCAGCTAACC
CTTCAGCCCCGAGCTTAGCAAAAATTAAACGAAGCAATAAATTATGCGTCTGCCAATGGGCGCC
AGTCAGGCATCAACCACAACAAAAAAAAAAACGAAAGACAAATTTTTATATATATACAAAAATTC
TGCCTTTGTGTGCGTGTGCGTGTGTGTGTGCTTAAAAATCCCATTTTGTATGCAGATTTGCCTA
ATCTTAGGGTTTTACTCTGTGTATATGTGTATATGTGTGTGTGTGCGTGTGAGCGTGCAACTGG
CAGCAGTTTAGCGACTATTATAAACCGGAAATTTTACTCAACACAGCTTCGTATTCCGGTCTTT
GGATCTCTTTGGGGCGCCTCAGCATATGACAGTGACAAGGCCGAAATGCAATCTTAAATTCATG
AATGCTTTATTTGTGATTTTTCGCTGGCTTAGATAAAAATGTCTGGCCGTGCTTGCAACCTGTT
GGGCTTACAGATTTATCAATTGCGTGGCAATAAACAATATTATTTGATTATCAATATTTTATAG
TTCGGATTCCATTCATCTAGACGAAAACTAAAAACTTAACACACCTACTATTTTGTAAAAGTAA
AAATAAAATTACTTTATATATATATATATATATATATATATACTATACTATATATATATATATA
TATGTATAATTGAACTGCTCGGGCGCGTGGCCAGATCGACTCGGCCGTTAAATCTGACCAACCC
ATTTGACACATTGCTATACTAATGACCAAATCATAATAGTTCATACATAATCTAATAAAAAATA
GGGTGTTCTTTGGTAAATTAAGTCCAACTTTAAGCCATGAATTGGTTAAATAAAGTTTGCTAAA
ATGTTTTTCAATTACTGTGGCAAACTCTTGTTAAGCCTCAACTCAAACTGTGCCCTTCCCAATT
CCCTTCAGTGACTGAATTGCAAAAAACTTATCTAATCGCCAGAGATTACATGGAAAAAAGGCTG
CAAAGGATGTCGTGCGAAAATCTTTTAACAGATTTCCATAAATTCATATATACAGACACATTTT
GTAAAAATATATGTTAATATATGCGTGCATTTGTGTCTGTATCTAAAAGGGCTGTGTCATTAGC
TGGGTCTAATAGGAACGCCCTAAGCCTCAAGGGCTCACAGGAGGTGGGTTGGGGCACAAGGAGA
GGTGGCGGGGAAAAGCTTGTGTATTTTGCGAGCTTGTCTATCTGTTGTGGGCTAATGTCTTCAT
TACGCACAGCACTGCCACGCCCACAAGCGTACGCCTACCGTAAACTGTATTTAATGCGCATATT
TATTTTATTTAAATATTTATGTATTTTTTGCTTCGCCTTTGGATTCTGCTTTTGAGTTACATTT
TATTTGGTTGTGAATTAACTTAATTAATGCGTCGCATAAGCTGCCGCTTAGGCATTAGCTGACA
TTCTCGTCTTTGTTGTCCTTCATCAAGTCAATCACAACGGCGGCAAGAACAACAAAAACAACAG
CAACAACAGCTTGTTATAACATTTCAAATGTTGCTTTGGCAGGTTTATGTTTTGTCGCATTTTT
AATTAATTTCGCAGAGGATTTTTGCGGCTTATGAACTTTAAATATTTCCAGCCCCTTACGCCCA
GTCCACGCCCCCTACTTGCCCAAAGCAAACATAAATTGACAATTCAGAGACATATGTATATATC
TCTATATATATGTATTTACATACATGGTATATCTATCTATATAGGTAAAACACGTATGGGACAA
ACAAAAAAAAAAAACATCGACCTAAATTATGCGACACGCCTACATTAGGTATGCCTGTGTGTGT
ATGCTTGATGTTTATTTATGTAGTTGCTGTTGTGTTTGACATGTCACACACAAAAACTCACACA
CACACACACACACACACTAATAGCAACAACCACCGCCGACGCAAAGCACGTAACTCATACGC
CATGCAGTACGCGCGCCCATTGGCTAACAATCTTTGGTAGGAATAGTAGAAATCGTATACAGTT
TAACTTATGCTGATTCTTATCCGATTTGCAGTTATACATCACAGACATATCCTGGAATGAATCG
CTAGCCAATCGGCAACAGACTTTCAATTGATCAAGCTATATACTCAAATATATTCATATATTTC
TAGCTCGACGAACTCAGGTTTTGATTCGACAATTTAGTTCGTCACGTATTTTACAAACTTTCAT
TGGAGCAATATAATTTTATTTAAATTCATTACAGTAAATGCACATATATTTATTTGTTGAAATT
AGTTCAATACGCACAATACCAGACACTCAGTTAAGGTTCGGCTATATCGTTTATACATACTTAG
TTCTAAGATTTTTTGACTTCGCTCCCACAAATTGCAAATCTTATATCTAAAAAAGTTTTCAAT

CACATTTTTGAACCACCTTCGTAGGTTTGGAGTCAATTGATGTTGATCGATATTAAAAGAATGT
ATCAAAAAAAGATAAACAGCGCTTACATCAAATATATGAGCTCCTCGAAAATAACAAACGGATC
TACCTTGTGAGTTCAGATATTTGAAACAGGGTATAAAATACTTGTCGAGCTATAGAACTACCGA
CAGACTCCTTGGTTGTATGGTTTTAATACTCGCGTAATTATATGTTTGTGAAAATAGGGAAAAG
CTCGACCACTTTCAGGTTTGCAGCACAAACAAAAGGCAATTAAATGCAAAACTGGTGTTTTATC
GCCTAAAAGAATATATGTAAATAAATATGTATAATTTATAATATTTTTAGGTTTAATGTATTTT
TAATATGACCTCTCCAGTATTTGCTAAGGTCAAGCGTATCAATTACGATACGAACTATACGAAT
GAACGAGAACCAAGCAGCGGTTTACTTTTTGTAAGAGTCCAGTGAGTGGGCGACAGTACAAGGT
GTCTACTGGGAAGTGAGGTGCCTAGACAAAGGTGTAGGGCAACCCAAACCAAACGCCAACGCAT
ATAAAAGATTAATATTTAAGGCCAGGCCAAGCGCCCGGAGGCGATGCCCACGCAGCAATGACTT
TCATGGCATCCTCTTCGAATGCCTTTGCTACCCCTCCCGCTGATTCTATCTGTTCTGGCAGTGT
ACTCGCATTTTGTAATTTATGATGGCAAATTCTCTGTGTGAGTACATCATACTGGCACACACTC
ACACACACACACACACACACACGCACACACGCAGATGTTTACACATGTATGCTGAGTGTTTCCA
GAAAGCAACAACAAACAAGTCTAATGCAACAACACGTGGCAGGCGCGAGAGCTTCAATAAAAAT
GCATAAATTATTGTTTATACGTATTGAATTAAGCGACGCAAACACTAACACGCCCCCAAAAATT
ACGCATGCCACGCCTTCCCCCTCGCACCACCCACACACATACACGCTCTCGCACACACTCACAC
GCAGATGATAAACTTTGCCGAGATTGGAAATGTATGCAAAAAATGTTTCGCCGCTGTCAATAAA
ATAGAATTTTTCTATCCACGCCCACAAAATGCCAGGCGCAGGCATTATAAACAATGAGGCTGTG
CCAGTCGACAATATTTGTTATTTATTGAGATTTGTTTACATTATATTGTATGCGGAATTTGAAA
TGCCAGCCCCAGAGTGCCACGCCCAAGTCCCCATTCCGCCTTCACCGCCAGACTATGCTCCGGT
ATCCTGTGCCGGCCACCGGAAGCCGTTTTCCTCTGCCCCCAACATTGTTGCTCTACATATTTCC
CACTCATCCACTTCCTCCTTCAGCTGAATGGAGGCATTTTAATATATGTTCACTTCACAGCCTG
TGTGTCAGTGTGTGGGCGTGGCATGTCAACAGGGAAATTCGAAACTGGAAAAGCTAAGCGAGTT
GTTGGACGTGGACGCGCTATGTTTCCCTGTCTATACTTGAGCAGATTTTGGATGGACATTAACC
ACTATAAAATGCTAGTTATATGCGTATATATTATGAAACTAATAATAATAATTTCAAATAAATA
TATTATATTCAACTGAATCTAATCGATATCAATATAATAGGGGGTATGCCCTGATGCCCTTATT
TTAATTGAGTACAACTTATTTAGTTTGTGCTTAATTTGTTATTTCCTGCACATACTAAATGTAT
GTTATCTTAAATAGAAATCTTTAGTATATTCGGCAATGAAAATATGAAGCTTTTATTTCTACTT
AACATAATTGTTTTTCTGTATTGTACTAACAGGAACTATTTAGGGTCAGAATTCACAATTAAAT
GTATACTGAATGTGAATGAAGACAATGGGTTGGTTTTTGAATGGTAAACTTCTTGCCGAATATC
TTACACACGTACATCGTTAATTGTATGTAGATTGAGTTGAATATTAATAAGCACTATCAAATAT
AGGGAAATCTATACTTCTTCCTATAATTCCATGACACGAAACGCACACTCACTGTGCCAATCAA
ACATTTGACATTGATCTTATTAGACATATATACAGACATTTGAAATACCAAGCATAAATCAGCA
GAATTCATGAGCCATAAAATCTTAATATTCATTTAAATTGCTTCAGTTGCCATGGAAATGAGGA
CTAAATAACCCAAATGTGTGCATGTAGGTTTATATATGTTATATTATATATATATATATATATA
TGTATATATAAATACATCGGGCTGAGTTGAGGCAAGCCTCTGTGGAGTCATTTGAGGGATGGTC
AAGTTAACAACTTGCGATTGAGCTGGGGTGAATCTTTCCTATAAATCTGAGGCGGGGAGCAACT
TAATCAAAGCCGCAACTGCCATAAAATTAGAAAGCATGCGATGAGGTCGACCAAGCGATGACAA
TAGGGGTCACGGTCAGCCTTAGACTTGAAGCTAAGGGCCAGAACGAGTCGGCACGTAAATCCTT
CAGGCTTGCAGGCGTCGAACAGTTGATTAATTCGTCGATTTTATTTGGCGTTTTTATGAGCAAC
TGCAGCCGTGGGTCGTGTGACTGCTCGTATGTCAGTTCGGAGTCAGGCTGTTACCAACTTTTGT
TTGCTTGACATCAGGCTTCGGATTACGCGAAACTTGACNNNNNNNNNNNNNNNNNNNNNNNNNN
NNNNNNNNNNNNNNNNNNNNNNNNNNNNNNNNNNNNNNNNNNNNNNNNNNNNNNNNNNNNNNNN
NNNNNNNNNNNNNNNNNNNNNNNNNNNNNNNNNNNNNNNNGGCGTCAAACCGGCCCAACATGGA
GAAAATGCGATACTTTACTTTACCATTAATCGTAAAAATTACTCATTTTATAGCTTTCGAAGTG
TTTTCTCGGTGCCAGTCGCTGAGAAAGTTTGCATGTGCCACCGCCTAGCATAAGGACTCAGGCT
CCGTGAGTGTCATCGAGTTGCGAAGCGCTGCGGCGTGAAGACAAGCGATGCGTGAAACAGTCGA
GAAAAAAAAGACCAACAAAGCCTGACACCAAATGCCACACAGCGACCAGGTGGCCTCCAGCAAT
GCCTCAAATATCGATGTTAAGCGTTATGCATAAATCAGCGTATTGTCTCAGATATGTCTTCTTT
TCGTCTTGTGGGATGTGCCGGCAGAGAGGACATGTGTAACTGCTCAAACGCATGTCGGAGGGGT
GTGTGCCTATGTGTGTGTATACATACAGGTGTGCAAGGTATTCGATTAATATTGAGGAAGCCAA
GGATTTATATCCTAAGAATCGTTCTAATTAAAAGGAAATCTTTTATATTCGTTGATCCGAAAAT
AATTAATTTAGCTACAGGCGTAGCATATGAATTATGAGCTGCTTGTGGCTCTTAAGTACTTAAT
TAATTGACATACTTAAGAGTTTAGATGAGTAATAATTCAGTTTAGGCAATATTTTTAAATGGAT
TTTTCCTATTAAAGCAAGAGTCTTCCTTTTGATTTTCATTTATCAAAAACAAAACCTTCTTACA
TCGCATTAATTGGGTAAAGAAAAGTTGAACCCAAAACGAAACAAATGTTTAAAAAAAATATATG

TACTAATAGCATTTAAAAAAATTTGTTCTGTGAATCTAAAAACTTATTTTTTATAAATAAATTA
AAATGTCAAATTAAATATCTTTCAATTGGGGCTAATTGCTGCATAAATGTTTGAAAACAGATCA
AATGTTAGCCTCAAATATGCTTGTCTATTAAAAGTTCTATTCAACACGGAAGGCTTGTCCTGAT
CAAAAGGCTTGTTGGTTATTTATAATTGAATCAATATGCGGGCATTGTATTTAAATTGAAGACA
AATATTTAAAAATTGGGCACCTTTACCTAGGAGATATAACATGTGCCTCTTATGGAGGCACTGC
TAAAAAGCACAACAGCTGCAGCATTTTTATTGCACACATACATATTAATACATTTTTATGTATA
TATTTTTGTTATTTTGTGTTTCTTTGATTTTATTGAAGGGGCACTTAAAAACGAAACAACTTCG
AAAATAAAAAGGCGACTCAACCTAAACAAATTTCACGTCGTGTCCTTCCATGGAACTGAGCACC
CAGTCGACGTTCATCTGCCTTCAATTTGCCCGCCCCTACGCGCGCACCCATGCCCCGCCGCAGT
TATTACTCATTTACAGACATTTACTTAAAGCAGGGTCTGGGTAGAGTAGGAAAGAGAACTGGTG
GTTGGGGACTGGGGGCATCTTCTGTTATTTGCATTGGACAACAAAATGAGCAGCGCCGAAAAAG
TTTCAAATCTTTTGAATGAATTTAATATTCACAAAGTATGAAAAATACTCGCAAGAAAACTTTG
CACAGAGCAGCGAGAGTTAGGCTGGGGTGGAACACCATCCTCCAAAGTTCAAACTCCTTACTCC
ATGCCCATAATTACCATTCACATCATCATCCACGTCATCATCAACATTATCCTTACCCTGGTTA
GTATCAGCAGCATCGTCAGTATCATGTGCTCGTATATGGTTTGTCTATTTTATTTTTGAATATG
TTTCATGAAAGACTTCTTCCCCCTCCGTTCCGACCAGTCTGGCAGCCACTTGCTCGCAAAAGGT
TTAGCATGGCTGGCCATGAATACCATCTGGCTGGTGGCGGCTGACGGATGGCAGGTGGCAAGTA
CAGGTGGCAGGTAAAAGTGACAAGTATATCGACATCCGGAAAGTTGCAATTTGTGCTACGTTCT
ACCGAAATGCTTTGCTGACTTTTCTTACATAACGTTTTAATTGCCTCTGCAGTAGAAAAAATAA
ACCAAAAGAGAAGAAATGAATTGTTTGCGATTTAGTCATCATAAGGACCTCAAGCCAGAGCGAA
AAAGTTTAGATGAATTCTGAATGCAGTCGAGTGTGGAGCAGATTATGAGAAAATTAACGACTTT
TTAATTGTTTGTTAGGGTTTGTTTTGCGTTTTAGTTTCTTTTTTGTTTTTCTTTCTTTTTTGTT
GATGCTAATTGGATTGCCAATTAATTATTTGCATGTCCAGCTTGAGCATAAATTGTTAATTCAG
ATATACAACGTAAAATACTTTGTTTTTAATTTAATTTAAGTCCTAAGCCATATTAAGTCAGTTG
GATCTTGTTCCCATATGTAAATCAAACGCTTCGTAAGCGTTGCTCATTAAAACAGAAGCCGCTC
AATTTTTTTTAGTAGCCGCTACGGCATTCCAAAGCGGGTCAGAGTCCTGGGTCCTGGCTGAACC
CCTTAGAGAAGCTCCTTCAAAAGGTACGTTCTCTTTTTTTTTATTAGTGTAACACCTTGCCATT
GTAACATATGTTTCCTCTAAGCTACACAACGTAGAAACTGTAATCCACACTCTGAGTCCATTCG
CTTGGTGGAGATCAGAATGTTCTGATCGATTCTAGCGTACATTAAAAGTAAAATTCGATGCAAC
GCGTGGTTTAGGTTTCCAATCAAAAGTGATGCCGATTCAATCTTGACTCAGTTTTGACAGTAAG
ACATACAACTCAAAGTAAAATATTACTACTAGCGGGTATTTCCCGGCAAGTTTTCGGCTTTAAA
TTTGAGAATAAACTCTTTCAGCCCGTAGACAATTTAAATATATACGTACTTAATGCCGCAGTCC
GCTCCATAATTGAATCCGTCTTCTCTATTGTTCAAGCTGCCATCACCCCATCTGCTTACGTTGC
CGTCTACTCCATAGTAGAAGCCGCTTCCTCCAATATCAAAGCGGACACCGCCTTCACTTTAAGT
GAAGCCTGTGAGACTTACTTGAATTCTTGAGAATCGTAGTAGGGCATTAGTTAGGAAAACGTTT
TTTTTTTTAATTTGCAGCGCAGCTAAAGAGAAAATGGGTTTTGCATATAAACATACAAGAAATA
TGCATAGATTCATCTACATATATAAATGCAAACATGTATGCATACAAACATACAAAAATTCTTA
TGTAAATACATATGTACCTCTATACAAGCATGTACACATACTCGCATGCATACAAAAATATACG
TAAATACATAAATATCTATATGTATATGCTAGGCACATCCATACAGATATAATGCATACATAAG
TACTCACATCAATGCATACTCACATGGATACACCTATACAAACACATATTCAAACAAACATATC
TAATCACACATATATTCATACATACTTACATACATACATACATACATACATATATATATA
CATACATACATACATACATACATACATACATACATACATACATACATACATACATACATA
CATATATTCATACATACATACATACCTACATACATAAATATGTAGATAAATATATACGTACGTA
CATACACCGATCGGATGGTCAACAAAGAAGACTTTTACAAACGTATAACCCAAAAAGCCATAAG
GTATGGACTGTGCATTGTTCAGCAATCAGTCAACTGATCAGTCAAGAGAAACAGATTTATTTAT
AGGGATATATAAGTGTAATTTGCAATTGAATTATTTTACCTAGGCTTATTTATAGCACTTAAAA
ACAAATTCATTTGCCCGTTTTCTCATTGCTATTCTACTATTGTAATTTCCTTATGTAATTTTCA
CTGAAATCGCTTAGTAAGATTTACATAACGCTTCATATGTTGTTGGCCAATGAATTTGTTGCCT
TGCAAAAAAAAAAAAATCACAGATGAAAATATGGCAGCCTCCTGCACCTTGTAAACAAAAAAAA
GGCAACTGACTGCTGTTAAATAAGAATTTTCTATGCAAGCATTAAAAAGGATTTCAATAAACTA
AAAATACATAAATTTCCAACTTATATCTATCCCTTTGTGTAATTGTATACACTAAGTTTTGTTG
CAATATAGATAAACAAAAGGGATTATTTCAATTTTAAAGTATTGACAACGGTCTTAAAGGCAAT
AGAATATAACAGACTAGCTTCATTAAGAATTCTCTACCTGAATAGTAACAAACAATTAAGAGGT
TCTAGTCGTTTTGCACAAATACAATATACCCTTATACCCATTTTTAATGGGTTCAGGGTATAAA
AATAGCAAAGTTATAGCTCTGTTTTATTCCAATTTTAATTGTGCTAATATTTTCTCTGTACATA
TACATATGTTAACTTCTTAACAGACGGTTAACTTATGTTCCAGGGCTCTGAATGCTTATCAGGA

TAATTCAACATTCTAATGAAATGATTATTACCTGAAAAAGTAAAGCAAACAAATTTAAATTGCA
CTTGGTTTGATCATGAAGATCTGTAAATATAAGTATATGTAAGTTAATCAGTTAACAGACGGTT
AACCACATGTTTAAGTGGTTAAATAAAACCACTTAACCTTTTAACAGACGAATAACATTATGTT
CAAGGTTAATTTCGTGTGTTCAGCTTAAATAACAGCCAAAACCATAGGCCCAAGCTCAATCTAA
AAAGCTATTCGCAAAATCGTTGCCAGCACCGTTGACAGTCCAATTGACCGGGATGCGCAGTCCG
GGCAGTCCAAGCAGTAAAAATCACTTTGAAGCCAGCTCAAAGGGAATTCGCACGCACAGGATCA
AATAGTGCCGTATGTGGAAAGTGTTTAAGCGAAGAGCGTTGAGCGATGAGCATTGAGTAATGGC
TTTAATGATGGCCCAAGCACATCATCGACTGTTTAACGGGACTTTAGCCTTTGAACCGTCACAC
ACACACATACGCACAAACACAGTGAACGAGCAGCGGGGGCATTAAAAGAGCATTAAAGCATATC
TGGATGCGAAACGCATCCGCTCGATCTTATTGCGAAACATATATGTCCATGCATATATATACAT
TTTTATATACATCTATATATGTATATAAATGTGTGTGTGTGTTGGTAAATGCGAAAATGTGAAA
TGGCAAATTCAATGAAATATTTATTAACCTTTAATCAGCGAACACAGGCAGACAGCAGACAGCA
GACAGTTGGCCAATAGACAGAGTAAAGCGTCGCTCTAAGCATATTTTATTACACATTTCATTGG
ACAACACTGCGTATGTGTGATGCGATACGTTGTGATGTGATATATTTGCTTGATTTATTTCTGC
TGCTGCAATAATCAGAATTATTTGGTACCAGGTCAGTATCTGAATGTTAATTGCCATGCCAGAC
TAGGCCAACTTAAGTGGAATGAGATTATGGTCGGATATTGCTAGGGTATATTTTGGGTATAATG
TGACTTCAGGCAGGAAGCATGAAAATTGTGAGAGAAATTGCATTTTATGGCTTTGCATATGTGA
GCGACTCTTAATTTGATTTGTTCAGCCAGCATGAAAATCTGATTAAATAAACAATTTGATCAAT
TTTGATTGTTTTTGTAACCAAACCAAATGAATTGTAATTAAGTCATGCACAGCATAAAAATAAC
TAAAACGATTGCTTACCCAATTTAATATAAAGCGTTTCCATATATCGTTTTATGAAATTTAATC
TCGAAGCGCTTTCCTTCAGTTCATTGTATGCTGGCCATTTCGGGCCTTAATTCAAGTATTGGAC
CAAGTTCTTGAATCGCACCGCCCATTGGGCCGAGTTTCAGCTTGTTTCAGTGTTGGCTTGACAT
TGTGTGTCAATGTCTCATGTTTCAGCTGCGGAGCGATGTCCAGCACAATAGTTGTCAGCTGCAC
AATGCTAGAGCAATGGCCTGGGCTTCCACGCTAGCCAGACAATAGAGCCCCAAATCTAGTCTGC
CATACATACTAAAGGGATTTAAACAAAGTACTTGAATATGCCGTCCGTTTTCGTTTGCAGCGGT
ACTCTAAAAAATAGGTATACAAGTCGAACCGGTTTAAACGAAGCCGAGCCTTAAACCGGCCCCA
AAGTACACATAAGTTATGTAGATCGGGCAGTAAAGAAAAATAAGTATTATTATTTCTATTATTA
AAATAAACGAAAATTGCTAAATAACGTTTTGACAAGGCCAAGGCCAAACAAATGATCAGAATGA
ATAAATAAATTATTTGGCAATCCGTTTTTGAGCCAGGAGCTGTTTCTTCTAGTCTGGCTCGCAT
TACTTCAGTTCAGTTTTACGCCTTGATTTCACACAACTAACTCAGTTGCCTTAGTTTTTCACTC
ATTAGTGTTAGGCGTTCAGCATGACATTAGATACCCTAAATAAATTAAAGTAACATTTCTCCTA
ACACAATAGAAAGATTATCTTATGTTTACGTTCCGATGAGTCTATCAGCTTATCCTTAAATCAG
CTTAGCCCAATTATCAGCATAATTTGAAAGATTTTGGAGCCGCACTAAAAAACAGATTTCGAAA
AGTATTGAGAATTTGTGAAAGCATTCAAGGAATTAATCGTAACGTTTACCTATTGACTACAACG
ACTGTCTCTCTAAGTGTCTCCCTCTCTCTCTCTCTCTCTCTCTCACTTTCTCGTTCTCTCTG
TCTGGACTTAATGTTTTCTTTGCGAGCAAAGAGGATTCAGCAGAGGATGGGTGGGGGGTTTCTC
TGTTAGTTCATCTTATGCTGCTCTTTGAATTTTTCGTGTTTTCCCAAGTAGCTTTCATTTGGGG
GGCTTGAACTTGGCCAAGTCAAAACGCGTGGCGTGCCAAAGCTTCAGCTACAGCTGAATGCTCG
TGTTGTTGTTGTTGTTATTGTTGTTGTTGTCAGCCTAATGTGCTTGTATTTACTTAAACGCACA
AAACGGCACAATTCATTGGCGTTGGGGGCCAAAGGCATTGTCCTGGTCCTGGTCCTGGTCCCGG
CTCTGTGCACAACATTAGCGCCCACTTTACTTTTGCTCAACCACTGATGAGCAGCAAATTGAAT
TTTATTGACTTGATTTGCGCCCCGTTGGCTGCATTTCATTTAGTTTGTTGTTACTTTATTTATT
TGCCTGACATTTCTGACCATTTGGCTGGCCTTAGACATCCAAGCATTCTAGCCAGCTCCTGTAT
ACACAATAAATTGATTTCTGATTATCATTGCATAATAAAATGACTAAATCTGCTCGCGCGTCCG
CTGACTAACAGACGCACTGAAGGCCAGGCAACCCCAACAGAAGTTTTAATGAAGCGTATTAAAA
TTTAATTAAATAACAATTTGCACAAATGTAAATAAACACGACGCTGCTTATGCAAAGCGTGAAA
GAGCAGGACTACTAGAAGACTCCAGGAGGACGCTAAAGGCTGGAGGCTGGATGCTGGATGCTGG
CGATTTAAGACGTGGGTGTACCCCCCCGGCTGCCATGCCAACGCTCAGTGCGGCGAACTTTGGT
CATTAAATTAAGTGCACAAGACGAGCACGGCGACGACGAGAACACGGACCACGATGTGACGAAC
AAAAGACGAAGATGACGCCGACGCCGTCATCGACGGCGACTCAAACGATTGTTGTGGTCCCTGT
GGCTACGGACTGCCCACATAGACCACGCGCGAGTGTCTATCAAATTTAAATCACGAACGCTGAC
ATAAAGTCGCGTTTTATATATCCACGTCCCAGCCTGGCATTTGAAAAACATAATATTTTTAAGC
ATTTTAATACGTTTTTATGCTCTGCAACAATTTAAATAATAGGCAGAAGAAGGCATCTTCAAGC
CTATAAAAAATATACATTGAATATATAATAATTTTAAATTGGCAAAAATGTAAAATTTTATATT
AAAAAAAAAATTGTTTTCGGTTTGAAATAAGTATATTTTTTATATGAAATACTTTGTATTTGAA
AAAAAAAAAGTTGGCGGTTTTTGAAAATGTGTGAAAAACGTCTCAAGTGCATTTGTGCCAAGAA

ACTTGCATAAACGATCGAATGATAGGCCAAAAAGTGTTATAACAGAGTTTTTTTAAAAACCCT
TGACTTAGTGCCTAAACTTTGTAGTGGCTGCTTTGCACTCCCCAAACGAATCTGCTTGTTGATC
AGCTGCTCTAGTTTAGTTACTGCTTTACCGACCGCGCTGGAAGCCAATTCTTCGTTTATTGCAC
AGCTGAGGGCATGCGCAACCGAATAGGGCGAATAGAGAGGAAAAAGGTGATAAGATCAGGCCAT
AGTACCGTTTTAAAATATAATCGCGTGATGTAGAGCTTTTGAAAGCTTCCTTATTTTTTTTGTC
GTTGGGAAGTAGTAAAGGTTAAAAGTACAGCTTAGCATGTCAGATATATTTGAAGCGCGTGCGA
GTTAAGTTCATAACATTTTTTCTTTCTTTGCAAATAATTTTTGCAAAGACATCAACGACCAAGG
CTGCAATTGTGGTACAGTTTGGAAGTGTTCTTTTGGTACGCTAATAGAAACCAAAAACCTTCAT
AACAACTCGACCCACTAACCTAATTGTGATTCGGGGTGACCCTTAAAGAAAAGGTATTTATATG
TGCTAGTGTTGAGACTTGGAACTAATTAAGGCTTCCACAGTTTCCGTGCCTGCTCAGTTGACTA
GATTTAGACGTACTAGTCGGCACTACCAGAGGGGGAGATCGGACCAGACAAGGGCAACACCAAG
AGCACGTGTCGCACTTAGCAACCCAGCAGAACCAATTTTGTTGCTATTTTACTTATCAGCCGCT
GTCTTGCGAAGAACAACATCTGGCATAGCCTCGGAAAACGGCGTCAAGCGGGTGAAACGCTTAC
TATTTATGCAGCACCATCGGGACCGCATGCACTAAGCATATAGTGTAACCTGCAGAGAGTAACA
AAGGAGCCGAGTCTGTATTACTTTTGGTGTACCCCTTTCGCTTGCTTAGCAGCATTGCCATATA
ATTCCATTAATTGTTTAACGTTGCCAAGATGGACGGCTAATAGATCCTTAGTTTTAATTTTTAT
TTCCTTTAATTTTTTTTTCTTTTACTAATTTCTTTAAAACAAAATGTGCAATGAATATGCCTAA
ATAAGTTAAAGCGAGAGGAAATTCCTACACCACCGACCTTGACCGTTGCTCATATATATATATA
TATATATATATATATATATACATGTGTTTATATACTTATAAATTTCTTTTGTAATATTTAGCAA
TAGCGATCATAATCGTCGTATACATTGGGCCCAACGTCTGCTTCGAAGGATATCGCGTGAGTAA
GAGTTTAAACCAATGCCTATAGATATATGTAGTTATATACACACATGAACATTTAAATGCAGTT
AATTTAGCAAGACGGTAAATTAATTACTATCCGTCAAGTCTCAAGATAATGTTGGTGAATTTCT
CCTTCCCCTTAATTTCTTTATGGGATTTAAACTCGTTATTGCACATTCATCATATTAACTTTGG
ATTCAAATTTTGTTTGGAAACTGTATATTTTTTCTTGTAAATAGAGTTATACTCACCTTGCTAT
TAATTGGCAACAAAATTGAACAATGCGTGAATAAACGAATTATATTAGCTTATATGATATGATA
TCCTAATTCCATAATTAATTGTTTTATCTGTCAAGTACCCATGTTAGCCGCTTGCAATTTATAG
TGATGGTTTAAGAACCTGAAATAGGGACAAGCATCCACATTCCATTTTTTTTTTGCTTGTCAAG
GTAGGGAGGGTAAAGAGAAAGAGAGACGATCAACACCTTAGTTCTCTCACTTACGCGAAATTGA
ATAGTTTGAATTTGGGTGTTCGGTCGGAACACATTAATGGTACCCATACTAGAATCCGGTTTTT
TTTTTGTTTCAATGGATTTACTTAAATCAATAAGTTTTTTTTATTTTTTTTGATCGCACTTAAA
CTATGTGTTTATGGTAAGTGCAAAAGGGAAAGGAAAAAACCCCGACCAGTCCGACGAGCTATTG
TCCGAGTATTAGCAAGAAGTGCGAACCTCCAATCCCTATGCACCCGGTTAGGCAACAGCCACGG
CCGTTTGTTTTTATTTTTGGTAGATGGCCAAACGAAAGGCAAAAACTGAACCCGAACCTCCCCA
CCATTACATATATGGCGCCCAACACGTGGGACCCTAGCAAGAACGTAAAACTTTTACTTCTTGC
GTAAGCACGACTAACACCTAGTGCACAACAATCCCCAATCGAAACTCGAAGTTATACCATATCA
ATTACTACCTATTATTTTTTAATTACCCTGTTAGGCCCCTTTCTCGGGAAAATACGTTGTGTTC
AGACAGTAGTTAGTTGTAGGAATTTGCGATTGTAATTCAGGCATTGCTGGATTAGTCGACCACA
ATTAATTACTAGTACTTTAAGCAGCGGGTAAGATCGGAAAAGAGGGAATAATAATCAAATTTGG
GTTAATTGTTTCAAGGACTATTTGGAATTACAACAAACTGGAAATATTTCGGATGTTGTCTAGT
AGTCCATGAGCAGTAAAAATCCAAATCAGAAATAGACTCCAGTAAAATAAATAGCACAAATTCA
ACTACTCAGGTTTGCTTAAGGATTGTAAGCAATAACGTGTGGGAAAAAGACAGATAGAATAAAT
AACCAAGACTTAATAATACAAGTCCTATTTAGTTTTCCTTTTGTTGTTTGGAAACAAGCAAAAG
TTTGCATTCATTATGGCAGTGAGACGAAGCACAGAGAGCATAGTTGCAGCGATTAACGCGCAAG
ATCCATTTTGTGGCATTTGCCATGAGCAATTGCAAGCAGAACAGCTATCTGCGACTACTCCTTG
TCAACACCGTTTTCACGACGTTTGCATAAGCAATACACTAAAGACAACCCCAAAATGTCCAGTA
TGTGAGGCAGATTGCAACCCAGGGCAACTAATATTTATGAACAATACACTGGCAGCGGAGTTAC
ACTCAGCTGGTAATGCTGAAACAGACGAAACTCTATCGCGAAATACTCTACCTTGCCAAGATGG
TTCGACTACTACAGGTATCAGGAGAGGTCAGCGATTCGGAAAAGGGCGAGGAGCCACGCCCAAG
CGAAGTATGCAAACTAGGTCTATGAATCGAAATCATAGAGAATATTTAGGTAGTTGCATGTCTT
TAGGCAGCAACGGAAATAAGTCAGTGGACAATGGTCCAAATGTAGAGACAATTACCTATATCAA
CGAAGCTCTACAATCTCAGCAAAGGACTTTAATAGCAGAACTCAAAAACGTTATTAAAGATTCT
GTTGAACAATCACTGCAAGAGAAGTTAGCAACATTAAATGTACGAAGTGAGCAAGCTGAATCAA
CACGCTCTAGATTAGTTCAGTCTCCAAGGGGATTAGGTGCTGATACTCTCCCTAATAGTGCCCA
GAACTCTGACATACCCCTTGACTGCGGACCATACAGCCCCAGAGGTAGCAATCGATCAAACTCA
AGTTTGAGTTCAGAAAAAGCAGCTAGCATTATGCAAACCTGGCGACTTAAATTTGACGGTTCGA
AAGACTCTATGCAGATAGATGAATTCTTGTATCGAATTCGTTCATTAACGGCTCAAAATTTGAA

CGGCGACTATAGGCTGCTTTGTGACAATCTTCATTTACTTTTCTTCGGGAAAGCCAATGATTGG
TTTTGGAGCTTCCACAAGAAAAACCCACAGTATACCTGGTTGGCTTTCTGTACGGATTTCCGAC
TTAGATTCGAGGACGTTAAAGACGACTTTGACTTATGGGAACTAATTAGGAAAAGACGCCAGAA
AGATCAGGAGACATTTGATGATTTTCAATCAGCGATTGAAGGCCTGGTTAGCCAATTATCTAAC
GAAATAAGCGAAGACAAGCTTGTTGACCTATTAAAACGCAATGCGAGAACAGCGCTCAGGTACG
AACTATTGCATTTGAAGATTAGAACTCGAGCAGAATTAAGAGAGGAAGTTAAGAAACATGAAAG
TTTCTGTAGAGAAACTGGCTCGTTTCCCAATCGAACCAAATACGGTAGAGGTAATGTCTCTGAG
TTACTGGAAAATAATGATATAGAAGATAATATTTCAGAAATACAGAACAAGAAAATAGTGTGCT
GGAATTGCGATAAAGTGGGACACCGATTCGATGACTGCATGGGAGAAAGGCGTATATTCTGCTA
CGGTTGCGGTGCTAAAAATACTTTTAAACCCAATTGTAGCAAATGCAATCTTTTGGGAAACCGG
AAGCGGGATGCGCAGACCAACACCAGTTTGACGCATCCCACAAAAGAATAAAGTCCTTGCCAGA
AATACAGTCATATGGTAGTTTGGAGCAATCAACTCCAAATACTATATTTACAACTTATATGGCA
AATACATGTTCAGTCGCCGAGACTGCAACTCAAACTGATATTGAAAATAAGTACGTACCGTACC
ATATTAGAGTAAAACACTATCTTGACACACGAAGACGAATTTTTAGCAGCATAGAACCCATCGA
ATTTCTCAGTCGGCCAAAACGCTCTACTGTTCGACTTAGGAAATTCTGGAAAAATATTAAAAGT
CTTCGTAAGAAGCTCGTTCAATCAGTGTTTATGAGCTCAGATAACCGCTTGTACACTGAGTTAA
GCCTAGAGGGAGAAAAATATGTAGCATTATTAGATTCCGGAGCGACGATTAGTTGCCTCGGTAA
TGTTGCAGCTAAGTCACTGTTGTCACATCCCAAAGCGCGCAAATGCTCTGGATCAATCCGAACA
GCTAACAACGCTATTTGCCCAGTGGTAGGCAAACTGACACTTAAAGTTGAATTTCGAAAACAAG
TGCATAATGTTGATTTTTATGTTATACCCGATTTGAAACAAGATGTTTACTTAGGCATAAATTT
TTGGCAGGACTTTGGACTGTTAACGAATTTAATTCGTCCTGATGAGTCCGTAAATGAATTAGAC
AACAGTAACGAACAGGATATCCAGGAAACAGCGAAAATGCATCAGCTAACCGCGAAGAAAAGTC
ACGATTAAATTCGATCATTTCTAATTTCTATTCTTATGAAAAGGAAGGATTAGGACGCACTGAG
TTAATCGAACATCGCATTGAAATAGATAATGTGACCCCCGTCAAACAAAGACATTGGCCTATTT
CGCCAGCCGTTGAAAAGCTAATGTTTGACGAGATCGAGAAAATGCTAGCTTTAGATATAATAGA
GGTATCGCATAGCCCCTGGAGCAGCAACTGCGTCCTTGTGCAAAGAAAGGATAAAAACAGGCTG
TGTCTAGATTCTCGAGTCATAAATAAGTACACTCGTAAAGATGCGTACCCTCTACCCCATATCG
ACGGAATTCTTAGTCGTCTGCCGCCGGCTAAATATATAACGGGCCTCGATATGAAGCACGCATT
CTGGCAGATACCCCTAGAAGAAAAATCACGTCAGTATACCGCTTTTACAGTCCCTAATCGTCCA
TTATTTCAATACAAAGTAATGCCGTTTGGATTGTGTAACGCTCCTCAAACGCTTTGTAGACTAA
TGGAACGCGTGATACCAGCCCATTTGCGGACCCGCGTATTTGTCTACTTAGATGATTTACTTCT
GCTATCAGAGGACTTCGATTCGCATATGTTGCTTTTGCAGGAAGTAGCGCTGCACTTGCGTAAA
GCGAACTTGACAATCAACATCGCAAAATCAAAATTTTGTATGCGGGAAATTAAATATTTGGGCT
TTATAATTGGGTATGGTCAACTAAAGACGGACCCTAGCAAAATACAAGCTATTAACGAATTCCC
TGTACCAAAAAGTGTAAAACAACTGAGACGTTTTCTTGGACTCGCTGGATGGTATAGACGTTTC
GTTAATCATTATGCAACAGTTAGCTTTCCGCTTACAGAGTTGCTAAAGAAATCGAAAGTATTGA
AATGGAACACTGATGCTGATTCCGCTTTCAACACCTTAAAAACACTTTTAACTTCTGCACCTGT
ATTGGCTACACCCGACTTCTCAAAACCTTTTAGATTAGTTTGCGATGCTAGCACAACTGGTTTA
GGATGCGTTCTAGCTCAACTAAATGAACAGGAAGAGGAGGCTCCAATAGCATACATGTCAGAGA
AACTGTCAAAGGCCCAACGGAATTATAGTGTGACCGATCTGGAATGCTTAGCCGTAATAAGAGG
TATTTTTAAATTTAGAGCGTATATAGAAGGACAGGACTTCGAAGTAATAACTGATCATGCTTCC
TTACAATGGCTCATGAGACAGAAGGATCTCAGAGGACGTCAAGGTAGATGGGTGATCAAATTAC
AGGGCTTCAAGTTTAAAGTCTCGCATCGTAGCGGTACACAAAATGTTGTCGCTGATGCGCTATC
GCGTCAAAATGAGACAGAGCTTGCGGAATTCGAGGAGCAAGGTCCCATAGTTGACCTGTCTTCA
CCCGAATTCAAATCGCCAAACTATCTGGATCTGATAGACAGTATTTCTTCTAACCAATCCAGAT
TACCGGATTTACAGATAATCGACGGTTTTATATATAAACGTACTGAGCCAGCGACGGGATATAG
CATTCAGGAGAAACAATCCTGGAAACTCTGGATACCTTCGTCTCTCACCCTACAAGTGATAGAG
AGAGCCCACAATCCCCCTAACGCTGCACACAGTGGCATCGGAAAAACAATAGAGAAACTCAAGA
GATACTTGTTTTGGCCAAAAATGTCGGCCCAAATTCATGAATATATAGTCAATTGTAAAGTTTG
TCGGCAAACGAAAAGCCCCAATGTAGTGTTGAGACCTCCAATGGGCAAGCCTATGACGTCAGAA
CGGCCATTTCAGAAGCTCTACATGGATCTTCTAGGGCCATATCCTCGGTCCAAGAACGGCAATA
TAGGTCTCCTGATGGTTGTAGACCACAATACTAAATTTCATTTTATGTGGCCTCTTCGCGCGTT
TACTACCAACAAAATTATCGACTATCTAGAAAACACACTGTTTTGCACTTTCGGCGTGCCAGAA
ACTATTCTGACCGACAATGGCTCTCAGTTCATTTCTAGTGTCTTTAAAGCTTTTCTTACCCGGT
ATGGAATCATCCACATTCGAACGGCAATATACTCGCCACAAAGTAATGCGAGTGAGCGAGTAAA
TAGATCAGTATTAGCGGCTATTAGAGCCTATATCGGAAAAGATCATTCAAATTGGGATAAAAAG

CTGTACGCCATTTGTGCTGCCTTGAGAGCCAGCGTTCACCAAAGTACAGGGTACTCTCCATATT
TTTTAGCGTTTGGACAGAATATGATGTTAAACGGTAAAGACTATGACTTGCTAAAGCGACTTAA
CCTCTTGTCCGAAGACACTGCTATTGAGAGGCCCACAGCCTTACAAGTAATCCGTCAGATAGCT
AAAGACAATGTAGCTCAGTCACATAATAGAAATGCTAAGATATATAATTTGCGCAGCCGAAACA
TAAGTCTTACTGCCGGTACGAAAGTCTTCGCCCGCAATTTTTGTCAGAGTAACGCGCAAAAGAA
GTTTAGTTCTAAGCTTGCACCAGTTTTCATTCCGGCGATTGTTGTACAAAAAGTCAGCCCAGCC
TACTATCAACTAGCTAATGAAACAGGTAAATGCATCGGTACTTTTCATCTCAAAGATATACAAA
CTAGTAACAAGTTCTAACTCAACAGTTATTATAGTTATTTCAGTTTATGTCCCGCTAGCAAGTA
GCGCTGCATAAACTGTGGTGTAGTGGCTGCTTTGCACTCCCCAAACGAATCTGCTTGTTGATCA
GCTGCTCTAGTTTAGTTACTGCTTTACCGACCGCGCTGGAAGCCAATTCTTCGTTTATTGCACA
GCTGAGGGCATGCGCAACCGAATAGGGCGAATAGAGAGGAAAAAGGTGATAAGATCAGGCCATA
GTACCGTTTTAAAATATAATCGCGTGATGTAGAGCTTTTGAAAGCTTCCTTATTTTTTTTGTCG
TTGGGAAGTAGTAAAGGTTAAAAGTACAGCTCAGCATGTCAGATATATTTGAAGCGCGTGCGAG
TTAAGTTCATAACATTTTTTCTTTCTTTGCAAATAATTTTTGCAAAGACATCAACGACCAAGGC
TGCAATTGTGGTACAGTTTGGAAGTGTTCTTTTGGTACGCTAATAGAAACCAAAAACCTTCATA
ACAACTCGACCCACTAACCTAATTGTGATTCGGGGTGACCCTTAAAGAAAAGGTATTTATATGT
GCTAGTGTTGAGACTTGGAACTAATTAAGGCTTCCACAGTTTCCGTGCCTGCTCAGTTGACTAG
ATTTAGACGTACTAGTCGGCACTACCAGAGGGGGAGATCGGACCAGACAAGGGCAACACCAAGA
GCACGTGTCGCACTTAGCAACCCAGCAGAACCAATTTTGTTGCTATTTTACTTATCAGCCGCTG
TCTTGCGAAGAACAACATCTGGCATAGCCTCGGAAAACGGCGTCAAGCGGGTGAAACGCTTACT
ATTTATGCAGCACCATCGGGACCGCATGCACTAAGCATATAGTGTAACCTGCAGAGAGTAACAA
AGGAGCCGAGTCTGTATTACTTTTGGTGTACCCCTTTCGCTTGCTTAGCAGCATTGCCATATAA
TTCCATTAATTGTTTAACGTTGCCAAGATGGACGGCTAATAGATCCTTAGTTTTAATTTTTATT
TCCTTTAATTTTTTTTTCTTTTACTAATTTCTTTAAAACAAAATGTGCAATGAATATGCCTAAA
TAAGTTAAAGCGAGAGGAAATTCCTACACCACCGACCTTGACCGTTGCTCATATATATATATAT
ATATATATATATATATATACATGTGTTTATATACTTATAAATTTCTTTTGTAATATTTAGCAAC
AGCGATCATAATCGTCGTATACATTGGGCCCAACGTCTGCTTCGAAGGATATCGCGTGAGTAAG
AGTTTAAACCAATGCCTATAGATATATGTAGTTATATACACACATGAACATTTAAATGCAGTTA
ATTTAGCAAGACGGTAAATTAATTACTATCCGTCAAGTCTCAAGATAATGTTGGTGAATTTCTC
CTTCCCCTTAATTTCTTTATGGGATTTAAACTAGTTATTGCACATTCATCATATTAACTTTGGA
TTCAAATTTTGTTTGGAAACTGTATATTTTTTTTGTAAATAGAGTTATACTCACCTTGCTATTA
ATTGGCAACAAAATTGAACAATGCGTGAATAAACGAATTATATTAGCTTATATGATATGATATC
CTAATTCCATAATTAATTGTTTTATCTGTCAAGTACCCATGTTAGCCGCTTGCAATTTATAGTG
ATGGTTTAAGAACCTGAAATAGGGACAAGCATCCACATTCCATTTTTTTTGCTTGTCAAGGTAG
GGAGGGTAAAGAGAAAGAGAGACGATCAACACCTTAGTTCTCTCACTTACGCGAAATTGAATAG
TTTGAATTTGGGTGTTCGGTCGGAACACATTAATGGTACCCATACTAGAATCCGGTTTTTTTTT
GTTTCAATGGATTTACTTAAATCAATAAGTTTTTTTTTATTTTTTTTGATCGCACTTAAACTAT
GTGTTTATGGTAAGTGCAAAAGGGAAAGGAAAAAACCCCGACCAGTCCGACGAGCTATTGTCCG
AGTATTAGCAAGAAGTGCGAACCTCCAATCCCTATGCACCCGGTTAGGCAACAGCCACGGCCGT
TTGTTTTTATTTTTGGTAGATGGCCAAACGAAAGGCAAAAACTGAACCCGAACCTTCCCACCAT
TACAACTTCAGCTCCATATCCCGAAAGGAAAATGAGCCAATAAATTCTACAACAATTAAACCTC
ACAATAATTCATTTGATCTCATATTGATTCCTCATCTCAGTAAATTTTATATCGATCAATTCTA
AGAGCTGCAATAACTGAACTTGGCCTAGCCTCTAGCAGTTAATGTGATTTTTAATTTTCTATAC
AATTCTATTTTATACTCACACACACATACACATACACACATTCATCAGCATACACGCGATTTGA
GTTTATTATTTTCATTTTTGGGGGTGGTCTGTATCTAAAGGGTCGTGTCTTCGGGCTGTGCTGA
CTTTTACGAGCGCATCGCTCATACGCCGCGTGGGCGGTGGTGCGGCAATTTCTCGCGTGTGGCA
GGTAAAGTCTTAATTGAATTTGCTTTCCGAGCGTTGGCTGGGAAACACGAGTCGCTGAGCAGAG
CTACTCCCAGCTCCCAGCATTCGTATATATGAATATGCATGTACATATATATTACACATACGTC
ACGTATTTCTAAATGAAATTGTGTACATATTTTATTAAATTTTTGAGGGAATATTAACAAAAAG
GCAGCCACTTTAAGGGGCGGCAAGAAATAAGAGAACTTAAAAGCCAGAGCTTAATTTTCATTTA
TTGATAGCTTGAATGATATGACATTCATGCGTACCTGGCGTGGGACTTAAGTCCGCCCACTTTG
AGCAGCAAACTGTCCACTCAGCTATAAACAAGATTTGTTATGAATGAGAAAGCTCAACTCAACG
TTAATGTCAGAAAAAGGTATCGTGTCCAGCACCAAAATAGGAGAGCACCTAAGCGGCTTGAGTT
TGCCTTCTATATTGCCAACGAATTGGTCATTATTTGGACATGGTGACTCCCTTCCTATCGGCGT
CTAGTTGTGTGACATCTTTCGGGACATCGTATTTGCATTTTAGAATTGTCTCAGCGATTTTCAG
TTTATGACTCAAGGCAATAAAAAGGCTGGCATTCATTTTCATCCTTATTTGACAAGAGCACATT

GCGTGCTTTCCGGCAATTCATCATATCGCTGTGGGCTCCAAGATGCGTGCCTAACTTTCGCCTG
GGTTTTTCCAGTTGTCTCAAATTAACTTTAATAAACTATATGTATGCAATTTCTTCACAGCCTA
CCCGTCACTCAGTCTGTGCTGTGATTAATAGTTTGGGGCATGTTTGAGCATGTCAAAATGTAAA
AGTTATGCTGGGTTTTTGCCGAAAAAGTTGCGGTTGAACTACAAGAGAAATATTAAGTATTAAA
ATGCATATAATGTACACTCTATATAATGTGTGTTGTGCTTATTAATTTAAACAGTCTTTTATAT
ACACACATATACCCCGTAACTAGCAAAATGAAGGTATCTGCAACCCCAATAAACTTAAATATTC
TCTATCTGTTTGTCGGCCTGTTCGCCTGTAAGAATGTTTAGATTTTAGAGACCTAAAGAACTTA
AATTTTATAAGTGCACTTATTTATAGATTATATATGGAATATAAATATGTGTTAGACTTGATAT
TTATTTAAGCTCATGTAGCCCATTTAATGTACCAGGTCGTGATGGACAGACAGACAGACGGACG
GACAGATAGACGAGCATTCAGGGTATAATAATATTATTGTAGTTAAAAACAAAAACATAATATG
TATTTATATTTATATTGTCATATATATTTTTCTTTTAGTTTTTATTATTTCGCCCTCGAGTGTC
CCTACAATGCAGGTCATCAAATAGAGCAAAAGTTTAATAATTCAGAAGTCATACCAAATTAAAA
TGTTCAGTTTTATAACTTAATTTAATTACGATTTCATTTGCTTGTTTATTGTTGTTCTTGTTGT
TTTTGTGTTGTGGGCCAACTTGCTGTGCACACATACTTACTTGTTTGTTTTTCTTTTGGCCCTT
TCAGCCAATTTAATGCCAAGTTAATTTTTCAATTTACACGCAAAGTGTCAGAGCAGTTTTTCGA
GAAGGAGGAGGGGGGGGGGGGCCCCACTTGGGGCACGCTAGGGGCAAGAGCAACACGCATAGAA
GAATCATATAAAATTGCAAAGTGGAGTTAGTAGGAAAAACTTTGCTCGATAATAGCTAAAATAA
AATAAATAATAATAATACAAAATTATTATAAAGTGAAAAAAAAATATGTTAAAAATCAACAACA
ACAAAGGGACAATTAAGCCAATTAAGCTCTAGCTGCTGCATAATTTGTTAACAAAAAACCAAAG
CAACAGAAAGTTGAAGATTTTGCGGCAAATAGGAGCACGACCGACAAAAACGAAAAATATATGA
AATAAAAATGCAAATTTATTTTAATTTGTCCCAACCGCCGCCGACGCCGCCGCGGTGCACGTTG
AAAGCAAGGTGGGGGGTCTGCGCGCACGCTATTTGCATTTATTTGAAATGCATTTTGTGTTGGG
CCAACAAGTTGACAACAACTTTGCTGCCACTTGCCACCTGCCACTTGCCACTTGCCACCTGCCA
TGCTGCGCCGTAACGCGTCGTTGCCCAAACGCACGGAAAACTTTGGCTCGTCTACGTCTTGGAC
TTTGTCTGCGACTCCGACTGCGACTCTTGTTCTTGCAACTTGTAGGGCTGTTTTAGGTCCTTAG
CGGCACTGGCGCCTGGCGCTGGCAGGACCTCCTATCCACTCGAATCAACACAAAATTGCGCTCA
CTTAAATAAATTTATGAAAATATGCAAGCACGCGCCGGGCTCGAGTTAAGTGCAACCAATTGTC
GGTACAAGATTTAGGTCTGCGGCTGGACTCGGTCTGTAGTTTGGTGGTGGGTTCTAGCCGCATT
GAATTCAATTTTCAACAAGCTGTAGTTTAGTAGGAGGAGTAGAAAAAAAAACAGTGGGGTTAGA
GTCCGAGTAGGAGAGAATGAGCATGATAATGAGAAAGAGAGGTTCGCCGAGTTAGACAGAGAGA
GAGAGAGTGAGTGGGCGAGAATGTAAGAAGGCAAGAGAGTTATTAGAGTGGGGATATAAGAAGC
GGTAAAGCTTATCGGATTTCCGATACAACTTGCATGTATCAAGTTTGCAAGCCGGGTTTGAACC
GAATCGGCTTGAGGTCGAGCTTGTCTTCCTTACATTAACGAATAAGCGTACCTTGTACATACAG
TTATCACACTAAAAGTACGAGTATATATATATATATATATATATATAAATAGTGACGAATTTGT
GTATTTGTGAAAATGTATGCAACAGGCAGAAGGAAGCATATTCGACTCCATAAAGTATATATAT
ATTCTTGACCAGCATCAATAGCCTAGTCGATCTAGCCATGTCCGTCTGTCTGTCCGTCTGTCTG
TCCGTCCGACCGTCCGTCCGTCCGTATGTATGAACGCAAGGATCTCAAAACATATAAGAGCTAT
AGACTTGAAATTTTAGATGTAGGTGCTCCTAGTGCCTGCGCAGATCGAGTTTGTTTCCGATAAT
CGAAAACTTACTCCGTTTCCAAGCAATCGATTTGATGTTGAAAGAAGAGGGTTCAGGGTATCCC
CTAGTCGGGAGCTTCCAACTAGAACCTCTTACTTGTTGCATTATGCATCGATCATCAATCAATC
AAGTCAGACAGTCAGTAATATATAAATGTGCATATATGTATTCATATAACGTTATACTCTAGCT
CCTTGGAAATAAATTAGCTTCTGCTGCATACTTCTGCATACTATTACGTATATCTTTATTGCAT
TACTTCGTTTACTTTTGCAGCCCATTACTTATCCCTCCTATCGTTGACATAAAACCCAACTATT
AGTTAGCATCTAGTTGGAACGTCAAAACGTTTTACAATGCAAATTCTCCTGTCAACAGTCGGAG
GCTGAAACGCAAACAGTTTCACTTATAAACCTGCACTTTACTGGATGTTGCTTGCATATTATTT
TAATTAGCAACTGTCGCTCAAAGAAAATACCAAAAGCAACAACAACTTATGGCGACAGCAGCAG
CAGCAACAACAACAAAAACAACTCGAATAAAGTGTCGCCTGCTGGTCCAGCCCTTGGGGCGGGG
GTTGGGGTAGGCGGTGCGGCAGCCCTCTCGTTAGTTGAGAGCAACAGCAGCTGCTGCTGCTGTT
GGCATGCATTAAAACTTGTGCTGGACAAGTATTCCACACCAGCCCTGCCAGATACACACACATA
CACGCACACACACGCACACGCAAGCACTCAGGGCATGACTCGCGAAATGAGCGCTTAACAGGAA
GCAACGTTTTTACTTCTAATATCCTACTCCTAAAGGAAAGAAAATGGGGTATACTGATTTCTGT
TGAAGCTAGAGGCACCAAAATCTAGAATAATTAATTATTTGTGGGGGGTACTGGCTTCCAAATA
GGGCTTGTTGGCTTTTTGATGATCTTTTAAAGATCAAGCAAAACGAGCATTTTTACCCGTCAAT
TTCTCTCAATTTTAGATATCATCAGGTTTGCTTAGTCAGATTGCTGATGACTTGTTGAAAGATC
TAACCAAAAAAATTAGCGTCCCGCTCACACGCGAATCCACTGCAACGTTCATTTATTATTTTGT
ACCCTGTACTCAATTCGAAAAGTTGTAAGGGATAGAAAAGGGCATTAACGACCTCATAAAGTAT

ATATATTCTTTATCTTTGACGAGTGGAGTCGATCTACAAGCTCTTCGATATCGGAAGCTACAAA
TGCTCGAGAATTTATATTTAAAATATAAAATATGTGGCTCGTATATCTTACGCATACGTGTTTT
TTCCATAAAGTATACCTATTTTGAGATACGTCCATAATATTATTAATGAAAAAAAGAATATTGT
AAAAAGTTCTTTTAGCTTAGCGCATGCCCCACTACTTGTTTTATACTTTCTTGCTTTTACCTAT
CGCCTCATTTGGCTTGGTGTTTTTGCATGTCACTTAAACAGCACTACGCACAGTTCCCCGTCGC
ACACCCTTGCTTACCCGCCTGCCCATGCGCCGGTCTCTTGACAGAAAGGCATGCCGAGGCATTG
CGTGTTGTGGGAGCAGCGTGAATCCGGTCCTTAATTTAATTTACGAAAAACAACTCGCTTCCAA
ATACGAAATTGGTGTTTAAGATAAAACGCTTAATACATACTTTCGACGGTAAATTAAAGAAACT
AAAGCGTTTTTGATTACATATAATTATTTCAAAGTGTTTGACTAGAAGCTACCTTGTTCACTGG
GAAACGGCATCAAGTATACGTGCATTAAATGCGCATAAAAGTAGGTTATCGAAACTGTGACTGA
ATTTTAAAGTTTTTGTATATTATATATTATATAATATATTTTTACATCATAGCTTGGACGTCGC
TCCTGCTATTGAGATATTTAAGTACTTTCGATTGCTAGACGGACAAATGCATGCAGAAAACTTA
AATACTAGCCTAAAGATTCCGACTTTTGCCAACAATAAACTACAAAAGCAAGACAGTCCCATGC
AAAGCCTTAAGCCTGGGGAACTAGTTTGCGTAGAAACAGACAAGGCGAACATGGCTATATCGTC
TAAGCTGTTCATAATGATCAACAACACAGATATGTTATAAGTGATGTCTTTTTCCATAACTTTT
TAATCATTTCATGACAAAAATAAAATACACTCAAGGATATAAAGAAGATGAATGGTAAATCGAA
AGAGCTTTGACGAACCAAGGTTTGAGAAGAGTCTTGTTAAAATTAGGTTCACGTAAGGGAACCT
TAAGGCAATGCATTGGTTAAAAGCAGGTTTGAAAACTTAATTATAATGTCTGAGCAAATTTTCA
GCCTCTCCGCCATCTAATTTGCCCAGAAGCGACACTGGCACCCTGAAGGACGTCTGGTTGATCC
CGAAACTGGGCATAGGTTAAGCCCGAAAAAAAAACGCTTGCAACAATAGAATTCTTATAAGTAC
AAAACTCAAAAAAAGCCATCAGAATTTAAAAGGGGGGGTTAGTTTTTATAATACTTAACATTGA
CCCAAGACGGGTTCAAGTTGGCCTCGCCACATTAATTGGTAAACTTTGCCCAAGATCTAAACAA
CTCTTAAGGCTAAGCAAGTATTGGTGAAATGCCCAGTGTGGACAGTTTGGGGCTTGGGCTGTGG
CAGTGCAGTTTGTGGGGTATCATTTTGTATGGCTGCCAAATTTAGCTAACAATTTGTGGGCCAG
GGTCGGCCAAGAGGTAAATTGGCCACATACCAGTGTTAACCGAGTATGATTAGATTTTGCTTTT
AAGCGTTTTAGCAGTAGTTGTGGAGCTTGCGGGCCAGGCAACGGTGCTCTGTCATGGAGAATCC
AATCAGGTGGCTGTTGACAAATGGCTTATGGCACGTGCCTATGCCGCTGCTGTTACCCCCCCTC
CTCAACAGCCCCTTTACCGTAACAATAGGTGTAGGAAAACTGGAGTTTGCAAAACCATTTGCCA
CTTAAGGAAAAGTTTCAAATACAATTTTAACTAGAACAACAAGAGACAGCGCGCACGAAACTTT
CCGCGGGCCAAATGCAAAAAGTAAGCTGTGCCCATGGAATGGATGGAGGAAACGGAGGGAGATG
TCTTCAAGCTAGCGCTGGATGAGGGTTGGAGGGGCTGCCGCGAAAACAAATGAAAAATGTGCAA
CAAAAACAAAGAAAAGTCGAACAAAAAAACATGCTTGAAAATATGAGTTTCTGTGTATGCGAAG
AGTATAAATTAAGTTGATAAAGTTCACTTGACCCAAGTAACGATCCCAAGACAGGCCCGAGACC
TCACACTAGCCCTGGAGTCCGCCACGTCTTCAGTTTTATTTTTTAGCAGCAGCAACAATAAACA
ATACAATGTACAATGTAATTTTTCGCAAGGCCAGAACAAAACCAAATGAATAAAATAAATAAGA
AAACTGTCAGGTGCCAAGGCAGCGGTCAAGGTGGCACGCCCACAGCGCCCCACTCCCACAGCTA
AGCTGAATTAAAAGTTTTTAACACATATTTACGAGCAAGTGACAACCAAAAAAACAAAAAACAA
AAAAAAAAAAAAATCAACGAGCCAAATGCCACTAAAACTCTGAAAAACAAAAAACGTCGCGACG
TCTAGTCAAATTGCAAATGATTTGGCTGAAACCCAATCAGCGGGTGGCCAGTCGAGCAGCGGAC
GGGTGGGGGCTAGCCGGGTGTGTGGGCTGTCACTGTTAGTCTGACTCTGATGGGGGCCATTGAC
ACATGCCACGTGCCATGACACCACTTGCCACCAAAACGACTAAAAGGATCATTCTCTTTTTACA
CTTTTGGGAATCTGCGCCGTACTGGAAAAGTTTCCAAACGCTTTATTTTAAAAAAGAAATATCA
GAACGAGTTAACTCGACAGCTTTCAATTGATTAATCAAAAGCGATGCCTCGCAAACGCAGAAAA
TGCCGCAGGGGTAAGCACACAAGAGTAGGCTTTGGCTTTGCTAGCTTTGTCGCCTTTTTACTGC
TTATAATTCTCAAGTGGTTACAGTTTCGAGCTGAGTAAAAGGTCTTGCGAATCTAGAGTTGAAA
GCAAAGTCTGTCGTTAATACTTAGAACTGGGCGATTTTACACATAGTTCTTTAAACAATCTGTT
CAATCTTGGTACTTATACTACCTCAAGCCGGTCGGCTTTGGATTTGAAAAATTGCAGTTACATT
GTAACTACTTAGAATTTGACATCTTGACCATAACATTTATTTAATAAGAAACCGTGCTTTTTAA
CACTCTTCCCAGAGTAAAGAAGAGGACAGCAAGTATATCTATCTGTCCTTCGGATACTTGTTTG
AAAAGTAGATACAAATCTGGTAGAAATCTATATCATATTCAACTTTTATGTATCAGTCACGGCC
TGTAAAACGCAAAGAATGAGAAGTCTGCGGTTCGAAAGTATAAACATTTTTGACCAGTATCAGC
AACAGCCGAGTTGTTATAACCATGCCTGTTTGTCTGTTTTTGTGTAGGCTAATCACTCAAAAAG
TTGTGTTCATCAGCATATCTTCACCAAATTTGGCATTAACTAGATCAACCACGAAAAATCACTT
GCTCAATAGAAAGAGTCAGTTGATTTTGCAGATATGTTAATAAGTATAACAGCATAGCTGCAAA
TCCGTTTCCGTTTCAAATCAGGCTTCTGTATAATGTAACGCTTTTTATGTCATATGTGTATAAA
AGATACAATTATAATTGTTACTATTATTCAGTTTTATTTATATTATTATTAATATATTTTATAT

TATGATCTGTATTATTCTTATTATTATTATTATTAGCATGGGAGTAAAGCAAACTATCTATCGT
ACTCCGGGAAGGACAATACTATATTTATAGACTGACGTGGAGTGGAACATCGTTGCACAGTGTT
TTCCATATTATTCGGCAATATATAGTTGAAATTACTTTGTGGAACACGTTTTCAGAACGCACTC
ACAAACTATTTGCGGATGTCCGGTGCTTGGCAACACGGCTAGTTTTACATTCGACGAGTAGTCG
GCTTTTGTTAGTGAAAGTCATCATGTACGCGGTGGCGTATGCGTAATGCGTTTAGAGTTAATTA
AATTTGGTCATGTTTTGCGTGTGTGTGGTATTGGTGGAGAGGGATCCGAGAGGGAATGGGAATG
GGGGCTTGGCAAGTGTCCAAGCGGAAAGCCAATTCATCATGGCACGCAGCTAAAGTCGTAATCT
ACTTTAACAACTAATTGCCATTACTAAAATTATCATGATTATGATGATGATTAAGATTATGATT
ATTCAGTATGGACATCCTTTTCAGTAGGCAAAAACTTTTGGCTAATGGCCTGCCACAGTGTAAA
CTTATTCCTGTTGCCATTTCGCTGAGGCCTAGATTTTGGCTTTGGCTTTCGCTTTCGCTTTGGC
AAACCAAAGCACTTTCCCTACAATATTTTACAACTAATTAGGGATAATTACCAGCCATTACACA
AAAGGCTTCCCAGGACGGTGGCAAGTTGAAAGTTGAAGACAACTGACTCACTGACTGACAGGCT
GACACATTTTTGGGGCGTGGCAGTGTCTTTTGTTCAGCCTCTGTAATTTGGGCCACCTTAGTTA
TTATTACTGAGCCGTCGGCATACAAGCCGTCAAACATCCCCCAAAAATCCTTTGACTAACTCCA
TTGAGTAATACTCATTTGTCTGTTGATACAGGAGCAAATGTCAAAATGTCAGCTATCATATCTT
GTTCAAAAATAGTTAGTTGGAAACAAGCGTCTAGGGTTGTCATTTATTTATGTCTGCATTTTCT
GATTATACTCAACAGGTCGAATAAATGTCATACTTATGTTTATATTTTCGTAAAACTCCCTAAT
TCTACGTAAATACAGTTTGTAACCATACTCTGTGCCCAAGAGAAGCAGAGTTTATTGCTCGTAG
GCAATGTCCATCTTGGATCTCTCCCTCTAACTATGTGAACAACTTTTATAAATATAGGCGAGTG
CATACTGCGAGGCTAGGAAACAGGGTATCTAATTGTCAAACACTCTCAAGCTGGAACCGATCAT
ACTGATTTTCCAAATAGCACTAGCTATCTTCTGGTAGGACAATGCTACACCAGGCATTCAAACT
CAGATAAACTAAAGATACAATTATCTCATAACTAAGTACATATATCATCAGATTGGATAAAGTG
TTGCTGCAGTATGTGATTTTCACACAATGAAGAGCCAGCTAAGGATTATGGCATGTCGCCTGGG
AGGTGATCAAATCCGATTTACAGTGACACAAACAGCTTCAATAGCTTAACAAAATAACAATAGC
AACAATAATAGTACATGTGCATGGGTGCACTGGCAGCTTGGAGTTAGGAACTTGACTGTTCTAA
AGCGCTGAAATGCACTTCCCTTGAGTTTTTCGCATGCTCAGACACTAAGACGCAAATAAGTTAC
TGTGTGGGCGTGTGAGTATGTATGTGTGTATGTAGCTGCCATGTTTTTAATCCACTTGAAGGCA
CTTAAGTAAGCTTTTCTTCAGAGCTGCCTGGTTTGTGTGTTTGTGCCTGGTTGCATGTGTACCA
AGTGCTTTTGGCTTGCTTTAAATTCATAGTGCTTATCAGTAAATACAAGAGTGCTCGAATGGAA
GATACTCTATACTTAACTGCAGCCATCTGGCTAGAATGAAAACAAATTTCGCTCACCACAAATC
AAGTTTACATGCCCTCATATAGGGTATTACAACACTCTATGCAATGTGTAACGCATGAAAGAAT
ATATATAAATAACCGTAAGCCATGATTCTACAAGGGTGTTTCACTGTCAGCCTGCCAAATATAG
CCGCTCTTTCTATTTTTGACTGTCACTCTTTGGACATTTGATCTTCCTGTCTATACACAATATT
TAATACTGTTTGTGTTGACTGAAAGCTAAATTCAAAACAATAGTTGTCTTTCTGATTCAATTTA
AGCTTGGCAAATGAGAAAGGCATTTTGTGAAATGAAATTGAAATTTGGCATGTCTTACCGAATA
TTGATAGTCCACCTCCATATAATATCTATGTTTGATTCAACCAAAGTTAAATTTGCGTTGCATG
AACCTTAAACATATTTTTTATGCACAGGCACAAACAAAAATCCAAATCCTCTCTCCAGGTGGCA
AAAACCTGTGGTTGTCATGCTTTCCGGATAATTCAGTGCAAGCTAATTTAGAAGCAGTCTTGAG
AAAACATTTCATATTCTTCTTTCTTTTCCAGACAGATCATATCTATGTAGAAAACG~~GCCAAATC~~
~~GAACCAATATATTGTATAGCTGCCATTCGAGCAAAACCAAACTGATCTGAGTTGTACAAAGAAC~~
~~ACTAAAAACCTCTGAGAAAACCTATTGATGCTGTTTTGACGAGATTTTGGAAAAAAGTGATGTG~~

***Xb* inversion:**

***D. novamexicana* 15010-1031.00**

**Distal breakpoint**

>Nova00_Contig3651: …9023..7108… (*GJ14731*[-](*CG8206*) – *GJ18832*[+](*Crag*))

~~TTCAATTTGAGTTACTCATAGCTGGTTTGACCGGCGATAACAAATTATATTTAGCCGTCGGCTC~~
~~TCATATATGTACATAACAAAACAAAAAAAAAGAAGCTAAAATTGTTGTTTAGGTAGGTTGTTT~~
~~CTCCCCTCTCTCTCTCTCACTTTTCTGTCTCCTTTTCGTTTGTTCCGAGTGTTGACACGACC~~
~~ACACTGCATCGCTGTCCTGGCTGGAACTGGAACTA~~GATTAGTTCACCGTGCTCGTTCACTTTGA
TGATGCGCACTCGCTCCTGTCGCTTCGATAGCACCTCATCGATATTCATGATGCTGCCATCAGT
ATTGGCGAACAGGCCCAGATTGTCCTTGATCAGATGATCGACGGCACAGCCATCGCACTGGAGT
ATGACCACGCCGCTGTAGAAGGCCTCCTCGCTGATGGTCTTCGTGTTGCGCGTGTTGCACAGCT
TGCACAGGTAGACGATCTCCATGTGGCGCTGCATGCGACGGAACCGCTTCAGGGTTGTCGGCGA
CAATGAACCCTTTGGCTTAGCCCCAGCGGCATTGCCGGTTATCTCAAACGGTTTGGCCGTAAAG
CGGCAGGCGTCGAGTATGGAGCGCGGTATCGAGAGCGCCGTTGATCGTTGGGCATCCTTCAGCG
ATTTGTCCGCATCGGCATCGGACAACGGCACTGGCGCTGGTTGTTGGGCTGCATTGAGGATCGC
GTTCTGTCCATTCTGGCGTAGATAGTTGAGGGTACGCGATGTAAAAATATTGCGCAATGCATTC
AT~~TTTACATCGGCATATTCACGATTATAATGCATACGGATGACGATATCGAGTCGCGGCGCTTT~~
~~ATGCTACACCAAAATACTGCCGAACTGCACGTCTGTATGATCTGGTTGTTACTAAACTGTTTTT~~
~~AGCATAAACAAACGGCGCAATTTGCTCCAGCGAGACCGACCACGAAGGCAACACTATC~~AAAAGC
CGACTTGCGAACCAAGTGATGGCACTATCGATAGTAACGATAATTAAGTAATGCATACAGTGGT
GAAAATTGTGCAGAAGTTTGGGTAATGCTTCGTTTGTATTACTCTACAAATGTCAACTACAAAT
AAGTCGGGTGTGGTGTCTATTCTACTTAGTTTTTTCTCAAGACTCCATTTCCGGCGGTGAAACT
TAACTTCATAGCGTATTTTATTAACTATAAGCAAATGTTGCCGATAGCCCAGAGGCAGCACGAT
TGTTGAGTGTCGTGCAGCAACGATACTACTTTACGATGAATTTCTGATCGTTTTTAACTATCGC
GAATGCTTTGAGATGAGCGAATAAGAGCTGCTAACTATCGATAATGCATCATTAAAAATATCGT
ATTTTCGATTTGGCGGAAGCTATTGTGTATTTAAAGTGTGAAACTAAAATACCAAAACTACTAT
TTGCATTTGTTATATATATTTTTCAATAATTAAATCTAAATGTAATTGGAAATTAAATTGAATG
TTTTGTAATCAATGAAATCCATTTAATGTTGTTGTATAATAAATTTCAAAAATAAAATTATTCT
TACGTTGATGCAATCGTATTTTATGGGTGTTAACAATACAACAACAACACAACTGTAATAATTA
ATGAAATTGTACTTTGGAGGTTAATTTATGGTTGCACAAAACTTACAAGTTGAATTTGTTTCGC
TGGATATGGGTTTCAGGAGTCTCACTATTAAAAAATA~~ACATTAATAATAATAATCGATACATAT~~
~~ACTGTAATATCGAAATTGCATGTAAACGATGTGCCGATAAAACAACAACTCTAGCTTTCAATTGA~~
~~TTACGATTTCGCATGCATTAAACGGCTGCCGACGTAACAAATTGGACAAAAGACGCCCGATAGTT~~
~~AAGGCACAAAGTCAAGACAAAGTGCAAGTGGCCGAATGTCGCCCGCCTACATATCGATAT~~

**Proximal breakpoint**

>Nova00 Contig4878:…1545..1 + Contig2631:17151..1 + Contig6712:1..4134 + Contig15014:1..536 + Contig3226:13717..1 + Contig691:1..8756… (*GJ14858*[-](*CG15896*) – *GJ19325*[+](*Asta-R1*))

Note: the region in between the dark blue nucleotides corresponds to an overlap of 157nt between the beginning of Contig4878 and the end of Contig 2631 (these contigs didn't grow most likely because this overlap corresponds to an ITR)

AGATTGTTGGTGAGGAAAACACTGGCATTGTTTTGTATATACTGCATGGCTGGCCTGGACCAGT
TGCGCATGTGCTGGCGACCCAGGACGAGCACGCGCTTGCGTCGCTCCTTGAAGTAACGCACCAC
GGTGGCCAGCAGTTTGGCCAGCTGCTGGGGCGGCTTCTTGTTACCCGTGGAGTAGGCCACATTG
AGGCCATCGATGACGCAATCGTAGGGCGCAGTTTGCTCCACATACTGCTTGAAGCGTGCCACCT

CCTGCGGCGTAGACTTTTGGAACACGTCGTTGCGTATGAGCACCTTGGCCAGAAAGGATTCGCG
CAGCTCGGCGAACTGTGCATCGTTGATGGCCACATGCTGCAAGTGCTGCTGGCAGGCGCCACAT
TTGCCCTTGCGATCCAGTCGTGTGGTTGTCGCCTGCACTTGCTGCGGCAGCCGCTGCGACAGCG
CCAACAGCTGCTGCGCGACCAAATCGCTGATGACAATGTCGTGACGTTCCAGAAAGCGCAGCAG
TCGCTCCAGCTGTGCACCTAGCGTCTGCACCTGCTGTGCACTTTGCGCCAAGTGCGCCAGATAC
ACCTCACACTTGGGCAGCTTGCGCGCCTGCAGCATCTCCTCGAGCAGGCGCCAGGCCAGCTCCG
GCTGTTCCGCGGAAAATGCCTTGGCTGCCAGCGTACTGTAGGCTGCTACGCTGGGTGCACTGGT
CACCTTCATCATCTCCAGCAGTGGCACAGCGCGCTGCCAGTGCTGCTCTGTTGCCACCAGGCCG
TGTATCAAATGCTCACAGCTGCTGGCATCCAAAATCTCGTGGGCGCCCTGCAGCGTATCACAGA
TGTGCACAATTTCCGCCTGCTCCTCGTCGCTGAGCGTGCGTGCTTGGTAGGCGGCGTTATAAAC
GCGCAGCAGACGACCCAATGTCGCTGCATTGGGCTTAATGCCCTGCGCCTGCAGATAGCTCAGA
TAGCTTTTGGCCAGCGGCAGCTTCTCCGCACTGCTGCACATGCCCAGTACGACGGCGTCCACGT
TGCTGCCATTGATGTATTTGTAGCTGTCGGTCAGTGTGCTTCGCACCCGATGCCAATCGTCGTT
GCTCAGCTCGCTGTGCCGTTCAAAATAGTCAGTCTTCAGCTGATCCAGCTGATCCGAAGGCACT
GCACCCAGTTGGGGCCTTCGCTTGTGATGACTGGCCAGCAGACGGTATGGCGGTGATTGTGTCA
GTGCTGTCCCAACGCTCTTTGTGATCACACTGTGGCGCAGTTGACGCAGTATACGCAAATTGTA
CAT~~TATTCAGTTTGTTGTCAGTTACACTTACTTGTTGCCGGTTCGGCAA~~TCGAAAGACTATCGA
TAAGCACATTTTACAAACTATCGAGTAGCAAGGCACATATGCTAACGTGCGTCAAATTAGCGGC
GGTGACTTAGCGTCGAATTTTGGTACTTGTTATCGGAAAACTATCGGTCACTTTCAAATGCATT
TATTTATGTTTTTAAGCACGAAACCAAATAAATTTAAATGCAGAATTGCAAAAAATTAAATTAT
TTACTATCTAGATAGTAAATAATTTAATTTTTTGCAATTCTGCATTTAAATTTATTTGGTTTCG
TGCTTAAAAACATAAATAAATGCATTTGAAAGTGACCGATAGTTTTCCGATAACAAGTACCAAA
ATTCGACGCTAAGTCACCGCCGCTAATTTGACGCACGTCATATGCTACCTCACTTTGTTATCGT
TTTCAACACTCAATGGAAAATCGAATAGCCGATATTTGTGTTTGCCTCCATGATAAGATTACAG
TTAGCCTTTTTTTAAGAAAAGCTGGTATTCGCTGGCTTTGACATTTTCCAATCCATTTATCAT
ATTGAATTTAAGAAACTCATATTGATAATCTGAACAAGAATAGCTTCAAATATAGGTGTTGATT
ATAATGAATCGTTATCGATAGTTAGCAGCTATTATTTGCTCGAAGCTCGAAGAGCTGACGATAG
TCGAAAACTATCAGATATCGGTGCTGCACGACATTCGGCAATCGTGCTGCCAATGTGTTATTAG
GGTCTTCTAATTGCTTATATAAAATAATTTCGATTGCTTACCTGTTTTGAAATATGCAAAGCTT
CGCGGCTGGATATGAAATCTTTTTAAGAACGTAGAAGAATAGACACCAACCCGACTCGTTTGGA
CGTTCTACTTTATCTAGGATAACCAAAGCAAAGAAATATCAAATATACAATTACCGTTATACCG
TTGGGCGGCACAAGTATTGATATGAAAGTTATCGTAGTCGGTTGAGCGCCCGCAATTTCGCATT
TTAAATTTGGTAGCAACCTTTTCTCTTCTATAACACTTAAGACTTTTGTGTTTTGTGTACTATT
TTATTGCAATATTAAGAGACAAATCGGCTACTTTTAACAGCGACCCAATTAGTGCATTGCAATT
GTATTCAAAGTTCTTTGCTCTGTGGCAAAGCCAAGCGGAGCGTAACCGGCCCCAACAATGGCGA
CGCATGCATTAGACCTAATTATACTTTTTATGGACTTAAGCCAAGCTGCTGCTGTCTATTATTC
CCAATTTACAATGGCCCAGTTGGTGGCGACACTTGTCCATTGAGCCAAGCTGCTCAGTTTCATT
CATAACGGCAGTTGGCTCGAGTTTAAACTCAAGATGAGCGCATTGTTGACTGCGACTCGCGACT
CAAATTGTGCGCAGCCAGTGACGCCTTAAAGGGGAGAGTTCCTGTTGCCTGCTGTTGACCCCCC
CTGAGACAAGGACTCTGCTGCGCAGCTTGACTCGGTTTTGTTTACAAATATGCCATCGTGCTTG
CCGCTCGTACCGTAGTCCTTTGAGATTTGTATGCGCGCCTGTTGCGTGCGCCCGTTGGACGTTG
TCCTAATGGACTCTGCTGTGGCCTCAATTGCTCTGGTTGCCCCGATAGCCTGGCTGCTATCTGT
TTCTGTTTGTGATAATCTAATTATGCGGTCTTCCCGAGCTGGTTATCAGCTGTGCCATTGCCAT
TTTATTTACTTCTAATCAACTGGACAGCCCGTTGTCCTTCGTCCGTGCTCCGGCTGGCCATTAC
AAAATAATAATATAACAAAGATTTATTCACAAATACCATAATAAAAATCGCAGCCAAACAATTA
ACTAAGCCATTTAGGGCAACAAATCAGATTAATTAAGAACAATAGCCAACATTCAAATGCCAAA
TGGCATTTTGCGTTTGCTATTTGCGACACTTGTCAAAAATGGTCTATGAAATTGTATGTCTCGA
CTCAGGGATACTCTGTAATCACCTTGCTTTCAAAACTAATATACAAAATTGGGGACGCTTATCT
TATATTTATTGATTTAATATTTTTTATTAAGTTAACTAACGCTAGAATTTTCAATGTTTCTAAT
ACTTCTTCGTTGCAATGCTTACAAAATTTGTTCTGCGTTGAGCAGGAAAAATGTACAGCTCTTT
TTTTTTATTATTAATTATTTTGAAAATATTTATAAGATTCAAAGTTCTTAGCTATAAAATGATT
TGAAATATTACAATTGAAAAAAACTTTTTTCGAAAGCCATAGATTGATCAACCTTCTAAAAATA
TATAGAGTGCATGAACATTTTTTTATTATTTTCTTTTTATTATTTGGCATTTTGTGTCTGTTTG
AATATGAGCTCGTTCAAGGGATTGGAGTTAACTGTAAAACTTACCAAACTTAGATTCTATATAA
ACTATATTAAAGCTTTTTAGCAAAGGACATGTGTTAACATAATTAATAACTATAAGTTAATTTA
TACACAAAGCAAATGTTTAAGAAAAATAATAAAATAAATTGTATTTAATAATATTTTTATTTA

TTTTCCCATTACTCAGAGCCTTATATATATCATGACTGATTATGATGTTGGAAATGAGTATAAT
AAAAAACATTGAATCTTTAATTGTTTAGTGGAAAAAGTTCTAAAATAGATGAAAGAGAAAAATA
GAAACAGTTTTATGCGTTATATTCCTTGCAAAAGGGTGAAAGCAATTTGTTTGAGGTTTTTCAT
ACATACATACATTATTGTAAGTATTTCAGAGAACAATTTTCACTGCCAATTTTTTTTTCTTTCGC
ATCATGCTTAATCTTAATTCATTTGGTTTGCTTTTCGTGTTCCATGTCTCAGGCGACAATGTCA
CTCAGGACATTTGAGTGGGCGGCCGTTGCGAAAGCGCTCAGAGTGAAATGGGGAAAATAACTCA
TACGCCGTGTCGGCCGTCGTTGGATGCGTGTTCGGGTTTCAGTTCGTTGATTCTTTGGCTCTGG
CGATACTGCTACGGGGCGCTAGGATTGTGATTTGAATTGTAGAAAGCGCGCAGTTCGCATGCGA
ATGTCCTGACCGACGGTTGTCTGGCCGGGTTTCAGCCCAGCATCCGAGCAACCGCATCGTTTGA
AAAGAATCTTCGAAAATTGTTTTTGAAAATGTAAATTGGCCAAGTAGCCACGAGTACTGAAAAC
AATCAAATATAATAATATTCCTAAATACATAATTGTATCTGTGAAAGTTTTTCCTAATTCTTTA
TTTTTTTTTTTGGTGCTGTAAAAAACCCTCATCAACAAAAAACGGCAACAAAATCCAAAGTGAA
GAAATAACAAGAAATTGCCATAAAGGTTATTCAATTTTATAAAAGAATCTTGAGCGTGGCAAAC
TTCCGATTTGTGCTGATTTTCGGCTCTGCGTCTATATTCAAATCTAAGCAGGTGAGTGCACCGC
ATAATTACCGGAAATTTAATTATGAGGTGAGGCGTCGACTTTGTAAACGGTAATTGCATTACCT
AGTCAACCGCGCTACCGCTTCTCAGCCCGAAATCATAAACAAGTAATACGAAAATGTTGCTGTC
AGAAACTCCTTACTTCCATTTACAGAGTCTCTGCTTTAAAATAAACACGTCAACTCTCCATAAG
AAAGTTTAGAGTTTCGAGCATGAGAAGTGTCTATGCACTTATTTTTACTTTGAATACAGTGTGA
AAGAATACCTTTCGAGTTCTCGAATTACATTCATTCGTCGTTAGTTCGATTCAAATTAAATTTT
GCGGTTACCCCAATCAAAGGCGATTTGTAAGCAACTCAAAAGGTTGAGCAATGTTTCATTTCTT
TTTCCCTTTTTTTTTTTCTGTGTGTGTTCCTAATTCAATTAGGTGCCCACCAATTTGTACCGCT
TACATTTGAGTTAGGGGCAAGGGAGCTGCAGGACCTCTCGCCGGACGAAGAGCTTCCCGTTGTG
GGCGTAGACAATATCAAAGACAGGAGCAGAGCTATTTAAATTCGCAGACTGAATTAAGCTCATT
AGCATATTGTCGCCGGTCGGTCATCAAGTTCACGGGCAGATGTTACAATTTCCCATTTTCCAGT
TGCCAAGTTTTCCACTTGATTGATGAGTCCAGTTACAGACCAAAACTGCCGCCGCCTCATACTC
ATACTCGGCGTCACGTCTACAAATTTACCTGCTGCTCTCGCTGCAGAGCTAGAGAAATGATACT
CATTAAACAATAAGATGAGTTTGCCGTTTGTCCAGCGCCAATCAAATCGCTTGTACTATATTTT
CTTTGTTCGGGTTTCAGAGCATTTTAAAACAAAACATTTGTGACCAAGCAAACATTTGTATTGT
ATTTATTGTTGAAGTTGTGTGTCGAAAACTAGTTCGAAACGGGAACCTTAAACCAGGGCAGTTG
TCCCAAAAACTGCTGGAACGAATCATAGTGAGGCAGACTTAGCCCAACATATTTGGACCCGTAA
TACTATGCAACTATATGTTGTATATTTTGTAGATGTCAGTCTTGGGTAGAGATCAAATTTAGCC
AGCAACTTCTACATGAAGTTATATTGTCAAAGTTAAATAAAAAGTTTAATTGTATAATTCAAAT
ATGAAATATATATGACAAGCTTAGGCTTAAGCCAAAACCAGGACTGAAGAATGTGTATAGCCGG
GGTAATGAACCGAAGCTTAAGGCTGGGAATTGATTCGAACCATAAACCGAACCCGTTTTTTTTG
GGGCTGCAGACCTGTTAAATAATTTGAAAGTGAATCAAAACTGTTTGTACCAACTGACTGAGTG
ACAAATTGATCAAAAGTAAGTAACTACACTTTAGTTTTTGAAAATTGCTAAAAATGTATAATGC
GATTTTTGTGATGAATATATAAGAAAAGTCTACATTTTGCCGATAGCTTACGTCCCACTTTAAT
GTTTCGCCAAAAACCTATTTTGTTTTCAATTATTTCTTGAAAATAAGTTCAAAAGTTACCGATT
TTCAGCCAATCGAACCACTGTGCATAGCTGGACAGTTTCTTTGTGGCACACTCTTTGGCTCATG
TGGTGTTAGGAAGGATTTTTGATATGCTCCAACCGCAAGTGCGGTAAATGATGGCAAAAGGTTG
CAGCAACGGGCTCGAACTCATGTTCAATGGGGATCAAGGTAGTAAATTAAAGTTTTTCCTCGGG
CGTAAAATTGATTGCAATGAGATGGAAACTGGCAAATACTCGCTAGCTTAATCAACTTCGTATT
ATGATAATCTATAATTTTACTGCTCAAAAGAATAAAGTAATTCTCGTGAAATGCTTGAAAATCA
ACCTGACGTCGGCTTGATAATAACGGTTAGCTTGAGAATTTCAAAACTAGACCAGCAAAGTAGT
TATCCAAGGTTGCGTTTCAAAGCAAGCTCAGGCAATTTCAGAGAATATAAACTTCAAGTTAAAC
TGATTCTGGGTAAGAACTTTAAGCATTCTCCCTTTCTTATGCGCTTAACATACAACAAAGTAAT
ATATTTCAGTAGTTATGATAAAATGTGAGTAAGCGCGACTTGTTTCTACACATTTGGAATTGCA
ATCTTTGGAAACTAGGGTGAGCCAACCTTGAATATTATATTTTTGGAGTAAATTTCTCACTTTC
GCCTTTGACACGCGATTTTGTTTCATATAATGAAGAAAAGCTGAGTAGCATGCGACCTGCATTT
AATTTCAATTCCGACTTAATTTGCATTTAATGTGAGGACTTTTCGTCGAAATTTAATTGGCCTA
ATTAGGTGCGACATGGCCGTAAATGTAACGTGAACATTCACGCATTAAGTGATTAAATGAAGAC
TGTGACCCATGAAGCGAAGCAACTGAAACGAACTGAAGTGTAGTGAAACAAATGCGAGATATGA
GAATTGCACATTGAATTAATGAGGCAAATGTCAAAATTGAATGGGTTCAAAAATGTCTAACAAG
CGCGACAACAAAGACAAAGTTGCTCCAAGGGAGCCGGGGGTGTCCTGAAGAAGGATGCTCGTAC
TGGTTAAATGATTGAGCTAGCCGCAGTCTTTAAAGTTTGTCCGAATGCTATGAAAGAATGCAAT
TTTTATCAACTTTTCCATAATGTTTTAGCAAGAATTTAACGCTTCAGGCAAGGCAAAGCCAGCA

TACCCCCATTATAAAAAAAAAGCAAAAACAAAAACACAACCAACCTTTTACACTAAGGCAAACA
GACCAAGATAAGGCATTAAAAACGAACAGCATAGGCTTGTACGAGCTGCTGATGCCCTGTAGCT
TGGCATGCCGAAATATCTGTTATGGCTGTGGGCGTGACTCAAGTGTCATAGCTAATACACCTGT
CTTTGGCAGCCTTATAGGGTATGCGTATGAAAAAGCGTCGCAAAGCGTTGACTTCTCTTGAGTC
TTGCATCACTTTGGAAATAGGAACGGAACAGAACGGAACCGTAAAAATGCCATAACGCACAGCC
AAAGCCAGTTGGAGCATGGGCACAGGTGGGCGTGGCATGCTCGGCTCATCTGCTGGCTGCCGAG
CAAGCCACAACCCGTTCCAACCCATTGTCTATCTCTTCCGCCACGGCCTTCCTGTTAGCCCAGG
CAGTCACAATGTTTTGCTTATTGTCCTGCTGTTAGTTGGCATGGCAAGTGGTGTGTGTCCTCCC
AGCAATTGCAGTTAGATAAGCCAAGCCAGGCCCTGCACCCACCCCCAGCCCCCTAGTCGACACC
CTGCGACCCCTTTGTGGGATTTTCGCTCTCAGCTAAGCAGCTCAGCCAACAAGATGCTGCATAC
ATTGTGGCGCCAGTTAAAACAAATTTATATCTACGATATACATCTCAAATGTACGAGCACAAAG
AATTCCTGCGATTGCACTGAAACTTTGCACTGACATGTGGCTTGGTCAAGAATCTGCATCTTGG
CAATTTTAGGAGCAAAAAAAAAGGTATCTCAATTTTGGTGCGTAATAAGCTACATGTTTTATGT
CCTCGCAGTAGTTCAGACTTCGTACAAAAGTTTCCTTCTTCTTTTGCCTTCGCTTTGAGCTGCA
ATGGTAAGAATTTTACGAAAATGAAAAAGTTATATATGTACTGTTTCTTTAAGGTCAACGAGCT
GGTTAACATTTTAGCCAATAATCAGTTTTTTAATTATCCTTATTGACCAGCTTGCAACAAATTT
GAGTTGACCGAAGACTATAAGGAGAATGTTTAATCTGATATCAAATCAAATATAGTTGAATTCG
AATTTTCCTTAAATACGTAAATATCATACATTTTCTACAAGTTATATATATTTTGTAAAGGCTC
CCATATCCTAAAAGCCTTCTAACAACAAGAATATAACGGTGAATGAAGAGCCTAACAAGCAACT
AATTTTTACATATATTGTAATGACAAAATTATTCATTATCATTATTGGTCTATTAGTCCATTTC
AAACAAGTAAGAGGTTCCTGAACCCTCTTCTTCCAACCTCAAATGCAAATATATATTCTATTAT
AGAAGCTACATGTCAAGTTTGGTGACTCTAGATCTTATTATTTACCAAAATTGCTCAAAAAACA
GGATATCGATATCGATTTTTATCGATTACTTGAAAACGGGGCAAGTTATCGATTATCGGAAACA
ATCTCGACTTGCGCAGGCACTAGGAGAACCTTCATCTAAAATTTCAAGTCTCTAGCTCTTCTAG
GTTCTGAGATCCTTGCGTTTATTCATACGGACAGACGGACGGACATGGCTAGGTCGACTCGGCT
ATTGATGCTGATCAAAAATATATATATCCCTCTGCCTGTCACATACATTTGCATTTTGCACAAA
TACAATATACCCTTTTTACCCATTTTCAATGGGTTTAGGGTATAAAAATATGTTAAGAGTATTT
ACGGACGTTCGATAATAATATTTATTATATTAAGCAATGATAATTGTAAATTTCAAGCATAACT
GTTTATTTTTTCTTAAAACAGTTGCGTTAAAATTGACTGATGTTTTCCAGCAATACCGATCTAT
AATGCTCACATGTGATGCCATAAAAAATGATGTTTTATTGTTTCTTATACAAATTGTTCTTTGC
TGACTGCTCAGATAAAAATCTGGTGCAAGTAAGAACGTCGCATCTAACCGACTTCCGGATACTC
TGCTTACAATGTAATAAGAGCTCAAGACAGCTCAAATAGAGTCTGTTTAGTTGTCAATGCAAGA
CTGTGTTTTTTGAGCTGTGACTATAGAAAGAGTTGAGGTGTGGCAAATAATATTATTAATATTA
TTATATTTATCATTATTATTAGTTAATTATACGTTTAGGATATACTTATCTACAAATCTATCCA
CAATAGCTTTCGTCTATATAAATTTCAAATATTTAGCTGCAAAATAATTTATTGGAACAGACGG
ACATTGCCCAAAACAAATATGCTTATTTCTCTTTGTAACCAGAGTATAAATATATGTATAAAAT
AAAATGATGAAAAAAAAACACAAGGTAAATACGTTAAAAATTTGTATGCACCCTCGCACCAACA
CACACACACACACACACCCACACACACATCCCTAGTCAAAGCTTATGCATATCGTAAAAAATTT
ATAAAATTTTGCCAGCGGGCGGTTTCCAAGCCTGCTGTGACTCCACTGTGTGTGGGTGTGTGTG
TGTGGGTGTGTGTGTGTGTGTGTGCGTGTGTGTTTAAATATAATTGATTGACATTATTTAAGCG
TAAAGACAAAAAATTTACTAGTAAATAAATGATATGCAAATTGTTTTTGCACACACAGCGTGCC
TGGTATGTGTTCCCAAAAACAATAACTCCAACTGTTTTTGCCTAGACGTGGCTTAGTTTCGATA
TTTGATATACCGAATTCGGACTTTAATTGAAATTCGCAGCGGCTTTTAACAAATTTTTCATGCA
AATGCATTCATGAAATATTCGCCAATTGTGCATTTTATACAAATTTATATTTCAGGAGCCGGCT
CAAATAAATTCTATTTGCATATTACCTTAAAATAAAAGTATTTTATTTATTTTTTGATACCCTT
AAATAAAGCCAGAACTGCTCAAATAATCGAACCAAATAGTTTTGAGCTACATAAGGAAGATACT
CGCACCCGCGAAATTGTTGAGAATCGGCCGCTGGGAATGGCATACGTTTATGTTGATGTTCATT
TCAATTGTTCAAGTGCTTATTGCTGTTGTTATGGTTGTTTTTTATGGGTAAATTAAATATGAAT
TGTCCTTTGCCAGCCTACAGGATGCCTATGCTCTCGATTGCCTCACAATTAGTTAATTGACTTA
AAATTGTTCATTTCGTTGTTGTTGCCGTTGGTGTTGCTGCTGCTTGTCGAGATGAAAGTAAAGT
TCCATTTCTCAGAAGCAACACGCACAAACAGATAAACATGCCAAGAGACTTCTCGAGAGAGACA
GAGAGAAAGGCAGCAAATAGTGAGAGAGACAGGGACAGAGAGTGCATGGGAAGCAAATGTGGTG
CAAAGTCACTTTGAAGTTCCCCGCACCCGCTCCAACCACAAGTCGTTGTGAGGTATGGCGCAGG
GGCAGTGGGTGACAAGCTGGGTACGGGGGGGAGGGGGTTCGGGGCGGCACGTCGGGGCTTGCCT
GCCTGTGGCAATTGCTGCTGCATGCTAATCGAGTCAAAATGTGCCCCAGGAGCTGCTTCCGTCT
ACGTAACAAGATGTGCGGTTGTTCTTTTCGTCGTGGCTTTTGTGAATAACTTACGAACAAATTA

AACTGTTTTTGGTTTTAATCTCACACACACACACACTTATAAACACACACTGAAATTGCCTGTG
TTAATTATTTGTTGTGCTGTAAAAAATGGCGCTGAACTAGTTAATTGAGGCGCAAATGTAACAA
AATGTTGCTGCTGCTTCGAGGCTAACTCACTTTGGCCCTTGGCCATTTGTAAAATGTCAAAAAT
GAAAACTGTTTGTGGCTAAACAAGCACACACACATACCAGCACACCCATTCGCACGCCCACACA
CACACACACACACACACACACACACACACACACACACACACACACACACACACACACACACACA
CACACACACACACGAAGAAAGACAGCGCTAAGCAGAGTTATGAACAAAATTGTATTGCCTGCGA
CTCGGAGGACAGTCAAAAGTCCGCTCAGGCATGTTCCAAATTTGTCTTGGCTTATGAGGGCGAC
ACAAAGCGCATTGTTGGTCGTAAAGGGGGCAAGTGGTAGAAAGGAGCGGGGGCTGCTGGGTGTG
TGGCTGCAAATAACAAGGGAGGTCGGCCATAGCTTGTTGCTTTCGACTGGGGCCCATCAAGGCC
AAAGCGCTTTGTCCGCCAAGATAGTTTGTTCTGCATTTTGGCCTCACGAAATTGCGCACATTCA
TTTCCCGTTCAGGAACACGGAGACAGTCAAATGGACAGCGCTTTTCCAGCATAGCCCCTGCCTC
GTAAGACCCACTGGGCCAGGTCTTAAGAGAATAGGTTAATTGCGAATTTATCAGCTGTTAGGAA
TTTGTTTGGCCTTGTTTGAAGTCCAATGTATATTCATAGCCAGCCACTAATTCTTTCTAAAATT
AAACGGTGCTTGCATATCTTTTCACATATCCATATATATATGTTTATTCTCGAAGCAAATCCTC
AAAATGAGAGTGTAGCTGTTATTTTCACTTTTTTTTTCTTTTAATTTGATTTATTAAGTGCCCA
TCAGAATTTATAAGGTGAATGTTCTATAATGCGCTTAGTATCTATGTTAAGTATCTCCAGGCTT
GGACAGTAAATTGTAAATTTGGGACAAGCAGTATGACTTTCACTTTTACAACTGGTTGGGAGGG
TATTTTAATTATTCAGTTGTTTTACCCCTTTTTTTTAATTTAGAGCGAGTTTTAAGTCAATCGA
TTAAAAGGGGAAAATTACAATCAACAGTTTCCGCCCGTTTTATGATAAAGAACATTGCAAAATG
AGTTGAAACCCGCTCGGGCGATAATTAAAACTTCGCCCATGTAAGCTTTGTCCCATGTAACCTT
TGTCCTTAGTTTTTCACCCAAGGATGGTGTCCTTTCCGAAAAGTACAAAGAATGGTCTTTCGAA
TTAAAAAAAAAACCTCTGAAAATGAGCTTGAATCGTATCGAATAAAATAGAATGTGCTCCCACA
ATTGAGAAAACAAGGTAAACAATTACTAAACAAGTAAGAGGTTCTAGTCGGGAGCTCCCGACTA
GGGGATACCCTGCACCCTCTTCTTCCAACATCAAATGCATATATATATATATTCTATTTTAGAA
GCTATATGTCAAGTTTGGTGACTCTAGCTCTTATTATTTACCAAAATTGCCCAAAAAACAGGAT
ATCGATACCGATTTTTATCGATTACTTGAAAACGGGGCAAGCTATCGATTATCGGAAACAAACT
CGACCTGCGCAGGCACTAGGAGCACCTACATCTAAAATTTCAGTCTCTAGCTCTTATAGGTTCT
GAGATCCTTGCGTTCATTCATACGGACGGACGGACGGACGGACGGACGGACGGACAGACAGACG
GACATAACTAGATCGACTCGGCTATTGATGCTGATCAAGAATATATATACTTTATGGAGTCGGA
GATGCTTCCTTCTGCCTGTTACATATATTTGGATTTTGCACAAATACAATATACCCTTAAACCC
ATTTTTAATGGGTTCAGGGTATAAAAATGAACTGGATCGCAGCATACAAAGGTTCCGAATATAT
AAAATAAAATGAATGTATTTAAGAATCAAAAATTTATAATTTATTGGTGCCAATTGAATTATTA
AATGAACTAGTTCAGTGAGTTCTCTAGCTGCCTCAAACACTGGTTCCTATAGCCACACATATAT
ATATTAAAGATATTGAGAATACGTTTGTGCATTATTTATTAAGGTAAACTACTGCAAATTTCCT
TTGATTTTCTTAGGATCTGGATCATGCCAGCGATTGTGAATCAGTTTAGCTTGTAAAAAACTAG
TTCAGCTATGTATTCTTCAGGCACTTAATTATTGATAGAAGTTTCAATTTTCTGTCTTTCAACT
CTCGTTTAACCTTAAAAAAGCTTCGCAAACTAGTTGAAAACTTAGATAAAGTTGTGCTTGGCAT
GGTCCATGCCCGTATGATTCTTTTTCCCCGCAGTCGAGCAACAATTGTCATAGATTCGCCATGA
ATTGTGCCCAACTCATCTCGTCTCACACGATTAAGCCTAATTCAAGCTTAACATCAACGTGCGA
CTCGCTTGCTTATTAAAATTTATGATTTCCTTCTCTGCGTCCGTGTGTGGGTGCCTTTGTTTGG
GCTTGGGGCGGAGCGTTGTATGATGTGTTGGAAAAGTTTTACAACTTTTACCAAATTGTAAAGT
ACGCCCCTCAAGTACTAAAACTTTTACGCCTCCAAAAAGTATGCAATGTGCATGTTACAGTCGT
CAATTGTACTCATGATCTGCATGTGTGCGTGTGCGTGTGCGTGTGCGTTTGCGTGTGTGTCTGT
GTGTGCTTCGAGTCAAATGTCACAAAGGAGCATGCTGCTCAAGCAACGGGAGCAGCATTAAAAA
TTTAACAAAAGAAATGTGAAAATATGTCAGCGGGTTAAGCAGCAGCTGAAAAACAACGTGCTGG
CGCATGCCGTTTGCCCATGGAGCATGGGGCGTGGTCGTGGCATTAAATTATGAACTCTACAGTG
CATGTGCCATGTAGCAGACGAGACGGGAGTCCTGTGGCAAGGCAGGGCGTACAGAGTTTAGGGG
CGTGGACAGACACACAGCAGACATAGTTGCAATGCGACAAGCAACGAAACGTGAAAGTCACAAG
CTGCCAAATCTCTTCTACAATTCGCAACAATTTCGCCAACTATTCGCCAACTAAGTATATAACT
CAGCTTAAACTCAACAAAAGCTGGGTAAATAAATTTCGCGATTCAATGTTGTCGTTTATGTTCT
TAAAGCAAACTTTAGTGTTTGGACTATTTCGGTAACACTTTTGCGTGTTGAAGCGCTGTTGCAT
GTTCATGTTTAGTCTAGAACATAACTTGTGCTTAAATGCTGAGTAGTTTCATACACCCACACAT
ACATACACACACACATACACGCATAGACGGTTACCCTGAGGGAACAGCCAAGGGCTAATTTGAT
TTTATTAATGAAATCATTAGTAGAAATGACAAAACGTAGCCCGAAGGTAAGGTTGTAGTCAGGA
CACGGCTGCAACCCGGTCAGCAGTCGACCAGACGAAATGAATTTAATGTAGCATGTGAGTGGTC
GTAACAGCTATTCCCTCTTCATACCCTGTACTTTGGTGGGGTATCATACAATTTGTAATCCTAT

ACCATCTTATATGGAAACAATTTAATAATATATATAAAAATAAAATATATAAACAATATTATAA
ATTCAACTTATGTCAAAATGTATATACTTGGCTATTCCAAATATATATTGATTGTTATTATCAT
TATGATAATTATTAATAAATGTATAGGATATCAAGCTTACATTTCAAACATTAAACTGTCTACT
GGAGGCTTGAGGTAAGACAAATACCGCATTCAAACAATAGCCCGCATCCACGTTGTTCATTTGA
CGAAAATTCTGGTTCACTTAAACAATTCATTTACCGTGGAATGCATTGTCAATGTGACTAAATG
TTTGTATAAACGTCAGTCTTCTAATACAAAGCATCCAGGATTAATTGCCATTGAATTTCATCGA
TATTTATCGAAAAAGGGAAGTAATTCCGGGCCTATTCTTCCTTCTATCTATGCAAAGGGTATTT
TAGTTTTAAGAAATATCTTCGTCTTTAAGTATATATAAGTATATATATATATATATATTCTTGA
TCAGTCAATCAGTTTGCGTTGACAAACTTCACACATTTTTCTCCAAGGAACATTTATTATGTGT
ATTCACTATTGAGTTGATTCATAATGTTAAATATCTATACATCATATGACGACCGACAGAACTT
TTGGTCGTTTTGAGTTATTCTATTACAAGCCATATGTGCTTTTCAAGATATCGTGATTAAAGTT
AAAAGTTGACAGCATGGAAGTACTATTTATATCAAATAGTTCCTATAGAAGAAGTCTGTCCCAA
TAGTTGGCTTCACTATGCTCGCAATATATGTACGAGCGATAGCGTACGGGTATATAGGAATAAG
TCGTGCCACGGATATTCTTCTGCCAGGGTATATCAAATTCGAAGCTCCGCAGAGAAGTGTACTT
CCATATTTTTTTTTCGTTTGTGGCCATTGTCATTGCTTTGTCCTGTAGCAGGAGTCCTGGTGCG
ACTCTTTGTCATGTCAGTGGCTCATTTCATGCGTACCCTTTGTGGGCAAACTCCCACCCAACAG
ACAACAGCCACCACCCACGGGGCGGGGTATGCCGCTGAAAGCTGAATGGTAAATACGTAGTGCG
ATATAACGGTAACGGATGTGGTCATTCACACAAATTAAATATTTGCTTTTCGCTATTGTAAGCG
GAACGTCATGGTCCGATGTGGGAAATTTTATGCATTTCCCAACGCCATGTTAAATTAATTGAGG
CAAATTTTCATGGTAGACCAGAAAACATGAGCTTCTGGCACCCGGGTAAAACAGCTGTAAATAC
AAAATACGAAATACCAATTTACGCATTTCATTTGCGCGCTTTGTTCATTTTTTGGATCGTCGCA
AGGTTTCGGCGTTCAACAAAGTTGAACAACAGAAGCTATAAAACTGGCAACTGTTGAACACTTT
AATGGCACTTTAATAGTAGTTGAGTCTTTTAAACTGATTGCAATGCTAAAAAAAGTAGTCTTCT
GCTCAAATCAACTCGATCTAAGTTCCTTTACAACTTTCTGATTGCTCTATGGCACTATAATATA
TATATACATAAATAATATAATATATACATAAATAATATAAAATTGCCCAAAAAACAGGATATCG
ATATCGATTTTTATCGATTGCTTGGAAACGGAGTAAGTTATCGACTATCGAAAACAAACTCGAT
CTGCGCGGGAACTAGGGGTATCTACATCTAAAATTTTCTCTAGCTCTTATAGGTTTTGATATCC
TTGCGTTCATACATACGGACAGACGGACATAACTAGATCGACTCGTCTATTGATGCTGATCAAG
AATATATATACTTTATGGAGTCGGAGATGCTTCCTTCTGCCTGTTACATACATTTGCATTTTGC
ACAAATACAATATACCCTCATACCCATTTTTAATGGGTTCAGGGTATAAAAACTAAAAAAACAC
TTGTATAGTTTGATAGTTGGGACTATTTAAAAAAGAGGAATATATGTACATTTATTTGAACTAG
CTTTTAAAATTAACACATTAGAAATTCCTGCAGGTTGGGTTGGGAACTTAATTAAAGCATCGGC
CTGCACACAGATTTCCAGCCAGTCGAACCCTAACCTGAACTGAACAATATTTTGGGAAACGATG
AGAACAATTATCTAGTTTAAATAATTGAAAACTTGTTAATTTCTTAGTTCTATGAATAAGAAAA
ATTAACAAGCAAATTTGATTTATAATAAATTTAATTGTTCTACTAAAACTTGTAGAAAGTTTGT
TTCACGAACCGCACCTTTAAGCTTTTCTTTGTAAAAAACGTTTCGGTATTACTTGCTAGTTAAA
ATTGATTAAATTAAGCCGAGCAACGACGGGTTCTTTACTGTAGAAGCATGCATGAAGTAAAAGA
GATTCCTTGGAACTTTTTAAAAATATTACCATAGTATCATAACATCATGTCTCTGAACCTTTCT
CTGCTCATATTTGTATATAGATTTATTATATGAAACTCTTTTTTTTATACACTTTCCACATTTG
TTACGCTTTCACAGATGATTTTTAAAAAGAACCTAAATAGAACAAATATGCAATGTAATCTTTA
ATGTTCGACTTGCAATAGTTCTCTCCTCGTGCATCATCAAGGCTTGGCTTGGCTTAAGCCAAGT
TCCTTTGAATAGCATGCCTAACAATGAGCCACTTGCATTCGCCTGGATCTATTCAAATGGATTT
CTTAGAAGTGCCGCTAGACTAGAGACTATTTCCTTTTTCGCATTATTATTTGCATCTTGGTATG
CTGGCCATTGTCAGATGGACAGCCTTAATAATCATCTCACACACAGCAACAATCAAAGTCCCGC
CCATTGAGATGCTAATGTGGCCATGTGCGTGCCTCTAGGCCCAACACTTGCCGTTATGTCTACC
CGACACCTACTTACATATGCATATACATATCATGGCTACTCAGCTTGCCAATTTGTATATCCTT
GTAGAGGGTGTTATAATGTCACAAAGTGTGTAACGCACAGAAGGAGCAGTATCCGACCCCATAC
ACTATATGTACTAAAATACATATTCTTGATTATGTAGCCGTATCCGTTTGTGTGCTTCTATGTT
AACTTGGTTTTTTATAGATCCGTCTTTCTGTAACAGGATGTGCCGGGTCGGAACACGATACCAT
TTAGGTGTCATATAAATGTTGCCCATGAAATAAGTTGTCCTTCGACAGCCTTAACATTATTAAA
GATTCATTACCCAGAATTGGCATTCTCGAGCTTCATTGTAATCTCTACATAGGAGTGAAATCTT
ATCAGAATCGATTATATCATTGAAAAATTCGTTTTTAATGGTTCGATATTCTGGTCTTTAAGTT
CAGCAAATTCGAGCTCCACTTTTTTATTTAACATATTCGCTAGGGCGTATCCAACCGGATAAGA
TTGCTCCAACGCCTATTTATAAAAGCTTTGCTAAGTCGTAGTCGTATATCCAAATATATGTAAC
AGGCAGAAGGAAGCATCTTCGACCCCATAAAGTATATATATTCTTGATCAGCATCAATAGCGGA
GTCGATCTAGCCATGTCCGTCTGTCTGTCTGTCCGTCCGTCCGTATGTATGAACGCAAGGATCC

AAGAACCTATAAGAGCTAGAGAAAATTTTAGATGTAGATGCCCCTAGTTCCCGCGCAGATCGAG
TTTGCTTCCGATAATCGATAACTTACTCAGTTTACAAGCAATTGATAAAAATCGATATCGATAT
CCTGTTTTTTGGGCAATTTTGGTAAATAATAAGAGCTAGAGTCACCAAACTTGACATGTAGCTT
CTAAAATAGAATATATATATGCATTTGATGTTGGAAGAAGAGGGTTCAGGGTATGCCCTAGTCG
GGAGCTCCCGACTAGAACCTCTTACTTGTTTAATAATGTACCTACAATTGTTTTCCTTTTGTTA
GTCTAGGATATTTTGCAGTGAGGCATTTCTTAAAGTCTTAGTTATTCTTTAAGATGTTTCACGA
AAAATGGATTATAGAGCATGTTTGTGTAAATGGGTTTGCCAATGACACTGGCTCCTGGCTCAGA
CTGTGACTAAGCACAACGGTAGCATGTGTACACACACACACACACACACACACACACACACACA
CACACACA
>Contig6712:1..4134
ACACACACACACACACACACACACACACACACACACACACACACAGACAGACACACGCACACACCA
TAGGTAAGCATGCATATCTATGTGGACGTGCCCCATTGAAGCGCACCTAAGCACAACAAACAAA
TCAAATGTGGCAAGGATGCGTTGCGTATACGCACCGAGGCGCTAAACGTGCTGTCTAAATGTTT
GAGTTGGCTAGATAAAGACTAGAACGTACCGGCAGCATCAGCGGCAACGGTAGCGATAAACGGA
TCGTCCTCGTATCGGGTCTGCATTCCGCCCAGACACGCCACGTTTGGAGTACGAGTGCCTTCGA
CACGGGCACATCGCTTTCGCTAAGACAATAACCGATGCTAATGTATTATACGTGTCAATTTTGA
ATCCCGTTTGACTGATATGTGTGCTCACATCACTGGGCCACACTGCCTGGCTGCGCGTGTGCCC
ATATGGGCGTCATAATACATTGCCAGCCTGTGCCACGCCCAGACGCCCAGACGCCCACTGTTAT
GCCCACAGAGTCTGACTCTCTAGTCTAGGCAGTGGGTAGTCGTGGTCTCTTGATAGAAATGTCG
CTGTCAATTTGTGACAAATAACTTTATCATACAATGCAATTGATAAGCCAACAAGTTGTGCGGC
CAGGCGAGAGCAGAGGCAGGGGAAGTGGAAGTGCAAGGGGAAGGCTGTCGATTGATAACCGATA
ACATAGCATAGTGGAAATAGCTATCGATCATAAAAGGCTAGACAGCAGGAATAAATGCTAAATA
GATTTTTGCGCAAAAGTTATTTACGAAAATTGTGAAGGCAAAGGCAATCTGGTTAAACATTAAG
CCGCACAAATATATATATATATATATATTCGTGATCAGCATTGCTCAACCGAGTCGATTTAGGG
ATGTGCGTCTGTCTGTGTAATTTTTATACCTACCAATGAAGAGACTTTATAGACATAAACGGAT
TATGTGGGTGAAGCTTGCTTTAAATGATTTATAACTTTCGCGTTTTCACAAATCGATTATTAAT
TTAGTGGATAAAATATAAATACAAAAGCTTTTGTATTTTTGTAAAATCGATTACTATTGATTAA
TATAAGCCCATGGCTTTTTCATCTGTAAGGTATTAAGCGTACGATCATTTTGTATTCTACATGC
CGAGCATGCATTTCTCACTTTTTAGATACCTCTTCCCATTCCCCGCAAAATTGAAATGCAGCTT
ATTTTAACCGGTCAAACGCTGAATATTTTCCATAGACCGGACTACATATACACATCGAGGGCAT
TCAAGAATGGAGTTCACATAGCGTGTGCCTGAAGAAGCACACAGGTTCGAAGGCATTGCAGGGC
AGCTCGAAGTTGTGCATAGGGTATTTCGAGCACTTTCCACAATGATATCTAATATGCTTTGAAT
TCAAATGGCATTGCTCGAACGCATGTACTATAACTTAAATCACAGATCAAATAGGCTGCTGCGG
TATTTGGTCGAGCTGTCGAGCGGTTGGCTACGCGATGGCCTCAAATTCTGGCAGAGCTGTGGTT
TTTCTATTCAATACGAACAGAACACAGATCTGTGGCTACGGCCTCAATGCGAACTGCCATATTT
CTCCTAGACTTAATGGTAATACAATCGTCGGCAGAGCAGACTGCGGGCCAGAAATATCGATTTT
TGGCCTTTGGTTGAAGCCAAATCAAGCAGATAAATATGCTTAAAATTGCACTCTACTTTTTCAA
TGGAAGATCCCAACACAAGCTAAAATATGTAGCGATTGTTTCATATTCCAACAAAAAAAAACAT
TGAATGAATTTCTTTCAAACTTGAAGAAGATAAATTTGAGCACTAATTTGAAAGTGAAAAAAAA
AAGTCAAGAAATTATTCAGTTATTAGCATAAACATTTACTAATTCTTAGTGCACAGCATGTAAA
GTTTTTTTAGTCTTAGACTTTTTTGCAGCGTATCTTCTAGTCGAGCACTTTTGACTCTAGCTCG
CTGGTTTATTTGCTTCGTGAGCATGTGTGCGAGCTTTGATGTGTTTTATGCATCAAATGAGTTA
GCGTCGAGCCTAAATAAGAGCTAAACACTCGTGCAAGTACACACACACACACCCGCCCACAGAT
ACATTTGCAGATACAGTGGGCGGCAGCTACGTGAGCGGGCCACATTTATGGGCCTTCAACGGCA
AATATGTGCATGCTGGCTTTTACTGTGTGTGTGGGTGTGTGTGTGTTTTAAGTAAGACATTTGC
GTAAACTGTGAACATTTAAATTGAATCAAGTTCTGGGCATGCCGAAAGGCTTCCGGACTAACAC
TGAGAGTCGGATGCGTTGGTAATGAAACCCTTGTGGATTGTCAGCGTTTGGCCAACTTAAGTGA
CATTCTTTTCTTCTCTTTTATTTTTCTGGGCAACTTTTATGATGTTGGGGCAAAGTCCTGCGAC
AATAATTTAACTTGTCTGCTTTTTTATGCGACTTTCATACGCTGCTTGCCAACTGGGATAAAAC
GCAAAAAAGTTTAAACAGTTTACAGTTTGCAGCTGCCCTCAAGCGTCTCAGTCGACGTCGTCGT
CGCCGTCGCCATCGATACGCTTTCAGCGCCACGTTTCCATTCACAAATTCAAGCTTATGGGGCT
GGGCAGGGGTTGAAAGCCACAAGGCGACAGCTGGGTTACAAAACGTCTGGAACTGGTTCTGTTT
TCCTGTGCTACAAGCGAAAGTTTAACGCTTGGCAGCGCAGCACATCAAGCATACGCACTGTTGC
ATGCCCCATGTCTGATGGCTGTCGACTGTTGCTGCGGCTGTTGCTGCTGCTGCTGCTGCTGCTA
CTGCGGCTTCGGCTGTTCTTGTGGGAAGTACTGTGACTGGGCAGCTAACCCTTCAGATGCGGGC
TTAGCAAAAATTAAACGAAGCAATAAATTATGCGTCTGCCAATGGGCGCCAGTCAGGCAACAAC

AAAAAAAAAAAAAAAACGAAAGACAAATTTTTATATATATACGACTTTCTATATATAAAAAATGG
TGCCTTTGTGTGCGTGTGTGTGCGTGTGTGTGTGCTTAAAAATCCCATTTTGTATGCAGATTTG
CCTAATCTTAGGGTTTTACTCTGTGTATTTGTGTGTGTGTGTGTGTGTGTGTGTGTGCAACT
GGCAGCAGTTTAGCGACTATTATAAACCGGAAATTTTACCCAACACAGCTTCGTATTCCGGTCT
TTGGATCTTTTTGGGGCGCCTCAGCATATGACAGTGACAGGGCCGAAATGCGATCTTAAATTCA
TGAATGCTTCATTTGTGATTTTTTAAGATTTCTTGCCAATTCGCTGGCTTAGATAAAAATGTGT
GGTCGTGCTTGCCACCTGTTGGGCTAACAGATTTATCAACTGCGTGGCAATAAACTATATTATT
TGATTATTATATTTTGTTGTAGACATTATACCCAGTTTTGCCCTTCTAACAACATTTCTTGTAT
GCATCTTAAATTTTTTGTTAACTTGCATGTGCAAAATCGAACACTTTTAAATTATCTGGGCGTG
TCTCCAGTCTGTGACCATAAGTAATAGACCCTCCAGTCGTTATAACGACAGCCTCTAGATTCTG
GCAACCTGTGGGCTTTTGGAGGTGAGCCCATAGTTCGGATTCCATTCATCTAGACGAAAACTAA
ACACAACAACAAAATACAAATAATATATATATATATATAATTGAACTGCACGGGCGCGTGGCCAGA
GCGACTCGGCAACCCATTTGAAACATTGCTATACTAACGACCAAATCATAATAATTTATAGTTA
ATCTAATAAAAAATAGGGTGTTCTTTGGTGAATCAAGTCGAACTTTAAGCCATGAATGGGTTAA
AGAAAGTTTGCCAAAATGTTTTTCAATTACTGTGGCAAACTCTGGCTAAGCCTCAACTCAAACT
GTGCCCTTCCCAATTCCCTTCAGTGACTCAATTGCAGAAAACTTATCTAATCGCCAGAGATTAC
ATGAAAAAAAGGCTGCAAAATGTCGTGCGAAAATCTTTTAACAGATTTCCATAAATTCATGTAT
ACAGACACATTTTGTAAAAATATATGTTAATATATGCGTGCAGTCGTGTCTGTATCTAAAAGGG
CTGTGTCATTAGCTGGGTCTAATAGGAACGCCCTCAGC
>Contig15014:1..536
GCCACGCCCCCAAGCGTACGCCTACCGTAAACTGTATTTAATGCGCATATTTATTTTATTTAAA
TATTTATGTATTTTTTGCTCCTTCTTGGATTATGTTTTTGAGTTACATTTTATTTGGTTGTGGA
TTAACTTAATTAATGCGTCGCATAAGCTGCCGCTTAGGCATTAGCTGACATTCTCGTCCTTGTT
GTCCTTCATCAAGTCAATCACAACAGCGGCATGAAAAGCAACAACAACAACAACAACAACAACA
ACAACAACAGCTTGTTATAACATTTCAAATGTTGCTTTGGCAGGTTTTTGTTTTGTCGCATTTT
TAATTCATTTTGCAGAGGATTTTTGCGGCTTATGAACTTTAAATATTTCGAGCCCCCCACGCCC
CCTACTTGCCCAAAGCAAACATAAATTGACAATTCAGAGACATATATACATACAGTACATGTCT
CTAAATATATATTTACATACATGGTTTATCTATCTATACAGGAACAACACGTGTGGGACAAAAA
AAAAAAAAAAACGACCTAAATTAA
>Contig3226:13717..1(rev_compl)
CGACACGCCTACATTAGGTATGCCTGTGTGTGTATGCTTGATGTTTATTTATGTAGTTGATGTT
GTGTTTGACATGTCACACACAAAAACACACACACACACAAAAACACACACACACACACACTAAC
AGCAACAACCACTGCCGACGCAGGGCACGTAACTCATACGCCATGCAGTACGCGCGACCTTTGG
CTAACAATCTTTGGTAGGAATAGTAGAAATCGTATACAGCTTAACTTATGCTGATTCTTATCCG
ATTTGTAGTTATATATCACAGACATATCTTGGTATGAAATGGAATGAATCGCTGGCCAATCGAT
TGGAAACAGACTTTGAATTGATCAAGCTAAATACTCAAATACATTCATATATTTTTTTTAACTC
TACGAACTCAGGTTTTGATTCGACAATTTAGTTCGTCACGTATTTTGAAAACTTTCAATGGAAC
AATATAATTTTATTTAAATTCATTACAGTGAATGCAAATATATTTAATTGTTGAAATTAGTTCA
ATATGCACAATACCAGAAACTCAGTTAAGGTTCGGCTATATTTTACAGAAAGTGCAATTTCAGA
TTTAAGCCGGTTCGATATTTTGATAGATAGAACAACGATTTTTTGACTACGCTCCCGCAAATTG
CAAATTTAATATCTAACAAAGTTTGAAATTCACATTTTCGAAGCATCTTCGTAGGTAAACAGTT
TTGGAGTCAATTAGTGTTGATCGATATTAGATGTATCAAAAAAGGGTAAACAGCTTTTACAACA
AATATATGAGTTGTTCGAAAAGAACGAACGGATCTACCTTATTAGTTCAGATTTTTGATACAGG
GTATAAAAAACTTGTCGAGCTATTGAACTATCGACAGACTCCTTGGTTGTATGGTTTTATTACT
CGCGTAATAATATGTTTGTGAAAATAGGGAAAAGCTCGCCCACGTTCAGGTTTGCAGCACAAAC
AAAAGGCAATTAAATGCAAGCTGGTGTTTTATTGCCTATAAGAATATATGTAAATAAATATGTA
TAATTTATAATATATTTTGAGATTTAATGTATTTTTAATATGACCTCTCCAGTATTTGCTAAAG
TCAAGCGTATCAATTACGATACGAACTATGCGAATGAACGAGAATCAAGCAGCGGCTTACTTTT
GGTGAAAGTCCAGTGAGTGGGCGACGGTACACGGTGTCTACTTGGAGGTGAGGTGCCTGGACAA
AGGTGTAGGGCAAACCAAACCAAACGCCAACGCATATAAAAGATTTATATTAAAGGCCCGGCCA
AGCGCCCGGAGGCGTTGCCCACGCAGCAATGACTATCATGGCATCCTCTTCGAATGCCTAAGCT
ACTCCTCCCGCTGATTCTATCTCTTCTGGCAGTGTAAGCGTATTTTGTAATTTATGATGGCAAA
CTCTCTGTGTGAGTACATCATACTGGCATACACACACACACACACACAGATGCTTACACATGTA
TGCTGAGTTGATTCAGAAAGCAAAAACAAACAAGTCTAATGCAACAACACGTGACAGGCGTAAG
TGCTTCAATAAAAATGCATAAATTATTGTTTATGCGTATTGAATTAAGCGACGCAAACACTAAC
ACGCCCCAAAAATTACGCATGCCACGCCCTCCCCCCTCCACACACACACACACACACACACACA

CACACACACACACACACGCAGATGATAAACTTTGCCGAGATTTGAAATGTATGCAAAAAATGTT
TCGCCGCTGTCAATAAAATAGAATTTTTCTATCCACGCCCACAAAATGCCAGACGCAGGCATTA
TAAACAATGAGGCTGTGGCATTCGACAATATTTGTTATTTATTGAGATTTGTTTACATTATATT
GTATGCGGAATTTGAAATGCCAGCCCCAGAGTGCCACGCCCAAGTGCCCATTCCACCTTCATGG
CCAGACTATGCTCCGGTATCCTGTGCCGGCCACCGGAAGCCGTTTTCCTCTGCCCCCAACATTG
TTGCTCCACATATTTCCCACTTTTCCACTTCCTCCTTCAGCTGAATGGAGGCATTTTAATATAT
GTACACTTCACAGCCTGTGTCTCAGTGTGTGGGCGTGGCATGTCAACAGGGAAATTCGAAACTG
GAAAAGCTAAGCGAGTTGTTGGACATGGACGCGCTATCTTTTCCGTCTATACTTGAGCAGATAT
CGGATGGACATTAACCATTACAAGGTTGAAGTAAAATATATAGTAATAATAAGAATAATTAAAA
TTAGAATAGTAGCTATATGCATGTATACTTATAAAAATAATAATAATAATAGGGGCCATGCCCT
GATGCCTTTATTTTAATTGGGTACAACTTAGTTTGTGCTTAATTTGTTATTTTCTGCACATACC
AAATGAATGTTATCTTAAATAGAAATCTTTAGTATATGCTGCGATGAAAATATGAAGCTTTTAT
TTCTGCTTAACACAATTGTTTTCTGTATTGTACTAAGAGGAATTATTTTGGGTCAGAATTCTCA
ATTAAATATATACTGAATGTGAACGAAGTAGTCGGGTTTTTGAATGTTAAACTTCTTGCCGCGT
ATCTTACACACGTACATCGTTAATTGTATGTAGATTGAGTTGAATATTAATAAGCACTATCAAA
TATAGGGAAATCTATTCTTCTTCCTATAATTCCATGACACGAAACGCACGCTCACTGTGCCAGT
CAAACAATTTGAATGATATTTGATCTTATACAGAATTTTGAATACCAAGCATAAATCAGCAGAA
TTCATGAGCCATAAAATCTTAATATTCACTTAAATTGCTTCAGTTGCCATGGAAATGCGGGCTA
AATAACCCAAATGTGTACATGTAGGTATACATATTTGTATATATATATATATATATATCGGGTT
GAGTTGAGGAAAGCCTCTGTGGATTCACTTGAGGGATGGTCAAGTTAACAACTTGTGATTGAGC
TGGGGTGAATCTTTCCTATAAATCTGAGGAGGGGAGCAACTTAATCAAAGCCGCAACTGCCATA
AAATTAGAAAGCATGCGATGAGGTCGACCAAGCGATGACAATGGGGGTCACGGTCAGCCTTAGA
CTTGAGGCTAGGGGCAGGACGAGCCGGCACGTAAATCCTTCAGGCTTGCAGGCGTCAAACAGTT
GATTAATTCGTCGATTTTATTTGGCGTTTTTATGAGCAGCTGCAGCCGTGAGTCGTGTCACTCC
TCGTATGTCAGTTCGGAGTCAGGGCTGTTGTCAACTTTTGTTTGCCTGACATCGGCTGCGGATT
ACGCGAAACTTGGCCAAAAATAACTGCGCCTATGGAGCCACGTGGCTATCAAATTTCCATTTCC
AAAGCCAAGCAGCACAAGTAACTCGCATGGCCCCAAAACGAAACCACATCGATAACCGAACCCA
ACCGAAACAGAAGCCGAAACCGCTCCGAAATCAAACATTGTTGAACCAAGCCTGAGCTGGTGCG
ACAAGGCGTCAAACCGGCCCAACATGGAGAATAGGCGATACTTTATTTTACCATTAATCGTAAA
AATTACTCATTTTATAGCTTTCGAAGTGTTTTCTCGGTGCCAGTCGCTGAGAAAGTTTGCATGT
GCCACCGCATGGCATAAGGACTCAGGCTCAGTGAGTGTCATCCAGTTGCGAAGCGCTGCGGCGT
GAAGACAAGCGATGCGTGAAGCAGTCGAGAAAAAAAAAGACCAACAAAGCCTGACACCAAATGC
CACACACCGACCAGGTGGCCTCCGGCAATGCCTCAAATATCGATGTTAAGCGTTATGCATAAAT
CAGCGTTTTGTCTCAGATATGTCTTCTTTTCGTCTTGTGGGATGTGCCGGCAAAGAGGACATGT
GTAACTGCTCAAACGCATGTGGGAGCGGTGTGTGTCTATGTGTGTGTATAAATACAGGTGTGCA
AGGTATTCGATTCATATTGAGGAAGCTAAGGATTTATGTCCCAGGAATCGTTCTAATCAAAAGG
AAATCTTTTATATTCGTTGATCCGAGAATAATTAATTTAGCTACTGCTGCAGCATATCGATTAT
GAGCTGCCTGTGCCTCCTAAGTACTTAATTAATTGACATACTTAAGAGTTTAGATGAGTAATAT
GTCAGTTTAGGCAATATTTTTAAATGGATTTTTCCTTACACCGCATTACTTGGGTAAAGAAAGG
TTGAACCCAAACTGAAATAAATGTTTTAGAATAATATATATATTTAGAGCATGGAAAAAATTT
GTTCTCTGAATCTGATAACTTATTTTTTTGAATCAACTAAAAAGGTTTTTATTGAAAAATGGCA
AAAAAGTGTCAAGCCAAGGCTCACACCAAGGCATGGTTTGATAACAGTGGAAAACTAGGCTCAC
GAACCGAAACCTTGGCGAAGCATTGATTCGAACCACCAAGAATTTGTATTGGGTTGGCAAACGT
ATTTTTCTTTAAAGCAGATTGTGGCTAACAGCTGCTGAAATGTTTAAAAACAGATCAAATGCTA
GCCTCAAATACGCTTGTCTATTAAACGTTGTATTCAATATGGAAGGCTTGCCCCGATCAAAAGG
CTTGGCTAACATACTTATTGGTTATTTATAATTGAATCAATATGCGGGCATTGTCTTTAAATTG
AAGACAAATATTTAAAAATTGGGCACCTTTACCTAGGAGATATAACATGTGCCTCTTATGGAGG
CAGTGCTGAAAAGCACCAACAGCTGCATCATTTTTATTACACACATACATAATAATACATTTGT
ATGCATATATTTTTGTTATTTTGTGTTTCTTTGATTTTATTGAAGGGGCACTTAAAAAAGAAAC
AACTTCGAAAATAAAAAGGCGACTCAACCTAAACAAATTTCACGTCGTGTCCTTCCATGGAACT
GAGCATCCAGTCGACGTTCATCCGCCTTCAATTTTCCCGCTTCTACACGCGCACCCATGCCCAG
CCGCAGTTATTACTCATTTACAGACATTTACTTAAAGCAGGGTCTGGGTAGAGTAGAAAAGACT
GGGAAAGGTTGGGGGCTTGGGGCTGGGGGCACCTTCTGTTATTTGCATTGGACAACAAAATGAG
CAGCGCCGAAAAAGTTTCAAATCTTTTGAATGAATTTAATATTCACAAAGTATGAAGAATACTC
GCAAGCAAACTTTGCACAGAGCAGCGAGAGTTAGGCTGGGGTGGGACACCATCCCCCAAAGTTC
AAGCTCCTTACTCCATTCCCAAAATTACCATTCACATCATCATCAACATTATCCTTACCCTGGT

TAGTATCATCAGCATCGTCAGTATCGTGTGCTCGTATATGATTTGTCTATTTTCTGTTTGAATA
TGTTTCATGAAAGACTTTATCACCCTCCGTTCCGACCAGCCAAGCAGCCACTTGCTCGCAAAAG
GTTTAGCATGGCTGGCCATGAATGCCACCTGGCTGATGGCTGGTGGCGGGTGACAGGTGGCAGG
TGGCAAGTACTGGTGGCAGGTAAAAGTGACAAGTATATCGACATCCGGACAGTTGCAATTTGTG
CTACGTTCTACCGAAATGCTTGGCTGCCTTTTCTTACATAACGTTTTAATTGCCCCTGCAGTAG
AAAAAATAAACCAAAAGAGAAGAATTGAATTGTTTGCTATTGGTCATCATGAGGACCTCAAGCC
AGAGCGAAAAAGTTTAGATGAATTCTGAATGCAGTCGAGTGTGGAGCAGATTATGAGAAAATTA
ACGACTTTTTAATTGTTTGTTAGGGTTTGTTTTGCGTTTTATTTTGTTTTTTTTTTTTTCTTTC
ATTTTTTCTTGATGCTAATTGGATTGCCAATTAATAAGTTGCATTCGCCTTATGTCGAGTTTGA
GCATAAATTGTCAATTCACAAATATAACGTAAAATACTTTGTTTTTAATTGAATTGAAAAGTCC
TAAGCCATATTAAGCCAGTTGGATCTTGTTCCTATGTAAATCAAACGCCTAAAAGGCGCTGCTC
ATCAAAAAGGAGCCGCTCAATTTTTTCAGCAGCCGCTACGCAGACAATCTGCCTCGGCATTCCA
AAGCGGGTCAGGGTCCAGATCCGCATTCTCTAATGGGCTCCTTCAAAAGATACACACCCGCGAA
CTATCCTCAAAGCGAATCTTTTTACGTTCTCTTATTTTATTTAAATATCTTATCAGTGTAAACA
CCCTGTCATTGAAACATATGTACCCTCTAAGCTACACAACCTAGAAACTAAAATCCACACTCTG
AGTCATTCGCTTGGTAGAGATCAGAATGTTCTGATCCATTCCAGCGTACATTTAAAGTCAAGCT
ATCATATTTTTTGGATTATTAAATTGAAACAACGCGATTTCATTTGCTCGTTTTCTCATTGCAA
GTCTAACGTTCGCTTCATGTAATTTCCTTATGTATTTGTTACTGAATTCGCTTTGTAAGATTTA
TATAACACTTCATATGTTGATGTCTAATGGATTTCTTGACTTGCAAAGAAAAAATTCACAGATG
AAAATATGGCAGCCTGCTGCACCTTGTGAACAAAAAAAAAAAGTAACTGACTGCTGTTAAATAA
GAATTTTCTATGCAAGCATTAAAATGGATTTTAATAAACTAAAATGCATTTTTATCAATAAATT
TCCAACGTATATCCATCCCTTTGTGTAATTGTATACACTAAATTTCGTTGCAATATAGATAAAC
AAAAGGAATTATTTAATTTGTAAACTATTGACAACGGTCTTAAAGGCAATAATACATAACAGAC
TAGCTTGATTAAGCATTCTCTCCTCTGTTTAGTTGCAATTGTAAATGTGCTAACATTTACTCTG
TAAATATACATATGTTAACTTCCTAACAGACGGCTAACATTATGTTCCAGGGCTCTGAATGCTT
ATGAGGGTAAGTCAAATGCTAATGAAATAATTATAACCTGAAATAGTGAAGCAACAATTTGAAA
TTGCACTTGGTTTCACTTAAAATTATTTGCTTTTGATCATGAAGATCTGCAAATATATAACTTA
ATTAGTTAATAGACGGTTAACAACATGTTTAAGTGGTTAAATCAAACAACTTAACCTTTTAACA
GACGAATAACATTAGTTTCAAGGTTAATTTCGTTTGTTCAGCTCAAATAGCAGCCAAAAACATA
AGCCCAAACTCAATCTAAAGAGCTATTTGCAAAGTGGTGGCCAGTACCGTTGACAGTCCAATTG
ACCAGGATGCGCAGTCCGGGCACAGTCCGGGCACAGTCCGGACAGTGAAAATCACTTTGAAGCC
AAATCATAGGGAATTCGCACGCACAGGATCAAATAGTGTAGTAGAAGAGCGTTGAGCGATGAGC
ATTGAATAATGGCTTTAATGATGGCCCAAGCACATCAACGACTGTTTAACGGACTTTAGCCTT
TGAACCGTCACACACACATACGCACACACACACACAGTATACGAGCAGCGGGGGCATTAAAATA
GCATTAAAGCATATCTAGATGCGAAACGCATCCGCTCGATATTATTGCGAAACATATATGTCCA
TGCATATATATACATTTTTATATACATTTATATATGTATATAAATGTGTGTGTGTGTGTGTTGG
TAAATGCGAAAATGTGAAATGGCAAATTCAATGAAATATTTATTAACCTTTAATCAGCGAACAC
AGGCAGACAGCAGACAGCAGACAGCAGACAGTTGGCCAATAGACAGAACATAGCGTCGCCTAAG
CATATTTTATTACACATTTCATGGGACAACGCTGCGTATGTGTGATGCGATACGTTGTGATGTG
ATATATTTGCTTGATTTATTTCGGCTACTGCAATAATCAAAATTATTTGGTACCAGGTCAGTGT
CTGAATGTTAATTGCCATGCCAGACTAGGCCAGCTTAAGTGGAATGAGATGATTGTCGGATATT
GCTAGGGTATATTTTGGGTATAATGTAACTTCAGGCAGGAAGCATGCAAAATTGTGAGAGAAAT
TGCATTTTATGGCTTTGCATATGTGAGCGACCCTTAATTCGATTTGTTCAGCCAGCATGAAAAT
CTCATTAAATACACAATTTGATCAATTTTGATTGTTTTTGTGACCAAACCAAATGAATTGTAAT
TAAGTTATGCACAGCATAAAAATAACTAAAACGATTGCTTACCCAATTTAATATAAAGCGTTTC
CATATATTGTTTTATGAAATTTAATCTAGAAGCACTTTCCTTCAGTTCATTGTATGCTGGCCAT
TTTGCGCCTTAAGTTAAGTACTGGACCTAGTTCTTGAACCGCACCGCCATTTGGCCGAGTTTCA
GCTTGATTCAGTGTTGGCTTGACACTGTGTGTGAATGTCTCATGTTTCAGCTGCGGAGCAATGT
CCAGCACAATAGTTGTCAGCTGCACAATGCTAGAGCAATGGCCTGGGCTTCCACGCTGGCCAGA
CAATAAGGCCTCAAATCTAGTCTGCAATACATACTATAGGGATTTAAATAAAGTACTTGAATAG
GCCGCCCGTTTTCGTTTGCAGGGGTACTCTAGGGAATAGGTCTGCAAGTCGAACCGGTTTGAAC
ATAGCCGAGCATTAAACCATTTTATTTTTATTACTAAAATAAACGACAACTGGCTAAATAACGT
TTTGACAAGGCCAAGGCCAAAAAAATGATCAGAATGAATAAATCAATTATTTGGCAAGCCGTTT
TTGTGCTAGGAGCTATTTCGCCTAGTCTGTCACGCATTACTTCAATTCAGTGTTACGCATTGAT
TTCAGACAACTCAGTTGCCTAAGTTTTCCACTCATTAGTGTTAGGCTTTCAGCATGACATTAGA
TACCTTAAATAAATTAAAGTTACATTTGGTATAAAAAGAATTTGCTCCTAACACAATAGAAAGA

TTATCTTACGTTTACGTTCCGATGATTCTATCAGCTTATCCCTAAATCAGCTTAGCCCAATTAT
CGGCATAATTTGCCAGATTTTGGAGCCGCACTAAAAAACAGATTTCGAAAAGTATTGAGAATTT
GTGAAAGCATTCAAGGAATTAATCGGAACGTTTGCCTATTGACTACAACGACTGTCTCTCTGAG
TGTCTCCCTCCCTCTCTCTCTCTCTGTCTCGTTCTCTCTGTCTGGATTTAGTGTTTTCTTTTGC
GAGCAAAGAGGATTCAGCAGAGGATGGCTGTGGGGTGTCTCTGTTAGTTCATCTTATGCTGCTC
TTTGAATTTTTCGTGTTTTCCCAAGTAGCTTTCATTTGGGGAGCTTGAACTTGGCCAAGTCAAA
ACGCGTGGCGTGCCAAAGCTTCAGCTACAGCTGGATGTTCGTGTTGTTGTTGTTGTTGTCAGCC
TAATGTGCTTGTATTTACTTAAACGCACAAAACGGCACAATTCATTGGCATTGGGGGCCAAAGG
CATTGTTTTGGTCCTGGTCCTGGTCCTGGTCCCAGCTCTGTGCACCACATTAGCGCCCACTTTA
CTTTTGCTCAACCACTGATGAGCAGCAAATTGAATTTTATTGACTTGATTTGCGCCCCGTTGGC
CGCATTTCATTTTGTTTGTTGTTGCTTTATTTATTTGCCTGACATTTCTGACCATTTGGCTGGC
CTTAGACGTCCAAGCATTCTAGCCAGCTCCTGTATACACAATAAATTGATTTCTGATTATCATT
GCATAATAAAATGACTAAATCGGCTAGCGCGTCCGCTGCCCAACAGACGCACTGAAGGCCAGGC
AACCCCAACAGAAGTTTTAATGAAGCGTATTAAAATTTAATTAAATAACAATTTGCACAAATGT
AAATAAACACGACGCTGCTTATGCAAAGCGTGAAAGAGCAGGACTACTAGAACACTCCAGGAGC
ACGCTAAAGGCTGGAGGCTGGACGCTGGACGCTGGCGATTTAAGACGTGGGCGTACCACGGCTG
CCATGCCAACGCTCAGTGTGGCGAACTTTGGTCATTAAATTAAGTGCACACGACGAGCACGGTG
ACGACGAGAACGCGGACCACGATGTGACGAACCAAAGACGAAGATGACGCCGGCGCCGACATCG
ACGGCGATTCAAACGATTGTTGTGGTCCCTGTGGCTACGGGCTGCCCTCATAGACTACAAGCGG
GTGTCTATCAAATTGAAATCACGAACGCTGACATAAAGTCGCGTCTTATATATCCACGTCCCGG
CCTGGCATTTGAAAAACATAATATTTTTAAGCTTTTTAATACGTTTTTATGCTCTGCAACAATT
TAAATAATAGGCAGAAGAAGGCATCTTCAAGCCTATAAAAAAATATGCATTGAATATATAATAA
TTTAAATCTGGCAAAAATGTAAAATTTTAGATTAAAAAAAATTGTTTTCGGTTTGAAATACGTA
TATTTTAACATAAAATATGCAAAGAAAAGTTGGCGGTTTTTGGTAATGTGTGAAAAACGTCTCA
AATGCATTTGTTGTGTTAGATCACAGTTTTTTTTTGCGAGTTTTTCTACCAAGCGGATACATTT
TGAAAATTCGCATAAATATAAAGCAAGTCTTCGACCTGTTACTCAATTTTCCAGACACTGGTGA
TTTTTCGATAAGTGTGATCAAATCTACAGAGACTTCCGAACGATTTATTTGAAAACGAGCTTTG
TTCAGAAAACGTCGCATGCAACCATAAACTATCAATACTCCAGTTGCAGGCTTAATTTTTCCAA
ATTTTCCAAAACTACATAGGACATCAATATACTGGAAAAAACAATCATAACGAAAGAAAGATTA
TAATTATTGATTTTTTTACATTTTTATCATGGGTCATAAAATAACAATTATAAAAAATTGACGA
TTGCTGCTGGAGTTAAGTCTGCGATCTCCCGCTTGCAAGCTCGATACTTAGATACCTAATCTAT
ACAGGCTGTGATGTTTTTAATGGGTTTCATACCAACTTTTATTAACGCTTGGGTCGAATTTTCA
AAAACCCTGACTTCGTGCCTAAACTTAAGCTTCATATCCCGTAAGTTAAATGCCCTGCCAATAA
GTTCTGCAACTATTAAATCTGACAATAATTCATTTAATCTCATATTGATTCAGCATATTTTATA
TCGATCAATTCTTAGAGCTGTAACAAATGAACTTGGCCTAGCCTCTAGCAGTTAATGTGATTTT
TAATTTTCTATACAATTCCATTCTATGCACATACACATACACAGACACACATTCATCAGCATAC
ACGCGATTTGAGTTTATTATTTTCATTTTTGGGGATGGTCTGTGTCCAAAGGGTCGTGTCTTCG
GGCTGTGCTGACTTTTACGAGCGCATCGCTCATACGCCGCGTGGGCGGTGGCGCGGCAATTTCT
CGCGTGTGGCAGGTAAAGTCTTAATTGAATTTGCTTTCCGAGCGTTGGCTGGGAAACACGAGTC
GCTGAGCAGAGCTGCTCGCAGCACACAGCATTCATATGTATATATGAATATGCATACACATATA
TATTACACATACGTCACGTATTTCTAAATGAAATTGTGTAAATTTTTTATTAAATTTTTGAGGG
AATATTAACAAAAGGGCAGCCACTTTAAAGGGCGGCAAGAAATGAGAGAACTTAAAAGCCAGAG
CTTAATTTTCATTTATTGATAGCTTGAATAATATGACATTCTGGCTTACCTGGCATGGGACTTA
AGTCCGTCCACTTTGAGCAGCAAACTGTCCACTCAGCTATAAACAAGATTTGTTATGAATGGGA
AAGCTCAACTCAACGTAAATGTCAGAAAAAGGTATCGTGTCCAGCACCAAAATAGGAGAGCACC
TAAGCGGCTTGAGTTTGCCTTCTATATTGCCAACGAATTGGTCATTACTTGGACATGGTGACTG
CCTTCCTATCAGCATCTAGTTGTGTCGCATCTTTCGGGACATTGTATTTTCATTTTAGAATTGT
CTCAGCGATTTTCAGTTTATGACTCAAGGCAATAAAAACGCTGCCATTCATTTTTATCCTTATT
TGACAAGAGCACATTGCGTGCTTTCCGGCAATTCATCATATCGCCGTGGGCTCCAAGATGCGTG
CTCAAGTTTCGCCTGGGTTTTGCCAGTTGTCTCAAATTAACTTTAATAAACTATATATATATGA
ATTTTCTTCACAGCCTGCCCGTCACTCAGTCTGTGCTTTGATTAATAGTTTGGGGCATGTTTTA
GCATGTCAAAATGTAAAAGTTATGCTGGGTTTTCGCCGAAAAAGTTGTGGTTGAACTACAAGAG
AAATATTAGGTATTAAAATGCATATTATGTGTACTATATATAATGTGTGTTCTGCTTATTAATT
TTGACAGTCTTTTATAGATACACGTATACCCTGTAACTAGCAAAATGAAGGCATCTCCTGCCCC
ATTAAGCGTAAATATTCTCTATCTGTTTGTCGACCTGTTCGTCTTGAAGAATGTTTTGATCTCA
GAGCCTATAAGACCTTAAGAACTTACATTTTATAAGTGCACTTACTTATAGATTATATATGGAA

TATAATTATATATTAGACTTAGCTGAAAAGCCCATATAATGTACCAGGTCGTGAAAATCCAACA
CTTATGAGCATGATAATAATACTATATAATATATTTTTAAGTGTCAGAATTTGTAAAAATATAT
AATAAAAGCAAGTAAGAGGTTCTAGTCGGGAGCTTTCTACTTCTTTAATTTGCTCAAGAAACAG
GATGTCGATATTGATTATTATCGTTTGCTTGGAAACGGAGTAGGTTATCGATTATCGGACAAAC
TCGATTTGCGCAGGCGCTAGGAGGACCTACATCTAAAATTTCAAGTTTCTAGCTCTTATAGGTT
CTGAGATCCTTGCGTTCATATATACGGACGGACGGACGGACAGACAGACAGACGGACAGACAGA
CAGACGGACAGACAGACAGACGGACATGGCTAGATCGACTCGGCTATTGATGCTGATTTTTAAT
ATATGTATATACACTTTTTAGCATCGGAGATGTTTCCTTCTGCCTGTTACATACATTTGTACAA
ATACATAATACCCTTTTTATCCATTTTCAATGGGTTCAGGGTTAAATAATATTATTGTAGTGAA
ACACAAAAACATAATATGTATTTACATTTCTATAGTCATATATATTTTTCTATTAGTTTTTATT
ATTTCACCCTCGAAAATAGGTTATCAAATAGAGCAAAAGTTTAATAATTCAGAAGTCATACCAA
ATTAAAATGTTCAGTCTTATAACTTAATTTAATTAGGAATTCATTTGCTTGTTTATTGTTGTCG
TTGTTGTTCTTGTTGTTTTTGTGCTGTTGGCCAACTTGCTGTGCACACATACTTACTTGTTTGT
TTTTCTCTTGGCCCTTTCAGCCAATTTAATGCCAAGTTAATTTTTCAATTTACACGCAAAGTGT
CAGAGCAGTTTTTCGAGGAGGACGAGGGGCCGGCCACAAGGGGCACGCTAGGGGCAAGAGCAAC
ACGCATAGAAGAATCATATGAAATTGCAAAGTGGAGTTGGAAGGAACAACTTTGCCCGATAATA
GCTAAAATAAAATAAATAATAATAATAAACACAAAATTATAATAAAGTGAAAAAAAATAAATAT
TAAAAATCAACAACAACAAAGGTACAATTAAGCCAATTAAGCTCTAGCTGCTGCATAATTTGTT
AACAAAAAACCAGAGCAACAGAAAGTTGAAGATTTTGCGGCAAATAGGAGCACGGCCGACAAAA
ACGAAAAATATATGAAATAAAAATGCAAATTTATTTTAATTTGTCCCAACCGCCGCCGACGCCG
CCGACGACGCTGCCGCGGTGC
>Contig691:1..8756…
TATTTGCATTTATTTGAAATGCATTTTGTGTTGGGCCAACAAGTTGACAACAACTTTGCTGCCA
CTTGCCACCTGCCACTTGCCACTTGCCACCTGCCATGCTGCGCCGTAACGCGTCGTTGCCCGAA
CGCACGCAAAACTTTGGCTCGTCTACGTCTTGGACTTTGTCTGCGACTCCGACTGCGACTCTTG
TTCTTGCAACTTGTAGGGCTGTTTTAGGTCCTTAGCGGCACTGCGCCTGGCGCTGGCAGGACCT
CCTATCCACTCGAATCAGCACAAAATTGCGCTCACTTAAATAAATTTATGAAAATATGCAAGCA
CGCGTCGAGCTCGAGTTAAGTGCAACCAATTGTCGGTACAAGATTTAGGTCTGCGGCTGGACTC
GGTCTGCATTTTGGTGGTGGCTTCTAGCCACATTGAACAAGAACCAGGGGGTTACAGTTCGAGT
AGGAGAGAATGAGCATGATAATGAGAAAAAGAGGTTCGCTGAGAGACACAGAGGGAGAGTGGGC
GAGAATGTGAGAAGGCAAGAGAGTTATTAGAGTGGGGCTACAAGAAGCGGTAAGGCTTATCGGA
TTTCCGATACAACTTTCTTGTATCAAGTTTGCAAGCCGAATCGGCTTGAGTTCGAGCTTGTCTT
ACTTACATTAACGAATAAGCTTACCTGTCCAAATCAATTGTTATACCCACACACAGTCATCACA
CTAAATAAGTGGTAGGAGTACATATATAAATATATGTCGCCACAAATTTGTTTATTTGTGCGTA
TGTATGTAACAGGCAGTAGGAAGCGTCTCCGACCCTACAAAGTATATATATTCTTGATCAGCAT
CAATAGCCGAGTCGATCTAGCCATGTCTGTCCGTCTGTCCGTCTGTCTGTCCGACTGTATGTAT
GAACGCAAGTATCTCAGAACCTATAAGAGATAGATGTAGGTCCTTCTAGTGCCTGCGCAGATCG
AGTTTGTTTCCGATAATCGATAACTTACTCCGTTTCCAAGCAATCGATAAGACTCGATATCGAC
ATCCTCTTTTTTTTTAACAATTTGGTAAATATTAAGAGCTAGAGTCACCAAACATGATATGTTG
CTTCTAGAATATTATATATATGTCAAGTATCTTTCATTTTATACCTATCGCCACCTCCCCGCTA
CCACCACAGAGTTAATAACCCATCTTATATTGCCAACCAATTTAAGCCACAATTGTAATGCAAT
TTTTAACTTTTTGAGATAACACAAATATTCTCTAGTACAATAAGATGTACTGTAGAAAATTTCA
TCAAGATCGGTTAAGAGAAAACCAAAGTTATTTGTAACTGCGCACTCGGATGGCACAGCTAGCG
AAAACATCTAGAATTTATGTATACATGTACACACACACATTCAAGCGCGTCTGCTTCGCATTCT
ATCGCATTCTAACAGCATTGTAATTCTTTCTGTAATGCTATTTTAGTTCGTTTTTTTCTCAATA
ATACTTAATTATTGGTGTAGCTGTTGAGTAGAGGGTTGGTTTTCAATTCGTATGACCATACACA
CATGCATACACATACACATGCATACATGCAACCAATGAAGCACGCCCTAAATAAGTTTTAAATT
CCAAATATTTTTTAGATATTAGTATGAAGTCGGTCGGACGTTTCAAAATGAAAAGCTAAAAATT
CCATAAAATTCCATATTCCCAGTCATAGTGTGTACTCTTAATACATAGGGTAATTAGGGTAAGG
TATTCACCTGTCTAAATCCTACTTTTTGGATTGATCATCATACAATCAGTCAAACAGTCAGTAA
TATATAGAAAGGCATTCATGTCTATGTAAATATACATTTATAAATTATTTTCTGCTGCATACTT
CTGCATACTATTATATTTTTATTGCATTGTATATATTTATTGCATTACTTCGTTTACCTTTGCA
GCCCATTACTTATCCCTGCTATCGTTGACATAAAACCCGACAATTAGTTTGCATCTAGTTGGAA
CGTCAAAACGTTTTACAATGCAAATTCTCCTGTCAACAGTCGGAGGCTGAAACGCAAACAGTTT
CACTTATAAACCTGCACTTTACTGGATGTTGCTTGCATATTATTTTAATTAGCAACTGTCGCTC
AAAGAAAATACCAAAAGCAACAACAACTTATGGCGACAGCAGCAGCAGCAGCAGCAGGAGCAAC

AGCAACAACAACAAAAACAACTGGAATAAAGTGTCGCCTGCTGGTCTAGCCTTTGGGGCGGGGT
TTGGGGTAGGCGCTGCGGCAGCCCTCTCGTTAGTTAAGAGCAACAGCAGCTGCTGCTGCTGTTG
GCATGCATTAAAACTTGTGCTGGACAAGTATCTCACACCAGCCCTGCCAGACACACACACATAC
ACGCACACACACGCACACGCAAGCACTCAGGGCATGACTCGCGAAATGAGCGCTTAACGGGAAG
CAACGTTTTTACTTCAAATATCCTACTTCTAAAGAGAAAATGGGGTATACTGATTTATGCTGAC
GCTAGAGGCACCAAAATCTACAATAAATATTTGTGGATACTCGCTTCCGAATTGGGTTTGTTGG
CTTTTTGATGATCTTTTAAAGATCAAGCCAAACGAGCATTTTTACCAGTCAAGCAGTTTGCCAG
TCAAGTGCTCTCAGTTTTAGATATCATCAAGTTTGCTTAGTCAGATTGCTGATGACCTGTTGAA
AGATCTTACCAAAATAATTAGCGTAACGCTCACATGCGAATCCATCATCAACAAAGCTGCCGAA
CACTTAGTTTATGACATTGTTATAACACTGCAACGTTGATTTATTATTTTGTACTGTGTACTCA
ATTCGAAAAGTGCATAAATGTAAGTTGTAAAGAATAGAAGAAAGCATTTACGACCCCATAAAGT
ATATATATTCTTTATCAGGACGAGTGGAGTCGATCTACAAACTCTTCGATCTCGGAAACTACAA
AAGCTATAGACTTCACATTTAACATATAAAATATATGACTCATATATCTTACGCATACGTGGTT
TTCCCATAGAAGAATAGCTTTAAGATACGTCCATTATATAATTTAAAAAAAAAAAAAAAGAATAT
TTTAAAAAGTTCTTCTAGTTTAGCGCATCCCCCACTACTTGCTTTATACATTCTTGCTTTTACC
TATCGCCTCATTTGGCTTGGTGTTTTTGCATTTCGCTTAAACAGCACTACGCACACTTTCCCGT
CGCACACCCTCGCTTACCCCCCTGCCCCTACGGCGTTCTCTTGACAGAAAGGCATGCCGAGGCA
TTGCGTGTTGTGGGAGCAGCGTGAATCCGGTCCTTAATTTAATTTACGAAAAACAACTCGCTTC
CAAATACGAAATTGGTGTTTAAGATAAAACGCTTAATACATACTTTCGACGCTGAATTAAAGAA
ACTGAAGCGTTTTTGATTACATATAATTATTTTAAAGTGTTTGATTAGAAGCTACCCTGTTCTC
TGGAAAACGGCATCAAGTATACGTTTTTAAACTACTAAAGCATGCAAAGCCTTAAGCCTGAGGG
ACTAGTTTGCATAAAAACAGACAAGGCGGACATAACTATATCGTCTTAGCTGTTCATGATGATC
AAGAACACACATATGTTATTTTCTTTCACTTAATACACTCAAGGGTATAAGGAAGATGAATGGT
AAATCGAAAGGGCTTTAACGAACCAAGGTTTGAGACCAGTCTTGTCAAAACTAGGTTCACGAAC
GGAACCTTAAAGCGATGCATTGGTTTAAAGCGGGTTTGAAAACCTTATTATAATATTTGAGCAA
ATTTCCAGCCAGAAGCTACACTGGCACCCTGTAGGACGTCTGGTTGATCTGGAAACTTGGCATA
GGCTAAGCCCAAAAAACCCGCTTGCAACAAAATTCCATAGAATTCTTATAGGCAAAAAACTGAA
AAAAGCCATCCGAATTTAAAAGGGGGGTTAGTTGTAATAATACTTAACATTGACCCAAGACGGG
TTCATGTTGGCCTCGCCACATTAATTGGTAAACTTTGCTCAAGATCTAAACAACTCTTGAGGCT
AAGCAAGTATTGCTGAAATGCCCATCGTGGACAGTTTGGGGCTTGGGCTGTGGCATTGCAGATT
GTGGGGTATCATTTTGTATGGCTGCCAAATTTAGCTAACAATTTTTGGGCCAGGGTCGGCCACG
AGGCAAATTGGCCACATACCAGTGTTAACCGAGTATGATTAGATTTTGCTTTTAAGCGTTTTAG
CAGCAGTTGTTGAGCTTGCGGGCCAGGCAACGATGCTCTGTCATGGAGAATCCAATCAGGTGGC
TGTTGACAAATGGCTTATGGCACGTGCCTATGCCGCTGCTGTTACGCCCCTCCCCAGCAGCCCA
TTTACCGTCACAAAAGGTGTGTGAAAACTGGAGTTTGCAAAACCATTTGCCACTTAAGCAAAAG
TTTCAAATACAATTTTAACTAGAACAACAAGAGACAGCGCGCACGACACTTTCCGCGGGCCAAA
TGCAAAAAGTAAGCTGTGCCCATGGAATAGATGGCGGGAACGGAGGAAGGTGCCTTCAAGCTAG
AGCTGGAGTTGGAGAGGCTGCCGCGAAAACAAATGAAAAATGCGCAACAAAAACAAAGAAAAGT
CGAACAAAAAAACATGCTTGAAAATATGAGTTTCTGTGTATGCGAAGAGTATAAATTAAGTTGA
TAAAGTTCACTTGACCCCAAGTAACGATCCCAAGACATGCCCGAGACCTCCTGCTAGCCCTGGA
GTCCGCAACGTCTTCAGTTTTATTTTTTAGCAGCAGCAACAATAAACAATACATTGTACAATGT
AATTTTTGCAAGGCTACAACAAAGCCAAATGAATAAAATAAATAAGAAAACTGTCAGGTGCCAA
CGCAGTGGTCAAGGTGGCACGCCCACAGAGCCCACAGCGTTCCACTGCCACAGCTGAGCTGAAT
TAAAAGTTTTTAACACATATTTACGAGCAAGTGACAACAAAAAAACAAAAAACAACGAGCCAAA
TGCCACTAAAACTCTGGAAAACAAAAAACGTCGCGACGTCTAGTCAAATTGCAAATGATTTGGC
TGAAACCCAATCAGCGGGTGGCCAGTCGAGCAGCGCACGGATGGGGGCTAGCTGGGTGTGTGGG
CTGTCACTGTTAGTCTGACTCTGATGGGGGCCATTGACACATGCCACGCGCCATGACACCACTT
GCCAACAACACGATTAAAAGGATCATTCGCTTTTTACACTTTTGGGAATCTGCGCCTTACTGAA
AAATTTTCGGAACGCTTTATTTTGAAATAGTTGTATTAGAACGAGTTAAGTGGACAGCTTTCAA
TTGATTAATCAAGAACGATGCTTCGCAAACGCAGAAAATGCCGCAGGGGTAAGCACACAAGAGT
AGGCTTAGGTTTTGCTAGCTTTGTTGCCTTTTTACTGCTTATAATTCTCAAGTGGTTATAGTTT
CAAGCTGAGTAAAAGGCCATGCGAATCGAGAGTTGATTGCAAAGTCTGCTGATGATAGAACAGG
GGGTTTTACACATAGTTCTACAGACAACCTGCTCAATCTTGGCACTTATACTACCTCAAGCCGC
TCGGCTATTGACTTGACAAATTTCATTTACTAATATTGTAAATAAATAAGTGGGCTGCTTACGG
TCTATAAATTATAGCAGATTAACTACCTAGAATGTCAAGATAACATTTATTTAATAAGAAAGCG
TGTTCCCAGAGTGAAGCAGAGTACATGGGCCCGTCAGACAGCAAGTATATCTATCTGTCCTTCG

GACACTTGTTGTTATATAAGTAGATACAAATCTGGTAGAAATCTATATCATATCCAACTTTTAT
GTATCAGTCACGGCCTAACCCTACAGAGGTTATTAAAACTTTGTCGCAATCTGTGAAACGCATA
AAAGGAGAAGTCTGCGGTTGCGAAAGTATATATGGGAACATATATGAATTTTTGACCAGTATCA
AGAGCCGAGTCGTTATAACCATGCCTGTTTGTCTTTTTCTGCTACTCTGTTCTACTCACTCAAA
AAATTGTGTTCATCAGTATATCTTCACCAAATTAAGCATTAATTAGATCAACCATGAACACTCG
TATCAACATCGCGTGCTCAATAGAAAGAGTCAGTCGAAAAACGGAATATTGGAAATATCTTTGA
TATGTTAACCATAACAGCATAACTGCAAATCCGTTTCCGTTTCAAATCGGGCTTCTATATCATT
TTACCTAATGAAAGGTTTTCTATGTCATATGTGTATAAACCATTATAATTGTTACTATTATTAT
TAAGTTTTTTTTTTATTATTATTATTAATATATTTAGTATTATGATCAGTATTATTCTGATTAT
TATTAGAAATTTACTGTTATTATTATTATTAGCTTGGGAGTCCAGCAAACTAACTATCATTCTT
TGGGAAGGATAAAACTATATTAATAGACTGGCGTGGACTGGAACGTCGTTGCACAGTGTTTTAC
GTATTATTCGGCAATATATAGTTAAAAACTATATAAACACTCACAAACTGTTTGCGGATGTCCG
GTGCTTGGCAACACGGCTAGTTTTGCATTCGACGAGTAGTCGGTTTTTGTTAGTGAAAGTCATC
CTGTGCGCGGTGGCGTATGCGTAATGCGTTTAGAGTTAATTAAATTTGGTCATGTTTTGCGTGT
GTGTGGTATTGGTGGAGAGGGATGCGAGAGGGAATGGGACTGGGGGCTTGGCAAGTGTCCAAGC
GGAAAGCCAATTCATCATGGCACGCAGCTAAAGTCATAATCTACTTTAACAACTAATTGCCATT
ACTAAAATTATCATGATTATGATGATGATTAAGATTATGATTATTAAGTATGGACATCCTTTTC
AGCAGGCAAAAACTTTTGGCTAATGGCCTGCCTCAGTGTAAACTTATTCCTGTTGCCATTTCGT
TGAGGCCTAGATTTTGGCTTTGGCTTTCGCTGTCGCTTTCGCTTTGGCAAACCAAAGCACTTTC
CCTACAATATTTTACAACTAATTAGGGATAATTACCAGCCATTACACAAAAGCCTTTCCAGGAC
GGTGGCAAGTTGAAAGTTGAAGACAACTGACTCACTGACTGACAGGCTGACACATTTTTGGGGC
GTGGCTGTGTCTTCCGTTCAGCCCCTGTAATTTGGGCCACCTTAGTTATTATTACTGAGCCGTT
GGCATACAAGGCGTCAAACGTTCCCCAAAAAGCCTTTGACTAACTCCATTGAGTACTTCTCATT
TGTCTGTTGATACAGGAGCAAATGTCAAAGTGTCAGCTATCACATCCTGTTCAAAAATAGTTGG
TTGGAAACAAGCATCAAGGGTTGTCATTTATTTATGTCTGCATTTCCTGATTATACTCAACATG
TCATACTTCTGTTTATATTTTCGTAAAATTGCTTAATTTTATGATAAAAAATTTTATTACAATT
TTAAAATACAGTTTGTAACCATACTCTGTGCCCAGGAGAAGCAGCGTGTACTGTCCATCTTGGA
GGCCATTTATCTCTCCCTCAAACTATGTGAACAACATTTATAAATAAAGGCGAGTGCATACTGT
GAAGCTAGGAAACAGGGAATCTGTTTATCGAATAATCCCAAGCTGGAACCGATCATATTGACTT
TCTAAATAGCACCAGCTATCTTCTGGTAGGACAATGCTACACCAGGCATTTAAACTCAGATAAA
CTAACATATGAGACTATGTCTTTCTTAAAAGATACAATTATCTCGTTATAGCTAAGTAAATATA
TCATCAGATTGGTTAAAGTGTTGCAGCAGTATGTGATTTTCACACAATGAAGAGCCAGCAAAGG
ATTATGGCATGTCGCGTGTCCTTCCTGGGAGGTGATCAAATCCGATTTACAGTGACACAAACAG
CTTCAATAGCTTAGCAGAATAACAATAGCAATAATAATAGTACATGTGCATGGGTGCACTCGCA
GCTTGGAGCTCGGAACTTAACTGTTCTAAAGCGGTGAAATGCACTTCCCTTTAGTTTTTCGCAT
GCTCAGACACTAAGACGCAAATAAGTTACTGTGTGGGCGTGTGAGTGTGTATGTGTGTATGTGT
GTATGTAGCTGCCATGTTTTTAATCCACTTGAAGGCACTTAAGTAAGCTTTCCTGCAGAGCTGG
CTGGTGTGTGTGCTTGTGCCTGGTTGCGTGTGTACCAAGTGCTTTCGGCTTGCTTTAAATTCAT
AGTGCTTATGAGTAAATACAAGAGTGCTCGAATAGAAGATACTCTATACTTAACTGCAGCCATC
TGCCTAGAATGAAAACAAATTTCGCTGACCACAAATCAAGTTTTCATACCATTATCTATTTGTT
CCCTCTGTCTATACACAATATTTACAACTGTTTGTGTTGACTGAAAGCTAAATTCAAAACAGTA
GTTGTCTTTCTGATTCAATTTAAGCTTGGCAAATGAGAAAGGCATTTTGTGAAATGAGAGTGGA
TAAACTTGCTACTACATAGATTAAATGAAATTGTTTGACATATCTTACCGATTTCTGACAATCC
ACTTGCTTATAGTATCTATGGTTGATTCAGCCAAAGTTAAATTTGCGTTGCATGAATCTTAAAC
ATATTTTCCATGCAGTCTTGTGAAAACATTTCATATTCTTCTTTCTTTTTCAGACAGAGCATAT
CTGAGTAGAAAACG~~GCCAAATCGAATCACTTTATTGTGTAGCTGCCATTCGAGCAAAACCTAAC~~
~~TCATTTGAGTTGTACGAAAAACACTAAAAACCTCCGAGTTAACCTGTTGATG~~

## ***D. americana* SF12**

### Distal breakpoint

>SF12 Contig680:…34,092..36,004… (GJ14731[-](CG8206) – GJ18832[+](Crag))

~~ATTTGAGTTACTCATAGCTGGTTTGACCGGCCGATAACAAATTATATTTAGCCGTCGGCTCTCAT~~
~~ATATGTAAATAACAAAACAAAAAAAAAAAGAAGGTAAAATTGTTGTTTAGGTAGGTTGTTTCTC~~
~~CCCTCTCTCTCTCTCTTTTCTTTCTCCCTTTCGTTTGTTCCGAGTGTTGACACCGACCACACT~~
~~GCATCGCTGTCCTGGCTGGAACTA~~GATTAGTTCACCGTGCTCGTTCACTTTGATGATGCGCACT
CGCTCCTGTCGCTTCGATAGCACCTCATCGATATTCATGATGCTGCCATCACTATTGGCGAACA
GGCCCAGATTGTCCTTGATCAGATGATCGACGGCACAGCCATCGCACTGGAGTATGACCACGCC
GCTGTAGTAGGCCTCCTCGCTGATGGTCTTCGTGTTGCGCGTGTTGCACAGCTTGCACAGGTAG
ACGATCTCCATGTGACGCTGCATGCGACGGAACCGCTTCAGGGTTGTCGGCGACAATGAACCCT
TTGGCTTACCTCCAGCGGCATTGCCGGTTATCTCAAACGGTTTGGCCGTAAAGCGACAGGCGTC
GAGTATGGAGCGCGGTATCGAGAGCGCCGTTGATCGTTGGGCATCCTTCAGCGATTTGTCCGCA
TCGGCATCGGACAACGGCACTGGCGCTGGTTGTTGGGCTGCATTGAGGATCGCGTTCTGTCCAT
TCTGGCGTAGATAGTTGAGGGTACGCGATGTAAAAATATTGCGCAATGCATTCAT~~TTTACATCG~~
~~GCATATTCACGATTATAATGGATACGGATGACGATATGGAGTCGGCGGCTTTATGCTACACCA~~
~~AAATACTGCCGAACTGCACGTCTGTATGATCTGGTTGTTACTAAACTGTTTTTAGCATAAACAA~~
~~ACGGCGCAATTTGCTCCAGCGAGAGCGACCACGAAGGCAACACTATC~~AAAAGCCGACTTGCGAA
CCAAGTGATGGCACTATCGATAGTATCGATAATTAAGTAACGCATACAGTGGTGAAAATTGTGC
AGAAGTTTGGGTAATGCTTCGTTTGTATTACTCTACAAATGTCAACTACAAATAAGTTGGGTGT
GGTGTCTATTCTACTTGTTTTTTTCTCAAGACTCCATTTCCGGCGGTAAAACTTAACTTCATAG
CGTATTTTATTAACTATAAGCAATTAAATGTTGCCGATAGCCCAGAGGCAGCACGATTGTTGAG
TGTCGTGCAGCAACGATACTACTTTACGATGAATTTCCGATCGTTTTTAACTATCGCCAATGCT
TTGAGATGAGCGAATGAGAGCTGCTAACTATCGATAATGCATCATTAAAAATATCGAATTTTCG
ATTTGGCGGAAGCTATTGTGTATTTAAAGTGTGTGAAACTAAAATACCAAAACTACTATTTGCA
TTTGTAATATATATTTTTCAATAATTAAATCTAAATGTAATTGGAAATTAAATTGAATGTTTTG
TAATCAATGAAATCCATTTAATGTTGTTGTATAATAAATTTCAAAAATAATAATATTCTTACGT
TGATGCAACCGTATTTTATGGGTGTTAACAATACAACAACAACACAACTGCAATCAATAATGAA
ATTGTACTTTGGAGGTTAATTTATGGTTGCACAAAACTTACAAGTTGAATTTGTTTCGCTGGAC
GTTTGGGTTTCAGGAGTCTCACTATTAAAAAATA~~ACATTAATAATAATAATCGATACATATACT~~
~~ACAATATCGAAATTGCATGTAAACGATGTGCGATAAAACAACAACTCTAGCTTTCAATTGATTA~~
~~CGATTTCGCATGCATTAAACGGCTGCGACGTAACAAATTGGACAAAAGACGCGCCGATAGTTAAG~~
~~GGCACAAAGTCAAGACAAAGTGCAAGTGGGCGAATGTCGCCCGCCTACATATCGATAT~~

### Proximal breakpoint

>SF12_Contig3223:…9569..1 + Contig18389:851..1+ Contig8501:3179..1 + Contig1707:22782..1 + Contig2813:13975..5192); (*GJ14858*[-](*CG15896*) – *GJ19325*[+](*Asta-R1*))
Dark blue sequences represent overlap between adjacent contigs that did not grow

AGATTGTTGGTGAGGAAAACACTGGCATTGTTTTGTATATACTGCATGGCTGGCCTGGACCAGT
TGCGCATGTGCTGGCGACCCAGGACGAGCACGCGCTTGCGTCGCTCCTTGAAGTAACGCACCAC
GGTGGCCAGCAGTTTGGCCAGCTGCTGGGGCGGCTTCTTGTTGCCCGTGGAGTAGGCCACATTG
AGGCCATCGATGACGCAATCGTAGGGCGCAGTTTGCTCCACATACTGCTTGAAGCGTGCCACCT
CCTGCGGCGTAGACTTTTGGAAAACGTCGTTGCGTATGAGCACCTTGGCCAGAAAGGATTCGCG
CAGCTCGGCGAACTGTGCATCGTTGATGGCCACATGCTGCAAGTGCTGCTGGCAGGCGCCACAT
TTGCCCATGCGATCCAGTCGTGTGGTTGTCGCCTGCACTTGCTGCGGCAGCCGCTGCGACAGCG
CCAACAGCTGCTGCGCGACCAAATCGCTGATGACAATGTCGTGACGTTCCAGAAAGCGCAGCAG
TCGCTCCAGCTGTGCACCTAGCGTCTGCGCCTGCTGTGCACTTTGCGCCAAGTGCGCCAGATAC

ACCTCACACTTGGGCAGCTTGCGCGTCTGCAGCATCTCCTCGAGCAGGCGCCAGGCCAGCTCCG
GTTGTTCCGCGGAAAATGCCTTGGCTGCCAGCGTACTGTAGGCTGCTACGCTGGGTGCACTGGT
CACCTTCATCATCTCCAGCAGTGGCACAGCGCGCTGCCAGTGCTGCTCTGTTGCCACCAGGCCG
TGTATCAAATGCTCACAGCTGCTGGCATCCAAAATCTCGTGGGCGCCCTGCAGCGTATCACAGA
TGTGCACAATTTCCGCCTGCTCCTCGTCGCTGAGCGTGCGTGCTTGGTAGGCGGCGTTATAAAC
GCGCAGCAGACGACCCAATGTCGCTGCATTGGGCTTAATGCCCTGCGTCTGCAGATAGTTCAGA
TAGCTTTTGGCCAGCGGCAGCTTCTCCGCACTGCTGCACATGCCCAGTACGACGGCGTCCACGT
TGCTGCCATTGATGTATTTGTAGCTGTCGGTCAGTGTGCTTCGCACCCGATGCCAATCGTCGTT
GCTCAGCTCGCTGCGCCGTTCAAAATAGTCAGACTTCAGCTGATCCAGCTGATCCGAAGGCACT
GCACCCAGTTGGGGCCTTCGCTTGTGATGACTGGCCAGCAGACGGTATGGCGGTGATTGTGTCA
GTGCTGTCCCAACGCTCTTTGTGATCACACTGTGGCGCAGTTGACGCAGTATACGTAAATTGTA
CAT~~TATTCAGTTTGTTGTCAGTTACACTTACTTGTTGCCGGCTCGGCAA~~TCGAAAGACTATCGA
TAAGCACATTTAACAAACTATCGAGTAGCAAGGCACATATGCTACCTCACTTTGCTATCGTTTT
CAACACTCAATGGAAAATCGTATAGCCGATATTTGTGTTTGCCTCCACGATAAGATTACAGTTA
GCCTTTTTTTTTAAGAAAAGCTGGTATTCGCTGGCTTTGACACTTTCCAATCCATTTATCATAT
TGAATTTAAGAAACTCATATTGATAATCTAAACAAGAATAGCTTCAAATATAGGTGTTGATTAT
AATAAATCGTTATCGATAGTTGGCAGCTATTATTTGCTCATCTCGAAGAGTTGACGATAGTCGA
AAACTATCAGATATCGGTGCTGCACGACATTGGGCAATCGTGTTGCCAATGTGTTATTAGGATC
TTCTAATTGCTTAAATAAAATAATTTCAATTGCTTACCTGTTTTGAAATATGCAAAGCTTCGCG
GCTGGATATGAAATCCTTTTAAGAACGTAGAAGAATAGACACCAACCCGACTCAATTGGACGTT
CTGCTATATCTAGGATAACCAAAGCAAAGAAATATCAAATATACAATTACCGTTATACCGTTGG
GCGGCACAAGTATTGATATGAAAGTTATCGTAGTCGGTTGAGCGCCCGTAATTTCGCATTTTAA
ATTTGGTAGCAACCTTTTCTCTTCTATAACACTTAAGACTTTTGTGTTTCGTGTACTATTTTAT
TACAATATTAAGAGACAAATCGGCTTCTTTTAACCGCGACCCAATTAGTGCATTGCAATTGTAT
TCAAAGTACTTGGCTCTGTGGCCAAGCCAAGCGGAGCGTAACCGGCCCAAACAATGGCGACGCA
TGCATTAGACCTAATTATACTTTTTATGGATTTAAGCCAAGCTGCTGCTGTCTATTATTCCCAA
TTTACAATGGCCCAGTTGGTGGCGACACTTGTCCATTGAGCCAAGCCGCTCGGTTTCATTCATA
ACAGCAGTTGGCTCGAGTTTAAACTCAAGATGAGCGCATTGTTGACTGCGACTCGCGACTCAAA
TTGTGCGCAGCCAGTGGCGCCTTAAGGGGGAGAGTTCCTGTTGCCTGCTGTTTGCCCCCCCTGA
GACAAGGACTCTGCTGCGCAGCTTGACTCGGTTTTGTTTACAAATATGCCATCGTGCTTGCCGC
TCGTACCGTAGTCCTTTGAGATTTGTATGCGCGCCTGTTGCGTGCGCCCGTTGGACGTTGTCCT
AATGGACTCTGCTGTGGCCTCAATTGCTCTGGTTGCCCCGATAGCCTGGCTGCTATCTGTTTCT
GTTTGTGATAATCTAATTATGCGGTCAGCCCGAGCTGGTTATCAGCTGTGCCATTGCCATTTTA
TTTACTTCTAATCAACTGGACAGCCCGTTGTCCTTCGTCCGTGCTCCGGCTGGCCATTACAAAA
TAATAATATAACAAAGATTTATTCACAAATACCATAATAAAAATCGCAGCCAAACAATTAACTA
AGCCATTTAGGGCAACAAATCAGATTAATTAAGAACAATAGCCAACATTCAAATGCCAAATGGC
ATTTTGCGTTTGCTATTTGCGACACTTCTCAAAAATATATTTATTGATTTAATATTATTTCTTA
AGTTAACTAACGCTAGAATTTGCAATGTTTCTAATACTTCTTCGTTGCAATGCTTACAAAATTT
GTTCTGCGTTGAGCAGGAAAAATGTACAGCCTTTTTTTTATTATTAATTATTTTGTAAATATTT
ATAAGATTCGAAGTTCTTAGCTATAAAATGATTTGAAATATTACACTTGAAAAACAGGTTTTTT
CGAAAGTTATAGATTGATCAACCTTCTAAAAATATATAGAGTGCATGATCATTTTTTATTATTT
TCCTTTTATTATTCGGCATTTTGTGTCTGTTTGAATATGAGCTCGTTTAGGGAATTGGAGTTAA
CTGTAAAACTTAGCAAAAGATTCTATATAAACTATATTAAAGCTTCTTAGCAAAGGACATGTGT
TAACATAATTAATAACTATAATTTTGAAATATACACAAAGCAAATGTTTAGGAAAAATAATAAA
ATAAATTGTATTTAATAATATTTTTATATTATTTTCCCATTACTCACAGCCTTATATATATCAT
GACTGATTATGATGCTGGAAATGAGTATAATAAAAAACATTGAATCTTTAATTGTTTAGTGGAA
AAAGTTCTAAAATAAATGAAAGAGAAGAAAAAGAAACAGTTTTATGCGTTATATTTCTTGCAAA
AGGGTGAAAGCAATTTGTTTGAGGTTTTTCATACATACATACATTATTGTAAGTATTTCAGAGA
ACAATTTTCACTGCCAATTTTTTTTTCTTTCGCATCATGCTTAATCTTAATTCATTTGGTTTGC
TTTTCGTGTTCCTTGTCTCAGGCGACAATGTCACTCAGGACATTTGAGTGGGCGGCCGTTGCGA
AAGCGCTCAGAGTGAAATGGTGAAAATAACTCATACGCCGTGTCGGCCGTCGTTGGATGCGTGT
TCGGGTTTCAGTTCGTTGATTCTTTGGCTCTGGCGATACTGCTACGGGGCGCTAGGATTGTGAT
TTGAATTGTAGAAAGCGCGCAGTTCGCATGCGAATGTCCTGACCGACGGTTGTCTGGCCGGGTT
TCAGCCCAGCATCCGAGCAACCGCATCGTTTGAAAAGAATCTTCGAAAATTGTTTTTGAAAATG
TAAATTGGCCAAGTAGCCACGAGTACTGAAAACAATCAAATATAATAATATTCCTAAATACATG
ATTGTATCTGTGAAAGTTTTCCTAATTCTTTATTTTTTTTTTTGGTGCTGTAAAAAACCATCA

TCAACAAAAAAAACGGCAACAAAATTAAAAGTGAAGAAATAACAAGAAATTGCCATAAAGTTTA
TTCAATTTTATAAAAGAATCTTGAGCGTGGCAAATTTCCGATTTGTGCTGATTTTCGGCTCTGC
GTCTATATTCAAATCAAAGCAGGTGAGTGCACCGCATAATTACCGGAAATTTAATTATGAGGTG
AGGCGTCGACTTTGTAAACGGTAATTGCATTACCTAGTCAACCGCGCTACCGCTTCTCAGCCCG
AAATCATAAACAAGTAATACGAAAATGTTGCTGTCAGAAACTCCTTACCTCCATTTACAGAGTC
TCTGCTTTAAAATAAGACAGTCAACTCTTCATAAGAAAGTTTAGAGTTTCGAGCTTGAGAAGTG
TCTATGCACTGATTTTTACTTTGAATACAGTGTGAAAGAATACCTTTCGAGTTCTCGAATTACA
TTCATTCGTCGTTAGTTCGATTCAAATTAAATTTTGCGGTTACCCCAATCAAAGGCGATTTGTA
AGCAACTCAAAAGGTTGAGCAATGTTTCATTTCTTTTTCCCTTTTATTTTTTTTCTGTGTGTGT
CCCTAATTCAATTAGGTGCCCACCAATTTGTACCGCTTACATTTGTGTTAGGGGCAAGGGAGCT
GCCGGACCTCTCGCCGGACGAAGAGCTTCCCGTTGTGGGCGTAGACAATATCAAAGACAGGAGC
AGAGCTATTTAAATTCGCAGACGGAATTAAGCTCATTAGCATATTGTCGCCGGTCGGTCATCAA
GTTCACGGGCAGATGTTACAATTTCCCATTTTCCAGTTGCCAAGTTTTCCACTTGATTGATGAG
TCCAGTTACAGACCAAAACTGCCGCCGCCTCATACTCATACTCGGCGTCACGTCTACAAATTTA
CCTGCTGCAGAGCTAGAGAAATGATACTCATTAAACAATAAGATGAGTTTGCCGTTTGTCCAGC
GCCAATCAAATCGCATGTACTATATTTTCTTTGTTCGGGTTTCAGAGCATTTTAAAACAAAACA
TTTGTGACCAAGCAAACATTTGTATTGTATTTATTGTTGTGTGTCGAAAACCAGGCTGCAATAC
GTTTTCGAATCGAACTAGTTCGAAGCGGAAACCTTAAACCAGGGCAGTTGCCCCAAAAGCTGCT
GCAACGAATCATAGTGAGGCAGACTTAGCCCAAGGGCATAACAAACTTGCAATTATATGTTGTA
GATGTCTGTCTTGGGTAGAGAACAAATTTAGCCAGCAACTTCTACATGAAGTTATATTGTCAAA
TTTGAATAAAAAGTTTAATTGTATGAATCAAATATGAAATATATATGACAAGCTAAGGCTTAAG
CCAAAACCAGGGCTGAAGAATGTGTATAGCTGGTGTAATGAACCGAAGCTTAAGGCTGGCAGTT
GATTCGAACCATAAACCGAACCGGCTGCAGACCTGTTAAATAATTTGAAAGTGAATCAAAACTG
TTTGTACCAACTGACTGAGTGACAAATTGATCAAAAGTAAGTAACTGCATTTTAGTTTTTGAAA
TTTACCAAAATTGTATAATGCGATTTTTGTGATGAATATATAAGAAAAGTCTACGTTTTGCCGA
TAGCTTACGTCCCACTTTAACGTTTCGCCAAAAACCTATTTTGTTTTCAATTATTTCTTGAAAA
TAAGTTCAAAAGTGACCGATTTTCAGCCAATCGAACCACTGTGCATAGCTGGACAGTTTCTTTG
TGGCACACTCTTTGGCTCATGTGGTATTAGGAAGGATTTTTGATATGCTCCAACCGCAAGTGCG
GTAAATGATGGCAAAAGGTTGCAGCAACGGGCTCGAACTCATGTTCAATGGGGATCAAAGTAGT
AAATTAAAGTTTTTTCTCGGGCGTAAAATTGATTGCAATGAGATGGAAACTGGCAAATACTCGC
TAGCTTATCAACTTCGTATTATGATAATCTATAATTTTACTGCTCAAAAGAATAAAGTAATTCT
CGTGAAATGCTTGAAAATCAACCTGACGTCGGCTTGATAATACCGGTTAGCTTGAGAATTTCAA
AACTAGACCACCAAAGTAGTTGCGTTTCAAAGCAAGCTCAGGCAATTTCAGAGAATATAAACTC
CAAGTTAAACTGATTCTGGGTAAGAACTTTCAGCATTCTCCCTTTCTTATGCGCTTAACATACA
ACAAAGTAAAATATTTCAGTAGTTATGATAAAATGTGAGTAAGCGCGACTTGTTTCTACACATT
TGGAATTGCAATCTTTGGCAACTAGGGTGAGCCAACCTTGAATATTATATTTTTTGAGTAAATT
TCACACTTTCGCCTTTGACACGCGATTTTGTTTCATATAATGAAGAAAAGCTGAGTAGCATGCG
ACCTGCATTTAATTTCAATTCCGACTTAATTTGCATTTAATGTGAGGACTTTTCGTCGAAATTT
AATTGGCCTAATTAGGTGCGACATGGCCGTAAATGTAACGTGAACATTCACGCATTAAGTGATT
AAATGAAGACTGTGACCCATGAAGCGAAGCAACTGAAACGAACTGAAGTGTAGTGAAACAAATG
CGAGATATGAGAATTGCGCATTGAATTAATGAGGCAAATGTCAAAATTGAATGGGTTCAAAAAT
GTCTAACAAGCGCGACAACAAAGGCAAAGTTGCTCCAAGGGAGCCGGGGCTGTCCTGAAGAAGG
ATGCTCGTACTGGTTAAATGATTGAGCTAGCCGCAGTCTTTAAAGTTTGTCCGAATGCTATGAA
AGAATGCAATTTTTATCAACTTTTCCATAATGTTTTAGCAAGAATTTAACGCTTCAGGCAAGGC
AAAGCCAGCATACCCCCATCATAAAAAAAAAAAAGTAAAAACAAAAACACAAGCAACCTTCTACA
CTAAGGCAAACAGACCAAGATAAGGCATTAAAAACGAACAGCATAGGCTTGTACGAGCTGCTGA
TGCCCTGTAGCTTGGCATGCCGAAATATCTGTTATGGCTGTGGGCGTGACTCAAGTGTCATAGC
TAATATACCTGTCTTTGGCAGCCTTATAGGGTATGCGTATGAAAAAGCGTCGCAAAGCGTTGAC
TTCTCTTGAGTCTTGCATCACTTTGGAAATAGGAACGGAACGGAACCGTAAAAATGCCATAACG
CACAGCCAAAGCCAGTTGGAGCATGGGCACAGGTGGGCGTGGCATGCTCGGCTCATCTGATGGC
TGCCGAGCAAGCCACAACCCGTTCCAACCCATTGTCTATCTCTTCCGCCACGGCCTTCCTGTTA
GCCCAGGCAGTCACAATGTTTTGCTTATTGTCCTGCTGTTAGTTGGCATGGCAAGTGGTGTGTG
TGTCCTCCCAGCAATTGCAGTTAGATAAGCCAAGCCAGGCCCTGCACCCACCCCCAGCCCCCTA
GTCGACAGCCTGCGACCCCTTTGTGGATTTTCGCTCTCAGCTAAGCAGCTCAGCCAACAAGAT
GCTGCATACATTGTGGCGCCAGTTAAAACAAATTTATATCTACGATATACATCTCAAATGTACG
AGCACAAAGAATTCCTGCGATTGCACTGAAACTTTGCACTGACATGTGGCTTGGTCAAGAATCT

GCATCTTGGCAATTTTGGGAGCAAAAAAAAGGTATCTCAATTTTGGTGCATAATAAGCTACATG
TTTTATGTCCTCGCATTAGTTCAGACTTCGTACAAAAGTTTCCTTCTTCTTTTGCCTTCACTTT
GAGCTGCAATGGTAAGAATTTTACGAAAAAGTTATATATGTACTGTTTCTTTAAGGTCAACGAG
CTGGTTAGCATTTTAGCCAATAATCAGTTTCTTAATTATCCGTATTGACCAGCTTGCAACAAAT
TTGAGTTGACCGAAGACTATAAGGAGAATGTTTAATCTGATATCAAATCAAATATAGTTGAATT
CGAATTTTCCTTAAATACGTAAATATCATACATTTTCTACAAGTTATATATATTTTGCTGTAAA
GGCTCCCATATCCTAAAAGCCTTCTCACAACAAGAATATAACGGTGAAGGAAGAGCCTAACAAG
CAACTAATTATTACATATATTGAAATGACAAAATTATTCATTATCATTATCCATTTTAAACAAG
AGGTTCTAGTCGGGAGCTCCCGACTAGGGGATACCCTGAACCCTCTTCTTCCAACATCAAATGC
ATATATATATATTCTATTCTAGAAGCTATATGTTAAGTTTGGTGACTCTAGATCTTATTATTTA
CCAAAATTGCTCAAAAAACAGGATGTCGATATCGATTTTTATCGATTACTTGAAAACGGAGCAA
GCTATCGATTATCGGAAACAATCTCGACCTGCGCAGGCACTAGGAGCACCTACATCTAAAATTT
CAGTCTCTAGCTCTTATAGGTTCTGAGATCCTTGCGTTCATTCATACGGACGGACATGGCTAGG
TCGACTCGGCTATTGATGCTGATCAAAAATATATATATCCTTCTGCCTGTCACATACATTTGCA
TTTTGCACAAATACAATATATCCTTTTTACCCATTTTCATTGGGTTCAGGCTATAAAAATATGT
TAAGAGTATTTACGGGCGTTCGATAATAATATTTATTATATTAAGCAATGATAATTGTAAATTT
CCAGCATAACTGTTTATTTTTTCTTAAAACAGTTGCGTTTAAATTGACTGATGTCCTTTCCAGC
AATACATCGATCTATAATGCTCACATGTGATGCCATAAAATGTGATGTTTTATTGTTTCTTATA
CAAATTGTTCTTTGCTGACTGCTCAGATAAAAATCAACTGCAAGTAAGAACGTCGCATCTAACC
GACTTCCGGATACTCTGCTTACAATGTAATAAGAGCTCAAGACAGCTCAAATAGAGTCTGTTTA
GTTGTCAATGCAAGACTGTGTTTTTTGAGCTGTGACTATAGAAAGAGTTGAGGCGTGGCACAAA
ATATTATTAATATTATTATATTTATCATTATTATTAGTCAATTACACGTTTAGGATATACTTAT
CTACAAATCTATCCACAATAGCTTTCGTCTATATAAGTTTCAAATATTTAGCTGCAAAATAATT
TATTGGAACAGACGGACATTGCCCAAAACAAATATGCTTATTTCTCTTTGTAACCAGAGTATAA
ATATATGTATAAAATAAAATGATGAAAAAAAAAACACAAGGTAAATACGTTAAAAATTTGTATG
CACCCTCGCACCAACACACACACACACACACACACACACCCACACACACATCCCTAGTCAAAGCTT
ATGCATATCGTAAAAAATTTATAAAATTTTGCCAGCGGGCGGTTTCCAAGCCTGCTGTGACTCC
ACTGTGTGTGTGTGTGTGTGTGTGTGTGTGTGAGTGTGTGTGTGTGTGTTTAAATATAATTGAT
TGACATTATTTAAGCGTAAAGACAAAAAATTTG
>Contig18389:851..1(revcomp)overlap 45nt
TATAATTGATTGACATTATTTAAGCGTAAAGACAAAAAATTTGCTAGTAAATAAATGATATGCA
AATTGTTTTTGCACACACAGCGTGCCTGGCATGTGTTCCCAAAAACAATAACTCCAATTGTTTT
TGCCTAGACGTGGCTTAGTTTCGATATTTGATATACCAAATTCTGACTTTAATTGAAATTCGCA
GCGGCTTTTAACAAATTTTTCATGCAAATGCATTCATGAAATATTTGCCAATTTTGCATTTTAT
ACAAATTTTTATTTTAGGAGCCGGCTCAAATAAATTCTATTTTCATATTACCTTAAAATAAAAG
TATTTTATTTATTTTTTGATGCCCTTAAACAAAGCCAGAACTGCTCAAATAATCGAACCAAATA
GTTTTGAGCTACATAAGGAAGATACTCGCCCCCGCGAAATTGTTGAGAATCGGCCGCTGGGAAT
GGCATACGTTTATGTTGATGTTCATTTCAATTGTTCAAGTGCTTATTGCTGTTGTTATGGTTGT
TTTTTATGGGTAAATTAAATATGAATTGTCCTTTGCCAGCCTACAGGATGCCTATGCTCTCGCT
TGCCTCACAATTAGTTAATTGACTTAAAATTGTTCATTTCGTTGTTGTTGCCGTTGGTGTTGCT
GCTGCTTGTCGAGATGAAAGTAAAGTTCCATTTCTCAGAAGCAACACGCACAAACAGATAAACA
TGCCAAGAGACTTCTCGAGAGAGACAGAGAGAAAGGCAGCAACTAGTGAGAGAGACAGAGACAG
AGAGTGCATGAGAAACAAATGTGGTGCAAAGTCACTTTGAAGTTCCCCGCACCCGCTCCAACCA
CAAGACGTTGTGAGGTATGGCGCAGG
>Contig8501:3179..1(rev_compl) no overlap
GGGGTTCGCCTGCCTGTGGCAATTGCTGCTGCATGCTAATCGAGTCAAAATGTGCCCCAGGAGC
TGCTTCCGTCTACGTAACAAGATGTGCGGTTGTTCTTTTCGTCGTGGCTTTTGTGAATAACTTA
CGAACAAATTAAACTGTTTTTGGTTTTAATCTCACACACACACACACACACACACACTTATGAA
CACACACTGAAATTGCCTGTGTTAATTATTTGTTGTGCTGTAAAAAATGGCGCTGAACTAGTTA
ATTGAGGCTCAAATGTAACAAAATTTTGCTGCTGCTTCGAGGCTAACTCACTTTGGCCCTTGGC
CATTTGTAAAATGTCAAAAATGAAAACTGTTTGTGGCTAAACAAGCACACACATACCAGCACAC
CCATTCACACACACACACACACACACACACACACACACGAAGAAAGACAGCGCTAAGCAGAGTT
ATGAACAAAATTGTATTGCCTGCGACTCGGAGGACAGTCAAAAGTCCGCTCAGGCATGTTCCAA
ATTTGTCTTGGCTTATGAGGGCGACACAAAGCGCATTGTTGGCCGTAAAGGGGGCAAGTGGTAG
AAAGGGAGGTCGGCCATAGCTTGTTGCTCTCGACTGGGGCCCATCAAGGCCAGAGCGCTTTGTC
CGCCAAGATAGTTTGTTCTGCATTTTGGCCTCACGAAATTGCGCACATTCATTTCCCGTTCAGG

AACACGGAGACAGTCAAATGGACAGCGCTTTTCCAGCATGGCCCCTGCCTCGTAAGACCCACTG
GGCCAGGTCTTAAGAGAATAGGTTAATTGCGAATTTATCAGCTGTTAGGAATTTGTTTGGCCTT
GTTTTAAGTCCAATGTATATTCATAGCCAGCCACTAATTCTTTCTAAAATTAAACGCTGCTTGC
ATGTCTTTTCACATATCCATATATATATGTTTATTCTCGAAGCAAATCCTCAAAATGAGAGTGT
AGCTGTTATTTTCACTTTTTTTTTCTTCTAATTTGATCTATTAAGTGCCCATCAGAATTTATAA
GATGGCTCAGAATGTTCTATAATGCGCTTAGTATCTATGTTAAGTATCTCCAGGCTTGGACAGT
AAATTGTAAATTTGGGACAAACAGTATGACTTTCACTTTTACAACTGGTTGGGAGAGTATTTTA
ATTATCAAGTTGTTTTACCCCTTTTTTATAATGTTGAGCGAGTTTTAAGTCAATCGATTAAAAG
GGGAAAATTACAATCAACAGTTTCCGCCCGTTTTATGATAAAGAGCATTGCAAAATGAGTTGAA
ACCCGCCCGGGCGATAATTAAAACTTCGCCCATGTAAGCTTTGTCCCATGTAACCTTTGTCCTT
AGTTTTTCACCCAAGGATGGCGTCCTTTCCGAAAAGTACAAAGAATGGTCTTTCGAATTAAAAA
ATAAAGCTCTGAAAATGAGCTTGAATCGTATCGAATGAAATAGAATGTGCTCCCACAATTGAGA
AAACAAGGTAAACAATTACTAAACAAGTAAGAGGTTCTAGTCGGGAGCTCCCGACTTGGGGATA
CTCTGAACCCTCTTCTTCCAACATCAAATGCATATATATATTCTATTCTAGAAGCTACAAGTCA
AGTTTGGTGACTTTAGCTCTTATTATTTACCAAAACTGCCGAAAAAACAGGATATCGATATCGA
TTTTTATCGATTACTTGGAAACGGAGTAAGTTATCGATTATTGGAAGCAAACTCGATCTGCGCA
GGCACTGGGAGCACCTACATTTCAAGTCTCTAGACGGACAGACGGACAGACAGACAGACGGACA
TGGCTAGATCGACTCCGCTATTGATGCTGATCAAGAATATATATACTTTATGGAGTCGGAGATG
CTTCCTTCTGCCTGTTACATATATTTGGATTTTGCACAAATACAATATACCCTTAAACCCATTT
TTAATGGGTTCAGGGTATAAAAATGAACTGGATCGCAGCATACAAAGGTTCCGAATATATAAAA
TAAAATGAATGTATTTAAGAATCAAAAATTTATAATTTATTGGTGCCAATTGAATTATTAAATG
AACTAGTTCAGTGAGCTCTCTAGCTGCCTCAAACACTGGTTCCTATAGCCACATTTATTAAAGA
TATTCAGAATACGTTTGTGCATTATTTATTTAGGTAAAAGACTGCAAATTTCCTTTGATTTTTT
TTGGGATCTGGATCATGCCAGCGATTGTGAATCAGTTTAGCTTGTAAAAAACTAGTTCAGCTAT
GTATTCTTCAGGCACTTAATTATTGATAGAAGTTTCAATTTTCTAGTTGAAAACTTAGATAAAG
TTGTGCTTGGCATGGTCCATGCCCGTATGATTCTTTTTCCCTGCAGTCGAGCAACAATTGTCAT
AGATTCGCCATGAATTGGGCCCAACTCATCTCGTCTCACACGATTAAGCCTAATTCTAGCTTCA
CATCAACGTGCGACTCGCTTGCTTATTAAAATTTATGATTTCCCTCTCTGCGCCCGTGTGTGGG
TGCCTTTGTTTGGGCTTGGGGCGGAGCGTTGTATGATGTGTTGGAAAAGTTTTACAACTTTTAC
CAAATTGTAAAGTACGCCCCTCAAGTACTAAAACTTTTACGCCTCCAAAAAGTATGCAATGTGC
ATGTTACAGTCGTCAATTGTACTCATGATCTGCATGTGTGCGTGTGCGTGTGCGTGTGCGTGTG
CGTTTGCGTGTGTGCCTGTGTGTGCGTCGAGTCAAATGTCACAAAGGAGCATGCTGCTCAAGCA
ACGGGAGCAGCATAAAAAATTCAACAAAAGAAATGTGAAAATATGTCAGCGGGTTAAGCAGCAG
CTGAAAAACAACGTGCTGGCGCATGCCGTTTGCCCATGGAGCATGGGGCGTGGTCGTGGCATTA
AATTATGAACTCTACAGTGCATGTGCCATGTAGCAGACGAGACGGGAGTCCTGTGGCAAGGCAG
GGCGTACACAGTTTAGGGGCGTGGACAGACACACAGCACACATAGTTGCAATGCGACAAGCAAC
GAAACGTGAATGTCACAAGCTGCCAAATCTCTTCTACAATTCGCAACAATTTCGCCAACTATTC
GCCAACTATATAACTCAGCTTAAACTCAACAAAAGCTGGGTAAATAAATTTCGCGATTCAATGT
TGTCGTTTATGTTCTTAAAGCAAACTTTAGTGTTTGGACTATT
>Contig1707:22782..1(rev_compl) overlap 35nt
ATGTTCTTAAAGCAAACTTTAGTGTTTGGACTATTTCGGTAACACTTTTGCGTGTTGAAGCGCT
GTTGCATGCTCATGTTTAGTCTAGAACATAACTTGTGCTTAAATGCTGAGTAGTTTCATACACC
CGCACATACACGCATAGACGGTTACCCTGAGGGAACAGCCAAAGGCTAATTTGATTTTATTAAT
GAAATCATTAGTAGAAATGACAAAACGTAGCCCGAAGGTAAGGTTGAAGTCAGGACACGGCTGC
AGTCGACCAGACGAAATGAATTTAATGTAGCATGTGAGTGGTCGTAACAGCTATACCCTCTTCA
TACCCTGTACTTTGGTGGGGTATCATACAATTTGTAATCCTATACCATCTTTTATGGAAACAAT
TTAATAATATAAAAATAATATATATAAACAATACTATAAATTCAACTTATGTCAAAATGTAATA
ATAAGTTAGATAGTTAGTGAGGTTTTAAATGTTTTTTTTTTTATACTTGGCTATTCCAAATATA
TATTGATTGTTATTATCATTATGATAATTATTAATAAATGTATAGGATATCAAGCTTACATTTC
AAACATTAAACTGTCTACTGGAGGCTTGAGGTAAGACAAATACCGCATTCAAACAATAGCCCGC
ATCCACGTTGTTCATTTGACGAAAATTCTGGTTCACTTAAACAATTCATTTGCCGTGGAATGCA
TTGTCAATGGGACTATGTTTGTATAAACGTCAATCTTCTAATACAAAGCAACCAGGATTAATTG
CCATTAAATTTCATCGATATTTATCGAAAAAGGGAAGTAATTCCGGGCCTCTTCTTCCTTCTAT
CTATGCAAAGGGTATTTTAGTTTTAAGAAATATCTTCGTCTTTAAGTATATATAAGTATATATA
TATATAAATATATTCTTGATCAGTCAATCAGTTTGCGTTGACAAACTTCACACATTTTTCTCCA
AGGAACATGTATTATGTGTATTCACGATGGAGTTGATTCATGATGTTAAATATCTATACATCAT

ATGACGACCGACAGAGCTTTTGGTCGTTTTGAGTTATTCTATTACAAGCCATATGTGCTTTTCA
TCGAAGTACTATTTATATCAAATAGTTCCTAGAGAAGAAGTCTGTCCCAATAGTTGGCTTCACT
ATGCTCGCAAGCGAACGCGTACGGGTATATAGGAATAAGTCGTGCCTCGGATATTCTTCTGCCA
GGGTATATCAAATTCGAAGCTCCGAAGAAAAGTGTACTTCCATCATTTTTTTCGTTTGTGGCCA
TTGTCATTGCTTTGTCCTGTAGCAGGAGTCCTGGTGCGACTCTTTGTCATGTCAGTGGCTCATT
TCATGCGTACCCTTTGTGGGCAAACTCCCACCCAACAGACAACAGCCACCACCCACGGGGCGGG
GTATGCCGCTGAAAGCTGAATGGTAAATACGTAGTGCGATATAACGGTAACGGATGTGGTCATT
CACACAAATTAAATATTTGCTTTTCGCTATTGTAAGCGGAACGTCATGGTCCGATGTGGGAAAT
TTTATGCATTTCCCAACGCCATGTTAAATTAATTGAGGCAAATTTTCATGGTAGACCAAAAAAC
ATGAGCTTCTGGCACCCGGGTAAAACAGCTGTAAATACAAAATACGAAATACCAATTTACGCAT
TTCATTTGCGCGCTTTGTTCATTTTTTGGATCGTCGCAAGGTTTCGGCGTTCAACAAAGTTGAA
CAACAGAAGCTATAAAACTGGCAACTGTTGAACACTTTAATGGCACTTTAATAGTAGTTGAGTC
TTTTAAACTGATTGCAATGCTGAAAAAACGTAGTCTTCTGCTCAAATCAACTCGATCTAAGTTC
CTTTACAGCTTTCTGATTGCTCTATGGCATTATAAGTACTAGGAGCTACAGGCAAAGTGCTATA
ACAAACTAGTTTAGTACAGTCCAAGTCTTTCCATACCTTCATACTTTAAAAGCAGTGCAAAAGT
ATATTGGAAATAATCAACTGTACTACTAGATATAGTTAATAGAATCAACATATATACAACGGTT
ATTATTGTATGTTTATTCAAAAACTAATAAAAACAAAAACTAATAAAAACAAGTAAGAGGTTCT
AGTCGGGAGCTCCCGACTACGGGATACCCTGAACCCTCTTCTTCCAACATAAAATGCATATATA
TATACATATATTCTGTTTTAGAAGCTATATGTCAAGTTTGGCGACTCTAGCTCTTATTATTTAC
CAAAATTGCCCAAAAAACAGGATATCGATATCGATTTTTATCGATTGATTGGAAACGGAGTAAG
TTATCGACTATCGGAAACAAACTCGATCTGCGCAGGCACTAGGAGGACCTTCATCTAAAATTTC
AGTCTCTAGCTCTTATAGGTTTTGATATCCTTGCGTTCATACATACGGACAGACGGACAGACGA
ACGGACGGACATGGCTAGATCGACTCGGCTATTGGTGCTGATCAAGAATATATACTTTATGGGG
TCGGAGATGCTTCCTTCTGGCTGTTACGTACATTTGCTTTTTGCACAAATACAATGCACCCTTA
TACCCATTTTTAATGGGTTCAGGGTATAAAAACTAAAAAAACACTTGTATAGTTTGATAGTTAG
GACTATTTAAAAAAAGAGGAATATATGTACATTTATTTATTATAAATAATTGAAAACTTGTTAG
TTAGTTCTATGAATAAGAAAAATGAACAAGCAAATTTGATTTATAATAAATTTAATTGTTCTAC
TAAAACTTGTACAATGTTTGTTTCACGAACCGAACCTTAAAGCTTTTACTCTGTAAGAAACGCT
TCGTTATTACTTACTTGTTAAAATTGATGAAATTGAGGCGAGCAACGACGGGTTCTTTACTGTA
GAAGCATGCATGAAGTAAAAGAGATTCCTTGGAACTTTTTAAAAATATTACCATAGTATCATAA
CATCATGTCTCTGAACCTTTCTCTGCTCATATTTGTATATAGATTTATTATATGAAACTCTTTT
TTTTATACACTTTCCACATTTGTTACGCTTTCACAGATGATTTGATTTGATAGAACAAATATGC
AATGTAATTTTTAATGTTCGACTTGCAATAGTTCCCTCCTCGTGCATCATCAAGGCTTTTGCAC
CTTCGATTTGACTTTGGCTTAAGCCAAGTTCCTTTGAATAGCATGCCTAACAATGAGCCACTTG
CATTCGCCTGGATCTATTCAAATGGATTTCTTAGAAGTGCCGCCAGACTAGAGACTATTTCCTT
TTTCGCATTATTATTTGCATCTTGGTATGCTGGCCATTGTCAGATGGACAGCCTTAATAATCAT
CTCACACACAGCAACAATCAAAGTCCCGCCCATTGAGATGCTAATGTGGCCATGTGCGTGCCTC
TAGGCCCAACACTTGCCGTTGTGTCTACCCGACACCTACTTACATATGCATATACATATCATGG
CTACTCAGCTTGCCAATTTGTATATCCTTGTAGAGGGTGTTATAATGTCACAAAGTGTGTAACG
CACAGAAGGAGCAGTATCCGACCCCATACACTATATGTACTATAATACATATTCTTGATTATGT
AGCCGCATCCGTTTGTGTGCTTCTATGTTAACTTGGTTTTTTATAGATCCGTCTTTCTGTAACA
GGATGTGCCGGGTCGGAACACGATACCATTTAGGTGTCATATAAATGATTGCCCATGAAATAAG
TTGTCCTTCGACAGCCTTAACATTATTAAAGATGTCTTACCCAGAATTGGCATTCTCGAGCTTC
ATTGTAATCTCTACATAGGAGTGAAATCTTATCAGAATCGATTATATCATTGAAAAATTTGTTT
TTAATGGTTCGATTTTCTGGTTTTTAAGTTCAGCAAATTCGAGCTGCACTTTATTATTTAACAT
ATTCGCCAGGGCGTATCCAACCGAATAAGATTGCTCCAACGCCTATTTATAAAAGCTTTGCTAA
GTCGTAGTCGTAGTAGTGTATATATCCAAACAGGCAGAAGGAAGCATCTTCGACCCCATAAAGT
ATATATATTCTTGATCAGCATCAATAGCGGAGTCGATCTAGCCATGTCCGTCTGTCTGTCCGTC
TGTCCGTCCGTATGTATGAACGCAAGGATCTTAGAACCTATAAGAGCTAGAGAAAATTTTAGAT
GTAGATGCCCCTAGTTCCCGCGCAGATCGAGCTTGCTTCCGATAATTGATAACATACTCAGTTT
ACAAGCAATTGATAAAAATCGATATCGATAATAAGAGCTAGAGTCACCAAACTTGACATAAAGC
TTCTAAAATAGAAATATATGCATTTGATGTTGAAAGAAGAGGGTTCAGGGTATGCCCTAGTCGG
GAGCTCCCGACTAGAACCTCTTACTGGTTTAATAATGTACCTACAATTGTTTTCCTTTTGTTAG
TCTATGACATTTTGCAGTGAGGCATTTCTTAAAGTCTCAGTTATTCTTTAAGATGTTTCAAGAA
AAATGGATTATAGAGCATGTTTGTGTAAATGGGTTTGCCAATGACACTGGCTCCTGGCTCAGAC
TGTGACTTAGCACAACGGTAGCATGTGTACACACACACACACACACACACAGATAGACACACGC

ACACACCATAGGTAAGCATGCATATCTATGTGGACGTGCCCCATTGAAGCGCACCTAAGCACAA
CAAACAAATCAAATGTGGCAAGGATGCGTTGCGTATACGCACCGAGGCGCTAAACGTGCTGTCT
AAATGTTTGAGTTGGCTAGATAAAGACTAGAACGTACCGGCAGCATCAGCGGCAACGGTAGCGA
TAAACGGATCGTCCTCGTATCGGGTCTGCATTCCGCCCAGACACGCCACGCTTGGAGTACGAGT
GCCTTCGACACGGGCACATCGCTTTCGCTAAGACAATAACCGATGCTAATGTATTATACGTGTC
AATTTTGAATCCCGTTTGACTGATATGTGTGCTCACATCACTGGGCCACACTGCCTGGCTGCGC
GTGTGCCCATGTGGGCGTCATAATACATTGCCAGCCTGTGCCACGCCCCGACGCCCAGAAGCCC
ACTGTTATGCCCACAGAGTCTGCCTCTCTAGTCTAGGCAGTGGGTAGTCGTGGTCTCTTGATAG
AAATGTCGCTGTCAATTTCTGACAAATAACTTTATCATACAATGCAATTGATAAGCCAACAAGT
TGTGCGGCCAGCCGAGACCAGAGGCAGGGGAAGTGGAAGTGCTAGGGGAAGGCTGTCGATTGAT
AACCGATAACACAGCATTGTGGAAATAGCTATCGATCATAAAAGGCTAGACAGCAGGAATAAAT
GCTAAATAGATTTTTGCGCAAAAGTTTTTAACGAAAATTGTAAAGGCAAAGGCAATGTGGTTAA
ACATTAAGTCGCACAAATCTGTGCTATATATATATATATTCGTGATCAGCATTGCTCAACCGAG
TCGATCTAGGGATGTGCGTCTGTCTGTATAATTTCTATACCTACCAATGAAGACACTTTATAAA
CATAAACGGATTATGTGGGTGAAGCTTGCTTCAAATGATCTTTAACTTTCGCGTTTTCACAAAT
CGATTATTAATTTAGTGGATAATATATAAATACATAAACTTTTGTATTTTTGTAAAATCGATTA
CTATTGATTAATATTTGCTCATGGTTTTTTCATCTGTAAGGTAATAAGCATACGATCATTTTGT
ATTCTACATACCGAGCATGCATTTCTCACTTTTTAGATACCTCTTCCCATTCCCCGCAAAATTT
AAAAGCAGCTTATTTTAACCGGTCAAATGCTGAATATTTTCCATAGACCGGACTACATATATAC
ATCGAGGGCATTCAAGAATGGAGTTCGCATAGCGTGTGCCTGTAGAAGCACGCAGGTTCGAAGG
CATTGCAGGGCAGCTCAAAGTTGTTCTTAGGGTATTTCGAGCACTTTCCACAATGATATCTAAT
ATGCTTTAAATTCAAATGGCATTGTTGGAACGCATGTACTATTATCTAAATTACAGATCAAATA
GGCTGCTGCGGTATTTGGTCGAGCTGTCGAGCGGTTGGCTACGCGATGGCCTCAAATTCTGGCA
GAGCTGTGGTTTTTCTATTCAATACGAACAGAACACAGATCTGTGGCTACGGATCTATATGTAT
CTAGAACAATCGTCGGCAGAGCAGACTGTGGGCCAGAAATATCGATTTTTGGCCTCTCTTCAAC
CAAATCAATCAGAAAAAATATGCTTAAAATTGCACTTTACTTTTTCAATAGAAGATCCCAACAC
AAGCTAAAATATGTAGCGATTGTTTCATATTCCAACAAAAAAAAACCTTGAATGAATTTCTTTT
AAACTTGAAGAAGATAAATTTGAGCACTAATTTGAAAGTGAAAAAAAAGTCAAGAAATTTTTCA
GTTATTAGCATAAACATTTACTAATTCTTAGTGCACAGCATGTAAAGTTTTTTAGTCTTAGAC
TTTTTTGCAGCGTAGTTTCTAGTCGAGCACTTTTGACGCTTGCTCGTTGGTTTATTTGCTTCGT
GAGCATGTGTGCGAGCTTTGATGTGTTTTATGCATCAAATGAGTTAGCGTCGAGCCTAAATAAG
AGCTAAACACTCGTGCAAGTACACACACACACACACCCGCCCACAGATACATTTGCAGATACAG
TGGGCGGCAGCTACGTGAGCGGGCCACATTTATGGGCCTTCAAAGGCAAATGTATGCATGTTGA
TGTATGTGCATGCTGGCTATTACTGTGTGTGTGTGTGTGTGTGTTTTTTAAGTAAGTCATTTGC
GTAAACTGTGAACATTTAAATTGAATCAAGTTCTGGGCATGCCGAAAGGCTTCCGGACTAACAC
TGAGAGTCGGATGCGTTGGTAATGAAACCCTTGTGGATTGTCAGCGTTTGGCCAACTTAAGTGA
CATTCTTTTCTTCTCTTTTATTTTTCTGGGCAACTTTTATGATGTTGGGGCAAAGTCTTGCGAC
AATAATTTAACTTGTCTGCTTTTTTATGCGACTTTCATACGCTGCTTGCCAACTGGGATAAAAC
GCAAAAAAGTTTAAACAGTTTACAGTTTGCAGCTGCCCTCAAGCGTCTCAGTCGCCGTCGCAGT
CGCCGTCGCAGTCGCCGTCGCAGTCGCCGTCGCCATCGATACGCTTTCAGCGCCACGTTTCCAT
TCACAAATTCAAGCTTATGGGGCTGGGCAGGGGATGAAAGCCACAAGGCGACAGCTGGGTTACA
AAACGTCTGGAACTGGTTCTGTTTGCCTGTGCTACAAGCGAAAGTTTAACGCTTGGCAGCGCAG
CACATCAAGCATACGCACTGTTGCATGCCCCATGTCTGATGGCTGTCGACTGTTGCTGCGGCTG
TTGCTGCTGCTGCTGCTGCTGCTACTGCGGCTTCGGCTGTTCTTGTGGGAAGTACTGTGACTGG
GCAGCTAACCCTTCAGATGCGGGCTTAGCAAAAATTAAACGAAGCAATAAATTATGCGTCTGCC
AATGGGCGCCAGTCAGGCAACAACAAAAAAAAAAAAAAAACGAAAGACAAATTTTTATATATATA
CGACTTTCTATATATAAAAAATGGTGCCTTTGTGTGCGTGTGTGCGTGTGTGTGTGTGTGTG
TGCTTAAAAATCCCATTTTGTATGCAGATTTGCCTAATCTTAGGGTTTTACTCTGTGTATTTGT
GTGTGTGTGTGTGTGTGCAACTGGCAGCAGTTTAGCGACTATTATAAACCGGAAATTTTACCCA
ACACAGCTTCGTATTCCGGTCTTTGGATCTTTTTGGGGCGCCTCAGCATATGACAGTGACAGGG
CCGAAATGCAATCTTAAATTCATGAATGCTTCATTTGTGATTTTTTAAGATTTCTTGCCAATTC
GCTGGCTTAGATAAAAATGCGTGGTCGTGCTTGCCACCTGTTGGGCTAACAGATTTATCAACTG
CGTGGCAATAAACTATATTATTTGATTATAATATTGTGTTGTAGGCATTATACCCAGTTTTGCA
CCTCTAACAACATTTCTTGCATCTTACATTTTTTGTTAACTTGCATGTGCAAAATCGAACACTT
TTAAATTAGCTGGGCGTGTTTCGAGTCTGTGACCATAAGTAATAGACCCTCCAGTCGTTATAAC
GACAGCCTCTAGATTCTGGCAACCTGTGGGCTTTTGAGAGGCGAACCCATAGTTCGGATTCCAT

TCATCTAGACGAAAACTAAACACAACAAAATACAAATAATATATATATATATAATTGAGCTGCACG
GGCGCATAGCCAGAGCGACTCGGCAACCCATTTGAAACATTGCTATACTAACGACCAAATCATA
ATAATTTATAGTTAATCTAATAAAAAATAGGGTGTTCTTTGGTGAATCAAGTCGAACTATAAGC
CATGAATGGGTTAAAGAAAGTTTTACCAAAATGTTTTTCAATTACTGTGGCAAACTCTGGCTAA
GCCTCAACTCAAACTGTGCCCTTCCCAATTCCCTTCAGTGACTCAATTGCAGAAAACTTATCTA
ATCGCCAGAGATTACATGGAAAAAAGGCTGCAAAATGTCGTGCGAAAATCTTTTAACAGATTTC
CATAAATTCATATATACAGACACATTTTGTAAAAATATATGTTAATATATGCGTGCAGTCGTGT
CTGTATCTAAAAGGACTGTGTCATTAGCTGGGTCTAATAGGAACGCCCTCAGCCTCAAGGGCTC
AGGAGGTGGGTTTGGGAACATAGAGTGTAAGGGGGGGGGTTCGGAAAAGCTTGTATATTTTGCG
AGCTTGTCTATCTGTTGTGGGCTAATGTCTTCATTAAGCACAGCGCTGCCACGCCCCCAAGCGT
ACGCCTACCGTAAACTGTATTTAATGCGCATATTTATTTTATTTAAATATTTATGTATTTTTTG
CTCCTCCCTTGGATTCTGTTTTTGAGTTACATTTTATTTGGTTGTGAATTAACTTAATTAATGC
GTCGCATAAGCTGCCGCTTAGGCATTAGCTGACATTCTCGTCCTTGTTGTCCTTCATCAAGTCA
ATCGCAACAGCGGCAAGAACAGCAACAACAACAACAACAACAACAACAACAACAGCTTGTTATA
ACATTTCAAATGTTGCTTTGGCAGGTTTTTGTTTTGTCGCATTTTTAATTAATTTTGCAGAGGA
TTTTTGCGGCTTATGAACTTTAAATATTTCGAGCCCCCCACGCCCCCTACTTGCCCAAAGCAAA
CATAAATTGACAATTCAGAGACATATATACATACTGTACATGTCTCTAAATATATATTTACATA
CATGGTTTATCTATCTATACAGGAACAACACGTATGGGACAAAAAAAAAAAAAAGACATCGACC
TAAATTAAGCGACACGCCTACATTAGGTATGCCTGTGTGTGTATGCTTGATGTTTATTTATGTA
GTTGGTGTTGTGTTTGACATGTCACACACAAAAACACACACACACACACACTAACAGCAACAAC
CACTGCCGACGCAGGGCACGTAACTCATACGCCATGCAGTACGCGCGCCCATTGGCTAACAATC
TTTGGTAGGAATAGTAGTAATCGTATACAGCTTAACTTATGCTGATTCTTATCCGATATATCAC
GGACATATCTTGGTATGAAACGGAATGAATTGCTGGCCAATCGATTGGCAACAGCCTTTAAATT
GATCAAGCTAAATACTCGTATATATTCATATATTTTTTTAACTCTACGAACTCAGGTTTTGAAT
CGACAATTTAGTTCGTCACGTATTTTGCAAACTTTCAATGGAACAATATAATTTTATTTAAATT
CATTATAGTAAATGCAAATTTATTTAATTGTTGAAATTAGTTCAATATGCACAATACTAGAAAC
ACAGTTAAGGTTCGGCTATATTTTACAGAAAGTGCAATTTCAGATTTAAGCCGGTTCGATATTT
TGATAGATATAACAACGATTTTTTGACAAATTTAATATCTAACAAAGTTTTAAAATCACATTTT
CGAAGCACCTTCGTAGGTAAACAGTTTTGGAGTCAATTAGTGTTGATCGATATTAGATGTATCA
AAAAAAGGGTAAACAGCTTTTACAACAAATATATGAGTTGTTCGAAAAGAACGAACGGATCTAC
CTTATGAGTTCAGATTTCTGATACAGGGTATAAAAAACTTGTCGAGCTATTGAACTATCGACAG
ACTCCTTGGTTGTTTGGTTTTATTGCTCGCGTAATAATATGTTTGTGAAAATAGGGAAAAGCTC
GCCCACGTTCAGGTTTGCAGCACAAACAAAAGGCAATTAAATGCAAACTGGTGTTTTATTGCCC
ATAAGAATATATGTAAATAAATATGTATAATTTATAATATATTTTGAGATTTAATGTATTTTTA
ATATGACCTCTTCAGTATTTGCTAAGGTCAAGCGTATCAATTACGATACGAACTATACGAATGA
ACGAGAGCCAAGCAGCGGCTTACTTTTGGTGAAAGTCCAGTGAGTGGGCGACGGTACACGGTGT
CTACTTGGAGGTGAGGTGCCTGGACAAAGGTGTAGGGCAAACCAAACCAAACGCCAACGCATAT
AAAAGATTTATATTAAAGGCCCGGCCAGGCGCCCGGAGGCGATGCCCACGCAGCAATGACTAGC
ATGGCATCCTGTTCGAATGCCTAAGCTACCCCTCCCGCTGATTCTATCTCTTCTGGCAGTGTAC
GCGTATTTTGTAATTTATGATGGCAAACTCTCTGTGTGAGTACATCATACTGGCATACACACAA
GCACACACACACAGATGTTTACACATGTATGCTGAGTTGATTTAGAAAGCAACAACAAACAGGT
CTAATGCAACAACACGTGACAGGCGTGAGTGCTTCAATAAAAATGCATAAATTATTGTTTATGC
GTATTGAATTAAGCGACGCAAACACTAACACGCCCCAAAAATTACGCATGCCACGCCCTCGCCC
CCCTCACACACACACGCAGATGATAAACTTTGCCGAGATTTGAAATGTATGCAAAAAATGTTTC
GCCGCTGTCAATAAAATAGATTTTTTCTATCCACGCCCACAAAATGCCAGACGCAGGCATTATA
AACAATGAGGCTGTGGCATTCGACAATATTTGTTATTTATTGAGATTTGTTTACATTATATTGT
ATGCGGAATTTGAAATGCCAGCCCCAGAGTGCCACGCCCAAGTGCCCATTCCACCTTCACGGCC
AGACTATGCTCCGGTATCCTGTGCCGGCCACCGGAAGCCGTTTTCCTCTGCCCCCAACATTGTT
GCTCCACATATTTCCCACTCTTCCAATTCCTCCTTCAGCAGAATGGAGGCATTTTAATATATGT
TCACTTCACAGCCTGTGTCTCAGTGTGTGGGCGTGGCATGTCAACAGGGAAATTCGAAACTGGA
AAAGCTAAGCGAGTTGTTGGACATGGACGCGCTATGTTTCCCGTCTATACTTGAGCAGATATCG
GATGGACATTAACCATTTCAAGGTTGAAGTAAAATATATAGTAATAATAAGAATAATTAAAATT
AGAATAGTAGTTATATGCATATATACCTTTAGAAATAATAATAATAATAGGGGCCATGCCCTGA
TGCCTTTATTTTAATTGGGTACAACTTAGTTTGTGCTTAATTGTTATTTCCTGCACATACCAAA
TGAATGTTATCTTAAATAGAAATCTTTAGTATATGCTGCGATGAAAATGTGAAGCTTTTATTTC
TGCTTAACATAATTGTTTTCCGTATTGTACTAAGAGGAACTATTTTGGGTCAGAATTCTCAATT

AAATATATACTGAATGTGAATGAAGTAGTCGGGTTTTTGAATGTTAAACTTCTTGCCGAATATC
TTACACACGTACATCGTTAATTGTATGTAGATTGAGTTGAATATTAATAAGCACTATCAAATAT
AGGGAAATCTATTCTTCTTCCTATAATTCCATGACACGAAACGCACGCTCACTGTGCCAGTCAA
ACAATTTGAATGATATTTGATCTTATACAGAATTTTGAATACCAAGCATAAATCAGCAGAATTC
ATGAGCCATAAAATCTTAATATTCACTTAAATTGCTTCAGTTGCCATGGAAATGCGGTCTAAAT
AACCCAAATGTGTACATGTAGGTATATATATATATATATATCGGGTTGAGTTGAGGAAAGCCTC
TGTGGACTCACTTGAGGGATGGTCAAGTTAACAACTTGTGATTGAGCTGGGGTGAATCTTTCCT
ATAAATCTGAGGCGGGGAGCAACTTAATCAAAGCCGCAACTGCCATAAAATTAGAAAGCATGCG
ATGAGGTCGACCAAGCGATGACAATGGGGGTCACGGTCAGCCTTAGACTTGAGGCTAGGGGCAG
GACGAGCCGGCACGTAAATCCTTCAGGCTTGCAGGCGTCGAACAGTTGATTAATTCGTCGATTT
TATTTGGCGTTTTTATGAGCAGCTGCAGCCGTGAGTCGTGTCACTCCTCGTATGTCAGTTCGGA
GTCAGGGCTGTTGCCAAATTTTGTTTGCCTGACATCGGCTGCGGGTTACGCGAAACTTGGCCAA
AAATAACTGCGCCTATGGAGCCACGTGGCTATCAAATTTCCATTTCCAAGGCCAAGCAGCACAA
GTAACTCGCATGGCCCCAAAACGAAACCACATCGAAAACCGAACCCAACCGAATCAGAAGCCGA
AACCGCTCCGAAACCAAACATTGTTGAACCAAGCCTGAGCTGGTGCGACAAGGCGTCAAACCGG
CCCAACATGGAGAATAGGCGATACTTTATTTTACCATTAATCGTAAAAATTACTCATTTTATAG
CTTTCGAAGTGTTTTCTCGGTGCCAGTCGCTGAGAAAGTTTGCATGTGCCACCGCATGGCATAA
GGACTCAGGCTCTGTGAGTGTCATCCAGTTGCGAAGCGCTGCGGCGTGAAGACAAGCGATGCGT
GAATCAGTCGAGAAAAAAAAAAAGACCAACAAAGCCTGACACCAAATGCCACACAGCGACCAGGT
GGCCTCCGGCAATGCCTCAAATATCGATGTTAAGCGTTATGCATAAATCAGCGTATTGTCTCAG
ATATGTCTTCTTTTCGTCTTGTGGGATGTGCCGGCAGAGAGGACATGTGTAACTGCTCAAACGC
ATGTGGGTGCGGTGTGTGTCTATGTGTGTGTATACATACAGGTGTGCAAGGTATTCGATTCATA
TTGAGGAAGCTAAGGATTTATATCCCAGGAATCGTTCTAATCAAAAGGCAATCTTTTATATTCG
TTGATCCGAAAATAATTAATTTAGCTACTGCTGCAGCATATCGATTATGAGCTGCCTGTGCCTC
CTAAGTACTTAATTAATTGACATACTTAAGAGTTTAGATGAGTAATAATTCAGTGTAGGCAATA
TTTTTAAATGGATTTTTCCTTACACCGCATTACTTGGGTAAAGAAAGGTTGAACCCAAACCGAA
ATAAATGTTTTAGAATAATGTATATATTTAGAGCAAGGGAAACAATTTGTTCTCTGAATCTGAT
AACTTATTTTTTTGAATCAACTAAAAAGGTTTTTATTGAAAAATGGCAAAAAAGTGTCAAGCCA
AGGCTCACGCCAAGGCATGGTTTTATAACAGTGGAAAACTAGGCTTGCGAACCGAAACCTTGGC
GAAGCATTGATTCGAACCACCAAGAATTTGTATTGGGTTTGCAAACGTAATTTTCATTAAAGCA
GATTGTGGCTAACAGCTGCTGAAATGTTTAAAAACAGATCAAATGCTAGCCTCAAGTACGCTTG
TCTATTAAACGTTGTATTCAATATGGAAGGCTTGCCCCGATCAAAAGGCTTGGCTAACATACTT
ATTGGTTATTTATAATTGAATCAATATGCGGGCATTGTCTTTAAATTGAAGACAAATATTTAAA
AATTGGGCACCTTTACCTAGGAGATATAACATGTGCCTCTTATGGGCGCAGTGCTAAAAAGCAC
CAACAGCTGCATAATTTTGTGCATATATTTTTGTTATTTTGTGTTTCTTTGATTTTATTGAAGG
GGCACTTAAAAAAGAAACAACTTCGAAAATAAAAAGGCGACTCAACCTAAACAAATTTCACGTC
GTATCCTTCCATGGAACTGAGCATCCAGTCGACGTTCATCCGCCTTCAATTTGCTCGCTTCTAC
ACGCGCACCCATGCCCAGCCGCTGTTATTACTCATTTACAGACATTTACTTAAAGCAGGGTCTG
GGTAGAGCAGAAAAGACTGGGAAAGGTTGGGGGGGCTGGGGGCACCTTCTGTTATTTGCATTGG
ACAACAAAATGAGCAGCGCCGAAAAAGTTTCAAATCTTTTGAATGAATTTAATATTCACAAAGT
ATGAAAAATACTCGCAAGCAAACTTTGCACAGAGCAGCGAGAGTTAGGCTGGGGTGGGACATCA
TCCTTCAAAGTTCAAGCTCCTTACTCCATACCCAAAATTACCATTCACATCATCATCAACATTA
TCCTTACCCTGGTTAGTATCATCAGCATCGTCAGTATCGTGTGCTCGTATATGATTTGTCTATT
TTGTGTTTGAATATGTTTCATGAAAGACTTTATCACCCTCCGTTCCGACCAGCCAAGCAGCCAC
TTGCTCGCAAAAGGTTTAGCATGGCTGGCCATGAATGCCACCTGGCTGATGGCTGGTGGCTGGT
GGCGGGTGACAGGTGGCAGGTGGCAAGTACTGGTGGCAGGTAAAAGTGACAAGTATATCGACAT
CCGGACAGTTGCAATTTGTGCTACGTTCTACCGAAATGCTTGGCTGACTTTTCTTACATAACGT
TTTAATTGCCCCTGCAGTAGAAAAAATAAACCAAAAGAGAAGAATTCAATTGTTTGCTATTAGT
CATCATGAGGACCTCAAGCCAGAGCGAAAAAGTTTAGATGAATTCTGAATGCAGTCGAGTGTGG
AGCAGATTATGAGAAAATTAACGACTTTTTAATTGTTTGTTAGGGTTTGTTTTGCGTTTTATTT
TGTTTTTTTTTTTTTTTGTCTTTCATTTTTTCTTGATGCTAATTGGATTGCCAATTAATAAGTT
GCATTCGCCTTATGTCGAGTTTGAGCATAAATTGTCAATTCACAAATATAACGTACAATACTTT
GTTTTTAATTTAATTGAAAAGTCCTAAGCCATATTAAGCCAGTTGGATCTTGTTCCTATGTAAA
TCAAACGCCTAAAAGGCGCTGCTCATCAAAAAGGAGCCGCCAGACAATCTGCCTCGGCATTCCA
AAGTGGGTCGGGGTCCAGGTCCCCATTCTCTAATGGGCTCCTTCAAAAGATACACACCCGCGAA
CTATCCTCAAAGCGAATCTTTTTACGTTCTCTTATTTTATTTAAATATCTTATCAGTGTTAACA

CCCTGTCATTGAAACATATGTACCCTCTAAGCTACACAACCTAGAGTCATTCGCTTGGTAGAGA
TCAGAATGTTCTGATCCATTCCAGCGTACATTTAAAGTGAAGCTATCATATTTTTTGGATTATT
AAATTGAAACAACGCGATTTCATTTGCTCGATTTCTCATTGCAAGTCTAACGTTCGCTTCATGT
AATTTCCTTATGTAATTGTCACTGAGTTCGCTTTGTAAGATTTACATAACACTTCATATGTTGA
TGTCTAATGGATTTCTTGACTTGCAAAGAAAAAATTCACAGATGAAAATATGACAGCCTGCTGC
ACCTTGTTAAATAAGAATTTTCTATGCAAGCATTAAAATGGATTTTAATAAACTAAAAACGCAT
TTTATCAATAAATTTCCAACGTATATCCATCCCTTTGTGTAATTGTATACACTAAATTTCGTTG
CAATATAGATAAACAAAAGGAATTATTTAATTTGTAAACTATTGACAACGGTTTTAAAGGCAAT
AATACATAACAGACTAGCTTGATTAAGCATTCTCTCCTCTGTTTAATTGCAATTGTAAATGTGC
TAACATTTACTCTGTAAATATACATATGTTAACTTCCTAACAGACGGCTAACATTATGTTCCAG
GGCTCTGAATGCTTACGAGGGAAAGTCAAATGCTAATGAAATAATTATAACCTGAAATAGTAAA
GCAACAATTTGAAATTGCACTTGGTTTTAGTTAAAATTATTTGCTTTTGATCATGAAGATCTGC
AAATATATAACCTAATTCGTTAATAGACGGTTAACAACATGTTTAAGTGGTTAAATCAAACAAC
TTAACCTTTTAACAGACGAATAACATTAGTTTCAAGGTTAATTTCGTTTGTTCAGCTCAAATAG
CAGCCAAAACCATAAGCCTAAGCTCAATCTAAAGAGCTATTTGCAAAATGGTGGCCAGTACCGT
TGACAGTCCAATTGACCAGGATGCGCAGTCCGGGCACAGTCCGGACAGTGAAAATCACTTTGAA
GCCAACTCATAGGGAATTGGCACGCACAGGATCAAATAGTGTGGTAGAAGAGCGTTGAGCGATG
AGCATTGAGTAATGGCTTTAATGATGGCCCAAGCACATCAACGACTGTTTAACGGGACTTTAGC
CTTTGAACCGTCACACACACATACGCACACACACACACACACACACAGTATACGAGCAGCGGGG
GCATTAAAATAGCATTAAAGCATATCTGGATGCGAAACGCATCCGCTCGATCTTATTGCGAAAC
ATATATGTCCATGCATATATATACATTTTTATATACATTTATATATGTATATAAATGTGTGTGT
GTGTGTGTGTTGGTAAATGCGAAAATGTGAAATGGCAAATTCAATGAAATATTTATTAACCTTT
AATCAGCGAACACAGGCAGACAGCAGACAGCAGACAGCAGACAGTTGGCCAATAGACAGAGCAT
AGCGTCGCTCTAAGCATATTTTATTACACATTTCATGGGACAACGCTGCGTATGTGTGATGCGA
TACGTTGTGATGTGATATATTTGCTTGATTTATTTCGGCTACTGCAATAATCAAAATTATTTGG
TACCAGGTCAGTGTCTGAATGTTAATTGCCATGCCAGACTAGGCCAGCTTAAGTGGAATGAGAT
GATGGTCGGATATTGCTAGGGTATATTTTGGGTATAATGTAACTTCAGGCAGGAAGCATGCAAA
ATTGTGAGAGAAATTGCATTTTATGGCTTTGCATATGTGAGCGACCCTTAATTCGATTTGTTCA
GCCAGCATGAAAATCTGATTAAATACACAATTTGATCAATTTTGATTGTTTTTGTGACCAAACC
AAATGAATTGTAATTAAGTTATGCACAGCATAAAAATAACTAAAACGATTGCTTACCCAATTTA
ATATAAAGCGTTTCCATATATCCTTTTATGAAATTTAATCTAGCAGCGCTTTCCTTCAGTTCAT
TGTATGCTGGCCATTTTGCGCCTTAAGTCAAGTACTGGACCTAGTTCTTGAAGCGCACCGCCAT
TTGGCCGAGTTTCAGCTTGATTCAGTGTTGGCTTGACACTGTGTGTGAATGTCTCATGTTTCAG
CTGCGGAGCAATGTCCAGCACAATAGTTGTCAGCTGCACAATGCTAGAGCAATGGCCTGTGCTT
CCACGCTGGCCAGACAATAAGGCCTCAAATCTAGTCTGCAATACATACTATAGGGATTTAAATA
AAGTACTTGAATATGCCGCCCGTTTTCGTTTGCAGCGGTACTCTAGGGAATAGGTCTGCAAGTC
GAACCGGTTTGAACAAAGCCGAGCCTTAAACCATTTTATTTTTATTACTAAAATAAACGACAAC
TGGCTAAATAACGTTTTGACAAGGCCAAGGCCAAACAAATGATCAGAAGGAATAAATCAATTAT
TTGGCAAGCCGTTTTTGTGCTAGGAGCTATTTCTCCTAGTCTGTCACGCATTACTTCAATTCAC
TTTTACGCATTGATTTCAGACAACTCAGTTGCCTAAGTTTTCACTAATTAGTGTTTGGCTTTC
AGCATGACATTAGATACCTTAAAAAAATTAAAGTTACATTTGGTATAACAAGAATTTGCTCCTA
ACACAATAGAAAGATTATCTTACGTTTACGTTCCGATGATTCTATCAGCTTATCCCTAAATCAG
CTTAGCCCAATTATCGGCATAATTTGCCAGATTTTGGAGCCGCACTAAAAAACAGATTTCGAAA
AGTATTGAGAATTTGTGAAAGCATTCAAGGAATTAATCGGAACGTTTGCCTATTGACTACAACG
ACTGTCTCTCTGAGTGTCTCCCTCCCTCCCTCTCTCTCTCTCTCTCTCTCTCTCTCTGTCTG
GACTTAGTGTTTTCTTTTGCGAGCAAAGAGGATTCAGCAGAGGATGGGTGTGGGGTGTCTCTGT
TAGTTCATCTTATGCTGCTCTTTGAATTTTTCGTGTTTTCCCAAGTAGCTTTCATTTGGGGGAC
TTGAACTTGGCCAAGTCAAAATGCGTGGCGTGCCAAAGCTTCAGCTACAGCTGGATGTTCGTGT
TGTTGTTGTTATTGTTGTTGTCAGCCTAATGTGCTTGTATTTACTTAAACGCACAAAACGGCAC
AATTCATTGGCATTGGGGGCCAAAGGCATTGTCCTGGTCCTGGTCCTGGTCCTGGTCCTGGTCC
CAGCTCTGTGCACCACATTAGCGCCCACTTTACTTTCGCTCAACCACTGATGAGCAGCAAATTG
AATTTTATTGACTTGATTTGCGCCCCGTTGGCCGCATTTCATTTTGTTTGTTGTTGCTTTATTT
ATTTGCCTGACATTTCTGACCATTTGGCTGGCCTTAGACGTCCAAGCATTCTAGCCAGCTCCTG
TATACACAATAAATTGATTTCTGATTATCATTGCATAATAAAATGACTAAATCCGCTCGCGCGT
CCGCTGCCCAACAGACGCACTGAAGGCCAGGCAACCCCAACAGAAGTTTTAATGAAGCGTATTA
AAATTTAATTAAATAACAATTTGCACAAATGTAAATAAACACGACGCTGCTTATGCAAAGCGTG

AAAGAGCAGGACTACTAGAACACTCCAGGAGCACGCCAAAGGCTGGAGGCTGGAGGCTGGACGC
TGGACGCTGGCGATATAAGACGTGGGCGTACCACGGCTGCCATGCCAACGCTCAGTGCGGCGAA
CTTTGGTCATTAAATTAAGTGCACACTACGAGCACGGTAACGACGAGAACGCGGACCACGATGT
GACGAACAAAAGACGAAGATGACGCCGGCACCGACATCGACGAACGATTGTTGTGGTCCCTGTG
GCTACGGGCTGCCCTCATAGACTACAAGCGGGTGTCTATCAAATTGAAATCACGAACGCTGACA
TAAAGTCGCGTTTTATATATCCACGTCCCGGCCTGGCATTTGAAAAACATCATATTTTTAAGCT
TTTTTAATACGTTTTTATGCTCTGCAACAATTTAAATAATAGGCAGAAGAAGGCATCTTCAAGC
CTATAAAAAAATATGCATTGAATATATAATAATTTAAAACTGGCAAAAATGTACAATTTTAGAT
TTAAAAAAATTGTTTTCGGTTTAAAATACGTATATTTTAACATGAAATACTGTGCAAAGAAAAG
TTGGCGGTTTTTGGTAATGTGTGAAAAACGTCTCAAATGCATTTGTTGTGTTATATCACAGTTT
TTTTTTGCGAGTTTTTCTACCAAGCGGATACATTTTGAAAATTCGCAGAAATATAAAGCAAGTC
TTCGACCTGTTACTCAATTTTCCAGACACTGGTGATTTTTCGATAAGTGTGATCAAATCTACAG
AGACTTCTGAAAGATTTTTTTTTGATAACGAGCCTAGTTCAGAAAACGTCGCATGTAATCATTA
ACTATCAATACTCCAGTTGCAGGCTTAATTTTTCCAAATTTTCCAAAACTACATAGGACATCAA
TATACTTGAAAAAACAATCATAACGAAAGAAAGATTATAGTTATTGATTTTTTTAAATGTTTAT
CATGGGTAAAAAATATCATAAAATAATAATTAAAAATATCATAAAATAATAATTATAAAAATTG
ACGATTGCTGCTGGAGTTAAGTCTGCGATCTCCCGCTTGCAAGCTTGATGCTTAGACACCCAGG
CTTATGACGTCACGCTCACAAGACACTGGCTGTAATGTTTTTAATGGGTTTCATACCAACTTTT
ATTAACGCTTGGGTCGAATTTTCAAAAACCCTGACTTCGTGCCTTAACTTAAGCTTCATATCCC
GTAAGTTAAATGCCCTGCCAATAAGTTCTGCAACTATTTAATCTGACAATAATTCATTTAATCT
CATATTGATTCAGCATATTTTATATCGTTCAATTCTTAGAGCTGTAACAAATGAACTTGGCCTA
GCCTCTAGCAGTTAATGTGATTTTTAATTTTCTATACAATTCCATTCTATACACATACACATAC
ACAGACACACATTCATCAGCATACACGCGATGTGAGTTTATTATTTTCATTTTTGGGGATGGTC
TGTGTCCAAAGGGTCGTGTCTTCGGGCTGTGCTGACTTTTACGAGCGCATCGCTCATACGCCGC
GTGGGCGGTGGCGCGGCAATTTTTCGCGTGTGGCAGGTAAAGTCTTAATTGAATTTGCTTTCCG
AGCGTTGGCTGGGAAACACGAGTCGCTGAGCAGAGCTGCTCGCAGCACACAGCATTCATATACA
TATATATGAATATGCATACACATATATATTACACATACGTCACGTATTTCTAAATGAAATTGTG
TACATTTTTTATTAAATTTTTGAGGGAATATTAACAAAAGGGCAGCCACTTTAAGGGGCGGCAA
GAAATGAGAGAACTTAAAAGCCAGAGCTTAATTTTCATTTATTGATAGCTTGAATCATATGACA
TTCTGGCTTACCTGGCATGGGACTTAAGTCCGCCCACTTTGAGCAGCAAACTGTCCACTCAGCT
ATAAACAAGATTTGTTATGAATGGGAAAGCTCAACTCAACGTAAATGTCAGAAAAAGGTATCGT
GTCCAGCACCAAAATGGGAGAGCACCTAAGCGGCTTGAGTTTGCCTTCTATATTGCCAACGAAT
TGGTCATTACTTGGACATGGTGACTGCCTTCCTATCAGCATCTAGTTGTGTCGCATCTTTCGGG
ACATTGTATTTTCATTTTAGAATTGTCTCAGCGATTTTCAGTTTATGACTCAAGGCAATAAAAA
CGCTGCCATTCATTTTCATCCTTATTTGACAAGAGCACATTGCGTGCTTTCCGGCAATTCATCA
TATCGCCGTGGGCTCCAAGATGCGTGCTCAACTTTCGCCTGGGTTTTGCCAGTTGTCTCAAATT
AACTTTAATAAACTATGTATATATGAATTTTCTTCACAGCCTACCCGTCACTCAGTCTGTGCTT
TGATTAATAGTTTGGGGCATGTTTTAGCATGTCAAAATGTAAAAGTTATGCTGGGTTTTCGCCC
AAAAAGTTGTGGTTGAACTACAAGAGAAATATTAGGTATTAAAATGCATATTATGTGTACTATA
TATAATGTGTGTTCTGCTTATTAATTTTAACAGTCTTTTATAGATACACATATACCCTGTAACT
AGCAAAATGAAGGCATCTCCTGCCCCATTAAGCTTAAATATTCTCTATCTGTTTGTCGACCTGT
TCGTCTGCAAGAATATTAAGATCTCAGAACCTATAAGACCTAAAGAACTTACATTTTATAAGTG
CACTTACTTATAGATTATATATGGAATATAATTATATATTAGACTTAGCTGAAAAGCCCATATA
ACGTACCAGGTCGTGAAAATCCAACACTTATGAGCATGATAATAATACTATATAATATATTTTT
AAGTGTCAGAATTTGTAAAAATATATAATAAGAGGTTCTAGTCGGGAGCTTTATATTTCTTTAT
AATTTGCTCAAGAAACAGGATGTCGATATTGATTTTTATCGTTTGCTTGAACACGGATCAAGTT
ATCGATTATCGGATCCTTGCGTTCATACATACGGACAGACGGACAGACAGACAGACAGACGGAC
ATGGCTAGATCCACTCGGCTAGTGATGCTGATTTTGAATATGTGTATATACACTTTTTAGCATC
GGAGATGTTTCCTTCTGCCTGTTACATACATTTGTACAAATACATAATACCCTTTTTATCTATT
TTCAATGGGTTCAGGGTAAAATAATATTATTGTAGTTAAAAACAAAAACATAATATGTATTTAT
ATTTCTATAGTCTCATATATATTTTTCTATTAGTTTTTATTATTTCGCCCTCGAAAATAGGTCA
TCAAGTAGAGCAAAAGTTTAATAATTCAGAAGTCATACCAAATTTAAATGTTCAGTCTTATAAC
TTAATTTAATTAGGATTTCATTTGTTGTCGTTGTTGTTCTTGTTGTTTTTGTGCTGTTGGCCAA
CTTGCTGTGCACACATACTTACTTGTTTGTTTTTCTCTTGGCCCTTTCAGCCAATTTAATGCCA
AGTTAATTTTTCAATTTACACGCAAAGTGTCAGAGCAGTTTTTCGGGGAGGACGAGGGGCCGGC
CACAAGGGGCACGCTAGGGGCAAGAGCAACACGCATAGAAGAATCATATGAAATTGCAAAGTGG

AGTTGGAAGGAACAACTTTGCCCGATAATAGCTAAAATAAAATAAATAATAATAATAAACACAA
AATTATAATAAAGTGAAAAAAAAAAAAAATATTAAAAATCAACAACAACAAAGGTACAATTAAGC
CAATTAAGCTCTAGCTGCTGCATAATTTGTTAACAAAAAACCAGAGCAACAGAAAGTTGAAGAT
TTTGCGGCAAATAGGAGCACGACCGACAAAAACGAAAAATATATGAAATAAAAATGCAAATT
>Contig2813:13975..5192 (rev_compl) no overlap
AACTTTGCTGCCACTTGCCACCTGCCACTTGCCACTTGCCACCTGCCATGCTGCGCCGTAACGC
GTCGTTGCCCGAACGCACGCAAAACTTTGGCTCGTCTACGTCTTGGACTTTGTCTGCGACTCCG
ACTGCGACTCTGGTTCTTGCAACTTGTAGGGCTGTTTTAGGTCCTTAGCGGCACTGCGCCTGGC
GCTGGCAGGACCTCCTATCCACTCGAATCAGCACAAAATTGCGCTCACTTAAATAAATTTATGA
AAATATGCAAGCACGCGTCGAGCTCGAGTTAAGTGCAACCAATTGTCGGTACAAGATTTAGGTC
TGCGGCTGGACTCGGTCTGCATTTTGGTGGTGGCTTCTAGCCACATTGAACAAGAACCAGGGGG
TTACAGTTCGAGTAGGAGAGAATGAGCATGATAATGAGAAAAAGAGGTTCGCTGAGAGACACAG
AGGGAGAGTGGGCGAGAATGTGAGAAGGCAAGAGAGTTATTAGAGTGGGGCTACAAGAAGCGGT
AAGGCTTATCGGATTTCCGATACAACTTTCTTGTATCAAGTTTGCAAGCCGGACTCGAACAGAA
TCGGCTTGAGTTCGAGCTTGTCTTACTTACATTAACGAATAAGCTTACCATGACCTAATTAATT
GTTATACCCACACACAGTCATCACACTAAATAAGTGGTAGGAGTACATATATAAATATAAGTCG
CAACAAATTTGTTTATTTGTGCGTATGTATGTAATAGGCAGTTGGAGGCGTCTCCGACCACATT
AAGTAAATATATTCTTGATCAGCATCAATAGCCGAGTCGATCTAGCCATGTCTGTCCGTCCGTC
CGTCTGTCTGTCTGTCCGTCCGTCTGTATGTATGAACGCAAGTATCTCAGAACCTATAAGAAAT
AGATGTAGGTCCTTCTAGTGCCTGCGCAGATCGAGTTTGTTTCCGATAATCGATGACTTACTCC
GTTTCCAAGAAATCGATAAGACTCGATATTCGACATCCTCTTTTTTTTAACAATTTGGTAAGTA
TTCAGAGCTAGAGTCACCAAACATGATATATGGCTTCTAGAATATTATATATATGTCAAGTGTC
TTTTATTGTATATCTATCGCCACTTCCCAGCTACCACCCCAGAGTTATAAATCAAGTTAATAAC
TCAACTTATATTGCCAACCAATTTAGGCCACAATTGTAATGCAATTGACTTTTTGAGATAACTC
AAATATTCTATAGTACAATAAGATGTACTGTAGAAAATTTCATCAAGATCGGTTAAGAGAAAAC
CAAAGTTATTTGTAACTGTGCAATCGGATGGCACAGCTAGCGAAAACATCTAGAATTTATGTAT
ACATGTACACACACACACATTCGCATTCTATCGCATTCTAACAGCATTGTAATTCTTTCTGTAA
CGCTATTTTAGTTGGTTTTTTTTCTCAAGGATACTTAATAATTGGTGTAGCTGTTGTGTAGAGG
GTTGGTTTTCAATTGGTATGACCATACACACATGCATACACATACACATGCATACATGCAATCA
ATGAAGCACGCCCTAAATAAGTTTTTAATTCCAAATATTTTTTAGATATTATTTTGAAGTCGGT
CGGACGTTTCAAAATGAAAAGCTAAAAATTCCATAAAATTCCATATTCCCAGTCATAGTGTGTA
CTCTTATTACATAGGGTAATTAGGGGAATACCTTACCCTGTCTAAATCCTACTTTTTGGATTGA
TCATCAATCAATCAGTCAGACAGTCAGTTATATATAGAAAGGCATTCATGTCTATGTAAATATA
CATTTTTAATATACGCTAGCTCTAAGAAATAAATTAGCTTCTGCTGCATACTTCTGCATACTAT
TATATTTTTATTGCATTGTATATATTTATTGCATTACTTCGTTTACCTTTGCAGCCCATTACTT
ATCCCTACTATCGTTGACATAAAACCCAACAATTAGTTTGCATCTAGTTGGAACGTCAAAACGT
TTTACAATGCAAATTCTCCTGTCAACAGTCGGAGGCTGAAACGCAAACAGTTTCACTTATAAAC
CTGCACTTTACTGGATGTTGCTTGCATATTATTTTAATTAGCAACTGTCGCTCAAAGAAAATAC
CAAAAGCAACAACAACTTATGGCGACAGCAGCAGCAGCAGCAGCAGCAGCAGCAGCAGCAGCAG
GAGCAACAGCAACAACAACAAAAACAAGTGGAATAAAGTGTCGCCTGCTGGTCTAGCCTTTGGG
GCGGGGTTTGGGGTAGGCGCTGCGGCAGCCCTCTCGTTAGTTGAGAGCAACAGCAGCTGCTGCT
GCTGTTGGCATGCATTAAAACTTGTGCTGGACAAGTATCTCACACCAGCCCTGGCAGACACACA
CACATACACGCACACACACGCACACGCAAGCACTCAGGGCATGACTCGCGAAATGAGCGCTTAA
CGGGAAGCAACGTTTTTACTTCAAATATCCTACTTCTAAAGGAAAATGGGGTATACTGATTTA
TGCTGACGCTAGAGGCACCAAAATCTACAATTAATATTTGTGGATACTCGCTTCCGAATTGGGT
TTGTTGGCTTTTTGATGATCTTTTAAAGATCAAGCCAAACGAGCATTTTTAACAGTCAAGCAGT
TTGCCAGTCAAGTGCTCTCAGTTTTAGATATCATCAATACCAAAATAATTAGCGTTCCGCTCAC
ATGCGAATCCATCATCAACAAAGCTGTCGAACACTTAGTTTATGACATTGTTATAACACTGCAA
CGTTCATTTCATTTTGTACTCTGTACTCAATTCGAAAAGTGCATAAATGTTAACTGTAAAGAAT
AGAAGAAAGCATTTACGACCTAATAAAGTATATATATTCTTTATCAGGACGAGTGGAGTCGATC
TACAAACTCTTCGATCTCGGAAACTACAAAAGCTAGAGACTTCACATTTAACATATAAAATATA
TGGCTCATATATCTTACGCATACGTGGTTTTCCCATATAGTACACATATTTTAAGAATAGCTTT
GAGATACGTCCATTATATATTTATATTTTAAAAAGTTCTTCTAGTTTAGCGCATCCCCCACTAC
TTGCTTTATACTTACATTCTTGCTTTTACCTATCGCCTCATTTGGCTTGGTGTTTTTGCATTTC
GCTTAAACAGCACTACGCACACTTTCCCGTCGCACACCCTCGCTTACCCCCCTGCCCCTACGGC
GTTCTCTTGACAGAAAGGCATGCCGAGGCATTGCGTGTTGTGGGAGCAGCGTGAATCCGGTCCT

TAATTTAATTTACGAAAAACAACTCGCTTCCAAATACGAAATTGGTGTTTAAGATAAAACGCTT
AATACATACTTTCGACGGTAAATTAAAGAAACTGAAGCGTTTTTGATTACATATAATTATTTTA
AAGTGTTTGATTAGAAGCTACCCTGTTCTCTGCGAAACGGCATCAAGTATACGTTTTTGATGAA
GATTCCGACTTTTGCCAACAATAAACTACTAAAGCAGGAGAGTCCCATGCAAAGCCTTAAGCCT
GAGGGACTAGTTTGCATAGAAACAGACAAGGCGGACATGACTATATCGTCTTAGCTGTTCATTA
TGATCAAGAACACACATACGTTATAGGTGATGACTCCTTCCTTTCACTTAATACACTCAAGGGG
TAAGGAAGATGAATGGTAAATCGAAAGGGCTTTGACGAACCAAGGTTTGAGAACAGTCTTGTCA
AAGCTAGGTTCACGAACGGAACCTTAAAGCGATGCATTGGCTTAAAGCGGTTTTGAAAACCTTA
TTATAATATTTGAGCAAATTACCAGCCAGAAGCTACACTGGCACCCTGTAGGACGTCTGGTTGA
TTTGTAAACTGGGCATAGGCTAAGCCTAAAAAACCGCTTGCAACAATATTCCATAGAATTCTTA
TAAGTACAAAACTGAAAAAAAGCCATCAGAATTTAAAAGGGGGGTTAGTTGTAATAATACTTAA
CATTGACCCAAGACGGGTTCATGTTGGCCTCGCCACATTAATTGGTAAACTTTGCTCAAGATCT
AAACAACTCTTAAAGCTAAGCAAGTATTGCTGAAATGCCCAGCGTGGACAGTTTGGGGCTTCGG
CTGTGGCATTGCAGATTGTGGGGTATCATTTTGTATGGCTGCCAAATTTAGCTAACAATTTTTG
GGCCAGGGTCGGCCATGAGGCAAATTGGCCACATACCAGTGTTAACCGAGTATGATTAGATTTT
GCTTTTAAGCGTTTTAGCAGCAGTTGTTGAGCTTGCGGGCAAGGCAACGGTGCTCTGTCATGGA
GAATCCAATCAGGTGGCTGTTGACAAATGGCTTATGGCACGTGCCTATGCCGCTGCTGTTACGG
CCCTCCCCAGCAGCCCATTTACCGTCACAAAAGGTGTGTGAAAACTGGAGTTTGCAAAACCATT
TGCCACTTAAGCAAAAGTTTCAAATACAATTTTAACTAGAACAACAAGAGACAGCGCGCACGAA
ACTTTCCGCGGGCCAAATGCAAAAAGTAAGCTGTGCCCATGGAATAGATGGCGGGAACGGAGGA
AGGTGCCTTCAAGCTAGCGCTGGAGTTGGAGGGGCTGCCGCGAAAACAAATGAAAAATGCGCAA
CAAAAACAAAGAAAAGTCGAACAAAAAAACATGCTTGAAAATATGAGTTTCTGTGTATGCGAAG
AGTATAAATTAAGTTGATAAAGTTCACTTGACCCCAAGTAACGATCCCAAGACATGCCCGAGAC
CTCCTGCTAGCCCTGGAGTCCGCAACGTCTTCAGTTTTATTTTTTAGCAGCAGCAACAATAAAC
AATACATTGTACAATGTAATTTTTTGCAAGGCCACAACAAAGCCAAATGAATAAAATAAATAAG
AAAACTGTCAGGTGCCGACGCAGTGGTCAAGGTGGCACGCCCACAGAGCCCACAGCGTTCCACT
GGCACAGCTGAGCTGAATTAAAAGTTTTTAACACATATTTACGAGCAAGTGACAACAAAAAAAC
AAAAAACAACGAGCCAAATGCCACTAAAACTCTGAAAAACAAAAAACGTCGCGACGTCTAGTCA
AATTGCAAATGATTTGGCTGAAACCCAATCAGCGGGTGGCCAGTCGAGCAGCGCACGGATGGGG
GCTAGCAGGGTGTGTGGGCTGTCACTGTTAGTCTGACTCTGATGGGGGCCATTGACACATGCCA
CGCGCCATGACACCACTTGCCAACAACATGACTAAAAGGATCATTCGCTTTTTACACTTTTGGG
AATCTGCGCCTTACTGAAAAAGTTTCCGAACGCTTTATTTTGAAATAGAAATATTAGAACGAGT
TAAGTGGACAGCTTTCAATTGATTAATCAAGAACGATGCTTCGCAAACGCAGAAAATGCCGCAG
GGGTAAGCACACAAGAGTAGACTTTGGTTTCGCTAGCTTTGTTGCCTTTTTACTGCTTATAATT
CTCAAGTGGTTACAGTTTCAAGCTGAGTAAAAGGCCATGCGATTCGAAAGTTGATAGCAAAGTC
TGCTGATGATAGAACTGGGGGGTTTTACACATAGTTCTACAGACAACCTGCTCAATCTTGGTAC
TTATACTACCTCAAGCCGTTCGGCTATTGACTTGACAAATTTCATTTACAAATTTTGTAAATAA
ATAAGTGGGCTGCTTACGGTCTATAAATTATAGCAGATTTACTACTTAGAATGTCAAGATAACA
TTTATTTAATAAGAAACCGTGTTCCCAGAGTAGAGCAGAGTACATGGGCCCGTCAGACAGCAAG
TACACTTGTTATACTTCGGACACTTGTTATATATGTAAGTAGATACAAATCTGGTAGAAATCTA
TATCATATCCAACTTTTATGTATCAGTCAAGGCCTAACCCTACAGAGGTTATTAAAACTTTGTC
GCAATCTGTGAAACGCATAAAAGGAGAAGTCTGCGGTTGCGAAAGTATATATGGGAACATATAT
ACATTTTTGACCAGTATCAAGAGCCGAGTCGTTATAACCATGGCTGTTTGTCTGTTTCTGCTAC
TCTGTTCTACTCACTCAAAAAATTGTGTTCATCAGTATATCTTCACCAAATTAAGCATTAATTA
GATCAACCATGAACACTCGAATCAACATCGCGTGCTCAATAGAAAGAGTCAGTCGAAAAAACGG
AATATTGGAAATATCTTTGATATGTTAACCATAACAGCATAACTGCAAATCCGTTTCCGTTACA
AATCGGGCTTCTATATCATTTTACCTAATGAAAGGTTTTCTATGTCATATGTGTATAAACCATT
ATAATTGTTACTATTATTATTAAGTTTTTTTTTTATTATTATTATTAATATATTTAGTATTATG
ATCAGTATTATTCTGATTATGATTAGTAATTTAATCACTATTAATATTATTATTACCATGGGAG
TACAGCAAACTAACTATCATTCTTTGGGAAGGATAAAACTATATTAATAGACTGGCGTGGACTG
GAACGTCGGTGCACAGTGTTTTACGTATTATTCGGCAATATATAGTTGAAAACTATATAAACAC
TCACAAACTGTTTGCGGATGTCCGGTGCTTGGCAACACGGCTAGTTTTGCATTCGACGAGTAGT
CGGTTTTTGTTAGTGAAAGTCATTCTGTGCGCGGTGGCGTATGCGTAATGCGTTTAGAGTTAAT
TAAATTTGGTCATGTTTTGCGTGTGTGTGGTATTGGTGGAGAGGGATGCGAGAGGGAATGGGAC
TGGGGGCTTGGCAAGTGTCCAAGCGAAAAGCCAATTCATCATGGCACGCAGCTAAAGTCATAAT
CTACTTTAACAACTAATTGCCATTACTAAAATTATCATGATTATGATGATGATTAAGATTATGA

TTATTAAGTATGGACATCCTTTTCAGCAGGCAAAAACTTTTGGCTAATGGCCTGCCACAGTGTA
AACTTATTCCTGTTGCCATTTCGTTGAGGCCTAGATTTTGGCTTTGGCTTTCGCTGTCGCTTTC
GCTTTGGCAAACCAAAGCACTTTCCCTACAATATTTTACAACTAATTAGGGATAATTACCAGCC
ATTACACAAAAGCCTTCCCACGACGGTGGCAAGTTGAAAGTTGAAGACAACTGACTCACTGACT
GACAGGCTGACACATTTTTGGGGCGTGGCTCTGTCTTCCGTTCAGCCCCTGTAATTTGGGCCAC
CTTAGTTATTATTACTGAGCCGTTGGCATACAAGGCGTCAAAGGTTCCCCAAAAAGCCTTTGAC
TAACTCCATTGAGTACTTCTCATTTGTCTGTTGATACAGGAGCAAATGTCAAAGTGTCAGCCAT
CACATCCTGTTCAAAAATAGTTGGTTGGATCAAGCATCAAGCATCAAGGGTTGTCATTTATTTA
TGTCTGCATTTCCTGATTATACTCAACAGGTCATACTTCTGTTTATATTTTCGTAAAATTGCTT
AATTTGATGATAAAAAATTGTATTACAATTTGAAAATACAGTTTGTAACCATACTCTGTGCCCA
GGAGAAGCAGCGTGTACTGCTTGTAGGCAATGTCCATCTTGGAGGCCATTTATCTATCCCTCTA
ACTATGTGAACAACATTTATAAATAAAGGCGAGTGCAAACTGTGAAGCTAGGAAACAGGGAATC
TGTTTATCGAATAATCCCAAGCTGGAACCGATCATATTGACTTTCTAAATAGCACCAGCTATCT
TCTGGTAGGACAATGCTACACCAGGCATTCAAACTCAGATAAACTAACATATGAGACTATGTCT
TTCTTAAAAGATACAATTATCTCGTTATAGCTAAGTAAATATATGATCAGATTGGTTAAAGTGT
TGCAGCAGTCTGTGATTTTCACACAATGAAGAGCCAGCAAAGGATTATGGCATGTCGCGTGTCC
TTCCTGGGAGGTGATCAAATCCGATTTACAGTGACACAAACAGCTTCAATAGCTTAGCAGAATA
ACAATAGCAATAATAATAGTACATGTGCATGGGTGCACTTGCAGCTTGGAGCTCGGAACTTAAC
TGTTCTAAAGCGGTGAAATGCACTTCCCTTTAGTTTTTCGCATGCTCAGACACTAAGACGCAAA
TAAGTTACTGTGTGGGCGTGTGAGTGTGTATGTGTGTATGTGTGTGTGTGTATATGTAGCTG
CCATGTTTTTAATCCACTTGAAGGCACTTAAGTAAGCTTTCCTGCAGAGCTGGCTGGTGTGTGT
GCTTGTGCCTGGTTGCGTGTGTACCAAGTGCTTTCGGCTTGCTTTAAATTCATAGTGCTTATGA
GTAAATACAAGAGTGCTCGAATAGAAGATACTCCATACCTAACTGCAGCCATCTGCCTAGAATG
AAAACAAATTTCGCTCACCGCAAATCAAGTTTTCATACCATTATCTATTTGTTCCCTCTGTCTA
TACACAATATTTAAAACTGTTTGTGTTGACTGAAAGCTAAATTCAAAACATTAGTTGTCTTTCT
GATTCAATTTAAGCTTGGCCAATGAGAGAGGCATTTTGTGAAATGAGAGTGGATATACTTGCTA
CCTCATAGATTAGCATTTCTGACAATTCACTTCCTTATAGTATCTATGGTTGATTCAGCCAAAG
TTAAATTTGCGTTGCCTGAATCTTAAACATATTTTCCATGCAGTCTTGTGAAAACATTTCATAT
TCTTCTTTCTTTTTCAGACAGAGCATATCTGTGTAAAAAACG~~GCCAAATCGAATCACTTTATTG~~
~~TGTAGCTGCCATTCGAGCAAAACCCAACTCATTTGAGTTGTACGAAAAACACTAAAAACCTCCG~~
~~AGTTAACCTGTTGATG~~
