# Supplementary material for: Origin and Consequences of Chromosomal Inversions in the virilis Group of Drosophila
Source: Genome Biol Evol. 2018 Oct 30;10(12):3152–66. doi: 10.1093/gbe/evy239 (PMC6278893; doi:10.1093/gbe/evy239)
Supplement: Supplementary Data [file evy239_supp.zip › File S4.pdf]

## Ancestral state:

### *D. virilis*

### Distal region

>Dvir scaffold_12928: 7,239,906..7,243,220 (*GJ17050*[+](*Ir7g*) - *GJ16378*[-](*CG10932*))

CTGCAGCAGCGACGCGCTCACCTGGCCATTGGATATATACGCAAACGTGTGCAGCATGCCGCCA
ATCTGACGGCCGTCTTTCCGCACTATTCCAGCCGGTTGATCGGCTGCCTGTCGCTGAACGCGCA
CAATTTGACCAGCTTTGAGCTGTTGGGTTTTCCCTTCCAGACGCTCGCCTGGCTGGGCGTGCTG
ACCAGCTTCCTGGGCGTCAGCTGTCTCATGTTGTGCACGCGACGACGTGTCGCCAGGGCGACAA
TGCTGCTCGCCGTGCTGGCGATTGCCTGGGGCCAGCCAATTATCGCGTCCGGCCTGCGTCCCGC
CCAGCAGCTGGTCTACATCAATTGGCTGGGCTTCACGCTGCTCGTGCGCGCCATGTACTCGGCT
CTGTTCTATCATATGCTGCGCCAGCAGGTGCATCAGCGTTTGCCCCGCAATCTGTTCGAGCTAA
TGGAGGGCGGCTATACGGCTGTGATGAATCGCATTACCGCCCAGGATGTGGGCGAGGTGAGCAG
CCTCCAGGTGCTATTGAGCAATATGCGTGCAATTGTCCTTGCAAGCGATGTGGAGCACGATGTC
CTGGAGCAGGTCGAGTCGCGGACAACGCAGCGGCGCATCTTTGGCATACTATCGCGCCAGACCC
TGCTGCATGCGGCACAGCGTGCCCACAAGCCGGGCGCCTACTATATGCTGCCGCAGCATGTGCT
CGAGCAGCAGCTGGCCATCTATCTGCAGAAGCATTCGCATCTGGTGCAGCGGCTTGATGAGCTC
ATCATGTCCATTCAGGCGGTCGGCCTGATCAACTACTGGGCCGGCCAGTTGGGCAGCGAGCGCT
ATTTTCGCAGCACATTCATGTATCGGGACAATCGGCTGCGCCAGCCCGATCTTTGGGGTATCTA
TATCATTGTGGGCGTGCTCTATGCCCTCGCCACGCTCGTGTTCATCTGGGAGCTGCTCTCGGCG
CGCCGGCGTCACGTCCAATAG~~TCGGCGAGGCTCAATTTCATTTAATGTCCAATTCACAAA~~GTGC
TCACGAGTGAAAATAAAAAAAAAAATAAAAAAACGAGAGGGCCACACTGTTTTTCAACTGTAC
GATATCGATAACATCTGTTATCGTTGTTGGTATTAATCGTTTCTGGAAGTGCAATTGGAACGGC
AACTGACAGAAAGAGTGAGATGACAAACAGCTCTAAAATGTCCGATAGCGATAACTTTCCAGCT
TTCAATAAGGTCAAAACATATTTAACATGTTCGATAACGATAACAGCTATAATAATAATTATTT
TTATGCAATTCACCTTAAAGTTCTCATGTTCGATAACGATAACGATAACAGGCGTCATCGATTG
AAAATTTCACTTAATGCAAACAGTTCAAAAGGCGGAAGCGTATTCCAAATTTTCGATAACGATA
ACAGCTAACATCGATGACCATTTAAGTCATTTGTTTTTATGCAATTCTTAAAGTTTATTTTGCA
AATCACAAAGTTTTCG~~ATTAAATGCTACACAAATAATAGACAAAAATTCATCTTTTCAGAGAAA~~
~~ATGAGAGTAAAAAAACAGTTTTAATGTTCGATAACGATAACGATAACGATAGCTGGCGTCATCG~~
~~ATTAATAATTTGAATAAATGCAGTTCTGGCTTTAATTGTTGCTCTTGTTGTAGTTGTTGGTTTT~~
~~CATGCTGTTTCCGCCAGC~~TTAGAGCTTCTCGAGCAGAATGGAGGAGGCGCCGCCGCCGCCATTG
CATATGGAGGCGCAGCCCAGTTCGCCGGCTTTGAGGGCATGCGCCAGATGGGTGACCAGACGGG
CACCAGACATACCAATCGGATGGCCCAGGGAGACGGCGCCGCCATGAATATTCACCTTAGCCGG
ATCGATGTCCAGTTTGCGTATATTGGCCAGCACGACCACAGAGAATGCCTCATTGATCTCCCAC
ATGGCGACATTCTCTTTGCGGACACCGGCACGTTCCAGCAGCTTGGGCACCGCCAATGCTGGCG
CAATGGGGAAATCAATGGGATCCGTCTCGGCATCCTGGAAGGCGACAATGCGCGCCAATGGCTT
GAGGCCAGCACACTTGGCCGCCTCGGCGGTCATCAGGACGACAGCGGCGCCGCCATCGTTCAGG
GTCGAGGCATTGCCGGCGGTAACGGTGCCATTTTCCTTTTGGAACACCGTCGCGAGCTGGGCGA
ATTTCTCAAAATTGACGCGCTTGAATTCCTCATCCTCGGCAATGATCTGCTCCGGCTTGCGCTT
AAGCTGAATCTTTACCGGGGCAATTTCATTGGCGAAGATTTTATCGCTCCAGGCCTGGGCCGAA
CGCTTGTAGGATTCAATGGCAAAGGCATCCTGCTCCTGGCGTGTCACCTGCATCTTCTTGGCGG
TGTTCTCGGCACAGTTGCCCATATGGAACTTGTTGTACACATCCCAGAGGCCATCGAACACAAT
GCCATCGGTTAAATTGACACCGCCATAGGGCGTGGCACCGCGCTTCAAATAGTATGGCACATTC
GACATGGACTCCATGCCGCCGGCAACAACGATCTCAGCCTGGCCGAGCATCAGCGACTGGGCGC
CGAGCATTATTGATTTCATGCCCGAGGAGCACACCTTGTTGACGGTGGTGCAGCACACACTGGT
CGGCAGGCCGGCAAAGATGGCTGCCTGACGTGCCGGCGCCTGGCCGAGTCCGGCGGACACAACA
TTGCCCATGATCACCTCGCTGACATCGCCCTTGCCAATGCCGGCACGCTGAATGGCCGCCTCGA
TGGCCGTGGCGCCCAGCTGTGAGGCCGTTAGCGGCGCCAGCTGGCTCTGGAAGCTGCCGATCGG
TGTTCGTGCGGCGGACACAACAACAACATCGCTGATCTTTGAGCTGTAGCGACGCGCCTGGGCG
CACAGCTGGCTTCTGGTTGCTTGCAACAT~~GGCACTAGATTTATATAATCGACTTCCTGCTAGTA~~
~~TCGAACGATCACTGGAATTTTACTACCGGACCTGATCACACTTCCAATAATATTGAGGCTGAGA~~

~~ACCTGTTGCTATCCACTTGCGTAACGAGCCGATGGCGTGTGAGCAGCTGTTGCGGACAGTGTTG~~
~~GAAAGTGTAAAGTGGAAAATGGCAAGTCGCTTGCTTGCAAGCTTTTCGTGTCCGTTCGTGTTTT~~
~~TTTGTTTTTTTTTTTTGTGTTGCCAATTAATGCAGTTTTAGTATTTTTGCTTGTCATTGCGTTT~~
~~TTATGCGTGCCGTTGCTTTAATAAAGAATACGACAGAATTGTTAATACTCTGTTTTCAATTGCCG~~
~~GCTAGAAACGATTCCGGTTTATCTTGGATTAAATTTTTTGTTAATCATTGC~~

**Proximal region**

>Dvir_scaffold_12970: 9,107,414.. 9,127,509 (*GJ18715*[-](*CG32698*) - *GJ18714*[-](*CG1354*))

CTAGCATCTTCGAAAGAAGTAGAAGGCATGTGTAACACAGCAACTGCAGCAATTCCAT~~ATTTCA~~
~~TCAGCTAGAAGAATGCGGTGAATCT~~TATAACTTCAACGAGCCGTTTCTTATTGTCTGAAACTTC
AGAGTGGATACTTATACATCCAAAGCGAAGAAATTATTGCGTTCAGAAATCTATAAAAATATGA
AGAATAATAGTAATGTTAAGTGCTGCAAAAATACATTTAAAAAACAAGTAACAGGGAGTGTCTG
ACTAAGGGATACTCTGAACCTCCTTCTACTAGCTTTAATGACAAAATTTAATAAAAAATATCGT
TTTCTCTGCCGGTTAAGAAATAGCAACCATGTTCAACCGACTAAACTAATTGCCTACGCCAAGT
AAGAGGCTTTAGTCAAGAGTTGCTTTAGCAGAAATTAAAATAAATTAACAGAAAAATTGGTTCA
ATTGGTTCCTCAATAAGACTCGAACTCGTAAGCTAAAACCAGATATAAAACCGGAGAGGAGACA
ATTTCTGAGGTCAGTTCTCTTTGGGAAGAGATATCTCCAGAGGGTATTAGAAATGTTATGAATT
TAGCACCAAGGCGCCTTTTAAAAATTATTAAAACCAAAGGATATACACTTTGCAAATTTCTTCT
TTATTTTTGTACTTTTGTTAAGCATAAACATTATTTTCTCGATATAAAATTCCTGCCAAAATAC
TTATGCCAACACATGAAATTGTATATTTATATTTGTTTGGTTATTTTTAAGATTGTATTTATTA
TGGTTTAAAATTCAAAGCTGTTTAAAAGTTGAACTTATAAATGCACGATTGTTCTCTCGTAAAA
ATACTTATGTCGACTAGTGTACACCGGGAATAGAATTTTAATGAAAACAAGTTAATTTAAATGT
CTATAGTAATTCTTGTATTTTGCCTTTGCTGTTTTTTTTTTTTTTTTTTTTTTATTTGGTTTAT
TTTGCGCTGGAATCGATCAGTGCCTTCTGATCTCTACCAAGCGGAGTTAAGGGTATAGTGTATA
GTTATTATTTAATAACTTAAATGATAATTACAGACTTAAAAATATATAACAATAATGGGAAAGG
GATGTTAAAGTGATGTGCCTCGTTGGTAGGTCGTCGGGGTGTAGCCTTCGGAGGCGCCTCTCAC
TGATGGGCTCAGCCAGGGCCCTTGCGGCCGCGTTCGGATGCTGCACCAATTTGTCGGAGTATCG
GCTGCTGAAGAAGTTGAGCTGATCTCCGACCTTTGTTAGGCGGAGATCGCGTTCGATGGCGTCT
GTTGTTACGTACCATGGCAGGCCAGCAACCTTGCGTGCTGCCTTGGCTTGGACCACTCTTATGC
GACGGAGCTGGTGTGCTGATGCCGTGCCCCAAACTTGGATCGCATATGTCCAGATAGGCGTTAC
CATCGTTTTGATAAGCAGTGTCTTCGTGGATATGTGCAGCTTCCTTTGCTGTTACGTGGCTCTG
CCTGGCCTAGGGCCAGTTATGGGCCACTAAAGTGCATATCGTCGCAATGAAGGCCTTGCCAAAA
CCCTAGAAAAATACCTAAACCCTCCATGCAGTTCGCAGGACTTAACACCGAAGTCGCAAGGATA
AACCCAGTTGCGAGGCTATAAATGCACATTTTGCAATATCAACATCACGTTAACTACGTTGCTG
ACATCGATGTAAAGCTCAGCTGATTAACATACATGCTACTTATTATTATTCTTTTTACTCGGCC
ATGTTGTGTTGAGCTTTCAATATAATACGCGCCTTTGATAAGCAAAGCGATCATCCAGATGCAC
ACAAATATTATTTGTTTTGACATGCAAGCTTTTTGATTTCTGATGATTCTTAGTGAATACACAG
AACTATTCAATTTTAAGTATTATTATATACTTGCACAGTTTAAAAGCTATCGCAAGAAACTTTG
AATGCGTCCCTTTCTCTGTAACGCACAGTACATACATGTATATCGGAAGCGGCTGGATCGGACC
ATTATATCATATATGTAGCTGTCATAGGAACGATTTGTTTTTGTTTTATGTTTTTCAAGATATC
TTGGTATTTTTAATTTGATCATCTTCCGACCAATATATACATATGTATATGCCATATGAAAAAT
CGACCGAAAATCAAGTTCTTGACGAGCTTCACTAATCTTACATATCTGTAAAGCCGTAAGATAT
ATTTGACAAGATTACTTTCTCGTTTTGTATTGATTTTTCGACAAGACGACTTGGTAGTGTGGGG
TCTCGGACGAGAAAAGAGTTGATTGAGTTACCTGTTTTCCTTGTCAATCTAAATCCAGAGTCGG
GAATTATTGACGATAGTGGGTCTTTAGTGAAAGGATTATTCAACTTTCAGTTATGCTTAGGATG
CTCATTTAAAATAAAAGTTAAGGAAAATGTTTAAGCATATAGTAGCTGAAGACTTGAAGATAGA
GTCGACAGCACCTGGGATTCTATGACCCGAGCTCCGTAAGCTGAGCGGGATGATGTCGAGTGTA
ACGCTTGATTTCCTCTGACATAAGTGGCAGCGACTTTACGCAGCTTTGTTTTTCTGTGTCGCTG
TTGTCCTTATTGCGTCATCGTTGTCAGTGTTGCTGTCCTCTTGCTCCTTCAACAACGGGATTTG
CAGTTTTGTATTTTGCTGGCCTTTCCTGGGTTACTCGGCATTGCCATTGTGTTGGACAACAGCT
GTAGCAGTGGCATCAGTGGCGATGGCGAAGAGTGCATGTTAAGTGCCTTATTATCCTGTTTATG
TAACTGTGTAGTTTTTTTTAAGGTTATACTCGCGGACATATGTCTTCACTGGCAGCTTGTTGCT
GCGTGTGTGCTTTATCAGCGCTAAAGTATTTTTCTCTTCATATTTAATTCATCTCAGCTTACAA

ATTGTCATAATGTCCTTTTTCCTCCATGTACCCAAATACGTAACATTTTCAACACAAAGTGCTA
ACTTTACTTTTTCCTAACTAACGAAGGCAAAGATGCAATAGCCAAAATTGATGAATACAGAACC
AGCTGTGACACAATGCAAATTTAAGCTAGAACACGTATCGCATTTAGTTTTTATACATATGAAC
AACATATATATTCTGTTCCATGCAAACTAGTTCTTGAGTAGTGAAGCTACAATGCAATGCTCCT
GAAAATCGTCTCAAATGCTTTTGCATACATTTCAAATCCGAGCTCAAGTCGTTTGAATATGTAA
TAAAAATATCTTGTACACACACTACATACAGAAATTGGCCACTCTTTAGTTGGTCCAAGGTAAA
ATACAGACAATTGTCTTGGTATTGGTGTTGGAATAAAAGGGTTCAGGGTATTTCCTAGTCGGTA
GCTCCCGTCTAGAACCTCTTACTTGATATGTATGGGTAATGTAGACAGAACAATGAAAAGTAAA
CAGTAGATAGTATTTAAACTGACCACTTCATCACAATTTAATGATTTAAGCAGCCGCAAAAATA
TTAAATTTATTTACCTTTGCTTATAATATTTATAAATTGTCTCCAAAAAGTATTTAATAATGAA
TATGCAAAGGTAAATGTGTGTTTTTCAACATTTTAATATGCATGGCAGGGCTGACATATGCAAA
AAAAGGCCGACAAAAAAGGAAAAACCGCTAACGGCAACGAAATAAATTTGTCAGCCATTGCAAT
ATTCTTATTTTCTCTGACGACGAGCTGCAACGAACTGGACCTACCCCATCGGCTGGCTCTGGCC
ACGTAAATAAATAAATGCACAAATATACATATACATATATAAACATTATATATATATATATTAT
ATATATATATATATATATGTATATATATGGACGAACTGAGACCGTGACTGAGGCAAAGGATGCA
GGCGACCTTCCATTTCCATGGCCATTTCAAGCCCCTTCCTGAAATTGTGCATGAAAAATATGGA
AAGACATTTGGCCAGAGACAAATTTTTGCAGCTGCATCCGCATCGGGCTGATCCAAAAACTGAC
AATAAATGTCATTTAGTTTACCAACGTACAGATGCAAACAAACAATATTTATTTAAAAAAAAGA
ATTATAAAAACGATGGTCAATTGGAGTTTCTTGGTTTTTGTTTTGTTTTTAACTCTACAAAAAA
AAGGGCCTTACAATCAATAATGACCACATCCATCCAAAAGACCACACTTTATGCCACTTTATAG
CATCTGAATAACTTTTCACAATCAAGGGATAATGTTGTTATGGCACTGTTGTAGAGAGCATGAT
AGCCTTGTCCAGAAAGTAAGCTGTGAAGCAAATATCTCTCTATATAAAATTATTTGTCCCAAAC
CATAGGAGCTACGAATCTGAAAGTAGTTACCGTATGTGATGAAGATATCCGCTAAGATGGTTCC
ACTGCAATGTGAAAGAATCGTGAAAAACGGCTGTAAAAAACCTTTTTTGATTACCAGCCAGATT
ACCAGATTTGGAAATTCCAAACCTGACTCGAAGTTAGGCAACTATCTTGTTTGAAGGACTGCTT
CCTAGTTCTTACGGTATGATACGGGCTGTGCATTGATCACCAATCAGTCAGTTAATTAGTCGCT
CAGTAATGGCAAACAGTTTTATATATAGATATATATATATATATATCTATTTGTTTATTTTTAG
AATGACAAGCACTACTGTAACTATGTTTTCAGTGTATTGTATCGCAAACTTACTTTAACTAACT
GGACAAGGATAACAAGGTATGATTCGTGTATGATTCGTGCATAAATTTATCAGCATCAACAGCC
GAGTTGATAAAGCAATGTCCGTTATTCCGTTTCTAGGCTATTGTCCTAAAACTTTGCATAGATC
CGTCTTTCTGTTGCAGGTAGTGCATATGTCGCAGCCGGATCGTAACCATATATCATATATCTGC
CATAGGAACGTGTCCAAAAAGTTATCTTAACTCGGCATTGGAAAATTGCACTAAACTCCCTACA
AAGCCTTATCTGCCTTGAATAACTATATCATATAGCTGCAAGAGGAGAGATCAGTGAAATACTT
AGTAGTATGTTATAGGGCAAAATACTCTGTTTTCATAGATATTTTAACAAACTTGGCATTTTCG
AGCTGCACTGCTCTCACATCTCTGCGAAAGGAATATGACAAGAGGGGTATTAATCTGGTTTGTT
ATAATTGATGTTGGTAAAAGGGGGTTCGAGAAATCCACTAGTCGGGAGATCGCCATCAGAGCAG
TCTTTTTGTTTTTATTTCGCAAATTTGTTTCGAGCCCTACTCAACTGTGCCCATTGGTCTGTTG
GGCGAGAGGTGGGCTGCAGCACGTATAGTTCCGGGCAAAACATTCAAAATACACTTTTAGCTGC
TGCAATAACGAAAAGATATACTTGTAATTCTTGTCCATTGTTCTGCATTTATGTTCTTTCATAA
TCTGAAAAGCCTATGTTGGTTTCGTGCGATTTTCCTCTTGCTGCAGCTCCGCCTGTCTTTATGA
TCGCAATTTCTTCGGCCAAGCGCAAAATATCTGAAGCTTTAGATCTGGATTGACCATGGTTTAA
ATAAAGACTACTTACAACTGGACCAAATATTTTACCTACGACCAGAGCGTGACAAATTGGTAAA
CTCGATGTGTACGTGCTTTTCAGCGTCTCTTCACGGTCGCATCAAACAACTTTTGGAAAAACAC
AAATTTCCGTCACGGGAGAAGGTTGTCACTGGCGAGCGCAAGTGGATTGAGTGAATACCAACGA
GATGAAGTTCACGTCAAATTTAGTTTCACAAAGCATTTAGAAACAGGGCTTCACACAGTGGGAG
AATCGGCCAACCTAGCTTATATCAATTTAAACTATGGAATCATGCATCTTAATGTTTTTTTTTT
AACTTTTTGATTAATATTACTTTATCTATCTAATGGATAATGTTTTTTATTTTGAATATCACTT
TTGATACATCTGTCAGCCACCAAAGCAACCTATGTTTTAGCTCGTGTAAAGACACATTCCGGCG
ATAATATACACTAAATACCGCACTATTTACAAATTATTGTCAAAAGTTTAAGCTATAAATCCAA
CAGGAGGTTCTAGTCGGGAGCTCCCGACTAGGGGATTCCCTGAACCCTCTTCTTCCAACATCAA
ATGCATATATGTATTCTATTTTAGAAGCTATATGTCAAGTTTGGTGTGTTATTTACCAAAAGTG
CCCAAAAAACAGGATATCGATATCGATTTCTATCGATTGCTTAGAAACGGAGTAAGTTATCGAT
TATCGGAAACAAACTCGATCTGCGCAGGCACTAGAAGCACCTACATCTAAAATTTCAAGAAATT
TCTAGCTCTTATAGGCTCTGAGATCCTTGCGTTCAAACATACGGACAGCCGGACAGACAGACAG
GCGGACATGGCTAGATCGACTCGGCTCTAGAATTTATATACTTTATGGGGTCGGAGATGCTTCC
TTCTGCCTGTTACATACATTTGGATTTTGCACAAATACCGTATACCCTTATAGCCATTTTTAAT

GGGTTTAGGGTATAAAAGTAACAAATAGGAGGCTTTGGTCGAGAGCTCCCGACTAGGGGATGCC
CTCAGCCCTCTTTTACCAACACAAAACACAGCTCGCACTACGAGCTCGTATGCTTTAGAAGTAA
ATAAGTCTTTCATTTTGTAGGTATCGCTGATTAATCGCTTAAGATCGATTAAGACTAGCCAGAG
ATTGATAGATAGATCAATAAGATCAACTGTTGAGAATTTTAATCAAGATCAGTTAAGAAATACA
GAAGTTATTAAAGTAAACGAATTGTTGGAATGGCTGTTGAAAAAAAGTGGCAGCAAGTTGTGCG
ATCTCATACGGGTTCAAGAGGGAGGGAACGTTTCTTTATTGAATAGTTTTTTATTACTGCCAAA
GGAAACGAGCTCGTGGAGTTATCTGCATTCGTTGTTTGTAGAAGATGGTTCAGGTTTTTCGCTA
GTCGAAAGGTCCCGACTTAGGTCCCTTACTTGTGTTTATTTGAGATAACCAAACCGGGATTTAC
TAGTTTCAGTTTGTTTCCTAAAAATATTAAAATCTGATTGGCCCACTCGGACCACTATATCATA
TAGCTGTTATAGGAACGATCGGTCGAAAATTAAATTCTTGTATGAAAACTATTTCGTTTATCAA
GATATCTTACCCAAACTTGGCATTTATAAGGTTCACTATGCTCCCGACCGATTTGCTAAATCCT
TTAAACAGCGGACCACTATATCATGTAGCGTCCACATTAAAGATAGGTCGAAATGTAAATTCTT
ATATGGCAAAATATTCATGATTCCAAGCGAATTTCATGAAACACAGACTTTACTATCATATTTC
TTTAGGTATCTGCAAAAGGGAAATGTTTACTTCAGAGGATTTTCCATATACCTGACCCTCTCTC
AGTTAGGGCTTAGACTCTTGATTCTGGGGAAATGAAAGCTGGGAAGCCATGAAAGAGGCACAAT
TAGCCCAGTTTGCCAAATTGAGAAATGGATTATTAAGATGATTGGCGGCATTCGAAAATTAGAT
TGGAGTTATTCTGCGGTTGCTTAAATGTTACATTTGGTAGTACTGGCATTTTAATCAAGTTTTT
CTGTTTGTGACGGCAATTAAAATTATTTATTAACACGCCGGAACAAATATTTGCTACACATCAG
CGTACTCACGAACACCCATTGTGACTTGGCCCCAACGTAACAGTTGCGTGAAGCATTACATAAG
AGTTTTTTCGCTACATAAACGCTAACTAATTGAAATATTAACACCTGTAAGCGATGGCCTTAAC
GAAGGTGACCCAAACGCAAAATATCACCGCAACTAGCGGCGGCAGCATAAAAATCCTGATAGCT
CATCTGGGCGGGTGTGTGCGTGTGAGCAATTAAACAAAGTGGGCATAAAAATTGGCTTGCCACG
ATATGAGTCCTAGAAAACATAACAAACGTAAATGTTGCGGCAGCCATAATACCAATAATAAAAC
GAAGAAGTATTAAGAACTAAGCAGAAGTGTTCCTGCGACTGCGGAAAAAATAATAAAGTTGGAA
AGTTAAAAGTAGGCTAAAAGGATTTAACGGTTTTGTGCAGATACAACAACAAATATATGTAAGC
ATGCAGAGGAAAGCATTCCATTCCTGATCATCATTAATAGCCGAGTCCATCTTGTTTCAAATAA
TCGATAGCATTTCGTGTTTCCAACATATCGTTAAATAAATCTTACCTTTTAAGAGCTAGAGCTT
CCAAAAATTACCTGTTGCTTTTCGTATGCCAAACAATGATTTTAGTTGTTTAATACATAATACA
AGATATTTCATCTTTTATAGAAGCTACATGCAACATTTGGTGGATCTAGCTCTAAAACCTAACA
AATTTGCTCAAAAACAGGACTACGAAATCGGTTTTAATCGAATGCTTGGAAATCGATAACTTTT
ATCGATTATCGATAACAAGGAGCATTAACATCCCAAATTTCAAGTCTCTAAGTCTTATAGGTTC
TGAGATCGTCGCGTTCATATATACGGACAAACAGACGGACGGACTGATCAAGAATATATATACT
TTGTGGGGTCGGAGAAGGAAGATGGGAGGAAGGAAGGAAGCATTCTGCCTTCTACATTTGCACA
AATACATTATATACCTTTTACCCTATTTTAATGGGTTTAGGGTATAAAAAGTTGCATTTTAATC
ATGTTTCTTGTGATTTTGTATTAGCTCGTAACTAAGAGACAAATTTTGTTATATGTTCAATGAA
TTGGAACAGATTTTCGGCTTAACAAAAATCGAATCACTTGCAATATGTCCAGCCTCTTTGCACT
ATTCTCTGCTAGCTCAAATGTAATTGTATTAGTTTCATTCAATATGTATTTAAGAGCAACGGCT
TTTCGTAGCTAAACGGCTTCGCGTTGCACTTACGTCCATGATTTGATGCTGGCATTTAAACGAG
AAACGATTAGTCAAACATTTCTCACTTGGCTTTTGTGCTCGGTCCGCGTTTGTGTTTTGTTGCT
TGTTGAGAGAATCGGTTAAGTTGCTTACACTAACAATGTTGCAAGCATTGTGCATATGCTTCCT
GTTGCTGTTATGCGCTCGAGTGCGTGTCCTTGTGTGATTCTGTGTGTGTGAGTGTGTGTCTTGT
ATATTTTAAAGGAATTAGAACAAATTACTGTAAATTAGCTTTACATTTGATTTTTTGAATTCGC
TGACATTTTGTTTACAGATATGATACACTTTCTTTTAGCCGCTCGTTAATCATTATTCGTAAAT
CAATAGGCATCCACGTAGTGTTATTGTTGGATTATATTCTGTTATTTTGGCATATAATTATGAA
TCACGAAAAAAAAAAATGTTTAATGTACAAAGTGCAGTCCGTGGAAACTGCTGAAAACACTTTA
CAAACTAACGTATAAATTCACACGCGACGTAAATTTAGCCACGATAAAACAAATGCGCACAGGG
TCATGGCGACCGCTTGTGTTGGCGTTTCAAACAAAACTGTTGCTTGGTTGAGTTACAGAACCGA
CTCGAGACAAGCGACTCATCGTGTGTTGGGATTCTCGCTGAAAGTCCTGTTCGTGCCAGTACTT
CGTGTTGAGCTTACGAGAGCTTATGGTATGCTCGTGTTATGCTAGCGGTTCATAACATGCTACC
GTTGGCTGTTTGCCAATTATTGCGACCATTGGCTATGCTTCCGGCTTCGGAAAGTTGCCAGCGC
CTGCTTCAATGTGACCGTTATGTTCTCTTAACACAGGCAAAACCGATGAAATTGAGTTCGCCTA
ACTTTTCCGCCTCTACCCATGCCAATATTCGCTCTTCAACTCGTGGTGAATTTTCGCTTAAGGC
CGGCTATCGTGCCAAACGGTTTGTTTACGTTTTGTGTTTACCTTCCAGTCGCGCAAAAAAACTA
GCTTCAGTTTAATAAACACGCTGGAAAGCCTCCGGGTGCCGGTAAAATCCCTTAAGAATTTATG
CCGGTAAAATCCCTTAAAAATTTATACCGGAATTGGGTTCGTACACTACTAGGCTTGTCATTCC
TAGAACCCCATAAAGTAGATTAAAAATAATCTTAAATAAAAATAATAATATATATACATATATA

TGTATAAATGTTTATATAATATCTATCTATCTGCTTCACAACGGCAAATTAATATTAGAATTTT
GATTGATATTTACATATTCCTGAAATATAAGAGAATATAAAATAAAATGTTGTATCTATAGTTC
TTTTATATGTATAGTACTATATACTTGAATGCTCGGACGCGACATTGTCCGCGTTATTTTCGTT
GAGCCGGTAAAATGTCCACTACTCCCCTGCCCCACCAATCGCGCGCATCCCCATCAATTATATA
ATTTAACAAAGTTGTGATAAAACACATACAAAGGCCCCTTTCCCCATTCCTTGTTCAAAAGTGT
ACATTTAGCGAAGAAATTACCCGACGTTGCCAGGGTCTACTCGAAAATGTTCCAAATTCCATTG
TACTTAAATTGCATCACCATGGGTCTATATAGTTTAGTGGTAGAATAGTTCATATATTTAACAA
TGAACCGTATATTACTTTTGTCCCAATATATACCATTTTACTTACTGTTAAAAAAACCCTTCAG
TCCTTAAAAGCCGGTGAAATAAATCTCGATTCTCACGAACATTGTAGATGTTGACTGTACTTAA
AGTGGCTTATAACTAATTCTCTTTGCTCAATTGTTGTTCGAGCACCTATTTTGGTTGTTTCCAA
GTCTAAACACTATAATGGACAAATCCTAATAAAATGCTTGTATTGCATATGCATATACAGAATT
AAACGTATTAAACGTTTTCACTTTCACCTTAATTGGAAACAAGAAATAGTGTCATTTAAATGTT
TACGTAAAGTTGCGTAAAAATCATATTGTATGCAACAAAGAACGAACGAAAATGAGTTTTCAAA
ACATAATTTTGTTTTCAAGCCGGAAGAAGATGCACACACACACACACGCACACACACACACAAA
CACACACTGCATAAACTGCGCAGCTTGCGACGTGCATTCATGTGTATGTATAGAATATAAAACA
CTTTTTAATACTGGAAATATCACAGGCGTAACTAATGAGGAGCCAATTGGGCCGTTTCGCGCTT
GGCTGGGTCATCTATGAAGCTGCTACCAGCGAGATACGTGCCAAGCCTGAGTCTGATATAAAAA
AAAACTATTGCTGCTATTAGCTTGTGTATTCGTGCTTGTACAAGTCGATGTGAGTGTGAGTGCG
AGTGTGAGTGTGAGTGCGAGTGTGGGTGTGAGTGTGAGTGTGAGCGTATTTTCCCGTGGGTATA
ACGTTATTTATGTATAGCAAGCTCCCTTGGCATCCATATACTTGATTTATAGCTGTTATTGAAA
AATTTCGGAGTGCAGCTTTTAATTATGAAGCGTGGAGCATTTTGGTGTGAAGTCCTGGCCTGGC
TTCGGTGTTGCCCTTTCGATGCTACGAAATGGATTAATGCATTCATTTACAGTTGTCTACAGTT
CCAAACAATTAACCATATCGTGAGCAGTATATGTAACTAAAGTTGCGGAGTAACACAGAAGCCA
TAATACAATAAGGATGTACTTTTCGTGGTTTAACTAAGTTAAATGCGTTATGATTCAGAACGTT
GTAGCCTATGGGTGACATATATTTTAATTTGACGGAAATTAAAGGAAAAACATCAAAAAAAAAA
AAAAAAAAAAAATATTGAAGCTACCAGAAACGCAGCGGAGGTGTAGCTGAGGCCCAGGCTGGCC
TGGCTGCCATAGAGGATGGCAATAGCGACAGTGGGCCGACAGTGGGACATGCAGGGCGCAAGTG
TTTTAACACAATTTAATATGTCCCGCCGACGATGACGACAACGTCACAGCATAGATTACTTACT
GCGACGACATGGGCCGGAGCCAGATAATATGGCTGGCTGAAAACGAGCCGAGAAGAGGCCGGCC
GCGATGACGATGCCGGAGTTGGCATCATTGATAATGTGGACGATTCCTATGCTCGCATGTATGA
GGCACGGCGGCGGCGCACACTTGCAACTTTATTAAATCATAAGGCACCAGATGACGCCGTCTGC
CGTTCACATTAGGGGCAGGCAAGGAATACGCTGTCTGGTGTCGAAGGAAACACAAATATAAGTA
TATGAATGGCAAAGTCAAGCTTGCCTTTAAAATTACTCTTCAGCAGCAACAAACGAAAACAAAT
AAGGGTGTAGCCGTCGGTTACTGCCGACTGCGTTTAGCGTATAATAGCAGCTCTTAAATATGCT
GCCCAAAATATTATACACGTATCATTGTAGTTAGTCAATCTTGGTGAAAACACGCAGTCCTAGC
CGTAAGAAATAAGAGTCTCAATCTGACTGGGGGAAAACATCAAACCCAACACTTAGATATAATT
GTGCAGACGGGATGGCTTTACTTGTTTAATTTGTTAATTTGTTATTTAATTTGATCAAACAAGT
AAAAAGGCTGCAGTCATCGATTGTGCTCGCTGCCAGATTCCTTGTACGCCGTCAGCAGCACGCT
TTGTGTTAATAGAATCATTATACTTATAAGACAACATTATTTTTATAAGATAAAAACAATATAA
CTTAACGAGAAGATGTTCTCTATTTAAAGTCAATTCGCAACTAATTGTACTTAAATCAAATCTA
ACGGCAGCGAAACATGTTTTTACTCGAGTGACACCGAAGAGTGATCCAATTTGTAGGCTTTGCC
AATTGCCGATTGCACTGTTAAAGCGAATTCAATTTAATTGTAAGCTCAATTTTAGTTGAGAGCT
GCGCACCAAATTAATTAAAATAATTTACCGGCTTGCATAAAAACAGCAAGCCCATCAACATTCT
ATGCACTTGTTGTTCTTGCTCTTCGTATAGTTGAATTTAATGGTACCGCAGAAAGATATGCTAA
CAATTTAATTAAAATTGCATTGAACAAGCATACACAAGAACAAACACTCTTGCACACTCTTACA
AACGCACCCACAATCATATTCCATGCTAGTCAGCCATTCAAACTATACTATATTATTCCAAGTT
ATTTCTTTCTACATCTTGCGGGATTGCGCCTATAGCAAAAATCAAGTACCCATTAAAAATGGGT
AAAAAGGGTATACTGTATTTGTGTAAAATTCAAATGTATGTAACAGGCATAAGGAAGCATCTCC
GACCCCATAAAGTATATATATTCTTGATCAGCATCAACAGTCGAGTCGAGAACAGTCCGAAGGA
AAGCATCTTCGTACCTATAAAGTATATACACATGTGTATCCTTGCTCAGCTTCAATACTCGAGC
CTATCTAGCCATTGCTGTCTATATTTGTGTCCGTCTGTGTGAACACGTCGATCTTAGAACCTAT
AACAGATTGGAGACAGACATTAGAATAACACGCACCTTGCATATGTAGGCTGCTTAAAAATCCA
GTAAGAGTGCTCTGCTTGTCAAAAATGATGACTCAAGGGAAGCTAGCTTCGGAATGGCCCCAT
TCGATCTGGTGTCCCTGAGGACAGGGTTCTTCTATCGGTGGATAAGGCTTTTTCGACCTTTTGT
CCAAGCGTTTCTTACGAGATCAAGCCTAGGTGTCGAGAATATCCAGCACTCTATGTTTGCGCTG
CAGCCGGGTCCATCGCGCAGGCTTTAGCTGGTTGGGCTGCGAAGTGTGAGGCACACTGACGGGT

TGCATGCCGCGTGCAGCTAGCCTTGGCTTAACCTATTTAAGAAGGAAGATGCTCTAGTCAGAGG
TTCCCGACCAGGAGATTCCCCTAACCCTCTTCTGTCAACATCAATTGCAGCTCGTGCTATGAGC
TCGTTTGCCTTAGCAGTTATATCAATGAAATATTAAAAAATAGTTCCCTTAATAAGACTTGAAC
CCATAACAGGCCTCGCTGCCAATAACAAATGTGCGTATAAAGCAACTGACTTCCTTTAGAAGTA
CCTGAAGCGCTTCGAAAACTGCGCTTGTAGGCGGAACATTGTATTAAGCATACGCCACGGTTAC
CCACACCCTGCGCAAAGACTCCTGACCCGTACTCTCTCTTCATGCAGAGCTCTCTGAGCATGTG
TGTGACAAATACCTAAGAGTCATTCCCGACAGACGAATAACATTTAAAAGGCATGTCACCAAGG
CAATCAACAACGTTAAATACCATATAAGGCGTAAGAGTTGGCTATCAATACCAACTCTCGTTCC
AATCTAAGGGTTTCATCCATAAGGTAATCCTGCCTCTGAGACAGTCGAACAAGCTAGAACCTAG
ACTAAACTCAACAAAATAAACATAGACTACCAAATATACCATATACAACCTGTATATATCTAAA
CGAAGCTACTTAAAGCCAGGCCTTTTAACTGGCCAGGCCACCCAAGATAATCCCTGCGACAGAG
ATCCTATGCACGACTAGGCCTATGGCGCAAGACTGCAAGACGAACAAATGGGCCTCTATAAGGC
AAGAATCCAACAGCGAAGAAGCCACAATTGAGTGAGTCCGTTGCATGCAGTCCTTTCGTGTCTC
GGTCTAGAGGCAGGGACCAGAAAACCCGAAGGGCGGTCCAACTCTGTGGCCCATCTGAGTTATT
TTAAAACGGAGAAAAAGATATTTATATAATTATGATTCGTTTTTTGCCTTTTTTCTAATTAGAA
TTTACTTTGCGAAGAAAGGGGAGGACACAAAGGTTATGGCTAATTCCAGGGAAACCTGAAAAGT
CTACACTAAGTATATTGATACATGCACGAACGTATTTGTTGGAGAGACAAACGCTCAATCTAAT
ATTTAGAAATCCAAAGCTGTTCCCAGCGGTAATTGGCATTAGCTTTCGGTAACGTCCTTTCATT
GTTGTTGTTCTGAAAAAAAAAAAAAATGATTTTAGAAATGAATATCAATTGCCATAACTATAAGA
TAATGCAAATAGTTATACGCACCTGTTGACGAGTTGAAGAGAAATGTCCTTACATGAGATTAAG
ATTGATGGCAAAGCAGAGATATCCTGCAAAGATTAATTAGAATTAATATGGATTGCTAATAACT
ATAAGAAATGCACTCAATTACATGCAAATACTGAAGGGATGATGGGGAAAGGGAGGCAAGCCGA
GTCGATGTAGCCATGTTTGTCTGTCTGTCCGTGTTCAACGAGAGGATATTTTTAGACATAAGCA
TTTTAAAAGGGTTTGGAAAGAAATCCTAATGGACTTCAACTGACCAAAGTGTAAAATAAATAGG
CAACAGAAAAATGAATAATAAAATTACCCTCCCAGTTCCCATTTTTCTTCTGCTGGCAGCACAA
TCGCGCAAAAATATTATAGTTCTTTGCACTTCAGTGAAAAGGAAAGGATTTCTAAACTAATATT
TGTATACCCTAAATTCATTGAATAGGCATAAAAAGTGTATAATGCCCGTCTGTCTGTCCAACCG
TTCCTTTGTCCGTCTATCGAATTTGACTACTGAACACTCTGATCTCAAAACCTTTAAGAGTTTG
AAATGATAAACATTGTTAAAAATCGATATCGAAATCCGGCTTGGTAGAAAGTTCAGGGTATCCA
CTCGTCGGGCAATCCCTTACTTGGCTTAAACTTCTTTAAATTTTGTCATGAAAGTCCATCTGTT
TGTCCATTCGTCCATTTGTCTATTCTAATGCAAACTAGAGTCAGCCTTAAAGCTGTCTACAAGA
AAGTTTGCATACATCCGCCTAGCTGTTTCAGGCAGCAAATATGTCGAAATCGACCGATTCGAAC
CACTATATCATGCTTGCCTTTATATCCATAAGTTTTTATGAGTTATCCTGTGTGCAAAAGTTCA
TACAAAGTCGGGCATTCTCTCTGCAAGGACAGGCAAATGCAAGCGCAGCAAAGTTCTGTTTGAT
TGCCTTGGCGTTTGTCAGGCGGCATTCATACTCTCAATACAAAAATTCATTCAATTCAATTGAA
AAATTGCGTTGAATATTTGACGTATGGCTTGGCAAGTAAGTGTGCTTGGCGGGGCAGATGTCAC
TGGCAGAGCGTCGGGCGTTGGGCGTCGGCATCAACGTCTACATCGTCGACGTTGGTAGCCAGCA
AAGCGTTAAAAATGACTGTCATAAATTAATGAGTGACGCCCGATTAAGTGACGCATTGAAATTG
AATATTGCTGAATTGCATTCATTATTCATTTGCGCTTCTCCTGTGAAGTTTCTTTGCCTATTCC
ACTTGTATTATCCTTAACCCTTCTATACGCCTCTGCCAGGTCAACGGCTACCTTAATTGCTCGT
ACAAGTTGCAATGCAGTGTAAGTAAAAACACAAGCGACACTATCAACAACGAAAGGAGTCCCTA
CCTGCTCAGCTTCCCTGAGTGCAGCATACTCCTATTACGAGATGGCAAGTCTCAATCTTGGGAT
AAACGTGACAATAATATAAATCGATAGATTACGCCGCCGTCGTCGGTTTGGTGTCGTTGTCTCA
TAGCCTTCGCCATCGTCGACATGAAGGTTGCCATTTAACTTAATGCCACATCTGGCAAAAGCGT
AAAACGTTTTAAATCTGATATTAGGAAACGCATCCTGCAATTGCACTGGCCACTGAAATTATGC
CCACAGCGCCTTTGCTACGCGAGCTGCTTAAACATTTGAACAGCTAATTATTCCGTAATGACAA
CAGCAAACCGCGATTGCGACTCAGCAATTTCTGTTGTACTGTTCAAACGGGTGATCCAACTATA
TAGTTCGACGTTTTGGTTAAGAGTGTATCTACTTTGCTGGATAAAATATAAATTCTTCTATATG
TAACGAATCACTGATATACTTGTGATACATTTTTTATTGTGACACAGCGCCCTGCTTTACGGAA
AATCAAATATATAACAAGTTCAATTCGAACCAGACAATAAGTTTAAGGCTGTTGTCTTTCGAAA
ACGAATCTTTATGCTTTGTGCTTTGGTTGTCCACATGTTTGCGGCAGCTAGTGCAAATTTCAAA
AATTTCTGAAAACATGTATACACACGCATTTATCCGCATCATTATTGAATTCTTTTAAAGCAGT
GCAATTGCGATTGTCTTGTTTTTTGTGTAGTGTGTATGGGATGCACCTTAAGTTGTTTTATTTT
TTGTGAGCTGGGCTGTTGGTTTCGCGGTTGAGTAGAGACGGCGGCAAAAAGCGAGGGCTATTGG
TAGAAGAGGGTTCAGGGTATCCCCTAGTCCTGAGCTCCCGACAGGAGCCTCTTATTTGTTTCAC
TTCTAACAGAGGTATGAATTGAATCATTTAGCGTCCCCAATAGTCAAGGTTAAGCTGTTATTAT

CTTCTTTACCTTCATTTTAAAGAATCCTTGAATTTATATTATAATAGAAATATATTGCCCTTTG
CTAACTAAAATAGTGCTTGAGTGTGGCTCACGATGGTCACGATCATTTCATGCTGCGTTTCCTG
CGTCGGAAAGGAACAATTATTATCCGACGTCCGTAGCGGCACTTCCTGCCCAGCGAAACTTTTA
TACAAACTCATAATTTTGAATTTTTGAGATAACAAATTGCAGCATGCAATTTGTGTCTATACGC
TTTCCTACGCTGCACGAATGCCTCCTTTACAGGGAGTTTTTTTTTCTGATTCTCGTTGCATGA
AAACTTTAAATTAGATTGTTCCGTAGACGTCTTGGCACTTTCTGAGTGAAAGCCGATTGTTATT
ATATATGACTGTTTTAGCGGCTTACTATCTTAAGTAACAAAAACAAGAGTGCTAAAAGCGGGAG
TGCTTGAAAAGAATATAATTTACCTATAATGCATTTAAACTTGCTTCTTAATCTACAAGTGTTT
ACTATTGTGGACTGGCTAGACAGGCTTGCAAGCAACATGCTCACTTATGGCTTGTTGATAAGCA
TTTTAATAAAAAGTTAACTTTTAAGAAAATATTTCTTAAAAATAAAATAAATCTTTTACCATCA
TTTTGCTGTCTTTTGGGTTACCAGTGCTGAACTGGGTCTGCAAGGGCATTAAATCGTCGACATG
CTGAAGGTATCACTACTTTCTCGTTCTTTTTTATTAATATATTTATTTATGATAGTTGTGATAG
TAAAAATTAACAAGAAGGCAAAAGAAAAACCAGTAGTAGGCACTAGTCGGGAGTTTCCGACTAG
GCGATACCCTGAACCCTCTGCTATCAGCACAAAATGCAGCAAACACAATACAATTGCAATCCTC
TTGATAGAATTGAATGAATTGTGGGTGAATGTTTGTGTGTGTACATAAATACAGCGCCTGCTCT
TTAACCGGGTGACACATACTCCAACCTCAAAAGCATCGGGTATTGCAGCAGCTTCGTCCGAAAT
GACGATGCCTGCACTCGACTACGCAACAGGGATAAGGTTGTGGAGCAGGAGACTGACTGTCCGC
CTCGTCTGTAGTTTAGAAAGCAATCGTGGAGTTTGCTTAACCACATGGGGGACACAGCTTTTGC
GTCCAGGCTAAGGATTGCCGGAGTATTAGCGTTACCCCGGACTTAAACCGTTTATTAAGTTCAC
GAACCGCACGAACAGCACTTTGTACTGATTTACACAGCTTAGTGTACTGCTTTCAAAACTTCTT
GTCAAGCAATGTACTGCTTTAAAATGCACTTTAGCACAAGCAGTTTTCTTATCCTTTACCGTAA
GCCTCGTTGCAGTTTTATGGCCTTGTCTACAAAGGGTATACAAATAAAGCTCCTCACAATCGGA
CTCGAGCCTGTACCGACCCTCACTGCTTGCCACCACCGCCAGTCAACAGTCCATTGTGTAACAA
ATAGACAGATAGAGCTTTGGGATCAAAAATGTCACAGTTTTGTAAAATTTGTAATTTTCCCGCG
TTTTGTTTTTTGCGCTATAATTTATCGAATTTCGAATCGATATTATGAAAAATGTGAAAGTATC
AATACTATAAATAGCTGATATATCAGCGTAGGCAAGCAGTAACTTTAAATTATAATTTTAAATT
TTAATAATCCCACACTTGCCACACTTGTGTGTGAAACTTAAAAATGTGTCATTTCTTAAGCAAA
GTGAGCAGAGCATATTACAATTTAAATACACAATTGAAAATTCGATGTTGTTAATGATTCATCA
TCGATAGTTAGCAGCCCTCACTCGCTCATCGCGAAGCGTTGACGATAGTACGAAACTATCGATA
GTACAAATCGTTGCTGCACGAGACTTGAAAATCGTGCTACCAGCGTGTTATCGGCAACTTTTAA
TTGCTTATATTTTATAAAATACACTAACCGCGGCCAGAAATTGAGTTTTAAGAAAAAACTGCGT
GGAATAGACATTATTCCTGACTATTTGGGCATTCCTTATGTGTGGTGGCTACGACGAATAATAA
ACGAAATCTACGTGAAGTGACCAACACGAAGAATTACCGTAAAGCATTTTTTTTTTTTAAATTT
AACTTTACCCATGCAACTCCTGGATCCCAACAAGATAGAATTCACAA~~TTTTTGTCATACAAAA~~
~~ATCAAATGCATTTTTACTTGGACATATATGTATGTGAATCGGCATCTGGCATCAGGAAAAGC~~
~~AAACAGTTGTGAATATAAAATTAAATGTACGAGATAGTCAACATGATTGTCTAGGTGGCCATAC~~
~~GATA~~TCATTTTTTCTTGGCATCCTTGAGACCAGCGCCGGCGTTGAATTTGAAGAAAATTATATC
GCCGTCTTCAACGGTATAGTTGCGTCCCTGTTGGCGATATTTGCCGGCCGCCTTAGCAGCTACC
TCACTGCCCTCCGCTTTAAAATCTTCGAAGTGCATCACTTCGGCCATAATGAAGCCCTTCTCAA
AATCAGTGTGTATACGTCCAGCGGCCTGTGGCGCCTTTGTGCCCTTTTGGACCGTCCAAGCCTT
GACCTCGTCGGGGCCGGCAGTAAAGAAATATTCCAGCTGCAAGGCCTTGTAACCAGTTATAATG
ATCTTGTCCAGCTGACTTTTGCATTTGGTCTCCTCTTCGTAGGCCTTGCGCTCTAAATCATCCT
TTTCACTTAGCTGCAGCTCGAATGCACCTGAGAAGGGTATAAGTAGCGCGCCCGGATCGTTCTT
GTCAATCCAATCCTTAATCTTGGGCAGCCATTTGTTCTTTTTGCGGATAAAATCTTTGTCGGAA
AGATTGACCAGATAAATGGCTGGCTTGGACGTCAAAAACAAATATTTGTTCAACGTTTCAATCT

***D. americana* SF12**

**Distal region**

>SF12_Contig4938:…6690..3403…( GJ17050[+](Ir7g) - *GJ16378*[-]
(*CG10932*))

CTGCAGCAGCGACGCGCTCACCTGGCCATTGGATACATACGCAAACGTGTGCAGCATGCCGCCA
ATCTGACAGCCGTCTTTCCGCACTATTCCAGCCGGCTAATCGGCTGCCTGTCGCTGAATGCGCA
CAACTTGACCAGCTTTGAGCTGTTGGGCTTTCCCTTCCAGACGCTCGCCTGGCTGGGCGTGTTG
ACCAGCTTCCTGGGCGTCAGCTGTCTCATGTTGTGCACGCGCCGACGTGTCGCCAGGGCGACAA
TGCTGCTCGCCGTGCTGGCGATTGCCTGGGGCCAGCCCATTGTCGCGTCCGGCCTGCGTCCCGC
CCAACAGCTGGTCTACATCAATTGGCTGGGCTTCACGCTGCTCGTGCGCGCCATGTACTCGGCT
CTGTTCTACCATATGCTGCGCCAGCAGGTGCATCAGCGTTTGCCCCGCAATCTGTTCGAGCTGA
TGGAGGGCAGCTATACGGCTGTGATGAATCGCATTACCGCCCAGGATGTGGGCGAGGTGAGCAG
CCTCCAGGTGCTATTGAGCAATATGCGTGCAATTGTCCTTGCGAGCGATGTGGAGCACGATGTC
CTGGAGCAGGTCGAGTCGCGGACAACGCAGCGGCGCCTCTTTGGCATACTCTCGCGCCAGACCC
TGCTGCATGCGGCACAGCGTGCCCACAAGCCGGGCGCCTACTATATGCTGCCGCAGCATGTGCT
CGAGCAGCAGCTGGCCATCTATCTGCAGAAGCATTCGCATCTGGTGCAGCGGCTCGATGAGCTC
ATCATGTCCATACAGGCGGTCGGCCTGATCAACTACTGGGCCGGCCAGCTGGGCAGCGAGCGCT
ATTTTCGCAGCACATTCATGTATCGGGACAATCGGCTGCGCCAGCCCGATCTCTGGGGTATCTA
TATCATTGTGGGCGTGCTCTACGCTCTCGCCACGCTCGTGTTCATCTGGGAGCTGCTCTCGGCG
CGCCGGCGTCAAGTCCAGTAG~~TCGCCGACGCTCAATTTAATTTAATGTCCAATTCACAAA~~GTTC
TCACGAGTGGAAATAAAAGCAAAAAAGAGAGAGGGCCACACTGTTTTTTTAAACGGCTTAATAT
CGATAACATCTGTTATCGTTGTTGGTATTAATCGTTTCTGGAAGTGCGATTGGGACGGCAAATA
AATGAACCTTGCTTGACGGAAAGAGGGAGATAACAAGCAGCTTTCAAATGTCGATAGCGATAAC
TTTTCAGCTTTCAATACGGTCAAAACGTATTCAACATTTTCGATAACGATAACAGCTATTTTAA
TCATTTGTTTTTATGCAATTCTTCTTTAAGTTTTAATGTTCGATAACGATAACGATAACAGGCG
TGATCGATTGAAAATTTCAATTAATGGAAACAGTTTAAAAGGAAACTAAGCGTATTCCAAATAT
TCGATAACGATTACAACTATCATCGATGACCATTTGTTTTTATGCAATTCTTCTTAAAGTTTAT
TTTGCAAATCACACAGTTTTCGT~~TTAAATGCTATACAATTATAATAGACAAAATTCATCTTTTC~~
~~AGAAACAAGTGAGAGTAAAAAACAGTTTTAATGTTCGATAACGATAACTGGCGTCATCGGTTGA~~
~~AAATTTGAATAAATGCAGTTCTGGCTTTAATTGTTGCTCTTGTTGTAGTTGTTAGTTTTCATGC~~
~~TGTATCCGCCGCC~~TTAGAGCTTCTCGAGCAGAATGGAGGAGGCGCCGCCGCCGCCATTGCATAT
GGAGGCGCAGCCCAGTTCGCCGGCTTTGAGGGCATGCGCCAGATGGGTGACCAGACGAGCACCA
GACATGCCAATCGGATGGCCCAGGGAGACGGCGCCGCCGTGAATATTCACCTTGGCCGGATCGA
TGTCCAGTTTGCGTATGTTGGCCAGCACGACCACGGAGAATGCCTCATTGATCTCCCACATGGC
GACATTCTCTTTGCGGACACCGGCACGCTCCAGCAGCTTGGGCACCGCCAATGCTGGCGCAATG
GGGAAATCAATGGGATCCGTCTCGGCATCCTGGAAAGCAACAATGCGCGCCAATGGCTTGAGGC
CAGCACATTTGGCCGCCTCGGCGGTCATCAGGACGACAGCGGCGCCGCCATCGTTCAGGGTCGA
GGCATTGCCGGCGGTAACGGTGCCATTCTCCTTTTGGAACACCGTCGCCAGCTGGCCGAATTTC
TCAAAATTGACGCGCTTGTATTCCTCATCCTCGGCAATGATCTGCTCCGGCTTGCGCTTAAGCT
GAATCTTCACCGGGGCAATTTCATTGGCGAAGATTTTATCGCTCCAGGCCTGGGCCGAACGCTT
GTAGGATTCAATGGCAAAGGCATCCTGCTCCTGGCGTGTCACCTGCATCTTCTTGGCGGTGTTC
TCGGCACAGTTGCCCATATGGAACTTGTTGTAGACATCCCAGAGGCCGTCGAAGACAATGCCAT
CGGTTAAATTGACACCGCCGTAGGGCGTGGCACCGCGCTTCAAATAGTATGGCACATTCGACAT
GGACTCCATGCCGCCGGCAACAACGATCTCAGCCTGGCCGAGCATCAGCGACTGGGCGCCGAGC
ATCACCGATTTCATGCCCGAGGAGCACACCTTGTTGACGGTGGTGCAGCACACACTGGTCGGCA
GGCCGGCAAAGATGGCTGCCTGGCGTGCCGGCGCCTGGCCAAGTCCGGCGGACACAACATTGCC
CATGATCACCTCGCTGACATCGCCCTTGCCAATGCCGGCACGCTGAATGGCCGCCTCGATGGCC
GTGGCGCCCAGCTGTGAGGCCGTTAGCGGCGCCAGCTGGCTCTGGAAGCTGCCGATCGGTGTCC
GTGCGGCGGACACAACAACAACATCGCTGATCTTTGAGCTGTAGCGACGCGCCTGGGCGCACAG
CTGGCTTCTGGTCGCTTGCAACAT~~GGCACTAGATTTATATAACCGACTGCCTGCTAGTATCGAA~~
~~AGATCACTGGAATTTTACAACCGGACCTGATCACACTTGCAATAATATTGAGGCTGAGAACCTG~~
~~TTGCTATCCACTTGCGTAACGAGCCGATGGCGTGTGAGCAGCTGTTGCGGACAGTGTTGGAAAG~~
~~TGGAAATTGGCAAGTGGCAAGTCGCTGCTTGCAAGCTTTTCGTGCTTTAGTATTTTTGCTTGTC~~

~~ATTGGGTTTTTAATGCTGCGTTGCTTTAATACGAAGAATACGACAGAATTGTTAATACTCTGTT~~
~~TTCAGTTGCCGGCTAGAAAAGATTCCGGTTTATCTTGGAACTCACATTGCCGTACTTTTTTTATT~~
~~AATGCCGTCTATGTTAATCATTGC~~

**Proximal region**

>SF12_Contig92: …25,867.. 50756…(*GJ18715*[-](*CG32698*) - *GJ18714*[-](*CG1354*))

CTAGCATCTTCGAAAGAAGTAGAAGGCATGTGTAACACAGCAACTGCAGCAATTCCAT~~ATTTCA~~
~~TCAGCTAGAAGAATGCGGTGA~~GTCTTATAACTTCAACAAGCCGTTTATTATTGTCTGAAACTTC
AGTGCGGATACTTATACATCAAAAGCGAAGAAATTATTGCGTTCAGAAATCTATAAAAATATGA
AATAGGAAGAAAAATAGTAATGTTTAGTGCTGAAAAAATAAATTAAAAAAACAAATAAAAGGGA
GTGCCCGACTAAGGGATACTCTGAACCCCTTCTACTAATGGAATATACCAGCATTAATAAGAAA
ATTTCTGCCGGTTACAAAATAGCAACTATATTCAACCGACTAAACTACTTGCTAATTGACTCTT
ATAACATGTAAGAGGCGCTAGTCGGGAGTTATCTTAGCAGAAATAAAAATAAATTAATAGAAAA
TATGACTCAATAGGACTCGAACTCGTAATATACCGCAAGAGAAAACCAGAGAGGAGACAATTTC
TGAGGTCACTTCTCTTTGGGAAGAGATATCTACAGAGGGTATTTAAAATGAAGCCTTCAAATAA
TTATTGAAACCAAAGATAGATATACATTTTGCTAACTTCTTTATTTTTGTACTTTAGTTAAGCT
TAAACATTTTTTTCTTAGTATAACATTTTAGTCAAAATACTTATGCCAACACATGAAATTACT
AATTAATTTATATCTTTTTACGGTTCTATGAAAATATATTTGTTTGGTTATTTTTTTTTTTAAG
ATTGTATTTGTTATATATAACAATGAGAAAATAAAGAAGAATTTTTACAAAGCCGTTTAAAAGT
TAAACTTACAAATGCACAATTTTTCTCACGTCAAAATACTTATGTCGACTAGTGTACACCAGGA
ATAGAATTTGAATGAAAACAAGTTAATTTACATGTCTACAGTAAATCTTGTGTTTTGCCTTTGC
TGTCACGTGGCTCTCCCTGGCCTAGGGCCTGTTATGGGCCACTAAAGTTCACATCGTCGCCATG
GAGGCCTTGCCAAAAACCTAGAAAAATACCTTAAACCCTCTATGCAGTTTGCAGGGCTTAACAC
CGAAGTCACAAGGATTAACCCAGTTGCGAGGCCTAATAAGTTGGTCATATTAGCACTATCACCC
TATGTCTTGTTTCGAAATCATGCTGAATAATTACTAGCATCGTCACGTACATGGAATAATTGAA
TTTTTAACATTTAGCCTGTTAATGATTCACACATTTACACATAAAAGCACATTTTGCAATATCA
ACATCAACGTTAACAGGCCTGCTGACATCGATTGACTCTATGAACTGTAAGGCACAGCCGGTGT
AAGAAACCATGCACGCGCAACCATATAGTAAAGCTCAGCCGATAATCATCCATGCAATGCCGGC
CATGTTGTGTTGAGCTTGCAATAGCGATCATCCAACATAGATACACACAAATATTATTTGTTTT
GACATGCGAGCTTTTTGATTTATGATGATTTTTGGTGAACACACAGAACTATTCAATTTCAAAT
ATTATTATGCCCTTGCACAGTTTTAAAGCTATCTCATGAAATATTGAATACGTCTGTTTTTCTG
GAACGCGCTGTACATATGTCGGAAGCGGCTGGATCGGACCATTATATCATATAGCTGCCATAGG
AACGATAGGTCGGAAATGTGGTTCTTGTATGAACATTTATTTTGTTTATCAAGATATTTTGACT
AAGCTCAAGCATTTTTAGTTTTACTATGCTTCTCATATATATGCAAAATCTTCTAAACATCGTA
ACCCTATAGCAAAAGTAAGTTTTTGCATGAATAACATTTTTGTTTTTCAATATATCTTGGTATT
TTTAATTCTATCATCTTCCGACCCATCATATATCATACCATATAAACAATCGACCGAAAATCAA
GTTCTTGACGAGCTTCACTATTCTTACATATCTGCAAAGCAGTTAGACATATTTAACAAGACAG
ACAAGACGGTTTGGTAGTGTGGGGTCTCGGACGAGAAAAGAGTTGATTGAGTTCAATTCATTTT
GGTTGTCAATTCTATTGAGCGCTGTAATGAAAAATCTAAATCCATGATCGGGAACTATTGATTG
CTGCGATAGTGGGTTGTTAGTGAAAGGCTTGTTTAAATTTCAGCTATGCTTATGATGCTTATTT
GAAATAAAAGTCAAGGAAAATGTTTAAGCATATAGTAGCTAAAGGCTTGAGTCGAAGCTGAAGC
TAGAGTCGACAGCACCTGGGATTCTATGACCCGAGTTCCGTAATCTGAGCGGGATGATGTCGAG
TGTAACGCTTGATTTCCTCTGACATAAGTGGCAGCGACTTTACGCAGCTTTGTTTTCCTGTGTC
GCTGTTGTCCTTATTGCGTCATCGTCGTCAGTGTTGCTGTCCTCTGGCTCCTTCAACAACGGGA
TTTGCAGTTTTATATTTTGCTGGCCTTTCCTGGTATACTCGGCATTGCCATTGTGTTGGACAAC
AGCTGTAGCAGTGGCATCAGTGGCGATGGCGAAGAGTGCATGTTAAGTGCCTTATTATCCTGTT
TATGTAACTGTGTAGTTTTTAAAGGTTATACGCGCCGACATATGTCTTGCCTGGCATTTTGTTG
CTGCATGTGTGCTTTATCAGCGCTAAAGTATTTTTCTCTTCATATTTAATTCATCTCAACTTAC
AAATTGTCATAATGGCCTTTTTCCTCCATGTACCAAAATACGTAATATTTTGAACACAATGTGT
TTACGTTTTCACGTTTGCCTAACTAACGAAGGCAATGATGCAATAGCCAAAATTGATGAATACA
GAGCCAGCTGTGACACAATGCAAATTTGAGCTGAAGCACGAAGTCCCATTTAGTTTTTTATACA
TATGAAGAACAAATATATTCTGTTGCATGAAAACTAGTTCTTAAGTGCTGAAGCTACAGTGCCA
AGCTCCTGAAAATCGTCTCAAATGCTTTAGCATACATTTTAAAATCCGAGTTTAAGTCGTTTGA

ATATGTAATAAAAATATCTTGTATACACACTACATTCACAAATTGGCCAATCCTTAGTTGCTAA
GAAGGGAAGTTTGCCAAGATAAAATTCAGAAGACTGTCTTCGGTGTTTGCTTTGTTTTATGACG
CTAGCTAAAAAACTGCGAAACAAGTTCGATTAAAGCTACAAATAAATATTATTTTCCGAAATAA
GGATCAAGGAATCAGGGCGCTAAAGTCGTTAGTGCCGAATAGGATTTGCATGAAATCGACGATC
TCAGTCCGCGCATCACGAGTTTTTCTTTAAACACCGATAACTTGCACCGTTTTCAAGCAATGGA
TAAAGATCGATATTGAAATTCTGTTTTTTGAGAAAATATTTTAGATTTTGAGAGCCAGAGTCTC
CAAATTTAAATGAAGCTACTAAAACTGTATGTACAGATCAAGTATATTTCATTTTATGACAACC
GTCGCACCTACGCCAACCACATCACGCAAAATTAAATAAAAAAAATCAATTGGAACTAAACATC
AATTAAAGACACAGTTTTATTTCTATTCATACGCATATGCTATAGCACTAAAAGATCTACTGCA
AGATATTTCATCAAGATCGGTTAAGAAACACAGAAGTTTTTAAAGGAAACGAGTCATTATATTG
GGAAGCCACGAATAAATGTGCTCTTCTTCATAAAAATGTCTGAATATAGTCTTATCAAGCTCCC
ACTTCTATTTTATATAGATTTATAGGCAGAATCTATGTATATAGAAGCATGAATATTATGGCAG
TTCGGAACTGGGTAATCGATGTCATTGCGACGGTTACTTTCGATTTGGGCCGTCTTTTCTTTTC
ATATAATGGTAATATAGACAATAAAATTAAAAGTAAACAATAGATAGTATTTAAACTGACCACT
TCATCACAATTTAATGGTTTAAGCAGCCGCAAAAATATTAAATGTATTTACCATTGCTTATAAT
ATTTATAAATTGTCTCCAAAAAGTATTTAATAATGAATATGCAAAGGTAAATGTGTGTTTTTCA
ACATTTTAATATGCATGGCAGGGCTGACATATGCAAAAAAGGCCGACAAAAAAGAAAAACCGCT
AACGGCAACGAAATAAATTTGTCAGCCATTGCAATATTCTTATTTTCTCTGACGACGAGCTGCA
ACGAACTGGACCTACCCCATCGGCTGGCTCTGGCCACGTAAATAAATAAATGCACAAATATACA
TATACACATATAAACATTATATATATTATATATATATATATATATATATATATATGAACGAACT
GAGACCGTGACTGAGGCAAAGGATGCAGGCGACCTTCCATTTCCATAGCCATTTCAAGCCCCTT
CCTGAAATTGTGCATGAAAAATATGGAAAGACATTTGGCCAGAGACAAATTTTTGCAGCTGCAT
CCGCATCGGGCTGATCCAAAAACTGACAATAAATGTCATTTAGTTTACCAACGTACAGATGCAA
ACAAACAATATTTATTTAAAAAAAAAAATTATAAAACCGATGGTCAATTGGAGTTTCTTGGTTT
TTGTTTTGTTTTCAACTTTACATAAAAAGAGCCATACAATCAATAATGACCACATCCATCCAAA
AGACCACACTTTATGTCACTTTATAGCATCTGAATAACTTTTCACAATCATGGGATAATGTTGT
TATGGCAATGTTTTTGAGAGCATGATAGCCTTGTCAAAAAAGTAAGCAGTGAAGCAAATATCTC
TCTATATAAAACTCTTTGTACTCACTGATTGGTGCGCGACGAACTGCCCAATCCATAAAAGCTA
CGAATCTGAATGTAGTTACCGCATTTGATGGAGAAACCCGCTAAGACGGTTCCACCGCAATGTG
AAAGAAGCCTGCAAAACGGCTGTATGCAACTTTTTTTATTACCAGCCGGCTTCAAAAACATTTC
CAGTTATGTTCAAATTCACCTGATGTGTGTTCCAAAATTTTTGGAAAATACACCCCTTTGAAGC
TATCGGAATTTTGGAAACTCCAAGCCTGGCTCGAAATTAGGCGGAAAATTTGCAACATTTTTGT
TTACCATCGGATCCTATTCAAACTAGTTTTCAAAGATCTTAGTGCAAATTTGTTTCATATGTTT
TTAGCAAAATCGACGGCGCATGGATGGGAAATGCGGGTCAAAGGTTTAACAATCGAAAGCTTAT
TTTTTCGGATTCAAGATTCTCACCACAAGACAAACATGCACGTAAATCGTTGATTCCAACTTTT
GGTGCTTTATTCAAAGGACTTTAAGCTAACAAATGTCTGTAATGTTTGTTCCGCCAATTTACCA
CTGTTCCGACCCCCATTTCCACAAAGAAGGGAAGTATTTTCCAAAAAGTGAAAAATGAATTTGA
ACAGAGATATTTGAAAATAGTTTGAGGCCGATCGGACGGTAAACAACAAAGTTTCATAAATCGT
TTTTGGCAGTTTTTCGCAACCCCGTCTTAATGTGCACCTTTATCACATAAGGTAACTAAAGTGA
ACATTTCAGCGTCCTAGCTCTTACGGTGTGATACGGGCTGTGCGTTGATCACCAATCAGTCAGT
CAATAAGTCAGTCAGTCATTCATATAGATATATGTATATATATATAACTATTTATTTATTTCAG
AACGACAAGCACTATTGTAACTATGTTATCTGTTTACTGTAGCGCAAACTTGCCCCTTGTGTCC
AGATGTAGTTAATTAGTTAAATACTCTATGTCTGTACATACAATACCAGAACTCAAATATCAAA
CTGACTTGTGCCTATACTAATATTTATCGGTATTATGTATATAAATATATCAACTTTACTAACT
GAACATGGATAAAAGGGTATCGTGATTTGTGCACAAATATATCAACATCAATAGCCGAGTTGAT
ATACCAGTGTCCGTCTTTCCGTTTCTGGGTTTTAAAGCTATTGTCTTAAAACTTTGCATAGGTC
TGTTTTTCTGTTGCAGGCAGTACATATGTCGGAGCCAGCCGGATCGTACTACTATGTCATAAGC
TTCCATAGGAACGTGCGTTCGAAAATGAAGTATGTACTTGTAAGAAAAACTGTTTTGTTTATCA
AGATATCTGAACTCGGCATTTAAAAATTGCACTATACTCCCTACACATTTGCAAAACCTTATCT
ACACTGAATACCTATATCATATACCTGCAAGAGGAAAGATCGTAAAAGCAAAATATTCTGTTTT
CAAAGAGATTTCAACAAACTCAGCATTTTCGAGCTGCACTGCTCTCACATGTCTGCGAAAGGAA
AATGATTAGAGGGTTATTAAACTTCGGCATGCCGAAGATATCGTTACTTTCTTGATTTTTGTTG
TATTTTATGTTGTGTAGTTCCGGGCAAAACATTCAAAATACACTATTAGCCGCTGCAATATCGA
AAATATATACTTGTAATTCTTGTGCATTGTTGTGCATTTATGCTCTTTCATAATCTAAAAAAGC
CTATATTGATTTCGTGTGATTTCCCTCTTGCTGCAGGTTCGTCTGTCATATGATCGCATTTCTT
TGGCCAATCGAAAAATATTAGAAGCTTTAGAACTAGATTGACCAAGTATTAAATAAAGACTACT

TACGATTGGCCCAAATATTTTGCCTACGACCAGAGCGTGACAAATTGGTACACTCGATGCGTAC
GTGCTTTTCAACGGTCTGCTTAAGCTACCGTGTCTACACATTCGCATCAAACAACTTTTGGAAA
AACACAAATTTCCGTCACGGTAGAAGGTTGTCACTGGCGAGCGCAAGTGGATGCCAAGTGAATG
CCAACGAGATGAAGTTCACGTCAAATTTGGTTTCACAAAGCATTTAGGAACAGGGCTAAACACA
GTGGGAGAATCGGCCAACCTAGCTTATATCAATTGAAACTACCAAATCTTGAATCAAAATGTTT
TCCTTTTTAATCTTTTTGATTTATATTACTTTATCTATCTAATGGATAACGTTTTTTAATTTGA
ATACCACTTTTAATACATCTGTGAGCCACCAAAGCAACATATGTTTTAGATCGTGTAAACACAC
ATTTCGCCGATAATATACACTAAGTATCGCACAATTTACAAATCATACAAATTACTTTGCCAAA
AGTTTAAGCTACGAACCTAAAAATAACAAATAGGAAGGCTCTGCCCGACTAGGGAATGCCCTCA
GCCCTCTTCTACCTACAAAAAATGCAGCTCGCGCTACGATGCGCGTACGCTTTAGCAGAAAATA
AGTCTTTCATTTTATAGGTATCGTCGACCCCCACCTACATTCCTTAGCTATAAATAAAATAAAT
AGATCGATTAAGACTACCCACCAATTTAAGCCAAAATATTAAAGCAATTGTGCTTTTGAGACTA
TACGAATATTCTATAGATCAATAAGATCAACTGTTGAGAATTTTGATCAAGATCGGTTAAGAAG
TCCATAAGTAAACGAATTGTTGGGATGGCTGTTAAATAGAAGTGGATGCAAGTTGTGCAATCTA
ATACGGATTCAAGGGGGAGGGAATGTTTCTATTTTGAATGTTTTTTTATTACTGCCAAAGCAAA
CGAGCTCGTGGGGTTATCTGCATTAGTTGTTTGTAGAAGATCGTTCAGGTTTTCCCCTAGTCGG
GAACTCCCGACTAGTGCCCATTACTTGTGTTTATTTGAGATAACCAAACCAGGATTTACTAGTT
TCAGTTTGTGCCCTAAAAATATGAAAATCGTATTAGCATCGGACAACTATAGCATATAGCTGTC
ATAGGAACGATCGGTCGAAAAGTAAGTTCTTGTATGAAAAACATTTCGTTTATCAAGACATCTT
ACCCAAACTTCCCATTTATAAGGTTTACTATGCTTCCGACCGATTTGCTAAATCCTTTTAACAT
CGGGCCACTATATCATATAGCTTCCATATGAAGGATAGGTCGAAACGTAAATTCTTATATGACA
AAATATTCTGTGTTCCAAGATAGTTTCATCAAACACAGACTTTTCTATCATATTTGTTCACGTA
TCTGCAAAAGGGGAATGTCTACAAGGTAATAACATCTTCGGCACGCCGAAGATATCCTTACTTT
GTCGTTTTTGACTATTTTCACAGACAGAGCTCAAATACAATAAGTGTCGAGAACTAAGCGACTT
TCCCATTCTAACAAAAGAAAGTGATTATTACAGGATTTCCCAAAAGATCAAACCTCAGAGAATT
TTTCATATACCTGACCCTTTCTCAGTTAGGACTCAGACTCTTGATACTAGGGAAATGAGAGCTG
GGAAGCCGTGAAAGAGGCACAATTAGCCCAGTTTGCCACAGTGACATTGGCTCAACCAATTGAT
AAATGGATTATTAGGATGATTGGTGGCGTTCGAAAATAAAAATGTACTTATTATGCGCTTGCTA
TGTGGTTGCTTAAATGTTACATTTGGTAGTTCTGGCATTTTAATGAAACCTTTTCTATTTATGA
CGGCAATTAAAATTATTTATTAACACACCGGAACAAATATTTTCTACACACCACACATCACGAA
CACCCATTGTGACTTGGCCCCAATGTAACAGTTGCGTGAAGCATTACATAAGAGTTTTTTCGCT
ACATAAACGCTAACTAATTGAAATATTAACACCTGTAAGCGATGGCTTTAACGAAGGTGACCCA
AACGCAAAATATCACCGCAACTAGCGGCGGCAGCATAAAAATCCTGATAGCTCATCTGGGCGTG
TGTGTGCGTGTGGGCAATTAAACAAAGTGGGCATAAAAATTGGCTTACCACGATATGAGACCTA
GAAAACATAACAAACGTAAATTTTGCGGCAACCATACGCCAGACCGGGAACAGTCATAATAACA
ATAATAAAACGAAGCAGTATTAAGAACGAGGAGGTGTTCCTGCGACAGTGTTCCTCACAAAAAA
ATTATAAAGTTTAAAAGTTAAAACTGGGTTAAAAGCGTATAACGGTTTTGTGCAGATACAACAA
CAAATATAAGTAAGAATGCAAAAGAAAGCATCCCATAAAGCATATATATTCCTGATCAGCATCA
ATAGCGGACTCCATATAGCCACGACCGCCTGCCCTGTTTCAAATAATCGATATCACTACGTGTT
TCCAACATATCGTTAGGTATATCTTACCTTTTAAGAGCTAGAGCTACCAAAAATTACCTGTTGC
TTTTCGTATGCTATACACATATAACCATGATTTCAGTTTTTTAATACATAATACAAGATATTTC
ATCTGTATATACTATTATAGAAGCTATATGTCACATTTGCGTGGATCTAGCTTAAAAACCTAAG
AAATTTGCTCAAAAACAGGATTACGATATCGGTTTTTTTCGAATGCATGCAAATCGATCACTTT
TATCGATTATCGGAAACAAGGAGCACCTACATCTCAAATTTCAAGTCTGTAGTTCTTATAGATT
CTGAGATCGTCGCGTTCATATATACGGACGAACAGACGGACATGACTAGATCGACTCGGCTATT
GATGCTGATCAGGAATATATATACTTTGTGTTATCGGTGATGCTACCTTTCGCCTGTTACATAC
ATTTGCACAAATACATTATACCCCTTTTACCCGTTTTCAATGGGTTCAGGACAACAAATATATT
TATACAAGTTTAATAAATCTTCAAGCATACCACAGATTTGCCAAATTTATGGTTACATAAATGA
AATCACGCCGAAAGACGGCTTATGTAAAGCAAGTAACAAAACAAGTCTCTTACAACATTAACCA
TATAAATTTATCATTTTCGTTATTAGAAGGTCCCAAAAATGTGAGTTGGCAGCATTACTGCAAA
CAAGAGGCAGGACACGAGACTTAATAGACCAGGGTACATTTTTTACATAATAAACCAATTTAAA
ACCGGCAAAGGGTATAAAACGAAAATATGGCACTCTTTGAATAGTCTACAGAACTTATGTATTT
AATGAAGCAATTTTATTAAATTATTATATAAGTATTGTGGATCGTCTAGCTTGCAATAAGTTCA
TTTGTCTCTTTGAACCATCATTTGTTCGATCATTGAATATATGCGCGTCTATAATCGCATTTGA
CGAAATTCTAAAAACTATTGCAGCATCGCAGACTTAGAAATCGCCCTTAAAAAAACAGTTTGAG
TGCTACCGCTGAAAGGCAACTGATAAAGCTACCAACCAAGATGGTCAAGATGCTATTTTCCACC

AGTCTTGATCTGCTATTGCAGCGAATAGTCTGTAATATATGTAGTCTGTTACATACCTAACAAG
TATGAGGTTCTAGTCGGGAACTCCCGACTAGGGAATACCCTGAACTCTCTTATTCCAACATCAA
ACGCAGTTCTCGCAACGCGCTCGTTTGCAGTAATAAAGATGAATTTAAAAAAAATGTCGATATC
GAATTTTATCGATTGCTTGCAACCGAGTAAGTTATCGTTTATCGGAAACAAACTCGATCTGCGC
AGGCACTAGGAGGACCTAACACTAAAATTTCGGACGGACGGACAGAGAGACGGACATGGCTAGA
TCGACTCGGCTTGTGATGCTGATCAAGAATATATATACTTTATAGGGTCGGAGTTGCTTCCTTC
TGCCTATTACATACATTTGCACAAATACATTACACACTTTTTGCCCATTTTTAATGGGTTCAGG
GTATAAATTCAATCATGTTTTTTGCGATTTTGTATTAATTTGTAACTAACACACAAACTTTGTT
ATATGTTCAACGAAATAGAAGAGCCGAAATAGCAAAATCGAATCACTTGCGATATGTCCAGCCT
CAGTGCACTTTTCTTTGCTAACTCAAATGTAAATGTATTAGTTTCATTCAATATGTATTCAAGA
GCAACGGTTTTTTGTAGCTAAACGGCTTTGCGTTGCACTTACGTCCATGGTTTGATGCTGGCAT
TTAAACGAGAAACGATTAGTCAAACATTTCATTTCTCACTTGGCTTTTGTGCTCGGACCGCGTT
TGTGTTTTGTTACTTGTTGAGAGAATCGTTAAAATCGCTTACACTAACAATGTTGCAAGCCTTG
TGCATATGCTTCCTGTTGCTGTTATGCGCTCGAATGCGTGTCCTTGTGTGTATGTGTGTGTG
TGTCTTGTATATTTTAAGGAATGAGAACAAATTACTGTAAATTAGCTTTACATTTGATTTTTTG
AATTCTCTGACATTTTGTTTAAAGATATGATACACGTGCTTTTAGCCTCTCGTCGTTGCCATTT
CATTGTCGAAATAACATACCGCATATCAATTATAACATTACCGATTTGAACCTAATTCTGTGTA
CATGGTTTATGAGGTTATTCTGAGTTCAATAGGCATCCAAGCAATGTTATTGTTGGAATTTATT
TTGTTTTTTTGGCATAGAATTTTGAATCACGAAAAAAAAATGTTTGATGTATAAAGTGCAGTCC
GTGGAAACTGCTGAAAACACTTTACAAACTAACGTAGAAATTCACACGCGACGTAAATTAAGCC
ACGACAAAACAAATGCGCACAGGGTCATGGCGACCGCTTGTGTTGACGTTTCAAACAAAACTGT
TGCTTGGTTGAGTTACAGAACGGACTCGAGGCAACCGACTCGTGAGTTGGAATTCTCGCTAAAA
GTCCTCGTCGTGCCACAACTTCGTGTTGAGCTTACGAGAGCTTATGGTATGCTCGTGTTATGCT
AGTGGTTCATAACATGCTACCGTTGGCTGTTTGCCAATTATTGCGACCATTGGCTATGCTTCCG
GCTTCGGAAAGTTGCCAGCGCCTGCTTCTATGTAACCGTTATGTTCTCTTAGCACAGGCAAAAC
GGATGAAATTGAGTTCGCCCAACTTTTTCGCATCTACCCACGCCAATATTCGCTCTTCAACTCG
TGGTGAATTTTCACTTAAGGCCGGCCAACGTGGCAAACGGTTTGTTTACGTTTTGTGTTTACCT
TCCAGTCGCGCAAAAAACTAGCTTTAATTTAATAAACACGTTGGAAAGCCACTTGGTGCCGGTA
AAATCTCCTAGAAATTGATACTGAATTTGGTTTCGTACTCAACTAGGCTTGTCATTCCTAGAAA
CCTATAAAATAGATAGAAAATAATCTTATATATTTTTATACCCTGAACCCATTAAAAATGGGTA
TAGGGGTATATTGTATTTGTGCAAAATCCAAATGTATGTAACAGGCAGAAGGAAGCTTCTCCGA
CCCCATAAGGTATATATATTCTTGATCAGCATCAATAGCCGAGTCGATCTAGCCATGTCCGTCT
GTCTGTTCGTCCGTCCGTCGGTTCGCCCGTCCATATGTATGAACGCAAGGATCTCAGAACCTAT
AAGAGCTAGAGACTTGAAATGATGTAGATGTAGATGCTCCAAGCTCCCGCGCAGATCGAGTTTG
TTTCCGATAATCGATACTTACTCCGTTTCCAAGCAATCGATAAAAATCGATATCGATATCCTGT
TTTTTGGGCAATTTTGGTACATAATAAGAGCTAGGGTCACCAAACTTGACATATAGCTTCTAAA
ATAGAATATATGTATATATGCATTTGATGTTGGAAGAAGAGGGTTCAGGGTATCCCCTAGTCGG
GAGCTCCCGACTAGAACCTCTTACTTGTTATATATGTAAAAGGTTTTATTTAATTTCTAATATT
ATGTTTGGTGTTAAAACTGCTTCACAAAGGCAAATAAATGTTAGAATTTTGATTCTTATTTACA
TACATAAATACTCAGAATATGAATTCCTAAAGTATTAGAGAATAAAAAATACAAAATTCTTCAC
TCCGGAGAAAAATGTTGTATCATCTCTTATATGTATAGTATTATATATATGCATGCTCCGACGC
GACATTGTCCGCGTTATTTTCGGTAAGCCGGTAAAATGTCCACAACTCCCCTGCCCCACAAATC
GCGCGCATCCCTATCCATTATTATATATGTACGTTGTACCAAATATATATACACTTATATATAA
GTATACATTTAGAGAAGAAATTACCCGACGTTGCCAGGGACAGCTCAATAATGTTGATTATCCC
ATTGTGATGAAATTGCATATTGGTAACCATGCGTCTATATAGTTCAGCGGTGGAATAGTTCATA
TTTATTAATGGGCTGTATATTACTTTGTCCGAATATATGTATGTATACCATTTTACTTACAGTT
AAAAAAAAGCAACTCTTAAAAGTCGGTGAAATTAATCCCGATTCTCACGAACGAAACGTTCGAT
TAGTTTTTGCATTGTAGAGACATTGATGTTGACTGTTCTTAAAGTGGCTTATAACTAATTCTCT
TGGCTCAATTGTTGTTCGAGGACCCATTTTGAATATTCCCCCGTCTAGACACTTTAAGTGGCAA
ATTCAGAATAATATGCTTGTATTGTATATACAGATTAAGAGTCTTTAACGTTCTCACTTTCACC
TTAATTGAAAACAAAAATCTTGTCTAAGGTAGTTCAATGCGATTTGTTGCATTTACTTGTTTTC
GTAAAGTTTCGTAAAGATCATATTCTATGCAACAAAGTTGAACGAAAATGAATTTTCAAAACAT
AATTTTGTTTTAAAGGAGCAAGAAGAAGCACACACACTGCATAAATTGCACAGCTTGCGACGTG
CATTCATGTGTATGTATAGAATATAAAACACTTTTTAATACTGGAAATATCACAGGCGTAACTA
ATGAGGAGCCAATTGGGCCGTTTCGCGCTTGGCTGGGTCATCTATGAAGCTGCTGCCAGCGAGA
TACGTGCCAAACCTGAGTCTGCTATATAAAAAAAAAAAAAAAAAAAAAACTATTGCTGCTATTAGC

TTGTGTGTTCGTGCTTGTACAAGTCGATGTGAGTGTGAGTGTAAGTGTGAGTGTATCTTCCCGT
GGGTATAACGTTATTTATGTATAGCAAGCTCCCTTGGCATCCATATGCTTGATTTATAGCTGTT
ATTGAAAAATTTCGGAGTGCAGCTTTTAATTATGAAGCGTGGAGCATTTTGGTGTGAAGTCCTG
GCCTGGCTTCTGTGTTGCCTCTCGGTACAACTGCTGCTGCTGCGGCTGGAAACAAAAACTGTGT
TTCCAAATGGATTAATGCATTTATTTACAGTTGTCTACCGTTCCAAACAATTGGCCATATTGTG
AGCCGTATATGTAACTAAAGCTGCGGTCGAAAATATGTCACTTCGGAGAAACACACAAGCCATA
ATACAATAAGGATGTATTTTTCGTGGTTTAAATAAGTTAAATGCGCTATGATTCAAAACGTTGT
AGGCTATGGGTGACATATGTTTTAATTTGACGGAAATTAAAGGAAAAACACCAAAAAAATTGAA
GCTGCCAGAAATACAGCGGAGGTGTAGCTGCGGCCCAGGCTGGGCTGGCTGCCATAGAGGATGG
AAATAACGACACTGAGCCGGTGGGACATGCAGGGCGCAAGTGTTTTAACACAATTTAATATGTC
CCGCCGACGATGACGACAACGTCACAGCATAGATTACTTTCTGCGACAACATGGGCCGGAGCCA
GATAATATGGCTGGCTGAAAACGAGCCGAGAAGAGGCCAGCAGCGCTCACGATGGCAGAGTTGG
TATCATTGATAATGTGGACGATTCCTCTTCTCTCGTATGTATGCCGCACATTGAGGCGGCGCAC
ACTTGCAACTTTATTAAATCATAAGGCACCAGATGACGCCGTCTGCCGTTCACATTAGGGGCGG
ACAAGGAATACGCTGTCTGGTGTCGAAGAAAACACAAATATATATGAATGGGAAAGTCACGCTT
GCCTTTAAAATTATCCTTCAGCAGCAACTAACGAAAACAAGTCGGTTACTGCCGACTGCGCTTA
GAGTATAATAGCAGCAGTTAAATATGCTAGTTAGTCAATGGTTTACTGGCAAGATTTGGTGAAT
CTAGTTTTAAAACTGTCGGAACAGTCCCAACCGTAAGAGATAACAGTCTGAAATCCTTACTGAC
AAAATTCGATCCAGAAATGGGGGAAAACATCAAACACAGCACAGCCATTTAATTGTGCAGACGG
GACGGCTTGAGCAAGGACAATGGCAGTAAATATATTGTGTTTGGTATGAGCCCGTGATTTGCGT
AGGGCTGACAATCGAGCAGTCGCCGGTTACAATTCAGGAGCGAATTTTCGAATTTTAATATATG
AATGTTTAATTTGTTATTTAATTTGATCAAACAGGTAAGAAGGCTGAAGTCATCGAATGTGCTC
GCTACCAGATCCCTTGTACCCCAGAGTCAGTTGGTTTTTAGCAGCACGCTTTTTAGAAGTAAGA
GGTTCTAGTCGGGAGCTCCCGACTAAGGGATACCCTTTATCCTCGGTCGGAGAAGCTTCCTTCT
GCCTGTTACATACATTTGGTTTTTGCACAAATACAATATACCCTTATACCCATTTTTAATGGGT
TCAGGGTATTAAAAACAATATAATTTTACGAGATGTTCTTTACTCAAAGTCAATTCGCAACTAA
TTGGATTTAAATCAAATCTGTGCCTAGTAAACTGTGGCCTTGCGGCAGCGGCGCATGTTCTGTC
TGTCGGTCAGAAACTCCCAACAGTGTTTTTACTCGAGCTAAAATGCCTGACACCGAAGAGTGGT
GCAATTTTTAGCCTTTGCCTTTTGCCGATTTCACAATTAAAGCGAATTCAATTTAATTGTAACT
TCAACTTTAGTTGAGAGCTGCGCACCAAATTAATTAAAATAATTTACGGGCTTGCATAAAAACA
GTAAGCCCATCAACATTCTATGCACTTGTTGTTCTTGCTCTTCGTATAGTTTAATTTAATGGTA
CCACAGAAAGATATGCTAACAATTTAATTAAAATTGCACTAAATAAGCATATACAAGAACAAAC
ACTCTTACACAGACTTACAAACGCACCCACAATCATATTCCTTGCTAGTCAGCCATTCAAACTA
TACTTTATTATTGAAAATTATTTCTTCCAACATCTCAGGCTTGCGCCTATAGCAAAAATTTGCA
AAAGGCGCAACAGCTGGCTGTGGACTTAACATTCAGATATTCTAAAAGTAAAATCAAACATTTT
GGGGATTTTTTTATTTATTCGTTTTTCCTCTGTTCACAAAGTTGGAATGCGTTTTATTATACCC
TGATCCATTGGCTAAATAGAGTATAATGCTTTTGTGCAATGTATTTAACAGTCCGAAGGAAAGC
GTCTCCGTCCCTATATACACATTTATATCCTTGCTCAGCATCAATACTCGAGCCTATCTAGCCT
TGGCCGTCTATATTTGTGTCCGTCTGTATGGAAACGTCGATCTTAAAACCTATAACGGATTAGA
CTTAACATACAATTTTGAATATAGAGGTCTCCCTCTATAGCACCCGCTGTACGAGTTTGTTCAA
ATCATCGACTAGACATTAGTATAACCAGACATTAATATAACACACACCTTGATTATACATATAA
GTGGGCTGGTTAAAAAACAAGTAAGAATGCTCTAGTTGGAAATGCTCCACTAGCAAATAACTTT
AAGCTTCTACCAAAAAGATACATAATTGATCTATATAAAATTTACGAAAAGCTACTTGTCAAAT
ATTATGACTCTAGGGAAGCTAGCTGCAGAATGCGCCACATTCGCGCTAAGACCTGTCCCTGACG
ACAGGGTCAGTGCATTACACAGTCTACACAGCGGATAAGGCTTTTTCGATATCGAGCCTGGGTC
GAGAAGATCCAGCACTCTATGTTTGCCTTGGCTTAACCTATTAAAGAAGGAAGATGCTCTAGTC
AGGGGTTCCCGACCTGGAGATTCCCCTAACCCTCTTCTGTCAACATCAACTGTCAACATCAATA
AAAAATAGTTCCCTTAATAAGACTTGAACCCATCAAAGACCTCGCTGCCAATAAAAAGTGTGTG
TATAAAGCAACTGACATCCAATTGGAGCACCTCAAGCGCTTCGAAAACTGCGCTTGTAGACGGA
ACATACGCTACGGTTACCCACAACCTGCACAAAGACTCCTGACCTGTCTCTCTTTTCATGGAGA
GCTCTCTGAGCATGTGTGTGACAAATACCTAGGAGTCATTCCCGACAGACGACTTACATTTAAA
AGGCATGTCTCCAAGCCAATCAACAACTTCAAATGCAATATCAGACGTGTGAGCTGGCTTATCA
ATACCAACACCAAACTCTCTCTAAGGGTCTCATCTATAAGGTAATCCTTGCCCCTGAGACAGTC
AAACAAACTAGAACCTAGTCTAAACTAAACAAACTGCACATAAACGACCATATATACCATCTAC
TACCTGTATATACCTGAACGAAGCTACTTCAAACCAAAAGTATTGTTTAACCGTAAACCCCCCG
ATATCTCTATATATCAAGATGTTTGTCCTGACTGACTGACCTGACCGTAAGAACCAGGAAGCTG

AAATTTTCACTGTAGTTACCTTTTGTAAAAAAGGTGCACATGAAGACAACATAGCTAAAAATCA
AATAAATAAACCAATTGAAACTAACCACCACTTTAAGCTAAGTTTTACTAGCGGGTATAACCCG
GCTTTGCCCGTGGCGCGTATAATATAAATTTTCTCAAAAAGCATTATAAATCTTTTTGACACTG
ATCGAACGGTAAACAAACGTTTTCCACCAATTTACCACTGTTGTGGATGGAATTTCCCAAGTCC
CTGAAACACGTATACATTAAGTTCTTATGTATGGCTTTGAAAATTACTCAAAAAACTTAAAACT
CCATCACAAAATCTTTGAATCGCGCTTTCCACCAAGGAATCTTGAATTCTGCAAAAAGGGTGAA
ACATGTCTCAAATGATTTGTTACATTTGACATTATCCGCAGCATCAATATATTGCTCAGTGGGC
TGCGTCCCATATTATCGGCCCCGTCGATATCTCTTCGTTGCACGGTCAGGGTATCGGGGGTGAA
GTCGAACGCGCTGTGGCTCCCAATTATCTGTAGGGTGGCAAAGCCCGGCTTGCGATCCACTGCG
CTGAGAGGGGAAACTGTTGAGATTGGCCGCCCGATTCGCTCATAGAGAGATCAGGAGGAGAGCG
GATCGGGTGCCCTTGGCAATGCCCTTGGCCTTCATCTGGGCCGTTGACCCAAAATGCAGCTCTG
CATTTGGGCAGCAAATGTCACCACCTTGTCTAGGTGCAGCGAGGAGTTCGACTTTGTCAGCAGT
CAACGGCGGGCAACCCGTGCTCATACCAGAGACCAAAATTTTTACACATTTTCCACACATAATA
ATTACACTAAGATTTTCCGTGCCACTAACGACCTTTAATAAAATAAAATAATTTAATAACGCCG
CGCAGGGAAAGGAAAGAACATATAGAGACAAGTAAGAGGCTCTAGTCGAGAGCTCCCGACTAGG
GCATACCCTGAACCGTCTTCTTCCAACATTAAATGCATATATATATTCTATTTTAGAAGCTATA
TGTCAAGTTTGATGACAGTGACGCCCTGATCGCAAAAGTTCGAAATGTTGAACATAGTTAAAAA
TCGATATCGATATTCGGTTTGGTAGAAGGTTCAGGGTATCCACTAGTCGGGCACTCCTTACTAA
AGGACCGTTACTTGGCTTAAAATTCTTTAAATTTTGTCATGAAAAGTGTTACAATAAAGCTTAT
GAATTCTTGATCAGTATCAAAAGCCGAAACGATATAGCCAAGTCCGTCTGCTTGTCCGTCCATT
TGTCTATTATGCAAACTAGTGTCAGCCTTAAAGCTGTCTACATGAAAGTTTGCATACATACGCC
TATCTGTTTCAGGCAGTAAATATGTCGAAATCGACCGATCCCAACCACTATATCATACATGCCT
TTATATCCATAAGTTTTTATGAATATCTTGTGTGCAAAAGTTCATACAAAGTTGGGCATTCTCT
CTGTAAGGACATGGAAATGCAAGCGCAGCAAGGTTCTGTTTGATTGCCTCGGCGTTTGTCAGGC
GGCATTCATACTCTCAATACAAAAATTCATTCAATTCAATTGAAAAATTGCGTTGAATATTTGA
CGTATGGCATGGCAACTAAGTGTGCTTGGCGGGGCAGATGTCACTGGCAGAGTGTCGGGCGTTG
GGCGTCGGCATCAACGTCTACATCGTTGACGTTGGTAGCCAGCAAAGCGTTAAAAATGACTGTC
ATAAATTAATGAGTGACGCCCGATTAAGTGACGCATTGAAATTGAATATTGCTGAATTGCATTC
ATTATTCATTTACGCTTCTCTTGTGAAGTTTCTTTGCCTGTTCCACTTGTATTATCTTGACCCC
TTCTACTCGCCTCTGACAGGTCAACGGCTACCTTAATTGCTCGCACAAGTTGCAATGCACTGTA
AAAACACAAGCGACACTATCAACAACGAATGCTTCTACCTGAAACCCTACCTGATCAGCTTCCC
TGAGCCTCCCCAGCCTCCTCCTATTACGAAATGGCAAGTCTCAATCTTGGGCTAAACGTGACAA
TAATATAAATCGATAGATTACGCCGCCGTCGTCGATGTCGGTGTCTCATAGCCTTTGCCATCGT
CGACATGAAGGTTGCCATTTAACTTAATGCCACATCTGGCAAAAGCGTAAAACGTTTTAAATCT
GATATTAGGAAACGCATCCTGCAATTGCACTGGCCACTGAAATTATGCCCACAGCGCCTTTGCT
ACGCGCGCTGCTTAAACATTTAAACAGCTAATTATTCCGTGATAGCAACAGCAAAACGCTATTG
CGAGTCAGCAGTTCTTTATTGAACTGTTGATCCAACTATATAGTTCGACGTTTTGGTTGAGAGT
GCATTTACTTTGCTGGATAACATATGAATTATTCTGTATGTAACAAATCCCTGATATCTTTTAC
CTAAGGTTGAAACATTAATACATGGATACATTTTTGTTTGTGAGACAGCACGCCCCTTTCCGGA
AAATCAGATAAATAACAAGTCCAATTCGAACCAGACAACAAGTTTAAGACTGTCGTCTTTCGAA
TATACGAATCTTCTTATCAACCTTTATTTTGAGCAATAAGATCATTTTTAGAAAATTGCACAAA
TATCGGTTAAGAAACACAGAAGTTAATAAAAAAAGTGCTTTGGCTGCCCACATGTTTGCGGCAG
CTAGCGCAAATTGCAAGAATTTCTGTACACATGTACACATACACATTTATCCGTATCTTTATTG
AATTCTCTTACAGCATTGCAATTGCGATTGTATTGCTTTTTGTGCAGTGTGTATGTGATGCACT
TTCAATTGATTTTTTTTGAGCTGAGTTGTTGTTTTGGCTGTTGAGTTGAGGCAAAAAGCGAGGT
CTATTTCGAGTTCTAGTTCTAGAACTATATTTCTGCTAAAGCAAACAAGCGCGAAGCGCGGTAT
CCTCTAGTCCGGAGCTCCCGACTGGAGCCTCTTATTTGTTTCCCTTTTAACAGAGGTATAAAAT
GAACCATTTAAATAGTGCTTGAGTGTGGCCCACGGTGCTCACGATCATTTTATGCTGCGTTTCC
TGCGTCGGAAAGAAACAATTATTATCCAACGTCCGTAGCGGCACTTCCTGCCCAGCAAAGCTTC
GTTGCGAAACTTTTATACAAACTCATAATTTTGAATTTTTGAGATAACAAATTGCAGCATGCAA
TTTGTGTCTATACGCAATCCTTCGCTACACGAATGCCTCCTTTGCAGGGAGTTTTTTTTATTCT
CGTTGCATGAAAACTTTAAATTAGATTGTTCCGTAGATGTTTACTGCCTCTCTTCGCACTTTGT
GAGTGAAAGGCGATTGTTATTATATATGACTGTTTTAGCGGTCATCTATCTTGGCAAGAACGCC
AATGCATATAGCTTTAGGTAGATCTAAGCTGTTATTAGTTATTAAATTGAGTTATCACATTCCA
GCTTTTGCATTTTTTTTTTTTACCTACTTTGGGCTTATATGTAACAAAATCAAGAGTGCCAAAAG
CGGGAGTGCTTGAAAAGAAGAAACATTGCATCATAAGCATTTAAACTTGCTTCTAAATCCAGAA

GTAGTTACTTTTGGCAAGCAACATGCTTACTTATGACCTTTAAAGCTTGTTTTTATTGACACGT
TTGCACGTTTTTAGCTACGCCTGGCAAAAGAAGCCTAAATTAATCGGATAAACATTTAAATAAA
AAATTATCTTAAAAGAAAATATTTTTAAAAAATAAAATAAATAAAGGAAATAGTTTTTTTAACA
CCTTTTTTTTTCTTTTGAGTCACCAGTGCTGATCTCTTAATGTTTTTATACTCAGAACTCATTAA
AAATGGGTAAAGACAAAAGTTATGAAAAATTGGTAAGTACATAGGATTAAAAGATTGTGATGGG
AAATCACCATCTTTCTTTTTTCCTATTTAGTGTATTTTCTTAGAATAAAATTAAGATAACTTTT
ATTTCTTACTTCAAAACACTATTATCTCTACTAACACCTTCCTTTTCGGCTGTAGGCTATAAAA
ACGTCACTGACTAAGTTAGTATAGCTGTGAAAAGGGGCATAGCAGGCCGGTGATGCTATTAAAA
AAGTGGTGTATTCTAATTAAGGCTTTTAGCATTTCATTTTCATAATCTTATGTACGCTTTTTTC
GACCATTAATAAATAATAAATAAATAAATAAAAATAAATACAGATCATGTATATTATATAATAC
CTCGAAATGTGTAACGCATAGAAGGAGACATCTCCGACCCCATAAAGTATATATTTATTCTTGA
TCAGTATCAACCCCCGAGTTGATACAGCATGCCCGTCTGTCTGTTTCTAATAAATTGTTGGTGG
GAGTGTCGGGTTACGGGTTCGAGTGCAATCGCGGAAGTTTGTATACCCTTGCAGAGTGTATTAT
AATTTTGTCGTGAAATGTGTAGCGCATAGAAGGAGTCATCTACGACCCCATAAAATATATATTT
TCATGATCAGTATCATCAACCAAGTCGATATAGCCATGTCCGTCTGTCCGTCTTTCTGTTTCTA
TGAGAATTAGTCTCTCAGGCAGAAGTGCCTCTTTCTGTTGCACGCAGTACATACACAGCTATCA
TATAGCTGCCATAGGAACGTTCTGTCGAAAATGAGGATCTTGTGTGAAAAAAACTCAAGAAGTC
TTAACCAGACTCGGCATTGACTATTTTCTCTATGCTTTTTTGATACGGCTAAAGCTTTATGAGC
ATTATGAAAAGGTTGGGTCTCCAAGGGTGATAAATGTTTGGCGTGCCGAAGATAGTCTTTCCTT
GTCGTTTTTCTATTAATATATTTATTTATGATAGTCTGACAATCAAAATTACCTAGAAGGCAAA
ATAAAAACAAGTAGGAGGCACTAGTCGGGAGTTCCCGATTAGGGGATACCCTGAACCCTCTGCT
TCCAACGCAAAATGCAGCAAAAACAATACAATTGCAATGCTCCTAATAGAATTCAATGAAGATG
TGGATGAATGTTTGTGTGTAGGCATATATAGAGCGCCTGCCCTTCAACCGGGTGACACATACTC
CAACCTAAAAAGCATCGTGCATTGAAGCAGCGTCGTCCGAAATGACGATGTCTGTACTCGACTA
CGCAACAGGGAAAAAGTTGTGGAGGAGGAGACTGACTGTCTGCCTTGTCTGTAGGTTAGAAAGA
ATTTGTCGTGTTTGCTGAACCACATGGGAACACAGCGTATGCTTTCAGGCTAAGGATGTCCGGA
GTATTCGCGATTCCCCGGACTCAAACCATTTATTCGGTTCACGAACCGCAGTTCGTACTATTTA
CATTGCTTAGTGCACTTCTTTTAAGACTGCTTATCAAGCAATGTACTGCTTTAAAATACACTAT
GTCACAAGCAGTTATATTACCCGTAACCTTAAGAGTCTACTTGTAGTTTAATGGTCTTGTTTAC
AAAGTGTATACAAATTAAGCTGCTCACGGTCGGAATCAGGTAATGTGCTGCTTTAAAATACAGT
ATGTCACAAGTTCTATTACCCTTAAACATAGGAGTCTACTTGTAGTTTAATGGTCTTGTTTACA
AAGGGTATACAAATTAAGCTGCTCAAAGTCGGAATCAAGCAATGTACTGCTTTAAAATACACTA
TGTCACAAGCAGTTATATTACCCGTAACCTTAAGAGTCTACTTGTAGTTTAATGGTCTTGTTTA
CAAAGTGTATACAAATTAAGCTGCTCACAGTCGGAATCAAGCAATGTACTGCTTTAAAATACAC
TATGTCACAAGCAGTTCTATTACCCTTAACCTTAAGAGTCTACTTGTAGTTTAATGGTCGTGTA
TACAAAGGGTATACAAATTAAGCTGCTCACAGTCGGAATCGAGCGCGTACCGACCTGCACTACT
TGCCACCATCAAAACTCAACAGTCCATTGTGTGATAAAAAGACAGATAGGGCTTTTGGATCAAA
AATGTCCCATTTTTGAAAAATTTGCAATGTTGCCGCGTTTTGTTTTTTTGGGATGATTTATCGA
TGTTCGTATCGTTGTTATGAAAAATGTAAAAGCATCATTACTATAAATAGCTGATATATCAACG
AAGGCAAGCAGTTACGTTAAATTATAAATTTAAATTTTAATAATCCCACATTTTTTAACATTTA
AAGATGGCTAAATCCCGCAAAGAGTCCATTAAATGGGCTCCATTTAGGTAGTGCGGTAGTATTA
TTTGACGTTTGGAAGGAAAACTGTGGCGGCTATGTGATTTAATTATTTGAAGAGGCGGTACTTT
CGCCACTCACATGTAACCGACAACAGACACTTGCCAATGGATCACCAATATGTGAAACTTGAAA
ATGTGACATTTCTTAAGCATAGTGGACAAAGCCTAATACACTTTAAATACACAATCGTCACCGT
CAAATCGAAAGCTTGATGTTTTTAATAATTCATCATCGATAGTTAGCAGTTCTCACTCGCTCAT
CTCGAAGCATTGACGATAGTACAAAACTATCGAAAATTCATCGTCAAGTAGAATCGGTGCTGCA
CGACACTCGACAATCGTGCTCCTACCGTGCTATCGGCAACTTTTAATTGCTTATATTTCATAAA
ATACATTATAAAGTTAAGTTCCGTCACCGGAAATTGAATCTTGAGAAAAAACTACGTGGAATAG
ATACCATCCCCGACTATTTGGACATTCCTTATGTGTGGTGGCTACGACGAATAATAATCGAAAT
CTACGTGAAGCGACCAACGTGAAGAATTACCGTAAAGCATTTTTTTTTTTTTTAAATTAACTA
TACCCATTCAATGCTGGATCCTAACAAAATAAAATTCAAAAC~~TTTTTGTCATACAAAAATCAAA~~
~~TGCATTTTTACTTGGACATGTATGTATGTGAAACGGCATCTGGCATCAGGAAAAGCAAACAG~~
~~TTGTGAATATAAAATTAAATGTACGAGATAGTCAACATGATTATCTAAGTGGCCATACGATA~~TC
ATTTTTTCTTGGCATCCTTGAGACCAGCGCCAGCGTTGAATTTGAAGAAAATTATATCGCCGTC
TTCCACGGTATAGTTGCGTCCCTGCTGGCGATATTTGCCGGCCGCCTTAGCAGCTACCTCACTG
CCCTCCGCTTTAAAATCTTCGAAGTGCATCACTTCGGCCATAATGAAGCCCTTCTCAAAATCAG

TGTGTATACGTCCAGCGGCCTGTGGCGCCTTTGTGCCCTTTTGGACCGTCCAAGCCTTGACCTC
ATCGGGGCCGGCAGTAAAGAAATATTCCAGCTGCAAGGCCTTGTACCCAGTAATAATGATCTTG
TCCAGCTGACTTTTGCATTTGGTCTCCTCTTCGTAGGCCTTGCGCTCTAAATCATCCTTTTCAC
TCAGCTGCAGCTCGAATGCACCTGAGAAGGGTATAAGTAACGCGCCCGGGTCGTTCTTATCAAT
CCAATCCTTAATCTTGGGCAGCCATTTGTTTTTTTGCGGATAAAATCTTTGTCGGAAAGATTG
ACCAGATAAATGGCTGGCTTGGAGGTCAAAAACAAATATTTGTTCAAAGTTTCAATCT

## *Xc* Inversion:

### *D. novameciana* 15010-1031.00

#### Distal breakpoint

>Nova00_Contig2186:…1713..28358…(*GJ17050*[+](*Ir7g*)-
*GJ18715*[+](*CG32698*))

CTGCAGCAGCGACGCGCTCACCTGGCCATTGGATACATACGCAAACGTGTGCAGCATGCCGCCA
ATCTGACAGCCGTCTTTCCGCACTATTCCAGCCGGCTAATCGGCTGCCTGTCGCTGAATGCGCA
CAACTTGACCAGCTTTGAGCTGTTGGGCTTTCCCTTCCAGACGCTCGCCTGGCTGGGCGTGCTG
ACCAGCTTCCTGGGCGTCAGCTGTCTCATGTTGTGCACGCGCCGACGTGTCGCCAGGGCGACAA
TGCTGCTCGCCGTGCTGGCGATTGCCTGGGGCCAGCCCATTGTCGCGTCCGGCCTTCGTCCCGC
CCAGCAGCTGGTCTACATCAATTGGCTGGGCTTCACGCTGCTCGTGCGCGCCATGTACTCGGCT
CTGTTCTACCATATGCTGCGCCAGCAGGTGCATCAGCGTTTGCCCCGCAATCTGTTCGAGCTGA
TGGAGGGCAGCTATACGGCTGTGATGAATCGCATTACCGCCCAGGATGTGGGCGAGGTGAGCAG
CCTCCAGGTGCTATTGAGCAATATGCGTGCAATTGTCCTTGCGAGCGATGTGGAGCACGATGTC
CTGGACCAGGTCGAGTCGCGGACAACGCAGCGGCGCCTCTTTGGCATACTCTCGCGCCAGACCC
TACTGCATGCGGCACAGCGTGCCCACAAGCCGGGCGCCTACTATATGCTGCCGCAGCATGTGCT
CGAGCAGCAGCTGGCCATCTATCTGCAGAAGCATTCGCATCTGGTGCAGCGGCTCGATGAGCTT
ATCATGTCTATTCAGGCGGTCGGCCTGATCAACTACTGGGCCGGCCAGCTGGGCAGCGAGCGCT
ATTTTCGCAGCACATTCATGTATCGGGACAATCGGCTGCGCCAGCCCGATCTTTGGGGTATCTA
TATAATTGTGGGCGTGCTCTACGCCCTCGCCACGCTCGTGTTCATCTGGGAGCTGCTCTCGGCG
CGCCGGCGTCAAGTCCAGTAG~~TCGGCCGAGGCCCAATTTAATTTAATGTTCAATTCACAAA~~GTTC
TCACGAGTGAAAATAAAAACAAAAAAGAGAGAGGGCCACACTGTTTTTTCAACTGTTTGATATC
GATAGCATCTGTTATCGTTGTTGGTATTAATCGTTTCTGGAAGTGCGATTGGGACGGCAAATAA
ATGAATGAATGAAATAAGCTTGACGGAATGAGGGAGATGACAAGCAGCTTTGAAATGTCGATAG
CGATAACTTTTCAGCTTTCAATAAGGTCGAAACGATGTGCGTCAAATTAGCGGCGGTGACTTAG
CGTCGAATTTTGGTACTTGTTATCGGAAAACTATCGGTCACTTTCAAATGCATTTATTTATGTT
TTTAAGCACGAAACTAAATAAATTTAAATGCAGAATTGCAAAATATTAAATTATTTACTATCTA
CATTCATTTATTTTTGAATTTTAATTTATAATATGGGTAAACAAAGGGGTTGAAAAGTAAGGTT
TTCGCCATTCTTTAAATATCTTAATTCAGGGATAATGGCCCCGAAAACCGCATATACACGATTG
AAGTCTATAGTTTCACCTAACCAATCGTGAAAAAATTATAAAAATCGATCAGCCCTTTTTTAAG
ATATTTGTACTACAGTGCAGCAACCTCGTTGCTAGCCCCATACAAAATGACCGTAAAAAATCAT
CCTTGCACTTGTTATACAGTGTTGAGCGGGAACAAATGAATTCAAAATACTTGTATATGGAAGA
GAAATGCAGATATATATGAAATAATTTTCATTTTACAATAAAACATTATCTTTGGTAAAATAGA
CAGCTGCAAAAGACGACAACTATGCATTTAAGTATTTATACACGTATAAAAACGAAAAGGTGGC
AATATTTCAACATTTATTATTCTATTTTCCGATATAGATCGCGAATTATCTATTATTTTGCTTT
TTACATCAAGAAAATCGTCCAGCCAGTTTTGAGAAATTCGCATTTTTCTATTTTTTTTATTAT
ACCCCCTGTTATACAAAAAAAACTAGCTGGCAACATTTTGCCATCCTTAAATGTTAAAAACTGT
GGGACTATTAAAATTTAAATTTATTATATAAAGTAACTGCTTGCCTTCGTTGATATATAAGCTA
TTTTTAGTATTGATACTTTTTTATAATATCGATACGAAATTCGATAAATCATCGAAAAAAAATC
AAAACGCCACAAAATTACAAATTTTTCAAAAATGTGACATTTTTGATCCAATAGCCCTGTCCGT
CTCTTTATTACACAATGGACAGCTGAGTTTTGATGGTGGCAAGTAGTGCAGGTCGGTACGGGCT
GGAGTTCGACTGTGAGGAGCCTGTGTAAACAGGACCATTAAACTACAAGTAGACTCTTATGGTT
GAGGGTAATAGAACTGCTTGTGACAAAGTGTATTTTAAAGCAGTACATTGCTTGATAAGCAGTC
TTAGAAGAAGTGCACTAAGCAGTGTAAATCAGTACGAAGTGCGGTTCGTGAACCGAATAAACGG

TTTGAGTCCGGGGAATCGCGAATACTCCGGACATCCTTAGCCTGAAGGCATACGCTGTGCTCCC
ATGTAGTTCAGCAAACACCACAAATTCTTTCTAACCTACAGACAAGGCAGACAGTCAGTCTCCT
CCTCCATAACTTTTTCCTTGTTGCGTAGTCGAGTGCAGACATCGTCATTTCGGACGACGCTGCT
TCAATGCACGATGCTTTTGAGGTTGGAGTATGTGTCACCCGGTTGAAGGGCAGGCTCTCTATAT
ATGCCTACACACAAACATTCATCAACATCTTCATTGAATTCTATCAAGAGCATTGCAATTGTAT
TGTTTTTGCTGCATTTTGCGTTGGAAGCAGAGGGTTCAGGGTATCCCCTAATCGGATACTCCCG
ACTAGTGCCTCCTACTTGTTTTTATTTTGCCTTCTTGATAATTTTTATTGTCAGACTATCATAA
ATAAAAATATTAATAAAAAAACGACATGAGGAAAGACTATCTTCGGCACGCCGAAGATTTATCA
CCCTTGGGGAGACCCAACCTTTTCATAATGCTCATAAAGCTTTAGCCGTATCTAAAAAGCATAG
AGAAAATAGTGAATGCCGAGTCTGGTTAAGATTTCTTGAGTTTTTCACACAAGAACCTCATTTT
CGACAGATCGTTCCTATAGCAGCTATATGATATGATATGCATGTACTGCGTGCAACAGAAAGAG
GCACTTCTGCCTGAGAGACTAATTCGCATAGAAACAGACGGACATGGCTATATCGACTCGGTTG
ATTATACTGATCAAGAAAATATATATTTTATGGGGTCGTAGATGACTCCTTCTATGCGCTACAC
ATTTCACGACAAAATTATAATATCCTCTGCAAGGGTATACAAACTGCCTCGCGTCGGAGATGTC
TCCTTCTATGCGTTACACATTTCGAGGTATTATATAATATACAAGATCTGTATTTATTTTTATT
TATTTATTTATTATTTATTAATGTTCGTCAAGAACGTACATTAGATTATGAAAATGAAATGCTA
AAAGCCTTAATTAGAATACACCATTTTTCACAGCTATACTAAGTTAGTCAATGACGTTTTTAGA
GCCTACAGCCGGAAAGTTAGGTGTTAGTAGAGATAATAGTGTTTTGATGTAAGAAATAAAAGTT
ATCTTAATTTTCTGTATTCTAAGAAAATACAATAAATGGGAAAAAAGAAAGATGGTGATTTCCC
ATCACTATCTCTTTATCCTATGTACTTACCAATTTTTCATAACTTCTGTCTTTACCCATTTTTA
ATGAGTTGTGAGTATAAAAACATTAAGAGATCAGCACTGGTGACCCAAAAGAAAGAAAAAGGTT
GTAAAAAAAACTATTTCTTTTATTTATTTTATTTTTGAAAATTATTTTTTTTTAAGATAACTTT
TTATTAAAATGTTTATACGATTAATTTACGGCTTCTTTTGCCAGGCGTAGCTAAAAACTGGCAT
TATTTGAAGCTGACCGGCTCGAGGCCATTGTTGGTCTAAGCCAACAACAAAAACAAGCTTTAAA
GCCCATAAGCTTGCCAAAAGTAACTACTTCTAGTTTTAGAAGCAAGTTTAAATGCTTAGAATGT
AATGTATCTTCTTATCAAGCACTCCCGCTTTTAGCACTCTTGTTTTTGTTACATATAAGCCCAA
AGTAGTCTAGCAAAAGCTGGAATGTGATAATTCAATTTGATAACTAATAACAGCTTAGATCTAC
CTAAACCTATATGCATTGGAGTTCTTGCCAAGATAGATAACCGCTAAAACAGTCATATATAATA
ACAATCGCCTTTCACTCACAAAGTGCGAAGAGAGGCAGTAAACATCTACGGAACAATCTAATTT
AAAGTTTTCATGCAACGAGAATAAAAATAAAACTCCCTGCAAAGGAGGCATTCGTGTTGCGAAG
GATTGCGTATAGACACAAATTGCATGCTGCAATTTGTTATCTCAAAAATGCAAAATTATGAGTT
TGTATAAAAGTTTCGCAACGAAGCTTTGCTGGGCAGGAAGTGCCGCTACGGACGTTGGATAATA
ATTGTTCCTTTCCGACGCAGGAAACGCAGCATAAAATGATCGTGAGCACCGAGAGTCACACTCA
AGCACTATTTAAATGGTTCATTTTATATCTCTGTTAAAAGGGAAACAAATAAGAGGCTCCAGTC
GGGAGCTCCGGACTAGAGGATACCTTGAATCCTCTTCTACCAACTCCAAATGCAGCTCGCGCTA
CGCGCTCGTTCGCTTTAGCAGAAATATAGTTCTTGAACTAGAACTCGAAATAGATCTCGCTTTT
TGCCGCCGTCTCTACTCAACCCAAAACAACAACTCAGCTTAAAAAAAAATCAATTAAAAGTGCA
TCACATACACACTGCACAAAAAGCAATACAATCGCAATTGCAATGCTGTAAGAGAATTCAATAA
AGATACGGATAAATGTGTGTGTGTACATGTGTACAGAAATTCTTGCAATTTGCGCTAGCTGCCG
CAAACATGTGGACAGCCAAAGCACATTTTTCATTAACTTCTGTGTTTCTTAACCGATATTTGTG
CAGTTTTCTTGAAATGATTTTATTGCTGCAAATAAATATTCATAAGAAGATTCGTATATTCGAA
AGACAACAGTCTCAAACTTGTTGTCTGGTTCGAATTGGACTTGTTATTTATTTGATTTTCCGAA
AATGGGGTTGCTGTGTCACAAACAAAACGGCATCAATGTATTTATATTTCAACCTTAGGTAAAG
GATATCAGGGATTTGTTACATACAGAATAATTTATATGTTATCCAGCAAAGTAAATGCGCTCTC
AACCGAAACGTCGAACTATATAGTTGGATCAACAGGTCAATAAAGAATTGCTGACTCGCAATAG
CGTTTTGCTGTTGCTATCACGGAATAGTTAGCTGTTTAAATGTTTAGGCAGCGCGCGTAGCAAA
GGCGCTGTGGGCATAATTTCAGTGGCCAGTGCAATTGCAGGATGCGTTTCCTAATATTAGATTT
AAAACGTTTTACGCTTTTGCCGCCAGATGTGGCATTAAGTTAAATGGCAACCTTCATGTCGACG
ATGGCGAAGGCTATGAGACACCGACATCAAACCGACGACGGCGGCGGCGTAATCTATCGATTTA
TATTATTGTCACGTTTATCCCAAGATTGAGACTTGCCATTTCGTAATAGGAGGAGGCTGCACTC
AGGGAAGCTGATCAGGTAGGGTTTCAGGTAAAAGCATTCGTTGTTGATAGTGTCGCTTGTGTTT
TTACAGTGCATTGCAACTTGTACGAGCAATTAAGGTAGCCGTTGACCTGGCAGAGGCGAGTAGA
AGGGTTAAGGATAATACAAGTAGAACTAGGCAAAGAAGCTACACAAGAGAAGCGTAAATGAATA
ATGAATGCAATTCAGCAATATTCAATTTCAATGCGTCACTTAATCGGGCGTCACTCATTAATTT
ATGACAGTCATTTTTAACGCTTTGCTGGCTACCGACGTCGACAATGTAGACGTTGATGCCGACG
CCCAACGCCCGACACTCTGCCAGTGACATCTGCCCCGCCAAGCACACTTAGTTGCCATGCCATA

CGTCAAATATTCAACGCAATTTTTCAATTGAATTGAATGAATTTTTGTATTGAGAGTATGAATG
CCGGCTGACAAACGCCAAGGCAAGCAAACAGAACCTTGCTGCGCTTGCATTTCCATGTCCTTAC
AGAGAGAATGCCCAACTTTGTATGAACTTTTGCACACAAGATATTCATAAAAACTTATGGATAT
AAAGGCAAGTATGATATAGTGATTCGAATCGGTCGATTTCGACATATTTACTGCCTGAAACAGA
TAGGCGTATGTATGCAAACTTTCATGTAGACAGCTTTAAGGCTTACACTAGCTTGCATAAAAAT
AGACAAATGGACGGACAAACAGACGGACTTGGCTATATCGTTTCGGCTTTTGATACTGATCAAG
AATTCATAAACTTTATTGGCTCGGATATGTCTAGTTCTATGCGTAATACTATTCATGACAAAAT
TTAAAGAATTTTAGGCCAAGTAACGGGCCTTCAGTCAGGAGTGCCCGACTAGTGGATACCCTAA
ACCTTCTACCAAACCGGATATCGATATCGATTTTTAGCTATGTTCAACATTTCGAACTTTTAAA
GGTTTTGCGATCAGAGCGTTCAATAGTCAAATTCGACAGACGGACAAAGGAACGGATGAACAGA
CTGACGTGCAGACATTATACTCTTTTTACTCCTATTCCATGAATGCAGGGTATACAAATATTAG
TTTAGAAATTCTTTCCGCCAAAATGCGGTTACGATGTTCATTAAAAGAAATGCCTGAAAATGCA
CTTTCGGTTGTCACTGAAGTGCAAAGAACTATAATATTTTTCCGCGATTCTGTTGCCAGCAGAA
GAAAAATGGTAACTGGGAGGGTAATTTTATTATTCAAACCCTTCCAAAATGTTTATATCGAAAA
ATATCCTCACGTTGAACACGGACAGACACACGGACATGGCTAGATCGACTCGGCTGTTGATGCT
GATCAGGATATATCATATGTATACAGAGGGGGCACCCCTCTCCCCATCATCAAATAATTAGCCG
GACATTTCTCTCTTCAGTATTTGCATGTAATTGAATGCATTTCTTTTAGTTATTAACAATTTAT
ATTATTTCTAATTGCACTTATCTTTGCAGGATATCTCCACTCTGCTATTTATAATCTCATGCAA
GCGGACATTTCTTTTCAAGTCGTCAACAGGTGCGTGTAACTATTTGCATTATCTTATAGTTATG
GCAATTGATATTCATTTCCAAAGTCACATTCTTTTTTGTCAAAACAACAACCAAGAAAGGACAT
CACCGAAAGCTAATAAATATGGAGAACATATTTATAAATATTAGATTGAGCGATTGGCTCTCCG
CCAAGTACTCAATCGCACATATACATATATATCATATACATATATATCATACTTATTAACACGC
TGAAACTGATATGCAAGAGTGTCATATATGTATAAATGATATTAATTAAATCGCTTTAATAAAT
TTAGCCTACTGAAAGAGCTCTCTGGGGCGTTAAGGGCGATATATTTATATATGTATATGCATGG
AATAGTGTGGACTTTTCTTTTCAGGTTTCCCTGGAATTAGCCCTTTCTTCGCAAACCGTTAAAT
ATATATATATTTTTGAAAAATTAAATTATAATTAGAAAAAGGCAAAAACGAATCTTAATTATAT
AAATATCTTTTTCTCCGTTTTAAAGTAACCCAGATAGGCCACAGAGCTGGACCGTACTTCGGGT
TCTCTGGTCCCTGCCTCTAGACCGAGACACGGAAGGACTGCATGGAACGGACTTACTCAATTCT
GGCTTTTTCACTGTTGGATTCTCGCCTTATATGGCCCGCTTCTTCGTCTTGCAGTGTTGCGCCT
TAGGCCTGGTCGCGCGTAGGATCTCTGGCCCGAGGAATATCTTGGGTGGCCTGGCTGGCATACT
TCGTCTTGTGTATAAGGGTATATAAGGCGAAAGCCAAATGTATGTAACAGGCAGAAGGAAGCAT
CTCCGACCCCATAAAGTATATATATTCTTTATCCGCATCAATAGCCGAGTCGATCTAGCCATGT
CCGTCCGTCTGTCCGTCCGTCCGTCCGTCCGTATGTATGAACGCAAGGATCTCAGAACCTATAA
GAGCTAGAGACATTGAAATTTTAGATGTAGATGCTCCTAGTTCCCGCGCAGATCGAGTTTGTTT
CCGATAATCGATAACTTACTCCGTTTCCAAGCAATCGATAAAAATCGATATCGATATCCTGTTT
TTTGGGCAATTTCGGTAAATAATAAGAGCTAGAGTCACCAAACTTGACATATAGCTTCTAAAAT
AGAATATATATGCATTTGATGTTGGAAGAAGAGGGTTCAGGGTATCCCCTAGTCAGGAGCTCCC
GACCTGAACCTCTTTCTTGTTATTTTTGGTATTTTGCATTTTTTTTCGGCGTTGCTTTTAATTT
GGCAATGTACATTTGTATCACTTCCGCGTGTCGTGCATCGCTACTATCCTTTGTTTTTTTTTTA
ATAAATAAATACCTTTTTTTTTAAATATGTTCCTTTTATTCCCTGCGCGGCGTTATTCTTTTA
TTTTATTTTAGGTCGTTAGTGGCACGAAACATCTTAATGTAATTATTATGTGTGGCCGGTTAGA
GCTGCATTTTGGGTCAACGGCCCAGATGAAGGCCAAGGGCATTGCCAAGGGCACCCGATCCGCT
CTCCTCCTGATCTCTCTATGAGCGAATCGGGCGGCCAATCGCAACCGTTTCCCCTCTCAGCGCA
GTGGATCGCAAGCCGGGCTTTGCCACCCCACAGATAATTGGGAGCCACAGCGCGTTCGACTTCA
CCCCCGATACCCTGACCGTGCAACGAAGAGATATCGACGGGGCCGATAATATGGACGCAACCA
ACTAAGCAAAATATTGATGCTGCGGATAATGGCAAATGCAACAAATCATTTGAGACATGTTTCA
CACTTTTTGCAGAATCCAAGTTTCCTTTGTGGAAAGGGCGATTCAAAGATTTTGTGATGGAGTT
TTAAGTTTCTCCAATTTGCTTCATAAGAACCTAATGTATACGACAGGGACTTGGGAAATTCCAC
CCACAACAGTGGTAAATTGGTGGAAAACGTTTATTTACAGTTCGTTCAGTGTCAAAAACATTAT
TTTTTGAGAAAATTTATATTATACGTGCAACGGGCAAAGCCGGGTTATACCTGCTAGTAAACCT
TAGCTTAAATTGGTGGGTAGTTCCAATTGGTTTATTTATTTGATTTTTAGCTATGTTGTCTTCA
TGTGCACCTTTTTTACATTTGCGCTGTGCGTTGATCACGAATCAGTCAGTCAGTCAGGACAAAC
ATTTTTATATATAGAGATATCGGGGGGTTCACAATTAAACAATACTTTTGTTTTAAAGTAGCTT
CGTTCAGGTATATACAGGTAGTAGATAGTATATATGGTAGTTTATGTGCAGTTTGTTTAGTTTA
GTCTAGGCTCTAGTTTGTTTGACTGTCTCAGGGGCAAGGATTACCTTATAGATGAGACCCTTAG
CGAGAGTTTGGTGTTGGTATTGATAAGTCAGCTCATACGCCTGATATTTCATTTGAAGTTGTTA

ATTGGCTTGGTGACATGCCTTTTCAATGTAAGTCGTCTGTCGGGAATGACGCCTAGGTATTTGT
CACACACATGCTCAGAGAGCTCTCCATGAAAAGAGAGAGAGGTCAGGAGTCTTTGCGCAGGTTG
TGGGTAATGGTAGCGTATGTACTCCTATAGGATGTCAGTTGCTTTATACACACACTTTTTATTG
GCAGCAAGGTCTGTTATGGGTTCAAGTCTTATTAAGGGAACTATTTTTTATTGATCTTGATAGA
AGAGGGTTAGGGGAATCTCCTGGTCGGGAACCCTTGACTAGAGCATCTTCCTTCTTTAATAGGT
TAAGCCAAGGCAAACATAGAGTGCTGGATCTTCTCGACCCAGGCTCGATATCGAAAAAGCCTTA
TCCGCTGTGTAGACTGTGTAAAGCACTAACCCGAGGACCCTGTCGTCAGGGACACCAGCTCGAA
TGGGGCGCATTCTGAAGCTAGCTTCCCTAGAGTTATAATATTTGGCAAGTAGCTTTTCGTAAAG
TATATATAGATCAATTATATACCTTTTTGGTAGAAGTTTAAAGCTATTTGCTAGTGGAGCATTT
CCAACTAGAGCATTCTTACTTGTTTTTTAACCAGCTCACATATATGTATAAGCAAGGTATGTGT
TATATTTGTGTCTGCTTATACTTATGTCTAGTCGATGATTTGAACAAACTCGTACAGAGGGTGC
TTTAGAGGGAGACCTCTATATTCAAAATTGTATGTTGAGTCTAATCTGTTATAGGTTTTAAGAT
CGACGTTTCCATACAGACGGTCACAAATATAGACGGCCATGGCTTGATAGGCTCGAGTATTGAT
GCTGAGCAAGGATATAAATGTGTATATAGGGACGGAGACGCTTTCCTTTGGACTGTTTAATACA
TGAGGGTATAATAATGCGCTTTCCAACTTTTTGAACAGAGGAAAAAAGAATAAATAAACAAGTA
AGAGGTTCTAGTCGGGAGCTCCCGACTAAGGGATACCCTGAACCCTCTTCTTCCAACATCAAAT
GCATATATATATTCTATTTTAGAAGCTATTTGTCAAGTTTGGTGACTCTAGCTCTTATTATTTA
CCTAAATTGCCCAAAAAACAGGATATCGATATGGATTTTTAACATTATCGGAAACAAACTCGAT
CTGCGCGGGAACTAAGAGCATCTACATCTAAAATTTCAATTTCTCTAGCTCTTATAGGTTCTGA
GATCCTTGCGTTCATACATACGGACGGACGGACAGACAGACAGACGGACATGGGTAGATCGACT
CGGCTATTGATGCTGATCAAGAATATATATACTTTATGGGGTCGGAGAAGCTTCCTTCTGCCTG
TTACATACATTTGGATTTTGCACAAATACAATATACCCTTATACCCATTTTTAATGGGTTCAGG
GTATAAAAAATCCCCAAAATGTTTGATTCTACTTTTAGAATATCTGAATGTTAAGTCCACTGCC
AGCTGTTGCGCATTTTGAAATTTTTGCTATAGGCGCAAATCTGAGATGTAGTAAGAAATAATTT
TCAATAATAATGTATAGTTTGAATGGCTGACTAGCAAGGAATATGATTGTGGGTGCGTTTGTTA
GTCTGTGCAAGAGTGTTTGTTCTTGTATATGCTTATTTACTGCAATTTCAATTAAATTGTTAGC
ATATCTTTCTGTGGTACCATTAAATTAAACTATACGAAGAGCAAGAACAACAAGTGCATAGAAT
GTTGCTGGGCTTACTGTTTTTATGCAGGCCCGTAAATTATTTTAATTAATTTGGTGCGCAGCTC
CCAACTAAAATTGAAGTTACAATTAAATTGAATTCGCTTTAATTGTGAAATCGACAAATAGCAA
ATGCTAAAAATTGGACCACTCTTCGGTGTCAGGCATTTTAACTCGAGTAAAAAAACTGTTGGGA
GTTTCTGACCGACAGACAGAACATGCGCCGCTGCCGCAAAGCCACAGTTTACTAGACATAGATT
TGATTTAAATCCAATTGGTTGCGAATTGACTTTGAGTAAAGAACATCTCGTGAAATTATATTTT
TTTTTATACCCTGAACCCATTAAAAATGGGAATAAGGGTATATTGTGTTTGTGCAAAATCCAAA
TGTATGTAACAGGCAGAAGGAAGCATCTCCGACCCCATAAAGTATATATATTCTTGATCAGCAT
CAATAGCCGAGTCGATCTAGCCATGTCCGTCTGTCTGTCTGTCCGTCTGTCCGTATGTATGAAC
GCAAGGATCTCAGGTTCTATAAGAGCTAGAGACTTGAAAGAGACTCCTAGTTCCCGCGCAGATC
GAGTTTGTTTCCGATAATCGATAACTTACTCCGTTTCCAAGCAATCGATAAAAATCGATATCGA
TATCCTGTTTTTTTTGGCAAATTTGGTAAATAATAAGAGCTAGAGTCACCAAACTTGAGATATA
GCTTCTAAAATAGAATATATATATGCATTTGATGTTGGAAGAAGAGGGTTCAGGGTATCCCCTA
GTCGGGAGCTTCCGACTAGAACCTCTTACTTGTAGAAAGCGTGCTGCTAAAAACCAACTGACTC
TGGGGTACAAGGGATCTGGTAGCGAGCACATTCGATGACTTCAGTCTTCTTACCTGTTTGATCA
AATTAAATAACAAATTAAACATATAATATTAAAATTCGCAAAAGCTTCTGAATTGTAACCGGCG
ACTGCTCGATTGTCAGCCCGACACTCGAAAGCCTACGCAAAGCACGGGCTCATACCAAAGACAA
TATACTTACATACATTGTCCTTGCTCAAGCCGTCCCGTCCCGTCTGCACAATTATATGGATTTG
TGGATCGAATTGGTCCGTAAAAATGTGTGTTGGCCACCAATCAGTCAGTCTTCCACTTAATCGG
TAAAGACAAACTGATATATATATATAAATACATTAGGTGTGCCTGGAAATCAAAACTCGCCAAC
CAATGGAAAATACAATTGCAAGTAAACCATTGACTAACTAGCATATTTAAGGGCTGCTATTATA
CTCTAAACACAGTCGGCAGTAACCGACGGCTACAACCTTACTTGTTTTCGTTAGTTGCTGCTGA
AGGATAATTTTAAAGGCAAGCATGACTTTCCCATTCATATATATATTTGTGTTTTCTTCGGCAC
CAACGGCAGACGGCGTCATCTGGTGCCTTATGATTTAATAAAGTTGCAAGTGTGCGCCGCCTCA
ATGTGCGGCATACATACGAGAGAAGAAGAATCGTCCACATTATCAATGATACCAACTCCGCCAT
CGTCAGCGCTGCCGGCCTCTTCTCGGCTCGTTTTCAGCCAGCCATATTATCTGGCTCCGGCCCA
TGTCGTCGCAGAAAGTAATCTATGCTGTGACGTTGTCGTCATCGTCGGCGGGACATATTAAATT
GTGTTAAAACACTTGCGCCCTGCATGTCCCACCGGCTCACAGTCGTTATTTCCATCCTCTATGG
CAGCCAGCCCAGCCTGGGCCTCAGCTACACCTCCGCTGCATTTCTGGCAGCTTCAATTTTTGTT
GGTGTTTTTCCTTTAATTTCCGTCAAATTAAAACATATGTCACCCATAGCCTACAACGTTTTGA

ATCATAGCGCATTTAACTTATTGAAACCACGAAAAATACATCCTTATTGTATTATGGCTTTTGT
GTTTCTCCGAAGTGACATATTTTCGACCGCAGCTTTAGTTACATATACTGCTCACGATATGGTC
AATTGTTTGGAACGGTAGACAACTGTAAATAAATGCATCTATCCATTTCGAACACACTGTTTTT
GTTTTCAGCCGCAGCAGCAGCAGTTGTACCGTGAGGCAGCACAGTGGCAAGGCCAGGACTTCAC
ACCAAAATGCTCCACGCTTCATAATTAAAAGCTGCATTCCGAAATTTTTCAATAACAGCTATAA
ATCAAGCATATGGATGCCATGGGAGCTTGCTATACATAAATAACGTTATACCCACGGAAGATA
CACTCACACTCACATCGACTTGTACAAGCACGAACACACAAGCTAATAGCAGCAATAGTTTTTT
TTATACCCTGAACCCATTAAAAATGGGTATAAGGTTATATTGTATTTCTGCGTAAGCCAAATGT
ATGTAACAGGCAGAAGGAAGCATCTCCGACCCCATAAAGTATATATATTCTTGTTCAGCATCAA
TAGCCGAGTCGATCTAACCATGTCCGTCTGTCTGTCCGTCCGTCCGTCCGTCCGTCCGTCCGTC
CGTATGTATATCGATATCGATATCCTGTTTTTTGGGCAATTTTGGTAAATAATAAGAGCTAGAG
TCACCAAACTTGACAAATAGCTTCTAAAATAGAATATATATATGCATTTGATGTTGGAAGAAGA
GGGTTCAGGGTATTCCCTAGTCGGGAGATCCCGGCTAGAACCTCTTACTTGTTTTTTTATATAG
CAGACTCAGGTTTGGCACGTATCTCGCTGGCAGCAGCTTCATAGATGACCCAGCCAAGCGCGAA
ACGGCCCAATTGGCTCCTCATTAGTTACGCCTGTGATATTTCCAGTATTAAAAAGTGTTTTATA
TTCTATACATACACATGAATGCACGTCGCAAGCTGCGCAATTTATGCAGTGTGTGTGCTTCTTC
TTGCTCCTTTAAAACAAAATTATGTTTTGAAAATTCATTTTCGTTCAACTTTGTTGCATAGAAT
ATGATCTTTACGAAACTTTACGAAAACAAGTAAATGTAACAAATCGCATTAAACTTCCTTAGAC
AATATTTTTGTTTTCAATTAAGATAAAAGTGAGAACGTTAAATACTCTTAATCTGTATATACAA
TACAAGCATTTTATTCTGAATTTGCCACTTAAAGTGTCCAGACTTGGGAATATTCAAAATGGGT
CCTCGAACAACAATTGAGCAAAGAGAATTAGTTATAAACCACTTTAAGAACAGTCAACATCAAT
GTCTCTACAATGCAAAAACTAATCGAACGTTTCGTTCGTGAGAATCGGGATTAAGAGTTGCAGG
CTTTTTTTTTAACTGTAAGTAAATACAGTAAGTAATATACAGTTCATTAATAAATATGAACTAT
TCCACCGCTGAACTATATAGACGCATGTTGACCAATATGCAATTTCAGTACAATGGGATTTTCT
ACATTATTGAGCTGTCCCTGGCAACGTCGGGTAATTTCTTCTCTAAATGTATACCTATACATAT
GTACATATATTCGGTACAACAATTGATAGGGATGCGCGCGATTGGTGGGGCAGGGGAGTTGTGG
ACATTTTACCGGCTTAACGAAAATAAGACGGACAATGTCGCGTCGGAGCATGCAAGTATATAAT
ACTATACATATAAGAGATGATACAACATTTTTCTCCGTAGTGAAGAATTAATTATTTTTTATTT
TCTAATACTTCAGGAATTCATTTTCTGAGTATTTATGTATGTAAATAAGAATCAAAATTCTAAC
ATTTATTAGCCTTTGTGAAGCAGTTTTAACACCAAACATAATATTAGAAATTAAATAAAACCTT
ATATATATATATATATAAATATCTATAAGATTATTTTCTATCTATTTTATAGGTTTCTAGGAAT
GACAAGCCTAGTTGAGTACGAAACCAAATTCAGTATCAATTTCTAGGGGATTTTACCGGCACCA
AGTGGCTTTCCAGCGTGTTTATTTAATTAAAGCTAGTTTTTTTGCGCGACTGGAAGGTAAATAC
AAAACGTAAACAAACCGTTTGGCACGTTAGCCGGCCTTAAGTGAAAATTCACCACGAGTTGAAG
AGAGAATATTGGCGTGGGTAGATGCGAAAAAGTTGGGCGAACTCAATTTCATCGGTTTTGCCTG
TGTCAAGAGAACATAACGGTTACATAGAAGCAGGCGCTGGCAACTTTACGCAGCCGGAAGCATA
GCCAATGGTCGCAATAATTGGCAAACAGCCAACGGTAGCATGTTATGAACCACTAGCATAACAC
GAGCATACCATAAGCTCTCGTAAGCTCAACACGAAGTTGCGGCACGAGTAGGACTTTCAGCGAG
AATTCCAACTCACGAGTCGGTTGCCTCGAGTCCGTTCTGTAAGTCAACCAAGCAACAGTTTTGT
TTGAAACGTCAACACAAGCGGTCGCCATGACCCTGTGCGCATTTGTTTTGTCGTGGCTAAATTT
ACGTCGCGTGTGAATTTCTACGTTAGTTTGTGAAGTGTTTTCAGCAGTTTCCACTGCAAACATT
TTTTTTCGTGATTCAAAATTCGATGCCAAAATAACAGAATATAATGCAACAATAACACTGCATG
GATGCCTATTGAATTCAGAATAATCTCATAAACCATGTACACAGAATTAGGTGCAAATCGGTAA
TGTTATAATTGATATGCGGTATGTTATATCGACAATGAAATGTCAACGACGAGAGGCTAAAAGC
ACGTGTATCATATCTTTAAACAAAATGTCAGAGAATTCCAAAAATCAAATGTAAAGCTAATTTA
CAGTAATTTGTTCTCATTCCTTAAAATATACAAGACACACACACACACACACACAGATACACAC
AAGGACACGCATTCGAGCGCATAACAGCAACAGGAAGCATATGCACAATGCTTGCAACATTGTT
AGTGTAAGCGACTTTAACGATTCTCTCAACAAGTAACAAAACACAAACGCGGACCGAGCACAAA
AGCCAAGTGAGAAATGAAATGTTTGACTAATCGTTTCTCGTTTAAATGCCAGCATCAAACCAGC
ATCAAACCATGGACGTAAGTGCAACGCAAAGCCGTTTAGCTATAAAAAACCGTTGCTCTTGAAT
GCATATTGAATGAAACTAATACATTTACATTTGAGTTAGCAAAGAAAAGTGCACTGAGGCTGGA
CATATCGCAAGTGATTCGATTTTGTTATTTCGGCTATTCTATTTCGTTGAACATATAACAAAGT
TTGTCAGTTAGTTACAAATTAATACAAAATCGCAAAAAACATGATTAAAATGCAACTTTTTATA
GCCTGAATGGGTAAAAAGTGTGTAATGTATTTGTGCAAATGTATGTAATAGGCAGAAGGAAGCA
TCTCCGACCCTATAAAGTATATATATTCTTGATCAGCATCACAAGCCGAGGCGATCTAGCCATG
TCCGTCTCTCTGTTCGTCCGTCCGAAATTTAGTGTTAGGTCCTCCTAATGCCTGCGCAGATCG

AGTTTGTTTCCGATAAACGATAACTTACTCGATTTGCAAGCAATCGATAAAATGCGATATCGAC
ATTTTTTTTAAATTAATCTTTATTACTACAAACGAGCGCGTTGCGAGAACTGCGTTTGATGTTG
GAATAAGAGGGTTCAGGGTATCCCCTAGTCGGGAGTTCCCGACTAGAACCTCTTACTTGTTAGG
TATGTAACAGACTACATATATAGTACAGACTATTCGGTGCAATAGCAGATCAAGACTGGTGGAA
AAGGCGATTTCTAAGTCTGCGATGCTGCAATAGTTTTAGACTTTCATCGAATGCGATTATAGA
CGAGCATATATTCAATGATCGTTCGATCATTCGAAATGATGGTTCGTACAGACAGATGAACATA
TTGCAAGGTAGACGACCCACAATACTTATATAATAATTTAATAAAATTGCTTCAATAAATACAT
AAGTTCTGTAGGCTATTCAAAGAGTGCCATTTTTTCGTTTTTATACCCTTTGCCGGTTTTAAAT
TGGTTTATTATGTAAAAAATGTACCCTGGTCTATTAAGTCTCGTGTCCTGCCTCTTGTTTGCAG
TAATGCTGCAACCTTACATTTTTGGGACCTTCTAATAACGAAAATTATAAATTTATATGGTTAA
TGTTTTAAGAGACATTTTTTGTTACTTGCCTTACATAAGCCGTCTTTCGGGGTGATTTCATTTA
TGTAACCATACATTTCGCAAATCTGTGGTATGCTTGAAGATTTATAAAACTTGTATAAATATAA
GTATTTGTTGTCCCTGAACCCATTGAAAATGGGTAAAAGGGGTATAATGTATTTGTGCAAATGT
ATGTAACAGGCAGAAGGAAGCATCTCCGATAACACAAAGTATATATATTCTTGATCAGCATCAA
TAGCCGAGTCGATCGAGTCATGTCCGTCTGTTAGTCCGTATATATGAACGCGACGATCTCAGAA
TCTATAAGAACTACAGACTTGAAATTTGAGATGTAGGTGCTCCTTGTTTCCGATAATCGATAAA
ACTGATCCATTTGCAAGCATTCGGAAAAAACCGATATCGTAATCCTGTTATTGAGCAAATTTCT
TAGGTTTTTGAGCTAGATCCACACAAATGTGGCATGTAGCTCCTATAATAATATATACAGATGA
AATATCTTGTATTATACATAAAAAAACTGAAATCATTGTTATATGTGTATAGCATACGAAAAGC
GAAAAGCAACAGGTAATTTTTGGTAGCTCTAGCTCTTAAAAGGTAAGATGTATCTAACGATATG
TTGGAAACACGTAATGATATCGATTATTTGAAAAAAGGCAGGCGGTCGTGGCTACATGGGGTCC
GCTATTGATCCTGATCAGGAATATATATGCGTTATGGAATGCTTTCTTTTGCATTCTTACATAT
ACTTGTTGTTGTATCTGCACAAAACCGTAATACCCTTTTAACCTAACTTTATAATTTTTTGTAA
GGAACACTGTCGCAGGAACAGTTCTTCGTTCTTAATACTGCTTCGTTTTATGATTGTTATTATG
ACTGTTCCCGGTCTGGCGTATGGTTGCCGCAACATTTACGTTTGTTATGTTTTCTAGGTCTCAT
ATCGTGGTAAGCCAATTTTTATGCCCACTTTGTTTAATTGCCCACACGCACACACACGCCCAGA
TGAGCTATCAGGATTTTTATGCTGCCGCCGCTAGTTGCGGTGATATTTTGCGTTTGGGTCACCT
TCGTTAAAGCCATCGCTTACAGGTGTTAATATTTCAATTAGTTAGCGTTTATGTAGCGAAAAAA
CTCTTATGTAATGCTTCACGCAACTGTGACATTGGGGCCAAGTCACAATGGGTGTTCGTGAGTA
CGCTGGTGTGTAGCAAATATTTGTTCCGGCGTGTTAATAAATAATTTTAATTGCCGTCATAAAT
AGAAAAGGTTTGATTAAAATGCCAGATTTACCAAATGTAACATTTAAGCAACCACATAGCAAGC
GCATAATAAGTACATTTTAATTTTCGAACGCCACCAATCATCCTAATAATTCATTTATCAATTG
GTTGAGCCAATGTCACGGTGGCAAACTTGGCTAATTGTGCCTCTTTCACGGCTTCCCAGCTCTC
ATTTCCCTAGAATCAAGAGTCTAAGTCCTAACTTAGAAAGGGTCAGGTATATGAAAAATTCTCT
GAGGTTTGATCTTTTTGGAAATCCTGTGATAATCACTTTCTTTTGTTAGAATGGGAAAGTCGCT
TAGTTCACGACACTTATTGTATTTGAGCTCTGTCTGTGAAAATAGTCAAAAACGACAAAGTAAG
GATATCTTCGGCATGCCGAAGATTTAATGCCCTTATAGACATTTCTCCTTTGCAGATACGTAAA
GAAATATGATAGAAAAGACAGAGTTTGATGAAACAGAATATTTTGCCATATAAGAATTTACATT
TCGACCCATCTTTAACATGGAAGCTATATGATATAGTGGCCCGATGTTAAAAGGATTTAGCAAA
TCGGTCGGGAGCATAGTGAACCTTATAAATGGGAAGTTTGGGTAAGATATCTTGATAAACGAGA
TGTTTTTCATACAAGAACTTACTTTTCGACCGATCGTTCCTATGACAGCTATATGCTAAAGTGG
TCCGATGCTAAAACGATTTTCATATTTTTAGGGCACAAACTGAAACTGGTAAATCCCGGTTTGG
TTATCTCAAATAACCACAAGTAATGGGCCTTAGTCGGGAGTTCCCGACTAGGGGAAAACCTGAA
CCATCTTCTACAAACAACGAATGCAGATAACGCCACGAGCTCGTTTGCTTTGGCAGTAATAAAA
AAACATTCAAATTAGAAACATTCCCTCCCCCTCTATCCCCTATTAGATTGCACAACTTGCTGCC
ACTTCTATTTAACAGCCATCCCAACAATTCGTGCTTCTTAACCGATCTTGATCAAAATTCTCAA
CAGTTGATCTTATTGATCTATAGAATATCCGTATAGTCTCAAGAGCACAATTGCTTTAATATTG
TGGCTTAAATTGGTGGGTAGTCTTAATCGATCTATTTATTTCATTTATAGCTAAGGAATGTAGG
CGCGGGTCGACGATACCTATAAAATGAAAGACTTATTTACTCCTAAAGCGTACGCGCATCGTAG
CGCGAGCTGCATTTTGTGTAGGTAGAAGAGGGCTAAGGGCATTCCCTAGTCGGGCAGAGCCTTC
CTATTTGTTATTTTTAGATTCGTAGCTTAAACTTTTGGCAAAGTAATTTGTATGATTTGTAAAT
TGTGCGATACTTAGTGTATATTATCGCCGAAATGTGTGTTTACACGATCTAAAACATATGTTGC
TTTGGTGGCTCACAGATGTATTAAAAGTGATATTCAAATTAAAGAACGTTATCCAATAGGTAGA
TAAAGTAATATTAATCAAAAAGATTTAAAAAGAAAACATTTTGATTCAAGATTTGATAGTTTCA
ATTGATATAAGCTAGGTTGGCCGATTATCCCACCGTGTTAAGCCCTGTTCCTAAATGCTTTGTG
AAACTAAATTTGACGTGAACTTCATCTCGTTGGTATTCACTTGGTATCCACTTGCGCTCGCCAG

TGACAACCTTCTCCCGTGACGGAAATTTGTGTTTTTCCAAAAGTTGTTTGATGCGAATGTGTAG
ACACGGTAGCTTAAGCAGGCCGTTGAAAAGCACGTACGCATCGAGTGTACCAATTTGTCACGCT
CTGGTCGTAGGCAAAATATTTAAGCCAATCGTAAGAAGTCTTTATTTAATACTTGGTCAATCTA
GTTCTAAAGCTTTTAATATTTTTCGATTGGCCAAAGAAATGCGATCATATAGACAGACGGACCT
GCAGCAAGAGGGAAATCACACGAAATCAATACAGGCTTCTCAGATTATGAAGGAGCATAAATGC
ACAACAATGCACAAGAATTACAAGTATATATGTTCGATATTGCAGCGGCTAATAGTGTATTTTG
AATGTTTTGCCCGGAACTATACGTGATGCATCCCATACGGATACCTCGAAATCTCTTCTACCAA
CATCAAATACAACAAAAATCAAGAAAGTAACGATATCATCAGCATGCCGAAGTTTAATAACCCT
CTAATAATTTTCCTTTCGCAGACATGTGAGAGCAGTGCAGCTCGAAAATGCTGAGTTTGTTAAA
ATCTCTTTGAAAACAGAATATTTTGCTTTTACGATCTTTCCTCTTGTAGGTTTATGATATAGTT
ATTCAATGTAGATAAGGTTTTGCAAATGTGTAGGGAGTATAGAAGAAATCTTGATAAACAAAAA
AGTTTTTTCTTACAAGTACATACTTAATTTTCGAACGCACGTTCCTATGGCAGCTTATGATATA
GTGGTACGATCCGGCTGGCTCCGACATATGTACTGCCTGCAACATAAAGACAGACCTATGCAAA
GTTTTAAGACAATAGCTTTAACACCCAGAAACGGAAAGACGGACACTATATTAACTCGGCTATT
GATGCTGATATATTTGTGCACAAATCACTATACCCTTTTATTCATGAACAGTACAGGCATTTGT
TAGCTTAAAGTCCTTTGAATACAGCACCAAAAGTTGGAATCAACGGTTTACGTGCATGTTTGTC
TTGTGGTGAGAATCTTGAATCCGTAAAATAAGCTTTCGATTGTTAAACCTTTGACCCGCATTTC
CCATCCATGCGCCGTAGATTTCGCTAAAAACATATGAAACAAATTTGCACTAAGATCTTTGAAA
ACTAGTTTGAATAGGATCCGATGGTAAACAAAAATGTTGCAAATTTTCCGCCTAATTTCGAGCC
AGGCTTGGAGTTTCCAAAATCCTGTTAACGTCAAAGGGGTGGGTTTTCCATGAATTTTGGAACA
CACATCAGGTGAATTTGAACATATCTGGAAATGATTTTGAAGCCGGTAATCAAAAAAGTTGCAT
ACAGCCGTTTTGCACGATTCTTTCACATTATGGTGGAACCATCTTAGCGGGTTTCTCCATCACA
TGCGGTAACTACTTTCAGATTCGTAGCTCTTATGGTTTGGGCAGTTCGTCGCGCACCAATCAGT
GAGGACAAACAGTTTTATATAGAGAGATATTTGCTTCACTGCTTACTTTTTTGACAAGGCTATC
ATGCTCTCAACAACATTGCCATAACAACATTATCCCTTGATTGTGAAAGGTTATTCAGATGCTA
TAAAGTGACATAAAGTGTGGTTTTTGGATGGATGTGGTCATTATTGATTGTATGGCTCATTTTT
TGTAAAGTTAAAAACAAAAGAAAAACCGAGAAACTCCAATTGACCATCGGTTTTATAATTTTTT
TTTTTTAAATAAATATTGTTTGTTTGCATCTGTACGTTGGTAAACTAAATGACATTTATTGTCA
GTTTTTGGATCAGCCCGATGCGGATGCAGCTGCAAAAATTTGTCTCTGGCCAAATGTCTTTCCA
TATTTTTCATGCACAATTTCAGGAAGGGGCTTGAAATGGCTATGGAAATGGAAGGTCGCCTGCA
TCCTTTGCCTCAGTCACGGTCTCAGTTCGTTCATATATATACATATATATATATATATATATAT
ATATATATACATACATATATATATAAAATATATAATGTTTATATGTGTATATGTATATTTGTGC
ATTTATTTATTTACGTGGCCAGAGCCAGCCGATGGGGTAGGTCCAGTTCGTTGCAGCTCGTCGT
CAGAGAAAATAAGAATATTGCAATGGCTGACAAATTTATTTCGTAGCCGTTAGCGGTTTTTCTT
TTTTGTCGGCCTTTTTTGCATATGTCAGCCCTGCCATGCATATTAAAATGTTGAAAAACACACA
TTTACCTTTGCATATTCATTATTAAATACTTTTTGGAGACAATTTATAAATATTATAAGCAAAG
GTAAATACATTTAATATTTTTGCGGCTGCTTAAATCTTTAAATTGTGATGAAGTGGTCAGCTTA
AATACTATCTACTATTTTACTTTTAATTTTATTGTCTATATTACCATTATATGAAAAGAAAAGA
CGGCCCAAATCGAAAGTAACCGTCTGAATGACATCAAATACCCAGTTCCGAACTGCCATAATAT
TCATGCTTCTATATACATAGTTTCTTCCTATAAATCTATATAAAATAGAAGTGGGAGCTTGATA
AGACTATATTCAGACATTTTTTTAGAAGAACAGCACATTTATTCGTGGCTTGCCAATATAATGA
CTCGTTTCCTTTAAAAACTTCTGTGTTTCTTAACCGATCTTGATGAAATTTCTTACAGTAGATC
TTTTTGTGCTATAGCATATGCGTATGAATAGAAATAAAACTGTGCCTTTAATTGATGTTTAGTT
CCAATTGATTTATTTTTATTAATTTTGCGTAATGTGGTTGGCGTAGGTGCGACGGTTGTCATAA
AATGAAATGTACTTGATCTGTACATACAGTTTTAGAAGCTTCATTTAAATTTGGAGACTCTGGC
TCTTAAAATCTAAAATATTTTTTCAAAAAACAGAATTTCAATATCGATCTTTATCCATTGCTTT
AAAAAGGTGCAAGTTATCGATGTTTAAAGAAAAAGTCGTGATGCGCGGACTGAGATCGGCGATT
TCATGCAAACCCTATTCGGCACTAACGACTTTAGCGCCCTGATTCTTGATCCTTATTTCGGAAA
ATAAGATTTATTTGTAGCTTAAATCGAACTTGTTTCGCAGTTTTTTAGCTAGCGTCATAAAACA
AAGCAAACACCGAAGACAGTCTTCTGAATTTTACCTTGGCCAACCTTTTTAGCAACTAAGGATT
GGCCAATTTCTGAATGTAGTGTGTATACAAGATATTTTTATTACATATTCAAACGGCTTAAACT
CGGATTTGAAAATGTATGCAAAAGCATTTGAGACGATTTTCAGGAGCTTGGCACTGTAGCTTCA
GCACTTAAGAACTAGTTTGCATGCAACAGAATATATATATATTCTTCATATGTATAAAAAACTAAA
TGGGACTTCGTGCTCCAGCTCAAATTTGCATTGCATCATTGCCTTCGTTAGTTAGCCAAACGTG
AAGTAAACACATTGTGTTCAAAATATTACGTATTTGGGTACATGGAGGAAAAAGGCCATTATGA
CAATTTGTAAGTTGAGATGAATTAAATATGAAGAGAAAAATACTTTAGCGCTGATAAAGCACAC

ACGCTGCAACAAAATGCCAGGGAAGACATACATATGTCCGCGCGTATAACCTTTAAAAACTACA
CAGTTACATAAACAGGATAATAAGGCACTTAACATGCACTCTTCGCCATCGCCACTGATGCCAC
TGCTACAGCTGTTGTCCAACACTATGGCAATGCCGAGTATACCAGGAAAGGCCAGCAAAATATA
AAACTGCAAGTCCCGTTAATGAAGGAGCCAGAGGACAGCAACACTGACGACGATGACGCAATAA
GGACAACAGCGATACAGGAAAACAAAGCTGCATAAAGTCGCTGCCACTTATGTCAGAGGAAATC
AAGCGTTACACTCGACACCATCCCGCTCAGATTACGGAACTCGGGTCATAGAATCCCAGGTGCT
GTCGACTCTAGCTTCAAGCCTTTAGCTACTATATGCTTAAACATTTCCCTTGACTTTTATTTTA
AATGAGCATCATAAGCATAGCTGAAATTTAAACAAACCTTTCGCTAACAACCCATTATCCCAGC
AATCAATAGTTCCCGATCATGGATTTAGATTTTTCATTATAGCGTTCAATAGAATTGACAAGGA
AAGCAGGTTACTCAATCAACTCTTTTCTTGTCCGAGACCTCACACTACCAAACCGTCTTGTCTG
TCTTGTAAAATATGTCTTACGGCTTTGCAGATATGTAAGAATAGTGATGCTCGTCAAGAACTTG
ATTTTCGGTCGACTGTTCATATGGCATACATATGATATATGATGGTTCGGAAGATGATAAAATT
AAAAATACCAAGATATATTGAAAAACAAAAATGTTATTCATGCAATAATTTACTTTTGATATAG
TGTTACGATGTTTAAAAGATTTTGCATATATATGAGAAGCATAGTAAAACTAAAAAATCTTCAG
CTTAGTCAAAATATCTTGATAAACAAAATAAATGTTCATACAAGAACCACATTTCCGACTTATC
GTTCCTATGGCAGCTATATAATATAATGGTCCGATCCAGCCGCTTCCGACATATGTACAGCGCG
TTACAGAAAAAGGGACGTATTCAATCTTTCATGAGATAGCTTTAAAACTGTGCAAGGGCATAAT
AATATTTGAAATTGAATAGTTCTGTGTGTTCACTAAGAATCATCATAAATCAAAAAGCTCGCAT
GTCAAAACAAATAATATTTGTGTGTATCTATGTTGGATGATCGCTTTACTTATCGACTGCGCAT
ATTATATACACAACATGGCCGAGCAAAAGCTCACGTGAATAAAAATAAATTGCATGGATGTTTA
ACGGCTGAGCCTTACAATATGGTTGCACGTCCATGGTTTCTTACATCGGCTGTGCCTTACAGTT
CATAGAGTCAATCGATGTCAGCAGGTCTGTTAACGTTGATGTTGATATTGCAAAATGTGCTTTT
ATGTGTTAAATGTTAAAAATTCAATTATTCCATGTACGTGACGATGCTAGTAATTATTCAGCAT
GATTTCGAAACATGACATAGGGTGATAGAGCTAATATGACCAACTTATTAGGCCTCGCAACTGG
GTTTATCCTTGTGACTTCGGTGTTAAGCCCTGCAAACTGCATAGGGAGTTTAAGGTATTTTTTT
AGGTTTTTGGCAAGGCCTCTCATGGCGACGATGTGAACTTTAGTGGCCCATAACAGGCCCTAGG
CCAGGAAGAGCCACGTGACGGCAAAGGCAAAACACAAGAATTACTGTAGCCATGTAAATTAACT
TGCTTTCATTAAAATTCTATTCCTGGTGTACACTAGTTGACATAAGTATTTTGACGTGAGAAAA
ATTGTGCATTTATAAGTTTAACATTTAAACAGCTTTGTAAGAATTCTTCTTTATTTTCTCATTG
TTATATATGTATAACAAATACAATCTTAAAAAAAAAATAACCAAACAAATGTATTTTCATAGAA
CCGTAAAAAGATATAAATTAATTGGTAATTTCATGTGTTGGCATAAGTATTTTGACAAGAATGT
TATACTAAGAAAACAATGTTTAAGCTTAACTAAAGTACTAAAATATAGAAGTTAGCAAAATGTA
TATCCATCTTTGGTTTCAATATTTTTTTGAAGGCTAAATTTTTAATACCTTTTGGAGATATCTC
TTCTCAAAGAGAAGTGACCTCAGAAATTGTCTCCTCTCTGGTTTTCTCTTGCGGTATATTACGA
GTTCGAGTCCTATTGAGTCCTATTTTCTATTAATTTATTTTTATTTCTGCTAAGACAACTCCCG
ACTAGAGTCTCTTACATGTTATAAGAGTCAATTAGCAAGTAGTTTAGTCGGTTGAATATAGTTG
CTATTGCGTAACCGGCAGTAATTTTTTTATTAATGCTGGTATATTCCATTAGTAGAAGGGGTTC
AGAGTATCCCTTAGTCAGACACTGCATTTTATTTGTTTTTTAAATTTATTTCTTCAGCACTAAA
CATTACTATTTTTCTTCATATTTCATATTTTTATAGATTTCTGAACGCAATAATTTCTTCGCTT
TTGATGTATAAGTATCCGCTCTGAAGTTTCAGACAATAATAAACGGCTTGTTGAAGTTATA~~AGA~~
~~CTCACCGCATTCTTCTAGCTGATGAAAT~~ATGGAATTGCTGCAGTTGCTGTGTTATACATGCCTT
CTACTTCTTTCGAAGATGCTAG

**Proximal breakpoint**

>Nova00_Contig288 45449..41378…(*GJ16378*[+](*CG10932*)- *GJ18714*[-](*CG1354*))

~~TCCAAGATAAACTGGAATCTTTTCTAGCCGGCAACTGAAAACAGAGTATTAACAATTCTGTCGT~~
~~ATTCTTCGTATTAAAGCAACGTAGCATTAAAAACCCAATGACAAGCAAAAATACTAATGCACGA~~
~~AAAGCTTGCAAACAGCGACTTGCCACTTTCCACTTTCCAACACTGTCCGCAACAGCTGCTCACA~~
~~CGCCATCGGCTCGTTACGCAAGTGGATAGCAACAGGTTCTCAGCCTCAATATTATTGCAAGTGT~~
~~GATCAGGTCCGGTTGTAAAATTCCAGTGATCGTTCGATACTAGCAAGCAGTCGGTTATATAAAT~~
~~CTAGTGCC~~ATGTTGCAAGCGACCAGAAGCCAGCTGTGCGCCCAGGCGCGTCGCTACAGCTCAAA
GATCAGCGATGTTGTTGTTGTGTCCGCCGCACGGACACCGATCGGCAGCTTCCAGAGCCAGCTG

GCGCCGCTAACGGCCTCACAGCTGGGCGCCACGGCCATCGAGGCGGCCATTCAGCGTGCCGGCA
TTGGCAAGGGCGATGTTAGCGAGGTGATCATGGGCAATGTTGTGTCCGCCGGACTCGGCCAGGC
GCCGGCACGCCAGGCAGCCATCTTTGCCGGCCTGCCGACCAGTGTGTGCTGCACCACCGTCAAC
AAGGTGTGCTCCTCGGGCATGAAATCGGTGATGCTCGGCGCCCAGTCGCTGATGCTCGGCCAGG
CTGAGATCGTTGTTGCCGGCGGCATGGAGTCCATGTCGAATGTGCCATACTATTTGAAGCGCGG
TGCCACGCCCTACGGCGGTGTCAATTTAACCGATGGCATTGTCTTCGACGGTCTCTGGGATGTC
TACAACAAGTTCCATATGGGCAACTGTGCCGAGAACACCGCCAAGAAGATGCAGGTGACACGCC
AGGAGCAGGATGCCTTTGCCATTGAATCCTACAAGCGTTCGGCCCAGGCCTGGAGCGATAAAAT
CTTCGCCAATGAAATTGCCCCGGTGAAGATTCAGCTTAAGCGCAAGCCGGAGCAGATCATTGCC
GAGGATGAGGAATTCAAGCGCGTCAATTTTGAGAAATTCGCCCAGCTGGCGACGGTGTTCCAAA
AGGAGAATGGCACCGTTACCGCCGGCAATGCCTCGACCCTGAACGATGGCGGCGCCGCTGTCGT
CCTGATGACCGCCGAGGCGGCCAAATGTGCTGGCCTCAAGCCATTGGCGCGCATTGTCGCCTTC
CAGGATGCCGAGACGGATCCCATTGATTTCCCCATTGCGCCAGCATTGGCGGTGCCCAAGCTGC
TGGAGCGTGCCGGTGTCCGCAAAGAGAATGTTGCCATGTGGGAGATCAATGAGGCATTCTCCGT
GGTCGTGCTGGCCAACATACGCAAACTGGACATCGATCCGGCCAAGGTGAATATTCACGGCGGC
GCCGTCTCCCTGGGCCATCCGATTGGCATGTCTGGTGCCCGTCTGGTCACCCATCTGGCGCATG
CCCTCAAAGCCGGCGAACTGGGCTGCGCCTCCATATGCAATGGCGGCGGCGGCGCCTCCTCCAT
TCTGCTCGAGAAGCTCTAA~~GGCGGCGGAAACAGCATGAAAACCAACAACTACAACAAGAGCAAC~~
~~AATTAAAGCCAGAACTGCATTTATTCAAATTTTCAATCGATGACGCCAGTTATCGTTATCGTTA~~
~~TCGAACATTAAAACTGTTTTTTACTCTCATTGTTTCTGAAAAGATGAATTTTTGTCTATTATTT~~
~~GTATAGCATTTAAT~~CGAAAACTTTGTGATTTGCAAAATAAACTAAGAAGAATTGCATAAAAACA
AATGATTTAAATGGTCATCGATGATAGTTGTGATCGGTATCGAATATTTGGAATACGCTTAGCT
TATGTGCGTCAAATTAGCGGCGGAGACTTAGCGTCGAATTTTGGTTCTTGTTATCGGAAAACTA
TCGGTCACTTTCAAATGCATTTATTTATGTTTTTAAGCAGGAAACCAAATAAATTTAAATGCAG
AATTGCAAAAAATTAAATTATTTACTATCTACATTCATTTATTTTTGAATTTAATTTATAATAT
GGGTAAATAAAGGGGTTGAAAAATTAGGTTTTCACCATTCTTTAAATATCTTAATTCAGGGATA
ATGGCCCCGAAAACCGCATATTCACGATTGAAGTCTATAGTTTCACCTAACCAATCTCGAAAAA
ATTATAAAAATCAATCAGCACGTTTTTAAAATATTTGTACTACAGTGCAGCAACCTCGTTGCCA
GCTCCATACAAAATGACCGTACAAAATCACCCTTGCACTCGAACCCTAATAACTTTTCTCAGGG
ATAATGGCGCTCAATGCACGGTATACCAACTTGAAGATACATGTTTAAGGAAGCTTTACGCATT
AATTCTAAACAAATCGATCAGCCAGTTTTTTTATAAAATAGCAAAATGTAAACACACCTCATGT
TGCTCATTCAAAAGGCCTATCGAGCTACTAGCTCCAACGGGAACCGAACCACTGCTCGGAATCA
GTGTTGAACAATGTTATACAGTATTGAGCGGGAACAAATTAATTCAAAATACTTGTATATGGAA
GAGAAATGCAGTAATATATGAAATAATTGCCATTTTACAATAAAACAGTATCTTTGGTAAAATA
GACTGCTGCAAAAGACGACAACTATGCATTTAAGTATTTATACACGTATGATAACGAAAAGGTG
GCAATATTTCAACCTTTATTATTCTATTTTCCGATTTAGATCGCGAATTATCAATCATTTTGCT
TTCTACATCAAGGAAATCGTCCAGCCAGTTTTTAGAAATTCGCATTTTTCTATTTTTCTTATT
ATACATAAATAAATGCATTTGAAAGTGACCGATACTTTTCCGATAACAAGTACCAAAATTCGAC
GCTAAGTCTCTGCCGCTAGTTTGACGCACATCTTTAAATACACAATCGCCTCCGTCAAATCGAA
AGTTCGATGTTTTTAATAATTCATTATCGATAGTTAGCAGCTTTCACTCGCTCATCTCGAAGCG
TTGACGATAGTACAAAACTATCGGAAATTCATCGTCAAGTAGTATCGTTGCTGCACGACACTCG
ACAATCGTGCTACTACCGTGCTATCGGCAACTTTTAATTGCTTATATTTCATAAAATACATTAT
AAAGTTAAGTTCCGCCGCCGGAAATTGAATCTTGAGAAAAAACTAAGTGGAATAGATACCATCC
CCGACTATTTGGACATTCCTTATGTGTGGTGGCTACGACGAATAATAATCGAAATCTACGTGAA
GCGACCAACGTGAAAAATTACCGTAAAGCATTTTTTTTTTAAATTAACTACTTATTTAATGCTG
GATTAGCAAAATAAAATTCAAAA~~CTTTTTGTCATACAAAAATCAAATGCATTTTTACTTGGCAC~~
~~ATGTATGTATGTGAAACGGGCATCTGGCATCAGGAAAAGCAAACAGTTGTGAATATAAAATTAA~~
~~ATGTACGAGATAGTCAACATGATTATCTAAGTGGCCATACGATA~~TCATTTTTTCTTGGCATCCT
TAAGACCAGCGCCGGCGTTGAATTTGAAGAAAATTATATCGCCGTCTTCCACGGTATAGTTGCG
CCCTTGCTGGCGATATTTGCCGGCCGCCTTAGCAGCTACCTCACTGCCCTCCGCTTTAAAATCT
TCGAAGTGCATCACTTCGGCCATAATGAAGCCCTTCTCAAAATCAGTGTGTATACGTCCAGCGG
CCTGTGGCGCCTTTGTGCCCTTTTGGACCGTCCAAGCCTTGACCTCATCGGGGCCGGCAGTAAA
GAAATATTCCAGCTGCAAGGCCTTGTAACCAGTAATAATGATCTTGTCCAGCTGACTTTTGCAT
TTGGTCTCCTCTTCGTAGGCCTTGCGCTCTAAATCATCCTTTTCACTAAGCTGCAGCTCGAATG
CACCTGAGAAGGGTATCAGTAACGCGCCCGGGTCGTTCTTATCAATCCAATCCTTAATCTTGGG

CAGCCATTTGTTTTTTTTGCGGATAAAATCTTTGTCGGAAAGGTTGACCAGATAAATGGCTGGC
TTGGAGGTCAAAAACAAATATTTGTTCAAAGTTTCAATCT
