# Supplementary material for: Origin and Consequences of Chromosomal Inversions in the virilis Group of Drosophila
Source: Genome Biol Evol. 2018 Oct 30;10(12):3152–66. doi: 10.1093/gbe/evy239 (PMC6278893; doi:10.1093/gbe/evy239)
Supplement: Supplementary Data [file evy239_supp.zip › File S5.pdf]

Ancestral state:

D. novamexicana 15010-1031.00

Distal region

>Nova00Contig824:…31,433..33,224…(*GJ23198*[+](*CG11755)* – *GJ10317*[+](*Invadolysin*))

ATGACTGACGATTCGCTCAATCACAGCTTCAACGTCGCCGCCGCAGCCGCCGCCGATGATGACG
ATGACGATTTGAATCTTGACAACGAGGCGGCAGAGATTAGGCGATTGCAGCTGCCGACCTCAAC
GGAGCCTCTGGTACAGGATGATGATGAGCGTGTGGCAAACATCGAGTTCAATTGCAGCGCTTGT
GATATGCACGAGATGGTGCACTTCTATGGTCGGCGCCGCCCTTTGCCCTGGGAGTTAAATTTC
GTGAGGATAGCTATGTGTTACGCGATCCATTCCAGGCACCTCCGCCACGCTGGCAATCCAAGCC
AGAGTTTTATGTGTCGTTGGGCGCTAAGTGCGCTATCTGCGGCCAAGTTGTGTGCAAGGATACG
TCGTGCAGTTTCTATTATACACAGACCTATTGCATGCCTTGTGCCAGGGCCGAACTTAAGTCTT
GGCCCGTTGAGGCGCAAAGTCGGCTACGCAAGCAATTGGCGGCCAAAAAATAG~~TAGCACTGAGA~~
~~CTGAGTCGTATATACACATAGTTTTTAAATATATACATTACGTTACATCGATGTGCCATTTCTT~~
~~GTATATTTTTGCATAAAACAGGACTATGTGGGGTTTGATATTTCCAACTGTTATTTAGCCGCAGA~~
~~AAAGAGCATGTTAACCAACTGTTGACGCATGCCGTGTCTTCTGTTAATTATGAATCGGTTGCCCC~~
~~AAAAAAGAGCATGTTAAGCAACTGTTGACGCATATATCTGTCTCTAC~~GATTAAATACATGCAAT
ATATAACATATCGATAGTTCGATTACTTTTCTTCGACAAACAACATTATCTGCTCATTCATTCA
ATTCGGAAGAGAGCG~~TCGACCAAGTAGGACGCTAAAATCGCCGATTGTGTACTGTTGTGTCTACC~~
~~CCCGTATTTATTGTTTTTGTGTTTGATTATTTTGTTGAATGAAAATGTTTAAATAAGTGTAATG~~
~~TAAAGACTTAAGTTGTTTTTATATTAATCAAATAACAAATGCTTTATCAAACGCAGCCGTCTTTT~~
~~AAAGCAGTTCATGAGAATTTGACATTTACGCGATGTTGCTGTAACAACAACAAAAGCGACTGCA~~
~~GGCAGAAATTGTGGCACTGCAGCGGCGAGTTGGCGTTTCGGCAGTTTGTGGACCTAATTCGCGG~~
~~ACGCGGACGCACCCACCCAAGTCCAAAACCAAAGCCCCAATTCCAATTTGCTGTGTAACTAGGC~~
~~GCGAGTGTGTGTGTGTGTTTGTGTTGAATGTGTGTGCGCGGGCGAGGCAGGACAGGAGCAT~~
~~GGGCTGCCCAGCCGGTTTCGGTGCGCGAGTTAATTGACAAAAAAAAATAAGCAAAAAACAACAA~~
~~ACAAAATAATACAGAGAAAGAAGCATAAAGCGCATGAGCAAATAACGGCTAATAAAGCATGCAA~~
~~CAAAAACAACAATTGAGAAACACTGTAAACAAATGCTTAAATGTTTGTGCATGTACCACACATA~~
~~CACACATATATTTATGCATGCTCCTTGTGTGTGTGCAGGTTTTCAAGTAAAGCAATCCAACAAC~~
~~AGCAACAATCCTCATCATGCATACCAGGTATTGAGTGAGAGGTTCTAGCACATAGTTCTATAAT~~
~~TAGCTCGGAGCTAGCTCAGATCATTAATTCCCACATATACGCTTCAACACGCCCAACCCGTCCC~~
~~GCCCACACATTGCGAGCGCC~~ATGCCACGGTCGGAGCTGCTTCATTTGACCATTCTTGCGTATTG
CTGCTGCTGCTGGCTGTTAGTCAATGCGCACAACTGTCAGCATCAACATCCCAAAGCACACGAG

Proximal region

>Nova00Contig159: …68,229.. 69,175…(*Caf1-55/GJ23199*[-] – *R1b1/GJ10856*[+])

CATTATCGCTGCGATCAACCAT~~TTTAGACCACGCAGCCAAATTATAATTTCACGCAAATTTTTG~~
~~TGCGCGTCGCAAATTCGGCGCCAAAACAACAAAGCGGTGGCGGCAAAAGCGTGCTGTCGGCAA~~
~~AATTTTACAGGCTTTGCGACTTCGTATTAAACAAGCGCTCTAGTTAATAATATAATACTTAAGA~~
~~TAACTCTTAAATTTTTATTATAAAAATTGAAGGTGCTCTTGCACTTTTTGCAACAATACTGTTA~~
~~GCAAAAAGTAAGAAAATTATCTTCCTTTTTTTTTGCAAGCG~~CGTCGGCTGCTAGATGCGGCAGA
GATGTGTAGAAATGTTATCGGTAGGAATGTAAGAAAGTCGATTAGTCCAACACAATTGAATTTT
GGTGTACAATGATTTCAATCGATAGTTTTATTTGTGTATTACATGCATGCATACGTTTCGTATT
TAATCATTTTTCACTTCAAACCTACCTGCTTACAATGCATTGTATGGTTAGTTATAATAGCCTT
CGCCAAATCGAAAATTCGATATTTAAAATGGCTCGTCATCGATAGTTAGCAGCTCTAACTTTCT
AGTTGACAATCGCTCAAAAATATCGGAGATTCATCGCATAGTTTATTTTTGCTGTACGACACT

CGACAATCGTGCCGCCACCGCGCTATCGGCAATTTTTAATTGCTTATATTTATAAAAATACGCA
AACAAGTTAAGCTTCGCCATCGCAAATGGAAACGTGATAAAAACTAA~~CATAAATAGAAACCACC~~
~~CCCCGATTCATTCGGACATTCTTTGTGTGGGGAACTAAAATGAAGAATTACAGCTTTGCATTA~~
~~CATGGAAAAAGCTAGTTTCGATAGTTGTTCGATTCCCAAAAAAATTCAACGGCAAAAGTAGCA~~
~~TGCTGCCTGTGCACGTACAAA~~ATGTTTAATAAATTAGAATTTAACAATTTG

***D. americana* SF12**

Distal region

>SF12Contig517: …10,689..8,916…(*GJ23198*[+](*CG11755*) – *GJ10317*[+](*Invadolysin*))

ATGACTGACGATTCGCTCAATCACAGCTTCAACGTCGCCGCCGCCGCCGACGATGATGATGACG
ATTTGAATCTAGACAACGAGGCGGCAGAGATTAGGCGATTGCAGCTGCCGACCTCAACGGAGCC
TCTGGTACAGGATGATGATGAGCGTGTGGCAAACATCGAGTTCAATTGCAGCGCTTGTGATATG
CACGAGATGGTGCACTTCTATGGTCGGGCGCCGCCCTTTGCCCTGGGAGTTAAATTTCGTGAGG
ATAGCTATGTGTTACGCGATCCATTCCAGGCACCGCCGCCACGCTGGCAATCCAAGCCAGAGTT
TTATGTGTCATTGGGCGCTAAGTGCGCTATCTGCGGCCAAGTTGTGTGCAAGGATACGTCGTGC
AGTTTCTATTATACCAAGACCTATTGCCTGCCTTGTGCCAGGGCCGAACTTAAGTCTTGGCCCG
TTGAGGCGCAAAGTCGACTACGCAAGCAATTGGCGGCCAAAAAATAG~~TAGCACTGAGACTGATT~~
~~CGTATATACACATAGTTTTTAAATATATGCATTACGTTACATCGATGTGCCATTTCTTGTATAT~~
~~TTTTGGATAAAACAGGACTATGTGGGTTTGATATTTCCAACTGTTATTTAGCGCAAAAAAGAG~~
~~CATGTTAACCAACTGTTGACGCATGCGTGTCTTCTCTTAATTATGAATCGGTAGACGCAAAAAA~~
~~GAGCATGTTAAGCAACTGTTGACGCATATATCTGTCTCTAC~~GATGAAATACATGCAATCGATAG
TTCGATTACTTATCTTCGACAAACAACATTATCTGCTCATTCATTCAATTCGGAAGAGAGCG~~TC~~
~~GACCAAGTAGGACGCTAAAATCGCCATTGTGTCACTGTTGTGTCTACCCCCGTATTTATTGTTT~~
~~TTGTGTTTGATTATTTTGTTGAATGAAAATGTTTAAATAAGTGTAATGTAAAGACTTAAGTTGT~~
~~TTTTATATTAATCAATTAACAAATGCTTTATCAAACGCAGCGTCTTTTAAAGCAGTTCATGACA~~
~~ATTTGACATTTACGCGATGTTGCTGTAACAACAACAAAAGCGACTGCAGGCAGAAATTGTGGCA~~
~~CTGCAGCGGCGAGTTGGCGTTTCGGCAGCTTGTGGACCTAATTCGCGGACGCGGACGCACCCAC~~
~~CCAAGTCCAAAACCAAAGCCCCAATTCCAATTTGCTGTGTAACTAGGCGCGAGTGTGTGTGTGT~~
~~CTGTGTGTGTTGAATGTGTGTGCGCGGGCGAGGCAGGGACAGGAGCATGGGCTGCCCAGCCGGT~~
~~TTCGGTGCGCGAGTTAATTGACAAAAAAAATAAGCAAAAAACAACAAACAAAATAAAACAGAGA~~
~~AAGAAGCATAAAGCGCATGAGCAAATAACGGCTAATAAAGCATGCAACAAAAACAACAATTGAG~~
~~AAACACTGTAAACAAATGCTTAAATGTTTGTGCATGTACACACATACACACATATATTTATGCA~~
~~TGCTCCTTGTGTGTGTGCAGGTTTTCAAGTAAAGCAATCCAACAACAGCAACAATCCTCACCAT~~
~~GCATACCAGGTATTGAGTGAGAGGTTCTAGCACATAGTTCTATAATTAGCTCGGAGCTAGCTCA~~
~~GAGCATTAATTCCCACATATACGCTTCAACACGCCCAACCCGTCCCGCCCACACATTGCGAGCG~~
~~CC~~ATGCCACGGTCGGAGCTGCTTCATTTGACCATTCTTGCGTATTGCTGCTGCTGCTGGCTGTT
AGTCAATGCGCACAACTGTCAGCATCAACATCCCAAAGCACACGAG

Proximal region

>SF12_Contig500: …29,592..30,946…**(Caf1-55/GJ23199[-] – Rlb1/GJ10856[+])**

CATTATCGCTGCGATCAACCAT~~TTTAGACCACGCAGCCAAATTATAATTTCACGCAAATTTTTG~~
~~TGCGCGTCGCAAATTCGGGCGCCAAAACAACAAAGCGGTGGCGGCAAAAGCGTGCTGTCGGCAA~~
~~AATTTTACAGGCTTTGCGACTTCGTATTAAACAAGCGCTCTAGTTAATAATATAATACTTAAGA~~
~~TAACACTTAAATTTTTATTATAAAAATTGAAGGTGCTCTTGGACTTTTTGCAACAATACTGTTA~~
~~GCAAAAAGTAAGAAAATTATCTTCCTTTTTTTTTTTTGCAAGCG~~CGTCGGCTGCTAGATGCGAC
AGAGATGTGTAGAAATGTTATCGGTAGGAATGTAAGAAAGTCGATTAGTCCAACACAATTGAAT
TTTGGTGTACAATGATTTCAATCGATATTTTATTTGTTTATTACATGCATGCATACGTTACGTA
TTTAATCATTTTTCCCTTCAAAACTACCTGCTTATAAATCTACTTAGCCAAATAATGTAATGTG

TATCTGTACAACATATATATATGTGGTAGTTTGTATAAAGTGTGATCATTTCTGCACGCATACA
TATGTACATTCGTATTGTTTGGGCATAGAAAGGACGTTAGTGGTATTATTATAATTTTACCTA
AAATGTTCCATGTTCTTAAATGATTTTCAGCATTTTGATGGATTGGCTGCCGGGTATGCTTAAG
ATAAATTGCAAGACATTTGCCAGTATTCGCCTCGATCCGATGCTGCACATCTGTGCTGAGGTTA
GCTTAAGTTCAATTACATTTAGATTTTTATTTAATTATTGAAAACAAAACATTACAAATGGGAA
CTGTAATCTTTGTATTAATTTTTTATACATTTTAAAAACATAATAGCACTAACCATACAATGCA
TTGTATGGTTAGTTATAATAGCCTTCGCCAAATCGAAAATTCCATATTTAAAATGGCTCGTCAT
CGATAGTTAGCAGCTCTCACTTTCTAGTTGGCAATCGCTCAAAAATATCGGAGATTCATCGCAT
AGTTTTATTTTTGCTGTACGACACTCGATAATCGTGCCGCCACCGCGCTATCGGCAATTTTTAA
TTGCTTATATTTATAAAAATACGCAATCACGTTAAGCTTCGCCATCGCAAATGGAAACGTGATA
AAAACTAA~~GATAAATAGAAACCACCCCCGATTCATTCGGACATTCTTTGTGTGGGGAACTAAA~~
~~ATGAAGAATTACAGCTTTGGATTACATGTAAAAAGCTAGTTTCGATAGTTGTTCGATTCCCAAA~~
~~AAAATTCAACGGGCAAAAGTAGCATGCTGCCTGTGCACGTACAAA~~ATGTTCAATAAATTAGAAT
TTAACAATTTG

2a inversion:

D. virilis

Distal breakpoint:

>Dvir_scaffold 13047: 7,909,966..7,907,534 (*GJ23198*[+](*CG11755*) – *GJ23199*[+](*Caf1-55*))

ATGACTGACGATTCGCTCGGCAACAGCTTCAATGTCGCCGCCGCCGCTGATGATGATGATGATT
TAAATCTTGACAACGAGGCGGCAGAGATTAGGCGATTGCAGCTGCCGACTTCAACGGAGCCTCT
GGCACAGGATGATGATGAGCGTGTTGCGAACATCGAGTTCAAGTGCAGCTCTTGTGATATGCAC
GAGATGGTGCATTTCTATGGTCGGGCGCCGCCCTTTGCCCTGGGTGTCAAATTTCGTGAGGATA
GCTATGTTTTACGCGATCCATTCCAGGCACCTCCGCCACGTTGGCAATCCAAGCCAGAGTTTTA
TGTATCATTGGGCGCCAAGTGCGCTATTTGCGGCCAAGTTGTGTGCAAGGATACGTCCTGCAGT
TTCTATTATACAAAGACACATTGCCTGCCTTGTGCCAGGGCCGAACTTAAGACTTGGCCCGTTG
AGGCGCAAAGTCGGCTACGCAAGCAATTGGCGGCCAAAAAATAG~~TAGCACTGCCACTGATTCGA~~
~~TTATGCACATAGTTTTTAAATATACTCATTACGTTACTTGTTATTTTTTGTCTATTGGATAAAA~~
~~CGGGACTTCCAACAACTGCTATTTAGCCGCAGAAAACAACATGTTAACCAACTGTTAACGCCGTGC~~
~~GTGTCCTGTAATTTAGGCCGTCGGTAGGCCCAAAAATGTTAACGCATATATCTGTCTCTAC~~GATT
AAATACATGCAATACATAACATATCGATAGTTCGATTATTTATTTTCGACAAACACGTGCAAAA
TGATTGATAAGTGGTGTGGTGCTCTATTTGCACAACTACCGTACATCAAATTGATATGCCTCCC
GCCCAATTCTGTACAAAATTATTCAACACTGATTCCAGGCAGTGATTCAGTTCACGTAGGAACT
AGCAGTTCGATAGACTTTATGAATTAACTATGTGAGAATGGTTTAAAATTCATTTATTTTTACA
AAATCTGTACCGTCCATTTACTTAAAATTAGTGACTAAAGCTTCTTAAAATATGTATCTACAAG
AGGGTATAGCGTGCATTGAGGGCCATTATCTCCAAGCCAAGAAAATAGGGCTCAAAAACAAGGT
TGATTTTTTCCGTTCATTTTTTATGGGGCTGGCAAGGGGAATGCTGCATTTTTTTCCAAATATC
TAAAAGATGGTACCGACGATTTTAATCAAATTTTCACAGTTGGCTAGGCGAAACTATAGGCTTT
AATCGTGTATATGCGGTTTTCGGGGCCATTATCCCTAAATTTAGATATTTATGGAATGGTGGAA
AACTAATATATCAACCCCTTGCTTCACACATATTATATATTAAAATCCAAAAACAAATGAATGT
AGGCACTAAATAATTTAATTTTTTACAATTCTGCATTTAAATTTATTTGGTTGCGTGCTTAAAA
ACATAAATAAAAGCATTTAAAAGTGACCGATACTTTTCCGATAACAAGTACCAAAATTCGACGC
TAAGTCATCGCCGCTAATTTGACGCACATGCCTCTGTCCACTGGTTTAAGAAACGTCACATTTT
CAAATTTCACATATTGGTGTTCCACCGGTTGGCGGTAAAAGCTGATCGATTTTCTTTAATTTAC
ATAAGCGCCGTAAGTTCTTCTTAAAAAACTTCAAAATTTCACTCTCGAAGTAAGGGCTCCATTT
TAGGTACTCTTTTTGAGATATCTCCATTTTTAAATTCTTAAAGCGGTGGGATAATTCTAATTTT
AAATTTCTTATTTAAAGTTGCCGCTTGCCTTCGTTGATATATCAGCTGTTTGTGTTTACGATAC
TTTACCATTTTTCACAGCATCGATAAAAATCTCGATTAATCAAATCACGAAATGTTCGGGAAAA
TTTATTCATTTTTAAAAAATGTGCCTTTTTTAAAAGGTGTGTGAAATGTGCTTTCTTATGGGGA
AATGCACAGGGCGAATTTCACACATTTTGCAAAAATGTGGCAATTTCTGAAATGTGTGAGAAAC

TGTGTTCAGTGGACAGAGGCTATATAATAAATAAAAAAAACTATCGATTAAAATATTTATATAC
CAAAATTCAATTGTGTTAGACTAATCGACTTTCTTACATTTCTACCGATAACATTTCTAAGCAT
CTCTGTCGCATCTAGCAGCCGACG~~CGCTTGCAAAAAAAGCGGATATAATTTTCTTACTTTTTGCT~~
~~AACAGTATTGTTGCAAAAAGTCCAAGAGCACCTTCAATTTTTATAATAAAAATTTAAGTGTTAT~~
~~TTTAAGTATTATATAATTAACTAGAGCGCTTGCTTAATACGAAGTCGCAAAGCCTGTAAAATTT~~
~~TGCCGACTGAGCACGCTTTTGCCGCCGCCGCTTTGTTGATTTGGCGCCCGAATTTGCGACGCGC~~
~~ACAAAAATTTGCGTGAAATTATAATTTGTCTGCTTGGTCTAAA~~ATGGTTGATCGCAGCGATAAT
G

Proximal breakpoint

>Dvir_scaffold_12855: 4,857,794..4,860,333 (*GJ10317*[-] (*Invadolysin*) – *GJ10856*[+](*Rlb1*)

CTCGTGTGCTTTGGGATGTTGATGCTGACAGTTGTGCGCATTGACTAACAGCCAGCAGCAGCAG
CAATACGCAAGAATGGTCAAATGAAGCAGCTTCGACCGTGGCAT~~GGCGTTCGCAATGTGTGGGC~~
~~GGGACGGGTTGGGCGTGTTGAAGCGTATATGTGGAATTAATACCCTGAGCTATCTCCGAGCTA~~
~~ATTATAGAACTGGGTGCTAGAACCTCTCACTCAATACCTGGTATGCATGGTGAGGATTGAAAAC~~
~~CTGCACACACACACAAGGAGCATGCATAAATATACTTATATATGTGTGTGTGTGTACATGCA~~
~~TAAAGATTTAAGCATTTGTTTACAGCGTTTCTCAATTGTTGTTTTTGTTGCATGCTTTATTAGC~~
~~CGTTATTTGCTCATGCGCTTTATGCTTCTTTCTCTGTATTATTTTGTTTGTTGGTTTTTGCTTA~~
~~TTTCTTTTTTTTTTTGTCAATTAACTCGCGCACCGAAACCGGCTGGGCAGCCCATGCTCCTGTC~~
~~CCTGCCTCGCCCGCGCACACACATTCAACACACACACACACACATACACTCGCGCCTAGTTACA~~
~~CAGCAAATTGGAATTGGGGCTTTGGTTTTGTACTTGGGTGGGTGCGTCCGCGTCCGCGAATTAG~~
~~GTCCACAAGCTGCCGAAACGCCAACTCGCCGCTGCAATGCCACAATTTCTGCCTGCAGTCGCTT~~
~~TTGTTGTTGTTACAGCAACATCGCGTAAATGTCAAATTCTCATGAACTGCTTTCAAAGACGCTG~~
~~CGTTTGATAAAGCATTTGTTGATGAATTAATAAAGTAAACTACTTAAATTATAACGTTACACTT~~
~~ATTTAAACATTTTCATTCAACATAATAATCAAACACAAAAACAATAAACACGGGGGTAATCAAA~~
~~GCAGCGACAGTCTCGCGATTCTAGCGTCCAGCTTGGTCGA~~CGCTCTCTTCCGTATTGAATGAAT
GTGCGTCAAACTAGCGGCGGTGACTTAGCGTCGAATTTTGGTACTTGTTATCGGAAAAGTATCG
GTCACTTTTAAATGCGATTATTTGGTTTTAAGCACGAAACCAAATAAATTAAATTGCAGAAATG
CAACAATTAAATTATTTACTGCCTACATTCATTTGTTTTTGAATTTTAATTTATAATAAAGGTA
AACCAAGGTGTTGAGATATTAGGTTTCCAGCATTCTTTAAATATCTCAATTTAGGTATAATGCC
CCCGAAAACCGCAAATACACGATTGAAGTCTATAGTTTTACCTAACCAATCTTGAAAAAATTAA
TTAAATCGCTCAGCATGTTTTTAAGACATTTTTACAACAGTGCAGCAACCACGTTGCCAGTCGT
AAAATATTACCCTTGCACTCGAACCTTAATATCTTTTCTCAAGGATAATGGCCCTCAATGCACG
GTATACCAACTTGAAGATACATGTTTAAGGAAGCTTTGCGCATTAATTCTAAGCAAATCGAGCA
ACCAGTTTTTGAAAAAATGACAATATGTAAACACACCTCTTGTTCATTCAAAAGGTCTATCGAG
CTACTAGCTCCAACGGTACCGAACCACTGCTCGAAATTAGTGTTGAACAATGATTTACAGTATT
GAGCGGGAACAAATTAATTCAAAATAAGAATTGTATATGAAAATGAACTGCAGTAATATATGAA
ATAATTGTCATTTTACAATAAAGCAATATCTGCGTAAAAAGGCGTAAAGCTTCCTTAAACATGT
ATCTTCAAGTTGGTATACTGTGCGTTGAGGGCCATTATCCCTGAGTAAAGATATTAAGGTTCAA
GTGCAAGGGTAACTTCTTACGGTCATTTTGTATGGGGGGGCTAGCAACGTGGTTGCTGCACTGT
TGTACAAATATCTTTAAAACGTGCTGATCGATTTTAATAATTCTTTCAAGATTGGTTAGGTAAA
ACTGTAGACTTCAATCGTGTATTTGCGGTTTTCGGGGGCATTATCCCTAAATTAAGACATTTAA
AGAATGGTGGGAACCTAATATTTCAACCCCATGGTTTACCCATATTATAAATTAAAATTCAAAA
ACAAATGATTGTAGGCAGTAAATAATTTAATTTTTTACAATTCTGCATTTAAATTTATTTGGTT
TCATGCTTAAAAACATAAATAATTGCATTTAAAAGTGACCGATACTTTTCCGATAACAGTAACA
AAATTCGATACTAATTCATCGCCGCTAATTTGACGCACATTTTGGTGTACGATATTCAACAATC
GTGCCGCCGCTGCGTTATCGGCAACTCTTTATTGCTTATATTTAGAAAAATACGCAAGCAAGTA
AAGCTTCGGCATTGGAAATGGAAACGTGATATAAACTAA~~GATAAATAGAAACTACTCCCGAGTC~~
~~ATTCGGACATTCTTTGTGTGGAAGAACTAACATGAAGAATTACCGCTTTGGATTACATGTTAAA~~
~~AGCTAGTTTCGATAGTTGTTCGATTCCCAAAAATAAATTCAACGGGCAGAAGTAGCATGCTGTC~~
~~TGTGCACGTACAAA~~ATGTTCAACAAATTAGAATTTAACAATTTG
