# Supplementary material for: Origin and Consequences of Chromosomal Inversions in the virilis Group of Drosophila
Source: Genome Biol Evol. 2018 Oct 30;10(12):3152–66. doi: 10.1093/gbe/evy239 (PMC6278893; doi:10.1093/gbe/evy239)
Supplement: Supplementary Data [file evy239_supp.zip › File S6.pdf]

# Ancestral state:

## *D. virilis*

### Distal region

>Dvir_scaffold_12822: 770,553..771,735 (Dvir ncRNA *GJ26151*[+] – *GJ14327*[-](*CG7903*))

~~CAGGACAAAAAAATGGAGGAACTCAAGCTTTTATTGGAGAACCGCAAGCAAGTTTGGAGGAACA~~
~~CAAGCTTGAATTTGAGAAAGAAAAGTTTAAGAAAGAAGAAGGATTGGAACAAAGAAGGTTGAAT~~
~~CTTGAGCCATACAAGATACTTTTCGAGAAACGTCAATTCGAAATAAAAAACAATAAATCGTTAT~~
~~TTGACCTCAACCGTTTTGAGGATCACTTAAAATGGACATAAGACTGAATTCAACCTCTGTATCT~~
~~CCATTACAGGTTGCTCTCATTTGTGTCTCTTGACCTTATAATAAAATGTGAAGTGGTCAAATCG~~
~~A~~TGATTTTATTTTTCTGTGATTGGCCATTAATTAGTATTGATAGTTTTCTTTTATGACGTCACG
TTCGATAGTGCCACCAGCGATAATATAAATACTATCGAATTTTGATAGAACTATCGATTTCCTA
CATTTTTGAATTAAGATTGTTTATCTACAGATATTATAAACATGAAAAGATGTACCTGATTATA
TTTAACAGGGTATCCTAAATGGCATCTACCTCAAATTGGCAATTGTATAAAAACTCAAAGCAAC
~~CAAGTTAGCAATTAATATTTGTATTGATAATATCGTAAGTCGTCAAAAATAGGAAGAACATGT~~
~~CTCACTAGACGTTCGTGTTGTCAAACTTTCTATTTGCACATTGCTCAAATGGATAAATACTTTT~~
~~CATACTTAATGAATACAATTAACAAATATACTGTTGATTGGTTGGCCTTAATATAACGCCTATT~~
~~GTTATTGAACATTTGTTTCAAACTGTTTGCCTTAACTTAAGATGTTGCATGGCCGCAATTATTG~~
~~GAAAACAAAATTCGGCTATTAGGTCAGCAATCTACATAGATTACACCGGCGTCTAGGTCCATG~~
~~ATAAAATGTTACATACAGCATGTTGTTGGCA~~TTAAAATCTTCTATTGCCGCTCATATTATGCGC
TGGCCTGGGTCCATTGAAC

### Proximal region

**>Dvir_ scaffold_13047: 17,183,527..17,177,270 (*GJ22656*[+](*5-HT2A*) – *GJ24596*[-](*CG9775*))**

TTCGGGCCGCCCGTTACGCTATCGCTCCGTAACCGAAGGACGCAGCGCCCTGAGCTTGTGCGCC
CCCTCGGCACTGCCGATGGCCATCTCATTTCAGGGGGCTCCACTAATGACGCCATCCACGGCTA
ATGCGACGCCGCTCAGCGAGTTCCGGGGCAGTTATAGCATCAACGATGACGAGTGA~~TGAGCGGA~~
~~ACGGAACAAGTCGGGCAAATACGAATTTATGGGCTATGTTGGTAAGAAAGTGCAAGTAATCGAG~~
~~TAGACGGATGTGGAATTGATAGCCAGATGAAATTATTGAGCAAACTTCAAACTGTACTTTAAGC~~
~~ATGTAATCAAATGTAAACTACAGGGTGATTTATTCGAAATCAAACCAATGGCGTATAAATAATC~~
~~GCTGACTTCTTACAATTGTAGCAACTGTACCTAGAAAAAGAAACGCTAAACTTCTTAGTTAACT~~
~~TCATCATATCATCAGACAGCAGGAAAGTGGAATATATTGTGTGTGTTAGCAATGCCTGACTGTA~~
~~CCTGTCATATTTCATGCAATCACATTATAGCTTTTAGTCAACTGTACTTACTAACTTAGCATTT~~
~~CAGAAACA~~TCTAATTACAGATTACATATACATATATATATATATATATATATTTTGAAAACTAT
TTAAAACTAGCATTAATGTCTGTAAGTGTTTGTTATTAACCGTAGCTGTGCTTATTAGTCACTT
GAATTAGTTATGTGTTAAACCTAATTGTAATTTAAAGAATTTTAAACTAAATGTGAAATTTACT
TTTACTGATAAGAATTTCCAAACTGTCTTGACACCGCAATGTCGAGTATTCCAAAGTCACGGCA
GCCAATTTCTAATTTTATTTTATGTGTGTAGCAGCTAGCCTCTAAGAATAACTGTATTGTCATG
TGTCAAAATATGATAAGAGATATGGTAAATATATATAAATAAAGTGCATACCTTTCAAGGCAGC
GTCCTTCTTTCTAAAATTTTAAAAAGACATTTTATTTAAATTACTTACATCTAACGAAGTGAGT
GGAAAAACTTGTCAAAAAAATGGTGGGATGCTTTCATTTTCTTGGCGTGAAATTTGTACAAGA
ATTTCTAATCTAATATAAACGCTCCGGCGCTGACAAGAACTGTACTCTGCCTATGAAAAAAAGT
TATCTAAAAAAATAAAGTGCAGCTGGAAAATGTTTGGCTTTTTGAACAAAGTTGAAAGTTTAAT
TTATGTGTTGTAATAAGTATATTTCAAAAATATTCCAAATATAAAATACGTACTGCAAACATAT
ATAGTATGTATATATGGCAATCAGATGTGTTGTTTACTTTTACCGGAACCCGCTTACAATTGAG
AGTGACAATGATTGTGGCAGAGCCACACCATATGTCACGCTTTAGGCACATATAATCAAGATCT
TTGAGCATTAGGCAGACTCTGTGTCAGTCACTCTCGCACCCATAAACCGCATGCAACTGCTGTC
TGTGCCGTTTACTTTGCAGCCCGACAGCAAACTGTTTCCGACAGCCGATGGCATTGCAGCCCAC
ATGCACATTTGGCGACTTCCTTGCTGTTCCGTCAATGCGTGCAGGGTAAGTAATCGGAGCAGGA

GCGGGTCGTGCGTGCAATGCGCGTGGAGGTAATGAAAATTGCATTATTGCCCTGAGCTTAATGC
AATTTAAATGCTTTTATGATCAGAGCACCAAAAGTTTTTCTTATTCTACATTAAGTGCAAAATA
TAATGCACCGAAAATTGGACAGATCGACAGCGCAGCAGCTGCGAGACGAACACACTCAGTAAGC
ACTTCGAGTTGTCGAGTCCTGCAGTAATGAAAATGAAAAAATTGTGTTGCATATCTCGTGGGGC
GTGCCGAAGGCGCTGAGCTGTTGACTAAGCGCTAAAGCGACGACGGCCGCAAAGTGCACGACAA
AATGGCGTCGCCAGGCGACGAAAACAACAACAACAAAGTTGTTGCCGACAATTATATTGCTTGG
GCAGCAGCAGACAAAAAGAATGTGCAAGTGCGAACGGGCCAGAGTAAGATGAAGGCAAGTATGT
ATGTAAGTAGAGAAAATGTGTGGAAGCGTGAAATTGATTGCCCCGCTGGCATTCGCTCAGACCT
CATCCTTAAACTGCTTGCAGTTTATTTGCTTTGGCTTTGGCTCCGGCTAATGGCTGCCCTGGGA
ATACGATTTTTAATTTGCAGACACCTTCAAGTCGCCAACGGAAGTGAATCTTTAACATATCAAC
AGCCAAGCATTTGCATCTGCAGACAATGGTGCGAAATAAAAACACATCCGCAAATAAAAGGGGA
AACAAGCGCTCATCGCTGAATTGTTCACATTTGCACATTTTCATAAGAAAAGCTCATTACCTGC
CGGATTTGGCTTATTTATTATGTGACACACACCCGCAAAACACACACAGTCACACACACACAGT
CATGCAGGGAATTGGCATTCAACACTTTGACGCTTCATTAGCTTTGAGTCTGGCGGGTTGATGT
GGGCCAAGCGTGAATAAAATACAAATGCGATTTGTGAAGGCATTTTCCTCACATACGATTTGCC
ATTTATTTTCAAATTGATTTCATCACGATTGCTATTTTTGTATATTTTTGCAATTGGGACTGCC
AAAACACTCTAGAATAAGCATTTATATAATATAAAAGTTGAAAAATGTAGCATTCAAAAACATA
TTATTAAAAATAGTTTTTCTTTCATCACTTTGAAAAAAGACTTTTATCTTCATAATACAACTGA
ATTTATAATATATTTAATATTATATAATTAATATGGAATCGTCTCGTGCACTTGAAATAACCAT
GTTCAAAATTTCCAATCCTTAATCCTTAATCTTAGATCAACTCGGCTATTCATCCTTATCCAGA
AATAAATAATCTTTGGAATCGGTTAAGCCTCCTATTCCTGACACATCCATTTGAAACAACCATG
ATACCCGCTTGACCTCTTTTCTAAGGGTTCATTGCACTAACACGTAAAAACATAATTGCACTTT
CAAATGGAAATTTTAGATAAATAAATATATTTGCCTATATTTGCCTGACTAAAGTAACAATGAA
TTACATAAAAACAATAATGAAAATGCAAATAAATATTGTAATAATAATATAAAATATTCATAAT
AAACAATTTGAATAATAATGAAACAAATGACAACAATACACTTTGTTAGCTGCGGATGACTTTC
TGGCTTATCCAAAATTGAAAATAAAAACAATGTTATATCTGTATTTGTGTGCATGTGTGTTTGT
GTGCACACTAATGCGATGTGACTGCTAGTTGAGCATCAGCCTGCAATTATTGTTCATTTTGTTA
CTGCTGTTGTATTGTTTTTGCACCCACTGTGTTTTTGTTTGCTTACACCATACACCACGCGTGT
CCCTTAGCTGAGAAGTCGACGCAGCCATGCATGTAATGGATAAAAGCATCTGTCGGAAGCGCTA
TATTAGCACTCGTCCTTTGTTTCAGTAATTAGCCACGGGTTGCTACTAACAATTGATATTTTAT
TCTCAAATAAGTACATCTTCCTTAAGGCCACGCGTAGATTAAGGCCGCAGGTTCTTGACTGCCG
CTGCAGCATTTGGCCTATAATCCGCAAACGCAAAATGGACCTAAACCTGGGCCTTTTTCACCTG
CTAACCGACAGGCCACCGCGTTTTAGGCAGCCCCCTTTGTTCACAATCCACTTTTGGTGGCAGA
TTAGTCAAATTGGTTTCTCGGCTGCCTTTTCACTGCGTTTTTAACTGGCACCTGATGGCCAAGT
TATGTGCTCTAAGCCGCAAGGTATTACACCACTCTCAGCGAACGGCAATGCCGGCAGCGCCGAT
AAATTGAAGTCTTGGCAAAAAGGTGTGACTGTAATTCTGTAGACGACAATTATGGAAATCAAAT
CAAAGCGGCGACACAAAAAGTTTGATTTGTAAGGAGTCAAAAAGAAATGTATGTACTGGAATTA
AATTTTAATTTAAAGTCTGCCTATTTACATTACCTTAACTTAAAGCAAGGAAATACAGGGACAT
ATGGAGTACCAGACAGACGTTAAAACCCTTCACAGTTGCTCATATTTCGCAGTGAACTCAATTT
CAGGACCTTAAATATAACCTATTGATAACCTCCTACATAATTCTGAGAAAACATTGTACCGCTT
CCTTACAATGTAATTATTGCCATTACTACATAAATTAAAGCTTTTGTTCGCTAAGATCTGGGCA
CAACACTGGGAACTTACCCTTATGCGGAATTCATTTCCCTTTTGTCGGCAATATCATTAGCAAC
ATTTTGTGCTTGCCGTCCAGAGAGACGACGGAACCCTTTTGGGAACCAAAAAGGTGGGCACATA
AATTGAATTCTCTAATTATGCAGCAACGTGTCGGCCGGCAACATTTGCTAATTGCTTATAAATT
GTTGAATTGTTTAATGTTTTTAACAGCTGACTGGCAAAAAGGGGTTAATGACGAGCATCCCACG
GGGACGATAACTTCAGCTAGGACACATTTTGGGCGGGAATTCACGCCGTGCCAGGCAATTAAGA
GGCGCAACAAATGGTCATTCTGTGTTAATTAGCCGCCTGCCTAGTAGCTGCCGGTTGTTCCTGG
TGATTAGTTACAGCCATCGCATATTGATGCCAGCTAATCGCATTGGTTGCCGAAACATGTTTAG
CTACTTAGTAGCCGGCAGGCTGATTGACCGAGCGATGCGGCCTAATGAAATGGCACATGACTGC
CGCAATGTTGCCGCAACTATCCAAGTCGGATCCTTGAGGTGGCGCACACAGACGGAAATTAATT
AGAATGACAAAACCTAATTTTCGCCCAACAACTCAAACAAACCAATGTCAACACCTTTTTGGCT
GCCACTCGTTCGTAAGCAACACGAAAACACACATGACCGCGTTGAGCCTGTCTTCGAATCTAGA
GCTAGGCAATCAATCACAGAAATTAAAAATAATCGCTTAAAGGAAAAACTAGACTGCTGAACT
GAAAGAACACCGAAAAGAAAATCTAGTTAAGGCGTAAGCACGCTGAAATAGTAAGTCTTATACC
CTGTATTTATGTTGCAAGTCGAGTAGAACACATTGGTAATGCTTGCCATATTAATACATCTACT
GTAAAAACTAGTATTGCACTGGGAAAATCAAAAGTAGTTATTACGATAATAAAAGTATTTATCT

TACAAAATTAAAATATACATAATATATTTTATTTGCTATAAGAAAAGTCGTAACCAATACAGTA
AAGCAAAAGCTCCATACCAATACAGAAGAAACATTTTTCATTTTAACGCACCTCACTTTTTTTA
CAAGCATCCATTTCACCTTATTGAAAAATAGGTGTTAATTTGAAAATATAGAAAAAATGCTATA
ATAAATTTCCCTACTTGATCCCAAATACCATAAAATCCTGCCATAAACTGTTATATTGTGTATG
CTCGAGCTACAGAATTAAAAAAGAACTACCGAGTACCGATATTATATATATAAACATCGATTAT
TTAATTATTAATTTATGACGCCCAAAATAAAATAGCATGGCTTTTATTCGATTTTGTAAATATA
TGCCCTTTTATTTTGTGCTACAGGGCAAAAGAAGCAAACACGTGAGCTAAAAAATCGACAACAA
ATAAATACATTTATCTTCATACGCCCCAAGGGATGTACAGAAAGTTGTTTGGCTTTTGTTTTTT
GCAGTTCGTCCACTTTATTTGCTTCCTCTCGCAGGTTGGCCAGACATAAGTAAACACAATCGCA
CAAAAGTCAATTTCCGGCACTTGGCAAGAACCCGTGTATATACTGAAAAAAACGGAATGCACAC
ACACACACACACTCGCTAGCCAAATAATTCACAAATACACACACATATTGATAGACGCACACAC
CCGCAGCATGTAGTTTGAACAAGGAGTCAATTGTATTTTGTTGCATATTATACAAATATTTCGA
TTGTAA~~TTATGCAAACAAAGTTATTTACTTATAGGTTATGCTATTTACAGAATGCCTCATTAGC~~
~~GCGAACTAAAGAGCATAAACACACCTTTCCGGTAGTATTTGTTATTTGGACGCTTAAAGTTAA~~
~~TTAGTATTAAGACACAACTTAAAAATTAGTAAAAACTGCCTTGCGGTGGTAATTCTCCTTGATC~~
~~GTCGCGTCCTGCC~~TTACTTTAGCAAAAAGTCCATTTCTGATTTTGTGGTTGACATGTACTGATC
CAGCTCATTGTCCAAATCCTCGCGCTTTACTTCCCTACGCTGCGGCTTAC

## ***D. americana*** **SF12**

### Distal region

>SF12_Contig2221: (…2362.. 1088…)(Dvir ncRNA *GJ26151*[+] – *GJ14327*[-](*CG7903*))

~~CAGGACAAAAAAATGGAGGAACTCAAGCTTATATTGGAGAACCGCAAGCTCAGATTGGACGAAG~~
~~ACAAGTTTGAATTTGAGAAAGAAAAGTTTAAGAAAGAACTAAGATTGGAAGAAAGAAGCTTGAA~~
~~TCTTGAGCGATACAAGATAATTTTCGAGAAACGTCAATTCGAAATAAAGAACAATAAAGTGTTA~~
~~TTTGACCTCAGCCATTTTGAGAATCAGTTATAATAGACACAAGACTGGATTCAACCTCTGTATC~~
~~TCCATAACAGGTTGCTCTCAATTGTGTCTCTTGATCTTCTAATAAAATGTGAAGTGGTCAAATC~~
~~GA~~TGATTTTATTTTTCTGTGATTGGAAATAATTAATATTGATAGTTTTCTTTTATGACGTCACT
TTCGATAGTGCCACCAGCGATAATATAAATACTATCGAATTTAGATAGAACTATCGATTTTTTT
TTGGACGTTAACGTCCGTCATTGTACACATCGTAATTTCCTAAATTTTTGAATTAAGTTTGTTT
ATCTATAGATATTATAAACATGAAAAAATGTACCTGATTCACACAACGGATTCCACGCACAATC
AACAAGTCCAATTTCATTGTGTCCTTATGTTTAACAGGGTATCCTAAATGGCATCTACCTCAAA
TTAGCAATTGTATAAAAACTCAAAGCAAC~~CAAGTTAGCAATTAATATTTGTATTGATAATATCG~~
~~CAAGTCGTCAAAAATAGGGAAGAACATATCGCACTAGACGTTCGTGTTGTCAAACTTTCTATTT~~
~~GCACATTGCTCAAATGGATAAATACTTTTCATACTTAATGAATACAATTAACAAATATACTGTT~~
~~GATTGGTTGGGCTTAATATAACGCGTATTGTTATTAAAAATTTGTTTCAAACTGTTTGCCTTAA~~
~~CTTAAGATGTTGCATGGGCGCAATTATTGGAAAACAAAATTCGGCTATTAGGTCAGCAATCCAC~~
~~ATAGATTACACCGGCGTCTAGGGTCCATGATAAAATATTACATGCAGCATGTTGTTGGCA~~TTAA
AATCTTCTATTGCCGCTCATATTATGCGCTGGCCTGGGTCCATTGAAC

### Proximal region

>SF12_Contig777: …16,346..10,074… (*GJ22656*[+](*5-HT2A*) – *GJ24596*[-](*CG9775*))

TTCGGGCCGCCCGTTACGCTATCGCTCCGTAACCGAAGGACGCAGCGCCCTGAGCCTGTGCGCC
CCCTCGGCACTGCCGATGGCCATCTCGTTTCAGGGGGCTCCACTAATGACGCCATCCACGGCTA
ATGCGACGCCGCTCAGCGAGTTCCGGGGCAGTTATAGCATCAACGATGACGAGTGA~~TGAGCTGA~~
~~GCGGAGCAAGTCGGGCAAATACGAATTTATGGGCTATGTTGGTAAGAAAGTGCAAGTAATCGAG~~
~~TAGACGGATGTGGAATTGATAGCCAGATGAAATTATTGAGCAAACTTCAAGCTGTACTTTAAGC~~
~~ATGTAATCAAATGTAAACTACAGGGTGATTTATTCAAAATCAAACCAATGGCGATTAAACATTC~~
~~GCTGACTTCTTACAATTGTAGCAACTGTACCTAGAAAAAGAAACGCTAAACTTCTTAGTTAACT~~

~~TCATCATATCATCAGACAGCAGGAAAGAGGAATATATTGTGTGTGTTAGTAAGGCCGTGACTGTA~~
~~CCTGTCATATTTCATGCAATCACATTATAGCTTTTAGTCAACTGTACTTACTATCTTAGCATTT~~
~~CAGAAACT~~TCTAATTACAGATTACATATACATATATATACATATTTTGAAAACTATTTAAAACT
AGTATTAATGTTTGTGTTTGTTAATAACCGTAGCTGTGCTTATTAGTCACTTGAATTAGTTATG
TGTTAAACCTAATTTTAAACTAAATGTGAATTTTAATTTTACTGATAAGAATTTCCAAACGGTC
TTGACACCAAAATGTCGAGCATACCAATGTCACGGCAGCCAATTTTTTATTTGATTTTATGTGT
GTAGCAGCTAGCCTCTAAGATTAACTATATTGTCATGTGTCAAAATATGTTAAGAGATATGGTA
AATATATATAAATAAAGTACATAACTTTCAAGGCAGCGTCCTTATTTCTAAAATTTTAAATGGA
CATTTTATTAAAATTACTTACATCTTAGGAAGTGAGTGGAAAAACTTGTTCAAAGATGTTGGGG
ATGCTTTTATTTTCTTGACGTGAAATTTGTACTTCGGAAGTGCAAGTATACAAGAATTTCTAAT
CTAATATAAACGCTCCGGCAAGACAAGAACTGTACTCTGCCTATAAAAATAAGTTATCTAAAAA
AATAAAGTGCAGCTGAAAGACCATTTGGAAGGAAACAGGAGGGATTAATATTCTCCTGAAGATG
AGTTCATCATAGTATTTGGCTTTTTGAACAAAAAGCCATTAAAAGTTGTATTTATATGTTGTAG
CATATTTCATATATATGGCAATCAGATGTGTTGTTTACTTTTGCCGGAACCCGCTTACGATTGA
GATTGACAATGATTGTGACATTAGATGTGCTGTGTTGGCAGAACCACACCATATGTCACGCTTT
AGGCACACATAATCAAGATCTTTGAGCATTAGGCAGACTCTGTGTCAGTCACTCTCGCACACAT
AAACCACATGCAACTGCTGTCTGTGTCGTTTACTTTGCAGCTCGACAGCAAACAGTTTCCGACA
GTCGATGGCATGGCAGCCCACATGCACATTTGGCGGCATCCTTGCTGTTCCATCAATGCGTGCA
GCGTAAGTAATCGGAGCAGGAGCAGGAGGCGGGTGTCGTTGCCGTCGTGCGTGCAATTCTCGTG
GAGGTAATGAAAATTGCATTATTGCCCTGAGTTTAATGCAATTCAAATGCTTTTATGATCAGAG
GCACCAAAAGTTTTTCTTATTCTCCATTAAGTGCAAAATGTAATGCACCGAAAATTGGACAGAT
CGACAGCGCAGCAGCTCCGAGACAGACACAGTAAGCAGTAAGCACTTCGAGTTGTCTAGTCCTG
CAGTAATGAAAATGAAAAAATTGTGTTGCATATCTCGTGGGGCGTGCCGAAGGCGCTGAGCTGT
TGACTAAGCGCTAAAGCGACGACGGCCGCAAAGTGCACGACAAAATGGCGTCGCCAGGCGACAA
AAACAACAACAACAAAGTTGTTGCCGACAATTATATTGTATGGGCAGCAGCAGACAAAAAGAAT
GTGCAAGTGCGAACGAGCCAGAGTGAGATGAAGGCAAGTATGTAAGTAGAGAAAATGTGTGGAA
GCGTGAAATTGATTGCCCCGCTGGCATTCGCTCAGACCTCATCCTTAAACTGTTTGCAGTTTAT
TTGCTTTGGCTTTGGCTTTCGCGTTGGCCTTGGCTAATGGCTGCCCTGGGAATACGATTTTTAA
TTTGCCGACACCTTCAAGTCGCCAACTGAAATGAATCTTTAACATATCAACAGCCAAGCATTTG
CATCTGCGAAATAAAAACACATCCACAAATAAAAGAGGAAACAAGCGCTGATCGCTGAATTGTT
CACATTTGCACATTTTCATAAGAAAAGCACATTACCTGCCGGATTTGGCTTATTTATTATGTGA
CACACACACGCAAAACACACACACACACTCATATGCAGGGGAATTGGCATTCAACACTTTGACG
CTTCATTAGCTTTGAGTCTGGCGGGTTGATGTGGGCCAAGCGTGAATAAAATACAAATGTGATT
CGTAAAGGCATTTCCCTCACATACGATTTGCCATTTATTTTCAAATTGATTTCATCACGATTGC
TATTTTTATATATTTTTGCAATTGGAACATGCCAAAACACTCTAGATTAAGCACTTATATAATA
TAATATTTGTTTACCTAAAAGTTGAAAAATGTTGCATTCAAATACATAAATTATCTCTACCATG
TCAAAATTTCAAGTCTTTAGTCTTTAGTCATAGATCAACTCGGCTATTAATGCTTACCCAGAAC
ATATGTAAATACTCTATGGAATCGGTCAAGCCTGGCCTCCATTCCATTTGACAAAACCATGTTA
CCCGTTTGACCTATTCTCTATGGGTTCATTAAATTAACACAGAAAAACATAATTGCATTTTCAA
ATGGAGTTTAAAGATAAATAAATATGCTGTGTATTTGCCTTACTTTAACAACTAAAGAAACAAT
GAATTACATAAACACAATAATGAAAATGCAAACATGTATTGTTATAATAATAATATAAAATATT
CATAATAAACAATTTGAATGCTAATGAAACAGATGACAACAATACACTTTGTTAGCTGCGGAAG
GCTTTCTGGCTTATCCAAAATTGCGAATAAAAACAATTTTATATATCTGGATTTGTGTGCATGT
TTTTTTGTGTGCTTGTAGATGTGTAATGCGATGTGACTGCTAGTTGAGCATCAGCCTGCAATTA
TTGTTCATTTTGTTACTGCTGTTGTATTGTTTTTGCACCCACTGTGTTTTTGTTTGCTTACAAC
ATACACCATGCGTGTCCCTTAGCTGCAGTTCCCCGATGAGCCGCCGGGGCAGCCATGCATGTAA
TGGATAAAGGCATCTGTCGGAAACGCTATATTAACAGTCGTCCTTTGTTTCAGTAATTAGCCAC
GGGTTGCTACTAACATTTGATATTTTATTCTCAAATAAGTACACCTTCTTTAAGGCCACACGTA
GACTAAGGCCGCAGCTCTTGACTGCCGCTGCAGCATTTGGCCAGTAAGCCGCAAGCACAAAATG
GACCTAAAGCTGGGCCTTTTTCACCTGCTAACTGACAGGCCAACGCGTTTTAGGCAGCCCCCTT
TGTCCACAATCCACTCTTGGTGCCAGATTAGTCAAATTGGTTTCTCGGCTGCCTTTTCCGCGTT
TCCAACTGGCACCTGATGGCCAAGTTATGTGCTCTAAGCCGCCAGTCGTATAATACGACTCTCA
GCGGACGGCAATGCCGGCAGCGCTGATAAATTGAAGTCTTGGCAAAAAACTGTGACGGTAATTC
AGTAGTCGATAATAATGGAAATCAAAGCGGCGACACAAAAAGTTTGATTTGTAAGGACAATTTA
TAGATTCAGAAAGAAATGTATGTACTGGAATTAAATATTAATGTAAGGTCTGCCTATTTACCTT
ACCTTAACTTAAAGCAAGGAAATACAGGGACATATGTAATTCGTATCAAAACAATAATAAAAAC

ATTTTTATTGCATTTTTAAGAAATACATCTGAAATCCCAGACAGACATTAAAACCTTTAACAGC
TGCACAGATTTCGCAGTGATCTCTTTTCCAGAACTTTAAATATAACCTATTAGGGACCTCTTAG
ATATTTGTGATTAAACATTGTACCGCTTCAATACAACATAATTAATGCCATTACTACATAAAAT
AAAGATTTTGTTAGCTAAGATCTTGGCACAACACTGGGAACTTGCGCGGGTGCAGCATTCATTT
CCCTTTCTCGGCAATATCATTAGCAACATTTTGTGCTTGCCGTCCAGAGAGGACGGAACCCTTT
TGGGAACCAAAAAGGTGGGCACATAAATTGAATTCCCTAATTATGCAGCAACGTGTCGGCCGGC
AACATTTGCTAATTGCTTATAAATTGTTGAATTGTTTAATGTTTTTAACAGCTGACTGGCAAAA
AGGGGTTAATGACGAGCGTCCCACGAGGACGATAACTTCAGCTAGGACACATTGTGGGCGGGAA
TTCACGCCGTGCCAGGCAATTAAGAGGCGCAACAAATGGTCATTCTGTGTTAATTAGCCGGCTG
CCTAGTAGCTGCCGCTTGTTACTGGTGATTAGTTACAGCCATCGCATATCGATGCCAGCTAATC
GCATTGGTTGCCGAAACATGTTTAGCTACTTAGTAGCCGGCAGGCTGATTGACCGAGCGATGCG
GCCTAATGAATTGGCACATGACTGCCGCAATGTTGCCGCAACTATTCAAGTCGGATACTTGAGA
TGGCGCACACAGACGGAAATTAATTAAAATGACAAAACCTCATTTTCGCCCAACAGCTCAAACA
AACCTCTGTCAACACCTTTTTGGCTTCCACTCGTTCGTTGGCGACACGAAAACACACATAACCA
CGTTGAGCCTGTCTTCGGACCTGGAGCTGGGCAATCAATCACAGAAATTAAAAATAATCGCTTA
GAGGGAAAAACTAGACTGCTGGACTGAAAGAAAACTGAAAAGAAAATCTTGTTAAAGCGTAAGC
GGGTATAACTTTTATACCCTGTATTTATGTTGCAAGTCGAGAAGAACACATTGGTAACGCTTTC
CATATTAATACATCTACTTTAAAAACTAATATTGCACTGGAAAAATCAAAAGTAGTGATTACGA
CTATAAAAGTATTTTAAAAAGAACATTTATTTTAACACAAAATTTAAATATAATACCAATACAG
TAAAGAAAAAGCTCCATACCAATACAGAGAATAAATTTTTTATTATTTTTGTAAAAAACTTGTG
CCAAAATTGGACTTAAAAATTAAAACTTTTAGCAATTCCCGAATTTTTCAACACACCTCACAAC
TTCTACAAGCATCCATTTCCCTGCTTGATACCGAATACCTTAAAATCCTGTTATATACTGTTAT
ACTGTTATACCAACTAAAGAACTACCGAGTACTGATATTATATATATAAACAATTCGACTATCT
AATTACTAATTTCTGTTTCCAGCCACAGCTGACGCCCAAAATAAATTAGCATGGCTTTTATTTG
ATTTTGTAAATATATGCCCTTTTATTTTGTGCTACAGGGCAAAAGAAGCAAATACGTGAGCTAA
AAAATCGACAACAAATAAATACATTTATCTTCATACGCCCCAAGGGATGCACAGAAAGTTGTTT
GGCTTTTTGTCTTTTTGCAGTTCGTCCACTTTATTTGCTTCCTCTCGCAGGTTGGCCAGACATA
AGTAAACAGAATCGCACAAAAGTCAATTTCCGGCCCTTGGCAAGAACGTGTGTATATTCTGGAA
AAACGTAATGCACACACACACACACAGACTCGCTAGCCAAATAATTCAGAAATGCACACATATT
GATAGAGCACACACCCGCAGCATGTAGTTTGAACAAGGAGTCAATTGTATTTTGTTGCATATTA
TACAAATATTTCGATTGTAG~~TTATGCAAACAAATTCGTTTACTTATAAGTTATGCTATTTACAG~~
~~AATGCCTCATTAGCGCGAACTAAAGAGCATAAACACACCTTTCCGGTGGTATTTGTTATTTGCG~~
~~ACGCTTAAAGTTAATTAGTATTAAAGACACAACTTAAGAATTAGTAAAAACTGCCTTGCCGTGG~~
~~TAATTCTCCTTGATCGTCGCGTCCTGCC~~TTACTTTAGCAAAAAGTCCATTTCTGATTTTGTGGT
TGACATGTACTGATCCAGCTCATTGTCCAAATCCTCGCGCTTTACTTCCCTACGCTGCGGCTTA
C

## *2b* inversion

### *D. novamexicana* 15010-1031.00

### Distal breakpoint

>Nova00_Contig1515: …15,199..7,520… (Dvir ncRNA *GJ26151*[+] - *GJ22656*[-] *5-HT2A*))

~~CAGGACAAAAAAATGGAGGAACTCAAGCTTATATTGGAGAACCGCAAGCTCAGATTGGAGGAAG~~
~~ACAAGTTTGAATTTGAGAAAGAAAAATTTAAGAAAGAAGAAAGATTGGAAGAAAGAAGGTTGAA~~
~~TCTTGAGCGATACAAGATACTTTTCGAAAAATGTCAATTCGAAATAAAGAACAATAAAGTGTTA~~
~~TTTGACCTCAGGCATTTTGAGGATCAGTTATAATGGACATAAGACTGGATTCAAACTCTGTATC~~
~~TCCATCATAGATTGCTCTCAAATGTGTCTCTTGATCTTCTAATAAAATGTGAAGTGGTCAAATC~~
~~GA~~TGATTTTATTTTTCTGTGATTGAAAATAATTGGTATTGATAGTTTTCTTTTCGAATTTTGGT
ACTTGCTATCGGAAAAGTATCGGTCACTTTCAAATGCATTTATTTATGGTTTTAAGCACGAAAC
TAAATAAATTTAAATGCAGAATTGCAAAAAATTAAATTATTTATTATCTACATTCATTTATTTT
TGAATTTTAATTTATAATATGGGTAAACAAAGGGGTTGAAAAGTTAGGTTTTCACCATTCTTTA

AATATCTTAATTCAAGGATAATGGCCCCGAAAACCGCATATAGACGATTGAAGTCTATAGTTTC
ACCTATCCAATCGTGAAAAAATTATAAAAATCGATCAGCACGCTTTTAAGATATTTGTACTACA
GTGCAGCAACCTCGTTGCTAGCCCCATACAAAATGACCGTAAAAAATCACCCTTGCACTTGAAC
CCTAATATCTTTTCTCAGGGATAATGGCCCTCAATGCACGGTATACCAACTTGAAGATACATGT
TTAAGGAAGCTTTACGCATTAATTCTAAACAAATCGATCAGCCAGTTTTTTAGAAAAATAGCAA
AATGTAAACACACCTCATGTTGTTCATTCAAAAGGCCTATCGAGCTACTAGCTCCAACGGGAAC
CGAACCACTGCTCGGAATCAGTGTTGAACAATGTTATACAGTATTGAGCGGGAACCAAATGAAT
TCAAAATACTTGTATATGGAAGAGAAATGCAGTAATATATGAAATAATTGTCATTTTACAATAA
AACATTATCTTTGGTAAAATAGGCTGTTGCAAAAGACGACAACTATGCATTTAAGTATTTATAC
ACGTATAAAAACGAAAAGGTGGCAATATTTCAACCTTTATTATTATATTTTCCGATATAGATCG
CGAATTATCAATCATTTTGCTTTTTACATCAAGAAAATCGTCCAGCCAGTTTTTGAGAAATTCG
CATTCTTCTATTTTCCTTAATATACACCCTGTTATGTAAGAAAAACTATCTGGCAGCATTCATC
CTTAATATATTAATGTTAGGGGCTGCCAACTTAAGAAGAATGCTAACAAAAAGATGCCAGCTTG
TGAAAATAAGTAAAAGTATACATGACGATGAATATTATGTAATCATGATATTTATATTAAATAA
ATATACTTTATATATGTTGAAACATTCTACATCTTTATGTTAGCATTCTTCATAAGTTGGCAGC
CCCTTTTGGTAAATAATAAGAGGTAGAATCACCAAACTTGACATATAACTTCTAAAATAGAATA
TATATATGCATTTGATGTTGGAAGAAGAGGGTTCAGGGTATACCCTAGTCGGGAGCTCCCGACT
AGAACCTCTTACTTGTTCTTTACTGTATTGATACTATATTTGAATTTTGTATTAAAAGAAATGT
ACTTTTTAAAATACTTTTATAGTCGTAATCACTACTTTTGATTTTCCCAGTGCAATACTAGTTT
TTACAGTAGATGTATTGATATGGAAAGCGTTACCAATGTGTTCTTCTCGACTTGCAACATAAAT
ACAGGGCATAAAATAGTTTAGTGCGCTTACGCTTTAACTAGATTCTCTGTTCAGTTTTTTTTCC
CTCTAAGCGATTATTTTTAATTTCTGTGATTGATTGCCCAGCTCCAGGTCCGAAGACAGGTTCA
ACGTGGTTATGTGTGTGCTTTCGTGTCGCTTACGAACGAGTGGAAGCCAAAAAGGTGTTGACAG
AGGTTTGTTTGAGCTGTTGGGCGAAAATGAGGTTTTGTCATTTTAATTAATTTCCGTCTGTGTG
CGCCACCTCAAGCATCCGACTTGAATAGTTGCGGCAACATTGCGGCAGTCATGTGCCAATTCAT
TAGGCCGCATCGCTCGGTCAATCAGCCTGCCGGCTACTAAGTAGCTAAACATGTTTCGGCAACC
AATGCGATTAGCTGGCATCAATATGCGATGGCTGTAACTAATCACCGCTAACAAGCGGCAGCTA
CTAGGCAGCCGGCTAATTAACACAGAATGACCATTTGTTGCGCCTCTTAATTGTCCGGCACGGC
GTGAATTCCCGCCCACAATGTGTCCTAGCTGAAGTTATCGTCCTCGTGGGACGCTCGTCGTTAA
CCCCTTTTTGCCAGTCAGCTGTTAAAAACATTAAACAATTCAACAATTTATAAGCAATTAGCAA
ATGTTGCCGGCCGACACGTTGCTGCATAATTAGGGAATTCAATTTATGTGCCCACCTTTTTGGT
TCCCAAAAGGGTTCCGTCCTCTCTGGACGGCATGCACAAAATGTTGCTAATGATATTGCCGAGA
AAGGGAAATGAATACCGCATAAGTGTAAGTTCCCAGTGTTGTGCCAAGATCTTAATTTATGTAG
TAATAGGATTAATTATATATATATAGTATTGAGGCGGCACAATGTTTTCTCAGAAATATGTAAGAG
GGTCCCTAATAGGTTATATTTAAAGTCCTGGAAAAGAGATCAGTGCGAAATCTGTGCATCTGTT
AAAGGTTTTAACGTCTGTCTGGGATTTCAGATGTATTTCTTAAAAATGTTTTTATTATTGTATT
TCCCTGTATTTCCTTGCGTTAAGTTAAGGTAAGGTAAATAGGCAGACCCTACATTAATATTTAA
TTCCAGTACATAGATTTCTTTTCTTACAAATCAAACTTTTTATGTCGCCGCTTTGATTTGATTT
CCATAATTGTCGTCTACAGAATTACCGTCACAGTTTTTTGCCAAGACTTCAATTTATCGGCGCT
GCCGGCATTGCCGTCCGCTGAGACTCGTATAATACCTGGCGGCTTAGAGCACATAACTTGGCCA
TCAGGTGCCAGTTGGAAACGCGGAAAAGCCAGCCGAGAAACGAATTTGACTAATCTGGCACCAA
AAGTGGATTGTGAACAAAGGGGGCTGCCTAAGACGCGTTGGCCTTTCAGTTAGCAGGTGAAAAA
GGCCCAGCTTTAGGTCCATTTTGCGCTGGCGGATTACTGGACAAATGCTGCAGCGGCAGTCAAG
AGCTGCGGCCTTAAAGAAGATGTACTTATTTGAGAATAAAATATCAAATGTTAGTAGCAACCCG
TGGCTAATTACTGAAACAAAGGACGACTGCTAATATAGCGTTTCCGACAGATGCCTTTATCCAT
TACATGCATGGCTGCCCCGGCTGCTCATCGGGGAACTGCAGCTAAGGGACACGCATGGTGTGTG
GTGTGGTGCACAGTGGGTGCAAAAACAATACAACAGCAGTAACACAATGTACAATAATTGCAGG
CTGATGCTCAACTAGCAGTCACACATGCACACAAATCCAGATATAACATTGTTTTTATTTGCAA
TTTTGGATAAGCCAAAAAGTCTTCCGCAGCTAACAAAGTGTATTGTTGTCATTTGTTTCATGAG
TATTTAAATTGTTTATTATGAATATTTTATGTTATTATTATAACAATACTTATTTGCATTTTCA
TTATTGTGTTTATGAAATTCATTGTTTCTTTAGTTGTTAAAGTATGGCAAATATACACCATATT
TATTTATCTTTAAACTCCATTTGAAAATGCAATTATGTTTTTTTCTGTGTTAATTCAATGAACC
TCAAACGGGTATCATGGTTTTGTCAAATGGATGATGGCTTGACCGATTCCATAGAGTATTTACT
GGGTAAGCATCAATAGCCGAGTTGATCTATGACTAAAGATTAAAGACTTGAAATTTTGAACATG
GTTATTTCAAGTGCACGAGACTATTCCATATTAATTATATAATATTAAATATAGTATTCAACGG
TTTGTTAGGCCTTATGAAAAAAGAATACTTAACATTTTGTTGCTGTACAAAGATACACAAGTAT

TTTATCAAAGTGATGAAATGAAAACTATTTTGAATAAAGCCTGTAGAGATCATTTATGTATTTG
AATGCAACATTTTTCAACTTTTAGGTAAACAAATATTATATTATATAAATGCTTAATCTAGAGT
GTTTTGGCATGTCCCAATTGCAAAAATATATAAAAATAGCAATCGTGATGAAATCAATTCGAAA
ATAAATGGCAAATCGTATGTGAGGAAAATGCCTTCACGAATCGCATTTGTATTTTATTCACGCT
TGGCCCACATCAACCCGCCAGACTCAAAGCTAATGAAGCGTCAAAGTGTTGAATGCCAATTCCC
TGCATATGAGTGTGTGTGTGTGTGTGTGTTTTGCGGGTGTGTGTGTTTTGCGGGTGTGTGTCAC
ATAATAAATAAGCCAAATCCGGCAGGTAATGTGCTTTTCTTATGAAAATGTGCAAATGTGAACA
ATTCAGCGATCAGCGCTTGTTTCCTCTTTTATTTGCGGATGTGATTTTATTTCGCACCATTGTC
TGCTGATGCAAATGCTTGGCTGTTGATATGTTCAAGATTCTTTTCAGTTGGCGACTTGAAGGTG
TCGGTAAATTAAAAATCGTATTCCCAGGGCAGCCATTAGCCAAGGCCAACGCGAAAGCCAAAGC
CAAAGCAAATAAACTGCAAGCAGTTTAAGGATGAGGTCTGAGCGAATGCCAGCGGGGCAATCAA
TTTCACGCTTCCACACATTTTCTCTACTTACATACTTGCCTTCATCTCACTCTGGCTCGTTCGC
ACTTGCACATTCTTTTTGTCTGCTGCTGCCCATACAATATAATTGTCGGCAACAACTTTGTTGT
TGTTGTTGTTCTTTTTGTCGCCTGGCGACGCCATTTTGTCGTGCACTTTGCGGCCGTCGTCGCT
TTAGCGCTTAGTCAACAGCTCAGCGCCTTCGGCACGCCCCACGAGATATGCAACACAATTTTTT
CATTTTCATTACTGCAGGACTAGACAACTCGAAGTGCTTACTGAGTGTGTGTCTGTCTCGGAGC
TGCTGCGCTGTCGATCTGTCCAATTTTCGGTGCATTACATTTTGCACTTAATGGAGAATAAGAA
AAACTTTTGGTGCTGTGATCATAAAAGCATTTGAATTGCATTAAGCTCAGGGCAATAATGCAAT
TTTCATTACCTCCACGAGAATTGCACGCACGACGGCAACGACACCCGCCCCCTGCTCCTGCTCC
GATTACTTACGCTGCACGCATTGATGGAACAGCCAGGATGCCGCCAAATGTGCATGTGGGCTGC
CATGCCATCGACTGTCGGAAACTGTTTGCTGTCGAGCTGCAAAGTAAACGACACAGACAGCAGT
TGCATGTGGTTTATGTGTTCGAGAGTGACTGACACAGAGTCTGCCTAATGCTCAAAGATCATGA
TTATGTGTGCCTAAAGCGTGACATATGGTGTGGTTCTGCCAACGCAGCACATCCAATGTCACAA
TCATTGTCACTCTCAATCGTAAGCGGGTTCCGGCAAAAGTAAACAACACATCTGATTGCCATAT
ATAAGTAGATATATATGAAATATACTACAACATATAAATAAAACTTTTAAAGGCATTTTGTTCA
AAAAGCCAAACACTATGATAAACTCATCTTTAGGAGAATATTAATCTCTCCTGTTTCCTTCCAA
ATGGTTTTTCAGCTGCACTTTATTTTTTTAGATAACTTATTTTTATAGGCAGAGTACAGTTCTT
GTCAACGCCGGAGCGTTTATATTAGATTAGAAATTCTTGTCAAGAAAATAAAAGCATCCCCAAC
ATCTTTGAACAAGTTTTTCCACTCACTTCCTAAGATGTAAGTAATTTAAATAAAATGTCTTTTT
ACAAATTTAGAAAGAAGGACGCTGCCTTGAAAGGTATGTACTTTATTTATAAATATTTACCATA
TCTCTTATCATATTTTGATACATGACAATATAGTTATTCTTAGAGGCTAGCTAAAATAAAACAA
GTAAGAGGTTCTAGTCGGGAGCTCCCGACTAGGGGATACCCTGAACCCTCTTCTTCCAGTATCA
AATGCATATATATATTCTATTTTAGAAGCTATATGTCAAGTTTGGTGACTCTAGCTCTTATTAT
TTACCAAAATTGCCCAACAAACAGGATATCGATATCGATTTTTATCGATTGCTTGGAAACGGAG
TAAGTTATCGATTATCGGAAACAAACTCGATCTGCGCGGGAACTAGGAGCATCTACATCTAAAA
TTTCATGTCTCTATCTCTTATAGGTTCTGAGATCCTTGCGTTCATACATACGGACGGACGGACG
GCCGGACGGACAGACGGACATGGCTAGATCGACTCGGCTATTGATGCTGATCAAGAATATATAC
ACTTTATGGGGTCGGAGATGCTTCCTCTTGCCTGTTACATCCATTTGGATTTTGCACAAATACA
ATATACCCTTATACCCATTTTTAATGGGTTCAGGGTATAGAAAGAAATTGGCTGCCGTGACATT
GGAATGCTCGACATTGCGGTGTCAAGACAGTTTGGAAATTCTTATCAGTAAAAGTAAAATTCAC
ATTTAGTTTAAAATTGTTGAAATTACATTTAGGTTTAACACATAACTAATTCAAGTGACTAATA
AGCACAGCTACGGTTATTAACAAACACAAACATTAATACTAGTTTTAAATAGTTTTCAATATAT
ATATATATATATATATATATATATATGTATATGTAATCTGTAATAAGA~~AGTTTCTGAAATGCTAAG~~
~~ATAGTAAGTACAGTTGACTAAAAGCTATAATGTGATTGCATGAAATATGACAGGTACAGTCACG~~
~~CCTTACTAACACACACAATATATTCCTCTTTCCTGCTGTCTGATGATATGATGAAGTTAACTAA~~
~~GAAGTTTAGCGTTTCTTTTTCTAGGTACAGTTGCTACAATTGTAAGAAGTCAGCGATTATTTAA~~
~~TCGCCATTGGTTTGATTTTGAATAAATCACCCTGTAGTTTACATTTGATTACATGCTTAAAGTA~~
~~CAGTTTGAAGTTTGCTCAATAATTTCATCTGGCTATCAATTCCACATCCGTCTACTCGATTACT~~
~~TGCACTTTCTTACCAACATAGCCCATAAATTCGTATTTGCCCGACTTGCTCCACTCCGCTCA~~TC
ACTCGTCATCGTTGATGCCATAACTGCCCCGGAACTCGCTGAGCGGCGTCGCATTAGCCGTGGA
TGGCGTCATTAGTGGAGCCCCCTGAAACGAGATGGCCATCGGTAGTGCCGAGGGGGCGCACAGG
CTCAGGGCGCTGCGTCCTTCGGTTACGGAGCGATAGCGTAACGGGCGGCCCGAA

**Proximal breakpoint**

>Nova00_Contig1236: …12,153.. 9,995… (*GJ14327*[+](*CG7903*) - *GJ24596*[-](*CG9775*))

GTTCAATGGACCCAGGCCAGCGCATAATATGAGCGGCAATAGAAGATTTTAA~~TGCCAACAACAT~~
~~GCTGTATGTAACATTTTATCATGGACCCTAGACGCCGGTGTAATCTATGTGGATTGCTGACCTA~~
~~ATAGCCGAATTTTGTTTTCCAATAATTGCGCCCATGCAACATCTTAAGTTAAGGCAAACAGTTT~~
~~GAAACAAATTTTCAATAACAATACGCGTTATATTAAGCCCAACCAATCAACAGTATATTTGTTA~~
~~ATTGTATTCATTAAGTATGAAAAGTATTTATCCATTTGAGCAATGTGCAAAGAGAAAGTTTGAC~~
~~AACACGAACGTCTAGTGCGACATGTTCTTCTTTATTTTTGACGACTTACGATATTATCAATACA~~
~~AATATTAATTGCTAACTTG~~GTTTCTTTGAGTTTTTATACAATTGCTAATTTGAGGTAGATGCCA
TTTAAGGTACCCTGTTAAATATAAGGACACAATGAAATTGGACTTGTTGATCGAGCGTAGAATC
CGTTGTGTGAATCAGGTACATCTCATGTTTATAATATCTGTAGATAAACAAACTTAATTCAAAA
ATTTAGGAATTATGATGTGGACAATGACGGACGTTAGCGTCTGAAAACATCGATAGTTCTATCG
AAATTCGATGGTATTTATATTATCACTGGTGGCACTATCGAAAGTGATGTGCGTCAAATTAGCG
GCGGTGACTTAGCGTCTAATTTTGGTACTTGTTATCGGAAAAGTATCGGTCACTTTCAAATGCA
TTTATTTATGTTTTTAAGCACGAAACCAAATAAATTTAAATGCAGAATTGCAAAAAATTCAATT
ATTTACTATCTACATTCATTTTTTTTTTTAATTTTAATTTATAATATGGGTAAAGGGGGTTGAA
AAGTTAGGTTTTCACCATTCTTTAAATATCTTAATTCAGGGATAATGGTCCCGAAAACCGCATA
TACACGATTGAAGTCTATAGTTTCACCTAACCAATCTTGAAAAAATTATAAAAATCGATCAGCA
CGTTTTTAAGATATTTGTACTAGAGTGCAGCAACCTCGTTGCTAGCCCCATACAAAATGACCGT
AAAAAATCACCCTTGCACTTGAACACTAATATCTTTTCTCAGGGATAATGGCCCTCAATGCACG
GTATACCAACTTGAAGATACATGTTTAAGAAAGCTTTACGCATTAATTCTAAACAAATCGATCA
GCCAGTTTTTTAGAAAAATAGCAAAATGTAAACACACCTCATGTTGTTCATTCAAAAGGCCTAT
CGAGCTACTAGCTCCAACGGGAACCGAACCACTGGTCGGAATCAGTGTTGAGCAATGTTATACA
GTATTGAGCGGGAAAAAATTAATTCAAAATACTTGTATATGGAAGAGAAATGCAGTAATATATG
AAATAATTGTCATTTTACAATAAAACATTATCTTTGGTAAAATAGACTGCTGCAAAGACGACAA
CTATGCATTTAAGTATTTATACACGTATAAAAACGAAAAGGTGGCAATATTTCAACCTTTATTA
TTATATTTTCCGATATTGATCGCGAATTATTAATCATTTTGCTTTTTACATCAAGAAATTCGTC
CAGCCAGTTTTTGAGAAATTCGCATTTTTCTATTTTTCTTATTATACAGACAAGTAAACACAAT
CGCACAAAAGTCAATTTCCGGCACTTGGCAAGAACGCGTGTATATTCTGGAAAAACGTAATGCA
CACACACACACACACACACACACTCGCTAGACAAATAATTCAGAAATACACACATATTGATAGA
GCACACACCCGCAGCATGTAGTTTGAACAAGGAGTCAATTGTATTTTGTTGCTATTATACAAAT
ATTTCGATTGTAA~~TTATGCAAAGTCGTTTACTTATAGGTTATGCTATTTACAGAATGCCTCATT~~
~~AGCGCGAACTAAAGAGCATAAACACACCTTTCCGGTGGTATTTGTTATTTGGCACGCTTAAACT~~
~~TAATTAGTACACAACTTAAGAATTAGTAAAAACTGCCTTGCGGTGGTAATTCTCCTTGATCGTC~~
~~GCGTCCTGCC~~TTACTTTAGCAAAAAGTCCATTTCTGATTTTGTGGTTGACATGTACTGATCCAG
CTCATTGTCCAAATCCTCGCGCTTTACTTCCCTACGCTGCGGCTTAC
