# Supplementary material for: Origin and Consequences of Chromosomal Inversions in the virilis Group of Drosophila
Source: Genome Biol Evol. 2018 Oct 30;10(12):3152–66. doi: 10.1093/gbe/evy239 (PMC6278893; doi:10.1093/gbe/evy239)
Supplement: Supplementary Data [file evy239_supp.zip › File S7.pdf]

Ancestral state:

D. virilis

Distal region

>Dvir_scaffold_13047: 9,276,840..9,274,968 (*GJ24114*[-](*Lkb1*)-*GJ23109*[+](*CG9588*))

AGGGTAACGCCACTGCTCCATATGTCCACTTTAAAGCCTGCAAACGTTTCATGTCCATTGGCAA
TTTCTGGCGGTTGAAACGCAGGCGAGCCCTGACCCGTTGTGCAGGTGTCGTCTGGGGCAAACAT
ATCGAGCTGCTCGGCCACGCCAAAATCTGATATTTTGAGCGTTTGGTCAAGCGTCAGCAGCAGA
TTACCCGGCTTGATGTCCTTGTGTATCACACGGCAGCTGTGTAAATACTCCAATCCATTCACCA
GCTGTTGAAAATATTGATGCGCTTGATAGAGCGGCATCTTCTTTTCCGGCGCATGATCCAGCAT
CTCCTGCAAGCCGCCAATGCAATACTCCATGACCAAGTACATCTTTTGTTTCTCCTCATTGTAC
ATCACATCGACCAATTGGACAACATTCTGGTTTTTCAGCTGCTTCAGCAAATGAATTTCCCGTG
TCACATTCTGCTCGCCATTGGGTATGCGGCGTAGCTTTCGTTTGGTGAGTATCTTAACGGCCAG
CCGGCAAAGGTTCTCCGAATTCATGGCTTCCTTTACTTTGCCATAGGAGCCCTCTCCTAGCACA
TCGCCCATCACATATTTGCCCACCATTTTGATGTTCTTCTTTTTCTGTTGATAAATAATTTCCT
GGCTGTCAACGCGGTTAAAGAACATATTGCCTATATCCAGTGTGACACGATCCAGGGCATCGAT
TGCATCATCGTCCAGCCAGGTGACTTGCGGATGCTCCGCATGGTGGTTACCACCAACAGCCGCA
TCGAGTTCCACATCTTTGCTACCATTTAGCTCTACGAAAGCATCTTCTATATCGGATGTTGCGT
CAAAATGGTAGTTTTGATTTGTTAACAGAACGGTCATCTCATCATCTGCCGCGCCAACTAAATC
AACATCCTTGCCAGCCACATCGACCGACTTGTCAGAGCCATGCTGCTGCTCACCGCCCCCGCCA
GATTGCAAAATTGTTGCTTTAGCTTGTGCCTCCATTACATGTGTGCTGGTCACAGTAACAGTCA
TTTGGATGCGTTGCGCTCGCAGCTCAACACACGCTATTTGTGTACACTTATTATATGAACGTAT
AAACGGCGCTCTATTGCAGTTGCCGTCTCTTCGCGCTCTCCTTGTGTGCTCGTGGGCCAAACTA
GAACATTGCAT~~AAACCTTCGAACTGCCCACAACGCACAGTTCACATTTCTATGACGGGTACCCT~~
~~GTTCGAACCTTCGGTCCGTTGTGCACGAAGATTGTCGCAACGCTTACTAGCTTGTTGGTTTTGT~~
~~CCAGTTTTTGCCTATAATGCCTATAAAATTCAGTGTTTACTTTAAACTGGAATATGTTGTATTTA~~
~~GATGTATATGTGTATGTCAA~~TTGTTGATATAATATCGATAATTGCGATATTTTTACTATCGATA
TAGCAGTACTCCCACTTTTGATTAAATTAATTTTTCACCATCTATCGATAACACATAACGTGAC
TTGGTACATGGTTCTATGACAACCACAATCAGTAAGTAAATAAGCCACTTTAGATACAAAATAA
ATCAATTTATAGAAAGGAATAAATATTTTGGTAGAAAAGTCAAAACAATCTCATCGAAAAAAGA
AAATATAAATAAGAAGCGGGAATATCTCTATAAATCGTGTTAGCTAATGCTATAAATACGATAG
ATCGGTTGTATTAGACTCATCT~~CTAACATAATTTATCGGCAGCGCAACAAAAAACTGTTGTTTT~~
~~TATTTTACTTATATTAAAATCCTTCGAAAGGAAAAAGCAAGTC~~ATGGCAACGGAAATCACAACT
AAAGCGCGATTGGAAAGACTAATGGCCGCGAAGGTGGACCTGGAGGCACAGATTAATAAAAATG
GACAAATATTGGCCGCG

Distal region

>Dvir_ scaffold_12855: 5,691,137.. 5,691,699 (*GJ10273*[-]*mura* – *GJ10904*[+](*RnpS1*))

~~CTCTGCACGATTCTCGACCTGACAAAGCGCTGTATATGTATGCACATATGTATTTCTTACTTCA~~
~~ACAAAACGGTCAC~~ACTAACTAGCAGGGTTGCATGGCGCATATATCGAATGTGTTTGGGGTACTT
ATGCACATAAAAGCATTCAATACAGTCCTGCACAGTTTGTTGAACTGACATCGATATGT~~TATCG~~
~~GCAAATAAAAAATATACATCGATGCCCCGAACGCCCATTGAATATTTAACTTGGTATTTTCGTGTT~~
~~TTAAAAAGTTTTGTTTTTGCAACAAATTTGTTAACAATTTGACAACTTATCACTGGATAACTAA~~
~~TTAAGTTACAGCTCAAGTGCAAA~~

D. americana **SF12**

Distal region

>SF12_Contig8643:1..1,573 (*GJ24114*[-](*Lkb1*) -
GJ23109[+](*CG9588*))

TTTTCCGGCGCATGATCCAGCATCTCCTGCAAGCCGCCAATGCAATACTCCATGACCAAGTACA
TCTTTTGTTTCTCCTCATTGTACATCACATCGACCAATTGGACAACATTCTGGTTTTTCAGCTG
CTTCAGCAAATGAATTTCCCGTGTCACATTCTGCTCGCCATTGGGTATGCGGCGTAGCTTTCGC
TTGGTGAGTATCTTAACGGCCAGCCGGCAAAGGTTCTCCGAATTCATGGCCTCCTTTACTTTGC
CGTAGGAGCCCTCTCCTAGCACATCGCCCATCACATATTTGCCCACCATTTTGATGTTCTTCTT
TTTCTGTTGATATATAATGTCCTGGCTGTCAACGCGATTGAAGAACATATTGCCTATATCCAGT
GTGACACGATCCAGGGCATCGATTGCATCATCGTCCAGCCAGGTGACTTGCGGATGCTCCGCAT
GGTGGTTACCACCAACAGCCGCATCTAGCTCCACATCTTTGCTACCATTTAGCTCCACGAAAGC
ATCTTCTATATCGGATGTTGCGTCAAAATGGTAGTTTTGATTTGTTAACAGAACGGTCATCTCA
TCATCTGGCGCGCCAGCTAAATCAACATCCTTTCCAGCCACATCGACCGACTTGTCAGAGCCAT
GCTGCTGCTCACCGCCCCCGCCAGATTGCAAAATTGTTGCTTTAGCTTGTGCCTCCATTACATG
TGTGCTGGTCACAGTAACAGTCATTTGGATGCGTTGCGCTCGCAGCTCAACACACGCTATTTGT
GTACACTTATTATATGAACGTATAAACGGCGCTCTATTGCCGTTGCCGTCTCTTCGCGCTCTCC
TTGTGTGCTCGTGGGCCAAACTAGAACATTGCAT~~AAACCTTCGAACTGCCCACAACGCACAGTT~~
~~CACATTTCTATGAGGGTACGCTGTTCGAACCTTCGGTCCGCTGTGCACGAAGATTGTCGCCAAC~~
~~GCTTACTAGCTTGTTGGTTCTGTCCAGTTTTTGCTCTATTGCTGTAAAATTCAGTGTTTACTTT~~
~~AAACTGGCAATATGTTGTATTTAGATGTATATGTGTATGTCAA~~TTGTTGATATAATATCGATAA
TTGCGATAGTTTTACTATCAATATATCAGTATTCCCACTTTTGATTAAATTTAATTTTTCACAG
CCTATCGATAACACATAACGTGACTTGAGACAAAGTTATATGACAAACACAATCATTGAATAAA
AACGCCACTAGCTTTAGGTACAAAATAAATCAATTTTAAAATAACTTAAATATTTTGAAAGAAA
AGTCAAAAGAATCGCATCGAAAATATAAATGAAAAGCGGGAATATCTCTATGAATCGTGTTAGC
TAATGCTAAAAATACGATAGATCGGTTGTGTTCAACTCATCT~~CTAACACAAATTATCGGCAGCG~~
~~CAACAAAAAACTGTTGTTTTTATTTTACTTATATTAAAATCCTTCGAAAGCAAAAAGCAAGTC~~A
TGGCAACGGAAATCACAACTAAAGCGCGATTGGAAAGACTAATGGCCGCGAAGGTGGACCTGGA
GGCACAAATTAATAAAAATGGACAAATATTGGCCGCG

>SF12_Contig779:? (*GJ10273*[-](*mura*) – *GJ10904*[+](*RnpS1*))

~~CTCTGCACGATTCTCGACCTGACAAAGCGCTGTATATGTATGCACATATGTATTTCTTACTTCA~~
~~ACAAAACGGTCAC~~ACTAACTAGCAGAGTTGCATGGCGCATATATCGAATGTGTTTGGGGTACTT
ATGCACATAAAAGCAATCAATACAGTCCTGCATAGTTTGTTGAACTGACATCGATATGT~~TATCG~~
~~GCAAATAAAAAATATACATCGATGCCCGAACGCCATTGAATATTAAACTTGCTATTTTCGTGTT~~
~~TTAAAAAGTTTTGTTTTTGCAAAAAATTTGCTAACAATTTGACAACTTATCACTGCATAACTAA~~
~~TTAATTTAAAGCTCAAGTGCAAA~~

2c inversion

D. novamexicana 15010-1031.00

Distal breakpoint

>Nova00_Contig909: …17,055..**14,108… (*GJ24114*[-](*Lkb1*) - *GJ10273*[+](*mura*))**

AGGGTAACGCCGCTGCTCCATATGTCCACTTTAAAGCCTGCAAACGTTTCATGTCCATTGGCAA
TTTCTGGCGGTTGAAACGCAGGCGAGCCCTGACCCGTTGTGCAGGTGTCGTCTGGGGCAAACAT
GTCCAGCTGCTCGGCCACGCCAAAATCGGATATCTTGAGCGTTTGGTCAAGCGTCAGCAGTAGA
TTACCCGGCTTGATGTCCTTGTGTATCACCCGGCAGCTGTGTAAATACTCCAATCCATTCACCA
GCTGTTGAAAATACCGATGCGCTTGATAGAGCGGCATCTTTTTTTCCGGCGCGTGATCCAGCAT
CTCCTGCAAACCGCCAATGCAATACTCCATGACCAAGTACATCTTTTGTTTCTCCTCATTGTAC
ATCACATCGACCAATTGGACAACATTCTGGTTTTTCAGCTGCTTCAGCAAATGAATTTCCCGTG
TCACATTCTGCTCGCCATTGGGTATGCGGCGTAGCTTTCGCTTGGTGAGTATCTTAACGGCCAG
CCGGCAAAGGTTCTCCGAATTCATGGCCTCCTTTACTTTGCCGTAGGAGCCCTCTCCTAGCACA
TCGCCCATCACATATTTGCCCACCATTTTGATGTTCTTCTTTTTCTGTTGATATATGATGTCCT
GGCTGTCAACGCGATTAAAGAACATATTGCCTATATCCAGTGTGACACGATCCAGGGCATCGAT
TGCATCATCGTCCAGCCAGGTGACTTGCGGATGCTCCGCATGGTGGTTACCACCAACAGCCGCA
TCTAGCTCCACATCTTTGCTACCATTTAGCTCCACGAAAGCATCTTCTATATCGGATGTTGCGT
CAAAATGGTAGTTTTGATTTGTTAACAGAACGGTCATCTCATCATCTGGCGCGCCAGCTAAATC
AACATCCTTTCCAGCCACATCGACCGACTTGTCAGAGCCATGCTGCTGCTCACCGCCCCCGCCA
GATTGCAAAATTGTTGCTTTAGCTTGTGCCTCCATTACATGTGTGCTGGTCACAGTAACAGTCA
TTTGGATGCGTTGCGCTCGCAGCTCAACACACGCTATTTGTGTACACTTATTATATGAACGTAT
AAACGGCGCTCTATTGCCGTTGCCGTCTCTTCGCGCTCTCCTTGTGTGCTCGTGGGCCAAACTA
GAACATTGCAT~~AAACCTTCCAACTGCCCACAACCCACAGTTCACATTTCTATGACGGTACCCT~~
~~GTTCCAACCTTCGGTCCGCTGTGCACCAAGATTCTCGCAACGCTTACTAGCTTCTTGCTTCTGT~~
~~CCAGTTTTGCTCTATTGCTATAAAATTCAGTCTTTACTTTAAACTGGAATATCTTCTATTTA~~
~~GATGTATATGTGTATGTCAA~~TTGTTGATATAATATCGATAATTGCGATATTTTTACTATCAATA
TATCAGTATTCCCACTTTTGATTAATGTGCGATGTGCGTCAAATTAGCGGCGGTGACTTAGCAT
CGATTCTTGGTACTTGGTATCGGAAAAGGAATCGATCACTTTTAAATGCATTTATTTATGTTTT
TAAGCACAGAACCTAATAAATTTAAATGCAGAATTGCAACAAGTTAAATTATTTACTATCTACA
TTCACTTATTTTTGAATTTTAATTTATAATATGGGTAAACCAAGGGGTTAAAATATTAGGTTCT
CACCATTCCATAAATATCTAAATTTAGGGATGATGGCCCCGAAAACCGCATATACACGATTAAA
GCCTATAGTTTCACCTAGCCAACTGTGAAAATTTGATTAAAATCGTCGGTACCATTTTTAAAAT
ATTTGGAAAAGAGTGCAGCAACCCCCAAGTCAGCCCCCTGTTATGCAAAAAAAAACTATCTGGC
AACATTACATACTTAACTATTATTATTATTAATAAACTAATAAAGTTTAAAATATGGTTGCCAT
TTAACAGTATGATATCTAATATTGCCAGCATGTTTTTTTGCATTAACATAGGATGTAAAGAAAA
AAAATGGAAAAATGCAATTTTTCCATAAACTGCGCTATTAAGTATGACTTAATACTTCTCATAT
AATTTGCTTTGACTTATTAATTTTATTAACTGTGAGTCCTTTCCATTTTTTTATATTTACATAT
AATTCAAATTGAATTGGTTCCCGCTCAGTTCTGCACAAAATTGTTCAACACTGATGCCAGGCAG
TGATTCAGTTCACGTAGGAACTAGCAGTTCGATAGACTTTTTAATTAACTATATGAGGTTGGTT
TACAGTTAATTAATTTTCTCAAAATCTGTACCGTCCATTTACTTATAATTAGTGCTTATAGCTT
CCTCAAATATGTTTCTACAAGAGGGTATACGGTGCATTGAGGGCCATTGTCTCTGAGTTAAGAA
ATAAGGGCTCGAAAACAAGGTTGATTTTTTGCGGTCATTTTATATGGGGCTGACTTGGGGGTTG
TTGCACTCTTTTCCAAATATCTTAAAAATGGTACCGACGATTTTAATCAAATTTTCACAGTTGG
CTAGATGAAACTATAGGCTTTAATCGTGTATATGCGGTTTTCGGGGCCATCATCCCTAAATTTA
GATATTAATGGAATGGTGAGAACCTAATTTATAATATTTACCCATATTATAAATTAAAATTCAA
AAATAAATGAATTTAGATAGTAGATAATTTAATTTATTGCAATTCTGCATTTAAATTTATTTGG
TTTTGTGCTTAAAAACATAAATAAATGCATTTAAAAGTGATCAATTCCTTTTCCGATACCAAGT
ACCAAGAATCGACGCTAAGTCACCGCCGCTAATTTGATGCACATTGTATTGATTGCTTTTATGT
GCATAAGTACCCTAAACATATTCGATATATGCGCCATGCAACTCTGCTAGTTAGT~~GTGACCGTT~~

~~TTGTTGAAGTATGAAATACATATGTGCATACATATACAGCGCTTTGTCAGGTCGAGAATCGTGC~~
~~AGAG~~

Proximal breakpoint

>Nova00_Contig1689: …1498..1… (*GJ23109*[-](*CG9588*) –

CGCGGCCAATATTTGTCCATTTTTATTAATTTGTGCCTCCAAGTCCACCTTCGCGGCCATTAGT
CTTTCCAATCGCGCTTTAGTTGTGATTTCCGTTGCCAT~~GACTTGCTTTTTCCTTTCGAAGGATT~~
~~TTAATATAAGTAAAATAAAAACAACAGTTTTTTGTTGCGCTGCCGATAAATTGTGTTAG~~AGATG
AGCTTAACACAACCGATCTATCGTATTTTTAGCATTAGTTAACACGATTCATAGAGATATTCCC
GCTTCTCATTTATATTTTCGATGCGATTCTTTTGACTTTTCTTCAAAATATTTAAGTTATTTTA
AAATTGATTTTTTTTTTTGTACCTAAAGCTAGTGGCGTTTTTATTCAATGATTGTGTTTGTCATA
TAACTTTGTCTCAAGCCACGTTATGTGTTATCGATAGGCTGTGAAAAATTAAATTTAATCAAAA
ATGTGCGTCAAATTACAGGCGGTGACTTAGCGTCGATTTTTGGTACTTGGTAACGGAAAAGGAA
TCGATCACTTTTAAATGCATTTATTTATGTTTTTAAGCACAGAACCTAATTAATTTAAATGCAG
AATTGTAAAAAATTAAATTATTTACTGCCTACATTAATTTGTTTTTGAATTTTAATTTATAATA
TGGGTAAGCCAAGGGGTTGAAATATTAGGTTTCCACCATTCCATAAATATCTAAATTTAGGGAT
GATGGCCCCGAAAACAGCATATACACGATTAAAGCCTATAGTTTCACCTAGCCAACTGTAAAAA
TTTGATTAAATGTCCGCAAAAAATCAACCTTGTTTTCGAGCCCTTATTTCTTAACTCAGAGACA
ATGGCCCTCAATCAATGTTATACCCTCTTGTAGATACATATTTTAAGAAACTTTAGGCACTAAT
TTTAAGTAAATAGACGGTACAGATTTTGAGAAATTCTCATTTTTCTATTTCTCTCATAACATCC
TATGTTAATGCAAAAAAACAAGCTGGCAATATTATTTATCATACTGTTAAATGGCAGCCATATT
TTAAACTTTATTATTTAATTTCTCCGAAAACGGCCCCAAATGCACGGTAGTTGTGCATATAGAG
TACGATTTGAATGTAAAGAAAAAAATGGAAAAATGCAATTTTTCCATAAACTGCGCTATTAATT
ATGACTTAGTACTTCTCATATAATTTGCTTTGACTTATTAATTTAATTTACTGTGAGTCCTTTC
CATTTTTTCATGATTACATATAATTCAAATTGAATTGGTTCCCGTTCAGTTCTGCACAAAATTG
TTCAACACTGATGCCAGGCAGTGATTCAGTTCACGTAGGAACTAGCAGTTCGATAGACTTTTTA
ATGAACTATATGAGGTTGGTTTACAATTAATTAATTTTCTCAAAATCTGTACCGTCCATTTACT
TAAAATTAGTGCCTATAGCTTCCTAAAATATGTATCTACAAGAGGGTATACCGTGCATTGAGGG
CCATTGTCTCTGAGTTAAGAAATAAG

>Contig545 ..52,162…51,567… - *GJ10904*[+](*RnpS1*)

CCATATTATAAATTAAAATTCAAAAATAAATGAATGTAGATAGTAAATAATTTAATTTTTTGCA
ATTCTGCATTTAAATTTATTTGGTTTCGTGCTTAAAAACATAAATAAATGCATTTGAAAGTGAC
CGATAGTTTTCCGATAACAAGTACCAAAATTGGCCGCTAAATCACCGCCGCTAATTTGACGCAC
ATAGTTTGTTGAACTGACATCGATATGT~~TATCGGCAAATAAAAAATATACATCGATGCCAGAAC~~
~~GCCATTGAATATTAAACTTGGTATTTTCGTGTTTTAAATAGTCTTGTTTTTGCAAAAAATTTGC~~
~~TAACAATTTGACAACTTATCACTGGATAACTAATTAATTTAAAGCTCAAGTGCAAA~~
