# Supplementary material for: Origin and Consequences of Chromosomal Inversions in the virilis Group of Drosophila
Source: Genome Biol Evol. 2018 Oct 30;10(12):3152–66. doi: 10.1093/gbe/evy239 (PMC6278893; doi:10.1093/gbe/evy239)
Supplement: Supplementary Data [file evy239_supp.zip › File S8.pdf]

Ancestral state:

D. virilis

Distal region

>Dvir_scaffold_12963: 11,831,501.. 11,830,849 (*GJ17390*[-] (*CG15435*) – *GJ23332*[+](*FIG4*))

TTTCAGTCCGGATGTCTCTTCAAGCAATACGTTAATTGGCTTCTGATAATTCAGAAACTCGTTA
GTCGAGCCGTGCAAATATGTGGGTATTTTGGCAAAGATATTGTAGTCTGCGGGACTGCTACATA
TGCGGCAAATATTTCCCCACGTCGTCAT~~AGTCTTTGATATTTATAAATATTGAAAATATCGACT~~
~~AGACTAAACAAAACAACAGTATACAAAAAGCGC~~AAGTGTTACTCTATTGGAAAAAAGGAAAAAC
TCTTGTCAGCTGTGTGATGCACTATACAGTGTGTGGCAACTCTGGCTTCAGAGTGCAATGGGCG
GTATTTAAATACCGACACGCACAACCAATGTAAGGTATCGGTACATTGATTGACTGCCATTTTT
TGGCGCCATTGTGAAAAATTCGGGAAATTAAAGCTAGAAATTCGTTTTTTCATTATTATTTTAT
ATAATTAGTGCATAAATTAGTTGCTGTGGCTTGCTAGTAGTGCTATAATTTATTTTCGCTATCG
ATATCTTGATGTTTCGATAAC~~TTATCATTTTATTAGAGGCTGCGATGACTTTGCGTTAAA~~ATGA
ATAATGATAATCCAAATATAATATTTAATCCACTAATTAGTTCCATACAAAAAGTGGTACTTTA
TGAAACACGATCG

Proximal region

>Dvir_scaffold_12723: 4,598,820.. 4,597,134 (*GJ18341*[-](*bwa*) – *GJ17892*[+](*vls*))

CGTGTTAACGAACTCGGCAATATTCGATGATATTAAATAATTGCCCTCGCACCAATCAACGGGC
GAACTTCCGGGTCGAAATAAATAAATGGCATAAATGTCCATTACGCTGCCCGTGTCCAT~~GTTGA~~
~~TTTCAATGAAAGTTAATAATATTAACTAATGCACTTGACGCTATAGTTGGCGTACAACTAATTG~~
~~CTTTGATTAGTTATCCTTTTGTTCTATATTTTTAAGGCACTTGTGTTCTAATGTACTCAATCTT~~
~~GAGCAATTGTTTGCATAATAGCCAAAAATTGCATATAGGCTGTTATTTGCCGTGCCTTTGTTTAT~~
~~ATACACTAATTTGGGTTTTAGTGATTTTGCATGTTCGTTTTCATTGATGTTGCATGCAACTTGT~~
~~GTTTTTGTGTACTAAGTGCCTTATATAAGTTTCTTCGGTTCATTTGTGCCGATGTCGAGGATAA~~
~~CTAGATTACATCTCTTTTATGTGTTTTATCATTGTGCGCTCCTTTTTACTAAATTTGCCAACAT~~
~~GCGTAGTAATGTTAATTTGACGAAAAAATTAAATTAAAACACAAATACGCAGAACAAACACGTC~~
~~TGTCGCGTTGGCGTACGCTTTGTTTACTG~~TTAATACAATGTTAAGCTTGTACTCGAAATGTCAT
TTGCTGATACGAGTAACTCTGAAAATGAGTACTTCGATACCTTAAACAGTGTTGAGAAACGCCA
CATAGCTATCGCAAATCAGATTTTTTTAAATGCAGTAAAACAATTATGTATAAAATTAATATTT
ATGCCTTTACATATATAAATGTTTGAGTAAATTTTGATTGTTGAAAAATGTAAAATTGTTATTG
ATATTATTCATATCCAAAAGTGTATTATTTTTCACACTAGGTTTATTGCCTACGAAAAGTTTTT
AAAATTTTGACTCCAGTATTGGCATTATTTAAACTCAATTGTTGTTATATACGCTACGAAAGTT
TTGCGGCAAATAGAAAATGGTGAAACAGAAAAGTAACGGCTTAAAATTTCATTCAAAAGTATCG
ATATCTGTGATGTATGTAAATGTATGTTAATCGATA~~TACTGATATTTGACAAGACTGTTTTTTG~~
~~GTTTGTTGCCGGCACGCGTTGCTTTCCGGCTTATTTTGATTCTGCATTGACTTTAAGCTCTT~~AT
GATGTTTCCACAGAGGAGTTCGGTAACATATCGCACGCCCGCGACGCACCAGCCACCACCGGCC
AAGGAACTGGCTGACAATGTGGACTATCCCAATCTCAATGTGGCCGATATGAATGCCCGCCTGG
AGAACCTGACACCACGACTGCACGACTGCTTTGACAGCATATCCATGAATGAACTGCAGCATTT
TGCGCTGGCCACAAATCATCGCGAGGGTGCCCAGTGGTGGGGCATGCTCTTCGGGTACAATCAG
ACAGATCACATGGCCGTGGACAATGCGGACTTCAAGCTGCAGTGTGAATGTACTGTTAACATCT
TACGGTATGCTGATAACAACGTGCTGCTCATAGCGCTGGGTGACACCAAATTGCAGGCCTGGTC
CACTTACTCCACGGTGCGCAATCCGGATGAGCCATACTGTTTGTTCCTCATTGGCGAAGATGCA
GCACACAAGGCGCCCATCAATCAGCTCTGTGTGTTTAAGTCGCATCCACAGCAAGCGGTTACCA
CTTGCCTGGACAATACGCTCAAT

Distal region

>SF12_Contig86:…19,602.. 20,248… (*GJ17390*[-] (*CG15435*) – *GJ23332*[+](*FIG4*))

TTTCAGTCCGGATGTCTCTTCAAGCAATACGTTAATTGGCTTCTGATAATTCAGAAACTCGTTA
GTCGAGCCGTGCAAATATGTGGGTATTTTGGCAAAGATATTGTAGTCTGCGGGACTGCTACATA
TGCGGCAAATATTTCCCCACGTCGTCAT~~AGTCCTTAATATTTATAAATATTGAAAATATCGACT~~
~~AGACTAAACAAAACAACAGTATACAAAAAGCGC~~AAGTGTTACTCTATTGGAAAAAGGAAAAACT
CTTGTCAGCTGTATGATGCACTATAAAGTGCTTGACAACTCTGGCATCAGAGTGCAATGGACGG
TATTTAAATACCGACTCGCACAGCCAAGGTATCGGTACTTTGATTGACTGCCATGTTGTGGCGC
CATTGTGAAAAATTCGGGAAATAAAATCTAGAAATTCGTTTTTTCATTATTATGTTATATTATT
AGTGCATAAATTAATTCCTGTGGCTTGCTGATAGTGCTATAATTTATTTGCGCTATCGATATTT
TGATATTTCGATAAC~~TTATCATTTTATTCGAGGCTACGACGACTTTGCGTTAAA~~ATGAATAATG
ACAATCCAAATATAATATTTAATCCACTAATTAGTTCTATACAAAAGGTGGTACTTTATGAAAC
ACGATCG

Proximal region

>SF12_Contig61: …88,741.. 90,420… (*GJ18341*[-](*bwa*) – *GJ17892*[+](*vls*))

CGTGTTAACGAACTCGGCAATATTCGATGATATTAAATAATTGCCCTCGCACCAATCAACGGGC
GAACTTCCGGGTCGAAATAAATAAATGGCATAAATATCCATAACGCTGCCCGTGTCCAT~~GTTTA~~
~~TGTCAATAAAAGTTAATAATATTAACTAATGCACTTGACGCTATAGTTGGCGTACTACTAATTC~~
~~CTTTGATTAGTTATCCTTTTGTTCTATATTTTTAAGGCACTTGTGCTCTAATGTACTTAATCTT~~
~~GAGCAATTGTTTGCATAATAGCCAAAAATTGCATACAGGCAGTTATTTGCGTGCCTTTGTTTAT~~
~~AAATTGTGGTTTCAGTGATTTTGCATATTCGTTTTCATTGATGTTGCATGCAACTTGTGTTTT~~
~~TGTGTACCAAGTGCCTTATATAAGTTTCTTCGGTTCATTTGTGCCGATGTCGAGGCATAACTAGA~~
~~TTACATCTCTTTTATGTGTTTTATCATTGTGCGCTCCTTTTACTAAATTTGCCAACATGCCGTAG~~
~~TAATGTTAATTTGACGAAAAAATTAAATTAAAACACAAATACGCAGAACAAACACGTCTGTCGC~~
~~GTTGGCGTACGCTTTGTTTACTG~~TTAATACAATGTTATGCTTGTACTCGAAATGTCATTTGATG
ATACGAGTAACTCTGCAAATGAGTATTTCGATAGAGTAAACAGTGTTCAAAAATGCCACATAGC
TATCGCAAGTCAGATTATTTTAAATGCTGTGGAAATGCAGTAAAACAATTATGTATACAAATTA
ATATTTATGCAAATATATATAAATGCTTAAGTCAACTTTGACTGTTGAAAAATGTAAAATTGTT
ATTGATATTATTAATACCCAACAGTGTATACTTTTTCATACTACTTGTATTGTTTTCAAGAAGT
TTTTAAATTTTTATCTCCAGTATTGGCATTATAATAATTTTAATACGCTGCGAAAGTTTTGCGG
CGAATAGGAAATTGTGATGCGGCAAAGTAACGGCTTAAAAAGTATCGATATCTGTGATATATGT
AAATGTATGTTAATCGATA~~TACTGATATTTGTAAACACTGTTTTTTGGTTTTTTGCCGGCACGC~~
~~GTTGCTTTCCAGCTTAAGATTTGTTATTTTGATTCTGCGTTGATTTTAAGCATTT~~ATGATGTTT
CCACAAAGGAGCTCGGTAACATATCGCACGCCCGTAACGCACCAGCCACCACCGGCCAAGGAAC
TGGCTGACAATGTGGACTATCCCAATCTCAATGTGGCCGATATGAATGCCCGCCTGGAGAACCT
GACACCACGGCTGCACGACTGCTTTGACAGCATATCCATGAATGAGCTGCAGCATTTTGCGCTG
GCCACAAATCATCGCGAGGGTGCCCAGTGGTGGGGCATGCTCTTTGGGTACAATCAGACTGATC
ACATGGCCGTGGACAATGCGGACTTCAAGCTGCAGTGCGAATGTACTGTTAACATTTTACGGTA
TGCTGATAACAACGTGCTGCTCATGGCGCTGGGTGACACCAAATTGCAGGCCTGGTCCACTTAC
TCCGCGGTGCGCAATCCGGATGAGCCATACTGTTTGTTCCTCATTGGCGAAGATGCAGCACACA
AGGCGCCCATCAATCAGCTCTGTGTGTTTAAGTCGCATCCACAGCAAGCGGTTACCACTTGCCT
GGACAATACGCTCAAT

4a inversion

D. novamexicana 15010-1031.00

Distal breakpoint

>Nova00_Contig5271:…789..1(*GJ17390*[-](*CG15435*)-

TTTCAGTCCGGATGTCTCTTCAAGCAATACGTTAATTGGCTTCTGATAATTCAGAAACTCGTTA
GTCGACCCGTGCAAATATGTGGGTATTTTGGCAAAGATATTGTAGTCTGCGGGACTGCTACATA
TGCGGCAAATATTTCCCCACGTCGTCAT~~AGTCCTTAATATTTATAAATATTGAAAATATCGACT~~
~~AGACTAAACAAAACAACAGTATACAAAAAGGCC~~AAGTGTTACTCTATTGGAAAAAGGAAAAACT
CTTGTCAGCTGTATGATGCACTATAAAGTGTTTGGCAACTCTGGCTTCAGAGTGCAATGGACGG
TATTTAAATACCGACTCGCACAGCCAATGTAAAGTATCGGTACTTTGATTGACTGCCATGTTGT
GGCGCCATTGTGAAAAATTCGGGAAATAAAAGTTAGAAATTCGTTTTTTCATTATTATGTTATA
TTATTGTGAGAAATTCGCATTTTTCTATTTTTCTTATTATACCCCATGTTATGCAAAAAAAAAC
TAGCTGGCAACATTCCATACTTAACATATTTATTCATCAAAATGGCGACGATATTTCAACCTTT
ATTATTCTATTTCTCCGATAATGGCCCCAAATGGCAACATTACATTCCCCAGTGTGGCGTTACG
GGCTTCAAGTTTACGGTATAGCTGCAAAAACACACCTGAACAAAATTCGCATCATTCAGGCCAA
AACCCTGCGCAAAATAACTGGAGCCGAATGGTATATTCGCACACGCGACATCGCCAAGGACCTC
AAGGTTCCAATGGTGGGCGAC

>Nova00_Contig1502:1..1006… - *GJ18341*[+](*bwa*))

CCAAACATCCTGGCGAGGAGCCTGCTAAGGAGGCGAAGAAGAAGGCGACTGAAGAGGACCTACC
CAACCGACCTAATCGATCGTTATGTATAGGTGACAAGCTTCGTTAATTACATCAAAATATGTTT
CTGTAATCTGTTAACCTAATTGTAAAACTAACTGTAAAACCACAATCATCGCTAAGCTAAGTAA
TTATGTCACTCTGATAAAAGTTGCTAACATAGAGTAACAGGATACCAGATTTCTTAATAAACAA
AATATAATTAGTAAAAAACAGCATTTAAAATAATCTGACTTGCGATAGCTAGTTGCATTTTTGA
ACACTGTTTACTCTATCGAAATACTCATTTCCAGAGTTACTCGTATCATCAAATGACATTTTGA
GTACAAGCATAACATTGTATTAA~~CAGTAAACAAAGCGTACGCCAACGCGACAGACGTGTTTGTT~~
~~CTGCGTATTTGTATTTTAATTTAATTTTTTCGTCAAATTAACATTACTACGCATGTTCGCAAAT~~
~~TTAGTAAAAGGAGCGCACAATGATAAAACACATAAAAGAGATGTAATCTAGTTATCCCTCGACA~~
~~TCGCACAAATGAACCGAAGAAACTTATATAAGGCACTTAGTACACAAAAACACAAGTTGCATGC~~
~~AACATCAATGAAAACGAACATGCAAAATCACTGAAACCCAAAATATATAAACAAAGGCACGCAA~~
~~ATAACTGCCTATATGCAATTCTTGGCTATTATGCAAACAATTGCTCAAGATTAAGTACATTAGA~~
~~GCACAAGTGCCTTAAAAATATAGAACAAAAGGATAACTAATCAAAGCAATTAGTAGTACGCCAA~~
~~CAATAGCGTCAAGTGCATTAGTTAATATTATTAACTTTTATTGAAATCAAC~~ATGGACACGGGCA
GCGTTATGGATATTTATGCCATTTATTTATTTCGACCCGGAAGTTCGCCCGTTGATTGGTGCGA
GGGCAATTATTTAATATCATCGAATATTGCCGAGTTCGTTAACACG

Important Note: there is no overlap between the Contigs which include the distal breakpoint of the inversion 4a (I've arranged the Contigs based on the information provided by Evans et al 2007). It should be noted that these authors reported an ITR in this region. Most likely this region is missing in Nova00 genome.

Proximal breakpoint

>Nova00_Contig1110: …596..1… (*GJ17840*[+](*FIG4*) -

CGATCGTGTTTCATAAAGTACCACCTTTTGTATAGAACTAATTAGTGGATTAAATATTATATTT
GGATTATCATTATTCAT~~TTCAACGCAAAATCATC~~ACATGCGTCAAATTAGCGGCGGTGACTTAG
CGTCGAATTTTGGTATTTGTTATCGGAAAACTATCGGTCACTTTCAAATGCATTTATTTATGTT
TTTAAGCATGAAACCAAATAAATTTAAATGCAGAATTTCAAAAAATAAATTATTAACTATCTAC

ATTCATTTATTTTTGAATTTTTATTTATAATATGGGTAAACAAAGGGGTTGAAAAGTTAGGTTT
TTACCATTCTTTAAATATCTTTATTCAGGGATAATGGCCCCGAAAACCGTATATACACGATTGA
AGTCTATAGTTTCACCTAACCAATCTTGAAAAAATTATAAAAATCAATCAGCACGTTTTTAAGA
TATTTGTACTACAGTGCAGCAACCTCGTTGCTAGCCCCATACAAAATGACCGTAAAAAATCACC
CTTGCACTTGAACCCTAATAACTTTTCTCAGGGATAATGGCCCTCAATGCACGGTATACCAACT
TGAAGATACATGTTTAAGGA

>Nova00_Contig2789:16,383..14,517… -*GJ17892*[+](*vls*))

CAAAATGACCGTAAAAAATCACCCTTGCACTTGAACCCTAATAACTTTTCTCAGGGATAATGGC
CCTCAATGCACGGTATACCAACTTGAAGATACATGTTTAAGGAAGCTTTACGCATTAATTCTAA
ACAAATCGATCAGCCAGTTTTTTAGAAAAATAGCAAAATGTAAACACACCTCATGTTGTTCATT
CCAAAGGCCTATCGAGCTACTAGCTCCAACGGGAACCGAACCACTGCTCGGAATCAGTGTTGAA
CAATGTTATACAGTATTGAGCGGGAACAAATTAATTCAAAATACTTGTATATGGAAGAGAAATG
CAGTAATATATGAAATAATTGTCATTTTACAATAAAAnATGTAAGCACACCTCATGTTGTTCAT
TCAAAATGCCTATCGAGCTACTAGCTCCAACGGGAACCGAACCACTGCTCGGAATCAGTGTTGA
ACAATGTTATACAGTATTGAGCGGGAACAAATTAATTCAAAATACTTGTATATGGAAGAGAAAT
GCAGTAATATATGAAATAATTGTCATTTTACAATAAAACAGTATCTTTGGTAAAATAGACTGAT
GCAAAAGACGACAACTATGCATTTTAGTATTTATACACGTATAATAACGAAAAGGTGGCAATAT
TTCAACCTTTATTATTCTATTTCTCCGATAATGGCCCAAATGCACGGTAGTTTAACATATAGAT
CGCGAATTATCAATCATTTTGCTTTTTACATCAAGAAAATCGTCCAGCCAGTTTTTGAGAAATT
CGCATTTATCTATTTTTCTTATTATACCCTCTGTTATGCAAAAAAAAAACTATCTGGCAACATT
ACATACTTAACATATTTATGTTAGGGGCTGCCAACTTATGAAGAATGCACACATAAAGATGTAG
AATGTATGTAGAATGTATAAATGGATGTAGAAAGATATTTAAAGAATGGTGAAAACCTAACTTT
TCAAACCCTTTGTTTACCCATATTATAAATTAAAATTCAAAAATAAATGAATGTAGATAGTAAA
TAATTTAATTTTTTGCAATTCTGCACTTACATTTATTTGGTTTCGTGCTTAAAAACATAAATAA
ATGCATTTGAAAGTGACCGATACTTTTCCGATAACAAGTACCAAAATTCGACGCTAAGTCACCG
CCGCTAATTTGACGCACATCTTAAATTTCATTCAAAAGTATCGATATCTGTGATATATGTAAAT
GTATGTTAATCGATA~~TACTGATACTTGTAAATACTGTTTTTGGTTTGTTGCCGGCACGCGCTGC~~
~~TTTCCGGCTTAAGATTTGTTATTTTGATTCTGCGTTGATTTTAAGCATTT~~ATGATGTTTCCACA
AAGGAGCTCGGTAACATATCGCACGCCCGCAACGCACCAGCCACCACCGGCCAAGGAACTGGCT
GACAATGTGGACTATCCCAATCTCAATGTGGCCGATATGAATGCCCGCCTGGAGAACCTGACAC
CACGGCTGCACGACTGCTTTGACAGCATATCCATGAATGAACTGCAGCATTTTGCGCTGGCCAC
AAATCATCGCGAGGGTGCTCAGTGGTGGGGCATGCTCTTTGGGTACAGTCAGACAGATCACATG
GCCGTGGACAATGCGGACTTCAAGCTGCAGTGCGAATGTACTGTTAACATCTTACGGTATGCTG
ATAACAACGTGCTGCTCATGGCGCTGGGTGACACCAAATTGCAGGCCTGGTCCACTTACTCCAC
GGTGCGCAATCCGGATGAGCCATACTGTTTGTTCCTCATTGGCGAAGATGCAGCACACAAGGCG
CCCATCAATCAGCTCTGTGTGTTTAAGTCGCATCCACAGCAAGCGGTTACCACTTGCCTGGACA
ATACGCTCAAT

Note: In dark blue it is represented a 107 bp overlap (100% similarity) between both Contigs), and a second region with 195bp including an “n” which is repeated immediately after. The repeated region in gray is 100% identical to the DAIBAM reported by Evans et al 2007 while the blue one has 2 differences and the “n”. Thus, something wrong happened here!!!!
