# Supplementary material for: Origin and Consequences of Chromosomal Inversions in the virilis Group of Drosophila
Source: Genome Biol Evol. 2018 Oct 30;10(12):3152–66. doi: 10.1093/gbe/evy239 (PMC6278893; doi:10.1093/gbe/evy239)
Supplement: Supplementary Data [file evy239_supp.zip › File S9.pdf]

Ancestral state:

D. virilis

Distal region

>Dvir_scaffold_12875: 16,940,842.. 16,937,665 (*GJ22245*[-](*aft*) – *mir-31a*[+])

GGTCACAAATTCAGCTTTTGTTTCGTTTTTGAGTCGCCAGGAGATCTCGCCCGACGGATCGCGG
CGATTTGTGTGTGTGCTCCACTCCTGTACGCCATAATTGTTCAGTTTGCTCTTGATGCGATTCA
ACTGTTCCTTGAGCTCCTGCAGCGACTCGTACTGGTAGAATTCGCTAAAGAGCGGCTCTTGCTG
TGGCAGCAACCAGGCGCTGGTCTTTGGCTTTTGGTATAGGAACTTCTTTTCAAAGAGCTGTTCC
ACTTCCTCTCTTATGCTCAGCTTGGGACGCAT~~ATTTGTTTAGGTTTCTTCAATTTGAGCAAGCA~~
~~AAGCTAATTCAAAATCTCTTTTGCATTTATTTTTCCCATAAACAGCGGTCTTAACAGCCCGAAT~~
~~AGCATTAACATTTCAAGAGCTGTTATTAACAGCGCCTAAGAAT~~AACAAGTTATTTGTGGATTAA
TATCATTAAGCAAATTAAGATATTTGCAATGTTGTAAGCTTAAATAGGGGAAGTTTATATTATT
TGGACTGGGCATTTTGGAACAAAAACTTTTGACAGGATGTTTCAATTTGAATTTAACAGTTGTC
TAACAGCTATAAGACTATCGAAAGCTATCGGCACTATCGCAACAGAAAGCGCATCTGAAACAGT
GTTGTAAACTGGCTGTATTTTACCGAGCTGCCAATGCGCAGATTATGGCTAACGTTTCCAATTC
AAAACTCTGATAGCGTGATTCTGTCTTTGCTTTAGCTGTATAATAGGTAAAGGTGTTTGATTGT
AATTATAAATATTTGAGTTATTAATGGACTTACATTACTAATAGAGTTCTTGGTGAGTAAAGAC
ACACAAGTTAGATAGCAGCTTCATACTATATTGCTTGAATTATCGTTTCGCTTTATATCGATAT
ATTAAGTAAAGTTAAAAGAGATACCGATGCATAGTTATCGATAGCTTATTTCACCCGCGAATTT
GTTCGCACTTAAATTCAAATTATTTTAAAACTTGCAATTTTTATGCACTCTCGCTAGGCTGTAA
ATGTAAGGGTTCCAACTCATAAGGCTTTGTCGAAATGTTTTTTGGTCTTTTAAGGTACGAAACA
AACTATGAATGATTTATCCTGGAAGTTAGACTTTAACTTACTTTGTAATATAATTAGTATAAGC
TTATTGGGAATTTCTTTCTATTTGCTTGTCAAGAAAGCGTTTCGTTGTATTCTCTTTAAATTAA
TATCGAAATCTTTCAATTTCTATAACCTGTTTTATATATTTAGTTAATATTTTTGGATATTTCG
AGGGGTTTCTGGATATTTCGAGTTTATAACTTTTATCGAAATTGCTTTCCCAATTCATTTCCGG
CTCAGTTAATGGGAATTTCTTGAGTTTGTGTCCAAAATATTAAATTGATAATTAAAGTAAATTT
CCTTGACTCAGTTAGGCTAACCATAAAAAATAATGGAAATAAGTTGGGGGTTAAAAAAAAAAGA
GATATTATGAAACAATCATACGTAAAAGTATTTATGGACAAATCGTTGCATCGGCCTGTGCATA
TAAATCTGTGCATTAAGTCACAATGGAAGTCAGCAACGGAAATAGAAAATATTGGACACAAAAA
CAAAAAAAAAAAAGATAGAAAAATAACGGTACTTTGTTAAGGCTAAGCCCGAAACCGAAAAGAA
AAGATTTTTGCTTTGGAAGCCAACCAAAAACGAGCAATACGAAAAACCTCTTTGAATCATGGCG
ATGATGTCTCAAACATTGGAATTCAAATTGTTTTTTTATTTTTTTCATATACTTTTTATATTTA
ACTTTTGGCTATATGGTCACTAAATCGGCGCGTGAGCTGCCGACTTTATAGCGAAACGTGCGTT
GTGTGTATATGAATAATAATATTTAAATTGAAGAGCTGCATTTATTAAAAGAAACTCTCTCGGC
TCTTAGGTCCGACAAAATAACAAGTATAGGATTCTATAGTATATAGGAATGCGTATATAATGCA
GACGAGACGCTCTTTTATGCATGTGTATGTGTGTGCATATGTGTGTAAGTCGGGGCACGACACG
TGACTGTTTTATATAATTGGACATAAATACATATATGTAATGTGCTGCGATGGCTCCAACGATT
CCAACTTCGAGAAGAAGCGATTGTCGCCGTGAGACATTCTCCTGGCTATTGGAGCGGAGCTCTA
TTGAAACGCAGGTGTCTGCGGCTTCTGCGGAAGTAACGCCTTCTGGCTAATGTCCAGGCGTGGC
CTTAGGCCCTCTTTGGCAAATGCATTCAATTGGCTTTTGGCTCCGTGGCGGCAAATTCCAATAA
GACGGCGACTCGCTGACGCTGAGTCGAATCGGACGTGTACGTGGACACGGCTTTTGTTGGCAAC
TAAATTTTATTTTAATTTCATATTTCTTTTCTCAAAAGGGAGTTCATTAATATGCCGGGCAGGC
GCCATTGCGCTAAAGGCAAAAAAAAAAACAAAAAAAAAAACGCTCAGCGTCAGCGTCAGCTACCTG
CCACTGGCAGTCTGTACTGTAAACACACACACACACACACGCGCACACGAACGTACGGGTATTT
ATGCTAGTGTGAGTGCACCCCCACTTTGCATAGGCATGCGACTCTGTGCGTGTGCGTGTGTGTG
TGTGTGCTGGCAACGGGTATCAATCCCCGTTTTAGACAACATTTGATTGTATTTGTTGCTTTAA
CTCTTTGAGCTGCACATCGCCGCACGCACACACACACACGCACACGCACGTAAATTGAAACTTC
TTCAATTGTCGCTTTTGGGCTTCTAAACGTCGCCGTTGTCGCCGCTATTTGCTCACGTTTAGTC
CGGCCGAGGTGTGGTGCTAAGCTCGACTCAGGGCTTTAAAATCAGTTGCCAATTAGTCTTCATC
GTTGACGGATTACAATTGCCTCTGCCTCTGCCTCAGCACAAAATAAAAAAAAGAAATAGAAAAA
CTTGTTAAGTGCCTGTCGAAAAAAAAAAGAGAAATAAGAAAACAAGAAATTCATTGATAAAAATA

AGTTTCGTTCATGCGAAATTACAATAACAAAATTTGCTAGCAACATTTTTGTCGCGTTGTTTAT
CCCGTCGCCTCGCA~~TCTGTTGTTCAATTGGCAAGATGTCGGCATAGCTGACGTTTGAAAGCCGCA~~
~~TTTTTATAGCGCTATGCTGCATCTAGTCAGTTATTCAATGGA~~

>Dvir_scaffold_12875: 5415206.. 5411138 (*GJ20866*[+](*FLASH*) - *GJ21471*[-](*AQP*))

TAAACGCAAAAGCTCACGGGAAATCGAAAGAAAACACCTTACCAAATCCTCTACACATGACCAT
AAACCAAAAACCATGGAAGATCTCTTTGGCAGCACGCCCAAGCACCAAAGCCCAGCGACAACAA
CAAATGCAGGCAGCAGCAACGTGGACAGTGAAAAAGTATTCCAGAACCTAATTGCAAATATGGA
ACCGCTAACGCCACAAGAGCTGTACACGCCGGAAAGCCAAATCGAAGCAAGAGTGCAGCAAACA
AGCAGCGGAAAATCTGGAGAAAATAAGCAAAAATCAGCCGAAAATATAAACAAATATTTTGTAT
GCAATATTGAAAGTAAGAACATGGAGCAGAAATTTGCAGAGGATGAACTGGATAATCAAGTTCT
CTCGAAACCAAAAAACCCTTTACCTAGGCCGCATTTAAAACCCTTGGTAAATGCAGAAAATGCA
AAACAAAATCTTCAAAAAGTTGTGCAAGTTGAGTTCGTAAATGAAGTAGCTAATGAAACAAAGC
AGGTACAGGATGCAAATGACGAAGAGCAGTCAGTTCATGAGGTGGATAACATAGAATCTCAGCA
GAACATGTCAGGCACGGACGAAGCGCAGTTAACTGCTCAGGATTTGTTGGATGCAGAAGAAATA
ATTAATAAAGTGCATAATACAAACGAGGAACAACACATTCCGGGCCTGGATTTCATAGCCGAAG
AGCAATTAAAAAGAGAATTGGATGACGCTCAAACAAATCTACACATACCGGGCCTGGATTTGGT
TGATAATGAGACACTAATGAACGATGTGGATAATGGAAAAACAAATGAGCAAATTCCTGGGCTA
GACTTTAAGGAGGAATTAATTACGGAATTGAATACGAAAATACACGACAATGCTCCTGCTCAGG
ATTTGAAGGCCATAAATAAAGTAGATACAAAAGCACATTTATACATACCAGGCCTGGATTTGAT
GAATGAAGAGGCTACCACAAGTAAGGTGGACCCGAGAACGCAGGAATACATACCGGGTCTGAAT
ATGTCCATTGAGGAGGAAGCAACAAAAAATGTTGTGGCAAAAACACATGAAAATATACTGGATA
TGGATTTTGTAATACAAGAGGATCTAACACGAGATGTGGCTAATATGAAAACAAATGAGCATAT
ATCTGTGCTGGACCTAAAGAGTGATTTGGAAGTAACTAAAGCAGTTGTTGCAGAACCACATGAA
TATATACCCGGACTGGACTTTACTGCTGCAGAGAATGTATCGGGCAATGTAGATACTGAACAAC
ACAATTGTCTGGGCTTTGCGGAGGAACAGGATCTGCCAAGCGATGTGGATAGAACAAAAACAAA
CACAACTGATAATCAAGACTGTGCACCAGCTGAGGAACTGAAAGATAGCACGAATAAGATCTCG
ACTGGGAATGAAACCGAAAATACAGAAGCAAAAGGCGAACGGAGCGACAATTCGACAGCAAACA
ATGCACCAAATGAGCACCGTGGGGATAATCAGTTAAGGGAAAGCAATGACAACACGGAATCGTC
TGATGATGATGCTCAGGAAGGCGAACGGAGCGACAATTCGACTGAAAACAATGCACCATGCGAG
CACCGTGGGGAAAATCAGTTAAGGGAAAGCAATGACAACACGGAATCATCTGATGATGGTGCTC
AGGAAGCAGAGAATGCGAAAAGTAAGGACCATAGGGAAAATCAGTTAGTGGAAAGCAACCAATA
CATGGAATCATCTGATGATGATTCTCAGGAGTTAGAGCAACCGAAATGCGAGGACCCTACGGGA
CATCATATGGAATTATCAGAGCTAGAGCAACCGAAATGTGAGGACCCTAGGGCAGGTCAATTAA
CGGAAGGCCATGACAACATGGATTCATCAGAAGATAATGCTCAGGAACTAGAGCAAGCGCAAAG
TGAGGACAACAGGGAAAATCAGTTAAGGGATAGCAATGATAAAATGGAATCATCAGATGATGAT
GCTCAGGAACTAGACGAATCATCATCATCTGATGATGATGATGATCAAGAACTGGAGCAAGCGA
AGAGTGAGGACAAAATGGAAAATCAGTTAATGGCCAGCAATCAAAACTCAGAATCATCCGAACT
TCAAGAAATAGATGCAGCAGCGAAGTCAACATTAAGCGAGTCCACAACGCCGCCCGGCCAGGAC
TCCATCACTTGTCAATTAGATCAATTACCAGCACAGGCAGAGGAGAACATGGTTACGCCCAAGC
GACCAGTCCGTGGCATGAAAAGCATACAAATAATTGAGGACATACGTCTGCCCGTCATGATGGA
TATTGAGAATCTTGCAGTTAAAGTAGATGGAACAAGCGAGGAGCACAGCTTGAAACAAACAGAC
CACCAGCTGTCCAATGCACATGATGTTACTGAGCAAATGCCAGGTCTGAACTTTAATGTCGACA
CATTAACGCAGCCAGCATCGGAGATTGAAGGAGAGTCTCAGCTGCAGTTCATCACAGTTAATCA
AAATGAACGCAACTCGAAAATTGTGGCAGTTAATACGACAATCACGCCCAAGCTGACAGTCGTT
GCAGCTACAGTTATGTACGCCCAGCCGGATTCAACCAAGATTGATCACACCGAGAACGATGCTG
CACTCGAGGCAGTCATGAATGAGCTAATACCAGATAAACAGCAGGCAATCACATTGGACGAAAG
CTATCCGAATTTGAGCCTAGAAACGGATACCATTGAGCTGGCATTGAAACAGCTGCATCAGCAG
TCACAAACCGATGAGCAGCCGGACGAAACAGCTGTAACGTCCACATCCATGAAGGGGCCGCAAA
CGCCCAATCAGGATCTTATGCAGATACTTATGCAATCGCCATTGCAGTCGACAGCTGCGTGCAC
GACGCGAAACAGTCCTACAAAAGTGAGGGTCAGAATAAAGAAACTCGAAGCACGGTCAAACAGC
AAAAGAAAAAGCAACAATGAGGCCAGCAGCGCTACGCCGCAAAATTCAGAAGAAACGCCGCTAA
AAAAACGTAGAGTAGATGTTAACGATGGCGACACAGTGCATACCGCCGATCCAATTGTGCCCCA

AACGCCACCAGAAACGGCCCTGACTACGGAGGCTTCGCAGTTGCATGTGACCATTGATGAGACC
TTCAATGCCAGCACAATGGAGCAAAGCCACTGCCAGAACAGCAACGACTCTTCCATGGTGACCA
AACGCTGCTCTCTGGGCAACAGCGACTATCAATTCGAGCGGATCAATGATGAGGTTGTGTTACG
CGTCACACGTCGTCGACGACGCCGACCAGCAGCTGCCAATCAGGAGTCATAA~~ATACGATATATC~~
~~ATAATGGTTCACACTAGTAAGATATCTGGCTATATGCTTACTTAAGCTATGGGAAGTAATATA~~
~~TACATATACATACATATTTTATGTAACTACAGGGAAACTTATCAATACATAACACAATATTCT~~
~~T~~AAATTATCTACTTAGTTTTAAACAGCATGTAAAAATTGTGATTTTAGATAATAACAGTTGATA
GTATTTGATGTTTAAAAATAAAAAAAAATTAGATAAAAAAATGTAATCCTTTTAAAATTAAAAA
AAAAAATATTGTATTCCATGTTTTATTATTTTACTGTTCGATCAATATTTTTACCCAGCTTTTA
AATCGGTAAATATATATTTTAAATATCGCAACGTGTTATTTTAAAATTAAAATTTTATTTTTTG
ATTGAAATT~~AACAAAATTAGGCTAACGAATTTAAAATTCGATCATTTTTGCTATTGTATTTGT~~
~~CATAAAGTATCGAATAGTATCGCTATCGATAGCAAGGCTTTATAAATTGTGCGGTACTATCGAT~~
~~AATTTTAAAGCATAAGTAAATGAACTATT~~TTAGGCCTGTTTTGGTTTCTCCTCGCCCAGAAGGA
AACGCCGTACAATGGACAGCTTGAAAATGGGCACCGATAACATGGCACCCAGACAGGCGCCAAT
CCAGTATACAATGATGTGCTCCAGATTGCTGTGTCCGCGACAGCCCCACTTGAGAGCCGTGGCC
AGCACCGGATTAAAGTAGCCGCCAGAAAAGTTGAAGG

***D. novamexicana* 15010-1031.00**

Distal region

>Nova00_Contig440:…13251..16505… (GJ22245[-]aft – mir-31a[+])

GGTCACAAATTCGGCTTTTGTTTCGTTTTTGAGTCGCCAGGAGATCTCGCCCGACGGATCGCGG
CGATTTGTGTGTGTGCTCCACTCCTGCACTCCATAATTGTTCAGTTTGCTCTTGATGCGATTCA
ACTGTTCCTTGAGCTCCTGCAGCGACTCGTACTGGTAGAATTCGCTGAAGAGCGGCTCTTGCTG
TGGCAGCAACCAGGCGCTGGTCTTTGGCTTTTGATATTGGAACTTCTTTTCAAAGAGCTGTTCC
ACTTCCTCTCTTATGCTTAGCTTGGGACGCAT~~ATTTATTTAGGTTTCTTCAATTTAAGCAAGCA~~
~~AAACTAATTCAACATGTTTTTTTTTTTCGCATAAACAGCGGTTTTAACAGCGCGTATAGCAGTA~~
~~ACATTTCAAGAGCTGTTATTAACAGCCGCGTAAGAAT~~AACAAAGTTATTTGTGGGTTAATATCAT
TAAGCAAATTAAGATATTTGCAATGATGCAATGGTGTAAGTTTATAAAGGCGTTTATATCTTAT
TTCTGCCGAGAGCTTTTTAAAACAGAAATTTTTGACAGGATGTTTCAATTTGAATTTAACAGTT
TTCTAACAGCTATAAAACTATCGAAAGCTATCGGCACTATCGATACAGAAAGCGCATCTGCAAC
AGTGTTGTAAGCTAGCTGTAGTTTACCGAGCTGCGCAGATTATGGCTTACGTCTCCAATTCAAA
ACTCTGCCAGCGAGATTCTGTCTTCTTTGTTTTAGCTGTATAGTGGGTAAAGGTGATTGATTGT
AATTATAAATATTTCAGTTCTTGGTGAGTAAAGACGCACAAGTTAGATAGCAGCTCATACTTTA
TTGCTTGAAATATCGTTTCGCTTAATATCGATATATCAACTAGAGTTAACAGAGCATAGTTATC
GATAGCTTATATCAACCGCGAATTTGTTCGCATTTAAATTCAAATTATTCCAAAACTTTCAATT
TGTATGCACTTTCGCTAGGCTATAAATGTAAGGGTTCCATCTCATAAAGCTTTATCGAAATGAT
TTGTGGTCTTTTATTTTACGAAAATATCTTTGTTTGCAAACTTTGAATGATTTATCCTGGAAGT
TAGACTTTAAGTGCATAAGCAATATAATTAGTATAAGCTTATTGGGAATTTCTTTCTATTCGCT
TTTCAAGATATTTGATAAATGAAGAACAAATGATGTCTTGTGATGTTCTGTTTTATATATTTTT
GAAAGTACAAAAGAGTTTCTGGTTATTTCGAGTTTAAAACTTTTATCGAAATTGCTTTCCCAAT
TCATTTCCGGCTGAGTTAATGGGAATTTCTTGAGTTTTTTTTATAGGTGTTGCAATAGAAAAGA
AAATAGCTTCCGTGCTGGTGGCAGCACATAAAAACATTTCTAAAACTGTGCCCAAAATATTAAA
TTGATAATTAAAGTAAATTTCCTTGACTCAGTTAGGCTAACCATAAAAAATAATGGAAATAAGT
TGGGGCACAAAAAAAAAAGAGATATTATGAAACAATCATACGTAAAAGTATTTATGGGCAAATC
GATGCATCGGCCTGTGCATATAAATCTGTGCATTAACTCACAATTTAAGTCAGCAACGGAAATA
GAAAATATTGGACATAAAAAAAAAACACACAGAAAAATAACGGTACTTTGGTAAGGGTAAGCCC
AAATCTGAAAAGAAAAGGTTTTTGCTTTGGAAGCCAACCTAAAACGAGCAATACGAAAAACCTC
TTTGAATCATTGCGATGATGTCTCGAACATTGGAATTCAAATTGTTGTCGTTTTTTTTTTTTCA
TATACTTTTTATATTTAACTTTTGGCTATATGGTCACTAAATCGGCGCGTGAGCTGCCGACTTT
ATAGCGAAACGTGCGTTGTGTCTATATGAATAATAATATTCAAATTCAAGAGCTGCATTTATTA
AGTGAAACTCTCTCGGGAGGATTCTATAGTATATAGGAATGCGTATATAATGCAGACGAGACGC
TCTTTTATGCATATATATGTGTATATATGTGCACATGTGTGTACATGTGTGTAAGTCGGGGCGC

GACACGTGACTGTTTTATATAATTGGACATAAATACATATATGTAATGTGCTGCGATGGCTTCA
ACGATTCCAAGTTCGAGAAGAAGCGATTGTCGAGGTAAGACTATCTGCTGGCTATTGGAGCCCA
GCTCTATCTAAGCGCAGGTGTTTTGCGGCTTCTGCGGAAGTAACGCCTTCTGGCTAATGTGCAG
GCGTGGCTTTAGGCCCTCTTTGGCAAATGCATTCAATTGGCTTTTGGCACCGTGGCGGCAAATT
CCAATAAGACGGCGACTCGCTGACGCTGAGTCGGGTCGGACGTGTACGTGGACACGGCTTTTGT
TGGCAACTCAATTTTATTTTAATTTCATATTTCTTTTCTCAAAAGGGAGTTCATTAATATGCCG
GGCAGGCGCCATTGCGCTAAAAAAAAAAAAAAACAACAACAACGCTCAGCGTCAGCGTCAGCTA
CCTGCCACTGGCAGTCTGTACAGTAAACACACACACACACACGTACACGAACGTACGGGTAATC
ATGCTAGTGTGAGTGCACCCCCACTTTGCATAGGCATGCGACTCTGTGCGTGTGTGTGTGTGTG
TGTGTGTGTGTATGTGTGTGCTGGCAACGGGTATCAATCCCCGTTTTAGACAACATTTGATTGT
ATTTGTTGCTTTAACTCTTTGAGCTGCACATCGCCGCACGCACACACACACACACACGCACACG
CACGTAAATTGAAACTTCTTCAATTGTCGCTTTTGGGCTTCTAAACGTCGCCGTCGTCGTCGCC
GTCGCCGTCGTTGTCGCCGCTATTTGCTCACTTTTAGTCCGGCCGAGGTGTGGTGCTAAGCTCG
ACTCAGGGCTTTAAAATCAGTTGCCAATTAGTCTTCATCGTTGACGGATTACAATTGCCGCTGC
CTCAGCAACACACAAATTAAAAAAGAAATAGAAAAACTTGTTAAGTGCTTGACGACAAAAAAAA
AAAGAAAAAAGAACAGAAATAAGAAAACAAGAAATTCATTGATAAAAAATAAGTTTCGTTCATG
CGAAATTACAATAATAAATTTGGTAGCAATATTTTTGTCGTTTTGTTGTTGTTTTTCTTTTGGT
TCAACTATGTTTATCCCTCGGCTCGCA~~TCTGTTGTTCAATTGGCAAGATGTCGGCATAGCTGAC~~
~~GTTTGAAAGCGCATTTTTATAGCGCTATGCTGCATCTAGTCAGTTATTCAATGGA~~

Proximal region

>Nova00_Contig2358: …7875..12141… (*GJ20866*[+](*FLASH*) - *GJ21471*
[-](*AQP*))

AAAAGCACACGGGAAACTGAAAGAAAACACCGTACCAAATCCTCTACACATGACCACAAACCAA
AAACCATGGAAGATCTCTTTGGCAGCACGCCCAAGCACCAAAGCCCAGCGACGACAACAAATGC
AGGCAGCAGCAATGTGGACAGTGAAAAAGTATTCCAGAACCTTATGGCAAATATGGAACCGCTA
ACGCCACAAGAGCTGTACACGCCGGAAAGCCAAATCGAAGCAAGACTACAGCAAACAAGCAGCG
GAAAATCTGGAAAATATAAGCAACAATTAGCCGAAAATATAAACACATATTTTGTATGCAATGT
TGAAAGTAAGGACATGGAACAGAAATTTGCAAGGGATGAGGAACAACTGGATAATCAAGTTGTC
GCGAAACCAAAAAACCCATTGCCTAGTCCGCATTTTAAGCCCTTGCTAAATGCAGAAAATACAA
AACAAAATCTTCAAAATGCTGTAAAAGTTGAGTTCGTAAATGAAGTAGCTAATGAAACAAATCA
GGGACAGGATGCAAATGATGAAGAGCAGTCAGTTCATGAGGATAACATGGATAACATAGAATCT
CAGCAGAACATGTCGGGCACAGACAAAGCGCAGTTAACTGCTCAGGATTTGTTGGATGCAGAAG
AAATAATTAATAAAGTGCATAATACAAACGATCAACAACACATTCCGGGCCTGGATTTTATAGC
CGAAGATCAGTTCAAAAGAGAATTGGATGACGGTCAAACCAATCTACACATTCCGGGCCTGGAT
TTGGTTGATAATGAGACACTCATAAACGATGTGGATAATGGAAAAGCAAATGAGCAAATTCCCG
GGCTAGACTTTAAGGAGGAATTAATTACGGAATTGGATACGAAAATGCACGATAATGCTCCTGC
TCAGGATTTGAAGGCCATAAATAAAGTGGATACAAAACCACATTTATACATACCAGGCCTGGAT
TTGATGGATGAAGAGGCCACCATAAGTAACATGGACCCGAGAACGCAGGAATACATGCCGGGTC
TGAATATTTCCATTGAAGAGGAAGCAACAAAAGATGCAGTGGCAAAAACACATGAAAATATACT
GGATATGGATTTTGTAATACAAGAGGATCTAACACGAGAGGTGGCTAATATGAAAGCAAATGAG
CATATATCTGTGCTGGACCTAAAGAGTGATTTGGAAGTAACTAAAGCAGTTGTTGCAGAACCAC
ATGAATATATACCCGGACTGGACTTTACTACTGCAGAGAAAATAGCGGGCGATGTTGATACTGA
AGCACACAATTGTTTGGGCTTTGCAGAGGAACAGGGGCTACCAAGCGATGTGGATAAAACAAAA
ACAAACACAACTGATAATCAAGACTGTGCACCAGCTGAGGAACTGAAAGATAGCACGAATAAGA
TCTCGACTGGGAATGAAACCGAAAATATAGAAGCAAAAGGCGAAAGGAGCGATGCACCAAGTGA
GCACCATAGGGAAAATCAGTTAAGGGAAAGCAATGACAACAGGGAATCATCTGATGATGATGCT
CAGGAAGGCGAACGGAGCGACAATTCGACTGCAAACAATGCACCAAGTAAGCACCAAAGGGAAA
ATCAGATAAGGGAAAGCAATGACAACACGGAATCATCTGATGATGATGCTCAGGAAGGCGAACG
GTGCGACAATTCGACAGCAAACAATGCACCAAGTGAGCACCATAGGGAAAGTCAGTTAAGGGAA
AGCAATGACAACAGGGAATCATCTGATGATGATGCTCAGGAAGGCGAACGGAGCGACAATTCGA
CTGCAAACAATGCACCAAGTAAGCACCAAAGGGAAAATCAGATAAGGGAAAGCAATGAGAACAC
GGAATCATCTGATGATGATGCTCAGGAACCAGAGCAAGCGAAAATTGAGGACCATAGAGAAAAT
CAGTTAGTGGAGAGCAACCAATACATGGAATCATCTGATGATGATTCTCAGGAGCTAGAGCAAC

CGAAATGTGAGGATCCTAGGGAAAGTCAATTAACGAAAGGCCATGACGACATGGAATCATCAGA
AGATAATGGTCAGGAACTAGAGCAAACGAAAAGTGAAGACAACAGGGAAAATCAGTTAGGGGAA
AGCAATGACAACATGGATTCTTTAGATGATGATGCTCAAGAACTAGAGGAATCATCATCTGATG
ATGATGATGATGATCATGAACTGGAGCAAGCGAAAAGTGAGGACCAAATGGAAAATCAGTTAAT
GGAAAGCAATCAAAACTCAGAATCATCCGATTTTCAAGAAATAGATGCAGCTGCAAAGCCAACA
TTAAGCGAGCCCACAACGCCTCCCAGCCAGGACTCCAACTATTGTCAATTAGATCAATTACCAG
CACAGGCAGAGAACATGGTTACACCCAAGCGTCCAGTCCGTGGCATGAAAAGCATACAAATAAT
TGAGGACATACGCTTGCCCGTCATGATGGATATTGAGAATCTTGCAGTTAAAGTAGATGGAACA
AGCGAGGAGCACAGCTTGAAACAAACAGACGAGCTGTCCAATGCACATGATGTTACTGAGCAAA
TGCCAGGGCTGAATTTTAATGTAGACACATTAACGCAGCCAGCATCGGAGATTGAAGGAGAGTC
TCAGCTGCAGTTCATCACAGTTAATCAAAATGAACGCAACTCGAAAATTGTGGCAGTTAATACG
ACAATCACGCCCAAGCTGACAGTCGTTGCAGCTACAGTTATGTACGCCCAGCCGGATTCAACCA
AGATTGATCACACCGAGGACGATGCTGCACTCGAGGCAGTCATGAATGAGTTAATACCAGACAA
ACAGCAGGCAATCACATTGGACGAAAGCTATCCGAATTTGAGCCTAGAAACGGATACCATTGAG
CTGGCATTGAAGCAGCTGCATCAGCAATCCCAAACCGATGAGCAGCCGGACGAAACAGCTGTAA
CGTCCACATCCATGAAGTTGCCGCAAACCCCCAAACAGGATCTTATCCAGATACTTATGCAATC
GCCATTGCAGTCGACAGCTGCGTGCACGACGCGAAACACTCCTACAAAAGTGAGGGTCAGAATA
AAGAAACTCGAAGCACGGACAACCAGCAAAAAAAGAAGCAACAATGAGGCCTGCAGCGCTGCGC
CGGAAGATTCAAAAGAAACGCCGCTAAAAAAACGTAGAGTAGATGTTAACGATGGCGACACAGT
GAATACCGCCGATCCAATTGTGCCCCAAACGCCACCAGAAACAGCCTTAACTACGGAGGCCTCG
CAGTTGCATGTGACCATTGATGAGACCTTCAATGCCAGCACAATGGAGCAGAGCCACTGCCAGA
ACAGCAACGACTCTTCTATGGTGACCAAACGCTGCTCTCTGGGCAACAGCGACTATCAATTCGA
GCGGATCAATGATGAGGTTGTGTTACGCGTCACACGTCGTCGACGACGCCGACCAGCAGCAGCA
GCTGCACCAGCTGCCAATCGGGAGTCATAA~~ATACTATATATCATAATGGTTCACACTAGTAAGA~~
~~TATCTGGCTATATGCTTACTTAAGCTATGGGATGTTATATATATACATACATATTCTATGTAA~~
~~CTACAAGGAAAACTTATCAAAACATAACACAATATTCTT~~AAATTATCTACTTAGTTTTTAACAG
CATGTAAAAATTGTGATTTTAGATAATAACACTTAATAGTATTTGATGTTTAATAATAAAAAAT
ATACGTCTGGATATAACACAATGAATCCTTTTAAATTATTAAAAAAATATATTGTATGCCAAGT
TTTATTATTTTATTGCTCGATCAATATTATTGCCCAGCCTTTAAATCGGTAAATAGTTACTTTA
AATATCGCAAATTGTTATTTTAAAATATTCATTTTATTTTTTTAATTGAAATT~~AACAACAAAAA~~
~~ATTAGGTCTAACGATTTTTAAATTCGATCATTTTTGCTATCGTATTTAATAATTGTTTAGTATT~~
~~TTTGGTGTCATGAAGTATCGAATAATATCGTTATCGATAACAAGGCTTTATACATAGTGCGGTA~~
~~CTATCGATAATTTTAAAGCATAAATAAATTAACTA~~TTAGGCCTGTTTTGCTTTCTCCTCGCCCA
GAAGGAAGCGCCGTACAATGGACAGCTTGAAAATGGGCACCGATAACATGGCACCCAGACAGGC
GCCAATCCAGTAGACGATGATGTGCTCCAGATTGCTGTGTCCGCGACAGCCCCACTTGAGAGCC
GTGGCCAGCACCGGATTAAAGTAGCCGCCAGAAAAGTTAAAGG

5a inversion

D. americana SF12

Distal breakpoint

>SF12_Contig268: …56,491..51,722…(*GJ22245*[-](*aft*) - *GJ20866*[-](*FLASH*))

GGTCACAAATTCGGCCTTTGTTTCGTTTTTGAGTCGCCAGGAGATCTCGCCCGACGGATCGCGG
CGATTTGTGTGTGTGCTCCACTCCTGCACTCCATAATTGTTCAGTTTGCTCTTGATGCGATTCA
ACTGTTCCTTGAGCTCCTGCAGCGACTCGTACTGGTAGAATTCACTGAAGAGCGGCTCTTGCGA
AGGCAGCAACCAGGCGCTGGTCTTTGGCTTTTGGTATTGGAACTTCTTCTCGAAGAGCTGTTCC
ACTTCCTCTCTTATGCTTAGCTTAGGACGCAT~~ATTTATTTAGGTTTCTTCAATTTAAGCAAGCA~~
~~AAGCTAATTCAAAATCTCTTTTGCATTTATTTTTTGCATAAACAGCGGTTTTAACAGCGCGAAT~~
~~AGCATTAACATTTCAAGAGCTGTTATTAACACCGCCTAAGAAT~~AACTAGTTATTTGTGGTTTAA
TATCATTAAGCTAATGAAGATATTTGCAGTGTTGTAATTGTGTAAGTTTATGAAGGAGTTTATA
TCTTATTTCCGCCGAAAGCTTTTTTGAAACAAAAATTGTTGACAGGATGTTTCAATTTGAATTT

AACAGTTGTCTAACAGCTATAAAACTATCGAACTATCGGCACTATCGAAACAGAAAGCGCATCG
GAAACAGTGTTGTAAACCAGCTGTTGTTTACCGCGCTGCGAAGATTATAAATTTTTGAGTTATT
AATGGACTTACTTACTGTGTATTAATACAAAAATGCAGTGTTGCCTTATTTAGCAGCAGTCTAT
TTTACCAAAGATGTTTTGTTGTAAAATGACAATTATTTCATATATTACTGCATTTCTTTTCCAT
ATACAAATATTTTGAATTAAATTGTTCCCGCTCAATACTGTATAACATTGTTCAACACTGATTC
CGAGTAGTGTTTAGGTTCCCGTTGGAGCTGCTAGCTCGATAGGCCTTTTGAATGAAGAACATGA
GGTATGTTAACACTTTGTTATTTTTCTAAAAAACTGGCTGATCGATTTATTTAGAATTAATGCG
TAAAGCTTCCTTAATCATGTATCTTCAAGTTGTTATACCGTGCATTAAGGGCCATTATCCCTGA
GAAAAGATATTAGGGTTCAAGTGGAAGGGTAATTTTTTACGGTCATTTTGTATGGGGCTAGCAA
CAAGGTTGCTGCACTGTCGTACAAATATCTTAAAAATAATTTTTTCAAGATTGGTTAGGTGAAA
CTATAGACAATTACATTGATCGAGCAATAATACAATAAAACTTGGGACGTAATATATTTTGTTA
ATAATTGAAAAGGATTCATTGTGTTATATCCAGACGTATTTTTTTTTATTATTAGACATCAAAT
ACTATCAACTGTTATTATCTAAAATCACAATTTTTACATGCTGTTAAAAACTAATTAGATAATT
T~~AAGAATATTGTGTTATGCATTGATAAGTTTCCCCTGTAGTTACATAAAATATGTATATGTTTC~~
~~CCATAGCTTAAGTAAGCATATTGCCAGATATCTTACTAGTGTGAACCTTTAGGATATATCGTAC~~
TTATGACTCCTGATTGGCAGCTGCTGTTGCCGGTCGACGTCGTCGACGACGTGTGACGCGCAAC
ACAACCTCATCATTGATCCGCTCGAATTGATAGTCGCTGTTGCCCAGAGAGCAGCGTTTGGTCA
CCATGGAAGAGTCGTTGCTGTTCTGGCAGTGGCTCTGCTCCAGGCTGCTGGCATTGAAGGTCTC
ATCAATGGTCACATGCAACTGCGAGGCTTCCGTAGTCAAGGCTGTTTCTGGCGGCGTTTGGGGC
ACAATTGGATCGGCGGTATTCTCTGTGTCGCCATCGTTAACATCTACTCTACGTCTTTTTAGCG
GCGTTTCTGTTGAATCTTGCGGCGTAGCGCTGCAGGCCTCAATGTTGCTTCTTTTTTTGCTGGT
TGTCCATGCTTCGAGTTTCTTTATTCTGACCCTCACTTTTGTGGGACTGTTTCGTGTCGTGCAC
GCAGCTGTCGACTGCAATGGCGATTGCATAAGTATCTGGATAAGATCCTGTTTGGGCGTTTGCG
GCCCCTTCATGGATGTGGACGTAACAGCTGTTTCGTCCGGCTGCTCATCGGTTTGTGACTGCTG
ATGCAGCTGCTTTAATGCCAGCTCAATGGTATCCGTTTCTAGGCTCAAATTCGGATAGCTTTCG
TCCAATGTAATTGCCTGCTGTTTATCTGGTATTAACTCATTCATGACTGCCTCGAGTGCAGCAT
CGTCCTCGGTGTGATCAATCTTGGTTGAATCCGGCTGGGCGTACATAACTGTGGCTGCAACGAC
TGTCAGCTTGGGCGTGATTGTCGTATTAACTGCCACAATTTTCGAGTTGCGTTCATTTTGATTA
ACTGTGATAAACTGCAGCTGAGACTCTCCTTCAATCTCCGATGCTGTGTCGACATTACAGTTCA
GGCCTGGCATTTGCTCAGTAGCATCATGTGCATTGGACAGCTCGTCTGATTGTTTCAAGCTATG
CTCCTCGCTTGTTCCATCTACTTTAACTGCAAGATTCTCAATATCCATCATGACGGGCAGACGT
ATGTCCTCAATTATTTGTATGCTTTTCATGCCACGGACTGGACGCTTGGGCGTAGCCATGTTCT
CCTCTGCCTGTGCTGGTAACTGATCTAATTGACAAGTGATGGAATCCTGGCTGGCAGGCGTTGT
GGGCTCGCTTAATGTTGGCTTCGCAGCTGCACCTATTTCTTGAAAATCGGATGATTCTGAGTTT
TGATTACTTTCCATTAACTGATTTTCCATTTGATCCTCACGTTTCGCTTGCTCTTGTTCCTGAG
CATCATCATCAGAGGATGATTCCTCTAGTTCCCGAGCATCATCATCTGATGATTCTATGTTGTC
GTTGCTTTCCTCTAACTGATTTTCCCTGTTGTCCTCACTTTTCGCTTGCTCTAGTTCCTGAGCA
TTATCTTCTGATGATTCTATGTTGTCATGGCCTTCCGTTAATTGACTTTCCCTAGGTTCCTCAC
ATTTCGGTTGCTCTGGTTCCTGAGCATCATCATCAGATGATTCCATGTATTGGTTGCATTCCAC
TAACTGATTTTCCCTATGGTCCTCACTTTTCGCTTGCTCTGGTTCCTGAGCATCATCATCAGAT
GATTCCGTGTTGTCATTGCTTTCCCTTAACTGATTTTCCCAATGGTGCTCACTTGGTGCATTGT
TTGCTGTCGACTTGTCGCTCCGTTCGCCTTCCTGAGCATCATCATCAGATGATTCCGTGTTGTC
ATCGCTTTCCCTTAACTGAGTTTCCCTAGGGTGCTCACTTGGTGCATTGCTCCTTTCGTCTTTT
GCTTCAATATTTTCGGTTTCATTCCCAGTCGAGATCTTATTCGTGCTATCTTTCAGTTCCTCAG
CTGGTGCACAGTCTTGATTATCAGTTGTGTTTGTTTTTGTTTTATCCATATCGCTTGGTAGCTC
CTGTTCCTCTGCAAAGCCCAGACAATTGTGTGCTTCAGTATCTGCATCGCCCGCTATTTTCTCT
GCAGCAGTAAAGTCCAGTCCGGGTATATATTCATGTGGTTCTGCAACAACTGCTTTAGTTACTT
CCAAGTCACTCTTTAGGTCCAGCACAGATATATGTTCATTTGTTTTCATACTAGCCACCTCTCG
TGTTAGATCTTCTTGCATTACAAAATCCATATCCAGTATATTTTCATGTGTTTTTGCCAATACA
TTTTTTGTTGCTTCCTCTTCAATGGACATATTCAGACCCGGTATGTATTCCTGCGTTCTCGGGT
CCACGTTACTTATGGTGGCCTCTTCATCCATCAAATCCAGGCCTGGTATGTATAAATGTGGTTT
TGTATCCACTTTATTTATGGCCTTCAAATCCTGAGCAGGAGCATTATCGTGCATTTTCCTATCC
AGTTCCGCAATTAATTCCTCCTTAAAGTCTAGCCCGGGAATTTGCTCATTTGTTTTTGCATTAT
CCACATCGTTTATTAATGTCTCATTATCAACCAGATCCAGGCCCGGAATGTGTAGATTGGTTTG
ACCGTCATCCAATTCTCTTTTGAACTGATCTTCGGCTATAAAATCCAGGCCCGGAATGTGTTGT
TCGTCGTTTGTATTATGCACTTTATTGATTATTTCTTCTACATCCAACAAATACTGAGCAGTTA

CCTGAGCTTTGTCTGTGCCCGACAAGTTCTGCTGAGATGCTATGTTATCCATTTTATCCTCATG
AACTGACGGCTCTTCGTCATTTGCATCCTGTCCCTGATTTGTATCATTAGCTACTTCATTTACG
AACGCAACTTTTACAACATTTTGAAGATTTTGTATTGTATTTTCTGCATTTACCAAGGGTTTTA
AATGCGAACTAGGCAACGGGTTTTTTGGCTTCGCGACAACTTGATTATCCAGCTGTTCCTCATC
CTTTGCAGAATTCTGCTCCATGTCCTTACTTTCAACATTGCATACAAAATATTTGTCTATATTT
TCGGCTAATTTTTGCTTATTTTTTTCAGATTTTCCGCTGCTTGTTTGCTGAAGTCTTGCTTCGA
TTTGGCTTTCCGGCGTGTACAGTTCTTGTGGCGTTAGCGGTTCCATATTTGCCATTAGGTTCTG
TAATACTTTATCACTGTCCGCATTGCTGCTGCCTGCATTTGTTGTCGTCGCTGGGCTTTGGTGC
TTGGGCGTGCTGCCAAAGAGATCTTCCATGGTTTTTGTTTTGTGGTCATGTGTAGCGGATTTGG
TATGGTGTTTTCTTTCGGTTTCCCGTGAGCTTTT

Proximal breakpoint

>SF12_Contig608:..41,532..45,225(*mir-31a*[-] -

~~TCCATTGAAGAACTGACTAGATGCAGCATAGCGCTATAAAAATGCGCTTTCAAACGTCAGCTAT~~
~~GCCGACATCTTGCCAATTGAACAACAGA~~TGCGAACCGAGGGATAAACATAGTTGAATCAAAAAA
ACCAACGCGACAAAAATGTTGTTACCAAATTTGTTATTGTTATCTCGCATGAACGAAACTTATT
TTTATCAATGAATTTCTTGTTTTCTTATTTCTCTTCTTATTTTTTGTTTTTTTTTTTGTTTTCG
ACAGGCACTTAACAAGTTTTTCTATTTCTTTTTTATTTCTTTTCAATTTGTGTGTTGCTGAGGC
AGACGCAATTGTAATCCGTCAACGATGAAGACTAATTGGCAACTGATTTTAAAGCCCTGAGTCG
AGCTTAGCACCACACCTCGGCCGGACTAAAAGTGAGCAAATAGCGGCGACAACGACGACGACGA
CGACGGCGACGGCGACGGTTAGAAGCCCAAAAGCGACAATTGAAGAAGTTTCAATTTACGTGCG
CGTGTGTGTGTGCGTGCGGCGATGTGCAGCTCAAAGAGTTAAAGCAACAAATACAATCAAATGT
TGTCTAAAACGGGGATTGATACCCGTTGCCAGCACACACACACACACACACACACACACACACG
CACAGAGTCGCATGCCTATGCAAAGTGGGGGTGCACTCACACTAGCATGAATACCCGTACGTTC
GTGTGCGTGTGTGTGTGTGCTTACTGTACAGACTGCCAGTGGCAGGGAGCTGACGCTGACGATG
AGCGTTTTTTTTTTTTTTTTTTTTTAGCGCAATGGCGCCTGCCCGGCATATTAATGAACTCCCT
TTTGAGAAAAGAAATATGAAATTAAAATAAAATTTAGTTGCCAACAAAAGCCGTGTCCACGTAC
ACGTCCGACCCGACTCAGCGTCAGCGAGTCGCCGTCTTATTGGAATTTGCCGCCACGAAGCCAA
AAAGCCAATTGAATGCATTTGCCAAACAGGGCTTAAAGCCACGCCTGGACATTAGCCAGAAGGC
GTTACTTCCGCAGAAGCCGCCAACACCTGCGTTTCGCTAGAGCTGGGCTTCAATAGTCTCACGG
CGACAATCGCTTCTTCTCGAAGTTGGAATCGTTGGAGCCATCGCAGCACATTACATATATGTAT
TTATGTCCAATTAGATAAAACAGTCACGTGTCGTGCCCCGACTTACACACATACACACATACAT
ATACACATATACATGCATAAAAGAGCGTCTCGTCTGCATTATATACGCATTCCTATATACTATA
GAATCCTCCCGCGAGAGTTTCACTTAATAAATGCAGCTCTCGAATTTGAATATTATTATTCATA
TACACACATCGCACGTTTCGCTATAAAGTCGGCAGCTCACGCGCCGATTTAGTGACCATATAGC
CAAAAGTTAAATATAAAAAAAGTATAAGAAAACAAAAAAAAAACCATTTGAATTCCAATGTTCG
AGACATCATCGCAATGATTCAAAGAGGTTTTTCGTAGTGCTTGTTTTTGGTTGGCTTCCAAAGC
AAAAACCTTTTCTTTTTAGATTCGGGCTTAGCCTTACCAATGTACCGTTGTTTTCTGTATTTTT
TTTTTTTTTGTGTCCAATATTTTCTATTTCCGTTGCTGACATAAATTGTGACTTAATGCACAG
ATTTATATGCACAGGCCGATGCATCGATTTGCCCATAAATACTTTTACGTATGATTGTTTCATA
ATATCTCTTCTTTTTTTTTTGTGCCCCAACTTATTTCCATTATTTTTTATGGTTAGCCTAACTG
AGTCAAGGAAATTTACTTTAATTATCAATTTAATATTTTGGGCACAGTTTTAGAAATGTTTTTA
TGTGCTGCTGCCAGCACGGAAGCTATTTTCTTTGCTATTGCAGCACCTATAAAAAAAAACTTAA
GAAATTCCCATTAACTGAGCCGGAAATGAATTGGGAAAGCAATTTCGATAAAAGTTTTAAGCTC
GAAATAACCAGAAACCCTTTGGTATTTACAAAAATATTAACTAAATATATAAAACAAAACATCA
CAAGACCTCATTTGTTATTAATTTATCACAAAAGAAAATAGAACAAAACGCTTTCTTGAAAAGC
GTATCGAGAGAAATGCCCAATAAGCTTATACTAATTATATTACTAATTAACTTAAATTCTAACT
TTCAGGATAAATCATTCAAGGTTTGCAAACAATTATATTTTCGTACAATAAAAGACCAAAAAAC
ATTTCGACAAAGCTTTATGACATGGAACCCTTACATTTATAGCCTAGCGAGAGTACACACAAAT
TGTCAGTTTTTGAATAATTTGAATTTAAATGCGAACAAATTCGCGGTTGAAATAAACTATCGAT
ATATATGTATCGGTAGCTCTGTTAACTTTATGTGCGTCAAGTTAGCGGCGGAGACGTAGCGTCG
AATTTTAAGTTGGCAGCCCCTAACATAAATATATTAAGTTTGTAGTGTTGCCAGCTAGTTTTTT
TTGCACAACAGGGGGTATAATAAGAAAAATAGAAAAATGCGAATTTCTTAAAAACCTGCTGGAC
GATTTTCTTGATGTAAGATAGTTCGCGATCCATATGATTAACTACCGTGCATTTGGGGCCATTA

ACGGAGAAATTAAATAATAAAGTTTGTTATATGGCCGCCATTTTACAGTGAGATATATAATATT
GCCAGCTTGTTTATTTTGCATTAACATAGGGTTTAATGAAAAATAAAAAGGAAAAATTCAACTT
TTCCATAAACTGCGCTATTAAGTATTATGAATACTAATATAATTTACTTTGACTTATTAATTTT
ATAATTAGTGAGTCCTTTATAATTCAAATTGATTTGGTTCCCGCCCAACTCTGTACAAAATTGT
TCAACACTGATTCCAGGCAGTGATTCAGTTCACGTAGGAAGTAGCAGCTCGATAGACTTTATGA
ATGAACTATAAAAAGTTGGTTTACAATTAATTTATTTTCTCAAAATCTGTACCGTCCATTTACT
TAAAATGAGTGCCTAAAGCTTCTTAAAATATGTATCTACAAGAGGGTAAAGCGTTAATTGAGGG
CCATTGTCTCTGAGTTCAGAAATAAGGCCTCGAAAACAAGGTTGATTTTTTTGCGGTTATTTTA
TATGGAGCTGACACGGAGGTTGTTGCACTCTTTTCCAAATATATTAAAAATGGTACCGACGATT
TGAATCAAATTTTCACAGTTGGCTAGGTGAAACTATAGGCTTTAATCGTGTATATGCAGTTTTC
GGGGCCATTTTTCCTAAATTATGTTATTTAAAGAATGGTGGAAACCTAATTTTTCAACCCCTTG
GTTTACCCATATTATAAATTAAAATTCATAAAACAAATGAATGTATATAGTAAATAATTTAATT
TTTAACAATTCTGCATTTACATTTATTTGATTTCGTGCTTAAAACATAACGTATTGCATTTCAA
AGTGACCGATACTTTTCCGATAACAAGTACCAAAATTGACGCACATAAAAATGGGGGATAACGA
ATTTTAAATTCGATAATTTTTGCTAACGTATGTAATAATTGTTTAGTATTTTTGGTGTCATGCA
GTATCGAGTAACATCGTTATCGATAACAAGGTTTTATAAATAGTGCGGTACTATCGATAACTTT
AAGGCATAAATAAATTAACTATTAGGCCTGTTTTGCTTTCGTCTCG

>SF12_Contig480:1..269 - ***GJ21471*[-](*AQP*))**

TTTATAAATAGTGCGGTACTATCGATAACTTTAAAGCATAAATAAATTAACTATTAGGCCTGTT
TTGCTTTCGTCTCG~~CAAATAAAAAAGCGGCGCTAACGAATTTTAAATTCGATCATTTTTGCTAAC~~
~~GTATTTAATAATTGTTTAGTATTTTTGGTGTCATGCAGTATCGAGTAATCTCGTTATCGATAAC~~
~~AAGGCTTTATAAATAGTGCGGTACTATCGATAACTTTAAAGCATTAATAAATTAACTA~~TTAGGC
CTGTTTTGCTTTCTCCTCGCCCAGAAGGCAGCGCCGTACAATGGGCAGCTTGAAAATGGGCACC
GATAGCATGGCACCCAGACAGGCGCCAATCCAGTAGACGATGATGTGCTCCAGATTGCTGTGTC
CGCGACAGCCCCACTTGAGAGCCGTGGCCAGCACCGGATTAAAGTAGCCGCCAGAAAAGTTAAA
GG

Note: region in blue corresponds to overlap between Contigs
