# Supplementary material for: Origin and Consequences of Chromosomal Inversions in the virilis Group of Drosophila
Source: Genome Biol Evol. 2018 Oct 30;10(12):3152–66. doi: 10.1093/gbe/evy239 (PMC6278893; doi:10.1093/gbe/evy239)
Supplement: Supplementary Data [file evy239_supp.zip › Legends supplementary materials.docx]

**Supplementary Material**

**Supplementary Table 1:** Genome assembly properties of the *D. novamexicana* 15010-1031.00 and *D. americana* SF12, H5 and W11 genomes.

**Supplementary Table 2:** Number of complete coding sequences (CDSs) and genes, annotated in the *D. virilis* (http://flybase.org FB2017_05), that could be annotated in *D. novamexicana* and *D. americana* draft genomes.

**Supplementary Fig 1:** Detailed schematic representation of a *D. virilis* *X* chromosome reordered based on the location of inversion *Xa*. This inversion is fixed in *D. virilis* and it is absent in all other species of the *virilis* phylad (Fonseca et al. 2012). Gene names are those of the *D. melanogaster* orthologous genes.

**Supplementary Fig 2:** Detailed schematic representation of a *D. virilis* *X* chromosome reordered based on the location of inversion *Xb*. This inversion is fixed in *D. lummei*, *D. americana* and *D. novamexicana* and occurred in an ancestral chromosome without inversion *Xa* (Hsu 1952). Gene names are those of the *D. melanogaster* orthologous genes.

**Supplementary Fig 3:** Detailed schematic representation of a *D. virilis* *X* chromosome reordered based on the location of inversion *Xc*. This inversion is polymorphic in *D. americana* and fixed in *D. novamexicana* and occurred in a chromosome with inversion *Xb* (Hsu 1952). Gene names are those of the *D. melanogaster* orthologous genes.

**Supplementary Fig 4:** Detailed schematic representation of a *D. virilis* *2^nd^* chromosome reordered based on the location of inversion *2a*. This inversion is fixed in *D. virilis* and it is absent in all other species of the *virilis* phylad (Throckmorton 1982). Gene names are those of the *D. melanogaster* orthologous genes.

**Supplementary Fig 5:** Detailed schematic representation of a *D. virilis* *2^nd^* chromosome reordered based on the location of inversions *2b* and *2c*. These inversions are polymorphic in *D. americana* in relatively low frequencies and are fixed and co-occur in the same chromosome in *D. novamexicana* (Hsu 1952). Gene names are those of the *D. melanogaster* orthologous genes.

**Supplementary Fig 6:** Detailed schematic representation of a *D. virilis* *4^h^* chromosome reordered based on the location of inversion *4a*. This inversion is polymorphic in *D. americana* and fixed in *D. novamexicana* as well as in *D. lummei* (Hsu 1952). Gene names are those of the *D. melanogaster* orthologous genes.

**Supplementary Fig 7:** Detailed schematic representation of a *D. virilis* *5^th^* chromosome reordered based on the location of inversion *5a*. This inversion is polymorphic in *D. americana* and fixed in *D. lummei* (Hsu 1952). Gene names are those of the *D. melanogaster* orthologous genes.

**Supplementary Fig 8:** Detailed schematic representation of a *D. virilis* *5^th^* chromosome reordered based on the location of inversion *5b*. This inversion is polymorphic in *D. americana* and fixed in *D. novamexicana* (Hsu 1952). Gene names are those of the *D. melanogaster* orthologous genes.

**Supplementary File 1**. Guide to read through the sequence annotation files (Supplementary Files 2-10)

**Supplementary File 2.** Sequence annotation of the genes and transposable elements found surrounding the inversion breakpoints of inversion *Xa*. Colors as in Figure 2.

**Supplementary File 3.** Sequence annotation of the genes and transposable elements found surrounding the inversion breakpoints of inversion *Xb*. Colors as in Figure 2.

**Supplementary File 4.** Sequence annotation of the genes and transposable elements found surrounding the inversion breakpoints of inversion *Xc*. Colors as in Figure 2.

**Supplementary File 5.** Sequence annotation of the genes and transposable elements found surrounding the inversion breakpoints of inversion *2a*. Colors as in Figure 2.

**Supplementary File 6.** Sequence annotation of the genes and transposable elements found surrounding the inversion breakpoints of inversion *2b*. Colors as in Figure 2.

**Supplementary File 7.** Sequence annotation of the genes and transposable elements found surrounding the inversion breakpoints of inversion *2c*. Colors as in Figure 2.

**Supplementary File 8.** Sequence annotation of the genes and transposable elements found surrounding the inversion breakpoints of inversion *4a*. Colors as in Figure 2.

**Supplementary File 9.** Sequence annotation of the genes and transposable elements found surrounding the inversion breakpoints of inversion *5a*. Colors as in Figure 2.

**Supplementary File 10.** Sequence annotation of the genes and transposable elements found surrounding the inversion breakpoints of inversion *5b*. Colors as in Figure 2.
