# Supplementary material for: Origin and Consequences of Chromosomal Inversions in the virilis Group of Drosophila
Source: Genome Biol Evol. 2018 Oct 30;10(12):3152–66. doi: 10.1093/gbe/evy239 (PMC6278893; doi:10.1093/gbe/evy239)
Supplement: Supplementary Data [file evy239_supp.zip › Table S1.pdf]

Table S1. Genome assembly properties of the *D. novamexicana* 15010-1031.00 and *D. americana* SF12, H5 and W11 genomes.

	D. novamexicana	*D. americana*		
	15010-1031.00	SF12	H5[§]	W11[§]
Contig number	16466	27687	17414	24840
N80	10825	5218	7914	5402
N50	30278	22571	21109	14204
N20	68568	57485	46040	32166
Minimum contig size*	500	500	500	500
Maximum contig size*	364063	261705	229026	135376
Total size#	157.1	160.2	160.6	162.4

N80, N50 and N20 are the shortest sequence length at 80%, 50% and 20% of the genome, respectively

* In base pairs (bp)

# in Megabase pairs (Mbp)

[§] Published in (Fonseca et al. 2013)
