# Supplementary material for: Origin and Consequences of Chromosomal Inversions in the virilis Group of Drosophila
Source: Genome Biol Evol. 2018 Oct 30;10(12):3152–66. doi: 10.1093/gbe/evy239 (PMC6278893; doi:10.1093/gbe/evy239)
Supplement: Supplementary Data [file evy239_supp.zip › Table S2.pdf]

Table S2. Number of complete coding sequences (CDSs) and genes, annotated in the *D. virilis* (Flybase.org), that could be annotated in *D. novamexicana* and *D. americana* draft genomes.

	CDS	Genes
D. virilis	20302	13374
D. novamexicana 15010-1031.00	14487 (71%)	10299 (77%)
D. americana SF12	13329 (66%)	9571 (72%)
D. americana H5	13878 (68%)	9155 (68%)
D. americana W11	12007 (59%)	7899 (59%)
