# Supplementary figures and images for: Origin and Consequences of Chromosomal Inversions in the virilis Group of Drosophila
Source: Genome Biol Evol. 2018 Oct 30;10(12):3152–66. doi: 10.1093/gbe/evy239 (PMC6278893; doi:10.1093/gbe/evy239)

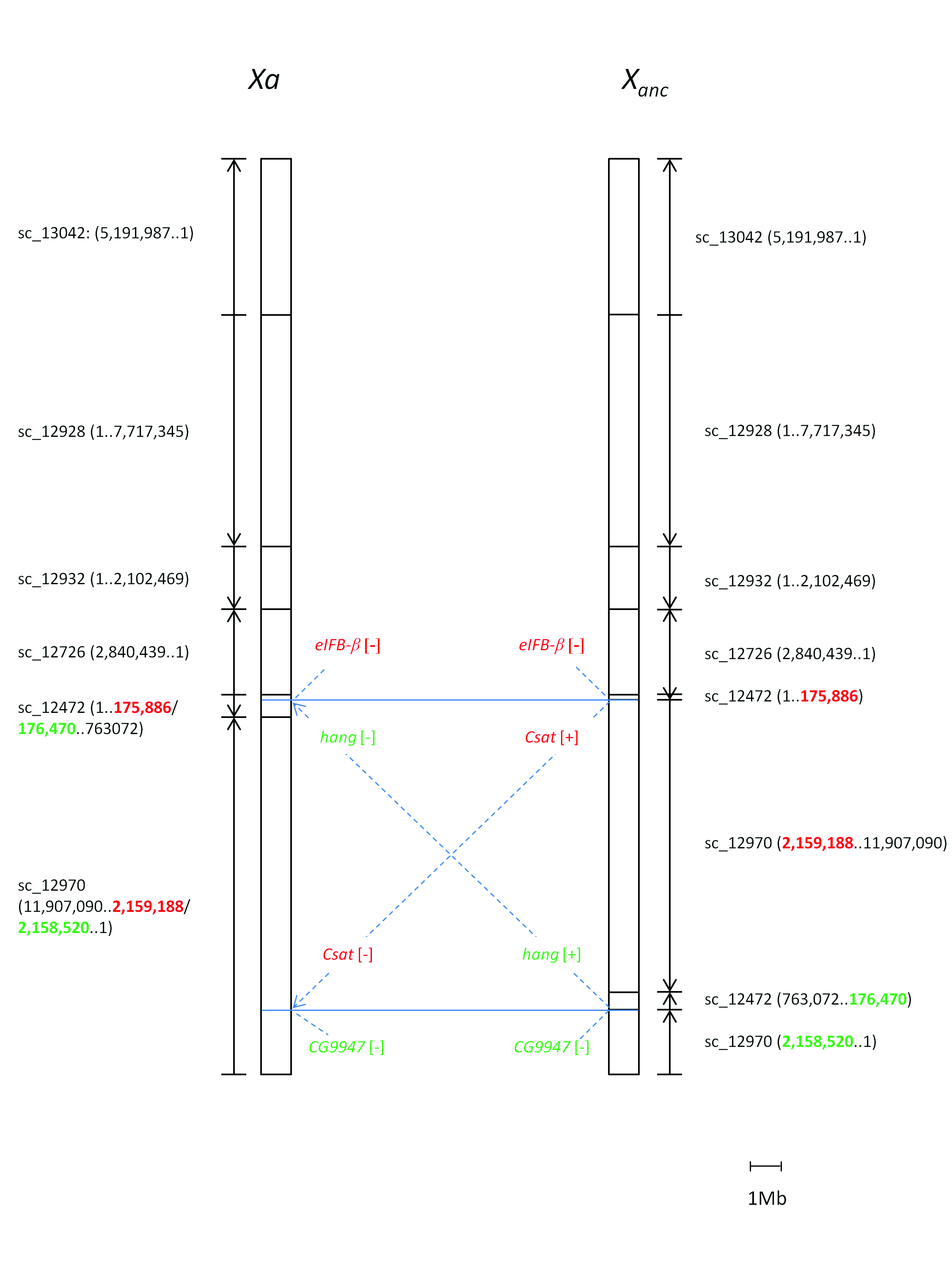

Supplement: Supplementary Data [file evy239_supp.zip › FigS1CMYK.tif]

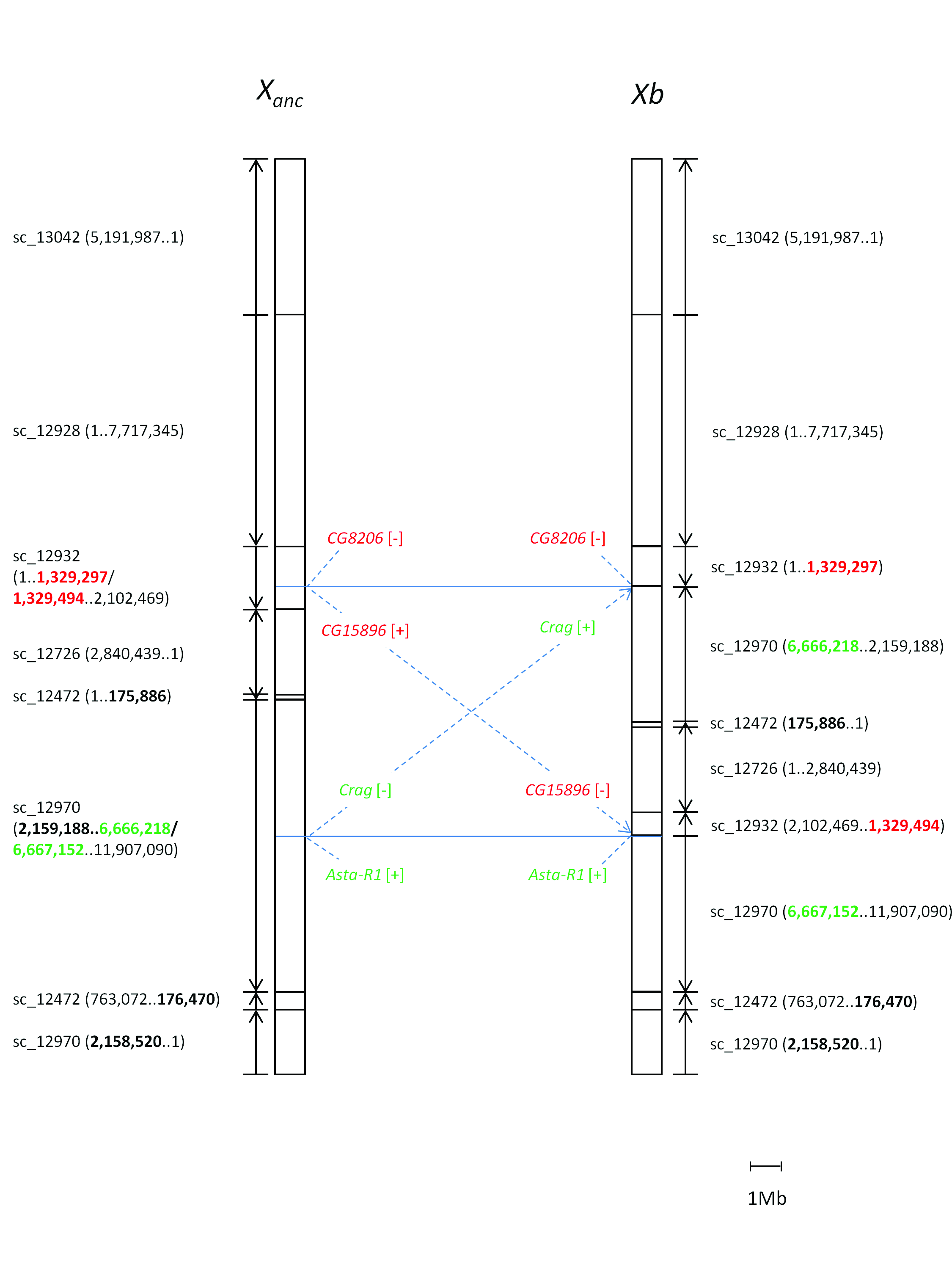

Supplement: Supplementary Data [file evy239_supp.zip › FigS2CMYK.tif]

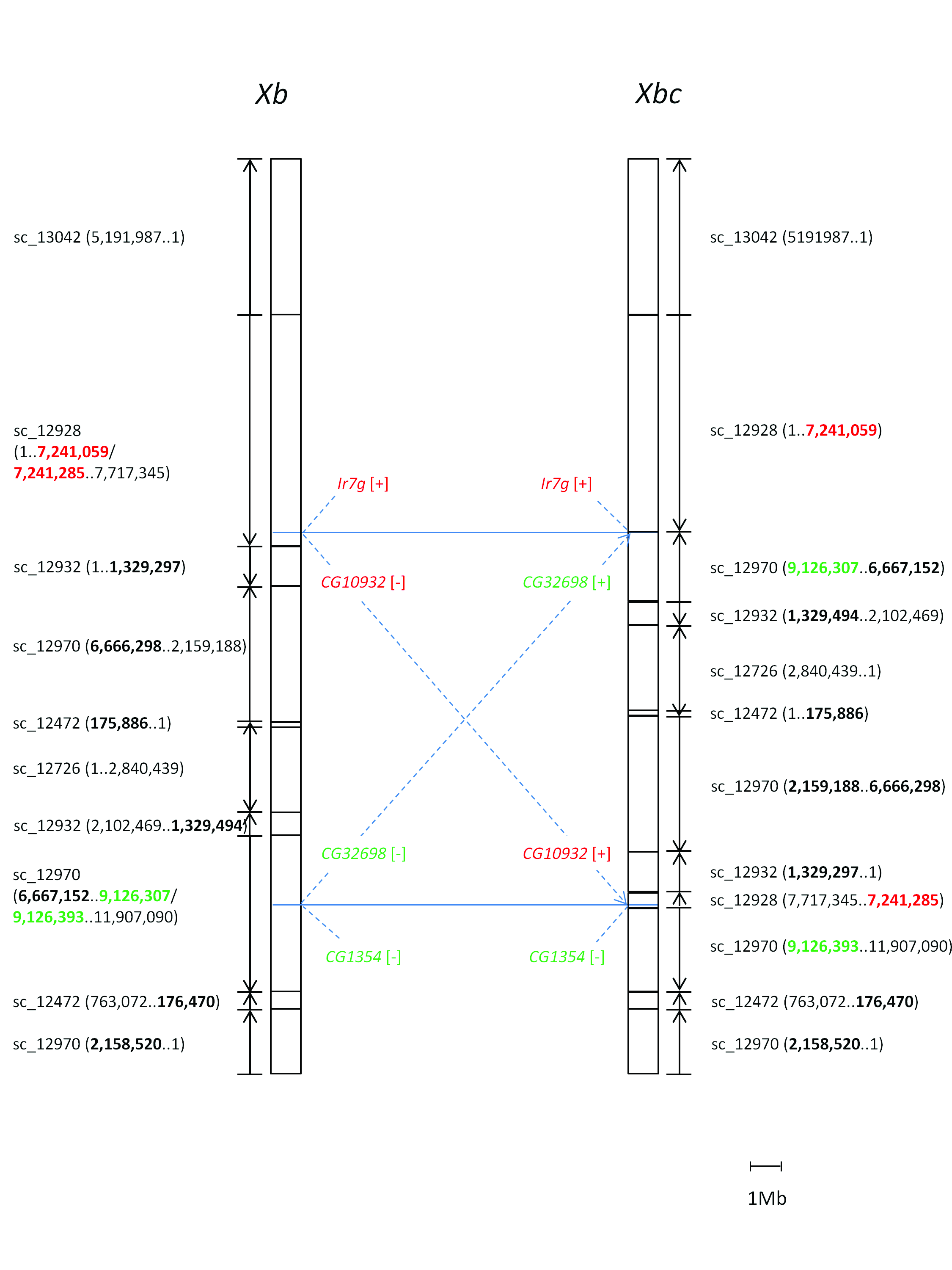

Supplement: Supplementary Data [file evy239_supp.zip › FigS3CMYK.tif]

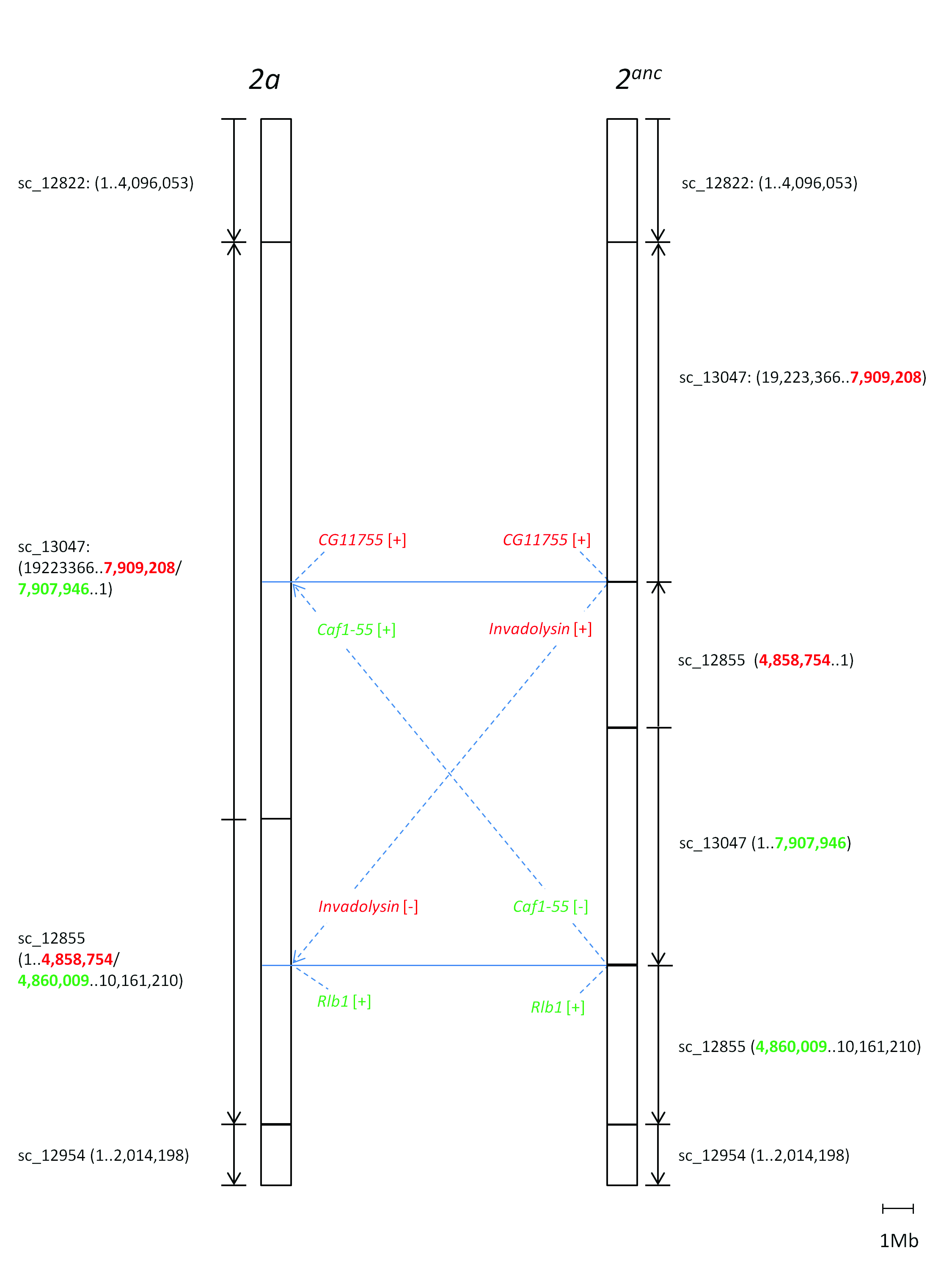

Supplement: Supplementary Data [file evy239_supp.zip › FigS4CMYK.tif]

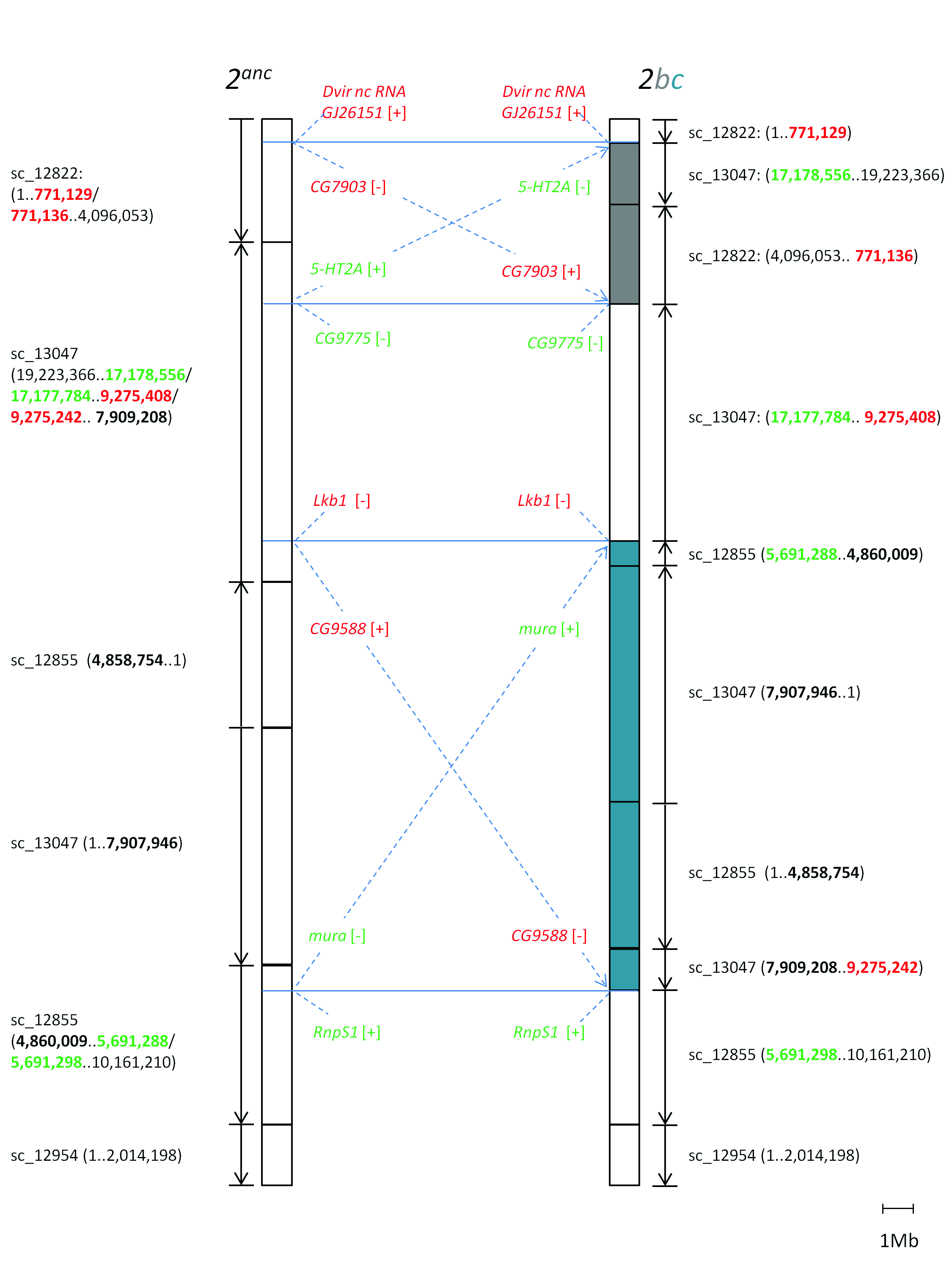

Supplement: Supplementary Data [file evy239_supp.zip › FigS5CMYK.tif]

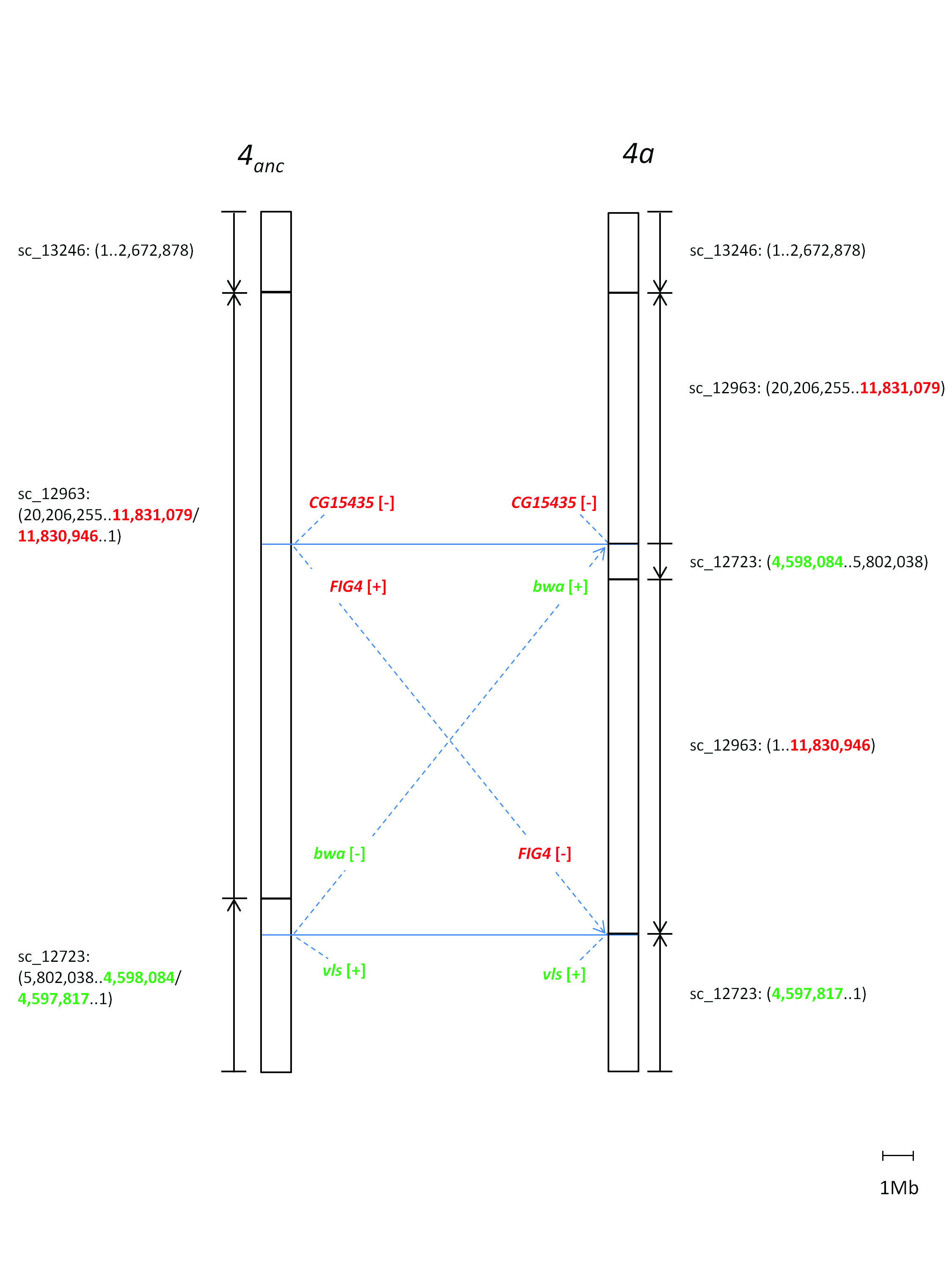

Supplement: Supplementary Data [file evy239_supp.zip › FigS6CMYK.tif]

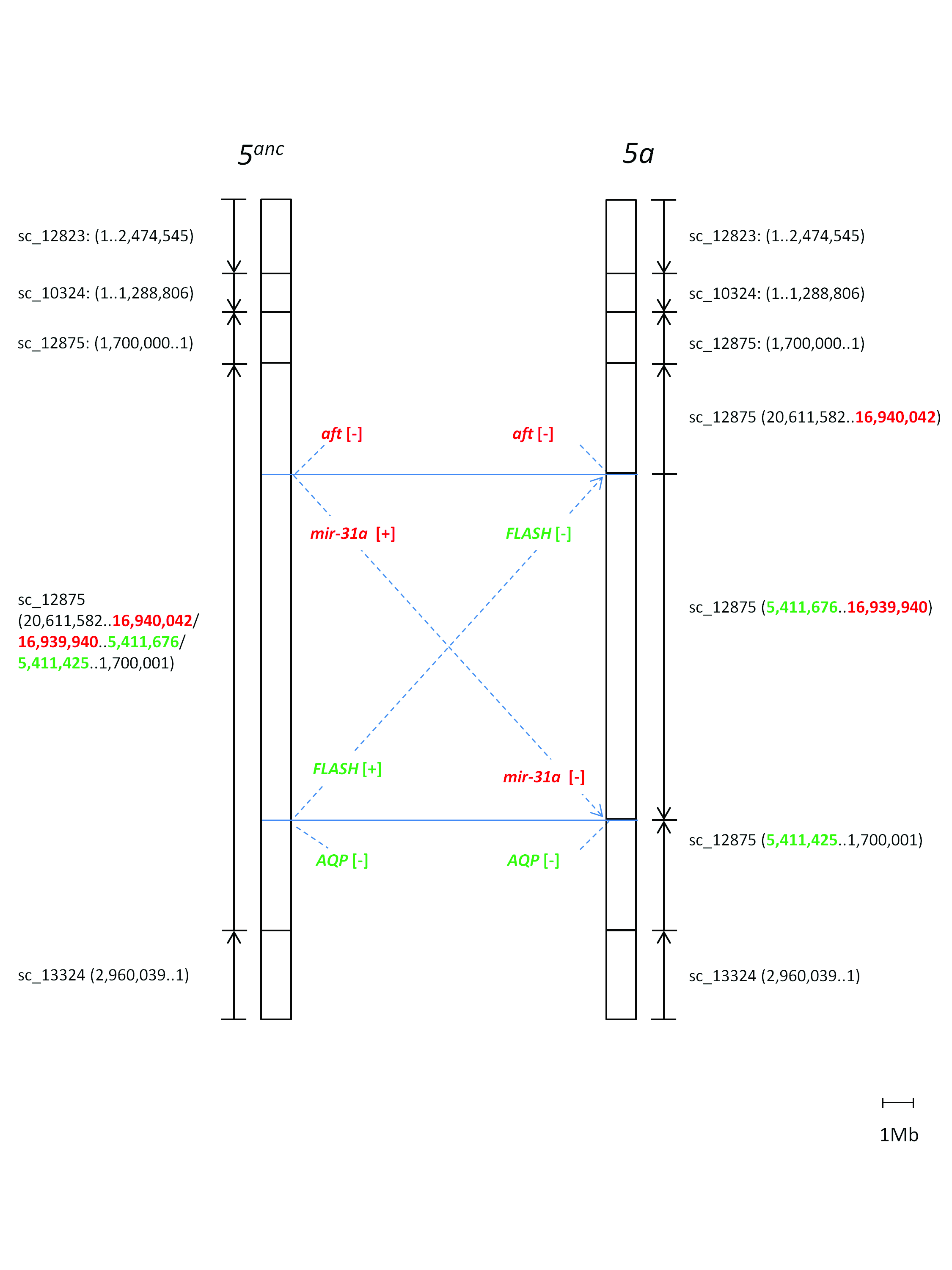

Supplement: Supplementary Data [file evy239_supp.zip › FigS7CMYK.tif]

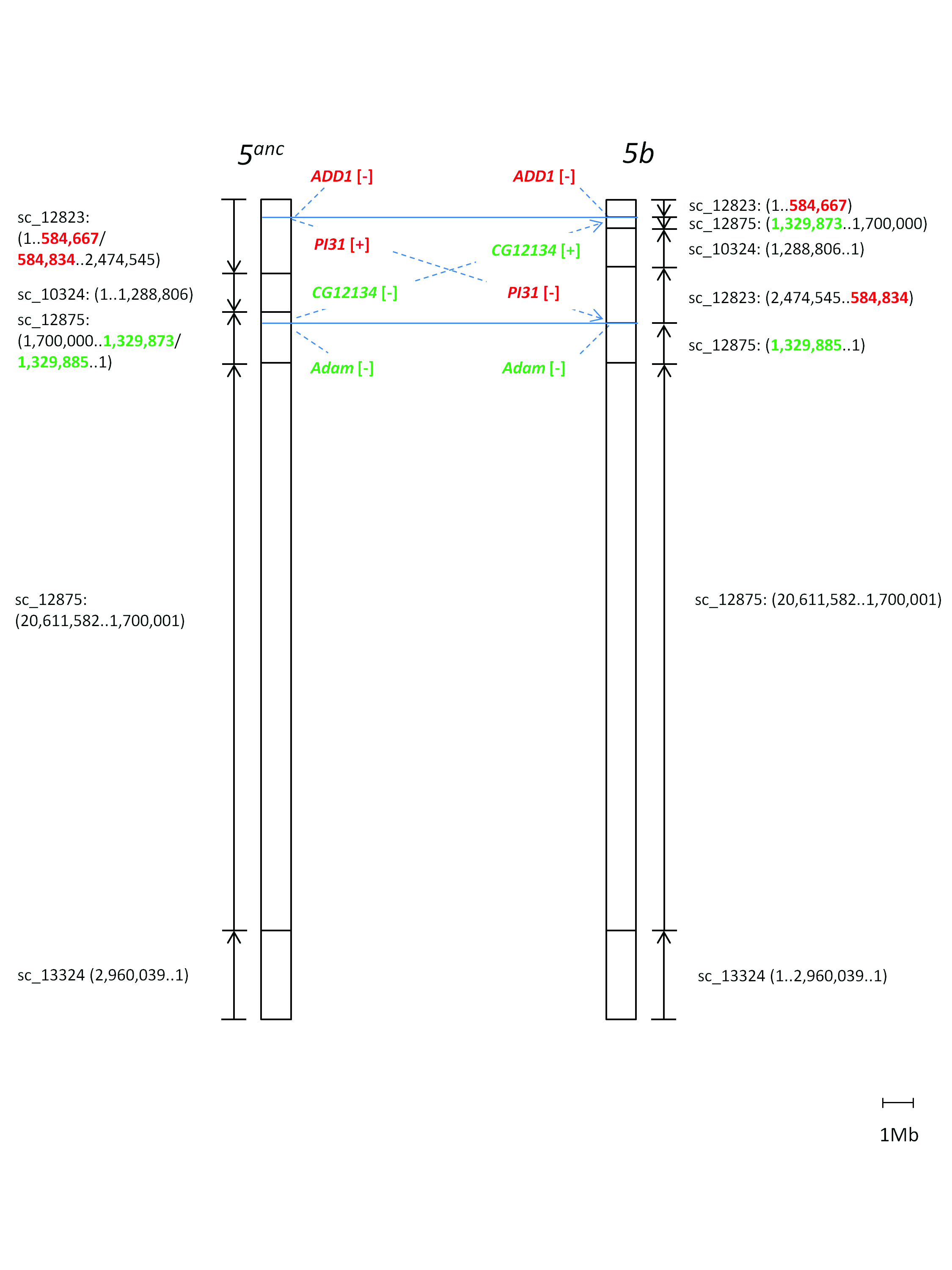

Supplement: Supplementary Data [file evy239_supp.zip › FigS8CMYK.tif]
